# Access to Chiral Diamine Derivatives through Stereoselective Cu-Catalyzed Reductive Coupling of Imines and Allenamides

**DOI:** 10.1021/acs.joc.0c02971

**Published:** 2021-03-16

**Authors:** Toolika Agrawal, Robert T. Martin, Stephen Collins, Zachary Wilhelm, Mytia D. Edwards, Osvaldo Gutierrez, Joshua D. Sieber

**Affiliations:** † Department of Chemistry, Virginia Commonwealth University, 1001 West Main Street, Richmond, Virginia 23284-3208, United States; ‡ Department of Chemistry and Biochemistry, University of Maryland, College Park, Maryland 20742, United States

## Abstract

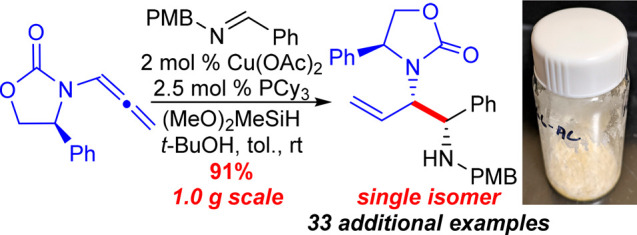

Chiral 1,2-diamino compounds are important building blocks in organic chemistry for biological applications and as asymmetric inducers in stereoselective synthesis that are challenging to prepare in a straightforward and stereoselective manner. Herein, we disclose a cost-effective and readily available Cu-catalyzed system for the reductive coupling of a chiral allenamide with *N*-alkyl substituted aldimines to access chiral 1,2-diamino synthons as single stereoisomers in high yields. The method shows broad reaction scope and high diastereoselectivity and can be easily scaled using standard Schlenk techniques. Mechanistic investigations by density functional theory calculations identified the mechanism and origin of stereoselectivity. In particular, the addition to the imine was shown to be reversible, which has implications toward development of catalyst-controlled stereoselective variants of the identified reductive coupling of imines and allenamides.

## Introduction

**Figure 1:**
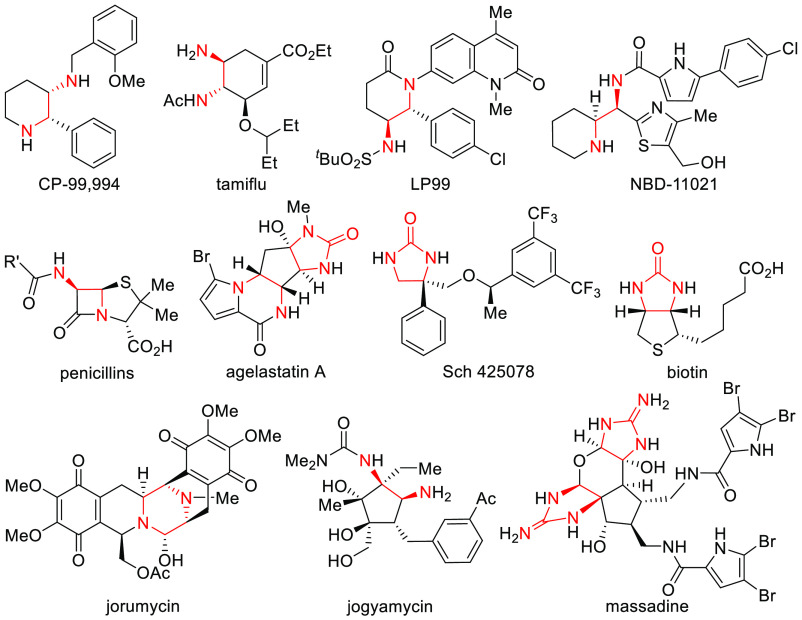
Selected examples of chiral 1,2-diamine- and urea-derived biologically active molecules.

Chiral vicinal diamines are extremely valuable and important motifs in organic chemistry that are exploited by both nature and the pharmaceutical industry for their biological activities,^1,2^ and in stereoselective organic synthesis as powerful chiral inducers through application as organocatalysts,^3^ chiral ligands^4^ for transition metal catalyzed reactions, and as chiral auxiliaries.^5^ For example, a variety of biologically active pharmaceuticals and natural products are given in Figure 1 possessing either the chiral 1,2-diamino-fragment or its corresponding urea form.^2^ Representative therapeutics being developed for the treatment of important human diseases include antibiotics (penicillin,^2e^ jogyamycin^2j^), anticancer compounds (cisplatin derivatives,^6^ LP99^2c^), HIV protease inhibitors (NBD-11021^2d^), NK_1_-antagonists^7^ (CP-99,994;^2a^ Sch425078^2g^) for central-nervous-system (CNS) related diseases and rheumatoid arthritis, and influenza (tamiflu).^2b^

**Scheme 1:**
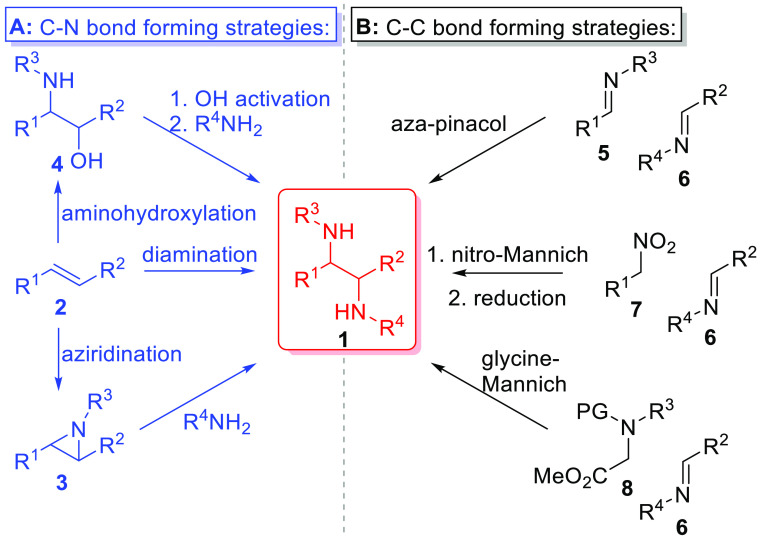
Synthetic Strategies toward the Synthesis of 1,2-Diamines

Due to the biological and synthetic value of chiral 1,2-diamines, stereoselective methods for their preparation are an important endeavor in organic chemistry.^1a,1c,1d,8^ Potential synthetic options to access the chiral vicinal diamine moiety can be envisioned to occur either by formation of the two C–N bonds starting from unsaturated hydrocarbons (**2**, Scheme 1A) or through direct C–C bond formation between C1 and C2 of the 1,2-diamine from two *N*-substituted reagents (Scheme 1B).^1a,1c,1d,8^ Using a C–N bond forming approach (Scheme 1A), diamination may be achieved by forming both C–N bonds at the same time,^8,9^ or sequentially through either aziridination^10^ followed by ring-opening with an amine nucleophile^1a,1c,1d,11^ or through aminohydroxylation^12^ followed by alcohol activation and amine substitution.^1a−1d^ While direct catalytic 1,2-diamination of **2** represents an ideal strategy for diamine synthesis, the amino-groups added across the π-system are typically identical leading to the formation of diamines with identical substituents (i.e., R^3^ = R^4^ in **1**),^8,9^ and a recent approach employing electrochemistry^9b^ suffers from potentially forming high-energy/explosive diazocompounds^13^ en route to the desired diamines. Additionally, the aziridination/ring-opening strategy can suffer from poor stereoselectivity in the aziridination step and regiochemistry issues in the subsequent opening step, while the aminohydroxylation route requires regiocontrol in the aminohydroxylation step followed by additional transformations to convert **4** to the desired diamine. Alternatively, synthesis of **1** through C–C bond formation can be achieved through aza-pinacol coupling of two imines,^14^ nitro-Mannich,^15^ or glycine-Mannich^16^ reactions (Scheme 1B). Typical aza-pinacol coupling protocols only afford symmetrical diamines through homocoupling of a single imine; however, recent photoredox strategies^14h−14l^ enabling the generation of α-aminoradicals^17^ from amines have enabled cross-selective coupling of imines and *N*-methylamines.^14j−14l^ Furthermore, nucleophilic additions to imines using α-aminoanion derivatives^18^ from nitroalkanes (**7**)^15^ or protected glycines (**8**)^16^ offer another entry into the diamine core **1**.

**Scheme 2:**
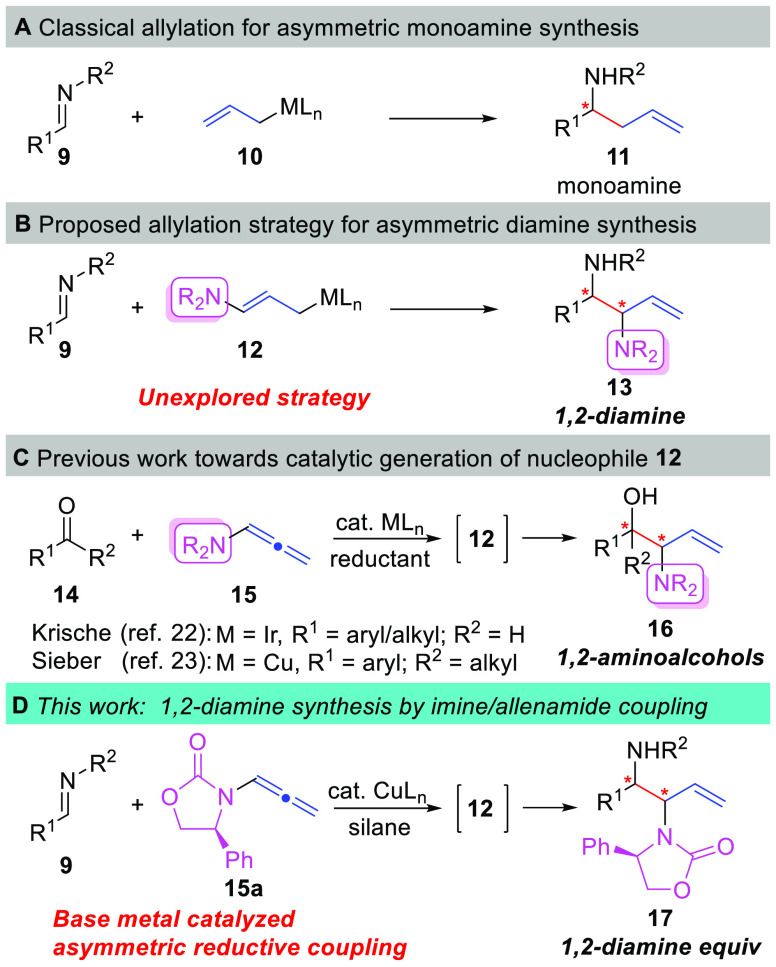
Proposed Allylation Strategy toward the Synthesis of 1,2-Diamines

In regards to chiral amine synthesis, asymmetric allylation of imines using allyl organometallic nucleophiles (**10**) by direct addition or through catalyst control has been an area of intense research in organic chemistry (Scheme 2).^19^ The chiral allylamine products (**11**) are highly valuable in the context of the synthesis of complex amine-containing organic compounds because of the high versatility of the olefin functional group present within **11**. Substituted allylorganometallic reagents (e.g., **12**) allow for increased molecular complexity by introducing two stereocenters in the allyl addition reaction (e.g., **13**, Scheme 2B). Therefore, we envisioned that use of an amino-substituted allyl reagent **12** in addition reactions with imine electrophiles would be a powerful strategy to prepare 1,2-diamines (**13**) with differential substitution patterns on nitrogen and containing an olefin motif for further functional group manipulations. Surprisingly, only a single example of such a strategy for the preparation of 1,2-diamines has been reported, which employs a lithiated derivative of **12** (M = Li) with chiral *tert*-butanesulfinimide derived aldimines affording products in moderate yields with mixtures of branched and linear allylation products.^20^ In contrast, amino-substituted allyl reagents **12** have been used in reactions employing carbonyl electrophiles to provide 1,2-aminoalcohols (**16**).^21−23^ Recently, the Krische^22^ group and our own lab^23^ have developed reductive coupling^24,25^ procedures for the catalytic generation of amino-substituted allyl reagents **12** and have studied their reactions with carbonyl electrophiles (Scheme 2C). These techniques represent orthogonal methodologies whereby the Krische^22a^ system employs a chiral Ir-catalyst and processes aldehyde electrophiles using an achiral allenamide (**15**), while our work utilizes a Cu-catalyst and a chiral allenamide (**15a**) for reactions using ketone electrophiles.^23^ Based on our success in the stereoselective Cu-catalyzed reductive coupling of ketones and chiral allenamides to afford branched chiral 1,2-aminoalcohols **16**(23a) or the corresponding linear products,^23b^ and the lack of literature data for imine allylation reaction utilizing amino-substituted allylic nucleophiles, we began to investigate the reaction of allenamide **15a** with imine electrophiles **9** for the stereoselective synthesis of chiral 1,2-diamine synthons **17** (Scheme 2D). The results of these studies leading to the identification of a practical and highly stereoselective synthesis of diamine synthons **17** using Cu-catalyzed reductive coupling are disclosed herein.

## Results and Discussion

### Reaction Optimization

**Scheme 3:**
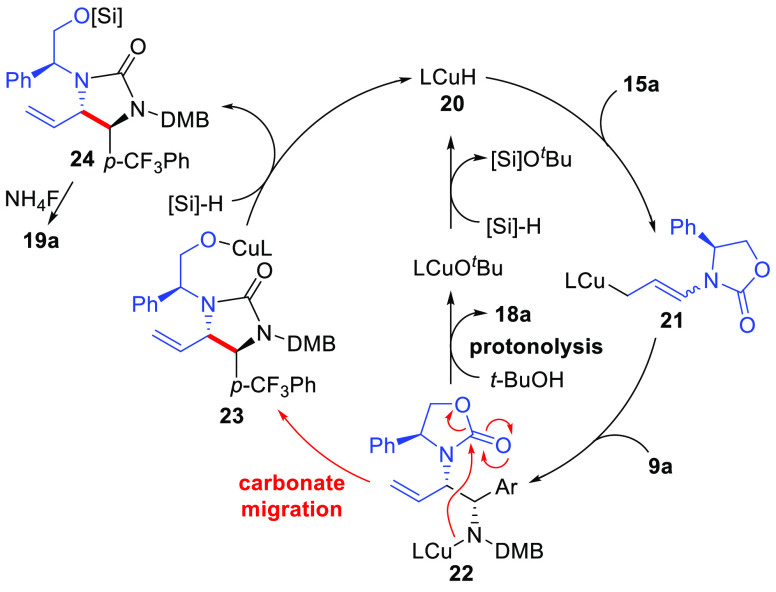
Proposed Reaction Catalytic Cycle

To investigate the proposed Cu-catalyzed reductive coupling of imines and allenamides, initial studies examined the ligand effect when employing DMB-protected imine **9a** with chiral allenamide **15a** in the reaction (Table 1). The phenyl-derived Evans oxazolidinone of allenamide **15a** was specifically targeted due to its low-cost and high-availability,^26^ and because it allows for more deprotection options of the desired diamine products over other alkyl-substituted oxazolidinones (i.e., hydrogenolysis). The DMB-group of the aldimine was employed due to its acid lability to allow for chemoselective differentiation of the two amine protecting groups in the final products (**18a**/**19a**). Gratifyingly, a variety of phosphine ligands (entries 1–7) afforded urea product **19a** presumably resulting from migration of the carbamate carbonyl (Scheme 3), whereas an *N*-heterocyclic carbene (NHC) ligand provided poor conversion (entry 8). In all cases, a single diastereomer of product was obtained as determined by ^1^H NMR spectroscopy of the unpurified reaction mixture. Notably, the bidentate phosphine dcpe that has been utilized previously in Cu-catalyzed reductive coupling of *C*-substituted allenes and imines^25c^ afforded only a moderate yield with a substantial amount of unreacted imine (20%, entry 1). Monodentate phosphine ligands (entries 2–7) worked well with the exception of sterically demanding ligands that afforded poor conversion of the imine (entries 3, 4). Ultimately, the use of PCy_3_ as ligand afforded the highest yield of **19a** in the reaction (entry 2). Use of solvents other than toluene in the reaction (entries 9–12) offered no improvements. Finally, addition of 2 equiv of *t*-BuOH to the reaction led to the exclusive formation of **18a** in excellent yield and diastereoselectivity.

**Table 1:**
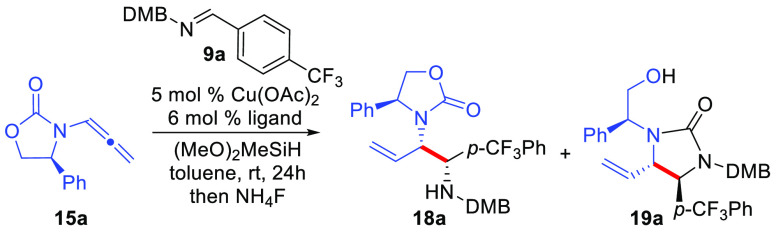
Ligand Optimization for the Reductive Coupling Using **15a**^a^

Entry	Ligand	%Yield **18a**^b^	%Yield **19a**^b^	% **9a**^b^
1	dcpe	<5	58	20
2	PCy3	<5	86	<5
3	P(adam)_3_	<5	28	31
4	XPhos	<5	22	38
5	P(NMe_2_)_3_	<5	66	<5
6	P(OEt)_3_	<5	60	<5
7	(PhO)_2_PNMe_2_	<5	66	<5
8	SIMes	<5	8	67
9^c^	PCy3	<5	51	<5
10^d^	PCy3	<5	54	<5
11^e^	PCy3	<5	52	21
12^f^	PCy3	<5	31	14
13^g^	PCy3	90	<5	<5

a 129 mg (0.400 mmol) **9a**, 96.6 mg (0.480 mmol) **15a**, 5 mol % Cu(OAc)_2_, 6 mol % ligand, and 1.0 mL of toluene. A single diastereomer of product was obtained in all cases by analysis of the unpurified reaction mixture by ^1^H NMR spectroscopy. See the Supporting Information for further details.

b Yield determined by ^1^H NMR spectroscopy on the unpurified reaction mixture using dimethylfumarate as analytical standard.

c Reaction performed in MTBE.

d Reaction performed in dioxane.

e Reaction performed in CH_2_Cl_2_.

f Reaction performed in THF.

g Performed using 2.0 equiv of *t*-BuOH as additive. DMB = 2,4-dimethoxybenzyl.

An initial working hypothesis to understand the difference in product selectivity between the formation of urea **19a** in the absence of *t*-BuOH versus the exclusive formation of diamino-derivative **18a** when *t*-BuOH was used as an additive is given in Scheme 3. Regioselective hydrocupration of allenamide **15a** by the LCuH^23,25c^ catalyst **20** initially is expected to afford substituted linear allylcopper reagent **21** that may undergo *E*/*Z* isomerization through σ–π–σ equilibration prior to reaction with the imine electrophile. Then, diastereoselective reaction of intermediate **21** with imine **9a** provides Cu-amide intermediate **22**. To afford product **18a** from **22**, direct silylation of the amine by the silane must occur to regenerate the LCuH catalyst **20**; however, this step is expected to be slow due to the weak strength of the N–Si bond (BDE ≈ 104 kcal/mol).^27^ Due to the strong basicity of the *N*-anion in **22**, intramolecular attack of the oxazolidinone carbonyl may occur competitively to provide **23** containing an O–Cu bond that should more easily silylate due to the high bond strength of the O–Si bond (BDE ≈ 190 kcal/mol)^28^ affording urea **24** and regenerating the LCuH catalyst (**20**). Alternatively, when *t*-BuOH is present, protonation of the Cu–N bond of **22** by *t*-BuOH to afford product **18a** directly and generate LCuO^*t*^Bu is thermodynamically favorable based on the p*K*_a_ values for a secondary amine (pyrrolidine: ∼44)^29^ vs *t*-BuOH (32) and supported by DFT calculations (*vide infra*).^30^ The LCu-O^*t*^Bu intermediate can then undergo silylation to regenerate the LCuH catalyst **20**. The role of alcohol additives to facilitate catalyst turnover by protonation of Cu–N intermediates has been documented previously.^25c,31^ Sterically hindered alcohols such as *t*-BuOH have been shown to be preferred since the rate of competitive protonation of the Cu–H catalyst is reduced with bulky alcohols.^31^

**Scheme 4:**
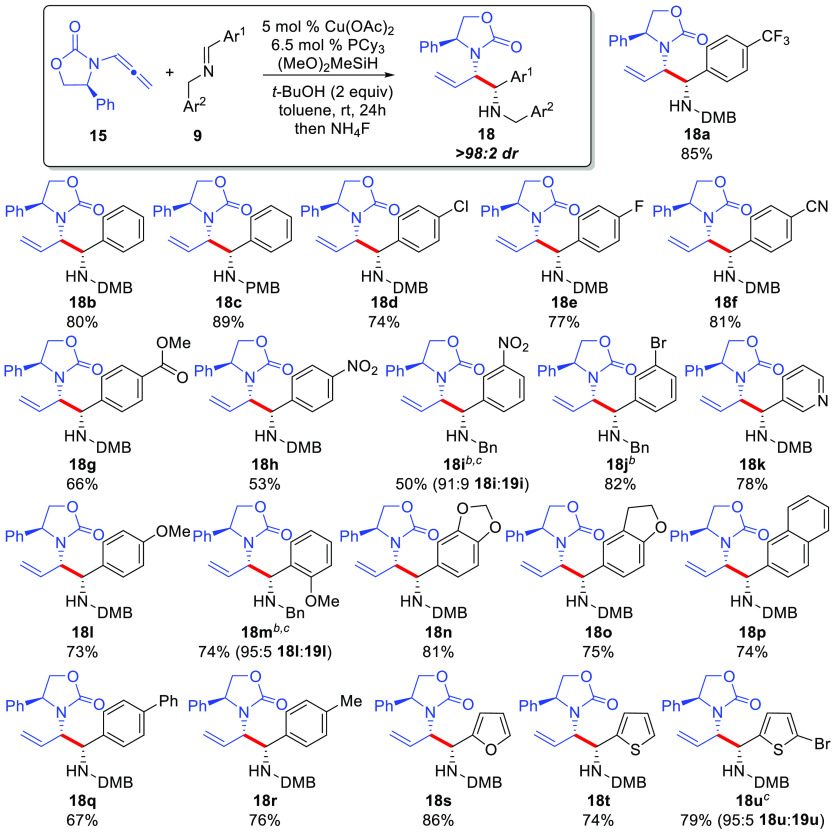
Imine Generality in the Cu-Catalyzed Reductive Coupling To Access 1,2-Diamino Synthons **18** Conditions: **9** (0.400 mmol), **15a** (96.6 mg, 0.48 mmol), Cu(OAc)_2_ (5 mol %), PCy_3_ (6.5 mol %), *t*-BuOH (76 μL, 0.80 mmol), Me(MeO)_2_SiH (99 μL, 0.80 mmol), and 1.0 mL of toluene, rt 24 h followed by treatment with NH_4_F/MeOH. See the Supporting Information for more details. A single diastereomer of product was obtained in all cases by analysis of the unpurified reaction mixture by ^1^H NMR spectroscopy. Yields represent isolated yield. Reaction performed at 65 °C. Isolated as an inseparable mixture of **18** and urea **19**.

Next, the substrate scope of the Cu-catalyzed reductive coupling reaction using *t*-BuOH as additive to provide branched diamino-derived products **18** was examined (Scheme 4). In all cases, a single diastereomer (the (*S,S,S*)-diastereomer) of product was obtained as determined by analysis of the unpurified reaction mixture by ^1^H NMR spectroscopy. In general, a wide variety of imines could be employed in the reaction in good to excellent yields. Electron-deficient (**18a** – **18k**) and electron-rich (**18l**–**18o**) aryl groups both performed well in the reaction. Heterocyclic imines (**18k**, **18s**–**18u**) and *C*-substituted arenes (**18p**–**18r**) were also well tolerated. Finally, a sterically demanding imine (**18m**) or a *m*-NO_2_Ph group (**18i**) required heating at 65 °C to afford good reactivity. Use of an aliphatic aldimine (i.e., Ar^1^ = Me) did not provide any desired products.

Initial analysis of the substrate scope for the urea-forming Cu-catalyzed reductive coupling reaction employing DMB-substituted imines in the absence of *t*-BuOH proved to be less general than the analogous reaction conducted with *t*-BuOH as the additive. In these problematic cases, a poor yield of desired product was obtained even at 65 °C; however, the imine remained while the allenamide had been consumed. As a result, the effect of the *N*-substituent of the imine electrophile was examined to improve the efficiency of the reaction to the desired product (Table 2). As an example, the 2-naphthyl *N*-DMB-imine (**9pa**) afforded a poor yield in the desired reaction (entry 1). A strong influence on reactivity and the electronic character of the aryl group (Ar^2^) of the imine was found (entries 1–5). Use of an electron-poor aryl group (entry 5) afforded the best reaction yield; however, a simple benzyl group also provided good reactivity (entry 3). As a result, for problematic DMB-derived imines, the reactivity can be improved by utilizing PMB, Bn, or *p*-CF_3_-benzyl as the *N*-substituent on the aldimine.

**Table 2:**
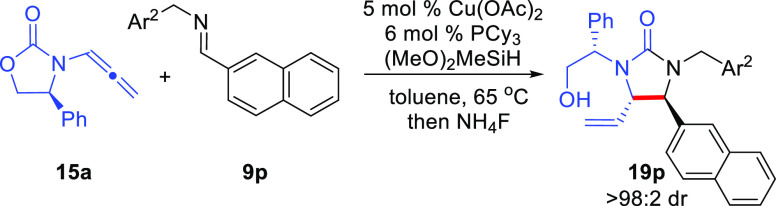
Effect of Imine *N*-Substitution on Reactivity^a^

Entry	Ar^2^	% yield^b^
1	2,4-dimethoxyphenyl (**9pa**)	17 (**19pa**)
2	4-methoxyphenyl (**9pb**)	58 (**19pb**)
3	Phenyl (**9pc**)	70 (**19pc**)
4	4-fluorophenyl (**9pd**)	71 (**19pd**)
5	4-trifluoromethylphenyl (**9pe**)	79 (**19pe**)

a Conditions: **9p** (0.400 mmol), 106 mg (0.480 mmol) of **15a**, 5 mol % Cu(OAc)_2_, 6 mol % PCy_3_, 99 μL (0.80 mmol) of (MeO)_2_MeSiH, and 1.0 mL of toluene. A single diastereomer of product was obtained in all cases by analysis of the unpurified reaction mixture by ^1^H NMR spectroscopy.

b Yield determined by ^1^H NMR spectroscopy on the unpurified reaction mixture using dimethylfumarate as analytical standard.

Based on the results from Table 2, the substrate scope for the urea-forming Cu-catalyzed reductive coupling reaction in the absence of *t*-BuOH was investigated using this new knowledge (Table 3). DMB-substituted imines could be employed in good yields affording single diastereomers of product at room temperature when Ar^1^ was a simple phenyl group (**19b**), heterocyclic (**19k**, **19s**, **19t**), or substituted at the *para*-position with an electron-donating group (**19l**, **19n**) or an electron-withdrawing group (**19a**). However, reactions employing imines containing halogenated arenes or more sterically demanding aryl groups were not successful utilizing the *N*-DMB derived imine and instead required heating and the use of either an *N*-Bn or an *N*-CH_2_-*p*-CF_3_Ph group on the aldimine (see **19de**, **19ee**, **19j** and **19m**, **19pe**, respectively).

**Table 3:**
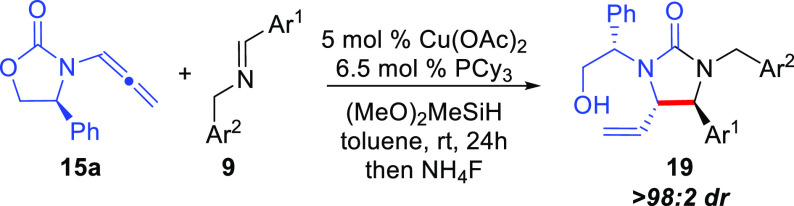

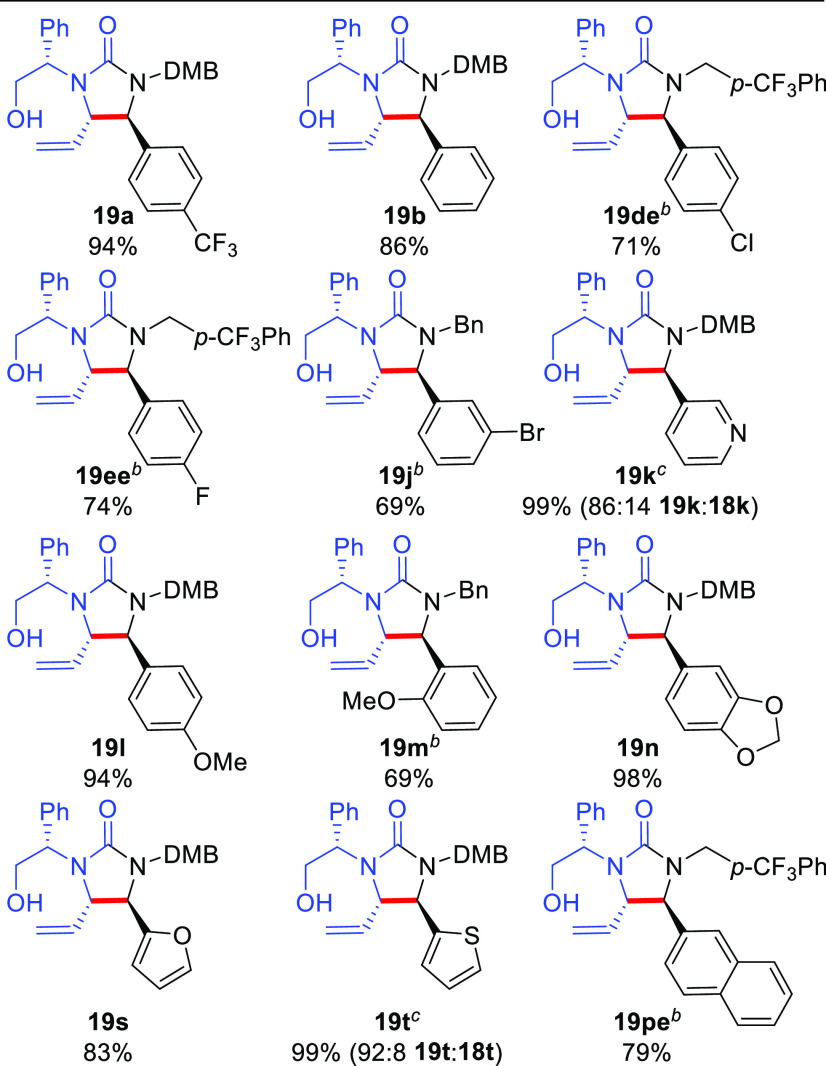
Imine Generality in the Cu-Catalyzed Reductive Coupling To Access Chiral Ureas^a^

a Conditions: **9** (0.40 mmol), **15a** (96.6 mg, 0.48 mmol), Cu(OAc)_2_ (5 mol %), PCy_3_ (6.5 mol %), Me(MeO)_2_SiH (99 μL, 0.80 mmol), and 1.0 mL of toluene, rt 24 h followed by treatment with NH_4_F/MeOH. See the Supporting Information for more details. A single diastereomer of product was obtained in all cases by analysis of the unpurified reaction mixture by ^1^H NMR spectroscopy. Yields represent isolated yield.

b Reaction performed at 65 °C.

c Isolated as an inseparable mixture of urea **19** and **18**.

**Scheme 5:**
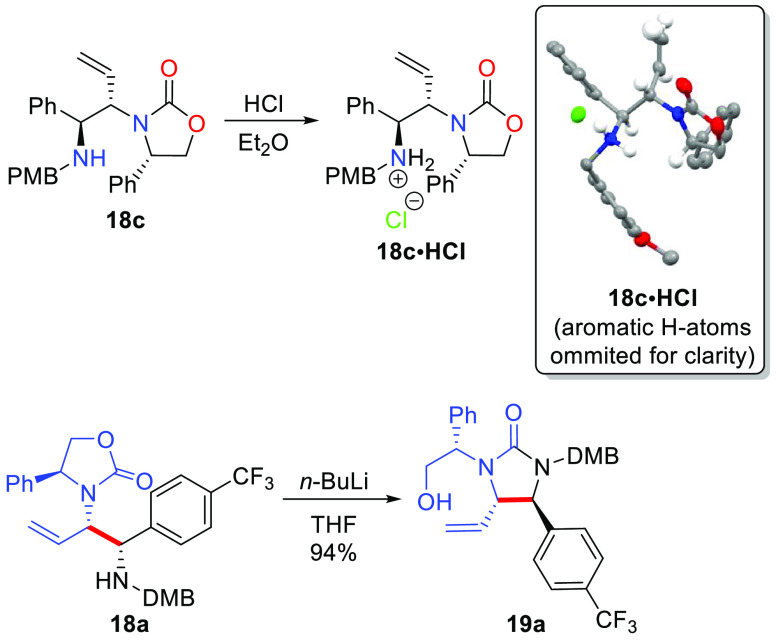
Stereochemistry Determination

Stereochemical assignment of the products obtained in the Cu-catalyzed reductive coupling reaction as the (*S,S,S*)-diastereomer was determined unequivocally by X-ray crystallography (Scheme 5). While the branched products **18** were typically noncrystalline, formation of the HCl-salt of **18c** afforded crystalline material whose structure was determined by single-crystal Xray analysis. Furthermore, conversion of products **18** to the urea **19** could also be achieved after isolation of **18** by subsequent treatment with *n*-BuLi (e.g., **18a** → **19a**). The urea product obtained from this sequence was identical to the material made from the reductive coupling reaction performed in the absence of *t*-BuOH by NMR spectroscopy confirming that the same stereoisomer of product was formed in both reductive coupling processes (i.e., with or without *t*-BuOH as additive).

**Scheme 6:**
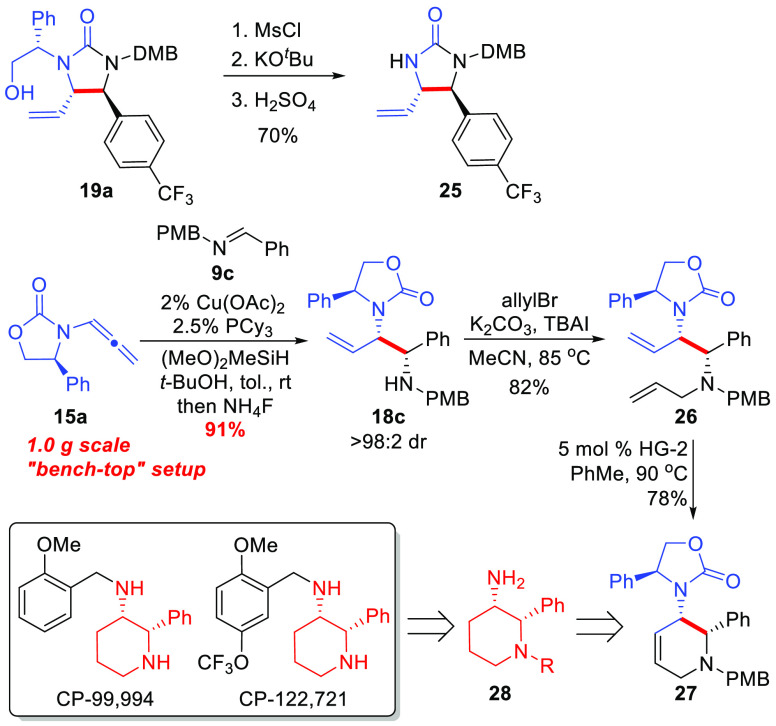
Synthetic Applications

The synthetic utility of the reaction products obtained in the allenamide/imine reductive coupling reaction is highlighted in Scheme 6. The phenethyl group of urea **19a** derived from the Evans oxazolidinone of the allenamide starting material could be cleaved in a three-step sequence consisting of alcohol activation (MsCl), base induced elimination, and enamide hydrolysis with aqueous acid to provide urea **25** in good overall yield without isolation of intermediates. Furthermore, synthon **27** is a viable intermediate to access chiral aminopiperidine **28** for the preparation of the potent NK-1 inhibitor compounds CP-99,994 and CP-122,721,^2a,7^ which could easily be accessed from reductive coupling product **18c** (Scheme 6). The Cu-catalyzed reductive coupling was scaled to 1.0 g without the need for an inert atmosphere glovebox by performing the reaction on the “benchtop” using standard Schlenk techniques and preparing the (PCy_3_)Cu-catalyst by adding the PCy_3_ as a 20 wt % solution in toluene that is commercially available.^32^ The catalyst loading could be reduced to 2.0 mol % Cu providing **18c** in good yield and excellent diastereocontrol in only 2 h of reaction time. Considering the low cost and high availability of the Cu-precatalyst,^32^ the ligand employed (PCy_3_),^32^ and the chiral allenamide **15a**,^26^ along with the high catalytic activity of this system (2 mol % catalyst loading), the current method represents a highly practical and scalable method for the synthesis of diamino-synthons **18**/**19**. Allylation of **18c** was then carried out using allyl bromide, followed by ring-closing metathesis with the Hoveyda–Grubbs second generation catalyst to provide access to compound **27** as an orthogonally protected aminopiperidine derivative as a single stereoisomer.

### Mechanistic Modeling by DFT Analysis

To shed light onto the mechanism and origin of diastereoselectivity, we used dispersion-corrected DFT calculations (see Supporting Information for details). Specifically, we performed extensive conformational analysis on all intermediates and transition states using the B3LYP-D3 functional and a def2-SVP basis set^33^ with toluene as the solvent using the CPCM solvation model^34^ as implemented in Gaussian16. Further, to refine the energetics and compare methods, single-point calculations using the M06-L functional,^35^ as well as a larger basis set (def2-TZVPP) with B3LYP-D3, which yielded similar energetic profiles, were subsequently performed. For simplicity, only B3LYP-D3/def2-SVP optimization energetics will be discussed in the text. Structures were visualized using CYLview Version 1.0.561.^36^

**Figure 2:**
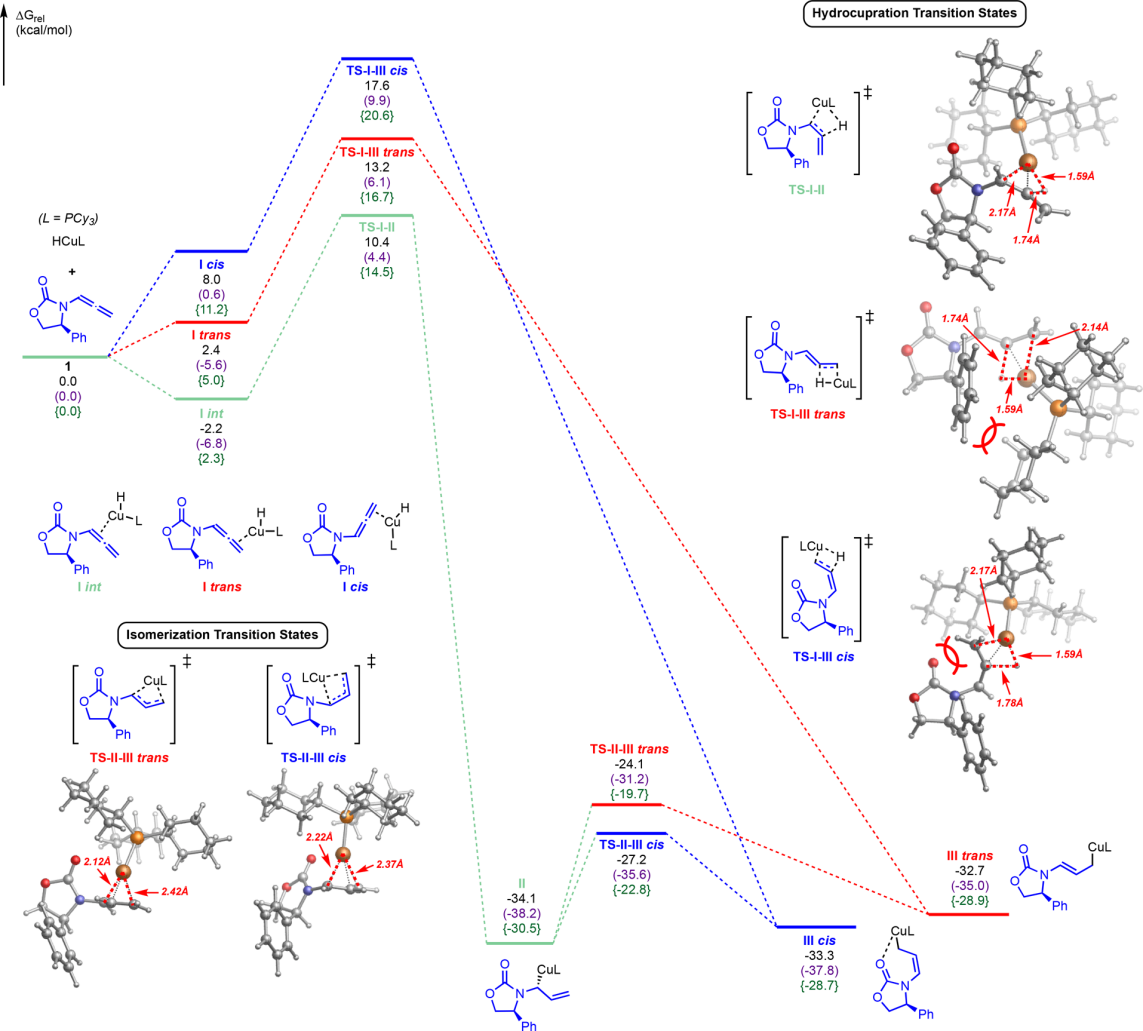
Structures and relative free energies (in kcal/mol, with respect to separate **LCuH** catalyst and reactants) of possible hydrocupration pathways, optimized using B3LYP-D3/def2SVP-CPCM(toluene), M06-L/def2SVP-gas//B3LYP-D3/def2SVP-CPCM(toluene) (in parentheses), and B3LYP-D3/def2TZVPP-gas//B3LYP-D3/def2SVP-CPCM(toluene) {in braces}.

As shown in Figure 2, initial investigations were conducted by first analyzing the hydrocupration of allenamide **15a** with (PCy_3_)CuH as catalyst. Following coordination of the copper and allenamide π-bond, the energetically favored hydrocupration proceeds via **TS–I-II** (barrier of 10.4 kcal/mol with respect to separated **15a** and **LCuH** structures) to form the branched allylcopper species **II**. Presumably this transition state benefits from lack of steric hindrance between the ligand and the chiral auxiliary, which were present in the alternative transition states. Specifically, alternate hydrocupration transition states leading to linear allylcopper species (**TS–I-III*****cis*** and **TS–I-III*****trans***) were found to be much higher in energy by ∼3 kcal/mol for **TS–I-III*****trans*** and by >7 kcal/mol for all other pathways and were therefore not productive.

**Figure 3:**
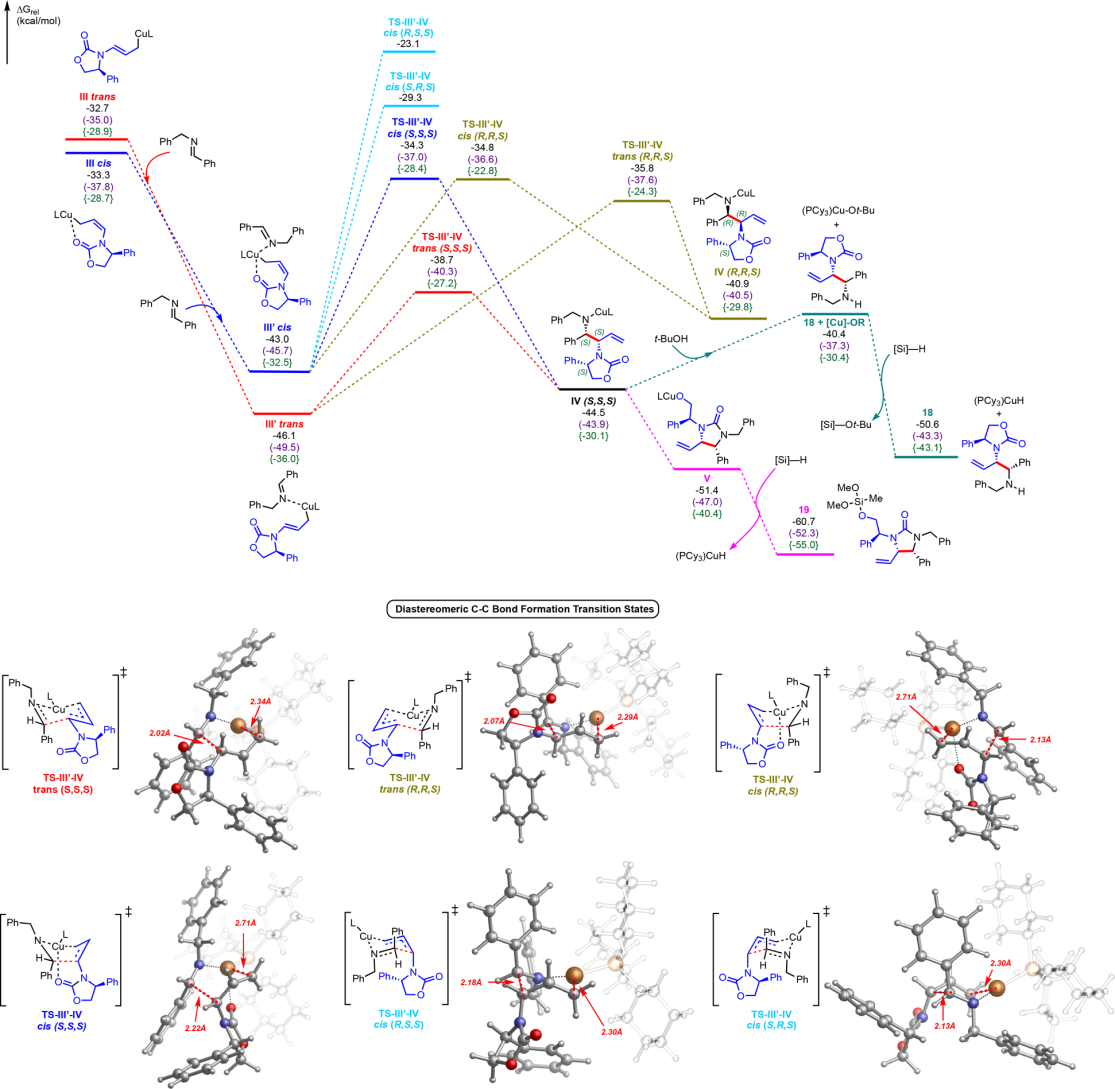
Structures and relative free energies (in kcal/mol, with respect to separate **LCuH** catalyst and reactants) for proposed mechanistic pathway, optimized using B3LYP-D3/def2SVP-CPCM(toluene), M06-L/def2SVP-gas//B3LYP-D3/def2SVP-CPCM(toluene) (in parentheses), and B3LYP-D3/def2TZVPP-gas//B3LYP-D3/def2SVP-CPCM(toluene) {in braces}. Optimized structures of transition states visualized with CYLview are shown (with PCy_3_ ligand faded out for clarity).

In turn, the branched allylcopper intermediate is expected to undergo isomerization to linear allylcopper species by σ–π–σ isomerization. Recently, Buchwald and co-workers reported branched-linear allylcopper isomerizations for a system with a bidentate phosphine ligand^25c^ as well as with a CuH-catalyzed allylation of ketones and dienes.^37^ In our case, it was calculated that the branched allylcopper intermediate **II** can readily isomerize (barrier of only 6.9 and 10.0 kcal/mol via **TS–II-III*****cis*****or TS–II-III*****trans***, respectively) to form the nearly isoenergetic *cis* or *trans* linear allylcopper intermediates (**III*****cis*** and **III*****trans***). Intermediate **III*****cis*** was slightly favorable compared to intermediate **III*****trans*** (by 0.6 kcal/mol), as was the *cis* isomerization transition state (**TS–I-III*****cis*** was favored by 3.1 kcal/mol), presumably due to coordination between the oxazolidinone and the copper (Cu–O bond distance = 2.37 Å). However, upon coordination of the imine to the copper, the *trans* conformation (**III′*****trans***) becomes significantly more favored (as seen in Figure 3), likely due to unfavorable steric hindrance between the imine and the oxazolidinone in the **III′*****cis*** conformation (see Supporting Information for further details).

Next, we focused on the key C–C bond formation steps. As shown in Figure 3, after performing extensive conformational analysis on the subsequent diastereomeric C–C bond forming transition states with the allylcopper intermediates and the imine substrate (see Supporting Information for details), the most favorable pathway for diastereoselective C–C bond formation was identified to proceed from the *trans* linear intermediate **III′*****trans*** through a Zimmerman–Traxler transition state **TS-III′-IV*****trans (S,S,S)*** (barrier of only 7.4 kcal/mol from complexed **III′*****trans*** intermediate) to branched addition product **IV*****(S,S,S)***. Further, in agreement with experiment, the competing diastereomeric transition state **TS-III′-IV*****trans (R,R,S)*** which would lead to the opposite diastereomer was determined to be much higher in energy. Notably, *all C–C bond formation steps from**III′trans**are reversible*, as the branched addition products **IV*****(S,S,S)*** and **IV*****(R,R,S)*** were each uphill in energy (by ∼2 kcal/mol and ∼6 kcal/mol respectively), which can have implications for rational catalyst and reaction design (*vide infra*).

**Figure 4:**
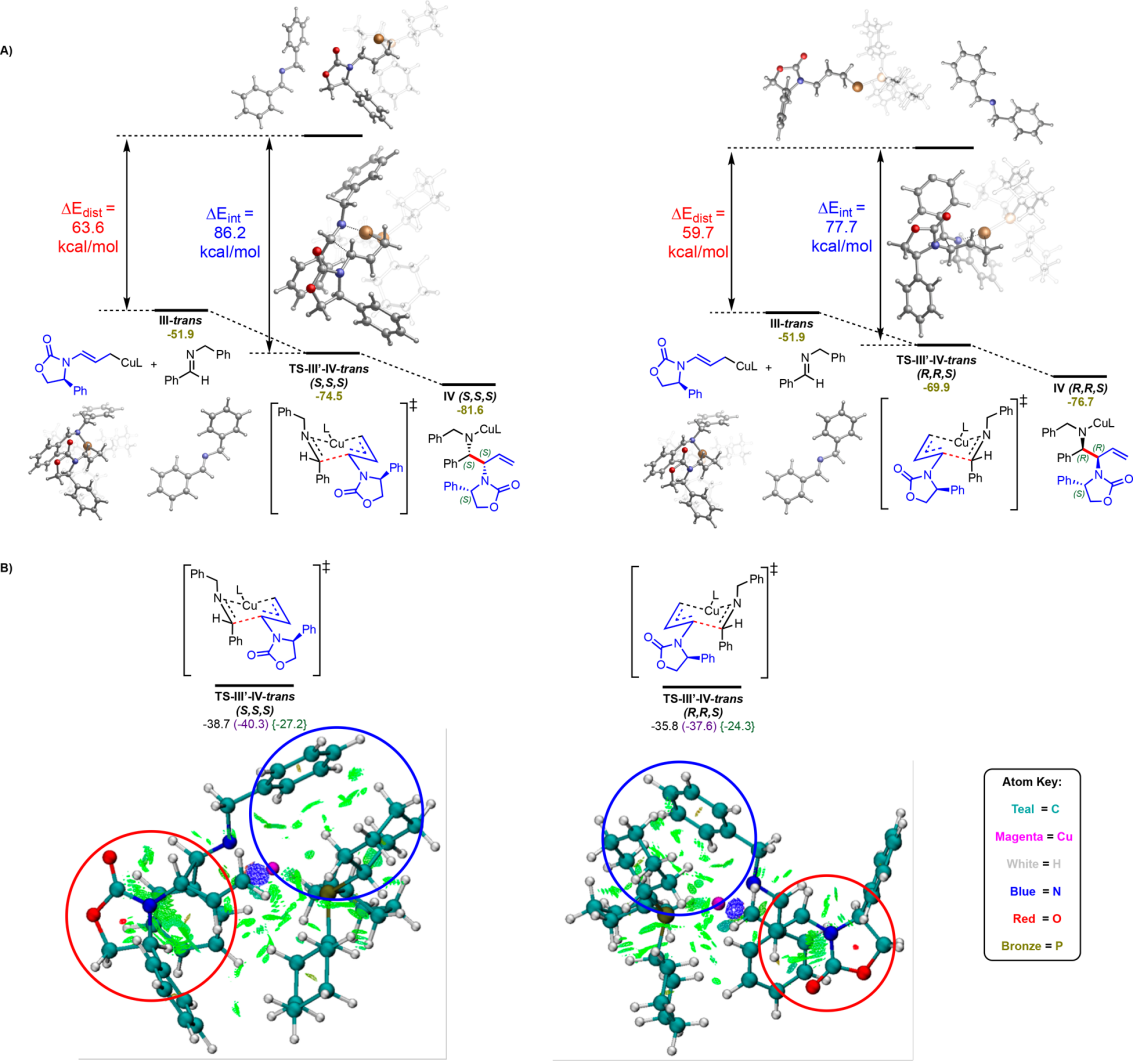
(A) Distortion–Interaction analysis of key diastereomeric C–C bond formation transition states. Electronic energies reported at B3LYP-D3/def2SVP-CPCM(toluene) level of theory. (B) Noncovalent Interaction analysis of key diastereomeric C–C bond formation transition states. Color code for the atoms is shown.

**Figure 5:**
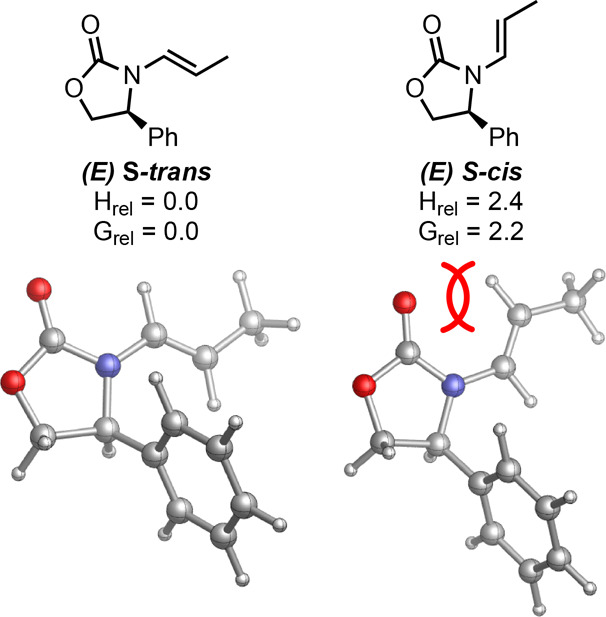
Energetic comparison of s-*trans* and s-*cis* conformations of an (*E*)-enamide system with chiral oxazolidinone, with steric hindrance causing allylic strain highlighted. Structures optimized using B3LYP-D3/def2SVP-CPCM(toluene) (H_rel_ and G_rel_ shown in kcal/mol).

To gain insights into the origin of diastereoselectivity, we performed distortion–interaction and NCI analysis (Figure 4). Overall, comparing the structures of the lowest energy competing diastereomeric transition states **TS-III′-IV*****trans*****(*****S,S,S*****)** and **TS-III′-IV*****trans*****(*****R,R,S*****)** reveals that the structures of these transition states were remarkably similar, with key C–C and C–Cu bond distances differing by no more than 0.05 Å. However, the orientation of the chiral auxiliary is different, as the **TS-III′-IV*****trans*****(*****S,S,S*****)** transition state has the oxazolidinone moiety of the enamide group of the substituted Cu(allyl) ligand in an s-*trans* conformation while the **TS-III′-IV*****trans*****(*****R,R,S*****)** has this group in an s-*cis* conformation that, as shown in Figure 5, leads to a 2.2 kcal/mol energy destabilization. Furthermore, the ground state structures of chiral oxazolidinone-derived enamides are known to favor an s-*trans* conformation.^38^ In addition, distortion–interaction analysis^39^ (Figure 4a) showed that the distortion energy of the **TS-III′-IV*****trans*****(*****S,S,S*****)** transition state was higher than that of the corresponding **TS-III′-IV*****trans*****(*****R,R,S*****)** transition state (by 3.9 kcal/mol). However, the (*S,S,S*) system benefited from much stronger interaction energy (by 8.5 kcal/mol). Overall, this favorable interaction between the imine and allylcopper makes the **TS-III′-IV*****trans*****(*****S,S,S*****)** the favorable diastereomeric transition state. Finally, noncovalent interaction (NCI) analysis (performed using Multiwfn^40^ software and visualized using VMD^41^ software) further supports the presence of favorable interactions in the **TS-III′-IV*****trans*****(*****S,S,S*****)** transition state (Figure 4b). Specifically, in both transition states, there appeared to be favorable C–H···π interactions between the ligand and the benzyl group of the imine (highlighted inside the blue circle). However, comparing the areas in red circles, the **TS-III′-IV*****trans*****(*****S,S,S*****)** system had stronger noncovalent interactions between the oxazolidinone group and the phenyl ring on the imine. This suggests that noncovalent interactions (i.e., between the oxazolidinone moiety and the protecting group) are critical for control of diastereoselectivity. Taken together, these results suggest that both the conformational preference for the s-*trans* geometry about the *N*-enamide group of the substituted Cu(allyl) ligand and favorable noncovalent interactions between the oxazolidinone group and the imine are the major contributing factors for diastereocontrol in these reactions.

Following C–C bond formation, intermediate **IV** serves as a fork between two reaction pathways depending on whether or not there is *t-*BuOH present (as supported by experiments; *vide supra*). Specifically, in the presence of *t-*BuOH, the alcohol can act as a proton source to protonate the Cu–N bond and yield branched product **18**, while the alkoxide binds to the copper. From this, the silane reagent can exchange hydride for the alkoxide group, reforming the catalyst. While this protonation of the amine moiety and concomitant release of the *t-*BuO-CuL was calculated to be initially energetically unfavorable (uphill by ∼4 kcal/mol), the exchange of hydride for the alkoxide was thermodynamically favorable (downhill by ∼10 kcal/mol), rendering this overall process energetically feasible. In the absence of *t-*BuOH, a thermodynamically favorable rearrangement of intermediate **IV** can yield the Cu-alkoxide urea intermediate **V** (∼7 kcal/mol exergonic), which can then readily undergo transmetalation with silane to reform the LCuH catalyst and furnish the silylated product of urea **19**. This mechanistic model is consistent with experimental findings that the presence of *t*-BuOH has a profound effect on the product selectivity (but not diastereoselectivity) of the reaction toward either of the products (*vide supra*).

**Scheme 7:**
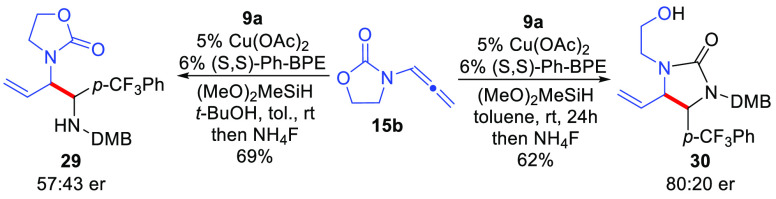
Mechanistic Implications Relevant to Catalyst-Controlled Enantioinduction

As previously noted, computational modeling of the imine addition predicts this step should be reversible. This phenomenon has important impacts for future developments of catalyst controlled enantioselective reactions utilizing a chiral catalyst in conjunction with an achiral allenamide. In this regard, reaction of achiral allenamide **15b** with imine **9a** using (*S*,*S*)-Ph-BPE as a chiral ligand was examined with and without *t*-BuOH as the additive (Scheme 7). Again, branched product **29** was formed as a single diastereomer when *t*-BuOH was present in the reaction, and urea **30** was formed as a single diastereomer in the absence of *t*-BuOH. Separate conversion of **29** to **30** using *n*-BuLi confirmed that the same relative stereochemistry was formed in both reactions. Importantly, **29** and **30** were formed in different enantiopurities (57:43 vs 80:20 er, respectively), *supporting a reversible imine addition step in these reactions*. For example, if imine addition were irreversible, reaction of **15b** with a chiral catalyst to afford the analogous intermediate to **22** (Scheme 3) must be enantiodetermining and requires that urea product **30** formed from rearrangement of the intermediate **22** derivative to have identical enantiopurity to that of **29**. However, when a chiral ligand is employed, rearrangement of the two enantiomers of intermediate **22** may occur at different rates because the transition states will be diastereomeric due to the chirality on the ligand. Therefore, the carbamate migration step may also be enantiodetermining if the imine addition step becomes reversible enabling different enantiomeric ratios to be obtained for a **29**-selective vs a **30**-selective process as was observed.

## Conclusion

In conclusion, a highly stereoselective method for the reductive coupling of imines with a chiral allenamide was developed as a convenient strategy for the asymmetric synthesis of valuable 1,2-diamino synthons. The method employs readily available and cost-effective starting materials^26^ and catalyst (Cu(OAc)_2_/PCy_3_)^32^ and can be performed on the “bench-top” using standard Schlenk techniques without issues. Use of *tert*-butanol as an additive was shown to aid in the amine release and catalyst regeneration to avoid the formation of urea products that are exclusively obtained in the absence of this additive. The oxazolidinone moiety of the final products could be removed chemoselectively without disruption of the pendant terminal alkene, and an orthogonally protected chiral aminopiperidine derivative en route to important biologically active pharmaceuticals was demonstrated. Finally, mechanistic investigations by density functional theory calculations identified the mechanism for stereoselection in these processes as determined from the relative transition state barriers of *N*-substituted allylcopper complexes to the imine electrophile. This C–C bond forming addition step was shown to be reversible by calculation and was experimentally supported by the catalytic asymmetric reaction of a chiral catalyst with an achiral allenamide. These mechanistic insights are important for the development of future asymmetric catalyst-controlled procedures and are currently under further investigation in these laboratories.

## Experimental Section

### General

^1^H NMR spectra were recorded on Bruker 600 MHz spectrometers. Chemical shifts are reported in ppm from tetramethylsilane with the solvent resonance as the internal standard (CDCl_3_: 7.26 ppm). Data are reported as follows: chemical shift, integration, multiplicity (s = singlet, d = doublet, t = triplet, q = quartet, p = pentet, h = hextet, hept = heptet, br = broad, m = multiplet), and coupling constants (Hz). ^13^C NMR spectra were recorded on a Bruker 600 MHz (151 MHz) instrument with complete proton decoupling. Chemical shifts are reported in ppm from tetramethylsilane with the solvent as the internal standard (CDCl_3_: 77.0 ppm). Liquid chromatography was performed using forced flow (flash chromatography) on silica gel purchased from Silicycle. Thin layer chromatography (TLC) was performed on glass-backed 250 μm silica gel F_254_ plates purchased from Silicycle. Visualization was achieved by using UV light, a 10% solution of phosphomolybdic acid in EtOH, or potassium permanganate in water followed by heating. HRMS was collected using a Jeol AccuTOF-DART mass spectrometer using DART source ionization. All reactions were conducted in oven or flame-dried glassware under an inert atmosphere of nitrogen or argon with magnetic stirring unless otherwise noted. Solvents were obtained from VWR as HPLC grade and transferred to septa sealed bottles, degassed by argon sparge, and analyzed by Karl Fischer titration to ensure water content was ≤600 ppm. Me(MeO)_2_SiH was purchased from Alfa Aesar and used as received. Allenamides **15** were prepared in one step as described in the literature.^26^ Aldehydes were purchased from Sigma-Aldrich, Combi-Blocks, TCI America, Alfa Aesar, or Oakwood Chemicals and used as received. Tricyclohexylphosphine and Cu(OAc)_2_ were purchased from the Strem Chemical Company and used as received. All other materials were purchased from VWR, Sigma-Aldrich, Combi-Blocks, or Alfa Aesar and used as received. Imines **9a**,^42^**9b**,^43^**9c**,^44^**9ee**,^45^**9h**,^46^**9i**,^47^**9j**,^48^**9l**,^45^**9m**,^47^**9pb**,^49^**9pc**,^50^ and **9u**(45) were synthesized as described in the literature.

### General Procedure A for the Synthesis of Imines

A 25 mL round-bottom flask equipped with a magnetic stirring bar was charged with aldehyde (6.0 mmol, 1.0 equiv) and dichloromethane (8 mL). Anhydrous magnesium sulfate was added to this solution while stirring followed by 2,4-dimethoxy benzylamine (6.0 mmol, 1.0 equiv) dropwise. The reaction mixture was stirred at room temperature for 12 h under a nitrogen atmosphere. After the reaction is complete the crude reaction mixture was filtered through Celite to remove magnesium sulfate. The filtrate was concentrated *in vacuo* to yield the pure imine, which was stored under nitrogen in the fridge.

#### (E)-1-(4-Chlorophenyl)-N-(2,4-dimethoxybenzyl)methanimine (**9d**)

Following General Procedure A, 4-chloro benzaldehyde (0.84 g, 6.0 mmol), 2,4-dimethoxybenzylamine (1.0 g, 6.0 mmol), magnesium sulfate (2.0 g), and dichloromethane (8 mL) were used. The title compound was obtained as a pale-yellow solid (1.54 g, 89%). Mp −59.5–60.5 °C. ^1^H NMR (600 MHz, CDCl_3_) δ 8.28 (s, 1H), 7.70 (d, *J* = 8.5 Hz, 2H), 7.37 (d, *J* = 8.5 Hz, 2H), 7.21–7.15 (m, 1H), 6.51–6.43 (m, 2H), 4.75 (s, 2H), 3.81 (s, 6H). ^13^C{1H} NMR (151 MHz, CDCl_3_) δ 160.3, 160.2, 158.3, 136.4, 134.9, 130.2, 129.4, 128.8, 119.6, 104.1, 98.5, 58.9, 55.4. HRMS (DART) *m*/*z* calcd for C_16_H_17_ClNO_2_ [M + H]^+^: 290.0948; Found [M + H]^+^: 290.0950.

#### (E)-1-(4-Chlorophenyl)-N-(4-(trifluoromethyl)benzyl)methanimine (**9de**)

Following General Procedure A, 4-chloro benzaldehyde (0.84 g, 6.0 mmol), 4-trifluoromethyl benzylamine (1.05 g, 6.0 mmol), magnesium sulfate (2.0 g), and dichloromethane (8 mL) were used. The title compound was obtained as brown solid (1.5 g, 84%). Mp −39.7–41.3 °C. ^1^H NMR (600 MHz, CDCl_3_) δ 8.39 (s, 1H), 7.80 (dd, *J* = 8.5, 5.6 Hz, 2H), 7.61 (d, *J* = 8.0 Hz, 2H), 7.47 (d, *J* = 7.9 Hz, 2H), 7.12 (t, *J* = 8.6 Hz, 2H), 4.85 (s, 2H). ^13^C{1H} NMR (151 MHz, CDCl_3_) δ 161.2, 143.3, 137.0, 134.4, 129.66 (C–F, ^2^*J* C–F = 33.22 Hz), 129.5, 129.45 (C–F, ^2^*J* C–F = 33.22 Hz), 129.23 (C–F, ^2^*J* C–F = 33.22 Hz), 128.9, 128.1, 126.97 (C–F, ^1^*J* C–F = 273.31 Hz), 125.49 (C–F, ^3^*J* C–F = 3.02 Hz), 125.46 (C–F, ^3^*J* C–F = 3.02 Hz), 125.44 (C–F, ^3^*J* C–F = 3.02 Hz), 125.41 (C–F, ^3^*J* C–F = 3.02 Hz), 125.17 (C–F, ^1^*J* C–F = 273.31 Hz), 123.36 (C–F, ^1^*J* C–F = 273.31 Hz), 121.56 (C–F, ^1^*J* C–F = 273.31 Hz), 64.3. ^19^F NMR (565 MHz, CDCl_3_) δ −108.88. HRMS (DART) *m*/*z* calcd for C_15_H_12_ClF_3_N [M + H]^+^: 298.0610; Found [M + H]^+^: 298.0640.

#### (E)-N-(2,4-Dimethoxybenzyl)-1-(4-fluorophenyl)methanimine (**9e**)

Following General Procedure A, 4-fluorobenzaldehyde (0.742 g, 6.0 mmol), 2,4-dimethoxybenzylamine (1.0 g, 6.0 mmol), magnesium sulfate (2.0 g), and dichloromethane (8 mL) were used. The title compound was obtained as a yellow solid (1.36 g, 84%). Mp −39.7–41.1 °C. ^1^H NMR (600 MHz, CDCl_3_) δ 8.29 (s, 1H), 7.76 (dd, *J* = 8.5, 5.7 Hz, 2H), 7.23–7.16 (m, 1H), 7.08 (t, *J* = 8.6 Hz, 2H), 6.52–6.45 (m, 2H), 4.76 (s, 2H), 3.81 (s, 6H). ^13^C{1H} NMR (151 MHz, CDCl_3_) δ 165.06 (C–F, ^1^*J* C–F = 250.66 Hz), 163.40 (C–F, ^1^*J* C–F = 250.66 Hz), 160.23, 160.21, 158.32, 132.80 (C–F, ^3^*J* C–F = 3.02 Hz), 132.78 (C–F, ^3^*J* C–F = 3.02 Hz), 132.2, 130.15, 130.11, 130.06, 119.84, 115.66 (C–F, ^2^*J* C–F = 22.65 Hz), 115.51 (C–F, ^2^*J* C–F = 22.65 Hz), 104.1, 98.54, 58.85. ^19^F NMR (565 MHz, CDCl_3_) δ −109.87. HRMS (DART) *m*/*z* calcd for C_16_H_17_FNO_2_ [M + H]^+^: 274.1243; Found [M + H]^+^: 274.1269.

#### (E)-4-(2,4-Dimethoxybenzyl iminomethyl)benzonitrile (**9f**)

Following General Procedure A, 4-formyl benzonitrile (0.784 g, 6.0 mmol), 2,4-dimethoxybenzylamine (1.0 g, 6.0 mmol), magnesium sulfate (2.0 g), and dichloromethane (8 mL) were used. The title compound was obtained as a yellow solid (1.59 g, 95%). Mp −54.0–56.2 °C. ^1^H NMR (600 MHz, CDCl_3_) δ 8.32 (s, 1H), 7.84 (d, *J* = 8.0 Hz, 2H), 7.67 (d, *J* = 8.1 Hz, 2H), 7.17 (d, *J* = 9.0 Hz, 1H), 6.51–6.44 (m, 2H), 4.80 (s, 2H), 3.80 (s, 6H). ^13^C{1H} NMR (151 MHz, CDCl_3_) δ 160.4, 159.6, 158.4, 140.3, 132.3, 130.3, 128.6, 119.0, 118.6, 113.7, 104.2, 98.5, 59.1, 55.4. HRMS (DART) *m*/*z* calcd for C_17_H_17_N_2_O_2_ [M + H]^+^: 281.1290; Found [M + H]^+^: 281.1306.

#### Methyl (E)-4-(2,4-Dimethoxybenzyl iminomethyl)benzoate (**9g**)

Following General Procedure A, methyl-4-formyl benzoate (0.982 g, 6.0 mmol), 2,4-dimethoxybenzylamine (1.0 g, 6.0 mmol), magnesium sulfate (2.0 g), and dichloromethane (8 mL) were used. The title compound was obtained as a yellow solid (1.87 g, 100%). Mp −54.5–56.3 °C. ^1^H NMR (600 MHz, CDCl_3_) δ 8.35 (s, 1H), 8.06 (d, *J* = 8.0 Hz, 2H), 7.82 (d, *J* = 8.1 Hz, 2H), 7.19 (d, *J* = 9.0 Hz, 1H), 6.48 (m, 2H), 4.79 (s, 2H), 3.91 (s, 3H), 3.79 (s, 6H). ^13^C{1H} NMR (151 MHz, CDCl_3_) δ 166.7, 160.6, 160.2, 158.3, 140.3, 131.6, 130.2, 129.7, 128.0, 119.4, 104.1, 98.5, 59.1, 55.3, 55.3, 52.2. HRMS (DART) *m*/*z* calcd for C_18_H_20_NO_4_ [M + H]^+^: 314.1392; Found [M + H]^+^: 314.1422.

#### (E)-N-(2,4-Dimethoxybenzyl)-1-(pyridin-3-yl)methanimine (**9k**)

Following General Procedure A, 3-pyridinecarboxaldehyde (0.64 g, 6.0 mmol), 2,4-dimethoxybenzylamine (1.0 g, 6.0 mmol), magnesium sulfate (2.0 g), and dichloromethane (8 mL) were used. The title compound was obtained as a pale-yellow oil (1.51 g, 99%). ^1^H NMR (600 MHz, CDCl_3_) δ: 8.85 (s, 1H), 8.63 (d, *J* = 4.8 Hz, 1H), 8.34 (s, 1H), 8.14 (d, *J* = 7.9 Hz, 1H), 7.33–7.31 (dd, *J* = 7.9 Hz, 4.8 Hz, 1H), 7.19 (d, *J* = 8.9 Hz, 1H), 6.49–6.47 (m, 2H), 4.78 (s, 2H), 3.81 (s, 3H), 3.80 (s, 3H). ^13^C{1H} NMR (151 MHz, CDCl_3_) δ 160.3, 158.7, 158.3, 151.3, 150.2, 134.5, 131.9, 130.3, 123.6, 119.2, 104.1, 98.5, 59.1, 55.3. HRMS (DART) *m*/*z* calcd for C_15_H_17_N_2_O_2_ [M + H]^+^: 257.1290; Found [M + H]^+^: 257.1297.

#### (E)-1-(Benzo[d][1,3]dioxol-5-yl)-N-(2,4-dimethoxybenzyl)methanimine (**9n**)

Following General Procedure A, piperonal (0.89 g, 6.0 mmol), 2,4-dimethoxybenzylamine (1.0 g, 6.0 mmol), magnesium sulfate (2.0 g), and dichloromethane (8 mL) were used. The title compound was obtained as a pale-yellow solid (1.77 g, 99%). Mp −54.3–55.7 °C. ^1^H NMR (600 MHz, CDCl_3_) δ: 8.2 (s, 2H), 7.4 (s, 1H), 7.18 (d, *J* = 8.9 Hz, 1H), 7.12 (d, *J* = 7.9 Hz, 1H), 6.8 (d, *J* = 7.9 Hz, 1H), 6.47–6.46 (m, 2H), 5.9 (s, 2H), 4.71 (s, 2H), 3.808 (s, 3H), 3.802 (s, 3H). ^13^C{1H} NMR (151 MHz, CDCl_3_) δ 160.8, 160.0, 158.2, 149.7, 148.2, 131.3, 130.0, 124.3, 120.1, 107.9, 106.7, 104.0, 101.4, 98.5, 58.6, 55.3. HRMS (DART) *m*/*z* calcd for C_17_H_18_NO_4_ [M + H]^+^: 300.1236; Found [M + H]^+^: 300.1253.

#### (E)-1-(2,3-Dihydrobenzofuran-5-yl)-N-(2,4-dimethoxybenzyl)methanimine (**9o**)

Following General Procedure A, 2,3-dihydrobenzofuran-5-carbaldehyde (0.886 g, 6.0 mmol), 2,4-dimethoxybenzylamine (1.0 g, 6.0 mmol), magnesium sulfate (2.0 g), and dichloromethane (8 mL) were used. The title compound was obtained as a pale-yellow oil (1.94 g, 72% purity, 78% yield). ^1^H NMR (600 MHz, CDCl_3_) δ 8.24 (s, 1H), 7.73 (s, 1H), 7.44 (d, *J* = 12 Hz, 1H), 7.19 (d, *J* = 8.9 Hz, 1H), 6.78 (d, *J* = 8.2 Hz, 1H), 6.49–6.43 (m, 2H), 4.71 (s, 2H), 4.60 (t, *J* = 8.7 Hz, 2H), 3.80 (s, 6H), 3.20 (t, *J* = 8.7 Hz, 2H). ^13^C{1H} NMR (151 MHz, CDCl_3_) δ 162.3, 161.3, 160.0, 158.2, 130.0, 129.8, 129.6, 127.7, 124.2, 120.3, 109.0, 104.0, 98.4, 71.7, 58.7, 55.3, 29.2. HRMS (DART) *m*/*z* calcd for C_18_H_20_NO_3_ [M + H]^+^: 298.1443; Found [M + H]^+^: 298.1466.

#### (E)-N-(2,4-Dimethoxybenzyl)-1-(naphthalen-2-yl)methanimine (**9pa**)

Following General Procedure A, 2-naphthaldehyde (0.937 g, 6.0 mmol), 2,4-dimethoxybenzylamine (1.0 g, 6.0 mmol), magnesium sulfate (2.0 g), and dichloromethane (8 mL) were used. The title compound was obtained as a white solid (1.78 g, 98%). Mp −91.5–93.9 °C. ^1^H NMR (600 MHz, CDCl_3_) δ: 8.48 (s, 1H), 8.05–8.03 (m, 2H), 7.89–7.87 (m, 1H), 7.85–7.83 (m, 2H), 7.51–7.49 (m, 2H), 7.24 (d, *J* = 8.9 Hz, 1H), 6.49–6.48 (m, 2H), 4.82 (s, 2H), 3.82 (s, 3H), 3.81 (s, 3H). ^13^C{1H} NMR (151 MHz, CDCl_3_) δ: 161.7, 160.1, 158.3, 134.6, 134.1, 133.1, 130.1, 129.9, 128.5, 128.3, 127.8, 127.0, 126.3, 124.0, 119.9, 104.9, 104.0, 98.5, 59.0, 55.4. HRMS (DART) *m*/*z* calcd for C_20_H_20_NO_2_ [M + H]^+^: 306.1494; Found [M + H]^+^: 306.1506.

#### (E)-N-(4-Fluorobenzyl)-1-(naphthalen-2-yl)methanimine (**9pd**)

Following General Procedure A, 2-naphthaldehyde (0.937 g, 6.0 mmol), 4-fluoro benzylamine (1.05 g, 6.0 mmol), magnesium sulfate (2.0 g), and dichloromethane (8 mL) were used. The title compound was obtained as an off-white solid (1.28 g, 90% purity, 73% yield). Mp −87.7–88.9 °C. ^1^H NMR (600 MHz, CDCl_3_) δ 8.53 (s, 1H), 8.09 (s, 1H), 8.08 (d, *J* = 12.0 Hz, 1H), 7.92–7.86 (m, 3H), 7.54 (tdd, *J* = 8.0, 6.1, 3.3 Hz, 2H), 7.36 (dd, *J* = 8.4, 5.5 Hz, 2H), 7.07 (t, *J* = 8.7 Hz, 2H), 4.85 (s, 2H). ^13^C{1H} NMR (151 MHz, CDCl_3_) δ 162.8 (C–F, ^1^*J* C–F = 244.62 Hz), 162.1, 161.2 (C–F, ^1^*J* C–F = 244.62 Hz), 135.14 (C–F, ^3^*J* C–F = 3.02 Hz), 135.12 (C–F, ^3^*J* C–F = 3.02 Hz), 134.8, 133.7, 133.1, 130.2, 129.6, 129.5, 128.6, 128.5, 127.9, 127.2, 126.5, 123.9, 115.4 (C–F, ^2^*J* C–F = 21.14 Hz), 115.2 (C–F, ^2^*J* C–F = 21.14 Hz), 64.37. ^19^F NMR (565 MHz, CDCl_3_) δ – 115.92. HRMS (DART) *m*/*z* calcd for C_18_H_15_FN [M + H]^+^: 264.1189; Found [M + H]^+^: 264.1193.

#### (E)-1-(Naphthalen-2-yl)-N-(4-(trifluoromethyl)benzyl)methanimine (**9pe**)

Following General Procedure A, 2-naphthaldehyde (0.937 g, 6.0 mmol), 4-trifluoromethyl benzylamine (1.05 g, 6.0 mmol), magnesium sulfate (2.0 g), and dichloromethane (8 mL) were used. The title compound was obtained as an off-white solid (1.71 g, 91%). Mp −105.9–107.9 °C. ^1^H NMR (600 MHz, CDCl_3_) δ 8.56 (s, 1H), 8.10 (s, 1H), 8.09 (d, *J* = 6 Hz, 1H), 7.95–7.85 (m, 3H), 7.64 (d, *J* = 8.0 Hz, 2H), 7.58–7.49 (m, 4H), 4.92 (s, 2H). ^13^C{1H} NMR (151 MHz, CDCl_3_) δ 162.7, 143.6, 134.9, 133.6, 133.1, 130.4, 129.61 (C–F, ^2^*J* C–F = 31.71 Hz), 129.40 (C–F, ^2^*J* C–F = 31.71 Hz), 129.18 (C–F, ^2^*J* C–F = 31.71 Hz), 129.18 (C–F, ^2^*J* C–F = 31.71 Hz), 128.69, 128.63, 128.1, 127.9, 127.3, 127.04 (C–F, ^1^*J* C–F = 271.8 Hz), 126.6, 125.50 (C–F, ^3^*J* C–F = 3.02 Hz), 125.47 (C–F, ^3^*J* C–F = 3.02 Hz), 125.45 (C–F, ^3^*J* C–F = 3.02 Hz), 125.42 (C–F, ^3^*J* C–F = 3.02 Hz), 125.24 (C–F, ^1^*J* C–F = 271.8 Hz), 123.8, 123.43 (C–F, ^1^*J* C–F = 271.8 Hz), 121.63 (C–F, ^1^*J* C–F = 271.8 Hz), 64.4. ^19^F NMR (565 MHz, CDCl_3_) δ −62.28. HRMS (DART) *m*/*z* calcd for C_19_H_15_F_3_N [M + H]^+^: 314.1157; Found [M + H]^+^: 314.1154.

#### (E)-1-([1,1′-Biphenyl]-4-yl)-N-(2,4-dimethoxybenzyl)methanimine (**9q**)

Following General Procedure A, 4-phenylbenzaldehyde (1.09 g, 6.0 mmol), 2,4-dimethoxybenzylamine (1.0 g, 6.0 mmol), magnesium sulfate (2.0 g), and dichloromethane (8 mL) were used. The title compound was obtained as a white solid (1.82 g, 94% purity, 86% yield). Mp −91.3–93.9 °C. ^1^H NMR (600 MHz, CDCl_3_) δ 8.41 (s, 1H), 7.88 (d, *J* = 8.0 Hz, 2H), 7.70–7.63 (m, 4H), 7.49 (t, *J* = 7.6 Hz, 2H), 7.40 (t, *J* = 6 Hz, 1H), 7.29–7.23 (m, 1H), 6.55–6.50 (m, 2H), 4.83 (s, 2H), 3.86 (s, 3H), 3.84 (s, 3H). ^13^C{1H} NMR (151 MHz, CDCl_3_) δ 161.3, 160.1, 158.3, 143.2, 140.5, 135.4, 130.3, 130.1, 129.0, 128.8, 128.7, 128.5, 127.7, 127.4, 127.2, 127.1, 120.0, 104.1, 98.5, 59.0, 55.4. HRMS (DART) *m*/*z* calcd for C_22_H_22_NO_2_ [M + H]^+^: 332.1651; Found [M + H]^+^: 332.1667.

#### (E)-N-(2,4-Dimethoxybenzyl)-1-(p-tolyl)methanimine (**9r**)

Following General Procedure A, *p*-tolualdehyde (0.72 g, 6.0 mmol), 2,4-dimethoxybenzylamine (1.0 g, 6.0 mmol), magnesium sulfate (2.0 g), and dichloromethane (8 mL) were used. The title compound was obtained as a pale-yellow oil (1.28 g, 80%). ^1^H NMR (600 MHz, CDCl_3_) δ 8.36 (s, 1H), 7.72 (d, *J* = 7.8 Hz, 2H), 7.27 (d, *J* = 7.4 Hz, 3H), 6.55–6.51 (m, 2H), 4.81 (s, 2H), 3.86 (s, 6H), 2.38 s, 3H). ^13^C{1H} NMR (151 MHz, CDCl_3_) δ 161.7, 160.1, 158.2, 140.7, 133.8, 130.0, 129.8, 129.7, 129.2, 128.2, 120.1, 104.0, 98.5, 58.9, 55.3, 21.5. HRMS (DART) *m*/*z* calcd for C_17_H_20_NO_2_ [M + H]^+^: 270.1494; Found [M + H]^+^: 270.1495.

#### (E)-N-(2,4-Dimethoxybenzyl)-1-(furan-2-yl)methanimine (**9s**)

Following General Procedure A, furfural (0.57 g, 6.0 mmol), 2,4-dimethoxybenzylamine (1.0 g, 6.0 mmol), magnesium sulfate (2.0 g), and dichloromethane (8 mL) were used. The title compound was obtained as a brown oil (1.6 g, 91% purity, 99% yield). ^1^H NMR (600 MHz, CDCl_3_) δ: 8.08 (s, 1H), 7.49 (s, 1H), 7.18 (d, *J* = 8.1 Hz, 1H), 6.73 (d, *J* = 3.4 Hz, 1H), 6.47–6.45 (m, 3H), 4.73 (s, 2H), 3.80 (s, 3H), 3.79 (s, 3H). ^13^C{1H} NMR (151 MHz, CDCl_3_) δ: 160.28, 158.46, 151.96, 150.13, 144.51, 130.69, 119.30, 113.59, 111.53, 104.07, 98.47, 58.89, 55.40, 55.33. HRMS (DART) *m*/*z* calcd for C_14_H_16_NO_3_ [M + H]^+^: 246.1130; Found [M + H]^+^: 246.1126.

#### (E)-N-(2,4-Dimethoxybenzyl)-1-(thiophen-2-yl)methanimine (**9t**)

Following General Procedure A, thiophene-2-carboxaldehyde (0.67 g, 6.0 mmol), 2,4-dimethoxybenzylamine (1.0 g, 6.0 mmol), magnesium sulfate (2.0 g), and dichloromethane (8 mL) were used. The title compound was obtained as a yellow solid (1.15 g, 74%). Mp −47.4–50.2 °C. ^1^H NMR (600 MHz, CDCl_3_) δ 8.36 (s, 1H), 7.37 (d, *J* = 6 Hz, 1H), 7.29 (d, *J* = 3.6 Hz, 1H), 7.18 (d, *J* = 8.4 Hz, 1H), 7.06 (t, *J* = 6 Hz, 1H), 6.50–6.46 (m, 2H), 4.74 (s, 2H), 3.80 (s, 6H). ^13^C{1H} NMR (151 MHz, CDCl_3_) δ 160.2, 158.3, 154.8, 142.9, 130.3, 130.2, 128.6, 127.2, 119.6, 104.1, 98.4, 58.2, 55.4, 55.3. HRMS (DART) *m*/*z* calcd for C_14_H_16_NO_2_S [M + H]^+^: 262.0902; Found [M + H]^+^: 262.0915.

### General Procedure B for the Synthesis of **18**

To a 20 mL crimp cap vial with a stir bar in an Ar filled glovebox were charged Cu(OAc)_2_ (3.6 mg, 20 μmol) and PCy_3_ (7.3 mg, 26 μmol) followed by toluene (1.0 mL) and *tert*-butanol (76.5 μL, 2 equiv). The mixture was stirred for 5 min. Allenamide **15a** (96.6 mg, 480 μmol) followed by imine (400 μmol) was then charged, and the vial was sealed with a crimp-cap septum and removed from the glovebox. Dimethoxymethylsilane (0.099 mL, 2 equiv) was then charged to the reaction mixture *(**Caution:**dimethoxymethylsilane should be handled in a well-ventilated fume hood because it is known to cause blindness. Syringes were quenched with 2 M NaOH, gas evolution! prior to disposal)*. The mixture was then stirred at rt for 24 h. The reaction was quenched by addition of 200 mg of NH_4_F and 2.5 mL of MeOH followed by agitation at rt for 30 min. A 10 mL volumen of 5% NaHCO_3_ was then added to the mixture followed by extraction with DCM (2 × 5 mL). The combined organics were dried with Na_2_SO_4_, filtered, and concentrated in vacuo. Crude product was purified by flash chromatography on silica gel to afford the desired product.

#### (S)-3-((1*S*,2*S*)-1-((2,4-Dimethoxybenzyl)amino)-1-(4-(trifluoromethyl)phenyl)but-3-en-2-yl)-4-phenyloxazolidin-2-one (**18a**)

According to General Procedure B, the product was purified by silica gel chromatography (5% E.A. in DCM) to provide 180 mg (85%) of **18a** as a white foam as a single diastereomer. *R*_*f*_ = 0.43 (50% EtOAC/hexanes). ^1^H NMR (600 MHz, CDCl_3_) δ 7.50 (d, *J* = 8.0 Hz, 1H), 7.36 (dd, *J* = 5.1, 1.8 Hz, 2H), 7.30 (d, *J* = 8.0 Hz, 1H), 7.21–7.17 (m, 1H), 6.99 (d, *J* = 6 Hz, 1H), 6.49 (s, 1H), 6.47 (d, *J* = 8.3 Hz, 1H), 5.15–5.09 (dt, *J* = 18 Hz, 12 Hz, 1H), 4.73 (d, *J* = 12 Hz, 1H), 4.70 (d, *J* = 17.1 Hz, 1H), 4.61 (t, *J* = 8.2 Hz, 1H), 4.52 (t, *J* = 12 Hz, 1H), 4.17 (d, *J* = 6 Hz, 1H), 4.11 (t, *J* = 8.0 Hz, 1H), 3.93 (t, *J* = 9.6 Hz, 1H), 3.83 (s, 6H). 3.66 (d, *J* = 12 Hz, 1H), 3.36 (d, *J* = 18 Hz, 1H). ^13^C{1H} NMR (151 MHz, CDCl_3_) δ 160.3, 158.8, 158.3, 145.2, 138.2, 132.6, 130.5, 129.94 (C–F, ^2^*J* C–F = 31.71 Hz), 129.73 (C–F, ^2^*J* C–F = 31.71 Hz), 129.51 (C–F, ^2^*J* C–F = 31.71 Hz), 129.29 (C–F, ^2^*J* C–F = 31.71 Hz), 129.17, 129.11, 128.8, 127.8, 126.90 (C–F, ^1^*J* C–F = 271.8 Hz), 125.12 (C–F, ^3^*J* C–F = 4.53 Hz), 125.09 (C–F, ^2^*J* C–F = 4.53 Hz), 123.29 (C–F, ^1^*J* C–F = 271.8 Hz), 121.49 (C–F, ^1^*J* C–F = 271.8 Hz), 120.5, 119.6, 103.6, 98.6, 70.2, 63.4, 61.2, 59.3, 55.4, 55.2, 46.0. ^19^F NMR (565 MHz, CDCl_3_) δ – 62.36. HRMS (DART) *m*/*z* calcd for C_29_H_30_F_3_N_2_O_4_ [M + H]^+^: 527.2158; Found [M + H]^+^: 527.2153.

#### (S)-3-((1*S*,2*S*)-1-((2,4-dimethoxybenzyl)amino)-1-phenylbut-3-en-2-yl)-4-phenyloxazolidin-2-one (**18b**)

According to General Procedure B, the product was purified by silica gel chromatography (10% E.A. in DCM) to provide 147 mg (80%) of **18b** as a colorless foam as a single diastereomer. *R*_*f*_ = 0.35 (50% EtOAc/hexanes). ^1^H NMR (600 MHz, CDCl_3_) δ 7.32 (dd, *J* = 4.5, 2.3 Hz, 3H), 7.25–7.22 (d, *J* = 6 Hz, 2H), 7.22–7.19 (d, *J* = 6 Hz, 1H), 7.18–7.16 (d, *J* = 12 Hz, 2H), 7.15–7.14 (m, 2H), 7.03 (d, *J* = 8.0 Hz, 1H), 6.48 (s, 1H), 6.46 (d, *J* = 8.3 Hz, 1H), 5.05–4.97 (dt, *J* = 18 Hz, 12 Hz, 1H), 4.70 (d, *J* = 17.0 Hz, 1H), 4.64 (d, *J* = 10.2 Hz, 1H), 4.59 (t, *J* = 6 Hz, 1H), 4.46 (t, *J* = 8.6 Hz, 1H), 4.12–4.04 (dt, *J* = 18 Hz, 6 Hz, 2H), 3.99 (d, *J* = 10.0 Hz, 1H), 3.81 (s, 6H), 3.67 (d, *J* = 13.4 Hz, 1H), 3.34 (d, *J* = 13.4 Hz, 1H). ^13^C{1H} NMR (151 MHz, CDCl_3_) δ 160.2, 158.8, 158.6, 140.6, 138.9, 133.3, 130.6, 128.98, 128.96, 128.4, 128.2, 127.8, 127.4, 120.9, 119.0, 103.6, 98.6, 70.2, 63.2, 61.5, 58.9, 55.4, 55.2, 45.8. HRMS (DART) *m*/*z* calcd for C_28_H_31_N_2_O_4_ [M + H]^+^: 459.2284; Found [M + H]^+^: 459.2300.

#### (S)-3-((1*S*,2*S*)-1-((4-Methoxybenzyl)amino)-1-phenylbut-3-en-2-yl)-4-phenyloxazolidin-2-one (**18c**)

According to General Procedure B, the product was purified by silica gel chromatography (10% E.A. in DCM) to provide 153 mg (89%) of **18c** as a white solid as a single diastereomer. Mp 101–104 °C. *R*_*f*_ = 0.41 (50% EtOAc/hexanes). ^1^H NMR (600 MHz, CDCl_3_) δ 7.37–7.33 (m, 3H), 7.31 (m, 2H), 7.27 (m, 3H), 7.20 (d, *J* = 6 Hz, 2H), 7.18–7.14 (m, 2H), 6.96 (d, *J* = 12 Hz, 2H), 5.10 (dt, *J* = 16.5, 9.6 Hz, 1H), 4.76–4.69 (m, 2H), 4.66 (t, *J* = 8.4 Hz, 1H), 4.60 (t, *J* = 12 Hz, 1H), 4.18 (t, *J* = 12 Hz, 1H), 4.12 (t, *J* = 12 Hz, 1H), 4.02 (d, *J* = 10.2 Hz, 1H), 3.86 (s, 3H), 3.64 (d, *J* = 13.0 Hz, 1H), 3.41 (d, *J* = 12.9 Hz, 1H), 1.91 (s, 1H). ^13^C{1H} NMR (151 MHz, CDCl_3_) δ 159.1, 158.7, 140.5, 138.5, 133.2, 132.5, 129.8, 128.9, 128.9, 128.4, 128.2, 127.8, 127.5, 119.2, 113.7, 70.3, 63.2, 61.5, 60.4, 59.0, 55.3, 49.9, 21.0, 14.2. HRMS (DART) *m*/*z* calcd for C_27_H_29_N_2_O_3_ [M + H]^+^: 429.2178; Found [M + H]^+^: 429.2196.

#### (S)-3-((1*S*,2*S*)-1-(4-Chlorophenyl)-1-((2,4-dimethoxybenzyl)amino)but-3-en-2-yl)-4-phenyloxazolidin-2-one (**18d**)

According to General Procedure B, the product was purified by silica gel chromatography (5% E.A. in DCM) to provide 146 mg (74%) of **18d** as a colorless foam and a single diastereomer. *R*_*f*_ = 0.33 (50% EtOAc/hexanes). ^1^H NMR (600 MHz, CDCl_3_) δ 7.36–7.33 (m, 3H), 7.23 (d, *J* = 8.2 Hz, 2H), 7.20–7.16 (m, 2H), 7.12 (d, *J* = 8.1 Hz, 2H), 6.99 (d, *J* = 8.1 Hz, 1H), 6.49 (s, 1H), 6.47 (d, *J* = 8.2 Hz, 1H), 5.11–5.03 (dt, *J* = 18 Hz, 12 Hz, 1H), 4.72 (s, 1H), 4.69 (d, *J* = 7.9 Hz, 1H), 4.60 (t, *J* = 8.1 Hz, 1H), 4.49 (t, *J* = 8.6 Hz, 1H), 4.10 (t, *J* = 7.9 Hz, 1H), 4.05 (d, *J* = 10.1 Hz, 1H), 3.95 (t, *J* = 9.6 Hz, 1H), 3.83 (s, 6H), 3.66 (d, *J* = 13.4 Hz, 1H), 3.33 (d, *J* = 13.4 Hz, 1H), 2.08 (s, 1H). ^13^C{1H} NMR (151 MHz, CDCl_3_) δ 160.3, 158.8, 158.4, 139.3, 138.4, 133.0, 132.8, 130.6, 129.8, 129.10, 129.07, 128.4, 127.8, 120.6, 119.4, 103.6, 98.6, 70.2, 63.4, 60.8, 59.2, 55.4, 55.2, 45.9. HRMS (DART) *m*/*z* calcd for C_28_H_30_ClN_2_O_4_ [M + H]^+^: 493.1894; Found [M + H]^+^: 493.1929.

#### (S)-3-((1*S*,2*S*)-1-((2,4-Dimethoxybenzyl)amino)-1-(4-fluorophenyl)but-3-en-2-yl)-4-phenyloxazolidin-2-one (**18e**)

According to General Procedure B, the product was purified by silica gel chromatography (5% E.A. in DCM) to provide 147 mg (77%) of **18e** as a colorless foam and a single diastereomer. *R*_*f*_ = 0.35 (50% EtOAc/hexanes). ^1^H NMR (600 MHz, CDCl_3_) δ 7.37–7.32 (m, 3H), 7.20–7.12 (m, 4H), 7.00 (d, *J* = 8.1 Hz, 1H), 6.94 (t, *J* = 8.6 Hz, 2H), 6.50 (s, 1H), 6.48 (d, *J* = 6 Hz, 1H), 5.06 (dt, *J* = 18.2, 9.5 Hz, 1H), 4.70 (d, *J* = 16.6 Hz, 2H), 4.60 (t, *J* = 8.1 Hz, 1H), 4.49 (t, *J* = 8.6 Hz, 1H), 4.10 (t, *J* = 7.9 Hz, 1H), 4.05 (d, *J* = 10.1 Hz, 1H), 3.96 (t, *J* = 9.6 Hz, 1H), 3.83 (s, 6H), 3.66 (d, *J* = 13.4 Hz, 1H), 3.34 (d, *J* = 13.5 Hz, 1H), 2.10 (s, 1H). ^13^C{1H} NMR (151 MHz, CDCl_3_) δ 162.93 (C–F, ^1^*J* C–F = 244.62 Hz), 161.31 (C–F, ^1^*J* C–F = 244.62 Hz), 160.2, 158.8, 158.4, 138.5, 136.4, 133.0, 130.5, 129.9, 129.8, 129.07 (C–F, ^3^*J* C–F = 4.53 Hz), 129.05 (C–F, ^3^*J* C–F = 4.53 Hz), 127.8, 120.7, 119.2, 115.13 (C–F, ^2^*J* C–F = 21.14 Hz), 114.99 (C–F, ^2^*J* C–F = 21.14 Hz), 103.6, 98.6, 70.2, 63.5, 60.7, 59.1, 55.4, 55.2, 45.9. ^19^F NMR (565 MHz, CDCl_3_) δ −115.14. HRMS (DART) *m*/*z* calcd for C_28_H_30_FN_2_O_4_ [M + H]^+^: 477.2190; Found [M + H]^+^: 477.2204.

#### 4-((1*S*,2*S*)-1-((2,4-Dimethoxybenzyl)amino)-2-((S)-2-oxo-4-phenyloxazolidin-3-yl)but-3-en-1-yl)benzonitrile (**18f**)

According to General Procedure B, the product was purified by silica gel chromatography (10% E.A. in DCM) to provide 157 mg (81%) of **18f** as a colorless foam and a single diastereomer. *R*_*f*_ = 0.36 (50% EtOAc/hexanes). ^1^H NMR (600 MHz, CDCl_3_) δ 7.54 (d, *J* = 7.9 Hz, 2H), 7.39–7.34 (m, 3H), 7.30 (d, *J* = 7.9 Hz, 2H), 7.22–7.18 (m, 2H), 6.95 (d, *J* = 8.1 Hz, 1H), 6.49 (s, 1H), 6.46 (d, *J* = 8.1 Hz, 1H), 5.14 (dt, *J* = 16.9, 9.8 Hz, 1H), 4.73 (d, *J* = 10.1 Hz, 1H), 4.66 (d, *J* = 17.0 Hz, 1H), 4.59 (t, *J* = 8.2 Hz, 1H), 4.51 (t, *J* = 8.6 Hz, 1H), 4.20 (d, *J* = 10.0 Hz, 1H), 4.12 (t, *J* = 8.1 Hz, 1H), 3.82 (s, 7H), 3.63 (d, *J* = 13.5 Hz, 1H), 3.32 (d, *J* = 13.5 Hz, 1H), 2.20 (s, 1H). ^13^C{1H} NMR (151 MHz, CDCl_3_) δ 160.4, 158.7, 158.2, 146.8, 137.9, 132.0, 130.5, 129.3, 129.2, 129.1, 127.8, 120.3, 119.9, 118.8, 111.2, 103.7, 98.6, 70.2, 63.4, 61.3, 59.4, 55.4, 55.2, 46.2. HRMS (DART) *m*/*z* calcd for C_29_H_30_N_3_O_4_ [M + H]^+^: 484.2236; Found [M + H]^+^: 484.2255.

#### Methyl 4-((1*S*,2*S*)-1-((2,4-Dimethoxybenzyl)amino)-2-((S)-2-oxo-4-phenyloxazolidin-3-yl)but-3-en-1-yl)benzoate (**18g**)

According to General Procedure B, the product was purified by silica gel chromatography (10% E.A. in DCM) to provide 137 mg (66%) of **18g** as a colorless foam and a single diastereomer. *R*_*f*_ = 0.34 (50% EtOAc/hexanes). ^1^H NMR (600 MHz, CDCl_3_) δ 7.93 (d, *J* = 8.0 Hz, 2H), 7.35 (dd, *J* = 4.6, 1.6 Hz, 3H), 7.29–7.24 (m, 3H), 7.21–7.15 (m, 2H), 6.99 (d, *J* = 8.1 Hz, 1H), 6.49 (s, 1H), 6.47 (d, *J* = 8.2 Hz, 1H), 5.07 (dt, *J* = 14.2, 9.5 Hz, 1H), 4.67 (d, *J* = 16.5 Hz, 2H), 4.61 (t, *J* = 8.1 Hz, 1H), 4.49 (t, *J* = 8.6 Hz, 1H), 4.11 (m, 2H), 4.01 (t, *J* = 9.6 Hz, 1H), 3.89 (s, 3H), 3.82 (s, 6H), 3.66 (d, *J* = 13.4 Hz, 1H), 3.33 (d, *J* = 13.4 Hz, 1H), 2.18 (s, 1H). ^13^C{1H} NMR (151 MHz, CDCl_3_) δ 166.9, 160.3, 158.8, 158.4, 146.4, 138.4, 132.7, 130.6, 129.5, 129.4, 129.1, 129.0, 128.5, 127.8, 120.6, 119.5, 103.6, 98.6, 70.2, 63.3, 61.3, 59.1, 55.4, 55.2, 52.0, 46.0. HRMS (DART) *m*/*z* calcd for C_30_H_33_N_2_O_6_ [M + H]^+^: 517.2339; Found [M + H]^+^: 517.2378.

#### (S)-3-((1*S*,2*S*)-1-((2,4-Dimethoxybenzyl)amino)-1-(4-nitrophenyl)but-3-en-2-yl)-4-phenyloxazolidin-2-one (**18h**)

According to General Procedure B, the product was purified by silica gel chromatography (5% E.A. in DCM) to provide 106 mg (53%) of **18h** as a pale-yellow foam and a single diastereomer. *R*_*f*_ = 0.39 (50% EtOAc/hexanes). ^1^H NMR (600 MHz, CDCl_3_) δ 8.13–8.07 (m, 2H), 7.40–7.32 (m, 5H), 7.21 (dd, *J* = 6.3, 2.4 Hz, 2H), 6.95 (d, *J* = 8.1 Hz, 1H), 6.49 (s, 1H), 6.46 (d, *J* = 8.1 Hz, 1H), 5.17 (dt, *J* = 16.9, 9.8 Hz, 1H), 4.74 (d, *J* = 10.2 Hz, 1H), 4.66 (d, *J* = 17.0 Hz, 1H), 4.61 (t, *J* = 8.3 Hz, 1H), 4.52 (t, *J* = 8.6 Hz, 1H), 4.27 (d, *J* = 10.0 Hz, 1H), 4.16–4.10 (m, 1H), 3.82 (s, 6H), 3.64 (d, *J* = 13.5 Hz, 1H), 3.33 (d, *J* = 13.5 Hz, 1H), 2.28 (s, 1H). ^13^C{1H} NMR (151 MHz, CDCl_3_) δ 160.4, 158.7, 158.2, 147.4, 137.9, 132.2, 130.5, 129.4, 129.3, 129.2, 127.8, 123.4, 120.2, 120.0, 103.7, 98.7, 70.2, 63.5, 61.1, 59.5, 55.4, 55.2, 46.2. HRMS (DART) *m*/*z* calcd for C_28_H_30_N_3_O_6_ [M + H]^+^: 504.2135; Found [M + H]^+^: 504.2119.

#### (S)-3-((1*S*,2*S*)-1-(Benzylamino)-1-(3-nitrophenyl)but-3-en-2-yl)-4-phenyloxazolidin-2-one (**18i**)

The reaction was set up according to General Procedure B and stirred at 65 °C for 24 h. The product was purified by silica gel chromatography (5% E.A. in DCM) to provide 88 mg (50%) of **18i** as a pale-yellow foam and a single diastereomer and as a 91:9 mixture of the branched **18i** to the rearranged product **19i**. *R*_*f*_ = 0.71 (20% EtOAc/DCM). ^1^H NMR (600 MHz, CDCl_3_) δ 7.98 (d, *J* = 6.0 Hz, 1H), 7.91 (s, 1H), 7.47 (d, *J* = 7.7 Hz, 1H), 7.35 (t, *J* = 7.9 Hz, 1H), 7.28–7.16 (m, 9H), 7.05 (d, *J* = 6.7 Hz, 2H), 5.16 (dt, *J* = 17.0, 9.7 Hz, 1H), 4.69 (d, *J* = 10.1 Hz, 1H), 4.59 (d, *J* = 17.0 Hz, 1H), 4.53 (t, *J* = 8.4 Hz, 1H), 4.47 (t, *J* = 8.6 Hz, 1H), 4.22 (d, *J* = 10.0 Hz, 1H), 4.02 (t, *J* = 8.2 Hz, 1H), 3.77 (t, *J* = 9.8 Hz, 1H), 3.56 (d, *J* = 13.3 Hz, 1H), 3.36 (d, *J* = 13.2 Hz, 1H). ^13^C{1H} NMR (151 MHz, CDCl_3_) δ 158.7, 148.3, 137.5, 134.3, 132.1, 129.4, 129.3, 129.2, 128.49, 128.47, 127.7, 127.3, 123.3, 122.8, 120.3, 70.2, 63.5, 61.5, 59.6, 50.9. HRMS (DART) *m*/*z* calcd for C_26_H_26_N_3_O_4_ [M + H]^+^: 444.1923; Found [M + H]^+^: 444.1952.

#### (S)-3-((1*S*,2*S*)-1-(Benzylamino)-1-(3-bromophenyl)but-3-en-2-yl)-4-phenyloxazolidin-2-one (**18j**)

The reaction was set up according to General Procedure B and stirred at 65 °C for 24 h. The product was purified by silica gel chromatography (2.5% E.A. in DCM) to provide 173 mg (82%) of **18j** as a pale-yellow foam and a single diastereomer. *R*_*f*_ = 0.53 (50% EtOAc/hexanes). ^1^H NMR (600 MHz, CDCl_3_) δ 7.41–7.29 (m, 10H), 7.16 (t, *J* = 7.7 Hz, 1H), 7.14–7.09 (m, 3H), 5.14 (dt, *J* = 16.8, 9.5 Hz, 1H), 4.75 (dd, *J* = 18 Hz, 12 Hz, 2H), 4.60 (t, *J* = 8.3 Hz, 1H), 4.55 (t, *J* = 8.5 Hz, 1H), 4.09 (t, *J* = 8.0 Hz, 1H), 4.04 (d, *J* = 10.2 Hz, 1H), 3.99 (t, *J* = 9.5 Hz, 1H), 3.68 (d, *J* = 13.2 Hz, 1H), 3.45 (d, *J* = 13.2 Hz, 1H), 1.96 (s, 1H). ^13^C{1H} NMR (151 MHz, CDCl_3_) δ 159.0, 143.1, 140.1, 138.1, 132.6, 131.2, 130.7, 130.0, 129.1, 129.0, 128.5, 128.4, 127.8, 127.2, 126.8, 122.6, 119.7, 70.2, 63.3, 61.4, 59.3, 50.7. HRMS (DART) *m*/*z* calcd for C_26_H_26_BrN_2_O_2_ [M + H]^+^: 477.1178; Found [M + H]^+^: 477.1182.

#### (S)-3-((1*S*,2*S*)-1-((2,4-Dimethoxybenzyl)amino)-1-(pyridin-3-yl)but-3-en-2-yl)-4-phenyloxazolidin-2-one (**18k**)

According to General Procedure B, the product was purified by silica gel chromatography (40% E.A. in DCM) to provide 143 mg (78%) of **18k** as a pale-yellow foam as a single diastereomer. *R*_*f*_ = 0.1 (60% EtOAc/hexanes). ^1^H NMR (600 MHz, CDCl_3_) δ 8.40 (d, *J* = 73.8 Hz, 2H), 7.57 (d, *J* = 7.8 Hz, 1H), 7.38–7.31 (m, 3H), 7.23–7.14 (m, 3H), 6.99 (d, *J* = 8.1 Hz, 1H), 6.49 (s, 1H), 6.47 (d, *J* = 8.1 Hz, 1H), 5.09 (dt, *J* = 17.0, 9.8 Hz, 1H), 4.72 (d, *J* = 10.2 Hz, 1H), 4.68 (d, *J* = 17.1 Hz, 1H), 4.57 (t, *J* = 8.1 Hz, 1H), 4.50 (t, *J* = 8.6 Hz, 1H), 4.16–4.08 (m, 2H), 3.93 (t, *J* = 9.7 Hz, 1H), 3.83 (s, 3H), 3.82 (s, 3H), 3.67 (d, *J* = 13.5 Hz, 1H), 3.35 (d, *J* = 13.5 Hz, 1H), 2.24 (s, 1H). ^13^C{1H} NMR (151 MHz, CDCl_3_) δ 160.4, 158.8, 158.3, 150.5, 149.0, 138.1, 136.3, 135.6, 132.5, 130.6, 129.2, 129.1, 127.8, 123.5, 120.3, 120.0, 103.7, 98.7, 70.2, 63.4, 59.2, 59.0, 55.4, 55.2, 46.0. HRMS (DART) *m*/*z* calcd for C_27_H_30_N_3_O_4_ [M + H]^+^: 460.2236; Found [M + H]^+^: 460.2264.

#### (S)-3-((1*S*,2*S*)-1-((2,4-Dimethoxybenzyl)amino)-1-(4-methoxyphenyl)but-3-en-2-yl)-4-phenyloxazolidin-2-one (**18l**)

According to General Procedure B, the product was purified by silica gel chromatography (10% E.A. in DCM) to provide 143 mg (73%) of **18l** as a colorless foam and a single diastereomer. *R*_*f*_ = 0.25 (50% EtOAc/hexanes). ^1^H NMR (600 MHz, CDCl_3_) δ 7.33 (dd, *J* = 4.4, 2.3 Hz, 3H), 7.16 (dd, *J* = 5.9, 2.7 Hz, 2H), 7.10 (d, *J* = 8.2 Hz, 2H), 7.04 (d, *J* = 8.1 Hz, 1H), 6.83–6.78 (m, 2H), 6.50 (s, 1H), 6.49–6.45 (d, *J* = 12 Hz, 1H), 5.06–4.98 (dt, *J* = 18 Hz, 12 Hz, 1H), 4.73 (d, *J* = 17.0 Hz, 1H), 4.67 (d, *J* = 10.1 Hz, 1H), 4.59 (t, *J* = 7.9 Hz, 1H), 4.47 (t, *J* = 12 Hz, 1H), 4.11–4.04 (m, 2H), 3.97 (d, *J* = 10.0 Hz, 1H), 3.83 (s, 6H), 3.78 (s, 3H), 3.68 (d, *J* = 13.5 Hz, 1H), 3.35 (d, *J* = 13.4 Hz, 1H). ^13^C{1H} NMR (151 MHz, CDCl_3_) δ 160.21, 158.9, 158.8, 158.5, 138.9, 133.4, 132.5, 130.6, 129.4, 128.96, 128.94, 127.8, 121.0, 118.9, 113.5, 103.6, 98.6, 70.2, 63.3, 60.8, 58.9, 55.4, 55.2, 55.1, 45.7. HRMS (DART) *m*/*z* calcd for C_29_H_33_N_2_O_5_ [M + H]^+^: 489.2389; Found [M + H]^+^: 489.2386.

#### (S)-3-((1*S*,2*S*)-1-(Benzylamino)-1-(2-methoxyphenyl)but-3-en-2-yl)-4-phenyloxazolidin-2-one (**18m**)

The reaction was set up according to General Procedure B and stirred at 65 °C for 24 h. The product was purified by silica gel chromatography (10% E.A. in DCM) to provide 127 mg (74%) of **18m** as a colorless foam as a single diastereomer and as a 95:5 mixture of the branched **18m** to rearranged product **19m**. *R*_*f*_ = 0.36 (50% EtOAc/hexanes). ^1^H NMR (600 MHz, CDCl_3_) δ 7.27–7.20 (m, 4H), 7.20–7.11 (m, 5H), 7.10 (t, *J* = 6 Hz, 1H), 6.92 (d, *J* = 6 Hz, 3H), 6.76 (t, *J* = 7.4 Hz, 1H), 6.68 (d, *J* = 8.1 Hz, 1H), 4.76 (dt, *J* = 18.1, 9.4 Hz, 1H), 4.61 (d, *J* = 16.8 Hz, 1H), 4.48 (t, *J* = 8.3 Hz, 2H), 4.42 (d, *J* = 10.1 Hz, 1H), 4.38 (t, *J* = 8.7 Hz, 1H), 4.08–3.95 (m, 1H), 3.92 (t, *J* = 7.9 Hz, 1H), 3.60 (d, *J* = 8.2 Hz, 1H), 3.59 (s, 3H), 3.29 (d, *J* = 13.0 Hz, 1H), 2.11 (s, 1H). ^13^C{1H} NMR (151 MHz, CDCl_3_) δ 159.4, 157.9, 140.8, 139.3, 134.3, 129.0, 128.68, 128.65, 128.49, 128.45, 128.2, 128.0, 127.0, 120.4, 118.0, 110.6, 70.2, 58.3, 55.1, 50.7. HRMS (DART) *m*/*z* calcd for C_27_H_29_N_2_O_3_ [M + H]^+^: 429.2178; Found [M + H]^+^: 429.2194.

#### (S)-3-((1*S*,2*S*)-1-(Benzo[d][1,3]dioxol-5-yl)-1-((2,4-dimethoxybenzyl)amino)but-3-en-2-yl)-4-phenyloxazolidin-2-one (**18n**)

According to General Procedure B, the product was purified by silica gel chromatography (15% E.A. in DCM) to provide 163 mg (81%) of **18n** as a colorless foam and a single diastereomer. *R*_*f*_ = 0.26 (50% EtOAc/hexanes). ^1^H NMR (600 MHz, CDCl_3_) δ 7.37–7.31 (m, 3H), 7.19–7.11 (m, 2H), 7.04 (d, *J* = 8.1 Hz, 1H), 6.72 (s, 1H), 6.69 (d, *J* = 7.9 Hz, 1H), 6.62 (d, *J* = 7.9 Hz, 1H), 6.49 (s, 1H), 6.48 (d, *J* = 7.3 Hz, 1H), 5.92 (s, 2H), 5.05 (dt, *J* = 17.3, 8.6 Hz, 1H), 4.75 (d, *J* = 17.0 Hz, 1H), 4.71 (d, *J* = 10.2 Hz, 1H), 4.58 (t, *J* = 8.0 Hz, 1H), 4.47 (t, *J* = 8.6 Hz, 1H), 4.08 (t, *J* = 7.8 Hz, 1H), 3.97 (m, 2H), 3.84 (s, 3H), 3.83 (s, 3H), 3.70 (d, *J* = 12.0 Hz, 1H), 3.36 (d, *J* = 13.4 Hz, 1H), 2.02 (s, 1H). ^13^C{1H} NMR (151 MHz, CDCl_3_) δ 160.2, 158.8, 158.4, 147.7, 146.8, 138.6, 134.6, 133.2, 130.6, 129.0, 127.8, 122.0, 120.9, 119.0, 108.1, 107.7, 103.6, 100.9, 98.6, 70.2, 63.4, 61.1, 59.0, 55.4, 55.2, 45.8. HRMS (DART) *m*/*z* calcd for C_29_H_31_N_2_O_6_ [M + H]^+^: 503.2182; Found [M + H]^+^: 503.2200.

#### (S)-3-((1*S*,2*S*)-1-(2,3-Dihydrobenzofuran-5-yl)-1-((2,4-dimethoxybenzyl)amino)but-3-en-2-yl)-4-phenyloxazolidin-2-one (**18o**)

According to General Procedure B, the product was purified by silica gel chromatography (15% E.A. in DCM) to provide 151 mg (75%) of **18o** as a colorless foam and a single diastereomer. *R*_*f*_ = 0.18 (50% EtOAc/hexanes). ^1^H NMR (600 MHz, CDCl_3_) δ 7.36–7.30 (m, 3H), 7.19–7.13 (m, 2H), 7.07–7.03 (m, 2H), 6.86 (d, *J* = 8.1 Hz, 1H), 6.65 (d, *J* = 8.1 Hz, 1H), 6.52–6.45 (m, 2H), 5.00 (dt, *J* = 16.9, 9.6 Hz, 1H), 4.75 (d, *J* = 17.0 Hz, 1H), 4.67 (d, *J* = 10.2 Hz, 1H), 4.58 (t, *J* = 7.9 Hz, 1H), 4.53 (t, *J* = 8.7 Hz, 2H), 4.47 (t, *J* = 8.6 Hz, 1H), 4.08 (t, *J* = 8.0 Hz, 2H), 3.92 (d, *J* = 10.1 Hz, 1H), 3.83 (s, 3H), 3.82 (s, 3H) 3.69 (d, *J* = 13.4 Hz, 1H), 3.36 (d, *J* = 13.4 Hz, 1H), 3.15 (t, *J* = 8.7 Hz, 2H), 2.06 (s, 1H). ^13^C{1H} NMR (151 MHz, CDCl_3_) δ 160.2, 159.4, 158.8, 138.9, 133.5, 132.5, 130.6, 128.96, 128.93, 128.4, 127.8, 127.1, 124.4, 121.0, 118.9, 108.6, 103.6, 98.6, 71.2, 70.2, 63.3, 61.0, 58.8, 55.4, 55.2, 45.7, 29.7. HRMS (DART) *m*/*z* calcd for C_30_H_33_N_2_O_5_ [M + H]^+^: 501.2389; Found [M + H]^+^: 501.2426.

#### (S)-3-((1*S*,2*S*)-1-((2,4-Dimethoxybenzyl)amino)-1-(naphthalen-2-yl)but-3-en-2-yl)-4-phenyloxazolidin-2-one (**18p**)

According to General Procedure B, the product was purified by silica gel chromatography (5% E.A. in DCM) to provide 151 mg (74%) of **18p** as a colorless foam as a single diastereomer. *R*_*f*_ = 0.30 (50% EtOAc/hexanes). ^1^H NMR (600 MHz, CDCl_3_) δ 7.83–7.75 (m, 3H), 7.63 (s, 1H), 7.45 (tdd, *J* = 7.7, 5.9, 3.2 Hz, 2H), 7.36 (dt, *J* = 6.4, 2.7 Hz, 4H), 7.23–7.19 (m, 2H), 7.06 (d, *J* = 7.9 Hz, 1H), 6.49 (m, 2H), 5.10 (dt, *J* = 17.4, 8.8 Hz, 1H), 4.71 (d, *J* = 17.0 Hz, 1H), 4.66 (t, *J* = 8.0 Hz, 1H), 4.62 (d, *J* = 10.2 Hz, 1H), 4.51 (t, *J* = 8.6 Hz, 1H), 4.22 (d, *J* = 7.4 Hz, 2H), 4.11 (t, *J* = 7.6 Hz, 1H), 3.83 (s, 6H), 3.70 (d, *J* = 13.4 Hz, 1H), 3.40 (d, *J* = 13.4 Hz, 1H), 2.23 (s, 1H). ^13^C{1H} NMR (151 MHz, CDCl_3_) δ 160.2, 158.8, 158.6, 138.8, 138.1, 133.22, 133.21, 133.1, 130.6, 129.0, 128.1, 127.88, 127.80, 127.7, 125.9, 125.8, 125.7, 120.9, 119.2, 103.6, 98.6, 70.3, 63.2, 61.7, 59.1, 55.5, 55.3, 46.0. HRMS (DART) *m*/*z* calcd for C_32_H_33_N_2_O_4_ [M + H]^+^: 509.2440; Found [M + H]^+^: 509.2411.

#### (S)-3-((1*S*,2*S*)-1-([1,1′-Biphenyl]-4-yl)-1-((2,4-dimethoxybenzyl)amino)but-3-en-2-yl)-4-phenyloxazolidin-2-one (**18q**)

According to General Procedure B, the product was purified by silica gel chromatography (5% E.A. in DCM) to provide 144 mg (67%) of **18q** as a colorless foam and a single diastereomer. *R*_*f*_ = 0.26 (50% EtOAc/hexanes). ^1^H NMR (600 MHz, CDCl_3_) δ 7.59 (d, *J* = 7.9 Hz, 2H), 7.51 (d, *J* = 7.9 Hz, 2H), 7.42 (t, *J* = 7.7 Hz, 2H), 7.37–7.30 (m, 4H), 7.26 (d, *J* = 6.0 Hz, 3H), 7.21–7.16 (m, 2H), 7.08 (d, *J* = 8.0 Hz, 1H), 6.52–6.47 (m, 2H), 5.08 (dt, *J* = 18.1, 9.6 Hz, 1H), 4.76 (d, *J* = 17.0 Hz, 1H), 4.70 (d, *J* = 10.2 Hz, 1H), 4.63 (t, *J* = 8.0 Hz, 1H), 4.50 (t, *J* = 8.6 Hz, 1H), 4.17–4.05 (m, 3H), 3.84 (s, 3H), 3.83 (s, 3H), 3.73 (d, *J* = 13.5 Hz, 1H), 3.42 (d, *J* = 13.4 Hz, 1H), 2.07 (s, 1H). ^13^C{1H} NMR (151 MHz, CDCl_3_) δ 160.2, 158.8, 158.6, 140.7, 140.1, 139.8, 138.8, 133.2, 130.6, 129.0, 128.9, 128.8, 128.7, 127.8, 127.2, 126.9, 126.8, 121.0, 119.2, 103.6, 98.6, 70.3, 63.2, 61.3, 59.0, 55.4, 55.2, 45.9. HRMS (DART) *m*/*z* calcd for C_34_H_35_N_2_O_4_ [M + H]^+^: 535.2597; Found [M + H]^+^: 535.2631.

#### (S)-3-((1*S*,2*S*)-1-((2,4-Dimethoxybenzyl)amino)-1-(p-tolyl)but-3-en-2-yl)-4-phenyloxazolidin-2-one (**18r**)

According to General Procedure B, the product was purified by silica gel chromatography (5% E.A. in DCM) to provide 143 mg (76%) of **18r** as a colorless foam and a single diastereomer. *R*_*f*_ = 0.29 (50% EtOAc/hexanes). ^1^H NMR (600 MHz, CDCl_3_) δ 7.33 (dd, *J* = 4.6, 2.3 Hz, 3H), 7.18–7.14 (m, 2H), 7.08 (s, 4H), 7.05 (d, *J* = 8.1 Hz, 1H), 6.51–6.46 (m, 2H), 5.00 (dt, *J* = 18.1, 9.6 Hz, 1H), 4.74 (d, *J* = 17.0 Hz, 1H), 4.66 (d, *J* = 10.2 Hz, 1H), 4.61 (t, *J* = 7.9 Hz, 1H), 4.47 (t, *J* = 8.6 Hz, 1H), 4.13 (t, *J* = 9.6 Hz, 1H), 4.08 (t, *J* = 6 Hz, 1H), 3.95 (d, *J* = 9.9 Hz, 1H), 3.83 (s, 6H), 3.68 (d, *J* = 13.4 Hz, 1H), 3.35 (d, *J* = 13.3 Hz, 1H), 2.31 (s, 3H), 2.02 (s, 1H). ^13^C{1H} NMR (151 MHz, CDCl_3_) δ 160.2, 158.8, 158.6, 139.0, 137.5, 137.0, 133.50, 130.6, 128.96, 128.94, 128.91, 128.3, 127.8, 121.0, 118.9, 103.6, 98.6, 70.3, 63.1, 61.2, 58.8, 55.4, 55.2, 45.7, 21.1. HRMS (DART) *m*/*z* calcd for C_29_H_33_N_2_O_4_ [M + H]^+^: 473.2440; Found [M + H]^+^: 473.2459.

#### (S)-3-((1*S*,2*S*)-1-((2,4-Dimethoxybenzyl)amino)-1-(furan-2-yl)but-3-en-2-yl)-4-phenyloxazolidin-2-one (**18s**)

According to General Procedure B, the product was purified by silica gel chromatography (5% E.A. in DCM) to provide 154 mg (86%) of **18s** as a colorless foam and as a single diastereomer. *R*_*f*_ = 0.33 (50% EtOAc/hexanes). ^1^H NMR (600 MHz, CDCl_3_) δ 7.32 (s, 4H), 7.20–7.14 (m, 2H), 7.11 (d, *J* = 7.9 Hz, 1H), 6.48 (dd, *J* = 7.9, 1.4 Hz, 2H), 6.26 (d, *J* = 1.7 Hz, 1H), 6.16 (d, *J* = 3.2 Hz, 1H), 5.18–5.07 (dt, *J* = 18 Hz, 12 Hz, 1H), 4.89 (d, *J* = 17.0 Hz, 1H), 4.75 (d, *J* = 10.2 Hz, 1H), 4.60 (t, *J* = 8.1 Hz, 1H), 4.49 (t, *J* = 12 Hz, 1H), 4.32 (t, *J* = 9.6 Hz, 1H), 4.12 (d, *J* = 10.2 Hz, 1H), 4.07 (t, _J_ = 6 Hz, 1H), 3.82 (s, 6H), 3.73 (d, *J* = 13.2 Hz, 1H), 3.47 (d, *J* = 13.1 Hz, 1H), 1.91 (s, 1H). ^13^C{1H} NMR (151 MHz, CDCl_3_) δ 160.2, 158.8, 158.7, 153.1, 141.9, 138.9, 133.0, 130.8, 128.9, 128.9, 127.7, 120.6, 118.8, 109.8, 108.7, 103.7, 98.6, 70.3, 60.8, 60.4. 58.8, 55.4, 55.2, 45.8. HRMS (DART) *m*/*z* calcd for C_26_H_29_N_2_O_5_ [M + H]^+^: 449.2076; Found [M + H]^+^: 449.2068.

#### (S)-3-((1*S*,2*S*)-1-((2,4-Dimethoxybenzyl)amino)-1-(thiophen-2-yl)but-3-en-2-yl)-4-phenyloxazolidin-2-one (**18t**)

According to General Procedure B, the product was purified by silica gel chromatography (5% E.A. in DCM) to provide 137 mg (74%) of **18t** as a pale-yellow foam and as a single diastereomer. *R*_*f*_ = 0.33 (50% EtOAc/hexanes). ^1^H NMR (600 MHz, CDCl_3_) δ 7.36–7.31 (m, 3H), 7.21 (d, *J* = 5.1 Hz, 1H), 7.18–7.13 (m, 2H), 7.07 (d, *J* = 8.1 Hz, 1H), 6.90 (ddd, *J* = 4.8, 3.5, 1.1 Hz, 1H), 6.85 (d, *J* = 3.4 Hz, 1H), 6.50 (s, 1H), 6.48 (d, *J* = 7.9 Hz, 1H), 5.15 (dt, *J* = 16.2, 9.6 Hz, 1H), 4.84 (d, *J* = 17.0 Hz, 1H), 4.77 (d, *J* = 10.2 Hz, 1H), 4.56 (dd, *J* = 8.6, 7.2 Hz, 1H), 4.47 (td, *J* = 8.6, 1.1 Hz, 1H), 4.39 (d, *J* = 9.6 Hz, 1H), 4.08 (ddd, *J* = 11.6, 6.7, 2.8 Hz, 2H), 3.82 (s, 3H), 3.83 (s, 3H), 3.80 (d, *J* = 13.4 Hz, 1H), 3.48 (d, *J* = 13.4 Hz, 1H), 2.11 (s, 1H). ^13^C{1H} NMR (151 MHz, CDCl_3_) δ 160.3, 158.8, 158.3, 145.5, 138.6, 132.8, 130.8, 129.0, 127.7, 126.2, 126.0, 124.8, 120.6, 119.3, 103.6, 98.6, 70.3, 63.5, 60.4, 59.0, 57.2, 55.4, 55.2, 46.0. HRMS (DART) *m*/*z* calcd for C_26_H_29_N_2_O_4_S [M + H]^+^: 465.1848; Found [M + H]^+^: 465.1852.

#### (S)-3-((1*S*,2*S*)-1-(5-Bromothiophen-2-yl)-1-((2,4-dimethoxybenzyl)amino)but-3-en-2-yl)-4-phenyloxazolidin-2-one (**18u**)

According to General Procedure B, the product was purified by silica gel chromatography (3% E.A. in DCM) to provide 172 mg (79%) of **18u** as a pale-yellow foam as a single diastereomer and a 95:5 mixture of the branched to rearranged product. *R*_*f*_ = 0.38 (50% EtOAc/hexanes). ^1^H NMR (600 MHz, CDCl_3_) δ 7.35 (dd, *J* = 4.2, 2.5 Hz, 3H), 7.17 (dd, *J* = 6.0, 2.6 Hz, 2H), 7.04 (d, *J* = 8.1 Hz, 1H), 6.84 (d, *J* = 6 Hz, 1H), 6.59 (d, *J* = 6 Hz, 1H), 6.50 (s, 1H), 6.48 (d, *J* = 12 Hz, 1H), 5.24 (dt, *J* = 16.7, 9.5 Hz, 1H), 4.89–4.83 (m, 2H), 4.54 (t, *J* = 8.0 Hz, 1H), 4.50 (t, *J* = 12 Hz, 1H), 4.39 (d, *J* = 9.6 Hz, 1H), 4.11 (t, *J* = 12 Hz, 1H), 3.84 (s, 3H), 3.83 (s, 3H), 3.80 (d, *J* = 13.5 Hz, 1H), 3.51 (d, *J* = 13.4 Hz, 1H), 2.24 (s, 1H). ^13^C{1H} NMR (151 MHz, CDCl_3_) δ 160.3, 158.8, 158.0, 147.7, 138.0, 132.2, 130.7, 129.2, 129.1, 129.1, 127.7, 126.4, 120.3, 119.8, 111.6, 103.6, 98.6, 70.2, 63.6, 59.4, 57.7, 55.4, 55.2, 46.1. HRMS (DART) *m*/*z* calcd for C_26_H_28_BrN_2_O_4_S [M + H]^+^: 543.0953; Found [M + H]^+^: 543.0949.

### General Procedure C for the Synthesis of **19**

To a 20 mL crimp cap vial with a stir bar in an Ar filled glovebox were charged Cu(OAc)_2_ (3.6 mg, 20 μmol) and PCy_3_ (7.3 mg, 26 μmol) followed by toluene (1.0 mL), and the mixture was stirred for 5 min. Allenamide **15a** (96.6 mg, 480 μmol) followed by imine (400 μmol) was then charged, and the vial was sealed with a crimp-cap septum and removed from the glovebox. Dimethoxymethylsilane (0.099 mL, 2 equiv) was then charged to the reaction mixture *(**Caution:**dimethoxymethylsilane should be handled in a well-ventilated fume hood because it is known to cause blindness. Syringes were quenched with 2 M NaOH, gas evolution! prior to disposal)*. The mixture was then stirred at rt for 24 h. The reaction was quenched by addition of 200 mg of NH_4_F and 2.5 mL of MeOH followed by agitation at rt for 30 min. A 10 mL volume of 5% NaHCO_3_ was then added to the mixture followed by extraction with DCM (2 × 5 mL). The combined organics were dried with Na_2_SO_4_, filtered, and concentrated in vacuo. Crude product was purified by flash chromatography on silica gel to afford the desired product **19**.

#### (4*S*,5*S*)-1-(2,4-Dimethoxybenzyl)-3-((S)-2-hydroxy-1-phenylethyl)-5-(4-(trifluoromethyl)phenyl)-4-vinylimidazolidin-2-one (**19a**)

According to General Procedure C, the product was purified by silica gel chromatography (5% E.A. in DCM) to provide 198 mg (94%) of **19a** as a colorless foam as a single diastereomer. *R*_*f*_ = 0.36 (50% EtOAc/hexanes). ^1^H NMR (600 MHz, CDCl_3_) δ 7.53 (d, *J* = 8.0 Hz, 2H), 7.35–7.30 (m, 2H), 7.26 (m, 4H), 7.18 (d, *J* = 8.0 Hz, 2H), 7.03 (d, *J* = 8.2 Hz, 1H), 6.41 (d, *J* = 6.0 Hz, 1H), 6.35 (d, *J* = 2.4 Hz, 1H), 5.59 (ddd, *J* = 17.0, 9.3, 8.7, 1.0 Hz, 1H), 5.20 (d, *J* = 10.1 Hz, 1H), 4.90 (t, *J* = 7.0 Hz, 1H), 4.85 (d, *J* = 17.1 Hz, 1H), 4.78 (d, *J* = 14.7 Hz, 1H), 4.32 (m, 1H), 4.25 (dd, *J* = 7.9, 3.4 Hz, 1H), 4.06–4.03 (m, 1H), 4.01 (d, *J* = 7.9 Hz, 1H), 3.86 (d, *J* = 14.7 Hz, 1H), 3.79 (s, 3H), 3.59 (s, 3H), 3.39 (t, *J* = 8.3 Hz, 1H). ^13^C{1H} NMR (151 MHz, CDCl_3_) δ 161.1, 160.7, 158.6, 143.0, 137.6, 134.8, 131.7, 130.55 (C–F, ^2^*J* C–F = 31.71 Hz), 130.39 (C–F, ^2^*J* C–F = 31.71 Hz), 130.18 (C–F, ^2^*J* C–F = 31.71 Hz), 129.96 (C–F, ^2^*J* C–F = 31.71 Hz), 128.7, 127.8, 127.6, 127.4, 126.7 (C–F, ^1^*J* C–F = 271.8 Hz), 125.51 (C–F, ^3^*J* C–F = 3.02 Hz), 125.48 (C–F, ^3^*J* C–F = 3.02 Hz), 124.89 (C–F, ^1^*J* C–F = 271.8 Hz), 123.09 (C–F, ^1^*J* C–F = 271.8 Hz), 121.4, 121.29 (C–F, ^2^*J* C–F = 271.8 Hz), 116.2, 104.2, 98.1, 66.2, 64.9, 63.3, 61.9, 55.3, 54.9, 40.8. ^19^F NMR (565 MHz, CDCl_3_) δ −62.54. HRMS (DART) *m*/*z* calcd for C_29_H_30_F_3_N_2_O_4_ [M + H]^+^: 527.2158; Found [M + H]^+^: 527.2173.

#### (4*S*,5*S*)-1-(2,4-Dimethoxybenzyl)-3-((S)-2-hydroxy-1-phenylethyl)-5-phenyl-4-vinylimidazolidin-2-one (**19b**)

According to General Procedure C, the product was purified by silica gel chromatography (10% E.A. in DCM) to provide 158 mg (86%) of **19b** as a colorless foam as a single diastereomer. *R*_*f*_ = 0.31 (50% EtOAc/hexanes). ^1^H NMR (600 MHz, CDCl_3_) δ 7.33 (t, *J* = 7.5 Hz, 3H), 7.30–7.24 (m, 7H), 7.07 (d, *J* = 6.0 Hz, 2H), 7.03 (d, *J* = 8.2 Hz, 1H), 6.40 (dd, *J* = 8.2, 2.4 Hz, 1H), 6.38 (d, *J* = 2.4 Hz, 1H), 5.61 (ddd, *J* = 17.0, 10.1, 8.7 Hz, 1H), 5.18 (d, *J* = 10.1 Hz, 1H), 5.12 (t, *J* = 7.8 Hz, 1H), 4.86 (d, *J* = 17.0 Hz, 1H), 4.81 (d, *J* = 14.8 Hz, 1H), 4.34–4.24 (m, 2H), 4.04 (m, 1H), 3.98 (d, *J* = 7.7 Hz, 1H), 3.83 (d, *J* = 6.6 Hz, 2H), 3.80 (s, 3H), 3.63 (s, 3H), 3.46 (t, *J* = 8.2 Hz, 1H). ^13^C{1H} NMR (151 MHz, CDCl_3_) δ 161.1, 160.5, 158.7, 138.8, 137.8, 135.2, 131.5, 128.6, 128.5, 128.0, 127.6, 127.1, 120.8, 116.6, 104.0, 98.1, 66.4, 65.1, 63.6, 61.9, 55.3, 55.0, 40.6. HRMS (DART) *m*/*z* calcd for C_28_H_31_N_2_O_4_ [M + H]^+^: 459.2284; Found [M + H]^+^: 459.2304.

#### (4*S*,5*S*)-4-(4-Chlorophenyl)-1-((S)-2-hydroxy-1-phenylethyl)-3-(4-(trifluoromethyl)benzyl)-5-vinylimidazolidin-2-one (**19de**)

The reaction was set up according to general procedure C and stirred at 65 °C for 24 h. The product was purified by silica gel chromatography (5% E.A. in DCM) to provide 143 mg (71%) of **19de** as a colorless foam as a single diastereomer. *R*_*f*_ = 0.46 (50% EtOAc/hexanes). ^1^H NMR (600 MHz, CDCl_3_) δ 7.52 (d, *J* = 7.9 Hz, 2H), 7.31 (t, *J* = 7.4 Hz, 2H), 7.24 (dd, *J* = 8.5, 6.5 Hz, 5H), 7.19 (d, *J* = 7.9 Hz, 2H), 6.98 (d, *J* = 8.1 Hz, 2H), 5.59 (ddd, *J* = 17.1, 10.1, 8.7 Hz, 1H), 5.17 (d, *J* = 10.1 Hz, 1H), 4.89 (d, *J* = 15.1 Hz, 1H), 4.85 (d, *J* = 17.0 Hz, 1H), 4.65 (s, 1H), 4.29 (m, 2H), 4.06 (m, 1H), 3.90 (d, *J* = 7.9 Hz, 1H), 3.70 (d, *J* = 15.1 Hz, 1H), 3.48 (t, *J* = 8.4 Hz, 1H). ^13^C{1H} NMR (151 MHz, CDCl_3_) δ 160.7, 140.1, 137.4, 135.8, 134.6, 134.3, 130.33 (C–F, ^2^*J* C–F = 31.71 Hz), 130.11 (C–F, ^2^*J* C–F = 31.71 Hz), 129.90 (C–F, ^2^*J* C–F = 31.71 Hz), 129.68 (C–F, ^2^*J* C–F = 31.71 Hz), 129.2, 128.8, 128.79, 128.73, 127.9, 127.6, 126.77 (C–F, ^1^*J* C–F = 273.31 Hz), 125.72 (C–F, ^3^*J* C–F = 3.02 Hz), 125.69 (C–F, ^3^*J* C–F = 3.02 Hz), 125.67 (C–F, ^3^*J* C–F = 3.02 Hz), 125.64 (C–F, ^3^*J* C–F = 3.02 Hz), 124.97 (C–F, ^1^*J* C–F = 273.31 Hz), 123.16 (C–F, ^1^*J* C–F = 273.31 Hz), 121.7, 121.36 (C–F, ^1^*J* C–F = 273.31 Hz), 66.4, 64.6, 63.2, 61.8, 45.4. ^19^F NMR (565 MHz, CDCl_3_) δ −62.50. HRMS (DART) *m*/*z* calcd for C_27_H_25_ClF_3_N_2_O_2_ [M + H]^+^: 501.1557; Found [M + H]^+^: 501.1583.

#### (4*S*,5*S*)-4-(4-Fluorophenyl)-1-((S)-2-hydroxy-1-phenylethyl)-3-(4-(trifluoromethyl)benzyl)-5-vinylimidazolidin-2-one (**19ee**)

The reaction was set up according to general procedure C and stirred at 65 °C for 24 h. The product was purified by silica gel chromatography (5% E.A. in DCM) to provide 149 mg (74%) of **19ee** as a colorless foam as a single diastereomer. *R*_*f*_ = 0.40 (50% EtOAc/hexanes). ^1^H NMR (600 MHz, CDCl_3_) δ 7.45 (d, *J* = 7.9 Hz, 2H), 7.25 (t, *J* = 7.7 Hz, 2H), 7.20–7.14 (m, 3H), 7.11 (d, *J* = 7.9 Hz, 2H), 6.94 (dd, *J* = 8.4, 5.3 Hz, 2H), 6.88 (t, *J* = 8.5 Hz, 2H), 5.53–5.45 (ddd, *J* = 17.0, 10.1, 8.7 Hz, 1H), 5.10 (d, *J* = 10.1 Hz, 1H), 4.79 (t, *J* = 15.6 Hz, 2H), 4.58 (t, *J* = 6.5 Hz, 1H), 4.24–4.19 (m, 2H), 4.01–3.95 (m, 1H), 3.84 (d, *J* = 8.0 Hz, 1H), 3.64 (d, *J* = 15.1 Hz, 1H), 3.41 (t, *J* = 8.3 Hz, 1H). ^13^C{1H} NMR (151 MHz, CDCl_3_) δ 163.63 (C–F, ^1^*J* C–F = 249.15 Hz), 161.98 (C–F, ^1^*J* C–F = 249.15 Hz), 160.7, 140.2, 137.4, 134.4, 133.01 (C–F, ^3^*J* C–F = 3.02 Hz), 132.99 (C–F, ^3^*J* C–F = 3.02 Hz), 130.31 (C–F, ^2^*J* C–F = 31.71 Hz), 130.09 (C–F, ^2^*J* C–F = 31.71 Hz), 129.88 (C–F, ^2^*J* C–F = 31.71 Hz), 129.66 (C–F, ^2^*J* C–F = 31.71 Hz), 129.1, 129.0, 128.8, 128.7, 127.9, 127.6, 126.78 (C–F, ^1^*J* C–F = 273.31 Hz), 125.69 (C–F, ^3^*J* C–F = 3.02 Hz), 125.67 (C–F, ^3^*J* C–F = 3.02 Hz), 125.64 (C–F, ^3^*J* C–F = 3.02 Hz), 125.61 (C–F, ^3^*J* C–F = 3.02 Hz), 124.97 (C–F, ^1^*J* C–F = 273.31 Hz), 123.16 (C–F, ^1^*J* C–F = 273.31 Hz), 121.6, 121.35 (C–F, ^1^*J* C–F = 273.31 Hz), 116.09 (C–F, ^2^*J* C–F = 21.14 Hz), 115.95 (C–F, ^2^*J* C–F = 21.14 Hz), 66.6, 64.7, 63.2, 61.9, 45.3. ^19^F NMR (565 MHz, CDCl_3_) δ −62.52, −112.82. HRMS (DART) *m*/*z* calcd for C_27_H_25_F_4_N_2_O_2_ [M + H]^+^: 485.1852; Found [M + H]^+^: 485.1861.

#### (4*S*,5*S*)-1-Benzyl-5-(3-bromophenyl)-3-((S)-2-hydroxy-1-phenylethyl)-4-vinylimidazolidin-2-one (**19j**)

The reaction was set up according to general procedure C and stirred at 65 °C for 24 h. The product was purified by silica gel chromatography (5% E.A. in DCM) to provide 131 mg (69%) of **19j** as a colorless foam as a single diastereomer. *R*_*f*_ = 0.46 (50% EtOAc/hexanes). ^1^H NMR (600 MHz, CDCl_3_) δ 7.42 (d, *J* = 8.1 Hz, 1H), 7.36 (t, *J* = 7.5 Hz, 2H), 7.34–7.24 (m, 8H), 7.17 (t, *J* = 7.8 Hz, 1H), 7.11 (d, *J* = 6.6 Hz, 2H), 7.01 (d, *J* = 7.6 Hz, 1H), 5.59 (ddd, *J* = 17.0, 10.0, 8.7 Hz, 1H), 5.21 (d, *J* = 10.1 Hz, 1H), 4.98 (d, *J* = 14.9 Hz, 1H), 4.91 (d, *J* = 17.0 Hz, 1H), 4.82 (t, *J* = 7.0 Hz, 1H), 4.38–4.27 (m, 2H), 4.08–4.05 (m, 1H), 3.94 (d, *J* = 7.5 Hz, 1H), 3.64 (d, *J* = 14.9 Hz, 1H), 3.48 (t, *J* = 8.1 Hz, 1H). ^13^C{1H} NMR (151 MHz, CDCl_3_) δ 160.6, 140.3, 137.5, 135.9, 134.5, 131.6, 130.4, 130.2, 128.8, 128.7, 128.6, 127.9, 127.7, 127.6, 125.9, 123.0, 121.4, 66.2, 64.8, 62.8, 62.0, 45.7. HRMS (DART) *m*/*z* calcd for C_26_H_26_BrN_2_O_2_ [M + H]^+^: 477.1178; Found [M + H]^+^: 477.1207.

#### (4*S*,5*S*)-1-(2,4-Dimethoxybenzyl)-3-((S)-2-hydroxy-1-phenylethyl)-5-(pyridin-3-yl)-4-vinylimidazolidin-2-one (**19k**)

According to General Procedure C, the product was purified by silica gel chromatography (50% E.A. in DCM) to provide 183 mg (99%) of **19k** as a pale-yellow foam as a single diastereomer and as a 86:14 mixture of the rearranged **19k** to branched product **18k**. *R*_*f*_ = 0.10 (60% EtOAc/hexanes). ^1^H NMR (600 MHz, CDCl_3_) δ 8.50 (s, 1H), 8.26 (s, 1H), 7.44 (d, *J* = 6.0 Hz, 1H), 7.36–7.30 (m, 3H), 7.28–7.24 (m, 4H), 7.06 (d, *J* = 8.2 Hz, 1H), 6.40 (dd, *J* = 8.3, 2.4 Hz, 1H), 6.35 (d, *J* = 2.4 Hz, 1H), 5.58 (ddd, *J* = 17.1, 10.0, 8.7 Hz, 1H), 5.20 (d, *J* = 10.1 Hz, 1H), 4.90 (t, *J* = 6.9 Hz, 1H), 4.85 (d, *J* = 17.1 Hz, 1H), 4.76 (d, *J* = 14.6 Hz, 1H), 4.34–4.29 (m, 1H), 4.26 (dd, *J* = 7.8, 3.3 Hz, 1H), 4.07–4.02 (m, 1H), 3.97 (d, *J* = 8.1 Hz, 1H), 3.86 (dd, *J* = 18.0 6.0 Hz 2H), 3.79 (s, 3H), 3.60 (s, 3H), 3.43 (t, *J* = 8.4 Hz, 1H). ^13^C{1H} NMR (151 MHz, CDCl_3_) δ 161.1, 160.7, 158.6, 149.6, 149.1, 137.6, 134.5, 131.8, 129.1, 128.7, 127.85, 127.82, 127.6, 121.6, 116.1, 104.2, 98.1, 66.3, 64.8, 61.8, 61.5, 55.3, 55.0, 40.7. HRMS (DART) *m*/*z* calcd for C_27_H_30_N_3_O_4_ [M + H]^+^: 460.2236; Found [M + H]^+^: 460.2247.

#### (4*S*,5*S*)-1-(2,4-Dimethoxybenzyl)-3-((S)-2-hydroxy-1-phenylethyl)-5-(4-methoxyphenyl)-4-vinylimidazolidin-2-one (**19l**)

According to General Procedure C, the product was purified by silica gel chromatography (10% E.A. in DCM) to provide 184 mg (94%) of **19l** as a colorless foam as a single diastereomer. *R*_*f*_ = 0.29 (50% EtOAc/hexanes). ^1^H NMR (600 MHz, CDCl_3_) δ 7.35 (t, *J* = 7.7 Hz, 2H), 7.31–7.25 (m, 3H), 7.02 (d, *J* = 8.0 Hz, 1H), 6.99 (d, *J* = 8.4 Hz, 2H), 6.83 (d, *J* = 6.0 Hz, 2H), 6.42–6.40 (m, 2H), 5.64–5.56 (ddd, *J* = 17.0, 9.3, 8.6 Hz, 1H), 5.18 (d, *J* = 10.3 Hz, 1H), 5.15 (t, *J* = 7.1 Hz, 1H), 4.87 (d, *J* = 17.0 Hz, 1H), 4.79 (d, *J* = 14.8 Hz, 1H), 4.35–4.24 (m, 2H), 4.07–4.04 (m, 1H), 3.94 (d, *J* = 7.8 Hz, 1H), 3.81 (s, 3H), 3.79 (s, 3H), 3.67 (s, 3H), 3.46 (t, *J* = 8.2 Hz, 1H). ^13^C{1H} NMR (151 MHz, CDCl_3_) δ 161.0, 160.5, 159.4, 158.7, 137.9, 135.3, 131.4, 130.6, 128.6, 128.4, 127.7, 127.6, 120.8, 116.7, 113.9, 104.0, 98.1, 66.6, 65.1, 63.1, 61.8, 55.3, 55.2, 55.1, 40.5. HRMS (DART) *m*/*z* calcd for C_29_H_33_N_2_O_5_ [M + H]^+^: 489.2389; Found [M + H]^+^: 489.2387.

#### (4*S*,5*S*)-1-Benzyl-3-((S)-2-hydroxy-1-phenylethyl)-5-(2-methoxyphenyl)-4-vinylimidazolidin-2-one (**19m**)

The reaction was set up according to general procedure C and stirred at 65 °C for 24 h. The product was purified by silica gel chromatography (10% E.A. in DCM) to provide 118 mg (69%) of **19m** as a colorless foam as a single diastereomer. *R*_*f*_ = 0.37 (50% EtOAc/hexanes). ^1^H NMR (600 MHz, CDCl_3_) δ 7.26 (tdd, *J* = 14.3, 11.1, 7.6 Hz, 10H), 7.13 (d, *J* = 6.9 Hz, 3H), 6.94 (t, *J* = 7.5 Hz, 1H), 6.80 (d, *J* = 8.2 Hz, 1H), 5.68 (ddd, *J* = 16.8, 10.0, 8.3 Hz, 1H), 5.20–5.14 (m, 2H), 4.94 (dd, *J* = 16.1, 12.9 Hz, 2H), 4.50 (m, 1H), 4.33–4.27 (m, 2H), 4.06–4.00 (m, 1H), 3.70 (d, *J* = 15.0 Hz, 1H), 3.61 (s, 3H), 3.59 (m, 1H). ^13^C{1H} NMR (151 MHz, CDCl_3_) δ 160.7, 157.5, 138.0, 136.8, 135.6, 129.3, 128.5, 128.4, 127.6, 127.5, 127.3, 120.6, 119.7, 110.9, 65.2, 61.9, 55.1, 45.6. HRMS (DART) *m*/*z* calcd for C_27_H_29_N_2_O_3_ [M + H]^+^: 429.2178; Found [M + H]^+^: 429.2195.

#### (4*S*,5*S*)-4-(Benzo[d][1,3]dioxol-5-yl)-3-(2,4-dimethoxybenzyl)-1-((S)-2-hydroxy-1-phenylethyl)-5-vinylimidazolidin-2-one (**19n**)

According to General Procedure C, the product was purified by silica gel chromatography (10% E.A. in DCM) to provide 197 mg (98%) of **19n** as a colorless foam as a single diastereomer. *R*_*f*_ = 0.26 (50% EtOAc/hexanes). ^1^H NMR (600 MHz, CDCl_3_) δ 7.36–7.32 (m, 2H), 7.29–7.25 (m, 4H), 7.03 (d, *J* = 8.1 Hz, 1H), 6.68 (d, *J* = 7.9 Hz, 1H), 6.57 (s, 1H), 6.49 (d, *J* = 7.9 Hz, 1H), 6.42–6.38 (m, 2H), 5.95–5.91 (m, 2H), 5.61–5.53 (ddd, *J* = 17.1, 9.3, 8.5 Hz, 1H), 5.18 (d, *J* = 10.1 Hz, 1H), 5.06 (d, *J* = 7.9 Hz, 1H), 4.88 (d, *J* = 17.0 Hz, 1H), 4.77 (d, *J* = 14.8 Hz, 1H), 4.33–4.22 (m, 2H), 4.03 (m, 1H), 3.89 (d, *J* = 7.7 Hz, 1H), 3.83 (d, *J* = 14.8 Hz, 1H), 3.80 (s, 3H), 3.68 (s, 3H), 3.42 (t, *J* = 8.2 Hz, 1H). ^13^C{1H} NMR (151 MHz, CDCl_3_) δ 160.9, 160.5, 158.7, 147.4, 137.8, 135.2, 132.6, 131.4, 128.6, 127.6, 120.9, 120.8, 116.6, 108.0, 107.1, 104.0, 101.1, 98.1, 66.5, 65.1, 63.5, 61.8, 55.3, 55.1, 40.6. HRMS (DART) *m*/*z* calcd for C_29_H_31_N_2_O_6_ [M + H]^+^: 503.2182; Found [M + H]^+^: 503.2211.

#### (4*S*,5*S*)-1-(2,4-Dimethoxybenzyl)-5-(furan-2-yl)-3-((S)-2-hydroxy-1-phenylethyl)-4-vinylimidazolidin-2-one (**19s**)

According to General Procedure C, the product was purified by silica gel chromatography (5% E.A. in DCM) to provide 149 mg (83%) of **19s** as a colorless foam as a single diastereomer. *R*_*f*_ = 0.28 (50% EtOAc/hexanes). ^1^H NMR (600 MHz, CDCl_3_) δ 7.34–7.28 (m, 3H), 7.27–7.21 (m, 4H), 7.06 (d, *J* = 8.0 Hz, 1H), 6.41–6.37 (m, 2H), 6.26–6.23 (m, 1H), 6.08 (d, *J* = 3.2 Hz, 1H), 5.61 (ddd, *J* = 17.1, 10.1, 8.7 Hz, 1H), 5.16 (d, *J* = 10.1 Hz, 1H), 5.08–5.05 (m, 1H), 4.98 (d, *J* = 18.0 Hz, 1H), 4.71 (d, *J* = 15.0 Hz, 1H), 4.30–4.23 (m, 2H), 4.08 (d, *J* = 6.7 Hz, 1H), 3.98–3.94 (m, 1H), 3.85 (d, *J* = 15.0 Hz, 1H), 3.77 (s, 3H), 3.74–3.69 (m, 4H). ^13^C{1H} NMR (151 MHz, CDCl_3_) δ 160.5, 160.2, 158.6, 151.0, 142.8, 137.7, 135.1, 131.0, 128.6, 127.6, 127.6, 120.6, 116.8, 110.2, 108.7, 104.0, 98.3, 65.2, 62.7, 62.0, 57.1, 55.3, 55.2, 40.7. HRMS (DART) *m*/*z* calcd for C_26_H_29_N_2_O_5_ [M + H]^+^: 449.2076; Found [M + H]^+^: 449.2066.

#### (4*S*,5*S*)-1-(2,4-Dimethoxybenzyl)-3-((S)-2-hydroxy-1-phenylethyl)-5-(thiophen-2-yl)-4-vinylimidazolidin-2-one (**19t**)

According to General Procedure C, the product was purified by silica gel chromatography (3% E.A. in DCM) to provide 184 mg (99%) of **19t** as a colorless foam as a single diastereomer and as a 92:8 mixture of the rearranged **19t** to the branched product **18t**. *R*_*f*_ = 0.34 (50% EtOAc/hexanes). ^1^H NMR (600 MHz, CDCl_3_) δ 7.37–7.32 (m, 2H), 7.30–7.25 (m, 4H), 7.24 (d, *J* = 5.1 Hz, 1H), 7.08–7.04 (m, 1H), 6.92 (t, *J* = 6.0 Hz, 1H), 6.76 (d, *J* = 3.5 Hz, 1H), 6.43 (m, 2H), 5.63–5.54 (ddd, *J* = 17.0, 10.1, 8.6 Hz, 1H), 5.21 (d, *J* = 10.1 Hz, 1H), 5.06 (t, *J* = 7.0 Hz, 1H), 4.98 (d, *J* = 18.0 Hz, 1H), 4.82 (d, *J* = 14.8 Hz, 1H), 4.35–4.25 (m, 3H), 4.01 (m, 1H), 3.94 (d, *J* = 14.9 Hz, 1H), 3.81 (s, 3H), 3.74 (s, 3H), 3.59 (d, *J* = 8.7 Hz, 1H). ^13^C{1H} NMR (151 MHz, CDCl_3_) δ 160.6, 160.4, 158.7, 142.9, 137.6, 135.0, 131.4, 128.7, 127.7, 127.6, 126.6, 125.8, 125.7, 121.1, 116.5, 104.0, 98.2, 66.7, 65.1, 62.0, 59.2, 55.3, 55.1, 40.8. HRMS (DART) *m*/*z* calcd for C_26_H_29_N_2_O_4_S [M + H]^+^: 465.1848; Found [M + H]^+^: 465.1881.

#### (4*S*,5*S*)-1-((S)-2-Hydroxy-1-phenylethyl)-4-(naphthalen-2-yl)-3-(4-(trifluoromethyl)benzyl)-5-vinylimidazolidin-2-one (**19pe**)

The reaction was set up according to general procedure C and stirred at 65 °C for 24 h. The product was purified by silica gel chromatography (5% E.A. in DCM) to provide 130 mg (79%) of **19pe** as a colorless foam as a single diastereomer. *R*_*f*_ = 0.43 (50% EtOAc/hexanes). ^1^H NMR (600 MHz, CDCl_3_) δ 7.59–7.56 (m, 1H), 7.55 (d, *J* = 8.5 Hz, 1H), 7.52–7.49 (m, 1H), 7.29 (d, *J* = 8.0 Hz, 2H), 7.27–7.22 (m, 3H), 7.11 (t, *J* = 7.6 Hz, 2H), 7.08–7.02 (m, 3H), 7.01–6.95 (m, 4H), 5.46–5.38 (ddd, *J* = 17.2, 10.0, 8.6 Hz, 1H), 4.96 (d, *J* = 10.1 Hz, 1H), 4.72 (d, *J* = 15.1 Hz, 1H), 4.62 (d, *J* = 17.0 Hz, 1H), 4.13–4.09 (m, 2H), 3.90 (d, *J* = 7.8 Hz, 1H), 3.86 (d, *J* = 8.9 Hz, 1H), 3.53 (d, *J* = 15.2 Hz, 1H), 3.42 (t, *J* = 8.2 Hz, 1H). ^13^C{1H} NMR (151 MHz, CDCl_3_) δ 160.8, 140.4, 137.5, 134.64, 134.62, 133.4, 133.1, 130.2 (C–F, ^2^*J* C–F = 31.71 Hz), 130.01 (C–F, ^2^*J* C–F = 31.71 Hz), 129.80 (C–F, ^2^*J* C–F = 31.71 Hz), 129.58 (C–F, ^2^*J* C–F = 31.71 Hz), 129.2, 128.9, 128.7, 127.9, 127.86, 127.81, 127.7, 127.1, 126.82 (C–F, ^1^*J* C–F = 273.31 Hz), 126.68, 126.62, 125.66 (C–F, ^3^*J* C–F = 3.02 Hz), 125.63 (C–F, ^3^*J* C–F = 3.02 Hz), 125.61 (C–F, ^3^*J* C–F = 3.02 Hz), 125.58 (C–F, ^3^*J* C–F = 3.02 Hz), 125.02 (C–F, ^1^*J* C–F = 273.31 Hz), 124.2, 123.21 (C–F, ^1^*J* C–F = 273.31 Hz), 121.5, 121.41 (C–F, ^1^*J* C–F = 273.31 Hz), 66.3, 64.8, 64.0, 62.0, 45.4. ^19^F NMR (565 MHz, CDCl_3_) δ −62.47. HRMS (DART) *m*/*z* calcd for C_31_H_28_F_3_N_2_O_2_ [M + H]^+^: 517.2103; Found [M + H]^+^: 517.2121.

#### (4*S*,5*S*)-1-Benzyl-3-((S)-2-hydroxy-1-phenylethyl)-5-(naphthalen-2-yl)-4-vinylimidazolidin-2-one (**19pc**)

The reaction was set up according to general procedure C and stirred at 65 °C for 24 h. The product was purified by silica gel chromatography (5% E.A. in DCM) to provide 125 mg (70%) of **19pc** as a colorless foam as a single diastereomer. *R*_*f*_ = 0.60 (20% EtOAc/DCM). ^1^H NMR (600 MHz, CDCl_3_) δ 7.81–7.71 (m, 3H), 7.49 (s, 1H), 7.48–7.43 (m, 2H), 7.34–7.19 (m, 9H), 7.10 (d, *J* = 12.0 Hz, 2H), 5.62 (ddd, *J* = 17.0, 10.1, 8.7 Hz, 1H), 5.15 (d, *J* = 10.1 Hz, 1H), 5.00 (d, *J* = 14.9 Hz, 1H), 4.82 (d, *J* = 17.0 Hz, 1H), 4.35–4.27 (m, 2H), 4.14 (d, *J* = 7.8 Hz, 1H), 4.08 (dd, *J* = 11.2, 2.5 Hz, 1H), 3.63–3.58 (m, 2H). ^13^C{1H} NMR (151 MHz, CDCl_3_) δ 160.9, 137.7, 136.2, 135.1, 134.9, 133.3, 133.1, 129.0, 128.74, 128.70, 128.6, 127.9, 127.8, 127.79, 127.74, 127.6, 127.1, 126.5, 126.4, 124.3, 121.3, 66.3, 64.9, 63.5, 62.0, 45.6. HRMS (DART) *m*/*z* calcd for C_30_H_29_N_2_O_2_ [M + H]^+^: 449.2229; Found [M + H]^+^: 449.2259.

#### (4*S*,5*S*)-1-(4-Fluorobenzyl)-3-((S)-2-hydroxy-1-phenylethyl)-5-(naphthalen-2-yl)-4-vinylimidazolidin-2-one (**19pd**)

The reaction was set up according to general procedure C and stirred at 65 °C for 24 h. The product was purified by silica gel chromatography (5% E.A. in DCM) to provide 132 mg (71%) of **19pd** as a colorless foam as a single diastereomer. *R*_*f*_ = 0.62 (20% EtOAc/DCM). ^1^H NMR (600 MHz, CDCl_3_) δ 7.85–7.75 (m, 3H), 7.54–7.48 (m, 3H), 7.36 (t, *J* = 7.6 Hz, 2H), 7.33–7.26 (m, 3H), 7.25–7.22 (m, 1H), 7.09 (dd, *J* = 8.4, 5.5 Hz, 2H), 6.98 (t, *J* = 8.6 Hz, 2H), 5.69 (ddd, *J* = 17.0, 10.2, 8.6 Hz, 1H), 5.20 (d, *J* = 10.1 Hz, 1H), 4.96 (d, *J* = 14.9 Hz, 1H), 4.91 (t, *J* = 6.1 Hz, 1H), 4.86 (d, *J* = 17.0 Hz, 1H), 4.39–4.30 (m, 2H), 4.15–4.08 (m, 2H), 3.68–3.61 (m, 2H). ^13^C{1H} NMR (151 MHz, CDCl_3_) δ 163.12 (C–F, ^1^*J* C–F = 246.13 Hz), 161.49 (C–F, ^1^*J* C–F = 246.13 Hz), 160.8, 137.6, 134.8, 134.7, 133.3, 133.1, 130.46, 132.02 (C–F, ^3^*J* C–F = 4.53 Hz), 131.99 (C–F, ^3^*J* C–F = 4.53 Hz), 130.46, 130.41, 129.1, 128.7, 127.87, 127.80, 127.7, 127.1, 126.6, 126.5, 124.3, 121.4, 115.60 (C–F, ^2^*J* C–F = 21.14 Hz), 115.46 (C–F, ^2^*J* C–F = 21.14 Hz), 66.3, 64.9, 63.6, 62.0, 45.0. ^19^F NMR (565 MHz, CDCl_3_) δ – 114.61. HRMS (DART) *m*/*z* calcd for C_30_H_28_FN_2_O_2_ [M + H]^+^: 467.2135; Found [M + H]^+^: 467.2105.

### Synthesis of **29** from Achiral Allenamide **15b**

To a 20 mL crimp cap vial with a stir bar in an Ar filled glovebox were charged Cu(OAc)_2_ (1.8 mg, 10 μmol) and Ph-BPE (5.1 mg, 10 μmol) followed by toluene (2.5 mL) and *tert*-butanol (26.3 μL, 275 μmol). The mixture was stirred for 5 min. Allenamide **15b** (37.5 mg, 300 μmol) followed by imine **9a** (250 μmol) was then charged, and the vial was sealed with a crimp-cap septum and removed from the glovebox. Dimethoxymethylsilane (0.061 mL, 2 equiv) was charged to the reaction mixture, and the reaction mixture was stirred at rt for 24 h. The reaction was quenched by addition of 100 mg of NH_4_F and 1.5 mL of MeOH followed by agitation at rt for 30 min. A 5 mL volume of 5% NaHCO_3_ was then added to the mixture followed by extraction with DCM (2 × 3 mL). The combined organics were dried with Na_2_SO_4_, filtered, and concentrated *in vacuo*. Crude product was purified by flash chromatography on silica gel (25% EtOAc/hexanes) to afford 78 mg (69%) of **29** as a white solid as a single diastereomer and as a 57:43 mixture of enantiomers as determined via chiral HPLC analysis (Chiracel AD-3 85:15 heptane/isopropanol 1.50 mL/min, 254 nm). *R*_*f*_ = 0.45 (50% EtOAc/hexanes). ^1^H NMR (600 MHz, CDCl_3_) δ 7.60 (d, *J* = 7.8 Hz, 2H), 7.47 (d, *J* = 7.9 Hz, 2H), 6.91 (d, *J* = 8.2 Hz, 1H), 6.44 (d, *J* = 2.3 Hz, 1H), 6.40 (dd, *J* = 8.1, 2.4 Hz, 1H), 5.47 (ddd, *J* = 17.2, 10.5, 6.9 Hz, 1H), 5.05 (d, *J* = 10.6 Hz, 1H), 4.99 (d, *J* = 17.2 Hz, 1H), 4.46 (t, *J* = 6.0 Hz, 1H), 4.29 (q, *J* = 12.0 Hz, 1H), 4.21 (q, *J* = 12.0 Hz, 1H), 3.79 (s, 6H), 3.75 (d, *J* = 9.6 Hz, 1H), 3.71 (d, *J* = 13.7 Hz, 1H), 3.42 (q, *J* = 8.1 Hz, 1H), 3.25 (d, *J* = 13.7 Hz, 1H), 3.16 (td, *J* = 8.7, 6.4 Hz, 1H). ^13^C{1H} NMR (151 MHz, CDCl_3_) δ 160.3, 158.7, 158.6, 144.8, 131.4, 130.7, 130.6, 130.2 (q, *J* = 31.71 Hz), 128.9, 128.7, 125.3 (q, *J* = 3.02 Hz), 119.7, 103.6, 98.6, 98.6, 62.2, 62.1, 61.7, 60.9, 55.3, 55.2, 46.1, 40.8. ^19^F NMR (565 MHz, CDCl_3_) δ −62.36. HRMS (DART) *m*/*z* calcd for C_23_H_26_F_3_N_2_O_4_ [M + H]^+^: 451.1845; Found [M + H]^+^: 451.1881.

### Synthesis of **30** from Achiral Allenamide **15b**

To a 20 mL crimp cap vial with a stir bar in an Ar filled glovebox were charged Cu(OAc)_2_ (1.8 mg, 10 μmol) and Ph-BPE (5.1 mg, 10 μmol) followed by toluene (0.5 mL). The mixture was stirred for 5 min. Allenamide **15b** (37.5 mg, 300 μmol) followed by imine **9a** (250 μmol) was then charged, and the vial was sealed with a crimp-cap septum and removed from the glovebox. Dimethoxymethylsilane (0.061 mL, 2 equiv) was then charged to the reaction mixture. The mixture was then stirred at rt for 24 h. The reaction was quenched by addition of 100 mg of NH_4_F and 1.5 mL of MeOH followed by agitation at rt for 30 min. A 5 mL volume of 5% NaHCO_3_ was then added to the mixture followed by extraction with DCM (2 × 3 mL). The combined organics were dried with Na_2_SO_4_, filtered, and concentrated in vacuo. Crude product was purified by flash chromatography on silica gel (50% EtOAc/hexanes) to afford 68 mg (60%) of **30** as a colorless liquid and as an 80:20 mixture of enantiomers as determined via chiral HPLC analysis (Chiracel AD-3 90:10 heptane/isopropanol 1.00 mL/min, 220 nm). *R*_*f*_ = 0.28 (50% EtOAc/hexanes). ^1^H NMR (600 MHz, CDCl_3_) δ 7.59 (d, *J* = 8.0 Hz, 2H), 7.28 (d, *J* = 8.0 Hz, 2H), 7.00 (d, *J* = 8.3 Hz, 1H), 6.37 (dd, *J* = 8.3, 2.4 Hz, 1H), 6.32 (d, *J* = 2.4 Hz, 1H), 5.62 (ddd, *J* = 17.1, 10.1, 8.6 Hz, 1H), 5.22 (d, *J* = 10.1 Hz, 1H), 5.04 (d, *J* = 17.0 Hz, 1H), 4.70 (d, *J* = 14.7 Hz, 1H), 4.01 (d, *J* = 8.2 Hz, 1H), 3.86 (d, *J* = 14.7 Hz, 1H), 3.78 (s, 3H), 3.76–3.68 (m, 2H), 3.63 (t, *J* = 8.4 Hz, 1H), 3.55 (s, 3H), 3.36–3.31 (m, 1H), 3.26–3.22 (m, 1H). ^13^C{1H} NMR (151 MHz, CDCl_3_) δ 162.3, 160.6, 158.6, 142.7, 135.0, 131.7, 130.6 (q, *J* = 31.71 Hz), 127.6, 126.7 (q, *J* = 273.31 Hz), 125.5 (q, *J* = 3.02 Hz), 121.2, 116.2, 104.1, 98.0, 68.2, 63.8, 62.2, 55.3, 54.8, 46.1, 40.8. ^19^F NMR (565 MHz, CDCl_3_) δ −62.50. HRMS (DART) *m*/*z* calcd for C_23_H_26_F_3_N_2_O_4_ [M + H]^+^: 451.1845; Found [M + H]^+^: 451.1882.

### Synthesis of **18c** on 1.0 g Scale

To a 20 mL crimp cap vial was charged Cu(OAc)_2_ (16.1 mg, 88.9 μmol), and the vial was sealed with a crimp-cap septum. The vial was evacuated and backfilled with nitrogen 3 times and then charged with toluene (5 mL), 20% PCy_3_ solution in toluene (194 μL, 111 μmol), and *tert*-butanol (0.85 mL, 8.89 mmol), and the mixture was allowed to stir at rt for 10 min until all the Cu(OAc)_2_ dissolved. A 50 mL two-neck round-bottom flask was then charged with imine **9c** (1.0 g, 4.44 mmol) and allene **15a** (1.07 g, 5.33 mmol), and the flask was evacuated and backfilled with nitrogen 3 times. The flask was then charged with toluene (5 mL). The imine/allene flask was then charged with the catalyst solution. Dimethoxymethyl silane (1.1 mL, 8.89 mmol) was charged to the reaction mixture *(**caution:**dimethoxymethylsilane should be handled in a well-ventilated fume hood because it is known to cause blindness. Syringes were quenched with 2 M NaOH, gas evolution! prior to disposal)*, and the reaction was allowed to stir at rt for 2 h. A 50 mL round-bottom flask was charged with NH_4_F (2 g) and MeOH (20 mL), and the reaction mixture was transferred via pipet to this flask and allowed to stir at rt for 30 min. The volatiles were concentrated *in vacuo*, and 50 mL of 5% NaHCO_3_ solution were added to the flask. The mixture was extracted with CH_2_Cl_2_ (2 × 20 mL), and the combined organics were dried over Na_2_SO_4_ and concentrated *in vacuo*. The crude residue was purified by silica gel chromatography (5% EtOAc/DCM) to afford 1.655 g (91%) of **18c** as a white solid as a single diastereomer.

### Synthesis of **25**

To a solution of 207.5 mg (393 μmol) of **19a** in 2 mL of CH_2_Cl_2_ at 0 °C were charged 66 μL (473 μmol) of triethylamine followed by dropwise addition of 30.5 μL (393 μmol) of MsCl. The mixture was stirred for 30 min at 0 °C, and then 4 mL of 10% NH_4_Cl were added. The mixture was extracted with CH_2_Cl_2_ (3 × 5 mL). The combined organics were dried with anhydrous Na_2_SO_4_ and filtered, and the volatiles were removed *in vacuo*. The crude residue was then dissolved in 2 mL of THF and cooled to 0 °C. A 1.0 M concentration of potassium *tert*-butoxide (433 μL, 433 μmol) in THF was then added, and the mixture was warmed to room temperature and stirred for 30 min. To the mixture was added 5 mL of 10% brine followed by extraction with CH_2_Cl_2_ (3 × 5 mL). The combined organics were dried with anhydrous Na_2_SO_4_ and filtered, and the volatiles were removed *in vacuo*. The crude residue was then dissolved in 4 mL of THF in a crimp cap vial. To the solution were then added 788 μL (3.94 mmol) of 5.0 M aqueous H_2_SO_4_. The vial was purged with argon, sealed, and immersed in an oil bath at 50 °C. After 3 h the reaction mixture was cooled to room temperature, and 15 mL of saturated aqueous NaHCO_3_ were added. The mixture was extracted with CH_2_Cl_2_ (3 × 5 mL). The combined organics were dried with anhydrous Na_2_SO_4_ and filtered, and the volatiles were removed *in vacuo*. The crude residue was purified by flash chromatography (20% EtOAc/DCM) to afford 112 mg (70%) of **25** as a colorless foam. *R*_*f*_ = 0.22 (50% EtOAc/hexanes). ^1^H NMR (600 MHz, CDCl_3_) δ 7.59 (d, *J* = 8.0 Hz, 2H), 7.30 (d, *J* = 7.9 Hz, 2H), 7.02 (d, *J* = 8.3 Hz, 1H), 6.37 (d, *J* = 8.3 Hz, 1H), 6.31 (d, *J* = 2.4 Hz, 1H), 5.74 (ddd, *J* = 17.2, 10.2, 7.2 Hz, 1H), 5.41 (s, 1H), 5.12 (d, *J* = 10.2 Hz, 1H), 5.10 (d, *J* = 12 Hz, 1H), 4.67 (d, *J* = 14.8 Hz, 1H), 4.06 (d, *J* = 7.5 Hz, 1H), 3.86–3.82 (m, 2H), 3.76 (s, 3H), 3.54 (s, 3H). ^13^C{1H} NMR (151 MHz, CDCl_3_) δ 161.6, 160.5, 158.5, 143.2, 136.1, 131.4, 130.6 (q, *J* = 33.22 Hz), 127.5, 126.7 (q, *J* = 271.8 Hz), 125.5 (q, *J* = 3.02 Hz), 118.0, 116.5, 104.1, 97.9, 65.5, 62.1, 55.3, 54.9, 39.8. ^19^F NMR (565 MHz, CDCl_3_) δ −62.49. HRMS (DART) *m*/*z* calcd for C_21_H_22_F_3_N_2_O_3_ [M + H]^+^: 407.1583; Found [M + H]^+^: 407.1600.

### Synthesis of **19a** from **18a**

To a solution of 100 mg (190 μmol) of **18a** in 1.0 mL of THF at −10 °C were added 114 μL (285 μmol) of 2.5 M solution of ^*n*^BuLi in hexanes. The reaction mixture was allowed to warm to room temperature and stirred for 1 h. To the mixture were added 2 mL of saturated NH_4_Cl, and the mixture was extracted with CH_2_Cl_2_ (3 × 3 mL). The combined organics were dried with anhydrous Na_2_SO_4_ and filtered, and the volatiles were removed *in vacuo*. The crude residue was purified by flash chromatography (5% EtOAc/DCM) to afford 94 mg (94%) of **19a** as a colorless foam.

### Synthesis of **26**

A crimp cap vial was charged with 100 mg (233 μmol) of **18c**, CH_3_CN (1 mL), K_2_CO_3_ (161 mg, 1.17 mmol), TBAI (17.2 mg, 46.7 μmol), and allyl bromide (101 μL, 1.17 mmol). The mixture was heated at 85 °C for 18 h. The reaction was quenched with 5 mL of water, and the mixture was extracted with MTBE (3 × 3 mL). The combined organics were dried with anhydrous Na_2_SO_4_ and filtered, and the volatiles were removed *in vacuo*. The crude residue was purified by flash chromatography (20% EtOAc/hexanes) to afford 90 mg (82%) of **26** as a yellow solid. *R*_*f*_ = 0.60 (50% EtOAc/hexanes). ^1^H NMR (600 MHz, CDCl_3_) δ 7.38–7.31 (m, 4H), 7.29–7.25 (m, 3H), 7.25–7.20 (m, 3H), 7.18 (d, *J* = 6.0 Hz, 2H), 6.98 (d, *J* = 8.5 Hz, 2H), 6.93–6.88 (m, 2H), 5.95 (dtd, *J* = 17.2, 9.5, 4.0 Hz, 1H), 5.25 (d, *J* = 10.1 Hz, 1H), 5.20 (d, *J* = 17.2 Hz, 1H), 5.12–5.05 (m, 1H), 4.88–4.82 (m, 1H), 4.61–4.52 (m, 2H), 4.52–4.45 (m, 2H), 4.08 (dd, *J* = 7.4, 5.3 Hz, 1H), 3.87 (d, *J* = 11.7 Hz, 1H), 3.85 (s, 3H), 3.82 (d, *J* = 13.3 Hz, 1H), 3.66–3.61 (m, 1H), 2.96 (d, *J* = 13.2 Hz, 1H), 2.56 (dd, *J* = 13.3, 9.1 Hz, 1H). ^13^C{1H} NMR (151 MHz, CDCl_3_) δ 159.0, 158.7, 140.1, 137.1, 134.7, 131.8, 131.0, 130.0, 128.7, 128.6, 128.0, 127.8, 127.6, 119.2, 118.0, 113.8, 70.4, 62.4, 56.9, 56.7, 55.4, 52.9, 52.5. HRMS (DART) *m*/*z* calcd for C_30_H_33_N_2_O_3_ [M + H]^+^: 469.2491; Found [M + H]^+^: 469.2504.

### Synthesis of **27**

To a 20 mL crimp cap vial with a stir bar in an Ar filled glovebox were added 48 mg (0.10 mmol) of **26** followed by 2 mL of toluene and 3.2 mg (5.1 μmol) of a Hoveyda–Grubbs II catalyst. The vial was sealed and removed from the glovebox. The solution was heated at 90 °C for 12 h. The reaction mixture was concentrated, and the crude residue was purified by flash chromatography (50% EtOAc/hexanes) to afford 35 mg (78%) of **27** as a colorless foam. ^1^H NMR (600 MHz, CDCl_3_) δ 7.44 (d, *J* = 7.3 Hz, 2H), 7.39 (t, *J* = 7.5 Hz, 2H), 7.35–7.31 (m, 1H), 7.28–7.21 (m, 6H), 7.15 (d, *J* = 6.0 Hz, 2H), 6.88 (d, *J* = 7.9 Hz, 2H), 5.41–5.37 (m, 1H), 5.20 (dd, *J* = 8.7, 2.8 Hz, 1H), 5.15–5.09 (m, 1H), 4.72–4.68 (m, 1H), 4.34 (t, *J* = 6.0 Hz, 1H), 3.99 (d, *J* = 4.4 Hz, 1H), 3.97 (s, 1H), 3.96–3.93 (m, 1H), 3.80 (s, 3H), 3.24 (dt, *J* = 17.9, 2.5 Hz, 1H), 2.95 (d, *J* = 13.2 Hz, 1H), 2.76 (dd, *J* = 18.0, 2.8 Hz, 1H). ^13^C{1H} NMR (151 MHz, CDCl_3_) δ 158.7, 157.9, 142.8, 138.2, 130.7, 129.5, 128.8, 128.7, 128.6, 128.5, 128.1, 128.0, 126.4, 123.2, 113.8, 70.7, 67.8, 59.4, 59.3, 55.3, 53.5, 51.6. HRMS (DART) *m*/*z* calcd for C_28_H_29_N_2_O_3_ [M + H]^+^: 441.2178; Found [M + H]^+^: 441.2205.

## Supporting Information Available

Computational details, intermediate and transition state coordinates, copies of ^1^H and ^13^C NMR spectra of all new compounds, and X-ray diffraction analysis data of **18c·HCl** (PDF)

## Accession Codes

CCDC 2045972 contains the supplementary crystallographic data for this paper. These data can be obtained free of charge via www.ccdc.cam.ac.uk/data_request/cif, or by emailing data_request@ccdc.cam.ac.uk, or by contacting The Cambridge Crystallographic Data Centre, 12 Union Road, Cambridge CB2 1EZ, UK; fax: +44 1223 336033.
