# Supplementary material for: Access to Chiral Diamine Derivatives through Stereoselective Cu-Catalyzed Reductive Coupling of Imines and Allenamides
Source: J Org Chem. 2021 Mar 16;86(7):5026–46. doi: 10.1021/acs.joc.0c02971 (PMC8025098; doi:10.1021/acs.joc.0c02971)
Supplement: Supplementary file 1 — jo0c02971_si_001.pdf [file jo0c02971_si_001.pdf]

Supporting Information

Access to Chiral Diamine Derivatives Through Stereoselective Cu-Catalyzed Reductive Coupling of Imines and Allenamides

Toolika Agrawal,[a] Robert T. Martin[b] Stephen Collins,[a] Zachary Wilhelm,[b] Mytia D. Edwards,[a] Osvaldo Gutierrez*[b] and Joshua D. Sieber*,[a]

[a]*Department of Chemistry, Virginia Commonwealth University, 1001 West Main Street Richmond, VA 23284-3208, USA.*
[b]*Department of Chemistry and Biochemistry, University of Maryland, College Park, Maryland, 20742, USA*

ogs@umd.edu
jdsieber@vcu.edu

Table of contents:

Crystal structure of **18c.HCl**.......... S2

Density functional theory calculations and coordinates.......... S4-S212

References.......... S213

^{1}H and ^{13}C NMR data.......... S214-S271

Crystal Structure of 18c·HCl at 50% ellipsoid probabilities

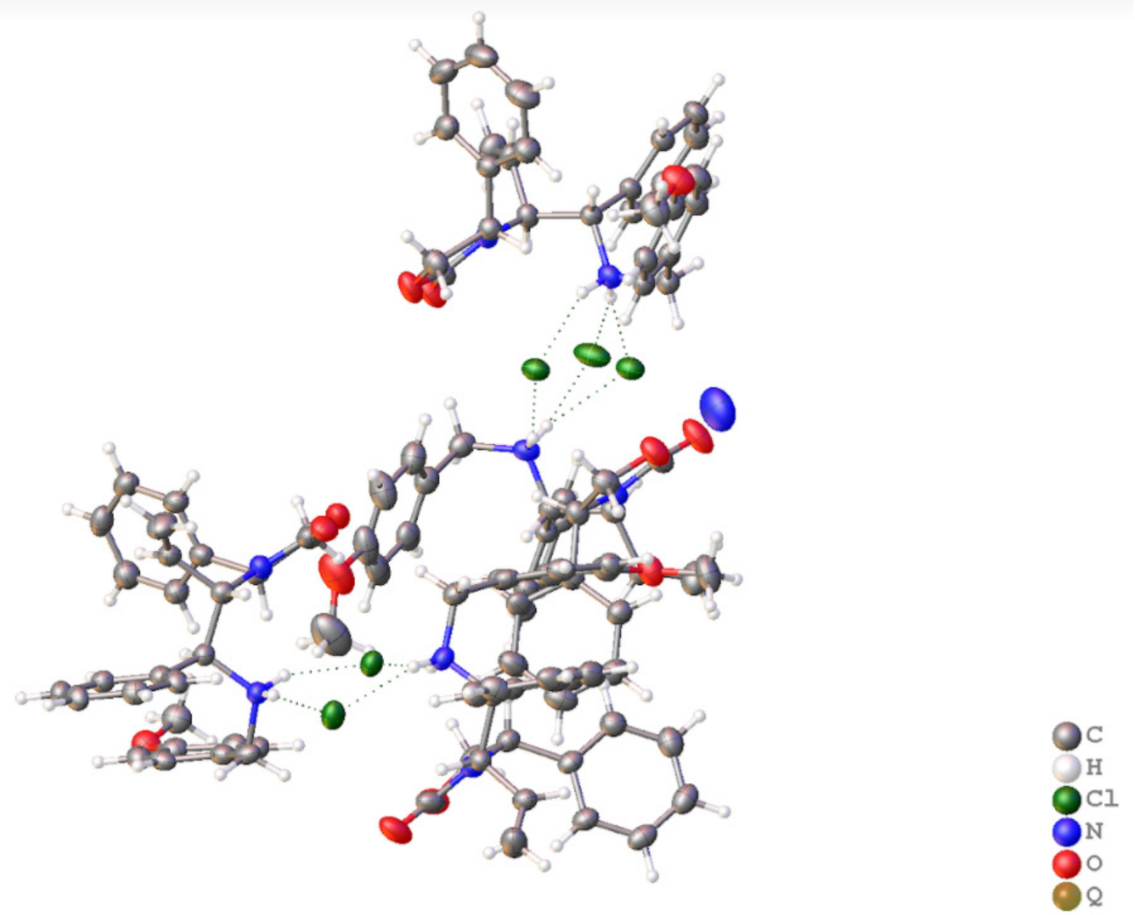

Single crystals of **18c·HCl** were prepared by vapour diffusion crystallization from ethyl acetate/hexanes at -20 °C. A suitable crystal 0.39×0.32×0.12 mm[3] was selected and MiTeGen microloop on an XtaLAB AFC11 (RINC): quarter-chi single diffractometer. The crystal was kept at a steady T = 100.00(10) K during data collection. The structure was solved with the ShelXT 2014/4[1] structure solution program using the Intrinsic Phasing solution method and by using **Olex2**[2,3] as the graphical interface. The model was refined with version 2016/6 of ShelXL 2016/6[4] using Least Squares minimisation. The structure was solved and the space group $P2_1$ (# 4). All non-hydrogen atoms were refined anisotropically. Hydrogen atom positions were calculated geometrically and refined using the riding model. Most hydrogen atom positions were calculated geometrically and refined using the riding model, but some hydrogen atoms were refined freely.

Data reduction, scaling and absorption corrections were performed using CrysAlisPro (Rigaku, V1.171.41.69a, 2020).[5] The final completeness is 97.00 % out to 67.077° in Θ. A multi-scan absorption correction was performed using CrysAlisPro 1.171.41.69a (Rigaku, Oxford Diffraction, 2020)[5] using spherical harmonics as implemented in SCALE3 ABSPACK.. The absorption coefficient μ of this material is 1.593 mm^{-1} at this wavelength (λ = 1.542Å) and the minimum and maximum transmissions are 0.759 and 1.000. Data were measured using ω scans of 0.5° per frame for 10.0/0.5/0.7 s using Cu K_α radiation. The total number of runs and images was based on the strategy calculation from the program CrysAlisPro (Rigaku, V1.171.41.69a, 2020).[5] The maximum resolution that was achieved was Θ = 67.077° (0.84 Å). The diffraction pattern was indexed. The total number of runs and images was based on the strategy calculation from the program CrysAlisPro (Rigaku, V1.171.41.69a, 2020)[5] and the unit cell was refined using CrysAlisPro (Rigaku, V1.171.41.69a, 2020)[15] on 18869 reflections, 44% of the observed reflections.

Crystallographic data and the structural refinement parameters for the compound **18c·HCl** are given in the Table S1. Crystallographic data have been deposited with the Cambridge Crystallographic Data Centre (CCDC) with the depository number: CCDC 2045972. This data is available from the CCDC *via* www.ccdc.cam.ac.uk/.

Table S1. Selected crystallographic data for 18c·HCl.

Formula	$C_{108}H_{118}Cl_4N_8O_{13}$
$D_{calc.}$/ g cm^{-3}	1.240
m/mm^{-1}	1.593
Formula Weight	1877.90
Colour	clear light colourless
Shape	plate
Size/mm^3	0.39×0.32×0.12
T/K	100.00(10)
Crystal System	monoclinic
Flack Parameter	0.035(8)
Hooft Parameter	0.038(5)
Space Group	$P2_1$
a/Å	13.4818(3)
b/Å	19.0641(3)
c/Å	20.4740(3)
a/°	90
b/°	107.045(2)
g/°	90
V/Å^3	5031.05(16)
Z	2
Z'	1
Wavelength/Å	1.54184
Radiation type	Cu K_a
Q_{min}/°	2.257
Q_{max}/°	67.077
Measured Refl.	42799
Independent Refl.	16324
Reflections with I > 2(I)	14783
R_{int}	0.0538
Parameters	1247
Restraints	2164
Largest Peak	0.400
Deepest Hole	-0.366
GooF	1.058
wR_2 (all data)	0.1441
wR_2	0.1364
R_1 (all data)	0.0553
R_1	0.0502

Density Functional Theory (DFT) Calculations

General Remarks

All optimizations of intermediates and transition states were calculated using B3LYP/def2-SVP level of the theory, with dispersion correction ("empiricaldispersion=gd3" keyword) in implicit solvent (toluene) using CPCM as the solvation model with the "guess=mix" keyword and with a ultrafine grid size, as implemented in Gaussian16. Frequency calculations (using the same level of theory) were used to obtain thermal corrections (at 298K) and to characterize optimized structures as transition states (if only a single imaginary frequency was found) or intermediates (if no imaginary frequencies were found). Single point energy calculations in the gas phase using M06-L/def2-SVP and B3LYP-D3/def2-TZVPP with an ultrafine grid size were also performed to compare energetics.[6] All 3-D structures were generated using CYLview.[7] In order to elucidate the mechanism of this reaction and determine the lowest energy pathway, we performed exhaustive conformational searches for the key transition states: hydrocupration, isomerization, and C-C bond formation. These conformational searches and additional computational results are outlined in supplementary figures **S1** through **S5** below.

Full Reference of Gaussian16 software:

Gaussian 16, Revision C.01, M. J. Frisch, G. W. Trucks, H. B. Schlegel, G. E. Scuseria, M. A. Robb, J. R. Cheeseman, G. Scalmani, V. Barone, G. A. Petersson, H. Nakatsuji, X. Li, M. Caricato, A. V. Marenich, J. Bloino, B. G. Janesko, R. Gomperts, B. Mennucci, H. P. Hratchian, J. V. Ortiz, A. F. Izmaylov, J. L. Sonnenberg, D. Williams-Young, F. Ding, F. Lipparini, F. Egidi, J. Goings, B. Peng, A. Petrone, T. Henderson, D. Ranasinghe, V. G. Zakrzewski, J. Gao, N. Rega, G. Zheng, W. Liang, M. Hada, M. Ehara, K. Toyota, R. Fukuda, J. Hasegawa, M. Ishida, T. Nakajima, Y. Honda, O. Kitao, H. Nakai, T. Vreven, K. Throssell, J. A. Montgomery, Jr., J. E. Peralta, F. Ogliaro, M. J. Bearpark, J. J. Heyd, E. N. Brothers, K. N. Kudin, V. N. Staroverov, T. A. Keith, R. Kobayashi, J. Normand, K. Raghavachari, A. P. Rendell, J. C. Burant, S. S. Iyengar, J. Tomasi, M. Cossi, J. M. Millam, M. Klene, C. Adamo, R. Cammi, J. W. Ochterski, R. L. Martin, K. Morokuma, O. Farkas, J. B. Foresman, and D. J. Fox, Gaussian, Inc., Wallingford CT, 2016.

Consider the hydrocupration of allene **15a** as shown in figure **S1** below. This allene can hypothetically undergo hydrocupration in to yield 4 unique regioisomers: Cu attaches on C1 with H on C2 ("Cu Branched"), Cu attaches on C2 with H on C1 ("Cu Vinyl Terminal"), Cu attaches on C2 with H on C3 ("Cu Vinyl Internal"), or Cu attaches on C3 with H on C2 ("Cu Terminal"). Based on our calculations, we found that the preferred pathway for this reaction is the Cu Branched Pathway ($\Delta G^{\ddagger}$ = 10.4 kcal/mol) as the other pathways were all significantly higher in energy (> 3 kcal/mol).

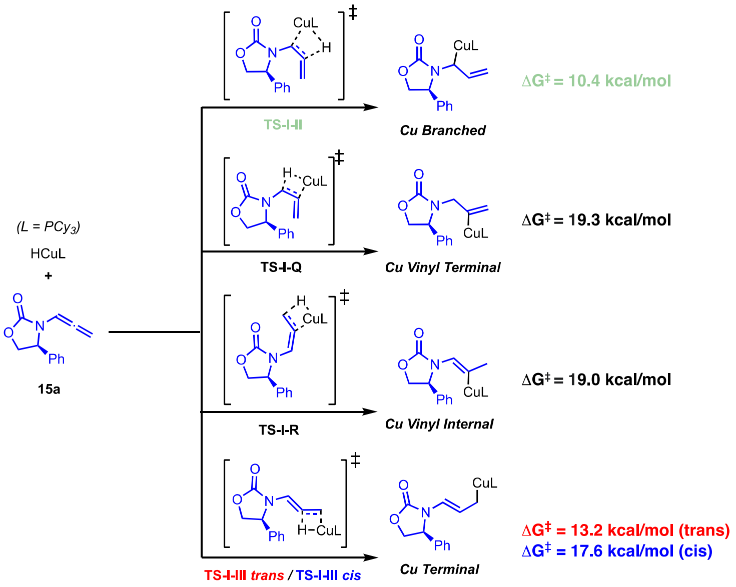

Supplementary Figure S1: Potential hydrocupration transition states for starting allene **15a**. Optimizations performed at B3LYP-D3/def2-SVP-CPCM(toluene) level of theory, $\Delta G^{\ddagger}$ reported in kcal/mol with respect to separate LCuH catalyst and reactants.

Figure **S2** below shows conformations of transition states located and their respective energies.

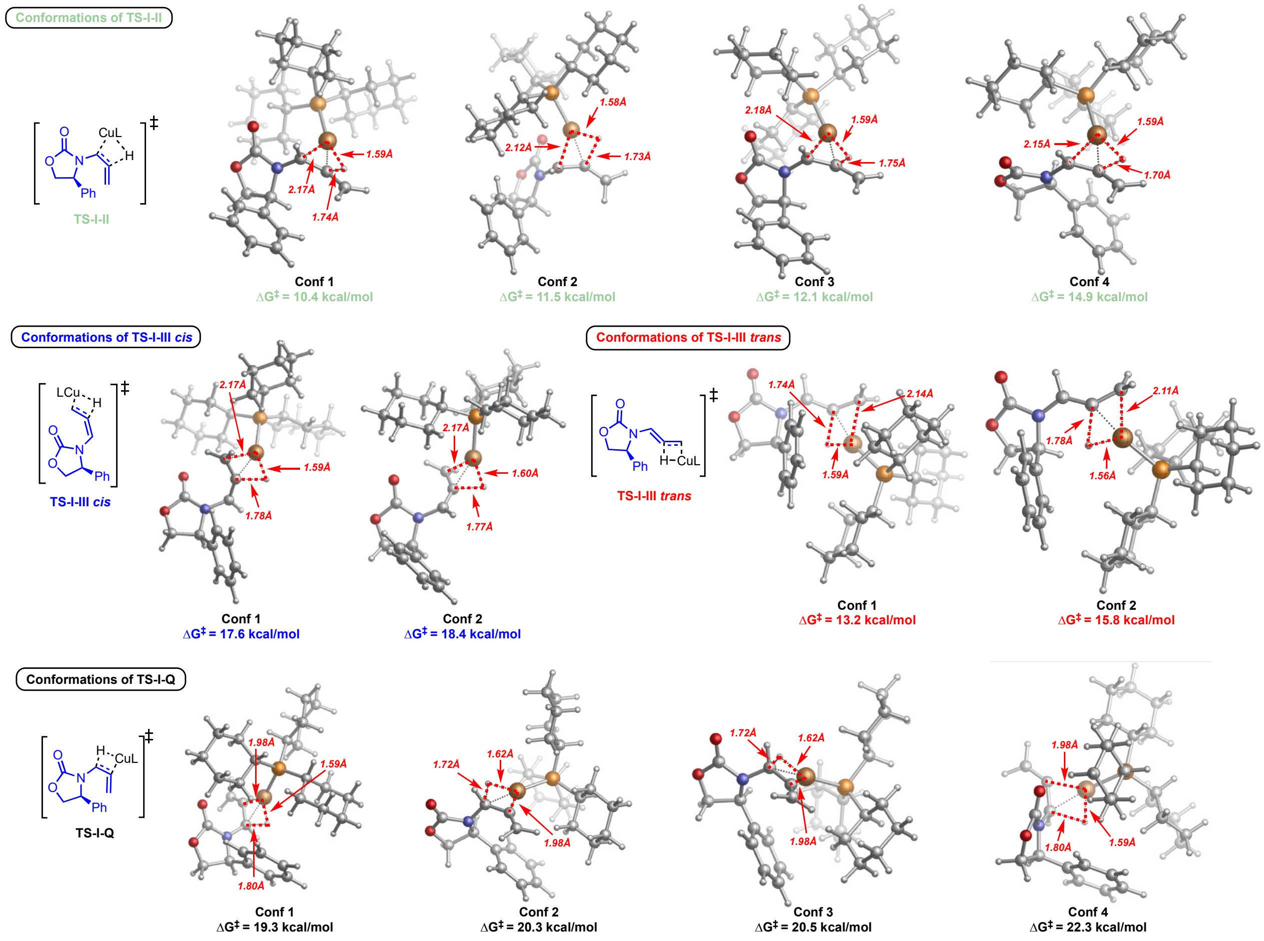

Supplementary Figure S2: Conformational search for hydrocupration transition state for starting allene **15a**. Optimizations performed at B3LYP-D3/def2-SVP-CPCM(toluene) level of theory, $\Delta G^{\ddagger}$ reported in kcal/mol with respect to separate LCuH catalyst and reactants.

Following the conformational search of the hydrocupration transition states, we sought to understand why the isomerization from branched allylcopper species **II** to **III *cis*** was favored over the same isomerization to **III *trans***. We recognized that there seemed to be coordination between the copper and the oxazolidinone in the *cis* transition state, as evidenced by the relatively short O-Cu distance (2.33Å) compared to the *trans* transition state (2.88Å) (see figure **S3**). We then optimized a transition state structure that would yield the *cis* isomer, but which did not have the coordination. As seen in figure **S3**, this coordination seems to stabilize the transition state by 2.7 kcal/mol (**TS-II-III *cis*** *vs.* **TS-II-III *cis* No Coordination**). As such, we propose that the *cis* isomer is benefitting from this coordination between the copper and oxazolidinone. This also provides justification for why the *cis* isomer **III' *cis*** becomes unfavorable once the imine is attached to the copper, as this coordination is no longer possible.

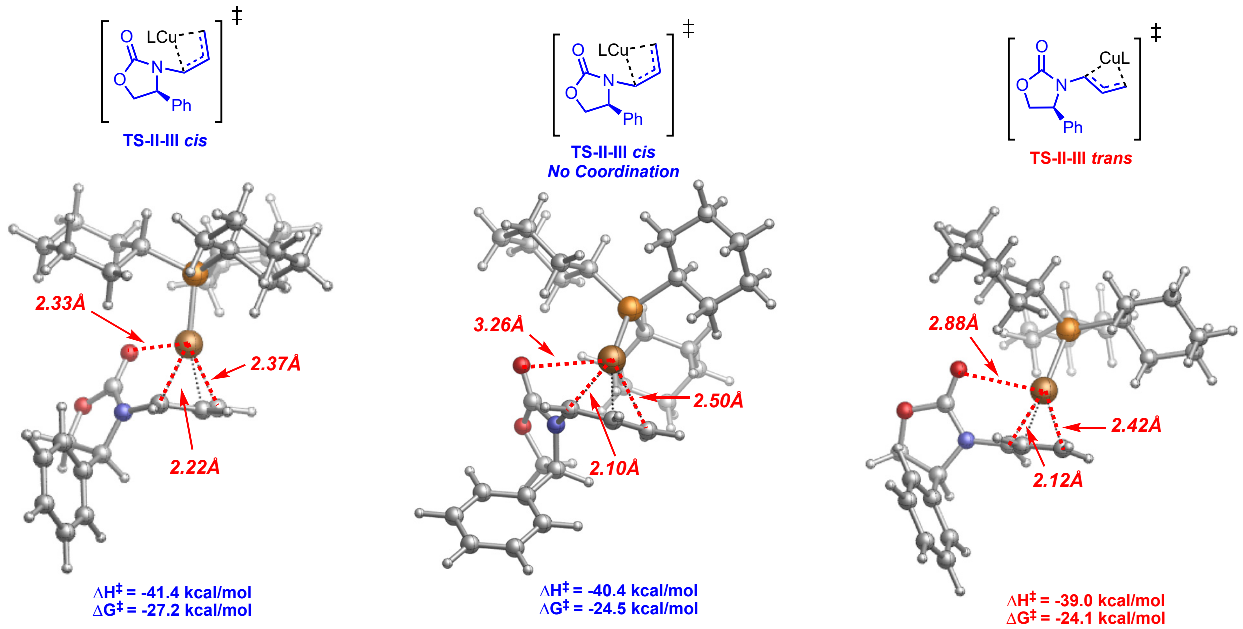

Supplementary Figure S3: Determination of the effect of coordination between copper and oxazolidinone on energies of isomerization transition states **TS-II-III *cis*** and **TS-II-III *trans***. Optimizations performed at B3LYP-D3/def2-SVP-CPCM(toluene) level of theory, $\Delta H^{\ddagger}/\Delta G^{\ddagger}$ reported in kcal/mol.

Prior to the isomerization from branched to linear allylcopper species (**TS-II-III**), it is possible for the branched allylcopper intermediate to coordinate to the imine and undergo C-C bond formation directly. To assess this possibility, we performed calculations on this pathway. As seen in figure **S5**, coordination of the imine to the branched allylcopper species (**II'**) is energetically unfavorable by 5.1 kcal/mol compared to imine coordination to the linear allylcopper species (**III' *trans***). In addition, the transition state from branched allylcopper species **II'** C-C bond formation (**TS-II'-X**) is 3.3 kcal/mol higher in energy than the lowest energy *(S,S,S)* branched C-C bond formation transition state **TS-III'-IV *trans (S,S,S)***. Based on these results, the pathway to form the linear product is unfavorable compared to the branched product, which aligns with the experimental observation that no linear product is formed.

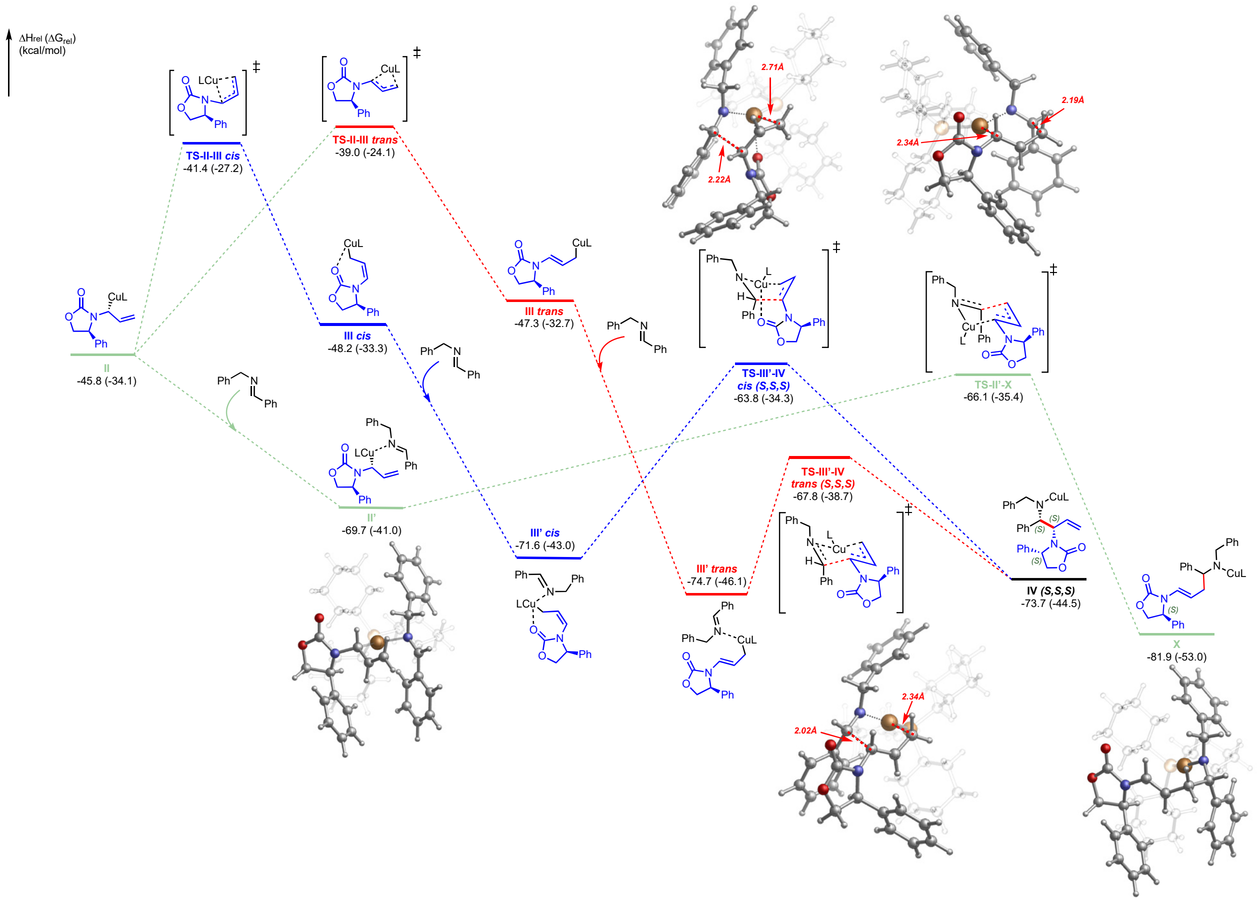

Supplementary Figure S4: Comparison of Linear vs. Branched C-C bond formation pathways. Optimizations performed at B3LYP-D3/def2-SVP-CPCM(toluene) level of theory, ΔH (ΔG; in parentheses) reported in kcal/mol with respect to separate LCuH catalyst and reactants.

Following the isomerization to linear allylcopper species, the C-C bond formation can proceed via numerous pathways. In order to find the lowest energy transition states for the desired stereochemical outcome (*(S,S,S)* stereochemistry), we performed an extensive conformational search. These conformations' geometries and their respective energies are shown in figure **S5** below.

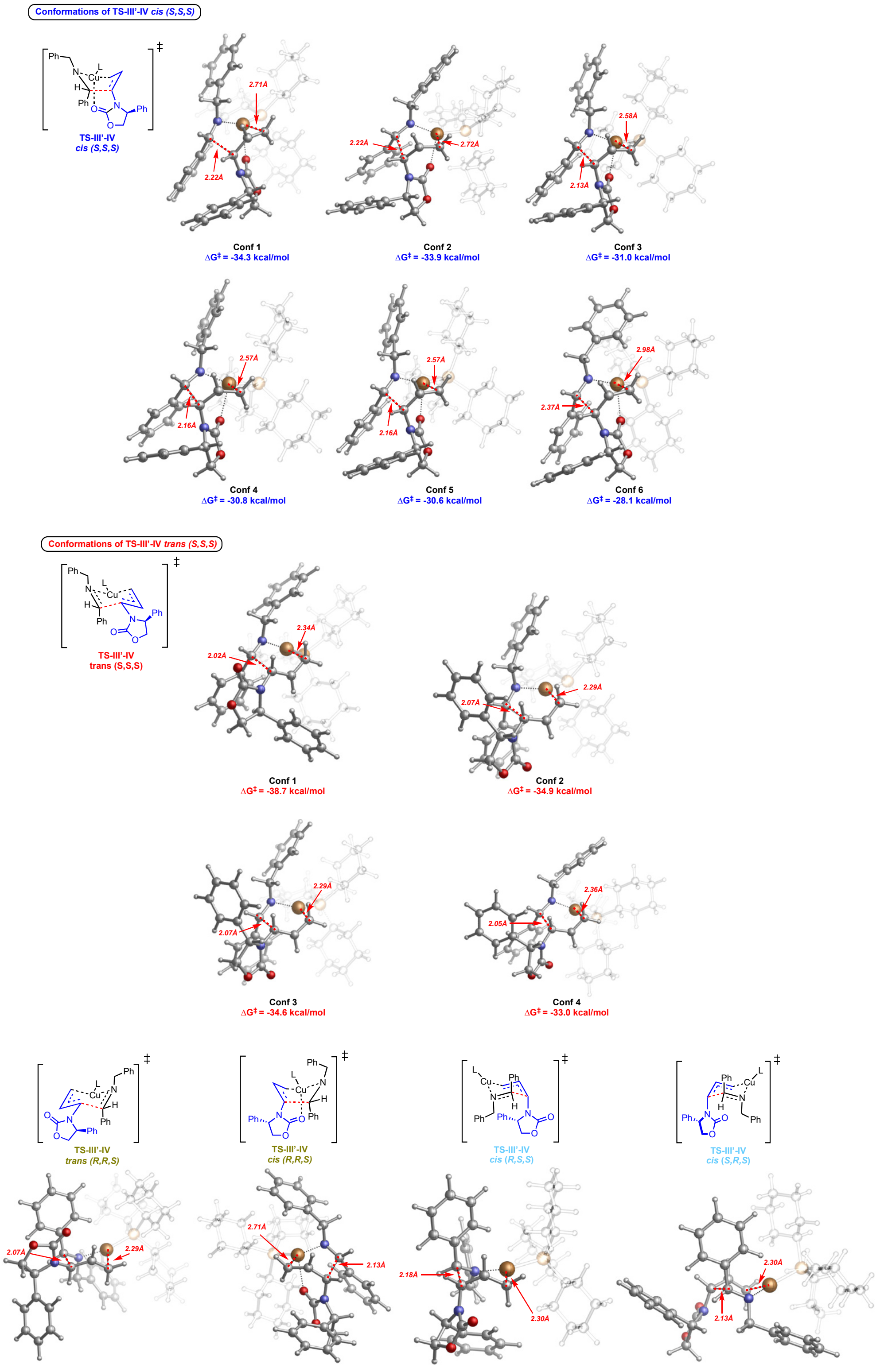

Supplementary Figure S5: Conformational search for Branched C-C bond formation transition states. Optimizations performed at B3LYP-D3/def2-SVP-CPCM(toluene) level of theory, ΔG‡ reported in kcal/mol with respect to separate LCuH catalyst and reactants.

Coordinates and Energies

I *cis*

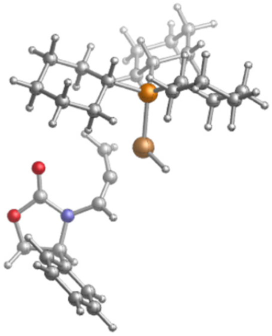

B3LYP-D3/def2-SVP-CPCM(toluene)

Zero-point correction= 0.697561 (Hartree/Particle)

Thermal correction to Energy= 0.733143

Thermal correction to Enthalpy= 0.734087

Thermal correction to Gibbs Free Energy= 0.626652

Sum of electronic and zero-point Energies= -3355.517076

Sum of electronic and thermal Energies= -3355.481494

Sum of electronic and thermal Enthalpies= -3355.480550

Sum of electronic and thermal Free Energies= -3355.587985

O	-2.00170300	-1.58853400	0.38978800
C	-0.93048800	-1.31579700	1.30150600
C	-1.61495000	-0.75508400	2.56861900
H	-0.37722400	-2.24578500	1.48650600
H	-0.25636100	-0.57113800	0.84836100
H	-1.04554600	0.10472800	2.96197600
N	-2.87386500	-0.28480900	1.98312100
C	-3.11846500	-0.90380000	0.76707300
O	-4.13437400	-0.89929200	0.11327000

C	-3.86446900	0.42593700	2.69780100
H	-4.13565900	0.03446300	3.68282300
C	-4.47708300	1.44778800	2.10277900
H	-6.03348400	1.87681400	4.01517100
C	-4.71054800	2.07137500	0.92465700
H	-4.45926400	3.13351400	0.78272400
H	-4.93311500	1.47106600	0.02737600
Cu	-6.38426500	1.97002500	2.31411000
P	-8.48086600	1.89350100	1.55983900
C	-8.52034400	0.44782700	0.35809900
C	-9.03872200	3.39428500	0.57825400
C	-9.86201600	1.56956100	2.79948300
C	-8.03966900	0.80429000	-1.06236600
C	-7.65224400	-0.71054800	0.90378700
H	-9.57382600	0.11243900	0.28363900
C	-9.83918500	4.45033500	1.36492800
C	-7.81438900	4.06641300	-0.07928800
H	-9.70194000	3.00764700	-0.21303200
C	-10.05116600	0.08928200	3.18262700
C	-9.61277100	2.40066300	4.07579000
H	-10.78835100	1.91113300	2.31722900
C	-8.07661000	-0.42079700	-1.98853200
H	-7.00330000	1.17693900	-0.99871900
H	-8.65003400	1.60894800	-1.49983000
C	-7.70004300	-1.94004100	-0.01132700
H	-6.60542900	-0.36556000	0.96405100

H	-7.95042600	-0.98761000	1.92407100
C	-10.26542600	5.61017600	0.45216100
H	-9.21262900	4.85120700	2.18116000
H	-10.73117800	4.00784100	1.83293600
C	-8.22553900	5.23116700	-0.98776000
H	-7.15223100	4.43352200	0.72614300
H	-7.21454000	3.33831700	-0.64064300
C	-11.19374800	-0.08038800	4.19483400
H	-9.11641100	-0.29125100	3.63125500
H	-10.25553900	-0.52688700	2.29428000
C	-10.74432200	2.23380000	5.09767800
H	-8.65455600	2.06690300	4.51297400
H	-9.47864900	3.46529600	3.83725100
C	-7.24935100	-1.58429200	-1.43148700
H	-7.71420600	-0.13314100	-2.98999000
H	-9.12686000	-0.74403600	-2.11575700
H	-7.05689700	-2.73179700	0.40724300
H	-8.73000500	-2.34360900	-0.03447100
C	-9.06107000	6.26804900	-0.22977100
H	-10.83168400	6.35329100	1.03836600
H	-10.95735500	5.22528800	-0.31957800
H	-7.32674600	5.70019100	-1.42181400
H	-8.81398300	4.83589500	-1.83642700
C	-10.96454200	0.76029400	5.45516900
H	-11.30162700	-1.14669400	4.45531400
H	-12.14454400	0.22476700	3.71990700

H	-10.51812800	2.82206200	6.00275100
H	-11.67762900	2.65156600	4.67653600
H	-7.32819200	-2.46207800	-2.09512300
H	-6.18401200	-1.29914100	-1.39679300
H	-9.39371500	7.07062900	-0.90917400
H	-8.42873200	6.74807000	0.53974600
H	-11.81359400	0.65247200	6.15083900
H	-10.07162200	0.38040000	5.98479300
C	-1.83315800	-1.76287200	3.68512200
C	-2.42622700	-3.00795100	3.41420700
C	-1.47369300	-1.45420000	5.00278700
C	-2.64933800	-3.92459500	4.44302800
H	-2.71435200	-3.26210300	2.39052300
C	-1.69665200	-2.37181300	6.03622100
H	-1.01779800	-0.48496200	5.22419700
C	-2.28481800	-3.60836300	5.75810500
H	-3.10993000	-4.89021700	4.21952300
H	-1.41238500	-2.11690800	7.06031300
H	-2.46107600	-4.32610100	6.56326100
O	-2.00170300	-1.58853400	0.38978800
C	-0.93048800	-1.31579700	1.30150600
C	-1.61495000	-0.75508400	2.56861900
H	-0.37722400	-2.24578500	1.48650600
H	-0.25636100	-0.57113800	0.84836100
H	-1.04554600	0.10472800	2.96197600
N	-2.87386500	-0.28480900	1.98312100

C	-3.11846500	-0.90380000	0.76707300
O	-4.13437400	-0.89929200	0.11327000
C	-3.86446900	0.42593700	2.69780100
H	-4.13565900	0.03446300	3.68282300
C	-4.47708300	1.44778800	2.10277900
H	-6.03348400	1.87681400	4.01517100
C	-4.71054800	2.07137500	0.92465700
H	-4.45926400	3.13351400	0.78272400
H	-4.93311500	1.47106600	0.02737600
Cu	-6.38426500	1.97002500	2.31411000
P	-8.48086600	1.89350100	1.55983900
C	-8.52034400	0.44782700	0.35809900
C	-9.03872200	3.39428500	0.57825400
C	-9.86201600	1.56956100	2.79948300
C	-8.03966900	0.80429000	-1.06236600
C	-7.65224400	-0.71054800	0.90378700
H	-9.57382600	0.11243900	0.28363900
C	-9.83918500	4.45033500	1.36492800
C	-7.81438900	4.06641300	-0.07928800
H	-9.70194000	3.00764700	-0.21303200
C	-10.05116600	0.08928200	3.18262700
C	-9.61277100	2.40066300	4.07579000
H	-10.78835100	1.91113300	2.31722900
C	-8.07661000	-0.42079700	-1.98853200
H	-7.00330000	1.17693900	-0.99871900
H	-8.65003400	1.60894800	-1.49983000

C	-7.70004300	-1.94004100	-0.01132700
H	-6.60542900	-0.36556000	0.96405100
H	-7.95042600	-0.98761000	1.92407100
C	-10.26542600	5.61017600	0.45216100
H	-9.21262900	4.85120700	2.18116000
H	-10.73117800	4.00784100	1.83293600
C	-8.22553900	5.23116700	-0.98776000
H	-7.15223100	4.43352200	0.72614300
H	-7.21454000	3.33831700	-0.64064300
C	-11.19374800	-0.08038800	4.19483400
H	-9.11641100	-0.29125100	3.63125500
H	-10.25553900	-0.52688700	2.29428000
C	-10.74432200	2.23380000	5.09767800
H	-8.65455600	2.06690300	4.51297400
H	-9.47864900	3.46529600	3.83725100
C	-7.24935100	-1.58429200	-1.43148700
H	-7.71420600	-0.13314100	-2.98999000
H	-9.12686000	-0.74403600	-2.11575700
H	-7.05689700	-2.73179700	0.40724300
H	-8.73000500	-2.34360900	-0.03447100
C	-9.06107000	6.26804900	-0.22977100
H	-10.83168400	6.35329100	1.03836600
H	-10.95735500	5.22528800	-0.31957800
H	-7.32674600	5.70019100	-1.42181400
H	-8.81398300	4.83589500	-1.83642700
C	-10.96454200	0.76029400	5.45516900

H	-11.30162700	-1.14669400	4.45531400
H	-12.14454400	0.22476700	3.71990700
H	-10.51812800	2.82206200	6.00275100
H	-11.67762900	2.65156600	4.67653600
H	-7.32819200	-2.46207800	-2.09512300
H	-6.18401200	-1.29914100	-1.39679300
H	-9.39371500	7.07062900	-0.90917400
H	-8.42873200	6.74807000	0.53974600
H	-11.81359400	0.65247200	6.15083900
H	-10.07162200	0.38040000	5.98479300
C	-1.83315800	-1.76287200	3.68512200
C	-2.42622700	-3.00795100	3.41420700
C	-1.47369300	-1.45420000	5.00278700
C	-2.64933800	-3.92459500	4.44302800
H	-2.71435200	-3.26210300	2.39052300
C	-1.69665200	-2.37181300	6.03622100
H	-1.01779800	-0.48496200	5.22419700
C	-2.28481800	-3.60836300	5.75810500
H	-3.10993000	-4.89021700	4.21952300
H	-1.41238500	-2.11690800	7.06031300
H	-2.46107600	-4.32610100	6.56326100

M06-L/def2-SVP-gas//B3LYP-D3/def2-SVP-CPCM(toluene)

HF = -3355.825245

B3LYP-D3/def2-TZVPP-gas//B3LYP-D3/def2-SVP-CPCM(toluene)

HF = -3358.07617

III *cis*

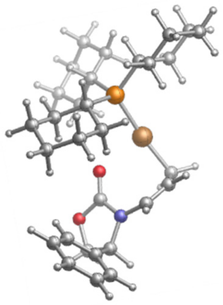

B3LYP-D3/def2-SVP-CPCM(toluene)

Zero-point correction= 0.703192 (Hartree/Particle)

Thermal correction to Energy= 0.738149

Thermal correction to Enthalpy= 0.739093

Thermal correction to Gibbs Free Energy= 0.634073

Sum of electronic and zero-point Energies= -3355.585243

Sum of electronic and thermal Energies= -3355.550286

Sum of electronic and thermal Enthalpies= -3355.549342

Sum of electronic and thermal Free Energies= -3355.654361

O	-2.00170300	-1.58853400	0.38978800
C	-0.93048800	-1.31579700	1.30150600
C	-1.61495000	-0.75508400	2.56861900
H	-0.37722400	-2.24578500	1.48650600
H	-0.25636100	-0.57113800	0.84836100
H	-1.05834600	0.10472800	2.96197600
N	-2.87386500	-0.29760900	1.97032100
C	-3.11846500	-0.90380000	0.76707300
O	-4.13437400	-0.89929200	0.11327000
C	-3.85166900	0.40033700	2.71060100

H	-4.07165900	0.00886300	3.70842300
C	-4.50268300	1.48618800	2.21797900
H	-5.02228400	1.88961400	3.28557100
C	-4.71054800	2.08417500	0.95025700
H	-4.56166400	3.15911400	0.80832400
H	-4.68991500	1.43266600	0.06577600
Cu	-6.39706500	1.97002500	2.30131000
P	-8.48086600	1.89350100	1.57263900
C	-8.52034400	0.44782700	0.35809900
C	-9.03872200	3.39428500	0.57825400
C	-9.86201600	1.56956100	2.79948300
C	-8.03966900	0.80429000	-1.06236600
C	-7.65224400	-0.71054800	0.90378700
H	-9.57382600	0.12523900	0.29643900
C	-9.83918500	4.45033500	1.36492800
C	-7.81438900	4.06641300	-0.07928800
H	-9.70194000	2.99484700	-0.21303200
C	-10.05116600	0.08928200	3.18262700
C	-9.61277100	2.40066300	4.07579000
H	-10.80115100	1.91113300	2.31722900
C	-8.07661000	-0.42079700	-1.98853200
H	-7.00330000	1.17693900	-0.99871900
H	-8.65003400	1.60894800	-1.49983000
C	-7.70004300	-1.94004100	-0.01132700
H	-6.60542900	-0.36556000	0.96405100
H	-7.95042600	-0.98761000	1.92407100

C	-10.26542600	5.61017600	0.45216100
H	-9.21262900	4.85120700	2.18116000
H	-10.73117800	4.00784100	1.83293600
C	-8.22553900	5.23116700	-0.98776000
H	-7.15223100	4.43352200	0.72614300
H	-7.21454000	3.33831700	-0.64064300
C	-11.19374800	-0.08038800	4.19483400
H	-9.11641100	-0.29125100	3.63125500
H	-10.25553900	-0.52688700	2.29428000
C	-10.74432200	2.23380000	5.09767800
H	-8.65455600	2.06690300	4.51297400
H	-9.47864900	3.46529600	3.83725100
C	-7.24935100	-1.58429200	-1.43148700
H	-7.71420600	-0.13314100	-2.98999000
H	-9.12686000	-0.74403600	-2.11575700
H	-7.05689700	-2.73179700	0.40724300
H	-8.73000500	-2.34360900	-0.03447100
C	-9.06107000	6.26804900	-0.22977100
H	-10.83168400	6.35329100	1.03836600
H	-10.95735500	5.22528800	-0.31957800
H	-7.32674600	5.70019100	-1.42181400
H	-8.81398300	4.83589500	-1.83642700
C	-10.96454200	0.76029400	5.45516900
H	-11.30162700	-1.14669400	4.45531400
H	-12.14454400	0.22476700	3.71990700
H	-10.51812800	2.82206200	6.00275100

H	-11.67762900	2.65156600	4.67653600
H	-7.32819200	-2.46207800	-2.09512300
H	-6.18401200	-1.29914100	-1.39679300
H	-9.39371500	7.07062900	-0.90917400
H	-8.42873200	6.74807000	0.53974600
H	-11.81359400	0.65247200	6.15083900
H	-10.07162200	0.38040000	5.98479300
C	-1.83315800	-1.76287200	3.68512200
C	-2.42622700	-3.00795100	3.41420700
C	-1.47369300	-1.45420000	5.00278700
C	-2.64933800	-3.92459500	4.44302800
H	-2.71435200	-3.26210300	2.39052300
C	-1.69665200	-2.37181300	6.03622100
H	-1.01779800	-0.48496200	5.22419700
C	-2.28481800	-3.60836300	5.75810500
H	-3.10993000	-4.89021700	4.21952300
H	-1.41238500	-2.11690800	7.06031300
H	-2.46107600	-4.32610100	6.56326100

M06-L/def2-SVP-gas//B3LYP-D3/def2-SVP-CPCM(toluene)

HF = -3355.893685

B3LYP-D3/def2-TZVPP-gas//B3LYP-D3/def2-SVP-CPCM(toluene)

HF = -3358.147305

TS-I-III *cis*

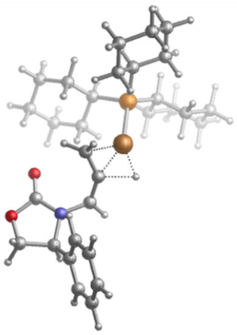

B3LYP-D3/def2-SVP-CPCM(toluene)

Zero-point correction= 0.695919 (Hartree/Particle)

Thermal correction to Energy= 0.731152

Thermal correction to Enthalpy= 0.732096

Thermal correction to Gibbs Free Energy= 0.626031

Sum of electronic and zero-point Energies= -3355.502786

Sum of electronic and thermal Energies= -3355.467553

Sum of electronic and thermal Enthalpies= -3355.466609

Sum of electronic and thermal Free Energies= -3355.572674

O	-2.00170300	-1.58853400	-0.38978800
C	-0.93048800	-1.31579700	-1.30150600
C	-1.61495000	-0.75508400	-2.56861900
H	-0.37722400	-2.24578500	-1.48650600
H	-0.25636100	-0.57113800	-0.84836100
N	-2.87386500	-0.29210900	-1.97582100
C	-3.11846500	-0.90380000	-0.76707300
O	-4.13437400	-0.89929200	-0.11327000
C	-3.85716900	0.41133700	-2.70510100
H	-4.09915900	0.01986300	-3.69742300
C	-4.49168300	1.46968800	-2.16847900
H	-5.45678400	1.88411400	-3.59907100

C	-4.71054800	2.07867500	-0.93925700
H	-4.51766400	3.14811400	-0.79732400
H	-4.79441500	1.44916600	-0.04927600
Cu	-6.39156500	1.97002500	-2.30681000
P	-8.48086600	1.89350100	-1.56713900
C	-8.52034400	0.44782700	-0.35809900
C	-9.03872200	3.39428500	-0.57825400
C	-9.86201600	1.56956100	-2.79948300
C	-8.03966900	0.80429000	1.06236600
C	-7.65224400	-0.71054800	-0.90378700
H	-9.57382600	0.11973900	-0.29093900
C	-9.83918500	4.45033500	-1.36492800
C	-7.81438900	4.06641300	0.07928800
H	-9.70194000	3.00034700	0.21303200
C	-10.05116600	0.08928200	-3.18262700
C	-9.61277100	2.40066300	-4.07579000
H	-10.79565100	1.91113300	-2.31722900
C	-8.07661000	-0.42079700	1.98853200
H	-7.00330000	1.17693900	0.99871900
H	-8.65003400	1.60894800	1.49983000
C	-7.70004300	-1.94004100	0.01132700
H	-6.60542900	-0.36556000	-0.96405100
H	-7.95042600	-0.98761000	-1.92407100
C	-10.26542600	5.61017600	-0.45216100
H	-9.21262900	4.85120700	-2.18116000
H	-10.73117800	4.00784100	-1.83293600
C	-8.22553900	5.23116700	0.98776000

H	-7.15223100	4.43352200	-0.72614300
H	-7.21454000	3.33831700	0.64064300
C	-11.19374800	-0.08038800	-4.19483400
H	-9.11641100	-0.29125100	-3.63125500
H	-10.25553900	-0.52688700	-2.29428000
C	-10.74432200	2.23380000	-5.09767800
H	-8.65455600	2.06690300	-4.51297400
H	-9.47864900	3.46529600	-3.83725100
C	-7.24935100	-1.58429200	1.43148700
H	-7.71420600	-0.13314100	2.98999000
H	-9.12686000	-0.74403600	2.11575700
H	-7.05689700	-2.73179700	-0.40724300
H	-8.73000500	-2.34360900	0.03447100
C	-9.06107000	6.26804900	0.22977100
H	-10.83168400	6.35329100	-1.03836600
H	-10.95735500	5.22528800	0.31957800
H	-7.32674600	5.70019100	1.42181400
H	-8.81398300	4.83589500	1.83642700
C	-10.96454200	0.76029400	-5.45516900
H	-11.30162700	-1.14669400	-4.45531400
H	-12.14454400	0.22476700	-3.71990700
H	-10.51812800	2.82206200	-6.00275100
H	-11.67762900	2.65156600	-4.67653600
H	-7.32819200	-2.46207800	2.09512300
H	-6.18401200	-1.29914100	1.39679300
H	-9.39371500	7.07062900	0.90917400
H	-8.42873200	6.74807000	-0.53974600

H	-11.81359400	0.65247200	-6.15083900
H	-10.07162200	0.38040000	-5.98479300
H	-1.76857589	-1.46460119	-3.35467526
C	-0.82799377	0.44866906	-3.11932624
C	-0.78764598	0.68620111	-4.49318894
C	-0.15492571	1.30134547	-2.24392221
C	-0.07445683	1.77666805	-4.99190181
H	-1.31784018	0.01399895	-5.18324777
C	0.55772035	2.39184315	-2.74240732
H	-0.18657510	1.11357732	-1.16077141
C	0.59764462	2.62974626	-4.11682528
H	-0.04347834	1.96410325	-6.07502238
H	1.08777438	3.06456825	-2.05263924
H	1.15928970	3.48964527	-4.50979476

M06-L/def2-SVP-gas//B3LYP-D3/def2-SVP-CPCM(toluene)

HF = -3355.809753

B3LYP-D3/def2-TZVPP-gas//B3LYP-D3/def2-SVP-CPCM(toluene)

HF = -3358.060558

TS-I-R

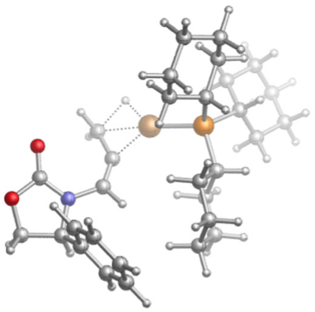

B3LYP-D3/def2-SVP-CPCM(toluene)

Zero-point correction= 0.696896 (Hartree/Particle)

Thermal correction to Energy= 0.732108

Thermal correction to Enthalpy= 0.733052

Thermal correction to Gibbs Free Energy= 0.626203

Sum of electronic and zero-point Energies= -3355.499742

Sum of electronic and thermal Energies= -3355.464530

Sum of electronic and thermal Enthalpies= -3355.463585

Sum of electronic and thermal Free Energies= -3355.570434

O	-3.53553800	6.61378500	-3.15011300
C	-3.70420500	5.24032600	-3.50450600
C	-4.67573800	4.66198300	-2.44633500
H	-4.10428600	5.18106500	-4.52582000
H	-2.72551600	4.73477200	-3.46759200
H	-4.30651100	3.69002900	-2.08370400
N	-4.53987500	5.67148200	-1.39724000
C	-3.97133600	6.83053500	-1.87182400
O	-3.86417600	7.89964800	-1.32447200
C	-5.27297000	5.55747900	-0.18020100
H	-6.12572500	4.88004800	-0.30233900
C	-5.02628700	6.16762800	0.97309900
C	-4.11319500	7.09194200	1.59214000
H	-3.15531700	6.71575800	1.97106400
H	-4.07223900	8.12450900	1.23084600
H	-4.58308900	7.64596300	3.25758700
Cu	-5.77179000	6.63912600	2.76641200
P	-7.81263200	6.15004300	3.47421300
C	-8.13824700	5.86519300	5.30341600

C	-8.48869000	4.63317300	2.58684200
C	-8.96516300	7.55759400	2.98013700
C	-7.97906700	4.41094300	5.78647900
C	-7.22543800	6.78878500	6.13698300
H	-9.19213300	6.15196400	5.47035200
C	-8.94560800	4.92982300	1.14372400
C	-7.40686300	3.53078300	2.56028000
H	-9.36383800	4.28127100	3.16335300
C	-9.20760800	8.62926700	4.06033700
C	-8.43115300	8.24212900	1.70180100
H	-9.93902400	7.08534700	2.75770600
C	-8.28185400	4.29167700	7.28776700
H	-6.94274000	4.07559300	5.60367600
H	-8.64049400	3.73343800	5.22588300
C	-7.51517300	6.67184600	7.63837000
H	-6.17676200	6.51002900	5.93024300
H	-7.31963400	7.83523700	5.81529000
C	-9.42863700	3.66288500	0.42264000
H	-8.09536500	5.35897300	0.58761100
H	-9.75022900	5.68005600	1.12965700
C	-7.89188200	2.26149100	1.84848200
H	-6.52575600	3.94303600	2.03676400
H	-7.06961000	3.27930400	3.57515900
C	-10.18461100	9.70350600	3.55809200
H	-8.24732100	9.10981200	4.31677400
H	-9.60154200	8.18314400	4.98570500
C	-9.39635800	9.31726800	1.18902100

H	-7.45817900	8.70774800	1.94615400
H	-8.22134900	7.51001400	0.91107900
C	-7.39556400	5.22276000	8.12179400
H	-8.15339600	3.24439800	7.60908600
H	-9.34382800	4.54564200	7.46179800
H	-6.82903600	7.32589100	8.20193200
H	-8.53779900	7.04141700	7.83971600
C	-8.36167600	2.56466900	0.42332300
H	-9.71361300	3.91694000	-0.61200900
H	-10.34085600	3.28675000	0.92202500
H	-7.08302400	1.51154200	1.83855800
H	-8.72562600	1.81630700	2.42292800
C	-9.68952400	10.36546900	2.26754600
H	-10.33845300	10.46009300	4.34574600
H	-11.16980900	9.23614800	3.37478300
H	-8.97640000	9.79495700	0.28827600
H	-10.34159200	8.83490000	0.87786200
H	-7.65605900	5.14738600	9.19083400
H	-6.34296400	4.89742400	8.02969100
H	-8.74673600	1.65319800	-0.06433100
H	-7.50145400	2.90022900	-0.18205200
H	-10.42683500	11.10091600	1.90444800
H	-8.76268200	10.92788900	2.48366000
C	-6.11406200	4.49274600	-2.91153500
C	-6.86962500	5.60854900	-3.30828800
C	-6.71581400	3.22865200	-2.91932900
C	-8.20098900	5.45917200	-3.70048200

H	-6.41596700	6.60313800	-3.29958800
C	-8.05066400	3.07556400	-3.31182700
H	-6.14038500	2.35523000	-2.59946600
C	-8.79678100	4.19104100	-3.70069000
H	-8.77946900	6.33583200	-4.00264900
H	-8.50971900	2.08390200	-3.30105700
H	-9.84126300	4.07589900	-4.00097100

M06-L/def2-SVP-gas//B3LYP-D3/def2-SVP-CPCM(toluene)

HF = -3355.808259

B3LYP-D3/def2-TZVPP-gas//B3LYP-D3/def2-SVP-CPCM(toluene)

HF = -3358.059313

TS-I-III *trans*

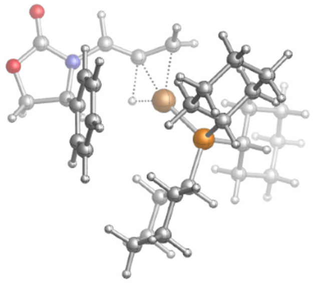

B3LYP-D3/def2-SVP-CPCM(toluene)

Zero-point correction= 0.696640 (Hartree/Particle)

Thermal correction to Energy= 0.731659

Thermal correction to Enthalpy= 0.732603

Thermal correction to Gibbs Free Energy= 0.628013

Sum of electronic and zero-point Energies= -3355.511101

Sum of electronic and thermal Energies= -3355.476083

Sum of electronic and thermal Enthalpies= -3355.475138

Sum of electronic and thermal Free Energies= -3355.579729

O	-2.78499300	-0.26404900	5.75948100
C	-3.26673100	1.05578500	5.49452200
C	-4.26656500	0.88110300	4.32944700
H	-3.73013500	1.45877600	6.40385400
H	-2.41870700	1.69441900	5.19588600
H	-4.18614100	1.72111400	3.62993600
N	-3.72102000	-0.33708400	3.73430800
C	-2.95106700	-1.03305100	4.65135900
O	-2.49548200	-2.14374000	4.53799300
C	-4.13170600	-0.96391200	2.54653900
H	-3.77116900	-1.99396800	2.48365400
C	-4.89950600	-0.47905000	1.56027400
H	-4.75094900	1.25326000	1.73754000
C	-5.75732700	-1.00611700	0.58571500
H	-5.57494100	-0.85818600	-0.48608000
H	-6.43194900	-1.82445000	0.85696300
Cu	-6.25320500	0.94857600	1.31386200
P	-8.08220200	2.05683700	0.72165000
C	-8.02915500	2.53510600	-1.09827100
C	-8.50263100	3.65594700	1.62265100
C	-9.58372000	0.94871300	0.93199400
C	-7.42495700	3.92654100	-1.37642600
C	-7.24586000	1.47547800	-1.90358500
H	-9.07976100	2.54818600	-1.44195700
C	-9.42117900	3.50808800	2.84942300
C	-7.19333900	4.35855500	2.03718000
H	-9.03552100	4.28691900	0.88942200

C	-9.78240800	-0.06342700	-0.21177800
C	-9.44816400	0.18625700	2.26800300
H	-10.47201400	1.60420900	0.96762500
C	-7.43586800	4.24604800	-2.87882000
H	-6.38226300	3.94675900	-1.01263400
H	-7.96785200	4.71483100	-0.83451500
C	-7.24978300	1.78480300	-3.40551000
H	-6.20547200	1.46271400	-1.53009100
H	-7.63808700	0.46636100	-1.72434500
C	-9.69507700	4.86944400	3.50500200
H	-8.93695700	2.85142000	3.58837700
H	-10.37583900	3.03485600	2.57526300
C	-7.45591100	5.71549000	2.70058000
H	-6.66069000	3.69086200	2.73704100
H	-6.52080800	4.48103100	1.17554800
C	-10.99305100	-0.97142300	0.05144100
H	-8.87294800	-0.68412500	-0.29437100
H	-9.91516300	0.45022600	-1.17619600
C	-10.65225300	-0.72095800	2.53963100
H	-8.53094200	-0.42776700	2.21368800
H	-9.29707400	0.87084500	3.11180700
C	-6.69137400	3.18219600	-3.69210600
H	-6.99330800	5.24208600	-3.04746400
H	-8.48385700	4.30499000	-3.22633400
H	-6.66941400	1.01733200	-3.94418200
H	-8.28634800	1.71872400	-3.78497100
C	-8.39607000	5.57864600	3.90282700

H	-10.34409500	4.72995500	4.38609300
H	-10.25751400	5.50773100	2.79870000
H	-6.49965000	6.17222700	3.00628100
H	-7.90835500	6.40068100	1.95991800
C	-10.87339700	-1.70877000	1.38975400
H	-11.10183800	-1.69089400	-0.77741300
H	-11.91166900	-0.35588800	0.05551500
H	-10.50134900	-1.25783500	3.49128300
H	-11.55689100	-0.09818800	2.66913700
H	-6.74859900	3.41019200	-4.76958400
H	-5.61988600	3.20481800	-3.42085700
H	-8.61470100	6.56782100	4.33865000
H	-7.89093800	4.99140500	4.69152500
H	-11.76986600	-2.32483500	1.57190400
H	-10.01530300	-2.40424500	1.34320600
C	-5.71747300	0.76760100	4.78103000
C	-6.39763200	-0.45467700	4.85923600
C	-6.37734500	1.93651700	5.19206100
C	-7.70918700	-0.50701600	5.34383900
H	-5.90517300	-1.37236600	4.53434400
C	-7.67908800	1.88356400	5.69522200
H	-5.86201100	2.89938900	5.12505800
C	-8.35224800	0.65878300	5.76872200
H	-8.22958500	-1.46684100	5.38980400
H	-8.17089300	2.80156600	6.02590200
H	-9.37570000	0.61534600	6.14907600

M06-L/def2-SVP-gas//B3LYP-D3/def2-SVP-CPCM(toluene)

HF = -3355.817728

B3LYP-D3/def2-TZVPP-gas//B3LYP-D3/def2-SVP-CPCM(toluene)

HF = -3358.068883

TS-I-Q

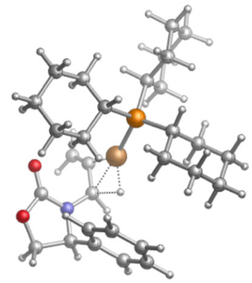

B3LYP-D3/def2-SVP-CPCM(toluene)

Zero-point correction= 0.696846 (Hartree/Particle)

Thermal correction to Energy= 0.731969

Thermal correction to Enthalpy= 0.732913

Thermal correction to Gibbs Free Energy= 0.627419

Sum of electronic and zero-point Energies= -3355.500561

Sum of electronic and thermal Energies= -3355.465438

Sum of electronic and thermal Enthalpies= -3355.464493

Sum of electronic and thermal Free Energies= -3355.569987

O	-1.23838900	1.23976200	0.56691100
C	-0.24991700	1.51710400	1.57063700
C	-0.97991100	1.26108600	2.90184200
H	0.61608900	0.86240900	1.40969000
H	0.05951800	2.57137000	1.48508200
H	-0.64871100	1.97314100	3.67234500
N	-2.34272000	1.59293700	2.46832400
C	-2.47733400	1.33632700	1.13018600

O	-3.49590800	1.17403400	0.49448500
C	-3.49396900	1.52322900	3.38738400
H	-3.08519400	1.51566600	4.41248000
C	-4.52474400	2.32626800	3.22363600
C	-4.76158000	3.58663800	2.86874600
H	-3.94347400	4.31993000	2.74871600
H	-5.77188300	3.98265100	2.70524400
C	-0.86090600	-0.15883400	3.44291700
C	-0.88809600	-1.26669700	2.57829800
C	-0.79884200	-0.38053100	4.82470700
C	-0.86789900	-2.56492700	3.08912400
H	-0.95489600	-1.11389500	1.49877400
C	-0.78568900	-1.68167100	5.33939800
H	-0.77415400	0.47340000	5.50785100
C	-0.82237700	-2.77706000	4.47231100
H	-0.90195300	-3.41607200	2.40461800
H	-0.74969700	-1.83906000	6.42026400
H	-0.82056400	-3.79412600	4.87195500
P	-6.12297200	-1.58111100	2.55824000
C	-5.86495200	-2.83634800	3.93648200
C	-5.55624200	-2.20960700	0.87621200
C	-7.99413200	-1.38266000	2.35058100
C	-4.51890100	-3.57687300	3.86326200
C	-5.97426700	-2.08017400	5.27946700
H	-6.67020000	-3.58594600	3.87716800
C	-5.96539400	-1.14769100	-0.17761200
C	-4.02360000	-2.38774500	0.81102100

H	-6.05600000	-3.17141900	0.65596200
C	-8.91754100	-2.41671600	3.01528600
C	-8.40350500	0.05237800	2.73779700
H	-8.14771500	-1.47886200	1.26496400
C	-4.28815400	-4.47963100	5.08346500
H	-3.70401400	-2.83768500	3.80041200
H	-4.47434200	-4.19089300	2.95219300
C	-5.75691600	-2.99808900	6.48757100
H	-5.20399800	-1.28670000	5.28566500
H	-6.94487600	-1.57039100	5.36185800
C	-5.48082800	-1.50768700	-1.58857400
H	-5.51603200	-0.18395100	0.12038900
H	-7.05527400	-1.00019600	-0.20056600
C	-3.53607200	-2.75132000	-0.59681500
H	-3.55964500	-1.43856600	1.12137700
H	-3.67621200	-3.14552300	1.51971700
C	-10.38556700	-2.13928200	2.65534100
H	-8.81667800	-2.35844800	4.11214000
H	-8.64063400	-3.44153200	2.71771400
C	-9.88037800	0.32491000	2.42725000
H	-8.20790700	0.20768400	3.81500300
H	-7.75409600	0.77252900	2.21375400
C	-4.40195800	-3.70671200	6.40046400
H	-3.29722100	-4.95692400	4.99768300
H	-5.03195000	-5.29797700	5.07934900
H	-5.83278400	-2.40931100	7.41700300
H	-6.56356000	-3.75338600	6.52583600

C	-3.96314000	-1.70525100	-1.62874800
H	-5.78309500	-0.71082400	-2.28872900
H	-5.98645700	-2.43219600	-1.92535800
H	-2.43739800	-2.85578000	-0.58216000
H	-3.94202200	-3.74042900	-0.88125000
C	-10.79758600	-0.72209600	3.06959000
H	-11.03686100	-2.89000900	3.13365200
H	-10.51981300	-2.25454100	1.56389300
H	-10.15395300	1.33935500	2.76227800
H	-10.02366700	0.30742900	1.33088200
H	-4.26302700	-4.38534700	7.25881200
H	-3.59320700	-2.95472700	6.45179800
H	-3.63714300	-2.00021800	-2.64056900
H	-3.47389500	-0.74504300	-1.39137800
H	-11.85085200	-0.53146100	2.80396300
H	-10.72990900	-0.63620100	4.17012200
Cu	-4.84449500	0.19444100	2.97468000
H	-3.43224500	-0.12029700	3.50702700

TS-I-II

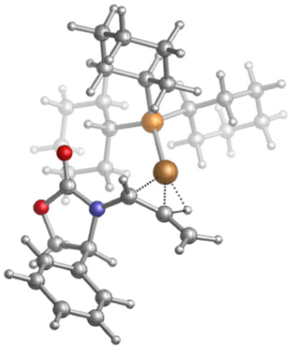

B3LYP-D3/def2-SVP-CPCM(toluene)

Zero-point correction= 0.696398 (Hartree/Particle)

Thermal correction to Energy= 0.731661

Thermal correction to Enthalpy= 0.732605

Thermal correction to Gibbs Free Energy= 0.627398

Sum of electronic and zero-point Energies= -3355.515154

Sum of electronic and thermal Energies= -3355.479892

Sum of electronic and thermal Enthalpies= -3355.478948

Sum of electronic and thermal Free Energies= -3355.584155

O	-2.24777982	-1.74149618	2.30763239
C	-0.96295259	-1.11390921	2.18934657
C	-1.26889561	0.39266057	2.01585688
H	-0.39088719	-1.29720796	3.11339781
H	-0.42659724	-1.55044325	1.33671797
H	-1.30223347	0.66576687	0.94504781
N	-2.62101500	0.44000900	2.57326400
C	-3.17201525	-0.80296051	2.63006532
O	-4.32640360	-1.08523077	2.90391292
C	-3.40316900	1.64508500	2.63463600
H	-4.11296900	1.68884800	3.46496200
C	-2.92218400	2.83286800	2.03269800
C	-2.25050200	3.93111600	2.37608500
H	-1.82204500	4.00560000	3.38329700
H	-2.10794000	4.77555500	1.69653200
C	-0.28905322	1.29917872	2.72992298
C	-0.30299499	1.39233335	4.13072409
C	0.66051690	2.03427243	2.00983343
C	0.61592205	2.20446078	4.79784066

H	-1.05312260	0.83457564	4.69551596
C	1.58782430	2.84371457	2.67625096
H	0.67216051	1.97763459	0.91765300
C	1.56730538	2.93120535	4.07126407
H	0.58516462	2.27500621	5.88801344
H	2.32340919	3.41267998	2.10200173
H	2.28648525	3.56826568	4.59220771
P	-6.00239100	1.14380200	-0.21374800
C	-6.58680441	1.89931296	-1.83065086
C	-7.48254115	1.31786627	0.92994399
C	-5.77720572	-0.70072142	-0.34860374
C	-6.15153045	3.38223599	-1.87366294
C	-6.07856288	1.18488242	-3.09703767
H	-7.69108737	1.84729885	-1.82577379
C	-7.18450211	0.63975046	2.28309853
C	-7.83717136	2.80364909	1.13707269
H	-8.34251922	0.81548137	0.45104419
C	-6.98380737	-1.53188840	-0.81315255
C	-4.48172202	-1.10056991	-1.08134375
H	-5.58608689	-0.93874990	0.71116088
C	-6.64219765	4.08780233	-3.14349624
H	-5.04790670	3.41152125	-1.82796252
H	-6.50034714	3.92385115	-0.98238779
C	-6.57409785	1.88341731	-4.37169554
H	-4.97420427	1.18436397	-3.09123481
H	-6.39701351	0.13306222	-3.10802863
C	-8.30045355	0.85968604	3.31275662

H	-6.23593451	1.05377784	2.66590067
H	-7.02374744	-0.44063427	2.14752578
C	-8.95932722	2.99911720	2.16482858
H	-6.92782988	3.33088453	1.47976245
H	-8.13579729	3.26364101	0.18302507
C	-6.69639217	-3.03371610	-0.65805742
H	-7.20956208	-1.31257862	-1.87095354
H	-7.88637524	-1.26677111	-0.23936493
C	-4.21136299	-2.60018680	-0.90362828
H	-4.56082903	-0.87683994	-2.15698168
H	-3.64473359	-0.50679071	-0.68396648
C	-6.16773601	3.36041072	-4.40587957
H	-6.29430691	5.13445037	-3.14602074
H	-7.74748851	4.12392194	-3.13513390
H	-6.18628717	1.35489298	-5.25880935
H	-7.67632908	1.80740247	-4.41782330
C	-8.61177434	2.34669266	3.50503908
H	-8.01355825	0.39542610	4.27200952
H	-9.21602860	0.33864607	2.97718852
H	-9.15676158	4.07633909	2.29593918
H	-9.89264235	2.55346665	1.77352932
C	-5.40343293	-3.44337031	-1.37451586
H	-7.55061409	-3.62182447	-1.03431283
H	-6.60218746	-3.26808291	0.41832445
H	-3.29781107	-2.88565097	-1.45188745
H	-4.01423708	-2.80123488	0.16491367
H	-6.56765363	3.85003954	-5.30972299

H	-5.06626333	3.43034575	-4.46938055
H	-9.43449817	2.47964311	4.22776377
H	-7.72883055	2.85255768	3.93464971
H	-5.20395820	-4.51678548	-1.21682236
H	-5.53540629	-3.30550202	-2.46414570
Cu	-4.19067200	2.05452900	0.70704600
H	-3.21698800	3.25018100	0.38071300

M06-L/def2-SVP-gas//B3LYP-D3/def2-SVP-CPCM(toluene)

HF = -3355.819825

B3LYP-D3/def2-TZVPP-gas//B3LYP-D3/def2-SVP-CPCM(toluene)

HF = -3358.071773

I *trans*

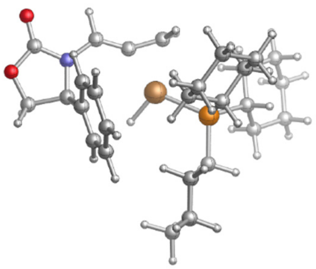

B3LYP-D3/def2-SVP-CPCM(toluene)

Zero-point correction= 0.698408 (Hartree/Particle)

Thermal correction to Energy= 0.733631

Thermal correction to Enthalpy= 0.734576

Thermal correction to Gibbs Free Energy= 0.630996

Sum of electronic and zero-point Energies= -3355.529607

Sum of electronic and thermal Energies= -3355.494384

Sum of electronic and thermal Enthalpies= -3355.493440

Sum of electronic and thermal Free Energies= -3355.597019

O	4.08662500	-1.99792700	3.89502900
C	3.53761700	-0.67534600	3.80721300
C	2.57149800	-0.72502700	2.60316200
H	3.03449800	-0.43676100	4.75269400
H	4.35901800	0.03925800	3.63442400
H	2.63247000	0.18525900	1.99172900
N	3.15369600	-1.84974900	1.87864200
C	3.94854200	-2.62863600	2.70314500
O	4.44391800	-3.69815100	2.44884700
C	2.78738000	-2.29736100	0.60717100
H	3.19193500	-3.29389900	0.39690800
C	2.03732700	-1.68174800	-0.30205300
H	1.61231600	1.05367100	0.55193300
C	1.30307600	-1.78152300	-1.46022200
H	1.72260100	-1.49746700	-2.43440300
H	0.39414700	-2.39448400	-1.50087900
Cu	1.01332700	0.03193900	-0.45578100
P	-0.90545900	0.92801900	-1.38772500
C	-1.00967600	1.28983300	-3.23356300
C	-1.44406400	2.52377500	-0.54548900
C	-2.33632500	-0.25948700	-1.06257700
C	-0.60241300	2.70995300	-3.67082100
C	-0.17746300	0.25259400	-4.01536600
H	-2.07519800	1.16508500	-3.49765600
C	-2.06449000	2.29621800	0.84757500
C	-0.24888300	3.49206300	-0.41398300
H	-2.21667300	2.97475900	-1.19601600

C	-2.63859000	-1.26713000	-2.18879100
C	-2.08212300	-1.02958300	0.25209900
H	-3.23371400	0.37425400	-0.94042800
C	-0.76580500	2.88726300	-5.18820500
H	0.45294600	2.89049900	-3.40066100
H	-1.20281300	3.47052700	-3.15029800
C	-0.33099800	0.42427700	-5.53159800
H	0.88275900	0.38185500	-3.73334300
H	-0.44543300	-0.77234200	-3.72428700
C	-2.46609400	3.61830900	1.51619300
H	-1.32650300	1.77974000	1.48377700
H	-2.94789300	1.64398100	0.78778400
C	-0.65225900	4.81693200	0.24515200
H	0.52245400	2.98840100	0.19242600
H	0.21532700	3.69047500	-1.38932100
C	-3.83447900	-2.15965400	-1.82302600
H	-1.75429200	-1.90612100	-2.35486600
H	-2.84536100	-0.75490100	-3.14022700
C	-3.27008100	-1.92165800	0.62735900
H	-1.18453800	-1.65826700	0.12076500
H	-1.84508900	-0.35203200	1.08148000
C	0.03617700	1.84451600	-5.97380000
H	-0.46034100	3.90723700	-5.47580100
H	-1.83667200	2.79533700	-5.44771200
H	0.29250300	-0.31871300	-6.05641200
H	-1.37855600	0.21154700	-5.81448100
C	-1.28342900	4.58528100	1.62116900

H	-2.88827700	3.41125200	2.51435800
H	-3.27287800	4.09175600	0.92644100
H	0.23123100	5.47175700	0.33014400
H	-1.37469000	5.34685600	-0.40362900
C	-3.60966900	-2.90093400	-0.50059000
H	-4.02530900	-2.87534500	-2.64031700
H	-4.73956000	-1.53014700	-1.73913900
H	-3.04122300	-2.46289000	1.55920100
H	-4.14880700	-1.28460000	0.83988900
H	-0.12758500	1.96802900	-7.05737200
H	1.11492800	2.01004000	-5.79697600
H	-1.60305100	5.54054100	2.07058900
H	-0.52331600	4.15432200	2.29868600
H	-4.49679100	-3.50256600	-0.24084400
H	-2.77154000	-3.61165100	-0.62347500
C	1.11499700	-0.92804900	2.99223300
C	0.54093900	-2.19941900	3.11887100
C	0.33452700	0.20073300	3.28532200
C	-0.78446600	-2.34087100	3.54441600
H	1.12703900	-3.08836600	2.87558400
C	-0.98546900	0.06155600	3.72069000
H	0.76442800	1.19722500	3.15811100
C	-1.54994300	-1.21211700	3.85161700
H	-1.22026900	-3.33917100	3.63431100
H	-1.58054100	0.95065700	3.94419500
H	-2.58614900	-1.32335500	4.18026000

M06-L/def2-SVP-gas//B3LYP-D3/def2-SVP-CPCM(toluene)

HF = -3355.839431

B3LYP-D3/def2-TZVPP-gas//B3LYP-D3/def2-SVP-CPCM(toluene)

HF = -3358.09042

III *trans*

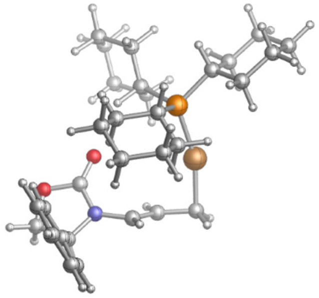

B3LYP-D3/def2-SVP-CPCM(toluene)

Zero-point correction= 0.702950 (Hartree/Particle)

Thermal correction to Energy= 0.738133

Thermal correction to Enthalpy= 0.739078

Thermal correction to Gibbs Free Energy= 0.634159

Sum of electronic and zero-point Energies= -3355.584027

Sum of electronic and thermal Energies= -3355.548843

Sum of electronic and thermal Enthalpies= -3355.547899

Sum of electronic and thermal Free Energies= -3355.652817

O	-1.47004100	-1.54348400	2.38416800
C	-0.36830200	-0.98925700	3.11250100
C	-0.52700100	0.53841200	2.96638500
H	-0.41965700	-1.33444900	4.15340500
H	0.57284900	-1.33908200	2.65741700
H	0.44561600	1.01757200	2.77914700
N	-1.33858900	0.58651300	1.74574300
C	-1.95224900	-0.61119000	1.50646100

O	-2.79684600	-0.88114700	0.68039900
C	-1.57948000	1.78988400	1.03163900
H	-1.92980300	2.63137700	1.63974200
C	-1.49152700	1.85454000	-0.32297700
C	-1.91583500	2.93762500	-1.20984800
H	-1.94336200	3.92835400	-0.72443700
H	-1.34391700	2.98023800	-2.14815700
C	-1.20417700	1.22606200	4.14193000
C	-2.35332300	0.67193800	4.73081700
C	-0.70536800	2.43713000	4.63873900
C	-2.97766100	1.31015300	5.80424000
H	-2.76570200	-0.26344000	4.34318500
C	-1.33561000	3.08367900	5.70828200
H	0.18461200	2.88010000	4.18290700
C	-2.47118700	2.51925900	6.29567400
H	-3.86910400	0.86719300	6.25454400
H	-0.93636700	4.02956000	6.08317200
H	-2.96362800	3.01999500	7.13297900
P	-5.70587100	1.27139600	-0.80766000
C	-7.20127400	1.75437500	-1.81529400
C	-6.14207900	1.76496800	0.95820800
C	-5.59608300	-0.58649600	-0.82852000
C	-7.50179000	3.26011200	-1.67961600
C	-6.98173200	1.37857100	-3.29433000
H	-8.06455100	1.18703000	-1.42361400
C	-5.65475000	0.76548100	2.02595100
C	-5.55102700	3.16305300	1.25121200

H	-7.24462300	1.82124300	1.01357800
C	-6.92962900	-1.34549900	-0.75534300
C	-4.70081200	-1.10587000	-1.97151800
H	-5.02366200	-0.78972400	0.08810100
C	-8.66556300	3.69833700	-2.57889900
H	-6.59154500	3.82627300	-1.95167800
H	-7.73331300	3.51507500	-0.63419800
C	-8.14620300	1.82833600	-4.18609000
H	-6.04404200	1.85353900	-3.63811600
H	-6.83697200	0.29316000	-3.39822500
C	-5.97430700	1.25366800	3.44651200
H	-4.56567100	0.62463700	1.91852100
H	-6.11376000	-0.22283600	1.87308900
C	-5.90506900	3.65045800	2.65993100
H	-4.45140600	3.09312000	1.15835400
H	-5.87393300	3.89582200	0.49698500
C	-6.68280700	-2.85507900	-0.61301800
H	-7.51709800	-1.16254100	-1.67239700
H	-7.54137700	-0.97920900	0.08646100
C	-4.46458500	-2.61381400	-1.81826800
H	-5.16390600	-0.90273400	-2.95252800
H	-3.74110100	-0.56502400	-1.94951200
C	-8.41833700	3.32895700	-4.04473600
H	-8.82654000	4.78427500	-2.47320600
H	-9.59378500	3.20763000	-2.23226600
H	-7.93092300	1.56894000	-5.23614700
H	-9.05510100	1.26582200	-3.90325700

C	-5.41687600	2.65449000	3.71474400
H	-5.57060600	0.53463600	4.17927600
H	-7.07115400	1.26383700	3.58734100
H	-5.46220700	4.64579000	2.83233600
H	-7.00116500	3.77360500	2.74090300
C	-5.78786100	-3.38637200	-1.74023500
H	-7.64454100	-3.39538400	-0.59514800
H	-6.19345200	-3.04672100	0.35930300
H	-3.85260700	-2.98612800	-2.65730600
H	-3.87990300	-2.77747000	-0.89684400
H	-9.27743500	3.62706200	-4.66870800
H	-7.54436300	3.89339400	-4.41906800
H	-5.68980700	2.99032100	4.72932500
H	-4.31536900	2.61305400	3.68617800
H	-5.59647400	-4.46346600	-1.59861100
H	-6.32452900	-3.28715000	-2.70270000
Cu	-3.76642400	2.22776600	-1.34917400
H	-1.14622300	0.93658400	-0.81880300

M06-L/def2-SVP-gas//B3LYP-D3/def2-SVP-CPCM(toluene)

HF = -3355.889435

B3LYP-D3/def2-TZVPP-gas//B3LYP-D3/def2-SVP-CPCM(toluene)

HF = -3358.147684

I int

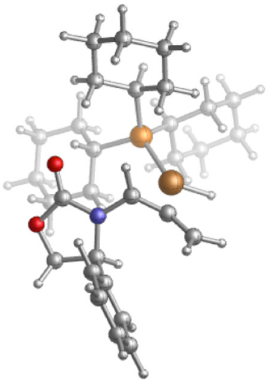

B3LYP-D3/def2-SVP-CPCM(toluene)

Zero-point correction= 0.697749 (Hartree/Particle)

Thermal correction to Energy= 0.733406

Thermal correction to Enthalpy= 0.734350

Thermal correction to Gibbs Free Energy= 0.628858

Sum of electronic and zero-point Energies= -3355.535315

Sum of electronic and thermal Energies= -3355.499658

Sum of electronic and thermal Enthalpies= -3355.498713

Sum of electronic and thermal Free Energies= -3355.604206

O	1.97553400	-2.39019900	1.63782300
C	3.23930800	-2.17899500	1.00079900
C	3.43071800	-0.65067100	1.00998300
H	4.01684900	-2.72205300	1.55355600
H	3.18684600	-2.56496200	-0.03120000
H	3.87943000	-0.32431600	0.06135200
N	2.02583900	-0.22923200	1.07341900
C	1.24121300	-1.23163200	1.55319700
O	0.07202600	-1.19794100	1.88135400
C	1.56258000	1.15423200	0.94600800
H	1.30694400	1.57764300	1.93181800

C	2.39148800	2.04188100	0.12794700
C	2.51657200	3.38750700	0.24846700
H	2.04079500	3.93105100	1.07184600
H	3.07018000	3.97292500	-0.48908300
C	4.24921500	-0.08815100	2.15750500
C	4.10735900	-0.59350400	3.46012700
C	5.11959100	0.98631700	1.93632300
C	4.83721300	-0.04249800	4.51564900
H	3.41947700	-1.42193200	3.64959600
C	5.84725900	1.54352900	2.99228400
H	5.21700700	1.40199500	0.93063900
C	5.71044000	1.02741800	4.28395900
H	4.72349200	-0.44770100	5.52447600
H	6.52045700	2.38405200	2.80491300
H	6.28004900	1.45887500	5.11109300
P	-1.73939000	0.09498000	-1.09976300
C	-2.51006300	0.47638400	-2.74972100
C	-3.08686300	0.45078300	0.17008100
C	-1.37458900	-1.73359200	-1.03257700
C	-2.93603500	1.95782600	-2.79161000
C	-1.51746300	0.16221100	-3.88632400
H	-3.40722200	-0.15805700	-2.86649300
C	-3.20871200	-0.60228300	1.28879400
C	-2.81935700	1.83963000	0.79796300
H	-4.04373900	0.48613800	-0.38203600
C	-2.44004900	-2.66007500	-1.63801000
C	0.03271600	-2.08349700	-1.56345200

H	-1.32790300	-1.90864500	0.05350600
C	-3.46585900	2.36922200	-4.17056800
H	-2.05935700	2.58139100	-2.53483800
H	-3.70161800	2.16131600	-2.02680200
C	-2.05501400	0.58634300	-5.25873000
H	-0.56907200	0.69301200	-3.68141100
H	-1.27783300	-0.91119000	-3.90099100
C	-4.29058700	-0.20865900	2.30571700
H	-2.23643300	-0.69904300	1.79776200
H	-3.44978100	-1.59078300	0.86917600
C	-3.90078100	2.23467600	1.80840800
H	-1.84423800	1.79673800	1.31685200
H	-2.71846300	2.61076300	0.01813700
C	-2.09984800	-4.13059400	-1.35040100
H	-2.48987600	-2.50688900	-2.73063100
H	-3.44072800	-2.41813500	-1.24375600
C	0.36884700	-3.55094900	-1.26581300
H	0.08601900	-1.91222500	-2.65184900
H	0.78222200	-1.41616200	-1.10857500
C	-2.45041500	2.06596800	-5.27668300
H	-3.72466600	3.44120200	-4.16326600
H	-4.40316600	1.82052200	-4.37692200
H	-1.29948300	0.37581900	-6.03404000
H	-2.93933800	-0.02931600	-5.50610300
C	-4.03535600	1.17691700	2.90874900
H	-4.34151800	-0.97097900	3.10115500
H	-5.27736500	-0.20988400	1.80586400

H	-3.66225300	3.22001300	2.24275500
H	-4.86821500	2.34657400	1.28431000
C	-0.69367000	-4.49373300	-1.84575300
H	-2.85327400	-4.78988500	-1.81348000
H	-2.15638300	-4.30222300	-0.26010900
H	1.36396600	-3.79757600	-1.67399800
H	0.43635500	-3.68531400	-0.17205600
H	-2.85815800	2.34507200	-6.26252000
H	-1.54774000	2.68534400	-5.12101300
H	-4.84264900	1.44740400	3.60993900
H	-3.10002900	1.14824500	3.49703400
H	-0.45863800	-5.54090900	-1.59179400
H	-0.67244300	-4.42581600	-2.94961900
Cu	0.06627400	1.11955600	-0.36973700
H	2.90184400	1.56614900	-0.72233000

M06-L/def2-SVP-gas//B3LYP-D3/def2-SVP-CPCM(toluene)

HF = -3355.839234

B3LYP-D3/def2-TZVPP-gas//B3LYP-D3/def2-SVP-CPCM(toluene)

HF = -3358.092678

II

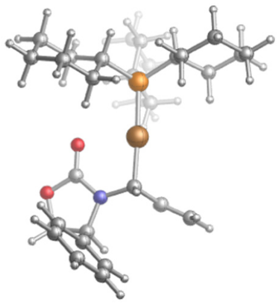

B3LYP-D3/def2-SVP-CPCM(toluene)

Zero-point correction= 0.702268 (Hartree/Particle)

Thermal correction to Energy= 0.737930

Thermal correction to Enthalpy= 0.738874

Thermal correction to Gibbs Free Energy= 0.629430

Sum of electronic and zero-point Energies= -3355.582218

Sum of electronic and thermal Energies= -3355.546557

Sum of electronic and thermal Enthalpies= -3355.545613

Sum of electronic and thermal Free Energies= -3355.655056

O	1.98770200	-2.53169000	1.62828400
C	3.15281400	-2.12027900	0.89572800
C	3.16076900	-0.57607900	0.98974200
H	4.03709900	-2.58214000	1.35156300
H	3.05068500	-2.46234000	-0.14610700
H	3.41898400	-0.12667300	0.01980700
N	1.74229000	-0.35307900	1.27718500
C	1.12079900	-1.48847500	1.72592400
O	-0.00533100	-1.59394200	2.15262400
C	1.10310900	0.90494100	1.24750600
H	0.26984200	0.99744400	1.95026800
C	1.65704700	1.99132500	0.61356800
C	2.49234200	3.01844300	0.60794500
H	3.12868900	3.22698600	1.47714500
H	2.57208200	3.68193700	-0.25665400
C	4.06889500	-0.01360200	2.07198900
C	3.93455000	-0.44605100	3.40180000
C	5.04866300	0.93692300	1.76206100

C	4.76290800	0.06756300	4.40145700
H	3.17837400	-1.19247700	3.65897400
C	5.87993500	1.45378500	2.76251500
H	5.15458400	1.28566200	0.73158800
C	5.73750500	1.02166300	4.08411100
H	4.64782600	-0.27694400	5.43220200
H	6.63755900	2.19901800	2.50745500
H	6.38391800	1.42643500	4.86686200
P	-1.58869600	0.09892100	-1.00926200
C	-2.39780900	0.45966000	-2.65872000
C	-2.96945500	0.45814400	0.22184500
C	-1.28073600	-1.74010900	-0.95822400
C	-2.85167100	1.93259400	-2.71983100
C	-1.43232400	0.14716000	-3.81810500
H	-3.28812400	-0.19023700	-2.73991400
C	-3.13290500	-0.57858500	1.34925600
C	-2.74115500	1.86216900	0.82470200
H	-3.90788000	0.47483300	-0.36158900
C	-2.35963700	-2.63834300	-1.58533900
C	0.11996800	-2.09633400	-1.49920500
H	-1.24882600	-1.94420300	0.12370000
C	-3.43375400	2.30259100	-4.09040100
H	-1.97558300	2.57005000	-2.50413500
H	-3.60210400	2.13934200	-1.94123600
C	-2.02044900	0.52881900	-5.18298600
H	-0.49677700	0.70770700	-3.64303900
H	-1.16917300	-0.92106800	-3.82270900

C	-4.25676600	-0.17573600	2.31565000
H	-2.18486800	-0.67417400	1.90236000
H	-3.35345200	-1.57326900	0.93299400
C	-3.86120400	2.26196100	1.79150900
H	-1.77805500	1.85272600	1.36484300
H	-2.63232400	2.61662100	0.03048500
C	-2.03776000	-4.12018500	-1.33906500
H	-2.40996800	-2.45745500	-2.67297500
H	-3.35566800	-2.39266800	-1.18211800
C	0.43871100	-3.57723200	-1.25413200
H	0.17553300	-1.88822100	-2.58034700
H	0.87493500	-1.44587500	-1.02856500
C	-2.44768500	1.99886800	-5.22207800
H	-3.71559700	3.36901000	-4.09632200
H	-4.36530300	1.72998600	-4.25681900
H	-1.28310900	0.31725400	-5.97566700
H	-2.89874100	-0.11022300	-5.39188900
C	-4.03146400	1.22142200	2.90360000
H	-4.33668100	-0.92484400	3.12128400
H	-5.22236000	-0.18997000	1.77646700
H	-3.64991300	3.25684900	2.21813800
H	-4.80858800	2.35540000	1.22891000
C	-0.64067700	-4.48567400	-1.85769300
H	-2.80280100	-4.75708400	-1.81424900
H	-2.08730300	-4.32237400	-0.25357800
H	1.42722400	-3.82469000	-1.67782700
H	0.50853500	-3.75577900	-0.16700200

H	-2.89237400	2.24730900	-6.20051300
H	-1.55438500	2.63936900	-5.10632800
H	-4.86539700	1.49941100	3.56988200
H	-3.11885400	1.20781500	3.52772000
H	-0.41649800	-5.54336600	-1.64022800
H	-0.62681200	-4.38107000	-2.95862600
Cu	0.28587100	1.42239200	-0.72614200
H	0.60296400	2.35944400	-1.94520300

M06-L/def2-SVP-gas//B3LYP-D3/def2-SVP-CPCM(toluene)

HF = -3355.889845

B3LYP-D3/def2-TZVPP-gas//B3LYP-D3/def2-SVP-CPCM(toluene)

HF = -3358.145514

III' *cis*

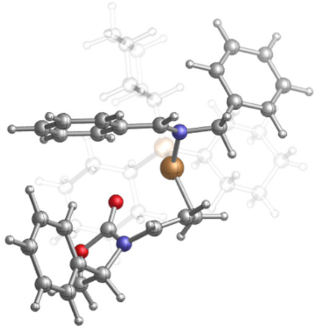

B3LYP-D3/def2-SVP-CPCM(toluene)

Zero-point correction= 0.937178 (Hartree/Particle)

Thermal correction to Energy= 0.985722

Thermal correction to Enthalpy= 0.986666

Thermal correction to Gibbs Free Energy= 0.852508

Sum of electronic and zero-point Energies= -3951.048741

Sum of electronic and thermal Energies= -3951.000198

Sum of electronic and thermal Enthalpies= -3950.999254

Sum of electronic and thermal Free Energies= -3951.133411

O	1.69054000	3.37580600	0.82984500
C	2.25608300	3.94189100	2.01699700
C	1.73393100	3.04932700	3.15860100
H	3.35475600	3.90661500	1.94085000
H	1.93506500	4.98804900	2.10667000
H	0.80351300	3.46884200	3.58188000
N	1.39040200	1.83152200	2.41073100
C	1.30168800	2.09158200	1.08024200
O	0.92782700	1.36623400	0.17420000
C	0.80684300	0.73438800	3.10256800
H	1.38886600	0.39816600	3.96006900
C	-0.51109200	0.35531600	2.93353200
H	-0.81357100	-0.44944600	3.61658700
C	-1.42546300	0.69102600	1.92215900
H	-2.45442400	0.34418200	2.04510400
H	-1.32576500	1.64489700	1.39399200
Cu	-0.53946700	-0.39309400	-0.00485700
P	-1.57902800	-0.08579700	-1.97539500
C	-1.33223300	1.63925200	-2.67324500
C	-3.46285100	-0.23050600	-1.86795600
C	-1.06739900	-1.27473700	-3.32741800
C	-1.81109400	2.68317000	-1.64236200
C	0.14314400	1.88884800	-3.04786100
H	-1.95504100	1.72474200	-3.58287200
C	-4.02198700	-1.59026200	-2.32639400

C	-3.89638800	0.04118800	-0.41231400
H	-3.88245800	0.55606400	-2.52182500
C	-1.45982000	-0.89215200	-4.76439900
C	0.42666600	-1.64174400	-3.22882400
H	-1.61464400	-2.19327500	-3.05532100
C	-1.54424100	4.12374700	-2.09730600
H	-1.27803700	2.49324200	-0.69623200
H	-2.88587500	2.55729600	-1.43876800
C	0.39099100	3.33648700	-3.49513200
H	0.76867500	1.66850400	-2.16817000
H	0.45479600	1.20542500	-3.85126700
C	-5.54216700	-1.68459200	-2.12725900
H	-3.53740200	-2.39363300	-1.74843100
H	-3.78807500	-1.77544600	-3.38513500
C	-5.41376500	-0.04189400	-0.22315900
H	-3.40361700	-0.70739800	0.23074500
H	-3.52053000	1.01523000	-0.06947800
C	-1.09505000	-2.01238900	-5.74999800
H	-0.93001700	0.02920700	-5.06016500
H	-2.53618300	-0.66335700	-4.82831600
C	0.78574200	-2.75325600	-4.22276500
H	1.05382900	-0.75794700	-3.43035500
H	0.65147800	-1.95212700	-2.19690100
C	-0.06563500	4.34104400	-2.43275200
H	-1.87191000	4.82454700	-1.31054600
H	-2.15775700	4.34505100	-2.99085400
H	1.46204800	3.47247500	-3.72249400

H	-0.15702800	3.52852700	-4.43695800
C	-5.95021800	-1.40204700	-0.67796500
H	-5.89074600	-2.68408500	-2.43891000
H	-6.04139200	-0.95596900	-2.79291300
H	-5.66636400	0.14213600	0.83489700
H	-5.90304000	0.75973100	-0.80754700
C	0.39224500	-2.37663500	-5.65703200
H	-1.35497100	-1.71120900	-6.77895600
H	-1.70354400	-2.90661300	-5.52004200
H	1.86380100	-2.97906100	-4.16388400
H	0.25418300	-3.67846100	-3.93373300
H	0.10377500	5.37468600	-2.77991800
H	0.53837600	4.20415400	-1.51819300
H	-7.04711100	-1.44714100	-0.56942200
H	-5.53207400	-2.19036000	-0.02606800
H	0.62993600	-3.20077800	-6.35059100
H	0.99646100	-1.50885700	-5.98123600
C	2.70880000	2.79825100	4.28886700
C	3.97495500	2.24819700	4.03417000
C	2.34456200	3.07847500	5.61176300
C	4.86438400	2.00102900	5.08163000
H	4.25783800	1.99168700	3.01148300
C	3.23413400	2.83355700	6.66383900
H	1.35242500	3.48791900	5.82092900
C	4.49768000	2.29721000	6.40052600
H	5.84680300	1.57128400	4.86885000
H	2.93662000	3.05910000	7.69105100

H	5.19431300	2.10454800	7.22043600
C	1.52928400	-1.51707400	1.78233000
N	0.45457700	-1.77586500	1.09343500
C	-1.32999400	-3.44062800	0.70577300
C	-0.74000600	-3.92951700	-0.46974900
C	-2.68523700	-3.70911000	0.94178200
C	-1.48609700	-4.67611500	-1.38417100
H	0.31135100	-3.70936900	-0.66377900
C	-3.43345300	-4.46521900	0.03336200
H	-3.16203000	-3.31643800	1.84456500
C	-2.83587800	-4.95115600	-1.13352700
H	-1.01242100	-5.04684600	-2.29684800
H	-4.49055200	-4.66136600	0.23022600
H	-3.42143500	-5.53237100	-1.85025400
C	-0.50948000	-2.67743900	1.72107300
H	-1.19133400	-2.11298800	2.37504100
H	0.01435700	-3.40622500	2.37079200
H	1.64221100	-1.95468000	2.78671700
C	2.76020900	-0.92985600	1.23851600
C	3.84760800	-0.69213700	2.10089300
C	2.94086000	-0.71141400	-0.14117300
C	5.07963200	-0.26403400	1.60292100
H	3.71961900	-0.84370800	3.17610300
C	4.17160300	-0.28920000	-0.63925200
H	2.10061800	-0.87951300	-0.81421400
C	5.24995700	-0.06491700	0.22791300
H	5.91140200	-0.08842000	2.29050500

H 4.29374100 -0.13382400 -1.71458300

H 6.21525300 0.26309900 -0.16628000

M06-L/def2-SVP-gas//B3LYP-D3/def2-SVP-CPCM(toluene)

HF = -3951.484104

B3LYP-D3/def2-TZVPP-gas//B3LYP-D3/def2-SVP-CPCM(toluene)

HF = -3954.480942

TS-III'-IV *cis (S,S,S)*

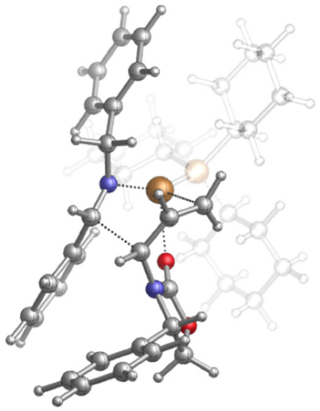

B3LYP-D3/def2-SVP-CPCM(toluene)

Zero-point correction= 0.937109 (Hartree/Particle)

Thermal correction to Energy= 0.984712

Thermal correction to Enthalpy= 0.985656

Thermal correction to Gibbs Free Energy= 0.853058

Sum of electronic and zero-point Energies= -3951.035476

Sum of electronic and thermal Energies= -3950.987872

Sum of electronic and thermal Enthalpies= -3950.986928

Sum of electronic and thermal Free Energies= -3951.119527

O -2.39973800 5.94181800 -2.21605300

C -2.54887100 6.55861600 -0.92869700

C -2.23079000 5.41023800 0.04854800

H	-1.86613200	7.41363600	-0.85190200
H	-3.59354700	6.89721600	-0.82201600
H	-2.85125500	5.47724400	0.95438700
N	-2.66989600	4.26879600	-0.76637700
C	-2.69838700	4.61442600	-2.08304900
O	-2.95581000	3.92194700	-3.04778200
C	-3.32677100	3.12947000	-0.16832300
H	-2.85195300	2.85564100	0.77567500
C	-4.67552700	2.97925900	-0.32299600
H	-5.11901000	2.22051100	0.33364300
C	-5.46648700	3.36925300	-1.46103700
H	-6.53957100	3.16136700	-1.38244600
H	-5.25098000	4.33717300	-1.92878200
Cu	-4.64420400	2.27544000	-3.04284700
P	-5.39055100	2.65151400	-5.16933600
C	-5.15909600	4.41719600	-5.78871500
C	-7.25879700	2.38281400	-5.31522500
C	-4.66699700	1.55285600	-6.50212900
C	-5.66856700	5.41720700	-4.72971300
C	-3.69043100	4.71286300	-6.16220300
H	-5.78693400	4.51410000	-6.69404500
C	-7.62842800	0.94235100	-5.72383100
C	-7.93338600	2.72430600	-3.96775500
H	-7.62836900	3.07696500	-6.09194600
C	-4.98616300	1.92491000	-7.95978500
C	-3.16918100	1.26898500	-6.28717300
H	-5.16614500	0.59211800	-6.29042200

C	-5.42438300	6.87521600	-5.13424600
H	-5.14197000	5.20787000	-3.78541100
H	-6.74165900	5.26321100	-4.54144400
C	-3.45098400	6.19040600	-6.50879900
H	-3.04616600	4.43039500	-5.31681500
H	-3.39198800	4.09891400	-7.02320100
C	-9.14496800	0.70124200	-5.71847400
H	-7.14990100	0.23750300	-5.02536600
H	-7.23983200	0.70655200	-6.72499800
C	-9.44911700	2.49878000	-3.99610800
H	-7.48285500	2.08027300	-3.19304900
H	-7.70841000	3.75690100	-3.66577400
C	-4.46832700	0.84760300	-8.92564700
H	-4.51672700	2.88898400	-8.21547300
H	-6.07111000	2.06520500	-8.09506400
C	-2.67762300	0.18850800	-7.25817100
H	-2.57769600	2.18454700	-6.43733000
H	-3.00989100	0.96047900	-5.24236600
C	-3.93688300	7.12038900	-5.39403400
H	-5.81050300	7.55163400	-4.35278200
H	-5.99696200	7.09736900	-6.05447800
H	-2.37878200	6.35121200	-6.71275100
H	-3.98749800	6.43542400	-7.44501300
C	-9.77244400	1.04928400	-4.36659100
H	-9.34967800	-0.34944300	-5.98557900
H	-9.61179700	1.32386700	-6.50446800
H	-9.88682500	2.76328600	-3.01852100

H	-9.90621000	3.17764300	-4.74020500
C	-2.97380900	0.57106200	-8.71395500
H	-4.65844400	1.15448500	-9.96813800
H	-5.03759700	-0.08632400	-8.76322600
H	-1.59878400	0.01027200	-7.11459400
H	-3.18753400	-0.76406600	-7.02513100
H	-3.74691400	8.17630000	-5.65108200
H	-3.37113900	6.90375400	-4.47009800
H	-10.86143500	0.87434500	-4.38057400
H	-9.35035200	0.38067900	-3.59464600
H	-2.62956700	-0.22169400	-9.39934500
H	-2.39969000	1.48055800	-8.97197600
C	-0.76218200	5.38354600	0.44212500
C	0.17392300	4.60385100	-0.24533500
C	-0.32021300	6.23487900	1.46823500
C	1.53087600	4.66927800	0.09033800
H	-0.15383900	3.94438800	-1.04800600
C	1.03387600	6.30253500	1.80268500
H	-1.04405700	6.85089700	2.01112500
C	1.96558200	5.51683800	1.11238800
H	2.24483800	4.04772900	-0.45489000
H	1.36373100	6.96698100	2.60557100
H	3.02578900	5.56622600	1.37358000
C	-2.84678700	1.19998500	-1.10997000
N	-3.77126800	0.89487100	-1.96090200
C	-5.43487700	-0.83174500	-2.61724900
C	-4.68606800	-1.23489700	-3.73443500

C	-6.77629000	-1.23371700	-2.53326000
C	-5.26060800	-2.02582500	-4.73313000
H	-3.64880100	-0.90580500	-3.81430800
C	-7.35350400	-2.03178500	-3.52719500
H	-7.38095400	-0.91384900	-1.67970100
C	-6.59663800	-2.43190300	-4.63247900
H	-4.66179500	-2.32681900	-5.59662700
H	-8.40342300	-2.32504100	-3.44628700
H	-7.04787500	-3.04471700	-5.41695700
C	-4.79583900	-0.03289200	-1.49406300
H	-5.59939100	0.49439400	-0.94455600
H	-4.35701900	-0.74518400	-0.76764900
H	-2.88149700	0.70610500	-0.12317000
C	-1.43870500	1.45323000	-1.51437900
C	-0.41883100	1.25138300	-0.56582800
C	-1.06842500	1.75119400	-2.83787400
C	0.92791800	1.34820400	-0.92277400
H	-0.68775100	1.00612100	0.46625500
C	0.27616700	1.87084900	-3.19071600
H	-1.85170200	1.88460500	-3.58096200
C	1.28322000	1.67085500	-2.23647100
H	1.70214100	1.17967100	-0.17006200
H	0.54264100	2.11264800	-4.22334900
H	2.33582100	1.75901700	-2.51774900

M06-L/def2-SVP-gas//B3LYP-D3/def2-SVP-CPCM(toluene)

HF = -3951.468767

B3LYP-D3/def2-TZVPP-gas//B3LYP-D3/def2-SVP-CPCM(toluene)

HF = -3954.466648

IV *(S,S,S)*

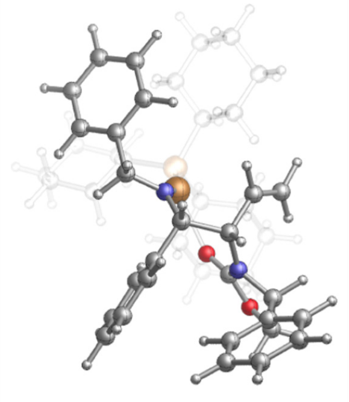

B3LYP-D3/def2-SVP-CPCM(toluene)

Zero-point correction= 0.939137 (Hartree/Particle)

Thermal correction to Energy= 0.986955

Thermal correction to Enthalpy= 0.987899

Thermal correction to Gibbs Free Energy= 0.854840

Sum of electronic and zero-point Energies= -3951.051519

Sum of electronic and thermal Energies= -3951.003702

Sum of electronic and thermal Enthalpies= -3951.002758

Sum of electronic and thermal Free Energies= -3951.135816

O	1.63978000	3.35541900	0.84746900
C	2.16855900	3.85163400	2.08040700
C	1.68315300	2.84605200	3.14633200
H	3.26790200	3.87023400	2.01452400
H	1.79827100	4.87106400	2.25039800
H	0.71293900	3.17025100	3.56449700
N	1.44936700	1.66036300	2.29142400
C	1.28677200	2.05380200	1.00183200

O	0.86958000	1.40313400	0.06313700
C	0.94107600	0.38895300	2.83690600
H	1.45255200	0.28509100	3.80271200
C	-0.53824800	0.36346800	3.04959900
H	-0.86736700	-0.31874400	3.84178100
C	-1.46933600	0.92197400	2.26067400
H	-2.53333600	0.74314000	2.43568800
H	-1.22302700	1.60239600	1.44474400
Cu	-0.49415500	-0.46620500	-0.13047300
P	-1.59243500	-0.06710700	-1.99700300
C	-1.34644100	1.65407300	-2.69399000
C	-3.46749200	-0.22226600	-1.86039000
C	-1.07190900	-1.26815700	-3.33382600
C	-1.82017600	2.69395300	-1.65646900
C	0.12851400	1.89641700	-3.07712200
H	-1.97419300	1.74497800	-3.59959700
C	-4.02564200	-1.58213900	-2.32020000
C	-3.87977000	0.04128500	-0.39532600
H	-3.90251800	0.56617800	-2.50154600
C	-1.46660500	-0.89333100	-4.77180400
C	0.42424600	-1.63195200	-3.23462500
H	-1.61695100	-2.18526800	-3.05336600
C	-1.54390600	4.13539900	-2.10154900
H	-1.28708800	2.49898800	-0.71133400
H	-2.89601700	2.57241800	-1.45492600
C	0.38562000	3.34711200	-3.50730500
H	0.76058500	1.65894400	-2.20843500

H	0.42564800	1.22089700	-3.89249900
C	-5.54401500	-1.67679400	-2.11295500
H	-3.53660400	-2.38712000	-1.74824500
H	-3.79689700	-1.76116300	-3.38105700
C	-5.39705100	-0.04496800	-0.19620400
H	-3.37620400	-0.70857700	0.24084300
H	-3.50550000	1.01901200	-0.05662700
C	-1.09893700	-2.01589800	-5.75318900
H	-0.94025300	0.02861300	-5.07172300
H	-2.54386200	-0.66857400	-4.83621100
C	0.78351700	-2.74649800	-4.22509600
H	1.04951600	-0.74768100	-3.43951400
H	0.65340900	-1.94227100	-2.20393500
C	-0.06341000	4.34375500	-2.43418700
H	-1.86776500	4.83366800	-1.31092100
H	-2.15504200	4.36604900	-2.99421400
H	1.45746800	3.47807200	-3.73357700
H	-0.16125300	3.55433400	-4.44652600
C	-5.94019400	-1.40058400	-0.65917100
H	-5.89522700	-2.67498600	-2.42532500
H	-6.04736000	-0.94538800	-2.77246400
H	-5.64470300	0.13045800	0.86450800
H	-5.88754300	0.76248300	-0.77134600
C	0.38933000	-2.37571600	-5.66023100
H	-1.36095000	-1.71866100	-6.78273900
H	-1.70465300	-2.91096400	-5.51949400
H	1.86182400	-2.97027700	-4.16489100

H	0.25354400	-3.67109900	-3.93187900
H	0.11416800	5.37948700	-2.77065100
H	0.53875300	4.19272800	-1.52071000
H	-7.03642500	-1.44113300	-0.54295500
H	-5.52092600	-2.19487800	-0.01563000
H	0.62832700	-3.20149700	-6.35136600
H	0.99119300	-1.50746600	-5.98761500
C	2.66548000	2.66203600	4.28371200
C	3.96252200	2.18545900	4.03615900
C	2.30329000	2.98945700	5.59786700
C	4.86838500	2.01300100	5.08413800
H	4.25587200	1.92409000	3.01853300
C	3.21330600	2.82754600	6.64935400
H	1.29700700	3.36569900	5.80262600
C	4.49508800	2.33077300	6.39576600
H	5.86919300	1.62587900	4.87610700
H	2.91547000	3.08280000	7.66942600
H	5.20304700	2.19285200	7.21692300
C	1.37550800	-1.02920300	1.99319900
N	0.40154800	-1.59543400	1.13683000
C	-1.27753000	-3.38609400	0.74114100
C	-0.70015700	-3.88165700	-0.44097800
C	-2.63306800	-3.66814100	0.97605200
C	-1.44680500	-4.63710800	-1.34968200
H	0.34875800	-3.65194200	-0.63768000
C	-3.38617900	-4.43028100	0.07244400
H	-3.10977200	-3.27931200	1.88106300

C	-2.79595100	-4.91870500	-1.09666000
H	-0.97299500	-5.01107800	-2.26145200
H	-4.44117500	-4.63038200	0.27870000
H	-3.38175100	-5.50571100	-1.80886100
C	-0.44295100	-2.60000600	1.74709000
H	-1.14797600	-2.19308800	2.50995300
H	0.17308200	-3.34172800	2.31132600
H	1.56400600	-1.70341200	2.85305200
C	2.73593800	-0.78083900	1.34026000
C	3.86623600	-0.60875000	2.15301600
C	2.90780600	-0.69006300	-0.04991500
C	5.11585800	-0.29674900	1.60746700
H	3.76405600	-0.69628600	3.23854500
C	4.15106400	-0.36920700	-0.60256100
H	2.04683100	-0.85313800	-0.69593700
C	5.26075400	-0.15573400	0.22361800
H	5.97675400	-0.15793400	2.26778800
H	4.25202400	-0.28243900	-1.68818600
H	6.23192900	0.10149600	-0.20764200

M06-L/def2-SVP-gas//B3LYP-D3/def2-SVP-CPCM(toluene)

HF = -3951.483587

B3LYP-D3/def2-TZVPP-gas//B3LYP-D3/def2-SVP-CPCM(toluene)

HF = -3954.483565

15a

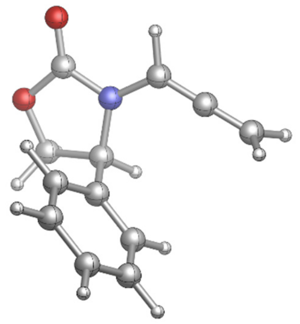

B3LYP-D3/def2-SVP-CPCM(toluene)

Zero-point correction= 0.205249 (Hartree/Particle)

Thermal correction to Energy= 0.218091

Thermal correction to Enthalpy= 0.219035

Thermal correction to Gibbs Free Energy= 0.164339

Sum of electronic and zero-point Energies= -668.394748

Sum of electronic and thermal Energies= -668.381906

Sum of electronic and thermal Enthalpies= -668.380962

Sum of electronic and thermal Free Energies= -668.435658

O	0.03769900	-0.35934100	2.19386000
C	0.29398200	0.92229700	2.93270200
C	-1.04031100	1.18566900	3.66247800
H	1.16578800	0.76629000	3.56122500
H	0.49653000	1.69413200	2.18495100
H	-1.24911000	2.25983700	3.70132500
N	-1.97096600	0.54986700	2.67432100
C	-1.33302000	-0.41836900	1.93111000
O	-1.83137300	-1.23760100	1.13114800
C	-3.31530000	0.92391600	2.41785700
H	-3.82323700	0.27430500	1.70743400
C	-3.92508200	1.97902100	2.93807800
C	-4.57406000	2.99226300	3.48319900

H	-5.14039000	2.87726500	4.40955200
H	-4.57276700	3.98594100	3.03216200
C	-1.18005900	0.57823500	5.06930700
C	-2.45986300	0.50541600	5.67056300
C	-0.06783100	0.12073300	5.80890800
C	-2.62261200	-0.01577000	6.96645300
H	-3.33639500	0.84911400	5.13293300
C	-0.22855000	-0.39988000	7.10943300
H	0.93457100	0.15974100	5.39627700
C	-1.50550900	-0.47137100	7.69554000
H	-3.61676400	-0.06299400	7.40477300
H	0.64384900	-0.74729500	7.65799400
H	-1.62895700	-0.87238600	8.69873000

M06-L/def2-SVP-gas//B3LYP-D3/def2-SVP-CPCM(toluene)

HF = -668.4895248

B3LYP-D3/def2-TZVPP-gas//B3LYP-D3/def2-SVP-CPCM(toluene)

HF = -669.3378016

[Cu]-H

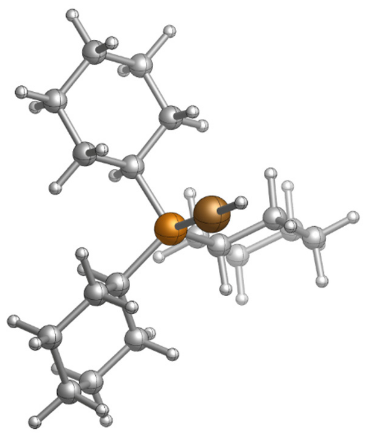

B3LYP-D3/def2-SVP-CPCM(toluene)

Zero-point correction= 0.489971 (Hartree/Particle)

Thermal correction to Energy= 0.511792

Thermal correction to Enthalpy= 0.512736

Thermal correction to Gibbs Free Energy= 0.439237

Sum of electronic and zero-point Energies= -2687.114380

Sum of electronic and thermal Energies= -2687.092558

Sum of electronic and thermal Enthalpies= -2687.091614

Sum of electronic and thermal Free Energies= -2687.165114

P	-8.68811700	1.44905100	1.47485000
C	-9.05026700	0.24450000	0.09398200
C	-8.75973000	3.16459800	0.73266200
C	-10.16568000	1.39941100	2.63963000
C	-10.11560200	0.68968300	-0.92204300
C	-7.76176800	-0.23128100	-0.60954200
H	-9.44241300	-0.63435900	0.63380700
C	-8.66310500	4.23303700	1.84117300
C	-7.62349200	3.35138500	-0.29259100
H	-9.73282900	3.27376600	0.22147200
C	-10.97132700	0.08664000	2.61612400
C	-9.67214300	1.68387500	4.07614000
H	-10.84126100	2.21549800	2.32553700
C	-10.41286400	-0.42882600	-1.93180300
H	-9.75383500	1.57677100	-1.46972700
H	-11.04149300	0.99714700	-0.40825000
C	-8.07103600	-1.34482900	-1.61891000
H	-7.28406800	0.61104500	-1.13682500
H	-7.03350600	-0.57424300	0.14405700

C	-8.61563700	5.65528100	1.26747800
H	-7.74794200	4.04213800	2.43206500
H	-9.51247800	4.14931100	2.53626700
C	-7.57654400	4.77862600	-0.85328600
H	-6.66460600	3.11353700	0.20439400
H	-7.73001000	2.63655600	-1.12166600
C	-12.13016300	0.12674900	3.62402300
H	-10.30295200	-0.75777400	2.86580100
H	-11.37286000	-0.11064900	1.61071000
C	-10.82906900	1.72829400	5.08045900
H	-8.96181100	0.88566200	4.35934800
H	-9.09526700	2.62033500	4.11405600
C	-9.13451200	-0.90497900	-2.63329900
H	-11.15298300	-0.08040400	-2.67153000
H	-10.87574600	-1.28053500	-1.40017300
H	-7.14629900	-1.64992600	-2.13624400
H	-8.43499800	-2.23519900	-1.07371500
C	-7.47128000	5.82149200	0.26335300
H	-8.51961200	6.38246900	2.09090200
H	-9.57586900	5.87395300	0.76474500
H	-6.73088500	4.87244600	-1.55474700
H	-8.49466800	4.96552900	-1.44046800
C	-11.64480800	0.43151800	5.04524300
H	-12.67583000	-0.83133500	3.59838200
H	-12.84921300	0.90588900	3.31032900
H	-10.43659600	1.91250700	6.09430000
H	-11.48756700	2.58276100	4.83742500

H	-9.36421900	-1.72800900	-3.33039600
H	-8.72967200	-0.07810100	-3.24601000
H	-7.47155300	6.84057000	-0.15800000
H	-6.50649200	5.69568900	0.78817300
H	-12.50044300	0.49247100	5.73828900
H	-11.01128400	-0.40282400	5.39838800
Cu	-6.70930400	1.11559900	2.48473900
H	-5.34066600	0.90186300	3.16451000
P	-8.68811700	1.44905100	1.47485000
C	-9.05026700	0.24450000	0.09398200
C	-8.75973000	3.16459800	0.73266200
C	-10.16568000	1.39941100	2.63963000
C	-10.11560200	0.68968300	-0.92204300
C	-7.76176800	-0.23128100	-0.60954200
H	-9.44241300	-0.63435900	0.63380700
C	-8.66310500	4.23303700	1.84117300
C	-7.62349200	3.35138500	-0.29259100
H	-9.73282900	3.27376600	0.22147200
C	-10.97132700	0.08664000	2.61612400
C	-9.67214300	1.68387500	4.07614000
H	-10.84126100	2.21549800	2.32553700
C	-10.41286400	-0.42882600	-1.93180300
H	-9.75383500	1.57677100	-1.46972700
H	-11.04149300	0.99714700	-0.40825000
C	-8.07103600	-1.34482900	-1.61891000
H	-7.28406800	0.61104500	-1.13682500
H	-7.03350600	-0.57424300	0.14405700

C	-8.61563700	5.65528100	1.26747800
H	-7.74794200	4.04213800	2.43206500
H	-9.51247800	4.14931100	2.53626700
C	-7.57654400	4.77862600	-0.85328600
H	-6.66460600	3.11353700	0.20439400
H	-7.73001000	2.63655600	-1.12166600
C	-12.13016300	0.12674900	3.62402300
H	-10.30295200	-0.75777400	2.86580100
H	-11.37286000	-0.11064900	1.61071000
C	-10.82906900	1.72829400	5.08045900
H	-8.96181100	0.88566200	4.35934800
H	-9.09526700	2.62033500	4.11405600
C	-9.13451200	-0.90497900	-2.63329900
H	-11.15298300	-0.08040400	-2.67153000
H	-10.87574600	-1.28053500	-1.40017300
H	-7.14629900	-1.64992600	-2.13624400
H	-8.43499800	-2.23519900	-1.07371500
C	-7.47128000	5.82149200	0.26335300
H	-8.51961200	6.38246900	2.09090200
H	-9.57586900	5.87395300	0.76474500
H	-6.73088500	4.87244600	-1.55474700
H	-8.49466800	4.96552900	-1.44046800
C	-11.64480800	0.43151800	5.04524300
H	-12.67583000	-0.83133500	3.59838200
H	-12.84921300	0.90588900	3.31032900
H	-10.43659600	1.91250700	6.09430000
H	-11.48756700	2.58276100	4.83742500

H	-9.36421900	-1.72800900	-3.33039600
H	-8.72967200	-0.07810100	-3.24601000
H	-7.47155300	6.84057000	-0.15800000
H	-6.50649200	5.69568900	0.78817300
H	-12.50044300	0.49247100	5.73828900
H	-11.01128400	-0.40282400	5.39838800
Cu	-6.70930400	1.11559900	2.48473900
H	-5.34066600	0.90186300	3.16451000

M06-L/def2-SVP-gas//B3LYP-D3/def2-SVP-CPCM(toluene)

HF = -2687.313561

B3LYP-D3/def2-TZVPP-gas//B3LYP-D3/def2-SVP-CPCM(toluene)

HF = -2688.733196

Imine

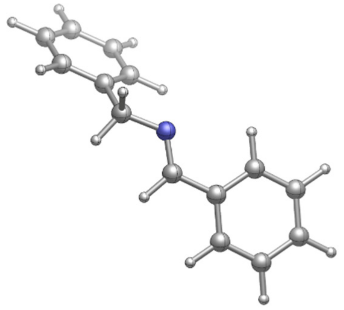

B3LYP-D3/def2-SVP-CPCM(toluene)

Zero-point correction= 0.231324 (Hartree/Particle)

Thermal correction to Energy= 0.242723

Thermal correction to Enthalpy= 0.243667

Thermal correction to Gibbs Free Energy= 0.192235

Sum of electronic and zero-point Energies= -595.424997

Sum of electronic and thermal Energies= -595.413598

Sum of electronic and thermal Enthalpies= -595.412654

Sum of electronic and thermal Free Energies= -595.464087

C	0.20822402	-3.08812667	-0.01752813
C	1.60338402	-3.08812667	-0.01752813
C	2.30092202	-1.88037567	-0.01752813
C	1.60326802	-0.67186667	-0.01872713
C	0.20844302	-0.67194467	-0.01920613
C	-0.48915798	-1.88015067	-0.01821013
H	-0.34153498	-4.04044367	-0.01707813
H	2.15289202	-4.04063967	-0.01621313
H	3.40060202	-1.88029567	-0.01689413
H	-0.34167898	0.28033633	-0.02015913
H	-1.58876198	-1.87996767	-0.01839013
C	2.37377210	0.66152130	-0.01880975
H	3.44376877	0.65899798	-0.01794682
N	1.73183948	1.78460733	-0.01991884
C	2.40570589	2.94017658	-0.01766960
H	3.02317442	2.99468043	-0.88982896
H	3.01903207	2.99369561	0.85746851
C	1.41005648	4.11502950	-0.01936733
C	0.95655099	4.64683637	1.18772105
C	0.96208438	4.64779385	-1.22845502
C	0.05471900	5.71133970	1.18595111
H	1.30983997	4.22719683	2.14073332
C	0.06010360	5.71173195	-1.23030316
H	1.32014265	4.22855228	-2.18003528

C	-0.39397314	6.24341223	-0.02268280
H	-0.30321900	6.12987883	2.13776369
H	-0.29376909	6.13145524	-2.18314250
H	-1.10529034	7.08205529	-0.02415871

M06-L/def2-SVP-gas//B3LYP-D3/def2-SVP-CPCM(toluene)

HF = -595.5515182

B3LYP-D3/def2-TZVPP-gas//B3LYP-D3/def2-SVP-CPCM(toluene)

HF = -596.3014042

[Si]-H

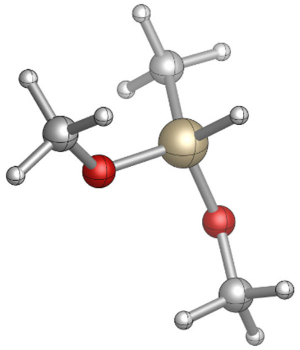

B3LYP-D3/def2-SVP-CPCM(toluene)

Zero-point correction= 0.129476 (Hartree/Particle)

Thermal correction to Energy= 0.139432

Thermal correction to Enthalpy= 0.140376

Thermal correction to Gibbs Free Energy= 0.093337

Sum of electronic and zero-point Energies= -559.959068

Sum of electronic and thermal Energies= -559.949112

Sum of electronic and thermal Enthalpies= -559.948168

Sum of electronic and thermal Free Energies= -559.995207

Si	-7.72559635	7.37652277	1.77112694
H	-7.23558787	8.06948103	2.97137713

O	-9.55559635	7.37654531	1.77112694
O	-7.11561729	5.65117482	1.77112694
C	-10.03295191	8.72451849	1.77112639
H	-11.10294998	8.72397200	1.76916999
H	-9.67814067	9.22853777	2.64575405
H	-9.67494703	9.22966856	0.89845468
C	-5.68561987	5.65043834	1.76851313
H	-5.32947197	4.64144944	1.76881714
H	-5.33029258	6.15383042	0.89373377
H	-5.32709743	6.15548409	2.64103273
C	-7.07891849	8.29103911	0.18712327
H	-6.00891864	8.29152008	0.18740885
H	-7.43512569	7.78631091	-0.68652762
H	-7.43603848	9.29968436	0.18683746
Si	-7.72559635	7.37652277	1.77112694
H	-7.23558787	8.06948103	2.97137713
O	-9.55559635	7.37654531	1.77112694
O	-7.11561729	5.65117482	1.77112694
C	-10.03295191	8.72451849	1.77112639
H	-11.10294998	8.72397200	1.76916999
H	-9.67814067	9.22853777	2.64575405
H	-9.67494703	9.22966856	0.89845468
C	-5.68561987	5.65043834	1.76851313
H	-5.32947197	4.64144944	1.76881714
H	-5.33029258	6.15383042	0.89373377
H	-5.32709743	6.15548409	2.64103273
C	-7.07891849	8.29103911	0.18712327

H -6.00891864 8.29152008 0.18740885

H -7.43512569 7.78631091 -0.68652762

H -7.43603848 9.29968436 0.18683746

M06-L/def2-SVP-gas//B3LYP-D3/def2-SVP-CPCM(toluene)

HF = -560.0115474

B3LYP-D3/def2-TZVPP-gas//B3LYP-D3/def2-SVP-CPCM(toluene)

HF = -560.524137

[Si]-OR

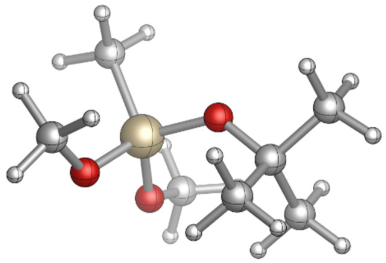

B3LYP-D3/def2-SVP-CPCM(toluene)

Zero-point correction= 0.247875 (Hartree/Particle)

Thermal correction to Energy= 0.264332

Thermal correction to Enthalpy= 0.265276

Thermal correction to Gibbs Free Energy= 0.202064

Sum of electronic and zero-point Energies= -792.217774

Sum of electronic and thermal Energies= -792.201317

Sum of electronic and thermal Enthalpies= -792.200373

Sum of electronic and thermal Free Energies= -792.263585

Si -7.72559635 7.37652277 1.77112694

O -9.55559635 7.37654531 1.77112694

O -7.11561729 5.65117482 1.77112694

C	-10.03295191	8.72451849	1.77112639
H	-11.10294998	8.72397200	1.76916999
H	-9.67814067	9.22853777	2.64575405
H	-9.67494703	9.22966856	0.89845468
C	-5.68561987	5.65043834	1.76851313
H	-5.32947197	4.64144944	1.76881714
H	-5.33029258	6.15383042	0.89373377
H	-5.32709743	6.15548409	2.64103273
C	-7.07891849	8.29103911	0.18712327
H	-6.00891864	8.29152008	0.18740885
H	-7.43512569	7.78631091	-0.68652762
H	-7.43603848	9.29968436	0.18683746
O	-7.11558579	8.23918510	3.26531596
C	-7.50200826	7.50628511	4.43083528
C	-6.98852162	8.23289485	5.68780376
H	-7.40927062	9.21585905	5.72844424
H	-7.27797380	7.68469184	6.55992239
H	-5.92154738	8.30298564	5.64839250
C	-9.03761387	7.40494621	4.48780750
H	-9.46794099	8.14443174	3.84525033
H	-9.34299698	6.43132798	4.16576913
H	-9.36884995	7.56866814	5.49198765
C	-6.89603635	6.09173765	4.37206868
H	-7.63115357	5.40355720	4.01025333
H	-6.05288863	6.09200091	3.71328051
H	-6.58303458	5.79682032	5.35184093

M06-L/def2-SVP-gas//B3LYP-D3/def2-SVP-CPCM(toluene)

HF = -792.339444

B3LYP-D3/def2-TZVPP-gas//B3LYP-D3/def2-SVP-CPCM(toluene)

HF = -793.1682918

[Cu]-OR

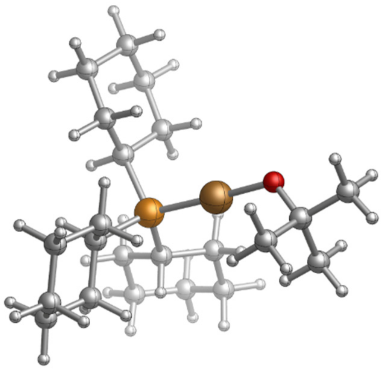

B3LYP-D3/def2-SVP-CPCM(toluene)

Zero-point correction= 0.608685 (Hartree/Particle)

Thermal correction to Energy= 0.637351

Thermal correction to Enthalpy= 0.638296

Thermal correction to Gibbs Free Energy= 0.547703

Sum of electronic and zero-point Energies= -2919.356235

Sum of electronic and thermal Energies= -2919.327569

Sum of electronic and thermal Enthalpies= -2919.326624

Sum of electronic and thermal Free Energies= -2919.417217

P	-8.68811700	1.44905100	1.47485000
C	-9.05026700	0.24450000	0.09398200
C	-8.75973000	3.16459800	0.73266200
C	-10.16568000	1.39941100	2.63963000
C	-10.11560200	0.68968300	-0.92204300
C	-7.76176800	-0.23128100	-0.60954200
H	-9.44241300	-0.63435900	0.63380700

C	-8.66310500	4.23303700	1.84117300
C	-7.62349200	3.35138500	-0.29259100
H	-9.73282900	3.27376600	0.22147200
C	-10.97132700	0.08664000	2.61612400
C	-9.67214300	1.68387500	4.07614000
H	-10.84126100	2.21549800	2.32553700
C	-10.41286400	-0.42882600	-1.93180300
H	-9.75383500	1.57677100	-1.46972700
H	-11.04149300	0.99714700	-0.40825000
C	-8.07103600	-1.34482900	-1.61891000
H	-7.28406800	0.61104500	-1.13682500
H	-7.03350600	-0.57424300	0.14405700
C	-8.61563700	5.65528100	1.26747800
H	-7.74794200	4.04213800	2.43206500
H	-9.51247800	4.14931100	2.53626700
C	-7.57654400	4.77862600	-0.85328600
H	-6.66460600	3.11353700	0.20439400
H	-7.73001000	2.63655600	-1.12166600
C	-12.13016300	0.12674900	3.62402300
H	-10.30295200	-0.75777400	2.86580100
H	-11.37286000	-0.11064900	1.61071000
C	-10.82906900	1.72829400	5.08045900
H	-8.96181100	0.88566200	4.35934800
H	-9.09526700	2.62033500	4.11405600
C	-9.13451200	-0.90497900	-2.63329900
H	-11.15298300	-0.08040400	-2.67153000
H	-10.87574600	-1.28053500	-1.40017300

H	-7.14629900	-1.64992600	-2.13624400
H	-8.43499800	-2.23519900	-1.07371500
C	-7.47128000	5.82149200	0.26335300
H	-8.51961200	6.38246900	2.09090200
H	-9.57586900	5.87395300	0.76474500
H	-6.73088500	4.87244600	-1.55474700
H	-8.49466800	4.96552900	-1.44046800
C	-11.64480800	0.43151800	5.04524300
H	-12.67583000	-0.83133500	3.59838200
H	-12.84921300	0.90588900	3.31032900
H	-10.43659600	1.91250700	6.09430000
H	-11.48756700	2.58276100	4.83742500
H	-9.36421900	-1.72800900	-3.33039600
H	-8.72967200	-0.07810100	-3.24601000
H	-7.47155300	6.84057000	-0.15800000
H	-6.50649200	5.69568900	0.78817300
H	-12.50044300	0.49247100	5.73828900
H	-11.01128400	-0.40282400	5.39838800
Cu	-6.70930400	1.11559900	2.48473900
O	-5.08612915	0.86211276	3.29093260
C	-4.96527355	-0.50213138	3.70218902
C	-6.09249694	-0.81032632	4.66488586
C	-5.05959324	-1.37995344	2.47222864
C	-3.62283933	-0.67732586	4.38005160
H	-6.03359671	-0.14353278	5.55840414
H	-7.08054428	-0.65479854	4.16888148
H	-6.03372026	-1.87045617	5.01066968

H	-4.24189704	-1.13151417	1.75380999
H	-4.96906620	-2.45724760	2.75182429
H	-6.04064813	-1.22887953	1.96117674
H	-3.48929488	-1.73327057	4.71785613
H	-2.79533216	-0.42472473	3.67444514
H	-3.54743542	-0.00912278	5.27128455

M06-L/def2-SVP-gas//B3LYP-D3/def2-SVP-CPCM(toluene)

HF = -2919.63165

B3LYP-D3/def2-TZVPP-gas//B3LYP-D3/def2-SVP-CPCM(toluene)

HF = -2921.356927

tBuOH

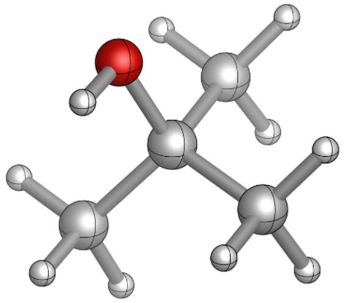

B3LYP-D3/def2-SVP-CPCM(toluene)

Zero-point correction= 0.135049 (Hartree/Particle)

Thermal correction to Energy= 0.141632

Thermal correction to Enthalpy= 0.142576

Thermal correction to Gibbs Free Energy= 0.106228

Sum of electronic and zero-point Energies= -233.381645

Sum of electronic and thermal Energies= -233.375061

Sum of electronic and thermal Enthalpies= -233.374117

Sum of electronic and thermal Free Energies= -233.410465

C	-4.96527355	-0.50213138	3.70218902

C	-6.09249694	-0.81032632	4.66488586
C	-5.05959324	-1.37995344	2.47222864
C	-3.62283933	-0.67732586	4.38005160
H	-6.03359671	-0.14353278	5.55840414
H	-7.08054428	-0.65479854	4.16888148
H	-6.03372026	-1.87045617	5.01066968
H	-4.24189704	-1.13151417	1.75380999
H	-4.96906620	-2.45724760	2.75182429
H	-6.04064813	-1.22887953	1.96117674
H	-3.48929488	-1.73327057	4.71785613
H	-2.79533216	-0.42472473	3.67444514
H	-3.54743542	-0.00912278	5.27128455
O	-5.08612915	0.86211276	3.29093260
H	-5.93285487	0.99240792	2.85771357

M06-L/def2-SVP-gas//B3LYP-D3/def2-SVP-CPCM(toluene)

HF = -233.4673658

B3LYP-D3/def2-TZVPP-gas//B3LYP-D3/def2-SVP-CPCM(toluene)

HF = -233.7890572

V

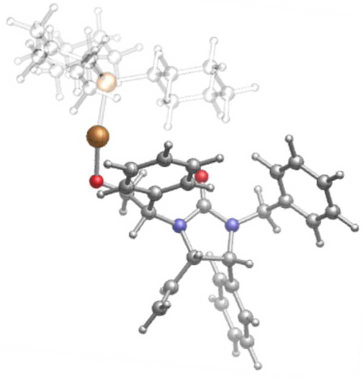

B3LYP-D3/def2-SVP-CPCM(toluene)

Zero-point correction= 0.940807 (Hartree/Particle)

Thermal correction to Energy= 0.988672

Thermal correction to Enthalpy= 0.989616

Thermal correction to Gibbs Free Energy= 0.854147

Sum of electronic and zero-point Energies= -3951.060126

Sum of electronic and thermal Energies= -3951.012261

Sum of electronic and thermal Enthalpies= -3951.011317

Sum of electronic and thermal Free Energies= -3951.146786

C	0.20822402	-3.08812667	-0.01752813
C	1.60338402	-3.08812667	-0.01752813
C	2.30092202	-1.88037567	-0.01752813
C	1.60326802	-0.67186667	-0.01872713
C	0.20844302	-0.67194467	-0.01920613
C	-0.48915798	-1.88015067	-0.01821013
H	-0.34153498	-4.04044367	-0.01707813
H	2.15289202	-4.04063967	-0.01621313
H	3.40060202	-1.88029567	-0.01689413
H	-0.34167898	0.28033633	-0.02015913
H	-1.58876198	-1.87996767	-0.01839013
C	2.37377210	0.66152130	-0.01880975
H	3.44376877	0.65899798	-0.01794682
N	1.73183948	1.78460733	-0.01991884
C	2.40570589	2.94017658	-0.01766960
H	3.02317442	2.99468043	-0.88982896
H	3.01903207	2.99369561	0.85746851
C	1.41005648	4.11502950	-0.01936733

C	0.95655099	4.64683637	1.18772105
C	0.96208438	4.64779385	-1.22845502
C	0.05471900	5.71133970	1.18595111
H	1.30983997	4.22719683	2.14073332
C	0.06010360	5.71173195	-1.23030316
H	1.32014265	4.22855228	-2.18003528
C	-0.39397314	6.24341223	-0.02268280
H	-0.30321900	6.12987883	2.13776369
H	-0.29376909	6.13145524	-2.18314250
H	-1.10529034	7.08205529	-0.02415871

M06-L/def2-SVP-gas//B3LYP-D3/def2-SVP-CPCM(toluene)

HF = -3951.487773

B3LYP-D3/def2-TZVPP-gas//B3LYP-D3/def2-SVP-CPCM(toluene)

HF = -3954.495183

TS-III'-IV *cis (R,R,S)*

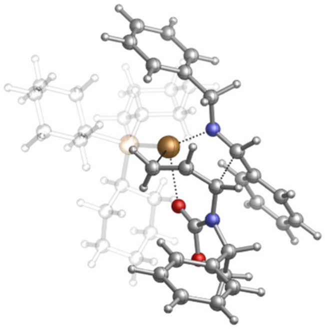

B3LYP-D3/def2-SVP-CPCM(toluene)

Zero-point correction= 0.937494 (Hartree/Particle)

Thermal correction to Energy= 0.985041

Thermal correction to Enthalpy= 0.985985

Thermal correction to Gibbs Free Energy= 0.853692

Sum of electronic and zero-point Energies= -3951.036531

Sum of electronic and thermal Energies= -3950.988984

Sum of electronic and thermal Enthalpies= -3950.988040

Sum of electronic and thermal Free Energies= -3951.120333

O	-1.94214900	5.68924800	2.43525700
C	-1.13003200	5.86515500	1.26458800
C	-2.00824900	5.32261400	0.12812900
H	-0.22065300	5.25350500	1.37290000
H	-0.85176800	6.92000500	1.18653900
N	-2.77525000	4.31822200	0.89198700
C	-2.76142400	4.62780900	2.21367400
O	-3.36411400	4.09577300	3.13237200
C	-3.35353100	3.14351800	0.29760800
H	-2.86151400	2.92188800	-0.65222100
C	-4.75801200	2.88787400	0.36178900
H	-5.07580100	2.08832700	-0.31943600
C	-5.67286000	3.32627500	1.27732800
H	-6.69948900	2.95729000	1.24266900
H	-5.47249400	4.16859700	1.93874600
Cu	-4.52591500	2.14423900	3.43323600
P	-5.52410400	2.46763800	5.39791000
C	-5.35166800	4.21286000	6.06008400
C	-7.39070300	2.18863500	5.38312300
C	-4.84737900	1.32864400	6.71941200
C	-5.96084900	5.20219100	5.04352300
C	-3.87388900	4.55390600	6.34475100
H	-5.92412400	4.27404100	7.00401100

C	-7.82685000	0.80044200	5.88715000
C	-7.91071600	2.40666200	3.94589300
H	-7.83494400	2.95446900	6.04495200
C	-5.18435100	1.69698600	8.17405200
C	-3.33770800	1.06908800	6.53813000
H	-5.34184800	0.37124500	6.48314800
C	-5.73594700	6.66749500	5.43711900
H	-5.49276400	5.01474500	4.06374200
H	-7.04032800	5.01596200	4.92947700
C	-3.67804800	6.02703400	6.72774500
H	-3.28306600	4.33480400	5.44204700
H	-3.48665100	3.91678600	7.15311400
C	-9.34502000	0.60044000	5.77227500
H	-7.31882400	0.02382800	5.29306000
H	-7.52370700	0.64903500	6.93366000
C	-9.42744400	2.21689000	3.83992500
H	-7.39979800	1.68365700	3.28586700
H	-7.62254000	3.40095800	3.57481200
C	-4.68363100	0.61638400	9.14392400
H	-4.70708200	2.65721100	8.43332000
H	-6.26914600	1.84783800	8.29835600
C	-2.84384600	-0.00320700	7.51724200
H	-2.76610700	1.99765900	6.69843200
H	-3.14736800	0.76345300	5.49799600
C	-4.25056300	6.96559600	5.66168200
H	-6.15220000	7.32566500	4.65561100
H	-6.29508200	6.88884700	6.36558300

H	-2.60409400	6.22556600	6.88385000
H	-4.17867300	6.22670900	7.69398900
C	-9.84693400	0.83243900	4.34361000
H	-9.60752700	-0.41558800	6.11319200
H	-9.85665000	1.30352800	6.45570300
H	-9.74892000	2.36417800	2.79493400
H	-9.93687900	2.99515000	4.43832600
C	-3.18139600	0.35963700	8.96894700
H	-4.90673300	0.90894200	10.18390700
H	-5.23732600	-0.32182600	8.95457200
H	-1.75742500	-0.15117800	7.39756500
H	-3.32233600	-0.96711400	7.26516200
H	-4.10786800	8.01934300	5.95577300
H	-3.69975500	6.81719400	4.71549000
H	-10.94270400	0.71490900	4.29523100
H	-9.41355200	0.06177500	3.68072000
H	-2.84595400	-0.43766500	9.65339400
H	-2.62449100	1.27221400	9.25228700
C	-2.64096300	1.34309400	1.38544600
N	-3.58484300	0.90409100	2.22601700
C	-5.18078700	-0.91531900	2.76476700
C	-4.49029200	-1.34015900	3.91135600
C	-6.52440600	-1.28892700	2.61821200
C	-5.12328400	-2.12368800	4.87924100
H	-3.44863900	-1.03810100	4.03553700
C	-7.16102600	-2.08068800	3.58112800
H	-7.08202100	-0.95108900	1.73967200

C	-6.46217600	-2.50140700	4.71637500
H	-4.56920800	-2.44436400	5.76535900
H	-8.21065900	-2.35663800	3.45017800
H	-6.95915300	-3.11151600	5.47491300
C	-4.47393600	-0.11541200	1.68837900
H	-5.23884300	0.32002900	1.01758200
H	-3.90094200	-0.82958600	1.05865100
H	-2.51712100	0.78301300	0.44400100
C	-1.35239600	1.90365900	1.87093100
C	-0.32660900	2.17913500	0.94597400
C	-1.10024800	2.15772900	3.23007800
C	0.89886500	2.70466000	1.35864700
H	-0.50075200	1.98215500	-0.11614600
C	0.12129700	2.69198400	3.64579700
H	-1.88208100	1.94746500	3.95920800
C	1.12745700	2.97263000	2.71479800
H	1.67904400	2.90869700	0.62020900
H	0.28774500	2.89100000	4.70794200
H	2.08270100	3.39129200	3.04170100
C	-2.89997700	6.34100000	-0.59197600
C	-2.66179900	7.72265400	-0.53723300
C	-3.98226400	5.88952600	-1.36961300
C	-3.48105800	8.62604900	-1.22480500
H	-1.82961500	8.12302700	0.04276700
C	-4.80204300	6.78839300	-2.05455200
H	-4.19787800	4.82329100	-1.42988900
C	-4.55643900	8.16402100	-1.98542200

H	-3.27312200	9.69719400	-1.15988100
H	-5.63980000	6.40950800	-2.64570600
H	-5.19803000	8.86826500	-2.52103900
H	-1.38403000	4.79992800	-0.61495800

M06-L/def2-SVP-gas//B3LYP-D3/def2-SVP-CPCM(toluene)

HF = -3951.470792

B3LYP-D3/def2-TZVPP-gas//B3LYP-D3/def2-SVP-CPCM(toluene)

HF = -3954.466648

TS-III'-IV *cis (S,R,S)*

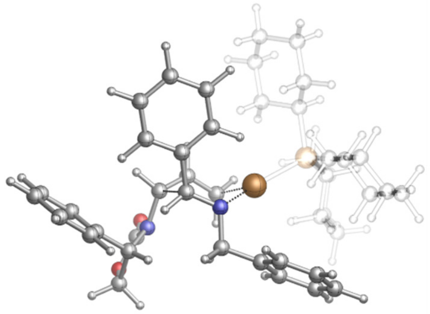

B3LYP-D3/def2-SVP-CPCM(toluene)

Zero-point correction= 0.937558 (Hartree/Particle)

Thermal correction to Energy= 0.985153

Thermal correction to Enthalpy= 0.986098

Thermal correction to Gibbs Free Energy= 0.853680

Sum of electronic and zero-point Energies= -3951.027701

Sum of electronic and thermal Energies= -3950.980106

Sum of electronic and thermal Enthalpies= -3950.979162

Sum of electronic and thermal Free Energies= -3951.111579

O	-4.65723700	3.92176800	-3.30627200
C	-3.61531000	2.94783000	-3.31054400
C	-4.34299600	1.58765300	-3.36355000

H	-3.02550600	3.04457600	-2.38360600
H	-2.96180000	3.11999400	-4.17647800
N	-5.64424100	1.96911300	-2.81202500
C	-5.84285800	3.33192600	-2.97026300
O	-6.86197900	3.96328900	-2.85662100
C	-6.76254000	1.05317700	-2.75661000
H	-7.11143700	0.76074300	-3.75444900
C	-7.79315600	1.31238800	-1.80916300
H	-8.77192900	0.89799800	-2.07533500
C	-7.64813000	1.83323100	-0.54326700
H	-8.53590600	2.08716400	0.03915700
H	-6.72557300	2.35354500	-0.26571300
Cu	-7.30035500	-0.20414300	0.47739300
P	-8.76016800	-0.61245400	2.11705200
C	-9.06365700	0.86042100	3.24788200
C	-10.46322400	-1.07648000	1.45700400
C	-8.29206500	-2.02106300	3.27271000
C	-10.15213800	1.84486700	2.77914900
C	-7.73329600	1.61703300	3.44304500
H	-9.39089600	0.44518800	4.21805100
C	-10.67390700	-2.57882900	1.18724700
C	-10.74408800	-0.28009600	0.16432700
H	-11.18917500	-0.77603000	2.23384000
C	-7.41782000	-1.61564000	4.47406400
C	-7.56553400	-3.11920500	2.46697000
H	-9.24051700	-2.42612400	3.66976900
C	-10.30570000	3.00959800	3.76875500

H	-9.87745700	2.25490400	1.79245300
H	-11.12087700	1.33800700	2.65598900
C	-7.87595200	2.79039800	4.41880000
H	-7.40605500	1.98843100	2.45545300
H	-6.93926100	0.93905100	3.78156400
C	-12.08367600	-2.85552900	0.64348300
H	-9.93339700	-2.92336700	0.44675500
H	-10.51209200	-3.17225300	2.09923200
C	-12.15027200	-0.55076800	-0.38285600
H	-9.99155400	-0.57439800	-0.58791700
H	-10.60651200	0.79787600	0.32419800
C	-7.09757300	-2.83013700	5.35853100
H	-6.47324700	-1.18261200	4.10614600
H	-7.91234600	-0.84458200	5.08378200
C	-7.24466200	-4.34084400	3.33658600
H	-6.63201300	-2.68528700	2.06745500
H	-8.15340500	-3.42797300	1.59107700
C	-8.98234300	3.75410900	3.97749800
H	-11.08526700	3.70110200	3.40777000
H	-10.66001500	2.61455100	4.73882400
H	-6.91233000	3.31986000	4.50776000
H	-8.11237000	2.39694500	5.42488000
C	-12.37554000	-2.04637200	-0.62494000
H	-12.19729700	-3.93519500	0.44806300
H	-12.82629600	-2.59717900	1.42083800
H	-12.30173600	0.01957700	-1.31436800
H	-12.89892900	-0.17779300	0.34054300

C	-6.41626100	-3.95024700	4.56493400
H	-6.45952200	-2.51383900	6.20114200
H	-8.03609200	-3.21260600	5.80108000
H	-6.71057400	-5.09574200	2.73537800
H	-8.19042600	-4.81140000	3.66403000
H	-9.10821700	4.56478000	4.71451900
H	-8.68493300	4.23425600	3.02716800
H	-13.40553300	-2.23312700	-0.97189400
H	-11.70259000	-2.38382600	-1.43342500
H	-6.24124000	-4.82777900	5.20991100
H	-5.42576000	-3.59798300	4.22970000
C	-6.01189600	-0.79814300	-2.02702600
N	-5.81589200	-0.71793000	-0.69771100
C	-7.04119700	-1.71866300	-2.59333900
C	-7.07822300	-1.93352100	-3.98611100
C	-7.97557900	-2.39890200	-1.79555300
C	-8.02865600	-2.77878700	-4.55955300
H	-6.34982300	-1.42516500	-4.62424500
C	-8.92697100	-3.24908900	-2.36831700
H	-7.93942100	-2.27402800	-0.71137500
C	-8.96461000	-3.43916000	-3.75289600
H	-8.03798400	-2.92593200	-5.64288400
H	-9.63976600	-3.77391800	-1.72698300
H	-9.71038700	-4.10154400	-4.19971700
H	-5.11588000	-0.76948000	-2.66103500
C	-4.54717400	-0.12268200	-0.28916300
H	-4.53518200	0.96704000	-0.48541600

H	-3.71132100	-0.55749300	-0.87403600
C	-4.26880100	-0.34923500	1.18012400
C	-3.85270300	-1.60967300	1.63786000
C	-4.44414800	0.68024900	2.11505100
C	-3.59361200	-1.82812500	2.99267900
H	-3.72785800	-2.42285300	0.91749300
C	-4.19314000	0.46487300	3.47546500
H	-4.78441700	1.66108900	1.77185000
C	-3.76278500	-0.78930800	3.91718500
H	-3.25525600	-2.81078500	3.33105000
H	-4.33536500	1.27935400	4.19040800
H	-3.56434000	-0.96123000	4.97813200
C	-4.43287100	0.95827000	-4.74552500
C	-3.69428400	-0.19592400	-5.03920500
C	-5.25935200	1.50435400	-5.74023400
C	-3.77800000	-0.79890400	-6.29878500
H	-3.05519800	-0.63705200	-4.26871000
C	-5.34746300	0.90351900	-6.99830400
H	-5.85417900	2.39612100	-5.52795400
C	-4.60848200	-0.25161900	-7.28104000
H	-3.20064300	-1.70282900	-6.50858200
H	-6.00098400	1.33544100	-7.76038200
H	-4.68367800	-0.72455800	-8.26339200
H	-3.82996300	0.88487600	-2.69652000

TS-III'-IV *trans (R,R,S)*

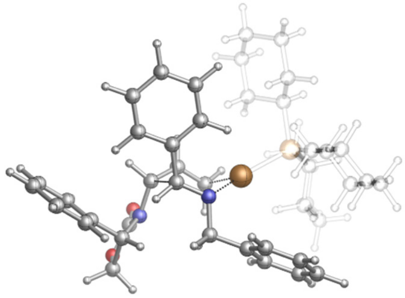

B3LYP-D3/def2-SVP-CPCM(toluene)

Zero-point correction= 0.936337 (Hartree/Particle)

Thermal correction to Energy= 0.984301

Thermal correction to Enthalpy= 0.985245

Thermal correction to Gibbs Free Energy= 0.850080

Sum of electronic and zero-point Energies= -3951.035706

Sum of electronic and thermal Energies= -3950.987742

Sum of electronic and thermal Enthalpies= -3950.986798

Sum of electronic and thermal Free Energies= -3951.121963

O	-0.00147600	6.73280500	0.15896400
C	-0.00018700	5.97968700	-1.05236400
C	-1.31715400	5.18387000	-1.01762800
H	0.88070300	5.31736900	-1.06584300
H	0.05840300	6.66504500	-1.90872500
N	-1.56635200	5.16618100	0.42863900
C	-0.88126600	6.17675800	1.05222900
O	-0.99458600	6.59109400	2.18109100
C	-2.68714300	4.44895900	0.96702300
H	-3.17273000	3.88223400	0.16848600
C	-3.57941100	5.05175200	1.90417200
H	-3.17776900	5.85564100	2.52640000
C	-4.82955500	4.54340300	2.19785300

H	-5.33603900	3.87734400	1.48737900
H	-5.48249000	5.09560900	2.87786500
Cu	-4.32672100	2.79355300	3.58406500
P	-5.44442800	2.54641600	5.49058000
C	-5.38831300	4.01892100	6.64529100
C	-7.28703200	2.24121200	5.24207000
C	-4.79586000	1.08575300	6.46327100
C	-6.13752100	5.22767700	6.05018000
C	-3.93061700	4.40478900	6.96382100
H	-5.89545700	3.72169500	7.58067500
C	-7.69607500	0.75717600	5.27049100
C	-7.71310300	2.87723500	3.90010400
H	-7.81130200	2.75785500	6.06623900
C	-5.21317700	1.01101000	7.94050500
C	-3.27268300	0.90205000	6.30513700
H	-5.25332300	0.23476600	5.93055900
C	-6.04559800	6.46096400	6.95916900
H	-5.69604500	5.46583400	5.06662500
H	-7.19598300	4.98277800	5.87334900
C	-3.84389300	5.63876000	7.87067200
H	-3.40966200	4.61804900	6.01420200
H	-3.39831900	3.56354700	7.43146100
C	-9.19453800	0.57646500	4.98735400
H	-7.11905000	0.20596800	4.51023400
H	-7.45634600	0.30400900	6.24408700
C	-9.21045300	2.70034000	3.62665100
H	-7.12989600	2.39668300	3.09359700

H	-7.44376200	3.94337800	3.87127600
C	-4.75468900	-0.31348700	8.56959200
H	-4.75367800	1.84865000	8.49400900
H	-6.30433800	1.12726400	8.04842600
C	-2.82285300	-0.42403900	6.93036000
H	-2.73419000	1.73116800	6.79395300
H	-3.00207500	0.94763700	5.23908200
C	-4.59078500	6.83089800	7.26493300
H	-6.57058700	7.30818900	6.48704500
H	-6.57509900	6.25113500	7.90691200
H	-2.78555300	5.89306600	8.04791900
H	-4.28012300	5.39599400	8.85723300
C	-9.60806600	1.22136100	3.66056600
H	-9.43992700	-0.49911400	4.98634800
H	-9.77678700	1.03104000	5.81037100
H	-9.46649900	3.14939100	2.65227900
H	-9.78839400	3.25404400	4.38984100
C	-3.24402900	-0.52266400	8.40245900
H	-5.03221100	-0.34088300	9.63680400
H	-5.29394300	-1.14672300	8.08235000
H	-1.73003000	-0.53478500	6.83276400
H	-3.27582200	-1.25922200	6.36424500
H	-4.54710100	7.70025700	7.94199700
H	-4.08855300	7.13367800	6.32778500
H	-10.69277300	1.10618900	3.49728400
H	-9.10228200	0.69402700	2.83205600
H	-2.94342700	-1.49581600	8.82562700

H	-2.70722900	0.25108600	8.98227600
C	-1.98657500	2.74205000	1.89818800
N	-3.05107900	2.01919100	2.31664500
C	-4.66211800	0.21548900	1.84638700
C	-4.25479300	-0.60642400	2.91147600
C	-5.95073100	0.04008100	1.32346800
C	-5.10644200	-1.58484800	3.42820700
H	-3.25946900	-0.45929800	3.33506900
C	-6.80677600	-0.94387000	1.83408200
H	-6.29003600	0.68381500	0.50653100
C	-6.38669400	-1.76137200	2.88701200
H	-4.77133200	-2.21301600	4.25816200
H	-7.80785500	-1.06544500	1.41221700
H	-7.05631400	-2.52408600	3.29257400
C	-3.71046800	1.24894200	1.27638800
H	-4.27499400	1.89665200	0.57055800
H	-2.96001700	0.72555900	0.64591900
H	-1.49064900	2.39114600	0.98054900
C	-1.01955000	3.28908100	2.88522800
C	0.29815100	3.57842800	2.48481600
C	-1.36949800	3.55874400	4.21997600
C	1.21433900	4.15198300	3.36871600
H	0.60430400	3.35593800	1.46164900
C	-0.45805000	4.12810200	5.10867200
H	-2.37265300	3.30964200	4.57173800
C	0.83972000	4.43776100	4.68523400
H	2.22780000	4.37813500	3.02654600

H	-0.76468300	4.33207200	6.13850500
H	1.55392000	4.89132000	5.37723000
H	-1.29394667	4.21324422	-1.46734682
C	-2.43460867	5.99423241	-1.70042212
C	-2.42437243	7.38757513	-1.63686562
C	-3.45756227	5.33453118	-2.38216262
C	-3.43695067	8.12151613	-2.25532122
H	-1.61832261	7.90759502	-1.09927299
C	-4.46967241	6.06824640	-3.00089687
H	-3.46559709	4.23591846	-2.43175848
C	-4.45912711	7.46220316	-2.93773126
H	-3.42817593	9.22001563	-2.20568609
H	-5.27575713	5.54862000	-3.53896022
H	-5.25694440	8.04065744	-3.42576275

M06-L/def2-SVP-gas//B3LYP-D3/def2-SVP-CPCM(toluene)

HF = -3951.468797

B3LYP-D3/def2-TZVPP-gas//B3LYP-D3/def2-SVP-CPCM(toluene)

HF = -3954.465382

TS-III'-IV *trans (S,S,S)*

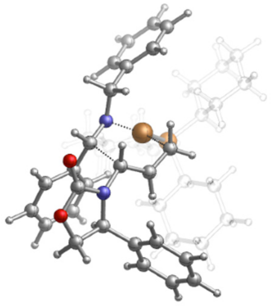

B3LYP-D3/def2-SVP-CPCM(toluene)

Zero-point correction= 0.937435 (Hartree/Particle)

Thermal correction to Energy= 0.985177

Thermal correction to Enthalpy= 0.986121

Thermal correction to Gibbs Free Energy= 0.852879

Sum of electronic and zero-point Energies= -3951.041933

Sum of electronic and thermal Energies= -3950.994191

Sum of electronic and thermal Enthalpies= -3950.993247

Sum of electronic and thermal Free Energies= -3951.126489

O	0.48942800	5.81398900	-0.14737200
C	0.37704400	6.61785200	-1.32104400
C	-0.94905800	6.18968600	-2.01087900
H	0.35299000	7.67814000	-1.02564200
H	1.24910600	6.43858700	-1.96527000
H	-0.73156800	5.59166300	-2.90943900
N	-1.52865100	5.33764800	-0.97850000
C	-0.64375900	5.08291500	0.03578700
O	-0.78867800	4.32149300	0.96570100
C	-2.68393500	4.50214600	-1.14596000
H	-3.04194700	4.16739300	-0.16891600
C	-3.68660900	4.87690000	-2.09039300
H	-3.40230500	5.55285300	-2.90114600
C	-4.93316000	4.28767400	-2.17071800
H	-5.33672300	3.72810600	-1.31937700
H	-5.67142500	4.69720700	-2.86249200
Cu	-4.37974100	2.47702000	-3.45588700
P	-5.38968000	2.39892900	-5.44248600
C	-5.25538100	4.02047800	-6.38531900
C	-7.23252600	2.03320900	-5.35575700

C	-4.67362200	1.08942700	-6.57270400
C	-6.26219900	5.08844800	-5.91598900
C	-3.81838000	4.56785500	-6.26934500
H	-5.47161300	3.79848300	-7.44462600
C	-7.57317000	0.53375700	-5.45175000
C	-7.79467600	2.61106500	-4.03761000
H	-7.70915900	2.54818300	-6.20903700
C	-5.09424000	1.16482100	-8.04938800
C	-3.14417800	0.95948800	-6.43914000
H	-5.09213800	0.16384600	-6.14136200
C	-6.08169300	6.40686500	-6.68135300
H	-6.11069200	5.28119200	-4.84044000
H	-7.29655700	4.73257600	-6.03525000
C	-3.63422300	5.89616100	-7.01250100
H	-3.59227100	4.71618600	-5.19945000
H	-3.09096100	3.83063000	-6.63979500
C	-9.08225400	0.28722500	-5.30676000
H	-7.03806700	-0.00952800	-4.65496800
H	-7.23414200	0.11440900	-6.41068000
C	-9.30263800	2.37087800	-3.90174100
H	-7.26386200	2.12836100	-3.19710900
H	-7.57805300	3.68567600	-3.95627100
C	-4.57621700	-0.05252500	-8.83019800
H	-4.67573800	2.08136900	-8.50141500
H	-6.19012100	1.24126000	-8.14414300
C	-2.63000600	-0.25667700	-7.22003800
H	-2.65163700	1.86724400	-6.82753000

H	-2.86829900	0.88562100	-5.37541200
C	-4.65255100	6.94175100	-6.54862700
H	-6.80863800	7.14980600	-6.31278100
H	-6.31450600	6.24109500	-7.74947600
H	-2.60521900	6.26287900	-6.85916900
H	-3.75095800	5.72586800	-8.09862800
C	-9.64034900	0.88104400	-4.00972900
H	-9.28232900	-0.79667000	-5.35212200
H	-9.60536600	0.73951400	-6.16980400
H	-9.65806100	2.78031400	-2.94125500
H	-9.83269600	2.92781300	-4.69674700
C	-3.05573600	-0.20122000	-8.69272000
H	-4.86169500	0.03184300	-9.89222200
H	-5.06868600	-0.96355700	-8.44321500
H	-1.53222400	-0.31883800	-7.13653300
H	-3.03340200	-1.17666900	-6.75805400
H	-4.53415800	7.87716100	-7.12098600
H	-4.45458400	7.19416900	-5.49174900
H	-10.73064400	0.72447500	-3.95257900
H	-9.19485600	0.35023600	-3.15086800
H	-2.70847100	-1.10051500	-9.22832200
H	-2.56416800	0.66259900	-9.17782300
C	-1.79729200	7.37632700	-2.42799500
C	-2.55503400	8.09525500	-1.49279400
C	-1.80543400	7.78135600	-3.76913800
C	-3.31101600	9.19894500	-1.89448100
H	-2.56430300	7.77058900	-0.44924400

C	-2.55542400	8.89137700	-4.17263000
H	-1.22967200	7.21357500	-4.50596800
C	-3.31248300	9.60152700	-3.23590100
H	-3.90632700	9.74613900	-1.15895500
H	-2.55606800	9.19383800	-5.22264000
H	-3.90648600	10.46361800	-3.54970000
C	-2.06049200	2.61589500	-1.71585100
N	-3.12873100	1.86864000	-2.07817200
C	-4.90779000	0.28636100	-1.45161900
C	-4.63512500	-0.64933500	-2.46566900
C	-6.19543700	0.31536000	-0.89862600
C	-5.61758500	-1.54209400	-2.89880600
H	-3.63994500	-0.66037800	-2.91558700
C	-7.18318100	-0.57918700	-1.32876300
H	-6.42818700	1.04837600	-0.12079200
C	-6.89649200	-1.51314100	-2.32795500
H	-5.38690800	-2.26376700	-3.68715500
H	-8.18118200	-0.54103400	-0.88451000
H	-7.66803000	-2.20803300	-2.66924800
C	-3.81952700	1.22445900	-0.97020400
H	-4.26896700	1.96205400	-0.27186700
H	-3.10103300	0.64231900	-0.35558600
H	-1.63340800	2.40359200	-0.72467100
C	-1.00701000	2.95412400	-2.70824200
C	0.31841500	3.14957100	-2.27322700
C	-1.28676600	3.15652800	-4.07109500
C	1.31230300	3.57761400	-3.15847100

H	0.56869300	2.97369800	-1.22422000
C	-0.29793900	3.57925600	-4.95934300
H	-2.30173200	2.99460000	-4.43300600
C	1.00811700	3.80685100	-4.50510700
H	2.33006600	3.73504300	-2.79147200
H	-0.54962500	3.74082000	-6.01131700
H	1.78241400	4.14849900	-5.19660800

M06-L/def2-SVP-gas//B3LYP-D3/def2-SVP-CPCM(toluene)

HF = -3951.475837

B3LYP-D3/def2-TZVPP-gas//B3LYP-D3/def2-SVP-CPCM(toluene)

HF = -3954.472808

III' *trans*

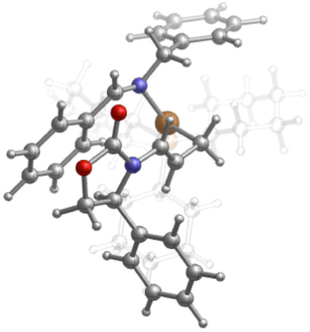

B3LYP-D3/def2-SVP-CPCM(toluene)

Zero-point correction= 0.937256 (Hartree/Particle)

Thermal correction to Energy= 0.985784

Thermal correction to Enthalpy= 0.986728

Thermal correction to Gibbs Free Energy= 0.852690

Sum of electronic and zero-point Energies= -3951.053687

Sum of electronic and thermal Energies= -3951.005159

Sum of electronic and thermal Enthalpies= -3951.004215

Sum of electronic and thermal Free Energies= -3951.138254

O	4.33438000	2.50194100	3.57672200
C	4.22497100	3.29491400	2.39660700
C	2.90128100	2.85886500	1.70421000
H	4.19825800	4.35737600	2.68320400
H	5.10018500	3.11260700	1.75721000
H	3.12913000	2.28037900	0.79702500
N	2.33138400	1.98324800	2.72830100
C	3.21140000	1.75595100	3.75454700
O	3.07260700	1.00272000	4.69073600
C	1.21387400	1.05381700	2.53038800
H	0.79403700	0.86026000	3.52652500
C	0.16064300	1.57505200	1.63176800
H	0.45035800	2.24873500	0.82292700
C	-1.11127200	1.11674600	1.65767600
H	-1.47549400	0.48158300	2.47072400
H	-1.86016700	1.50074200	0.96359600
Cu	-0.53468200	-0.86362900	0.28298500
P	-1.56567600	-0.90864300	-1.67885500
C	-1.46459800	0.71409100	-2.61746600
C	-3.40486000	-1.29433100	-1.60924300
C	-0.81027600	-2.20529000	-2.80114900
C	-2.42912900	1.78339200	-2.06911600
C	-0.01718800	1.24361600	-2.58554400
H	-1.75196000	0.50537300	-3.66242900
C	-3.75757600	-2.78021100	-1.80835800
C	-3.95696700	-0.80069400	-0.25339100

H	-3.88352000	-0.72095400	-2.42318600
C	-1.14020800	-2.07333300	-4.29681500
C	0.70534600	-2.38273800	-2.58197500
H	-1.27656300	-3.13194700	-2.42551100
C	-2.27881600	3.11523000	-2.81713500
H	-2.21288500	1.95001800	-1.00044200
H	-3.47298700	1.44148500	-2.13427800
C	0.13550500	2.58403200	-3.31361400
H	0.27687900	1.37274200	-1.53000700
H	0.67605700	0.50433600	-3.01385000
C	-5.27009300	-3.01704000	-1.68122000
H	-3.23050500	-3.38276600	-1.04942700
H	-3.42088400	-3.13394800	-2.79444200
C	-5.46699900	-1.03409500	-0.13032400
H	-3.42894000	-1.34345500	0.55132300
H	-3.72697000	0.26466600	-0.10337800
C	-0.60996200	-3.28003600	-5.08560500
H	-0.67021900	-1.15595300	-4.69315300
H	-2.22625400	-1.96153500	-4.45257700
C	1.23099200	-3.58837900	-3.37192900
H	1.24555300	-1.47803100	-2.90844700
H	0.91734700	-2.49724400	-1.50727100
C	-0.83747400	3.63030700	-2.76238300
H	-2.97231300	3.85879800	-2.39008900
H	-2.57862700	2.97384500	-3.87192600
H	1.17684200	2.93595400	-3.22040500
H	-0.05378000	2.43756900	-4.39289200

C	-5.82126700	-2.50900100	-0.34491600
H	-5.48504700	-4.09214000	-1.80291600
H	-5.78644700	-2.49810800	-2.51016400
H	-5.81624900	-0.69212300	0.85840000
H	-5.99292900	-0.41568600	-0.88142900
C	0.89525000	-3.47553300	-4.86449600
H	-0.83001000	-3.15407400	-6.15901600
H	-1.14834000	-4.18838700	-4.75809800
H	2.31973600	-3.68481500	-3.22636100
H	0.77623500	-4.51028200	-2.96466800
H	-0.74286600	4.57801100	-3.31857700
H	-0.57032200	3.85464300	-1.71441900
H	-6.91343300	-2.65579000	-0.29913100
H	-5.38313700	-3.10457100	0.47432300
H	1.24985900	-4.36730700	-5.40779400
H	1.43724700	-2.61132500	-5.29173800
C	2.05314000	4.05225100	1.30090100
C	1.31890200	4.78215700	2.24673600
C	2.03440300	4.46187400	-0.03918300
C	0.57595000	5.89910900	1.85676600
H	1.31822000	4.45638700	3.29006100
C	1.29639300	5.58429800	-0.43135100
H	2.59250600	3.88779900	-0.78476400
C	0.56279900	6.30447900	0.51605400
H	0.00029300	6.45511700	2.60128200
H	1.28693200	5.88850600	-1.48083800
H	-0.02110800	7.17681700	0.21182000

C	1.74287800	-0.48313400	2.06110500
N	0.69560200	-1.35800100	1.68558100
C	-1.05314000	-2.95584400	2.33263400
C	-0.81222800	-3.90319500	1.32012900
C	-2.34257900	-2.88744400	2.88062600
C	-1.82254100	-4.76553200	0.88727600
H	0.18253200	-3.94557500	0.87096500
C	-3.35976700	-3.74861600	2.44925800
H	-2.55401600	-2.14586600	3.65662700
C	-3.10232300	-4.69409100	1.45321800
H	-1.61287500	-5.49697500	0.10185200
H	-4.35761700	-3.67418700	2.88968700
H	-3.89501200	-5.36447700	1.11112500
C	0.05792400	-2.03020200	2.79958300
H	-0.38183600	-1.32993500	3.54955200
H	0.79357000	-2.63339700	3.38068900
H	2.23467400	-0.81852400	2.99119700
C	2.82913700	-0.28655500	1.02193200
C	4.17061400	-0.16172600	1.42332300
C	2.53200200	-0.09101800	-0.33450800
C	5.16551000	0.21319300	0.51409600
H	4.43453400	-0.33190700	2.47020600
C	3.51971600	0.27424500	-1.25173000
H	1.50303900	-0.21061000	-0.67315000
C	4.84231500	0.44879100	-0.82688600
H	6.19719200	0.32665300	0.85781500
H	3.25222100	0.43547700	-2.29987100

H 5.61591300 0.75234400 -1.53685900

M06-L/def2-SVP-gas//B3LYP-D3/def2-SVP-CPCM(toluene)

HF = -3951.490381

B3LYP-D3/def2-TZVPP-gas//B3LYP-D3/def2-SVP-CPCM(toluene)

HF = -3954.486666

4

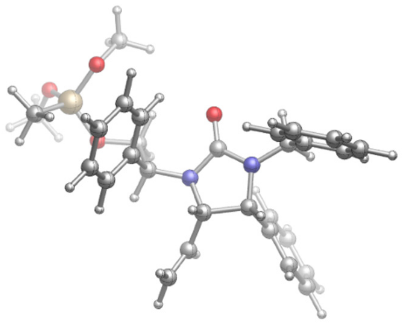

B3LYP-D3/def2-SVP-CPCM(toluene)

Zero-point correction= 0.578558 (Hartree/Particle)

Thermal correction to Energy= 0.614834

Thermal correction to Enthalpy= 0.615778

Thermal correction to Gibbs Free Energy= 0.502194

Sum of electronic and zero-point Energies= -1823.915293

Sum of electronic and thermal Energies= -1823.879018

Sum of electronic and thermal Enthalpies= -1823.878074

Sum of electronic and thermal Free Energies= -1823.991658

C 0.51669000 0.10030600 -1.75597000

H 0.41936900 0.08123900 -0.65366300

C 1.39440200 -1.12279100 -2.18142000

H 1.30972400 -1.90496300 -1.41115600

N 1.35994000 1.23897400 -2.14877200

N	2.71963800	-0.53036100	-2.15436700
C	2.69232000	0.84275900	-2.19099100
C	1.59983900	3.67794800	-2.57236400
H	2.68815700	3.66845900	-2.43665100
H	1.39385600	3.45750300	-3.63374100
O	1.03585400	4.93008100	-2.23700300
O	3.67572600	1.56860000	-2.25898600
C	-0.85318900	0.15220300	-2.36762800
C	1.04232400	-1.73367600	-3.52983500
C	0.27763200	-2.90635000	-3.58769200
C	1.44091300	-1.12331800	-4.72903400
C	-0.09154500	-3.45815100	-4.81859200
H	-0.03313800	-3.39237900	-2.65848300
C	1.07478000	-1.67307700	-5.96058200
H	2.04313300	-0.21251300	-4.69919800
C	0.30567600	-2.84154400	-6.00908500
H	-0.68662400	-4.37454800	-4.84735400
H	1.39244300	-1.18796500	-6.88711300
H	0.02160900	-3.27232400	-6.97254600
C	4.16410500	-2.19187400	-1.05232000
C	4.01383500	-1.66682500	0.24145700
C	4.53409900	-3.53491700	-1.19903800
C	4.23732200	-2.46768400	1.36370000
H	3.71364100	-0.62243700	0.36421000
C	4.76268600	-4.33906900	-0.07623400
H	4.64110300	-3.95788500	-2.20226400
C	4.61472000	-3.80745700	1.20806000

H	4.11749300	-2.04550700	2.36496200
H	5.04988300	-5.38571300	-0.20686000
H	4.78836600	-4.43480800	2.08611800
C	-1.98491000	0.10272600	-1.66022700
H	-2.96431800	0.13458900	-2.14520100
H	-1.97086900	0.02557400	-0.56773300
H	-0.88187500	0.23907200	-3.45858400
C	0.94614000	2.57940400	-1.71847000
H	-0.13053000	2.63787200	-1.93829200
C	1.11006000	2.79197500	-0.21733200
C	-0.02504600	2.85789800	0.60368100
C	2.37942900	2.91150000	0.37437600
C	0.09557400	3.04596500	1.98463200
H	-1.01738800	2.76767600	0.15280600
C	2.50199700	3.10141600	1.75326300
H	3.27068200	2.86464200	-0.25125100
C	1.36217100	3.17101000	2.56298000
H	-0.80099600	3.10027500	2.60758300
H	3.49531900	3.20487700	2.19747500
H	1.46196500	3.32457300	3.64058600
C	3.93234200	-1.30762900	-2.26426500
H	4.75308900	-0.58130100	-2.37240300
H	3.91042300	-1.92125700	-3.18177100
Si	1.76975900	6.15134600	-1.36317500
O	1.83594500	7.48379800	-2.35752800
O	3.33402900	5.76871500	-0.97921800
C	0.83669700	6.46985700	0.20526100

C	0.88471800	7.85433100	-3.33228300
C	4.45326000	5.79041200	-1.84486100
H	0.96739000	5.62821200	0.90241200
H	1.19106100	7.39306600	0.68963100
H	-0.23957800	6.57624200	-0.00420200
H	1.35804800	8.54105200	-4.05304600
H	0.50062900	6.97868600	-3.88533800
H	0.02242300	8.37903700	-2.87928800
H	4.28955600	6.45066800	-2.71419200
H	5.33129200	6.16085700	-1.29051400
H	4.68361100	4.77432900	-2.21196900

M06-L/def2-SVP-gas//B3LYP-D3/def2-SVP-CPCM(toluene)

HF = -1824.188146

B3LYP-D3/def2-TZVPP-gas//B3LYP-D3/def2-SVP-CPCM(toluene)

HF = -1826.303241

3

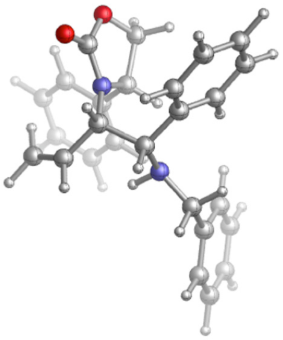

B3LYP-D3/def2-SVP-CPCM(toluene)

Zero-point correction= 0.467196 (Hartree/Particle)

Thermal correction to Energy= 0.492718

Thermal correction to Enthalpy= 0.493662

Thermal correction to Gibbs Free Energy= 0.408055

Sum of electronic and zero-point Energies= -1265.063319

Sum of electronic and thermal Energies= -1265.037797

Sum of electronic and thermal Enthalpies= -1265.036853

Sum of electronic and thermal Free Energies= -1265.122460

C	0.88173700	1.51702900	-1.07466400
O	2.07100700	2.18438000	-1.51826200
C	0.03727100	2.62503000	-0.41415800
H	0.39050200	1.05608500	-1.94045700
H	1.15885900	0.73819300	-0.34522200
H	-0.46379200	2.24016400	0.47854200
C	2.25767600	3.30315800	-0.76552200
O	3.28075800	3.94717800	-0.79436800
C	-1.01120900	3.21220400	-1.34399900
C	-2.34813000	3.28389700	-0.93495700
C	-0.66256100	3.68239900	-2.62048300
C	-3.32283100	3.82935800	-1.77737800
H	-2.62458700	2.92490300	0.06050500
C	-1.63274600	4.23089000	-3.46221300
H	0.37635000	3.62431700	-2.95527700
C	-2.96629500	4.30892200	-3.04114600
H	-4.36178600	3.88559700	-1.44244400
H	-1.34861200	4.59849700	-4.45155900
H	-3.72475300	4.74031400	-3.69932300
N	1.11908900	3.55417100	-0.03852600
C	1.17684000	4.44324300	1.11401400
H	2.09355200	5.03024000	0.94859900

C	0.06728800	5.46803200	1.20873100
H	-0.10578100	5.85059200	2.21981400
C	1.41925400	3.69320600	2.47338300
C	-0.61306200	6.00848100	0.19631600
H	-1.35029400	6.79521400	0.37771900
H	-0.46603300	5.69334600	-0.83752600
N	0.21323957	2.98675295	2.88770834
H	-0.55786949	3.65034793	2.92277826
C	0.26745868	2.23026165	4.14388398
H	1.08064328	1.49538283	4.02832205
H	0.57688341	2.90282548	4.95686590
C	-1.01701166	1.51270767	4.52879087
C	-1.71869201	1.86305435	5.69098529
C	-1.55484088	0.51689332	3.69652645
C	-2.92498458	1.23307156	6.01994905
H	-1.31396648	2.63820471	6.34856198
C	-2.75871850	-0.11411502	4.01820379
H	-1.02289920	0.23494722	2.78230176
C	-3.44889498	0.24343752	5.18355023
H	-3.45708351	1.51934163	6.93099550
H	-3.16091689	-0.88936226	3.36049364
H	-4.39141530	-0.24888386	5.43659378
H	1.75799864	4.33929580	3.25614736
C	2.56677060	2.71348550	2.16523789
C	3.89043063	3.14978914	2.22101753
C	2.28228450	1.38924421	1.83077932
C	4.92987622	2.26198612	1.94203708

H	4.11474994	4.19342030	2.48494497
C	3.32140824	0.50171918	1.55139036
H	1.23855866	1.04541527	1.78727931
C	4.64560378	0.93836926	1.60670247
H	5.97328368	2.60646669	1.98534769
H	3.09751672	-0.54196658	1.28702513
H	5.46482571	0.23869616	1.38618151

M06-L/def2-SVP-gas//B3LYP-D3/def2-SVP-CPCM(toluene)

HF = -1265.303506

B3LYP-D3/def2-TZVPP-gas//B3LYP-D3/def2-SVP-CPCM(toluene)

HF = -1266.906722

TS-II-III *cis*

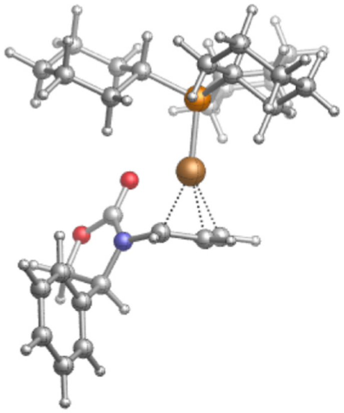

B3LYP-D3/def2-SVP-CPCM(toluene)

Zero-point correction= 0.701987 (Hartree/Particle)

Thermal correction to Energy= 0.736617

Thermal correction to Enthalpy= 0.737561

Thermal correction to Gibbs Free Energy= 0.632092

Sum of electronic and zero-point Energies= -3355.574160

Sum of electronic and thermal Energies= -3355.539530

Sum of electronic and thermal Enthalpies= -3355.538586

Sum of electronic and thermal Free Energies= -3355.644055

C	1.55026500	0.14110700	3.46816300
C	3.39986000	-1.37856100	2.61109600
Cu	1.86252100	0.06687300	1.32954700
H	1.35801700	1.06513100	4.01539900
H	4.46314600	-1.47943600	2.38880500
H	2.77863400	-2.27168100	2.50330200
C	0.44522700	-1.49238700	5.09747800
C	-0.66259100	-2.52785100	4.79894000
H	1.38387700	-1.99715200	5.39129700
H	-1.65913700	-2.11983400	5.03461900
H	-0.52529200	-3.48182400	5.32471000
N	0.60884200	-0.90577400	3.76405700
C	0.10236200	-1.73324300	2.81367000
O	0.23503300	-1.63324800	1.60084100
O	-0.57726000	-2.75689400	3.38332300
C	2.91160700	-0.20688900	3.16743300
H	3.62933100	0.61827000	3.28095100
P	1.33097500	0.74073300	-0.68996300
C	-0.53860500	0.93988000	-0.83338800
C	1.78358800	-0.49107000	-2.03720900
C	2.05993900	2.38158300	-1.23826000
C	-1.29054400	-0.36795900	-1.15480100
C	-1.10455900	1.53519400	0.47515900
H	-0.71631500	1.65098800	-1.66154700
C	3.17636800	-0.29846000	-2.66616000

C	1.68490600	-1.91975400	-1.45784500
H	1.03686800	-0.36759600	-2.84245100
C	1.26661000	3.62186400	-0.78482900
C	3.50883700	2.48498200	-0.71374300
H	2.07113900	2.37486000	-2.34320900
C	-2.80454500	-0.12912200	-1.25404600
H	-1.09074200	-1.09290200	-0.34825800
H	-0.93325600	-0.81107400	-2.09621500
C	-2.61607900	1.77758400	0.38510000
H	-0.88744700	0.82203800	1.28763700
H	-0.59064700	2.46755800	0.74591200
C	3.44222600	-1.35435100	-3.75003100
H	3.94547000	-0.39256400	-1.87885500
H	3.28290700	0.70693400	-3.10163200
C	1.94399900	-2.98458700	-2.52940900
H	2.43509200	-2.00668900	-0.65098000
H	0.71656200	-2.09134500	-0.97004300
C	1.94330500	4.91965200	-1.25015900
H	1.20412200	3.62505900	0.31795400
H	0.23443800	3.59147600	-1.16494200
C	4.19420800	3.77860400	-1.17018100
H	3.46860900	2.44877000	0.39078700
H	4.10581800	1.61516500	-1.02133100
C	-3.36559700	0.48937400	0.03055500
H	-3.31404300	-1.08157700	-1.47815200
H	-3.01228500	0.54638400	-2.10493600
H	-2.98384300	2.18941300	1.34030500

H	-2.81912800	2.54403200	-0.38617600
C	3.30540300	-2.77993100	-3.20319000
H	4.44709200	-1.20064500	-4.17856300
H	2.72276400	-1.20968200	-4.57714400
H	1.88299700	-3.98971300	-2.07951200
H	1.14559300	-2.93337500	-3.29304200
C	3.39059300	5.01542800	-0.75559900
H	1.35817700	5.78713700	-0.90116400
H	1.93143300	4.95685200	-2.35517700
H	5.21667500	3.82495200	-0.75925800
H	4.29852700	3.76235000	-2.27092600
H	-4.44673100	0.68317400	-0.07243700
H	-3.24894000	-0.23374300	0.85846500
H	3.45453500	-3.51682700	-4.01036800
H	4.10465000	-2.95890500	-2.46058200
H	3.86844900	5.93386100	-1.13592100
H	3.39059900	5.09288500	0.34732300
C	0.06531500	-0.49240400	6.16744800
C	-0.83868200	0.54444900	5.88648900
C	0.58065200	-0.61179800	7.46440600
C	-1.22160600	1.44128100	6.88611400
H	-1.22878500	0.65176800	4.87097000
C	0.19357100	0.28171700	8.46932500
H	1.29474300	-1.40911700	7.68951600
C	-0.70845400	1.31045600	8.18246200
H	-1.92084400	2.24848800	6.65302400
H	0.60344600	0.17671900	9.47713000

H -1.00722900 2.01299900 8.96461500

M06-L/def2-SVP-gas//B3LYP-D3/def2-SVP-CPCM(toluene)

HF = -3355.888342

B3LYP-D3/def2-TZVPP-gas//B3LYP-D3/def2-SVP-CPCM(toluene)

HF = -3358.135889

TS-I-III *trans*

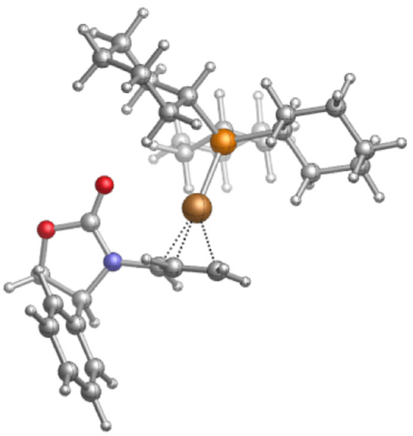

B3LYP-D3/def2-SVP-CPCM(toluene)

Zero-point correction= 0.701895 (Hartree/Particle)

Thermal correction to Energy= 0.736512

Thermal correction to Enthalpy= 0.737456

Thermal correction to Gibbs Free Energy= 0.632980

Sum of electronic and zero-point Energies= -3355.570311

Sum of electronic and thermal Energies= -3355.535694

Sum of electronic and thermal Enthalpies= -3355.534750

Sum of electronic and thermal Free Energies= -3355.639226

C 1.31661600 -0.29458900 3.42918700

C 3.08362900 -1.79419200 2.38305700

Cu 1.73509800 -0.06020800 1.36320800

H 4.14652500 -1.95942100 2.20149800

H	2.39601200	-2.60044700	2.10401900
C	2.66448200	-0.71528400	3.14598200
H	3.44101200	-0.00191900	3.45872500
P	1.25455900	0.63961000	-0.66254900
C	-0.56588000	1.00096900	-0.96293300
C	1.78540800	-0.51675500	-2.04462200
C	2.13959200	2.26900400	-0.99708000
C	-1.39957900	-0.17066400	-1.51361700
C	-1.20155000	1.51352800	0.34717700
H	-0.59426800	1.80650600	-1.71811200
C	3.27809600	-0.39827700	-2.40956700
C	1.48095500	-1.97351400	-1.63179400
H	1.19096800	-0.25351700	-2.93845900
C	1.35074200	3.53741300	-0.61358600
C	3.49461400	2.27252800	-0.25392800
H	2.31986500	2.30049400	-2.08731300
C	-2.86070800	0.25134700	-1.73242000
H	-1.37344600	-1.00827600	-0.79391400
H	-0.98194200	-0.54621900	-2.46019100
C	-2.66015100	1.93683400	0.14045000
H	-1.14691500	0.69862200	1.09165800
H	-0.61607100	2.33560100	0.77994900
C	3.66659300	-1.39152900	-3.51424700
H	3.88111400	-0.60792000	-1.50772600
H	3.52809700	0.62329600	-2.73285900
C	1.86745500	-2.97582200	-2.72643700
H	2.05317100	-2.18462300	-0.70960700

H	0.42192500	-2.10032500	-1.36779000
C	2.15944800	4.80341000	-0.93493800
H	1.14000400	3.51218300	0.46660500
H	0.38613100	3.57983600	-1.14166800
C	4.30871500	3.53501200	-0.56218000
H	3.27920300	2.22953600	0.82783000
H	4.08271700	1.37530300	-0.49457100
C	-3.49562300	0.79405600	-0.44720000
H	-3.44029800	-0.60419800	-2.11834400
H	-2.89546300	1.03232700	-2.51436500
H	-3.08955200	2.27707900	1.09771300
H	-2.69298400	2.80482200	-0.54399700
C	3.34159900	-2.83566200	-3.11886500
H	4.73982600	-1.28616800	-3.74596500
H	3.11868600	-1.13393600	-4.43952000
H	1.65413300	-4.00210100	-2.38325300
H	1.23347400	-2.80396200	-3.61607400
C	3.51667600	4.80213500	-0.22323100
H	1.57594500	5.69492900	-0.64928700
H	2.31941900	4.86883100	-2.02759500
H	5.25874800	3.51078100	-0.00213900
H	4.57631500	3.54423500	-1.63549300
H	-4.52954500	1.12657600	-0.63980300
H	-3.56006300	-0.02283500	0.29518400
H	3.59232500	-3.52711500	-3.94078200
H	3.97262800	-3.12485900	-2.25844000
H	4.09392300	5.70384600	-0.48908000

H	3.34929200	4.83920000	0.86829000
N	1.11846900	0.87580800	4.22499200
C	1.04948300	0.85928600	5.69557800
C	1.25408800	2.15014000	3.75843500
C	0.65852700	2.32728300	5.95686200
H	0.24693300	0.17724000	6.01613500
C	2.36015200	0.41367200	6.31908600
O	1.49902400	2.52434200	2.62786900
O	1.09516300	3.03390400	4.78755400
H	1.15086000	2.76155200	6.83685000
H	-0.43357600	2.44047600	6.05663600
C	3.50335400	1.22643100	6.23059700
C	2.46749100	-0.83984400	6.93280600
C	4.72431500	0.79341500	6.75077700
H	3.43726200	2.20358000	5.74435600
C	3.69063600	-1.27803400	7.45394100
H	1.58637800	-1.48452200	6.99730100
C	4.82153400	-0.46242800	7.36387400
H	5.60544800	1.43588600	6.67589700
H	3.75936300	-2.26089300	7.92724800
H	5.77819000	-0.80271100	7.76837000
H	0.52654200	-1.04759900	3.54257000

M06-L/def2-SVP-gas//B3LYP-D3/def2-SVP-CPCM(toluene)

HF = -3355.882164

B3LYP-D3/def2-TZVPP-gas//B3LYP-D3/def2-SVP-CPCM(toluene)

HF = -3358.131786

TS-I-II conf2

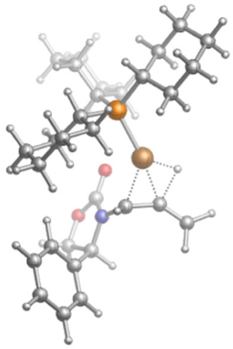

B3LYP-D3/def2-SVP-CPCM(toluene)

Zero-point correction= 0.696120 (Hartree/Particle)

Thermal correction to Energy= 0.731446

Thermal correction to Enthalpy= 0.732390

Thermal correction to Gibbs Free Energy= 0.627176

Sum of electronic and zero-point Energies= -3355.513471

Sum of electronic and thermal Energies= -3355.478146

Sum of electronic and thermal Enthalpies= -3355.477201

Sum of electronic and thermal Free Energies= -3355.582416

O	-1.40146700	-1.15654500	1.60757900
C	-1.14513200	-1.27417000	3.01438200
C	-1.69552100	0.03349000	3.63126900
H	-1.68602400	-2.15253900	3.40249500
H	-0.06753000	-1.41092900	3.17339600
H	-0.89418400	0.78878800	3.72983100
N	-2.62101500	0.44000900	2.57326400
C	-2.34639000	-0.20479100	1.40681000
O	-2.83356900	0.01575600	0.31077300
C	-3.40316900	1.64508500	2.63463600

H	-4.11296900	1.68884800	3.46496200
C	-2.92218400	2.83286800	2.03269800
C	-2.25050200	3.93111600	2.37608500
H	-1.82204500	4.00560000	3.38329700
H	-2.10794000	4.77555500	1.69653200
C	-2.36319200	-0.16142200	4.97588100
C	-3.60993900	-0.80139300	5.06058600
C	-1.73805800	0.26645200	6.15346600
C	-4.21868200	-1.00991400	6.29951900
H	-4.10946800	-1.12103000	4.14348300
C	-2.34247500	0.05230400	7.39766400
H	-0.77214800	0.77641800	6.09730500
C	-3.58405300	-0.58555300	7.47355700
H	-5.19446800	-1.49969200	6.34808400
H	-1.84388900	0.39178200	8.30911100
H	-4.06005800	-0.74711900	8.44400200
P	-6.00239100	1.14380200	-0.21374800
C	-7.26038300	2.33583400	-0.93709200
C	-6.97813100	0.17589100	1.06697600
C	-5.49272800	-0.16055500	-1.44259200
C	-7.16124600	3.68236600	-0.18383400
C	-7.11355200	2.58892300	-2.44932300
H	-8.25777500	1.89422700	-0.75719600
C	-6.09116100	-0.92669800	1.68120500
C	-7.51166000	1.11489200	2.16690300
H	-7.83971900	-0.29072400	0.55586500

C	-6.60312300	-1.01830900	-2.07013700
C	-4.49528200	0.35764300	-2.49693400
H	-4.89268700	-0.80849400	-0.78197800
C	-8.19960600	4.69549700	-0.68025000
H	-6.14198300	4.08056800	-0.33703200
H	-7.26388000	3.53801100	0.90153200
C	-8.15674700	3.59717000	-2.95275200
H	-6.10223000	2.98384300	-2.65158000
H	-7.20762500	1.65106900	-3.01451100
C	-6.78374600	-1.67018700	2.83075500
H	-5.16620100	-0.44973500	2.04820900
H	-5.78655500	-1.65202300	0.91141800
C	-8.20592400	0.35084300	3.30178200
H	-6.65854400	1.68692000	2.57591700
H	-8.21442500	1.84609700	1.73977600
C	-5.99808600	-2.17512000	-2.88122700
H	-7.23053500	-0.39858500	-2.73368900
H	-7.27278800	-1.42155200	-1.29354700
C	-3.89814300	-0.81487600	-3.28570000
H	-4.99592900	1.04263900	-3.19938900
H	-3.70138200	0.92868000	-1.99228400
C	-8.07973200	4.92419600	-2.19059800
H	-8.08248900	5.64664400	-0.13425600
H	-9.21350000	4.32120800	-0.44579500
H	-8.01948900	3.76381900	-4.03446100
H	-9.16596700	3.16287500	-2.82752200

C	-7.28325600	-0.70718900	3.91159100
H	-6.08901900	-2.41158400	3.26168100
H	-7.64118800	-2.24314000	2.43215200
H	-8.54504300	1.06267900	4.07305700
H	-9.11395400	-0.14128500	2.90670200
C	-4.99523700	-1.67368900	-3.92800000
H	-6.80124400	-2.75845500	-3.36267500
H	-5.48134500	-2.86342200	-2.18723900
H	-3.20540300	-0.43645100	-4.05612900
H	-3.29652800	-1.43590400	-2.59762700
H	-8.86271600	5.61714300	-2.54183000
H	-7.10934800	5.40818900	-2.40570500
H	-7.80132500	-1.26014300	4.71329400
H	-6.41901800	-0.20493100	4.38157400
H	-4.55143900	-2.52605100	-4.46966400
H	-5.53281200	-1.06840000	-4.68189400
Cu	-4.19067200	2.05452900	0.70704600
H	-3.21698800	3.25018100	0.38071300

TS-I-II conf3

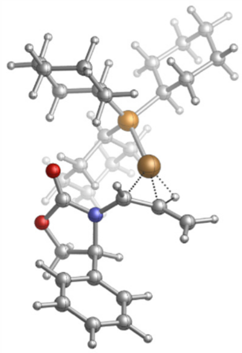

B3LYP-D3/def2-SVP-CPCM(toluene)

Zero-point correction= 0.696353 (Hartree/Particle)

Thermal correction to Energy= 0.731609

Thermal correction to Enthalpy= 0.732553

Thermal correction to Gibbs Free Energy= 0.627790

Sum of electronic and zero-point Energies= -3355.512882

Sum of electronic and thermal Energies= -3355.477626

Sum of electronic and thermal Enthalpies= -3355.476682

Sum of electronic and thermal Free Energies= -3355.581445

O	-3.04499100	-2.02106400	2.43349200
C	-1.82256500	-1.36352600	2.07028100
C	-1.92048400	0.03866800	2.70669200
H	-0.97652500	-1.95363000	2.44486500
H	-1.76749800	-1.29929900	0.97110800
H	-1.54800500	0.80685000	2.01458300
N	-3.38175600	0.14549300	2.81617700
C	-3.97969000	-1.07998100	2.76513700
O	-5.14139900	-1.35403600	2.97190600
C	-4.06765600	1.33447600	3.20044100
H	-4.71032200	1.26575700	4.08171500
C	-3.62627500	2.57850900	2.73356800
C	-3.11082800	3.71071100	3.22574400
H	-2.69506600	3.71805100	4.23871700
H	-3.09245900	4.63982900	2.65094600
C	-1.23165600	0.19946400	4.05094000
C	-1.37535800	-0.77745300	5.05027800

C	-0.47946000	1.34783600	4.32717500
C	-0.77102900	-0.60910200	6.29788200
H	-1.96584600	-1.67591300	4.85093400
C	0.12694600	1.51916200	5.57676100
H	-0.37887900	2.12119600	3.56114800
C	-0.01729400	0.54077300	6.56450600
H	-0.88855200	-1.37737700	7.06640000
H	0.71038700	2.42116000	5.77879100
H	0.45463000	0.67259500	7.54151400
P	-6.50703300	1.09953200	0.06719700
C	-7.41614500	2.36938600	-0.95279000
C	-7.88121200	0.21189100	0.99789700
C	-5.71566300	-0.07005300	-1.15968300
C	-8.43591900	1.81240900	-1.96080100
C	-8.05741700	3.43371000	-0.04141800
H	-6.60759900	2.87001300	-1.51309400
C	-7.85861600	-1.32660900	0.95073600
C	-7.88908500	0.70267200	2.46094900
H	-8.81759800	0.54245800	0.51537600
C	-4.98544500	0.71167600	-2.27189800
C	-4.71443000	-0.98359700	-0.42248300
H	-6.50702800	-0.68820700	-1.61749300
C	-9.07451000	2.94402400	-2.78056100
H	-9.23146600	1.26952900	-1.42077700
H	-7.96117200	1.08084000	-2.63416100
C	-8.69445300	4.55905600	-0.86729600

H	-8.83699400	2.96477300	0.58514500
H	-7.29729600	3.83807300	0.64900100
C	-9.04877100	-1.91020600	1.72663100
H	-6.92961900	-1.68831600	1.41621600
H	-7.87648600	-1.68659000	-0.09050600
C	-9.06566700	0.11017400	3.24524700
H	-6.93590000	0.40016500	2.92346200
H	-7.91802900	1.80478400	2.49672400
C	-4.22798100	-0.22597600	-3.22325300
H	-4.27388400	1.41307500	-1.79844300
H	-5.69157900	1.32285400	-2.85385800
C	-3.96383500	-1.91884100	-1.37520500
H	-3.98522500	-0.33328300	0.09408300
H	-5.21371400	-1.56523000	0.36109200
C	-9.71000300	4.00643500	-1.87541900
H	-9.82471300	2.52796500	-3.47370400
H	-8.29614800	3.41770400	-3.40661600
H	-9.17429400	5.29185400	-0.19733700
H	-7.89921100	5.10143800	-1.41063900
C	-9.06027300	-1.42174500	3.18024500
H	-9.00909600	-3.01204200	1.69111700
H	-9.99272900	-1.61274000	1.23224000
H	-9.02508000	0.45206000	4.29326300
H	-10.01636800	0.49089800	2.82650000
C	-3.24484800	-1.13202000	-2.47466800
H	-3.70110400	0.37100000	-3.98642800

H	-4.95850000	-0.85426400	-3.76562500
H	-3.25064500	-2.53106600	-0.79801700
H	-4.68077900	-2.62352300	-1.83513000
H	-10.13300800	4.82428100	-2.48233600
H	-10.55567000	3.55338000	-1.32530600
H	-9.93037800	-1.83453400	3.71832800
H	-8.15679300	-1.79882300	3.69153800
H	-2.73895300	-1.81545300	-3.17698700
H	-2.45553500	-0.50843400	-2.01518500
Cu	-4.93216000	2.03333400	1.33010000
H	-3.65915100	2.93337300	1.01789900

TS-I-II conf4

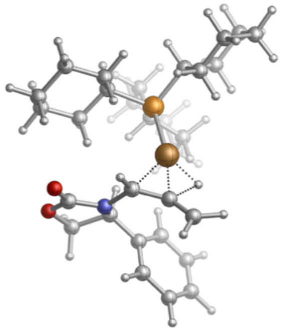

B3LYP-D3/def2-SVP-CPCM(toluene)

Zero-point correction= 0.696371 (Hartree/Particle)

Thermal correction to Energy= 0.731687

Thermal correction to Enthalpy= 0.732631

Thermal correction to Gibbs Free Energy= 0.627460

Sum of electronic and zero-point Energies= -3355.508103

Sum of electronic and thermal Energies= -3355.472788

Sum of electronic and thermal Enthalpies= -3355.471844

Sum of electronic and thermal Free Energies= -3355.577015

O	-2.83358900	-1.33212400	3.77300900
C	-1.90154700	-1.23063100	2.69637400
C	-2.23908000	0.10873900	2.00768400
H	-0.87592600	-1.21559700	3.10103500
H	-2.01720700	-2.09495600	2.02898600
H	-2.98036200	-0.05230000	1.20039100
N	-2.88890300	0.80341000	3.11257900
C	-3.33670000	-0.09016700	4.05023300
O	-4.08733300	0.11835600	4.97682800
C	-3.55027500	2.07808600	2.97396400
H	-4.23380000	2.26887400	3.80356600
C	-3.00944800	3.17430800	2.27557600
C	-2.45838000	4.35716000	2.55801700
H	-2.09282800	4.55402200	3.57264000
H	-2.34934600	5.14732200	1.81014500
C	-1.03652600	0.80799100	1.41430500
C	-0.08379500	1.42242500	2.23878100
C	-0.85172700	0.82520800	0.02692300
C	1.03292600	2.04852800	1.68239400
H	-0.23825300	1.42928000	3.32056100
C	0.26825500	1.44703700	-0.53387500
H	-1.59706900	0.35922600	-0.62187500
C	1.21220200	2.06251600	0.29337800
H	1.76409800	2.53459300	2.33334200

H	0.39690700	1.45852900	-1.61911500
H	2.08421600	2.55712900	-0.14197500
P	-5.85945500	1.24765000	-0.10657400
C	-7.16462700	2.35478100	-0.87624000
C	-6.82035600	0.10592800	1.03107300
C	-5.10532600	0.08606500	-1.35947500
C	-7.26600500	3.65268000	-0.04087700
C	-6.92328100	2.72053700	-2.35269400
H	-8.12089900	1.80514500	-0.80743900
C	-5.85694400	-0.91333400	1.66973400
C	-7.54425900	0.91551700	2.12533400
H	-7.57460500	-0.42547300	0.42338400
C	-6.06772400	-0.83080200	-2.13305400
C	-4.09049900	0.77458800	-2.29272000
H	-4.50604800	-0.56195000	-0.69428500
C	-8.36531800	4.58470900	-0.56384700
H	-6.28765000	4.16573000	-0.09406300
H	-7.43349900	3.42882200	1.02245200
C	-8.02554300	3.64873800	-2.88408300
H	-5.94699100	3.22893900	-2.44213900
H	-6.87251400	1.81824000	-2.97801000
C	-6.52739300	-1.79879400	2.72682900
H	-5.04657100	-0.33962500	2.14512500
H	-5.39314600	-1.55152200	0.90135900
C	-8.21955700	0.01205000	3.16587800
H	-6.79801400	1.55984900	2.62608800

H	-8.29791100	1.58029100	1.67580100
C	-5.28587500	-1.85466400	-2.97115500
H	-6.70609800	-0.22611800	-2.79918800
H	-6.74528100	-1.35657200	-1.44195800
C	-3.31440300	-0.26808000	-3.10757700
H	-4.61001000	1.45139700	-2.98822100
H	-3.41025100	1.40435200	-1.69644800
C	-8.15245500	4.92317200	-2.04297900
H	-8.39335400	5.50410500	0.04428600
H	-9.34750400	4.09315000	-0.43666500
H	-7.82078400	3.89973500	-3.93832800
H	-8.98859800	3.10578400	-2.87178400
C	-7.22211400	-0.95940400	3.80262500
H	-5.76386600	-2.45149100	3.18141000
H	-7.26549200	-2.45933000	2.23503100
H	-8.69849100	0.63826500	3.93716900
H	-9.03047300	-0.55977500	2.67701100
C	-4.26622600	-1.17761900	-3.89565000
H	-5.98662900	-2.47353200	-3.55605300
H	-4.75435500	-2.54299800	-2.28834200
H	-2.60829500	0.23626000	-3.78803400
H	-2.70373700	-0.88665300	-2.42344300
H	-8.97603500	5.55272100	-2.41914300
H	-7.22669800	5.51839400	-2.14692600
H	-7.73177900	-1.61351800	4.52976800
H	-6.45585400	-0.39474900	4.36077900

H -3.69700400 -1.93692700 -4.45729900

H -4.80507600 -0.57012400 -4.64625900

Cu -4.30546100 2.38452000 0.98867800

H -3.08357300 3.31725200 0.58171700

TS-I-III *cis* conf2

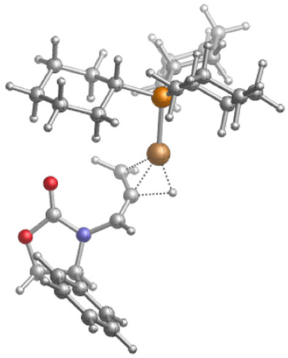

B3LYP-D3/def2-SVP-CPCM(toluene)

Zero-point correction= 0.696136 (Hartree/Particle)

Thermal correction to Energy= 0.731322

Thermal correction to Enthalpy= 0.732266

Thermal correction to Gibbs Free Energy= 0.626001

Sum of electronic and zero-point Energies= -3355.501275

Sum of electronic and thermal Energies= -3355.466089

Sum of electronic and thermal Enthalpies= -3355.465145

Sum of electronic and thermal Free Energies= -3355.571410

O -2.00170300 -1.58853400 0.38978800

C -0.93048800 -1.31579700 1.30150600

C -1.61495000 -0.75508400 2.56861900

H -0.37722400 -2.24578500 1.48650600

H -0.25636100 -0.57113800 0.84836100

H	-1.05284600	0.10472800	2.96197600
N	-2.87386500	-0.29210900	1.97582100
C	-3.11846500	-0.90380000	0.76707300
O	-4.13437400	-0.89929200	0.11327000
C	-3.85716900	0.41133700	2.70510100
H	-4.09915900	0.01986300	3.69742300
C	-4.49168300	1.46968800	2.16847900
H	-5.45678400	1.88411400	3.59907100
C	-4.71054800	2.07867500	0.93925700
H	-4.51766400	3.14811400	0.79732400
H	-4.79441500	1.44916600	0.04927600
Cu	-6.39156500	1.97002500	2.30681000
P	-8.48086600	1.89350100	1.56713900
C	-8.52034400	0.44782700	0.35809900
C	-9.03872200	3.39428500	0.57825400
C	-9.86201600	1.56956100	2.79948300
C	-8.03966900	0.80429000	-1.06236600
C	-7.65224400	-0.71054800	0.90378700
H	-9.57382600	0.11973900	0.29093900
C	-9.83918500	4.45033500	1.36492800
C	-7.81438900	4.06641300	-0.07928800
H	-9.70194000	3.00034700	-0.21303200
C	-10.05116600	0.08928200	3.18262700
C	-9.61277100	2.40066300	4.07579000
H	-10.79565100	1.91113300	2.31722900
C	-8.07661000	-0.42079700	-1.98853200

H	-7.00330000	1.17693900	-0.99871900
H	-8.65003400	1.60894800	-1.49983000
C	-7.70004300	-1.94004100	-0.01132700
H	-6.60542900	-0.36556000	0.96405100
H	-7.95042600	-0.98761000	1.92407100
C	-10.26542600	5.61017600	0.45216100
H	-9.21262900	4.85120700	2.18116000
H	-10.73117800	4.00784100	1.83293600
C	-8.22553900	5.23116700	-0.98776000
H	-7.15223100	4.43352200	0.72614300
H	-7.21454000	3.33831700	-0.64064300
C	-11.19374800	-0.08038800	4.19483400
H	-9.11641100	-0.29125100	3.63125500
H	-10.25553900	-0.52688700	2.29428000
C	-10.74432200	2.23380000	5.09767800
H	-8.65455600	2.06690300	4.51297400
H	-9.47864900	3.46529600	3.83725100
C	-7.24935100	-1.58429200	-1.43148700
H	-7.71420600	-0.13314100	-2.98999000
H	-9.12686000	-0.74403600	-2.11575700
H	-7.05689700	-2.73179700	0.40724300
H	-8.73000500	-2.34360900	-0.03447100
C	-9.06107000	6.26804900	-0.22977100
H	-10.83168400	6.35329100	1.03836600
H	-10.95735500	5.22528800	-0.31957800
H	-7.32674600	5.70019100	-1.42181400

H	-8.81398300	4.83589500	-1.83642700
C	-10.96454200	0.76029400	5.45516900
H	-11.30162700	-1.14669400	4.45531400
H	-12.14454400	0.22476700	3.71990700
H	-10.51812800	2.82206200	6.00275100
H	-11.67762900	2.65156600	4.67653600
H	-7.32819200	-2.46207800	-2.09512300
H	-6.18401200	-1.29914100	-1.39679300
H	-9.39371500	7.07062900	-0.90917400
H	-8.42873200	6.74807000	0.53974600
H	-11.81359400	0.65247200	6.15083900
H	-10.07162200	0.38040000	5.98479300
C	-1.83315800	-1.76287200	3.68512200
C	-2.42622700	-3.00795100	3.41420700
C	-1.47369300	-1.45420000	5.00278700
C	-2.64933800	-3.92459500	4.44302800
H	-2.71435200	-3.26210300	2.39052300
C	-1.69665200	-2.37181300	6.03622100
H	-1.01779800	-0.48496200	5.22419700
C	-2.28481800	-3.60836300	5.75810500
H	-3.10993000	-4.89021700	4.21952300
H	-1.41238500	-2.11690800	7.06031300
H	-2.46107600	-4.32610100	6.56326100

TS-I-III *trans* conf2

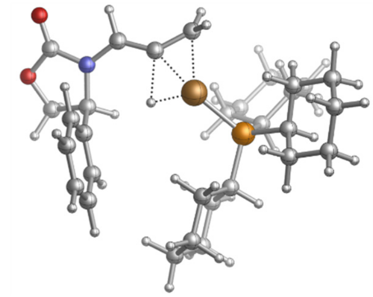

B3LYP-D3/def2-SVP-CPCM(toluene)

Zero-point correction= 0.696282 (Hartree/Particle)

Thermal correction to Energy= 0.731442

Thermal correction to Enthalpy= 0.732386

Thermal correction to Gibbs Free Energy= 0.626547

Sum of electronic and zero-point Energies= -3355.505837

Sum of electronic and thermal Energies= -3355.470677

Sum of electronic and thermal Enthalpies= -3355.469733

Sum of electronic and thermal Free Energies= -3355.575572

O	-3.07037400	-0.31440800	-5.85630700
C	-4.23032500	0.52430800	-5.87239000
H	-5.08104400	-0.04875600	-6.27730800
H	-4.03332300	1.39312100	-6.51289600
N	-3.77892300	-0.20757700	-3.74316900
C	-2.90596100	-0.83920200	-4.61479900
O	-2.11955700	-1.71634200	-4.36137100
C	-3.93894100	-0.67939000	-2.43334500
H	-3.35041000	-1.58569500	-2.26398300
C	-4.70157800	-0.21006000	-1.43521700
H	-5.07774100	1.42642300	-2.01782500
C	-5.25818900	-0.70674600	-0.23964400

H	-4.87983700	-0.38376000	0.73953000
H	-5.77909200	-1.67068300	-0.25441700
Cu	-6.22376600	0.97293800	-1.06387000
P	-8.08506500	2.04229000	-0.53096500
C	-8.27253100	2.48154000	1.29064800
C	-8.37997000	3.65424000	-1.45569500
C	-9.56327400	0.95146400	-0.93590100
C	-7.75397100	3.87622200	1.69257100
C	-7.58212700	1.41001200	2.16115200
H	-9.35939900	2.46496700	1.49043200
C	-9.00028700	3.48632000	-2.85572700
C	-7.04822600	4.42435200	-1.58184300
H	-9.08969200	4.23919900	-0.84312600
C	-9.94969200	-0.03639600	0.18172700
C	-9.27631600	0.15764200	-2.23058600
H	-10.42083300	1.62785000	-1.10400000
C	-7.98408500	4.13993400	3.18837500
H	-6.67185900	3.93846500	1.48164200
H	-8.24187600	4.66757900	1.10444300
C	-7.80061000	1.66603900	3.65718200
H	-6.50210400	1.42427200	1.92985100
H	-7.92318300	0.40036700	1.89641700
C	-9.20582100	4.84672300	-3.53811800
H	-8.32420300	2.87175200	-3.47713700
H	-9.96321100	2.95624700	-2.80457300
C	-7.23084700	5.78177600	-2.27127700

H	-6.34967600	3.79790000	-2.16252100
H	-6.57447000	4.56712500	-0.60060400
C	-11.15440300	-0.89680500	-0.22749800
H	-9.08691200	-0.69458400	0.38689100
H	-10.18261800	0.49255500	1.11784700
C	-10.47391300	-0.70329900	-2.65069500
H	-8.40357900	-0.49484000	-2.03990700
H	-8.98546700	0.82444000	-3.05367700
C	-7.32782600	3.06592900	4.06222700
H	-7.60001500	5.14005000	3.45036800
H	-9.07157300	4.15895100	3.38731800
H	-7.27736100	0.89476400	4.24662400
H	-8.87694100	1.56162300	3.88859700
C	-7.89451600	5.63156100	-3.64384400
H	-9.64886400	4.69717700	-4.53710000
H	-9.93820600	5.43451500	-2.95458000
H	-6.25111700	6.27816800	-2.36938600
H	-7.85384500	6.43620700	-1.63380700
C	-10.89314000	-1.66008500	-1.53010400
H	-11.40149500	-1.59722900	0.58774300
H	-12.03631600	-0.24272000	-0.35772900
H	-10.22580300	-1.26448900	-3.56701400
H	-11.32226500	-0.04190300	-2.90671800
H	-7.53909400	3.25399600	5.12818100
H	-6.23030600	3.12454800	3.94279700
H	-8.07127900	6.61977800	-4.10042900

H	-7.20741200	5.09108400	-4.31911600
H	-11.78572300	-2.23640300	-1.82553500
H	-10.08395200	-2.39431000	-1.36227300
C	-3.89670600	2.27775300	-4.04324200
C	-4.63893200	3.41655500	-4.39076700
C	-2.64646600	2.44071200	-3.43587600
C	-4.14580200	4.69598000	-4.12968100
H	-5.62135800	3.29743200	-4.85725300
C	-2.15241100	3.72217300	-3.16793900
H	-2.06522200	1.56165900	-3.15009500
C	-2.89888400	4.85299900	-3.51245600
H	-4.73875000	5.57308600	-4.40025100
H	-1.17898400	3.83546100	-2.68392100
H	-2.51287900	5.85316900	-3.30027400
C	-4.45769200	0.90655000	-4.39025700
H	-5.52752600	0.89164900	-4.12915400

TS-I-Q conf2

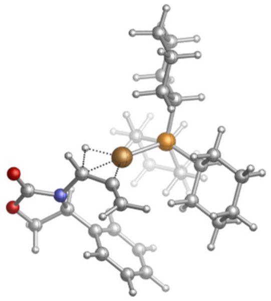

B3LYP-D3/def2-SVP-CPCM(toluene)

Zero-point correction= 0.696939 (Hartree/Particle)

Thermal correction to Energy= 0.731975

Thermal correction to Enthalpy= 0.732919

Thermal correction to Gibbs Free Energy= 0.628386

Sum of electronic and zero-point Energies= -3355.499887

Sum of electronic and thermal Energies= -3355.464851

Sum of electronic and thermal Enthalpies= -3355.463906

Sum of electronic and thermal Free Energies= -3355.568439

O	-0.40682400	-1.53007200	4.15219100
C	-0.61182700	-0.25026500	4.75786800
C	-1.17153000	0.62579600	3.62323000
H	-1.34239900	-0.34899000	5.57790400
H	0.34058500	0.12088100	5.15816100
H	-0.34404000	1.06417000	3.03095500
N	-1.79223600	-0.41141500	2.80060100
C	-1.15019100	-1.62089400	3.01886900
O	-1.21070500	-2.61563600	2.34240300
C	-2.34726800	-0.14898600	1.50532700
H	-2.21613400	-1.02173600	0.85421600
C	-3.54641600	0.62051300	1.33623300
C	-4.82049400	0.39905000	1.65926800
H	-5.16008900	-0.51093600	2.18255000
H	-5.60892000	1.12990100	1.43204300
C	-2.06219600	1.74923900	4.09851100
C	-3.35931100	1.50442300	4.57163600
C	-1.56478700	3.05891900	4.11888900
C	-4.14696900	2.55373200	5.04690300
H	-3.75903700	0.48948800	4.53459800

C	-2.34733200	4.11024500	4.60833900
H	-0.55756000	3.25834000	3.74325200
C	-3.64267000	3.86041000	5.06838800
H	-5.16274600	2.35384300	5.39687800
H	-1.94712900	5.12701000	4.61935500
H	-4.26177600	4.68139600	5.43846500
P	-1.99320800	3.73067500	-0.33133800
C	-1.79532300	3.54846300	-2.19379600
C	-0.63561400	4.92291800	0.19672600
C	-3.61533100	4.64896500	-0.07766200
C	-0.33794200	3.56799100	-2.69487400
C	-2.47063600	2.23913400	-2.66025600
H	-2.31959800	4.40956400	-2.64668100
C	-0.99627400	5.86847300	1.35783900
C	0.63258700	4.11983400	0.55416000
H	-0.42623000	5.55363500	-0.68548400
C	-4.74282200	4.16963900	-1.01259400
C	-4.06821500	4.48006000	1.38870000
H	-3.42123900	5.71625900	-0.28940400
C	-0.27625000	3.42326300	-4.22290600
H	0.21289600	2.73010500	-2.23226700
H	0.17553800	4.49398000	-2.39607100
C	-2.41549600	2.08165700	-4.18464900
H	-1.93872700	1.39485400	-2.18278400
H	-3.50986900	2.17745500	-2.31115500
C	0.17910400	6.79241300	1.70814800

H	-1.25460800	5.27135900	2.24694800
H	-1.88051200	6.47534900	1.11254300
C	1.80809100	5.03387300	0.91919900
H	0.38831500	3.45911800	1.40504300
H	0.91729700	3.44590700	-0.26607600
C	-6.06043400	4.90605100	-0.72906200
H	-4.89489700	3.08712700	-0.85514500
H	-4.46956100	4.30956000	-2.06948100
C	-5.38238800	5.21231100	1.67884700
H	-4.19424500	3.39979300	1.57794800
H	-3.29494400	4.81527600	2.09009200
C	-0.97485800	2.14629200	-4.70198100
H	0.77608900	3.43507200	-4.55282200
H	-0.76105300	4.30174300	-4.68737700
H	-2.89031800	1.13057900	-4.47770800
H	-3.01082100	2.88761900	-4.65232600
C	1.44050400	5.99458500	2.05462700
H	-0.10556500	7.45010000	2.54666700
H	0.39027500	7.45387000	0.84773200
H	2.68314400	4.42240400	1.19599700
H	2.10271300	5.61818100	0.02793700
C	-6.49493600	4.75265900	0.73214600
H	-6.84486200	4.53151200	-1.40802400
H	-5.93115300	5.97973400	-0.96020800
H	-5.66997200	5.04183800	2.72994400
H	-5.23119100	6.30221600	1.56528600

H -0.95463400 2.08565100 -5.80295100

H -0.41788200 1.26805800 -4.32704600

H 2.28006600 6.67492100 2.27466400

H 1.25739200 5.41204000 2.97677400

H -7.42547600 5.31448500 0.91936100

H -6.72022700 3.68883900 0.93192500

Cu -2.07108500 1.73605100 0.62544600

H -0.94464600 0.60240100 0.85854200

TS-I-Q conf3

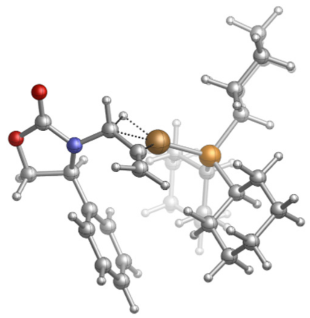

B3LYP-D3/def2-SVP-CPCM(toluene)

Zero-point correction= 0.696953 (Hartree/Particle)

Thermal correction to Energy= 0.731973

Thermal correction to Enthalpy= 0.732917

Thermal correction to Gibbs Free Energy= 0.628666

Sum of electronic and zero-point Energies= -3355.499854

Sum of electronic and thermal Energies= -3355.464834

Sum of electronic and thermal Enthalpies= -3355.463890

Sum of electronic and thermal Free Energies= -3355.568141

O -3.43595300 -2.54796000 3.55770900

C -2.11615400 -2.10898100 3.22239600

C	-2.32994200	-0.76409000	2.50595000
H	-1.53151800	-1.97533700	4.14772800
H	-1.63124800	-2.86216100	2.58790100
H	-2.52479500	-0.92844300	1.42762700
N	-3.59504400	-0.36184000	3.11910400
C	-4.28704100	-1.48946100	3.53410100
O	-5.45096700	-1.56646500	3.83431100
C	-4.33821200	0.78438900	2.68557900
H	-5.41345600	0.60598400	2.80716400
C	-3.86589700	2.12143300	2.90607100
C	-3.68846100	2.85123000	4.00721800
H	-3.88950200	2.46907100	5.02246500
H	-3.31158300	3.88238100	3.96150700
C	-1.16995000	0.19406900	2.63797600
C	-0.89647200	0.84850900	3.84765900
C	-0.31772200	0.39727900	1.54470100
C	0.20526800	1.69779700	3.95715100
H	-1.57261100	0.71249700	4.69366900
C	0.79532700	1.23745300	1.65628400
H	-0.53091400	-0.10200400	0.59575100
C	1.05615200	1.89374800	2.86170600
H	0.39906700	2.21598800	4.89957700
H	1.45256400	1.38706000	0.79619800
H	1.91701900	2.56135200	2.94827000
P	-3.12265000	3.07544300	-0.75276800
C	-2.02108300	2.32551500	-2.08196200

C	-2.22533800	4.61094600	-0.13996700
C	-4.64339800	3.70395500	-1.66709900
C	-0.50539000	2.48544000	-1.85970400
C	-2.36778400	0.83077400	-2.24481900
H	-2.27139200	2.85711700	-3.01715400
C	-3.17588300	5.68330700	0.42749600
C	-1.21190100	4.20410200	0.95160200
H	-1.69103200	5.04406500	-1.00521300
C	-5.08853300	2.83307000	-2.85808500
C	-5.82195000	3.84240500	-0.67717700
H	-4.37844200	4.70260800	-2.05945500
C	0.29393900	1.84106300	-3.00157900
H	-0.22286700	2.00080000	-0.91126600
H	-0.22944500	3.54670900	-1.77113500
C	-1.56497700	0.17505200	-3.37445100
H	-2.15426200	0.32514000	-1.28614300
H	-3.44459900	0.69125400	-2.41507300
C	-2.40145500	6.89138300	0.97459100
H	-3.76493500	5.23306500	1.24610400
H	-3.88964600	6.02624600	-0.33681500
C	-0.43655000	5.40522100	1.50281300
H	-1.77479100	3.71854500	1.76770500
H	-0.50870300	3.44690300	0.58532800
C	-6.32678300	3.42729400	-3.54613400
H	-5.33280900	1.82044400	-2.49155700
H	-4.28080700	2.72127700	-3.59665300

C	-7.06595900	4.43390000	-1.35138900
H	-6.05754600	2.83388000	-0.28841800
H	-5.54270000	4.44533900	0.19701600
C	-0.05753000	0.35938900	-3.17172900
H	1.37355200	1.96344400	-2.81144400
H	0.07809400	2.37805500	-3.94363900
H	-1.81984600	-0.89594400	-3.43943500
H	-1.86150200	0.62739100	-4.33883600
C	-1.38552100	6.47830400	2.04425100
H	-3.11216800	7.63107600	1.37998500
H	-1.87151200	7.38877400	0.14109800
H	0.25397500	5.06180500	2.29138300
H	0.18986400	5.84025200	0.70155600
C	-7.49185300	3.60391800	-2.56664700
H	-6.62545000	2.78271500	-4.38976200
H	-6.06114600	4.40917700	-3.97954000
H	-7.88860700	4.50107200	-0.62020100
H	-6.84483600	5.46883500	-1.67190300
H	0.50374000	-0.07656400	-4.01497600
H	0.25597900	-0.19249400	-2.26602400
H	-0.82116400	7.35634800	2.40081300
H	-1.92677300	6.07177600	2.91842600
H	-8.35361700	4.07040900	-3.07258800
H	-7.82831600	2.60854900	-2.22295200
Cu	-3.73848800	1.80755300	0.95384100
H	-4.40510200	0.33840400	1.02955600

TS-I-Q conf4

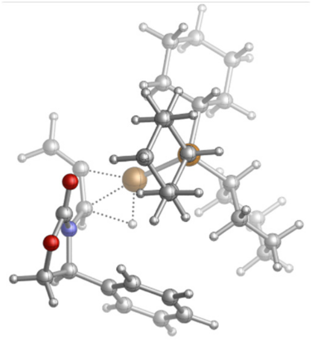

B3LYP-D3/def2-SVP-CPCM(toluene)

Zero-point correction= 0.697362 (Hartree/Particle)

Thermal correction to Energy= 0.732247

Thermal correction to Enthalpy= 0.733191

Thermal correction to Gibbs Free Energy= 0.629548

Sum of electronic and zero-point Energies= -3355.497499

Sum of electronic and thermal Energies= -3355.462614

Sum of electronic and thermal Enthalpies= -3355.461670

Sum of electronic and thermal Free Energies= -3355.565313

O	-0.09962500	-1.21052400	4.29924400
C	0.04067800	0.01520900	5.02940100
C	-1.16827300	0.85959800	4.58879800
H	0.04492200	-0.21005200	6.10341000
H	0.99155900	0.49564300	4.74720300
H	-0.89295100	1.92060300	4.49603700
N	-1.37168600	0.28667800	3.24974200
C	-0.89455700	-1.00952400	3.20939600
O	-1.12051300	-1.87668700	2.40319000

C	-2.31007500	0.84125200	2.33537500
H	-2.42804100	1.91597200	2.51767000
C	-2.41815300	0.40026100	0.96846400
C	-1.57941900	0.48952000	-0.06411800
H	-0.57110500	0.92909400	0.01870700
H	-1.84610400	0.11205600	-1.05961800
C	-2.39755900	0.73266600	5.47794200
C	-2.81394200	-0.52256700	5.95161900
C	-3.16192100	1.86301300	5.79256900
C	-3.98055700	-0.64244000	6.70778000
H	-2.23725800	-1.41773800	5.70832700
C	-4.33362800	1.74514900	6.54765000
H	-2.84825100	2.84501800	5.42698400
C	-4.74862200	0.49040300	7.00272900
H	-4.29822900	-1.62684200	7.06015600
H	-4.92680200	2.63449100	6.77483100
H	-5.66843200	0.39341100	7.58474600
P	-5.75934600	-1.59963100	1.85163900
C	-5.09349300	-3.22692700	2.52937000
C	-6.51048900	-2.04804500	0.18192900
C	-7.20639900	-1.08575600	2.93666400
C	-4.31509000	-4.02922400	1.46471900
C	-4.16215200	-2.95931900	3.73264500
H	-5.96422800	-3.82508900	2.85711200
C	-7.78521900	-1.27673500	-0.21163600
C	-5.44971800	-1.87297900	-0.92593100

H	-6.78277700	-3.11679400	0.25581300
C	-7.01001700	-1.38481000	4.43390300
C	-7.44777800	0.42759400	2.74070100
H	-8.09189100	-1.64702500	2.59009900
C	-3.72080600	-5.32069700	2.04557400
H	-3.49322300	-3.39803800	1.08384200
H	-4.95845800	-4.28175900	0.60868600
C	-3.57546700	-4.24948800	4.31533400
H	-3.33443700	-2.32289600	3.38372100
H	-4.67091100	-2.39178300	4.51803600
C	-8.32044500	-1.75522100	-1.56994900
H	-7.55393500	-0.19927800	-0.28132800
H	-8.57114100	-1.38688600	0.55060100
C	-5.97081600	-2.34425800	-2.28883400
H	-5.17932800	-0.80319000	-0.97291200
H	-4.51752300	-2.39619600	-0.67289300
C	-8.18554400	-0.87191200	5.27731600
H	-6.08645200	-0.89059900	4.77834700
H	-6.88452900	-2.46518700	4.60192800
C	-8.61851300	0.94434200	3.58543600
H	-6.51873000	0.95139200	3.03177000
H	-7.61720500	0.66630800	1.68081800
C	-2.81361400	-5.03861400	3.24718500
H	-3.16384000	-5.85257600	1.25592900
H	-4.54404600	-5.99113500	2.35608000
H	-2.91071700	-3.99875800	5.15985800

H	-4.39043500	-4.87214700	4.73018700
C	-7.26614300	-1.62188000	-2.67434100
H	-9.22852900	-1.18634500	-1.83180100
H	-8.62717500	-2.81398400	-1.48300700
H	-5.19671100	-2.18682800	-3.05837500
H	-6.15675000	-3.43367200	-2.24882900
C	-8.41615900	0.62811700	5.07049200
H	-7.99447100	-1.09042500	6.34180600
H	-9.10301100	-1.42460500	5.00242300
H	-8.73572700	2.03031600	3.43208200
H	-9.55648200	0.47446300	3.23559200
H	-2.41927400	-5.98103700	3.66327100
H	-1.94780000	-4.43845700	2.91647800
H	-7.65811500	-2.00953700	-3.62968400
H	-7.04652900	-0.55028000	-2.83496300
H	-9.28042600	0.97276000	5.66285200
H	-7.53394800	1.18164100	5.44129500
Cu	-4.07252400	-0.16346500	1.89979100
H	-3.79945800	0.54089900	3.29641600

(E)* Allylic System/(E) s-*trans

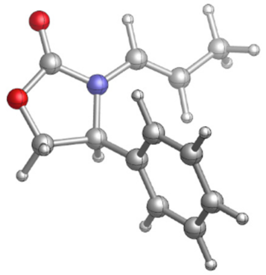

B3LYP-D3/def2-SVP-CPCM(toluene)

Zero-point correction= 0.229284 (Hartree/Particle)

Thermal correction to Energy= 0.242539

Thermal correction to Enthalpy= 0.243483

Thermal correction to Gibbs Free Energy= 0.188213

Sum of electronic and zero-point Energies= -669.626959

Sum of electronic and thermal Energies= -669.613704

Sum of electronic and thermal Enthalpies= -669.612760

Sum of electronic and thermal Free Energies= -669.668030

O	0.55494800	5.67559000	-0.32647000
C	0.38547400	6.59219100	-1.40962300
C	-0.97040300	6.22011200	-2.06598900
H	0.35924500	7.61702200	-1.00731000
H	1.23296700	6.49615900	-2.10134500
H	-0.81298300	5.59778000	-2.96616700
N	-1.55073800	5.39890300	-1.01129400
C	-0.61321700	5.04370000	-0.05434500
O	-0.76458000	4.29165000	0.87369300
C	-2.78124200	4.73571900	-1.09101400
H	-2.97033500	4.11629400	-0.20992100
C	-3.65520700	4.81113600	-2.10627400
H	-3.43547700	5.44818700	-2.96824800
C	-1.78266700	7.44192300	-2.45097300
C	-2.47241800	8.18117500	-1.47883600
C	-1.81149000	7.87158700	-3.78375100
C	-3.18020600	9.33070700	-1.83610900

H	-2.46407100	7.84254200	-0.43975100
C	-2.51458300	9.02689000	-4.14246400
H	-1.28446100	7.29516400	-4.54960800
C	-3.20158400	9.75825400	-3.16924700
H	-3.72095900	9.89443300	-1.07171100
H	-2.52957700	9.35179700	-5.18583900
H	-3.75670900	10.65734200	-3.44819900
C	-4.96093000	4.07181400	-2.12787100
H	-5.02929600	3.40311100	-3.00418200
H	-5.10025600	3.45912700	-1.22325900
H	-5.81507400	4.76840100	-2.20083200

***(Z)* Allylic System**

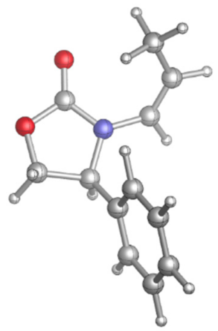

B3LYP-D3/def2-SVP-CPCM(toluene)

Zero-point correction= 0.229135 (Hartree/Particle)

Thermal correction to Energy= 0.242288

Thermal correction to Enthalpy= 0.243233

Thermal correction to Gibbs Free Energy= 0.188188

Sum of electronic and zero-point Energies= -669.622133

Sum of electronic and thermal Energies= -669.608979

Sum of electronic and thermal Enthalpies= -669.608035

Sum of electronic and thermal Free Energies= -669.663080

O	-0.09664000	7.01830800	0.71502800
C	-0.20910900	6.57154500	-0.63781700
C	-1.27009000	5.45021500	-0.59871500
H	0.77166300	6.22569400	-0.98983200
H	-0.54967500	7.40944200	-1.26838900
N	-1.98206100	5.83100500	0.61998300
C	-1.19734700	6.64228300	1.42301300
O	-1.39492000	6.97763900	2.56145000
C	-3.08547800	5.08620000	1.08975400
H	-3.02399500	4.01215800	0.88077200
C	-4.14089200	5.60506800	1.73312800
H	-4.89074300	4.88954600	2.08647000
H	-0.78291100	4.46715300	-0.45215900
C	-2.13508900	5.39079500	-1.83898100
C	-3.16275700	6.32508700	-2.04204000
C	-1.88824900	4.42393800	-2.82259800
C	-3.92669300	6.29276300	-3.21072500
H	-3.36871100	7.07199200	-1.27168900
C	-2.64840700	4.39435100	-3.99661300
H	-1.09634300	3.68534700	-2.66817400
C	-3.66984900	5.32856900	-4.19277200
H	-4.72856600	7.02126200	-3.35497500
H	-2.44545700	3.63538700	-4.75637200
H	-4.26892300	5.30307600	-5.10649000

C	-4.37864600	7.05571000	2.02015800
H	-5.45341300	7.29307700	1.97845100
H	-4.00748400	7.32698200	3.02264300
H	-3.84398000	7.69992800	1.30508300

II'

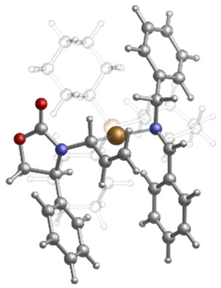

B3LYP-D3/def2-SVP-CPCM(toluene)

Zero-point correction= 0.937321 (Hartree/Particle)

Thermal correction to Energy= 0.986032

Thermal correction to Enthalpy= 0.986976

Thermal correction to Gibbs Free Energy= 0.853079

Sum of electronic and zero-point Energies= -3951.045958

Sum of electronic and thermal Energies= -3950.997247

Sum of electronic and thermal Enthalpies= -3950.996302

Sum of electronic and thermal Free Energies= -3951.130199

C	1.58475300	0.26527500	-3.96487800
H	1.08493600	-0.62149100	-4.36800400
C	0.99230500	1.06141800	-3.04556200
H	1.55786100	1.93941600	-2.71810100
C	-0.26542200	0.80524900	-2.34220300
H	-0.90886300	0.10375000	-2.89513600

Cu	0.08961700	-0.28762900	-0.58279200
P	-0.77114000	-0.56040900	1.51992100
C	-0.64339400	0.90371400	2.69811000
C	-2.60742900	-0.96227700	1.42161900
C	-0.02187000	-1.99176100	2.47283000
C	-1.73955100	1.96685800	2.49705600
C	0.75754600	1.53560700	2.57330400
H	-0.75955700	0.50413400	3.71939800
C	-2.86650500	-2.46737000	1.22582800
C	-3.21170300	-0.17052900	0.24269000
H	-3.08259400	-0.64299600	2.36627400
C	-0.48623300	-2.15636000	3.92902600
C	1.51387300	-2.04684100	2.37200600
H	-0.39021300	-2.85609400	1.89780300
C	-1.54335900	3.17263300	3.42593200
H	-1.72486800	2.31694500	1.45387900
H	-2.73579800	1.53175000	2.66473600
C	0.94161500	2.75813200	3.48030700
H	0.90704900	1.83103200	1.52131000
H	1.53769200	0.79180600	2.79176100
C	-4.35003800	-2.77460400	0.98250000
H	-2.28287100	-2.82029000	0.36135400
H	-2.51989500	-3.04471300	2.09521000
C	-4.69242800	-0.47695700	0.00697400
H	-2.65237400	-0.43184800	-0.67086000
H	-3.07414200	0.91099600	0.37441300

C	0.05476400	-3.46154800	4.53150700
H	-0.11438100	-1.31058600	4.53376300
H	-1.58626300	-2.13013800	3.99665700
C	2.06088600	-3.34018700	2.99221300
H	1.96131700	-1.18625300	2.89804400
H	1.81384500	-1.96679600	1.31657900
C	-0.15694000	3.80075100	3.25158700
H	-2.33364800	3.91789000	3.23477800
H	-1.66539400	2.84679300	4.47532900
H	1.93838600	3.19945800	3.30938800
H	0.91976400	2.43244000	4.53634800
C	-4.91399100	-1.97811200	-0.19786000
H	-4.47061100	-3.85806300	0.81360800
H	-4.92623900	-2.53067300	1.89459800
H	-5.03209600	0.09387100	-0.87168900
H	-5.28791800	-0.13099600	0.87262900
C	1.58433800	-3.52344200	4.43825200
H	-0.27252200	-3.55810100	5.58042000
H	-0.38016300	-4.31841700	3.98482900
H	3.16273000	-3.34247200	2.94402500
H	1.71919000	-4.20139300	2.39087700
H	-0.02891500	4.65342000	3.93882000
H	-0.06636800	4.20774300	2.22760600
H	-5.98532700	-2.20047300	-0.33775300
H	-4.40013500	-2.29359300	-1.12449000
H	1.95686900	-4.47735600	4.84783500

H	2.01596100	-2.72201200	5.06643000
C	2.58844800	-1.42787000	-1.79054700
N	1.38944900	-1.71481800	-1.40597100
C	-0.16597100	-3.66704000	-1.44698100
C	0.35560200	-4.29202700	-0.30538900
C	-1.44726400	-4.02960800	-1.88863100
C	-0.38726500	-5.25606300	0.38160900
H	1.34989500	-4.01105200	0.04749000
C	-2.18842600	-5.00327700	-1.21110200
H	-1.87290800	-3.53809200	-2.76799300
C	-1.66155000	-5.61715700	-0.07061600
H	0.02915600	-5.72650800	1.27619200
H	-3.18815800	-5.26756800	-1.56446100
H	-2.24393100	-6.36863300	0.46817900
C	0.65780700	-2.67071000	-2.23178100
H	-0.01127000	-2.09578000	-2.89300400
H	1.35106800	-3.22180200	-2.89722500
H	3.03468100	-1.96820800	-2.64133000
C	3.44736200	-0.41543300	-1.16970500
C	4.70845900	-0.14933600	-1.73925200
C	3.05043100	0.33243800	-0.04447800
C	5.53578600	0.84263000	-1.21310000
H	5.03059800	-0.71840600	-2.61576300
C	3.87704600	1.32368200	0.48303100
H	2.08871400	0.12657400	0.42528900
C	5.12076000	1.58738500	-0.10112200

H	6.50680700	1.04137100	-1.67331700
H	3.54710200	1.89495400	1.35406700
H	5.76547800	2.36927100	0.30789300
H	2.58301000	0.48969600	-4.34881400
N	-1.09286600	1.98901100	-2.06731900
C	-0.70450100	2.99037600	-1.08565300
C	-2.45096500	1.91077100	-2.17295700
C	-2.01697700	3.79560700	-0.96092000
O	-3.03199200	2.92351700	-1.44847500
O	-3.11573700	1.08205100	-2.75653100
H	-2.24622000	4.08269700	0.07511300
H	-1.98720200	4.70538500	-1.58251100
H	-0.46857600	2.46170700	-0.14352900
C	0.48902100	3.84657100	-1.46030600
C	1.56828000	3.98338700	-0.57972700
C	0.53571000	4.50668700	-2.69840600
C	2.67880000	4.76190300	-0.92464800
H	1.55257800	3.45772900	0.37617900
C	1.64101500	5.28389600	-3.04675000
H	-0.28948400	4.37939100	-3.40391600
C	2.71795500	5.41396900	-2.15944700
H	3.51826400	4.84623400	-0.23014000
H	1.66957200	5.78383500	-4.01831900
H	3.58644200	6.01737600	-2.43548700

TS-II’-X

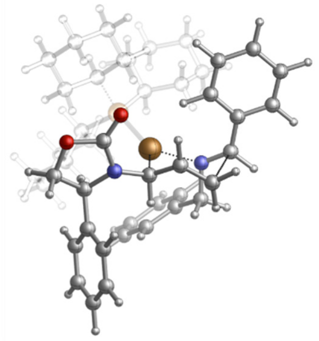

B3LYP-D3/def2-SVP-CPCM(toluene)

Zero-point correction= 0.937679 (Hartree/Particle)

Thermal correction to Energy= 0.985253

Thermal correction to Enthalpy= 0.986197

Thermal correction to Gibbs Free Energy= 0.855609

Sum of electronic and zero-point Energies= -3951.039139

Sum of electronic and thermal Energies= -3950.991566

Sum of electronic and thermal Enthalpies= -3950.990621

Sum of electronic and thermal Free Energies= -3951.121209

C	-2.88683700	3.50656300	-0.00221000
H	-3.37576700	2.70120400	-0.55841200
C	-3.67720800	4.44736800	0.66169400
H	-3.18322600	5.37352500	0.96303700
C	-4.98041500	4.23107800	1.13737200
H	-5.58538700	3.44609800	0.66956100
Cu	-4.61737800	2.95400900	3.06342500
P	-5.54255100	2.86723100	5.13050800
C	-5.39385400	4.33344600	6.29972400
C	-7.37527600	2.45994200	5.05446300
C	-4.77085400	1.44003200	6.07212300

C	-6.48446400	5.40358500	6.11120100
C	-3.98934300	4.95563800	6.16972100
H	-5.50532600	3.93112500	7.32041700
C	-7.62876100	0.95358700	4.86321100
C	-7.98655200	3.24449500	3.87599300
H	-7.84291700	2.78271900	6.00153900
C	-5.21867700	1.27879000	7.53405700
C	-3.23612300	1.38081700	5.95232500
H	-5.14704800	0.57586800	5.50223600
C	-6.27543200	6.60127300	7.04800500
H	-6.47229800	5.76034800	5.07061400
H	-7.48281800	4.97371600	6.28040400
C	-3.79263900	6.16616300	7.08993000
H	-3.84190100	5.26380600	5.12132400
H	-3.21322600	4.20396900	6.37310800
C	-9.11214900	0.63978300	4.62911000
H	-7.04877600	0.60283000	3.99536900
H	-7.27360800	0.37907500	5.73081300
C	-9.46685000	2.93161200	3.64523700
H	-7.42689700	2.97742800	2.96396600
H	-7.85349000	4.32682300	4.00445000
C	-4.67647700	-0.02934300	8.12915500
H	-4.83465700	2.12255000	8.13402900
H	-6.31766900	1.30980900	7.61623300
C	-2.68972300	0.08354100	6.56477100
H	-2.77925800	2.23813600	6.47552300

H	-2.94697800	1.45992200	4.89340800
C	-4.88404300	7.21953900	6.87622000
H	-7.06010300	7.35446400	6.86489300
H	-6.39805800	6.26792300	8.09486100
H	-2.79319900	6.60149800	6.92029600
H	-3.81355400	5.82845200	8.14215600
C	-9.68426900	1.42893800	3.44786500
H	-9.22850100	-0.44460700	4.46457200
H	-9.68499400	0.88485300	5.54300300
H	-9.81313200	3.49735400	2.76584200
H	-10.05984900	3.27980100	4.51159400
C	-3.14863800	-0.09931200	8.01645100
H	-4.99148600	-0.12418500	9.18196800
H	-5.12294000	-0.88355800	7.58769900
H	-1.58871200	0.07542500	6.50227900
H	-3.04352500	-0.77470700	5.96609000
H	-4.74772800	8.06333600	7.57269800
H	-4.79266300	7.63780800	5.85679100
H	-10.75555700	1.20297400	3.31401600
H	-9.17347600	1.11080400	2.52062100
H	-2.77662000	-1.05589700	8.42025300
H	-2.70482700	0.69884400	8.64032300
C	-2.22945800	2.17747900	1.61087700
N	-3.35379200	1.70453200	2.15215900
C	-4.91920800	-0.23807900	2.11569900
C	-4.44530300	-0.86136300	3.28013100

C	-6.20550600	-0.56016000	1.65859100
C	-5.23219100	-1.79047800	3.96495700
H	-3.44910600	-0.60345400	3.64477800
C	-6.99427900	-1.49716900	2.33625100
H	-6.59557000	-0.06933900	0.76227200
C	-6.50964300	-2.11569200	3.49238600
H	-4.84874800	-2.26277600	4.87348100
H	-7.99662200	-1.73055600	1.96834300
H	-7.12721800	-2.84032300	4.02897500
C	-4.02962500	0.70503000	1.33529600
H	-4.63812700	1.19690100	0.55069700
H	-3.28426200	0.09335000	0.78566500
H	-1.71349300	1.54893000	0.86917300
C	-1.32759900	3.10273800	2.33592300
C	-0.04324100	3.36533000	1.81731100
C	-1.70682700	3.77339000	3.51209300
C	0.81406600	4.27456100	2.43611100
H	0.27728200	2.85425600	0.90464900
C	-0.85243800	4.68779800	4.13352100
H	-2.68098400	3.56912500	3.95793400
C	0.41153100	4.94872800	3.59779200
H	1.80350500	4.46203500	2.01058200
H	-1.17883200	5.19623300	5.04464000
H	1.08087500	5.66497900	4.08091900
H	-1.91965400	3.82777800	-0.39360400
N	-5.81713700	5.33283500	1.53601600

C	-5.42443400	6.34005500	2.51462600
C	-7.17975700	5.23083600	1.44539100
C	-6.75559700	7.11049200	2.66877600
O	-7.76153900	6.22109000	2.18899800
O	-7.82738400	4.39823500	0.85266000
H	-6.97461400	7.38222300	3.71033600
H	-6.75800400	8.02513200	2.05487000
H	-5.15590000	5.81156300	3.44731900
C	-4.26635700	7.23458200	2.11916900
C	-3.17262800	7.39548600	2.97800900
C	-4.26820500	7.90476300	0.88578300
C	-2.09340400	8.20795500	2.61401500
H	-3.14889600	6.86012700	3.92840300
C	-3.19296000	8.71523500	0.51897300
H	-5.10590600	7.76226800	0.19787900
C	-2.10086200	8.86911600	1.38329000
H	-1.24090500	8.31026200	3.28972600
H	-3.20034500	9.22277800	-0.44893500
H	-1.25592200	9.49828400	1.09242100

X

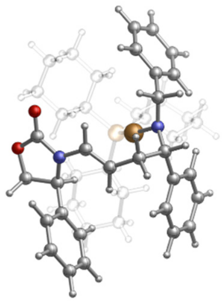

B3LYP-D3/def2-SVP-CPCM(toluene)

Zero-point correction= 0.939616 (Hartree/Particle)

Thermal correction to Energy= 0.987510

Thermal correction to Enthalpy= 0.988454

Thermal correction to Gibbs Free Energy= 0.854780

Sum of electronic and zero-point Energies= -3951.064559

Sum of electronic and thermal Energies= -3951.016665

Sum of electronic and thermal Enthalpies= -3951.015721

Sum of electronic and thermal Free Energies= -3951.149395

C	1.88090600	0.01095400	-3.43290200
H	1.37055000	-0.61894900	-4.17814900
C	0.93886700	1.03876100	-2.89507200
H	1.37977800	1.83833000	-2.30099000
C	-0.39541200	0.98761200	-3.02175600
H	-0.90465400	0.21054400	-3.59736800
Cu	0.42487200	-1.01839700	-0.25577100
P	-0.61697800	-0.72014300	1.65765100
C	-0.16995500	0.82414600	2.62950600
C	-2.46804100	-0.87949800	1.45997400
C	-0.11549900	-2.11710800	2.78913100
C	-1.23641500	1.92964200	2.69852600
C	1.15509400	1.38567100	2.07419200
H	0.00811400	0.48220500	3.66380000
C	-2.79138800	-2.34013200	1.07752000
C	-2.94729100	0.06890900	0.34176600
H	-2.97200400	-0.62241000	2.40785300

C	-0.85272200	-2.19649800	4.13534000
C	1.41152700	-2.14469800	2.98721700
H	-0.37259700	-3.01068400	2.19573800
C	-0.74202500	3.11210800	3.54359700
H	-1.45546100	2.29413900	1.68113500
H	-2.18233400	1.53812800	3.10438100
C	1.64685100	2.59702800	2.87408700
H	0.99965000	1.66524500	1.01706000
H	1.93138800	0.60694900	2.04730000
C	-4.26189400	-2.54142200	0.69444700
H	-2.15411200	-2.62535800	0.22268200
H	-2.53480300	-3.02816800	1.89575400
C	-4.42395900	-0.13251300	-0.01400200
H	-2.33001900	-0.13217800	-0.55247000
H	-2.77172000	1.11778300	0.61384000
C	-0.41652000	-3.44213500	4.92262400
H	-0.62999600	-1.29553200	4.73484800
H	-1.94407000	-2.21097000	3.98492300
C	1.83906000	-3.39231600	3.77022600
H	1.73524300	-1.24502900	3.54085400
H	1.91222500	-2.10755100	2.00703200
C	0.56992300	3.68315600	2.99196400
H	-1.51761100	3.89537600	3.58062700
H	-0.58685900	2.77311700	4.58434000
H	2.56001000	3.00122000	2.40573300
H	1.93657000	2.26299500	3.88730500

C	-4.69684400	-1.58311600	-0.41702500
H	-4.41049800	-3.58980400	0.38671900
H	-4.89679600	-2.37835000	1.58524900
H	-4.69515500	0.55636700	-0.82730200
H	-5.04899800	0.13266500	0.85915700
C	1.10476400	-3.48754500	5.11339300
H	-0.92909600	-3.46951000	5.89881300
H	-0.74083300	-4.34383100	4.37135400
H	2.93090700	-3.38436800	3.92543000
H	1.61334700	-4.28954400	3.16511600
H	0.92697900	4.51047800	3.62754200
H	0.37792000	4.11868800	1.99475800
H	-5.76369200	-1.72615800	-0.65706000
H	-4.13016200	-1.80807100	-1.33878800
H	1.39662300	-4.40701000	5.64783200
H	1.41369900	-2.64041900	5.75382300
C	2.44874100	-0.94731300	-2.32570200
N	1.43359300	-1.73639300	-1.68357800
C	-0.03276600	-3.69518600	-1.69419000
C	0.43674600	-4.22782300	-0.48000500
C	-1.32374500	-4.04429400	-2.11506700
C	-0.35432000	-5.09159500	0.28063300
H	1.42999500	-3.93416800	-0.13458400
C	-2.12103200	-4.91219200	-1.35749800
H	-1.71424300	-3.62304300	-3.04615900
C	-1.63919400	-5.43932800	-0.15636000

H	0.03090000	-5.49428900	1.22173300
H	-3.12796500	-5.16335800	-1.70127200
H	-2.26317000	-6.10865400	0.44159300
C	0.84529100	-2.75958000	-2.50737200
H	0.22826600	-2.37757600	-3.35600300
H	1.64092200	-3.37253200	-2.99946800
H	3.15341300	-1.61945400	-2.87448900
C	3.29526600	-0.22269300	-1.26644600
C	3.79952700	1.07945400	-1.41764600
C	3.59430500	-0.89756800	-0.06787300
C	4.53391900	1.69850800	-0.39759800
H	3.61906000	1.64043600	-2.33540900
C	4.33567300	-0.29134100	0.94812900
H	3.20779200	-1.90986900	0.05298500
C	4.80037700	1.02023700	0.79446900
H	4.89370300	2.72128000	-0.54015400
H	4.54265300	-0.84253200	1.86984600
H	5.36742100	1.50564200	1.59313800
H	2.71737400	0.49879800	-3.96351900
N	-1.28806500	1.90199300	-2.44020000
C	-0.91761700	2.85494700	-1.39853300
C	-2.65255800	1.69967800	-2.48772400
C	-2.30076100	3.47837400	-1.08911600
O	-3.26100100	2.58436300	-1.65434600
O	-3.25386700	0.87083800	-3.12404000
H	-2.49029400	3.57755300	-0.01159900

H	-2.40807700	4.46502300	-1.56527000
H	-0.53472600	2.28364100	-0.53379900
C	0.11974100	3.88661600	-1.79909100
C	1.19573400	4.17276100	-0.94914100
C	0.01427500	4.56796900	-3.02013000
C	2.15092100	5.12943200	-1.30804600
H	1.30477200	3.62580100	-0.01118500
C	0.96882600	5.52044500	-3.38282600
H	-0.81098900	4.33382200	-3.69785900
C	2.03965400	5.80545700	-2.52634200
H	2.98791200	5.33663400	-0.63670600
H	0.88155000	6.03886400	-4.34096300
H	2.78809700	6.54845600	-2.81281500

TS-III'-IV *cis (S,S,S)* conf2

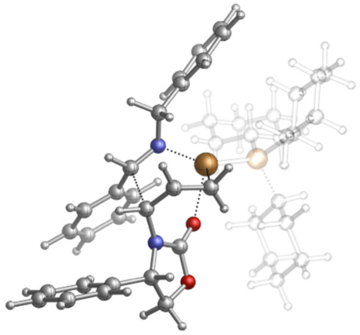

B3LYP-D3/def2-SVP-CPCM(toluene)

Zero-point correction= 0.937168 (Hartree/Particle)

Thermal correction to Energy= 0.984737

Thermal correction to Enthalpy= 0.985681

Thermal correction to Gibbs Free Energy= 0.853783

Sum of electronic and zero-point Energies= -3951.035426

Sum of electronic and thermal Energies= -3950.987856

Sum of electronic and thermal Enthalpies= -3950.986912

Sum of electronic and thermal Free Energies= -3951.118811

O	-2.12828200	6.06770400	-2.49252800
C	-1.65618400	6.73328500	-1.31590800
C	-2.13505000	5.84519100	-0.15142400
H	-0.55672500	6.79229700	-1.35609300
H	-2.07178900	7.74900500	-1.28395600
H	-3.12468500	6.18130700	0.20777300
N	-2.31245600	4.56194500	-0.85574700
C	-2.42170200	4.77281500	-2.19192300
O	-2.75047200	3.99006400	-3.06705300
C	-2.79556700	3.40441100	-0.15092300
H	-2.27276200	3.29585600	0.80206200
C	-4.19613100	3.12248000	-0.15418100
H	-4.49826200	2.40377100	0.61830500
C	-5.12521700	3.43981700	-1.10469200
H	-6.14810400	3.06989100	-1.01606100
H	-4.95097500	4.20153300	-1.86529200
Cu	-4.00379100	2.07140900	-3.16676000
P	-5.05202900	2.20440300	-5.12353100
C	-4.91240200	3.88350500	-5.94438400
C	-6.91535600	1.91686300	-5.03233300
C	-4.40295600	0.95399200	-6.35502100
C	-5.49496400	4.95957600	-5.00392400

C	-3.44791600	4.21094900	-6.30321600
H	-5.51431900	3.85630800	-6.87141600
C	-7.35229900	0.48501300	-5.39327000
C	-7.39927400	2.26368100	-3.60797100
H	-7.38322400	2.61535100	-5.75009400
C	-4.77909800	1.19124800	-7.82730000
C	-2.88816300	0.71493600	-6.19009900
H	-4.88684100	0.02007200	-6.02259000
C	-5.31508200	6.38012700	-5.55364100
H	-4.97775900	4.87696200	-4.03370200
H	-6.56411700	4.77097500	-4.81961300
C	-3.28814500	5.63915700	-6.84262900
H	-2.83107400	4.09420400	-5.39852200
H	-3.07027400	3.49885300	-7.05138300
C	-8.86500400	0.28654600	-5.21919600
H	-6.82130300	-0.22919200	-4.74344500
H	-7.07577400	0.23918600	-6.42910900
C	-8.91088300	2.07526400	-3.44390800
H	-6.86473800	1.60807800	-2.89822600
H	-7.11040200	3.28988100	-3.33864400
C	-4.29755000	0.03073100	-8.71069600
H	-4.31393800	2.12633800	-8.18259200
H	-5.86758400	1.32698800	-7.93593300
C	-2.41441500	-0.43827000	-7.08345400
H	-2.32459400	1.62730700	-6.44473900
H	-2.67056600	0.50096700	-5.13262400

C	-3.84515100	6.67777200	-5.86422200
H	-5.71528600	7.10875000	-4.82814000
H	-5.91406900	6.49390400	-6.47661500
H	-2.22270600	5.83529900	-7.05132300
H	-3.81929900	5.72848700	-7.80910100
C	-9.33112900	0.64734100	-3.80518000
H	-9.12705100	-0.75853700	-5.45691300
H	-9.40079800	0.91947800	-5.95097000
H	-9.20590200	2.31704300	-2.40874500
H	-9.44274700	2.79123300	-4.09798900
C	-2.79008600	-0.20542600	-8.55231100
H	-4.54842600	0.22987000	-9.76630700
H	-4.84152200	-0.88867200	-8.42528000
H	-1.32474000	-0.57276600	-6.97876500
H	-2.88229600	-1.37729400	-6.73526000
H	-3.73623400	7.69514000	-6.27721800
H	-3.26218900	6.64504300	-4.92669900
H	-10.42414800	0.52779300	-3.71649000
H	-8.87340000	-0.05539900	-3.08571600
H	-2.46805300	-1.05890800	-9.17239700
H	-2.24497900	0.68019800	-8.92877600
C	-1.18733400	5.78277600	1.02595300
C	0.13001500	5.32749000	0.85974500
C	-1.61506200	6.17229400	2.30169700
C	1.00122600	5.26428500	1.94867900
H	0.46780100	4.99896500	-0.12447000

C	-0.74207800	6.11621100	3.39415800
H	-2.64294100	6.51780400	2.44302100
C	0.56792900	5.66097400	3.22000600
H	2.02215200	4.90078700	1.80537200
H	-1.08939700	6.42386500	4.38371200
H	1.25005600	5.61186500	4.07259400
C	-2.08240100	1.51162900	-1.07649600
N	-3.04354400	0.97816100	-1.83839800
C	-4.63974000	-0.88887900	-2.14954800
C	-3.97190400	-1.41229900	-3.26867500
C	-5.97380600	-1.26284300	-1.93347500
C	-4.61731800	-2.29129400	-4.14167900
H	-2.93854300	-1.10950700	-3.44767400
C	-6.62258600	-2.14985700	-2.80059100
H	-6.51449700	-0.84965400	-1.07679900
C	-5.94624200	-2.66779900	-3.90901200
H	-4.08084000	-2.68692700	-5.00819200
H	-7.66452100	-2.42490400	-2.61679900
H	-6.45337100	-3.35253400	-4.59354600
C	-3.91859400	0.02248700	-1.17542800
H	-4.67380900	0.52463100	-0.54205600
H	-3.33124100	-0.61845100	-0.48367200
H	-1.94050300	1.06792800	-0.07794700
C	-0.79701800	1.98377500	-1.65871100
C	0.26417600	2.32334400	-0.80062500
C	-0.56911600	2.04029900	-3.04518600

C 1.50462800 2.72043400 -1.30522600

H 0.10844600 2.28499100 0.28108700

C 0.66732100 2.44106200 -3.55324700

H -1.37831100 1.77206200 -3.72321400

C 1.71212300 2.78784800 -2.68775100

H 2.31262200 2.97968800 -0.61531600

H 0.81741500 2.48126300 -4.63573400

H 2.68053000 3.10075200 -3.08677600

TS-III'-IV *cis (S,S,S)* conf3

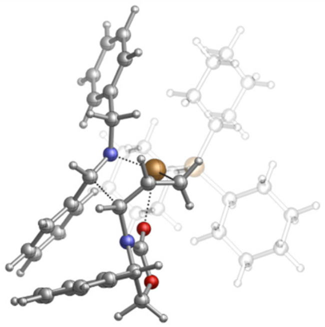

B3LYP-D3/def2-SVP-CPCM(toluene)

Zero-point correction= 0.936949 (Hartree/Particle)

Thermal correction to Energy= 0.984425

Thermal correction to Enthalpy= 0.985369

Thermal correction to Gibbs Free Energy= 0.853943

Sum of electronic and zero-point Energies= -3951.031304

Sum of electronic and thermal Energies= -3950.983828

Sum of electronic and thermal Enthalpies= -3950.982884

Sum of electronic and thermal Free Energies= -3951.114310

O -9.44369200 2.58291500 1.90438300

C	-9.61424800	2.59550600	3.32569400
C	-8.56375300	1.59820400	3.85305600
H	-9.42923700	3.61567300	3.69818000
H	-10.64471000	2.30440500	3.56864000
H	-9.00376600	0.58859300	3.94657300
N	-7.62014500	1.59000600	2.72229000
C	-8.22520400	2.06026400	1.59921600
O	-7.82316300	2.03654700	0.45004400
C	-6.45131900	0.74612700	2.74500600
H	-5.95102500	0.82917600	3.71386000
C	-6.56562500	-0.58231300	2.20841000
H	-5.72458300	-1.23723300	2.46865500
C	-7.42495500	-1.02078000	1.24357500
H	-7.34189700	-2.03440400	0.84442600
H	-8.33340800	-0.47291400	0.98620000
Cu	-6.50123600	0.47609000	-0.64668400
P	-7.56097100	0.20167200	-2.59337300
C	-7.17780000	1.71975000	-3.62895300
C	-9.44743800	0.11511900	-2.64951200
C	-6.96644300	-1.27259600	-3.58256300
C	-7.79997000	2.97378100	-2.97853500
C	-5.65175300	1.87873400	-3.76632100
H	-7.62429300	1.57988200	-4.62968200
C	-10.02883200	-1.30262700	-2.81123200
C	-10.03426700	0.77468000	-1.38431800
H	-9.75404900	0.70620200	-3.53215300

C	-7.14268400	-1.17135700	-5.10739500
C	-5.52945800	-1.67674600	-3.20090200
H	-7.61401500	-2.09018700	-3.22276700
C	-7.37847500	4.26910900	-3.68511800
H	-7.49626600	3.01482000	-1.91841300
H	-8.89833000	2.90031800	-2.98206500
C	-5.24899500	3.18013600	-4.46750300
H	-5.21124400	1.85392000	-2.75581900
H	-5.21706900	1.02188800	-4.30107200
C	-11.56530800	-1.28272900	-2.82321200
H	-9.68016000	-1.93743900	-1.97537600
H	-9.67050300	-1.77364000	-3.73884800
C	-11.56562100	0.80181200	-1.40342300
H	-9.69167700	0.20091400	-0.50715600
H	-9.63089500	1.78355300	-1.23579400
C	-6.71416700	-2.47169300	-5.80330100
H	-6.52492700	-0.34375300	-5.49517900
H	-8.18712500	-0.92668200	-5.36376700
C	-5.11647500	-2.97344500	-3.90618100
H	-4.81557700	-0.87949400	-3.46194800
H	-5.45817700	-1.78849800	-2.10779000
C	-5.85470400	4.40063500	-3.76925400
H	-7.81402300	5.13365100	-3.15618800
H	-7.79853000	4.28152000	-4.70812000
H	-4.14868700	3.25380800	-4.49862000
H	-5.59282600	3.15349400	-5.51829200

C	-12.14385800	-0.60614000	-1.57606200
H	-11.94819900	-2.31292300	-2.91870400
H	-11.90675800	-0.73701700	-3.72227300
H	-11.93662700	1.26518800	-0.47371300
H	-11.91043000	1.44431200	-2.23505200
C	-5.27901200	-2.86061700	-5.42666600
H	-6.81262100	-2.36319600	-6.89671500
H	-7.40111700	-3.28510200	-5.50454700
H	-4.07491000	-3.21925200	-3.64517400
H	-5.74413300	-3.80563500	-3.53576100
H	-5.57409600	5.32929300	-4.29399500
H	-5.43831300	4.47582500	-2.74823100
H	-13.24507200	-0.57377400	-1.63229500
H	-11.89066400	-1.21134800	-0.68579200
H	-4.99692100	-3.80741300	-5.91738100
H	-4.58296900	-2.08937600	-5.80599800
C	-7.93978800	1.97891000	5.17803200
C	-7.27702100	3.20721900	5.33160700
C	-8.00634000	1.10340300	6.26947100
C	-6.69430500	3.55112500	6.55274400
H	-7.20020500	3.88959700	4.48318400
C	-7.42817500	1.44770100	7.49663200
H	-8.51120500	0.13993900	6.15661500
C	-6.76979200	2.67211800	7.64047400
H	-6.17649100	4.50824800	6.65585300
H	-7.48863000	0.75449200	8.33942100

H	-6.31372600	2.94152400	8.59650900
N	-4.83086300	0.81361600	0.34424700
C	-3.90225600	-0.29880300	0.28383900
H	-3.17216100	-0.24529900	1.11999400
H	-4.41194400	-1.27506700	0.40792100
C	-3.11471400	-0.34052700	-1.01644200
C	-2.87857300	0.82864500	-1.75245600
C	-2.59957800	-1.55168200	-1.50178600
C	-2.16562500	0.78702500	-2.95447700
H	-3.28612800	1.76769200	-1.37478300
C	-1.87903900	-1.59839400	-2.69866400
H	-2.78592800	-2.47558900	-0.94533200
C	-1.66658700	-0.42807500	-3.43657500
H	-2.00510300	1.70841600	-3.52145700
H	-1.49428400	-2.55398600	-3.06528600
H	-1.11555500	-0.46396100	-4.38004600
C	-5.15090100	2.95910200	1.51421800
C	-5.06904000	3.68302200	2.71499100
C	-5.41788400	3.66751300	0.33045100
C	-5.26891000	5.06614300	2.73949400
H	-4.85416500	3.15081100	3.64541700
C	-5.62189600	5.04768000	0.34947000
H	-5.47323200	3.10901600	-0.60371100
C	-5.55355300	5.75629600	1.55565200
H	-5.20138400	5.60731700	3.68759100
H	-5.83705000	5.57534000	-0.58397600

H	-5.71349800	6.83757800	1.57157100
C	-4.88550300	1.48839700	1.50790300
H	-4.11467700	1.22233700	2.25116200

TS-III'-IV *cis (S,S,S)* conf4

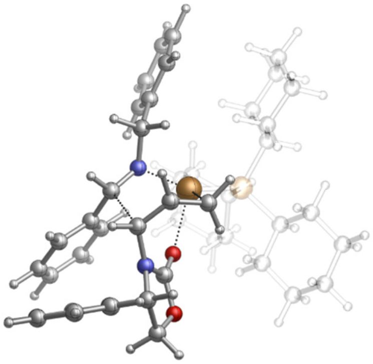

B3LYP-D3/def2-SVP-CPCM(toluene)

Zero-point correction= 0.937012 (Hartree/Particle)

Thermal correction to Energy= 0.984481

Thermal correction to Enthalpy= 0.985425

Thermal correction to Gibbs Free Energy= 0.852890

Sum of electronic and zero-point Energies= -3951.029482

Sum of electronic and thermal Energies= -3950.982013

Sum of electronic and thermal Enthalpies= -3950.981069

Sum of electronic and thermal Free Energies= -3951.113603

O	-9.28922500	2.52298700	1.91218300
C	-9.33926300	2.48527200	3.34213900
C	-8.21674500	1.50911400	3.74391500
H	-9.15433900	3.49877400	3.73277500
H	-10.33571200	2.15175400	3.66065200
H	-8.61315000	0.48113500	3.83195700

N	-7.36807000	1.57744100	2.54151100
C	-8.08083800	2.06191200	1.48914200
O	-7.77788800	2.09563500	0.30968700
C	-6.17033100	0.78198100	2.44481600
H	-5.59405500	0.85508600	3.37077000
C	-6.26053500	-0.51991200	1.85176000
H	-5.37328100	-1.14282500	2.02166100
C	-7.16396500	-0.95860500	0.92550400
H	-7.05757100	-1.94627700	0.47171500
H	-8.11805600	-0.45426200	0.76368100
Cu	-6.47048500	0.66767700	-0.94186000
P	-7.68260600	0.26922600	-2.75491500
C	-7.66477400	1.74392800	-3.92816400
C	-9.51691800	-0.11385400	-2.51855100
C	-7.05139000	-1.18313300	-3.76994600
C	-8.71311300	2.82293700	-3.59229400
C	-6.25965700	2.38197400	-3.91750700
H	-7.88368000	1.35563600	-4.94001100
C	-9.85379200	-1.60119400	-2.28974900
C	-10.08969400	0.71147100	-1.34746700
H	-10.01169800	0.19623700	-3.45791500
C	-5.92067000	-0.86703600	-4.76634000
C	-6.57814500	-2.29982200	-2.81446400
H	-7.91386900	-1.55022900	-4.35519500
C	-8.61335200	4.02312600	-4.54555400
H	-8.54996800	3.16919000	-2.55614200

H	-9.73238000	2.41146100	-3.63706800
C	-6.15429000	3.57838500	-4.86870100
H	-6.04831600	2.71460000	-2.88627900
H	-5.48241000	1.64494000	-4.15069200
C	-11.36854300	-1.81121200	-2.14130400
H	-9.35249600	-1.94914500	-1.36917600
H	-9.48219600	-2.22836100	-3.11339800
C	-11.60151500	0.51590900	-1.19288000
H	-9.58396400	0.39183400	-0.42355700
H	-9.84991500	1.77662800	-1.44248200
C	-5.51754700	-2.12430800	-5.55255800
H	-5.04227500	-0.48882200	-4.21773400
H	-6.22490900	-0.08126600	-5.47421200
C	-6.18530000	-3.56751300	-3.58245300
H	-5.70883700	-1.91556900	-2.25130100
H	-7.34430700	-2.53598100	-2.06291500
C	-7.21054300	4.63893500	-4.54417700
H	-9.36794400	4.77829900	-4.26784300
H	-8.86331500	3.69142600	-5.57045600
H	-5.13998200	4.00847800	-4.81110700
H	-6.28998200	3.23097100	-5.90984800
C	-11.95472800	-0.96326000	-1.00757000
H	-11.57914000	-2.88096600	-1.97301100
H	-11.86304900	-1.53995800	-3.09241700
H	-11.96404600	1.11141200	-0.33813400
H	-12.11558300	0.90683000	-2.09077600

C	-5.10338900	-3.27378600	-4.62701700
H	-4.69421700	-1.87641300	-6.24370800
H	-6.36991300	-2.44669300	-6.17938100
H	-5.83864000	-4.34121200	-2.87648900
H	-7.08226900	-3.97829500	-4.08258600
H	-7.15542800	5.47771800	-5.25837000
H	-7.00161600	5.06023300	-3.54319000
H	-13.04766200	-1.10067800	-0.94834200
H	-11.53946400	-1.31352700	-0.04442100
H	-4.87728500	-4.17893700	-5.21589400
H	-4.17370800	-2.99076600	-4.10621500
C	-7.50242700	1.86466500	5.02939600
C	-6.85512600	3.10261500	5.16945500
C	-7.47152600	0.95834300	6.09697200
C	-6.19123700	3.42579000	6.35424400
H	-6.85367800	3.80856900	4.33711700
C	-6.81219800	1.28197200	7.28832800
H	-7.96367500	-0.01273000	5.99335200
C	-6.16955700	2.51625100	7.41902200
H	-5.68600500	4.39075100	6.44646300
H	-6.79688600	0.56498300	8.11307200
H	-5.65009200	2.76944500	8.34673300
N	-4.74142500	1.04813800	-0.08499300
C	-3.79491900	-0.03016800	-0.29236300
H	-2.93494100	0.06295900	0.40528500
H	-4.23760300	-1.02315500	-0.07571700

C	-3.23665500	-0.06121400	-1.70629000
C	-3.21882900	1.09272400	-2.50270300
C	-2.69123300	-1.24332700	-2.23005700
C	-2.67294200	1.06676100	-3.78968700
H	-3.66265400	2.00375100	-2.09968200
C	-2.13460600	-1.27147400	-3.51259100
H	-2.70989100	-2.15661000	-1.62707200
C	-2.12745600	-0.11562800	-4.30136700
H	-2.67974500	1.97491200	-4.39886800
H	-1.71573800	-2.20264700	-3.90322100
H	-1.70415000	-0.13892000	-5.30887000
C	-5.03952300	3.10733000	1.23563200
C	-4.83291800	3.76815400	2.45834500
C	-5.48699200	3.86590400	0.14040400
C	-5.08143100	5.13684600	2.59102800
H	-4.48034900	3.19636200	3.32108500
C	-5.73828100	5.23245700	0.26808700
H	-5.64362100	3.35972200	-0.81155600
C	-5.54177600	5.87698900	1.49589500
H	-4.91401000	5.62765100	3.55401000
H	-6.09030000	5.79969200	-0.59829200
H	-5.73859400	6.94759600	1.59632600
C	-4.71171500	1.65705400	1.11137400
H	-3.87906500	1.37546800	1.77790500

TS-III'-IV *cis (S,S,S)* conf5

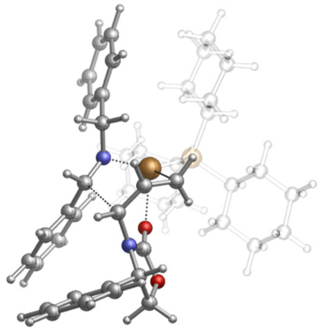

B3LYP-D3/def2-SVP-CPCM(toluene)

Zero-point correction= 0.936991 (Hartree/Particle)

Thermal correction to Energy= 0.984466

Thermal correction to Enthalpy= 0.985410

Thermal correction to Gibbs Free Energy= 0.852590

Sum of electronic and zero-point Energies= -3951.029489

Sum of electronic and thermal Energies= -3950.982015

Sum of electronic and thermal Enthalpies= -3950.981070

Sum of electronic and thermal Free Energies= -3951.113890

O	-9.28832100	2.47820400	1.90632900
C	-9.36765000	2.46962200	3.33543600
C	-8.28313300	1.46860500	3.77840700
H	-9.16028900	3.48460000	3.71063900
H	-10.37974900	2.17194100	3.63981600
H	-8.71212300	0.45498100	3.87913000
N	-7.40995800	1.48679900	2.59183000
C	-8.08662600	1.97250500	1.51647600
O	-7.75951600	1.97449300	0.34301600
C	-6.23556900	0.65357900	2.53453200
H	-5.67517900	0.72793600	3.47010000

C	-6.35434700	-0.65666200	1.96553400
H	-5.49020000	-1.30274800	2.16533700
C	-7.25274700	-1.08602200	1.02998600
H	-7.16859400	-2.08559000	0.59800800
H	-8.18771100	-0.55628500	0.84016100
Cu	-6.47473000	0.47981200	-0.85424700
P	-7.66699000	0.09517800	-2.68361100
C	-7.58304300	1.55911900	-3.86746400
C	-9.51584600	-0.22939900	-2.47366400
C	-7.06520500	-1.38597500	-3.67489800
C	-8.59843900	2.67509400	-3.55138200
C	-6.15715700	2.14892500	-3.84159600
H	-7.80181600	1.17185000	-4.87972500
C	-9.89826000	-1.70358300	-2.23100800
C	-10.08345900	0.62683400	-1.32239900
H	-9.98602000	0.08257100	-3.42504500
C	-5.91599400	-1.11426700	-4.66319600
C	-6.63431900	-2.50416600	-2.70126300
H	-7.93135200	-1.73556700	-4.26534300
C	-8.44456600	3.86713000	-4.50759500
H	-8.43870100	3.01977200	-2.51414200
H	-9.63029000	2.29815700	-3.60977900
C	-5.99850400	3.33703900	-4.79578200
H	-5.94779600	2.47836200	-2.80905800
H	-5.40266600	1.38476500	-4.06166500
C	-11.42028500	-1.86953200	-2.10303400

H	-9.42059200	-2.05262100	-1.29833900
H	-9.53215700	-2.35215300	-3.04041000
C	-11.60226500	0.47518500	-1.18838700
H	-9.60060200	0.30559200	-0.38694400
H	-9.81315700	1.68358400	-1.42739200
C	-5.54199100	-2.39186200	-5.43072300
H	-5.03204300	-0.75576800	-4.11029500
H	-6.19020300	-0.32823000	-5.38299700
C	-6.26811600	-3.79134100	-3.44999100
H	-5.76144000	-2.13748600	-2.13192000
H	-7.41566100	-2.71029200	-1.95634800
C	-7.02167800	4.43455200	-4.48898800
H	-9.17666800	4.64871200	-4.24307000
H	-8.69157700	3.54003000	-5.53470000
H	-4.97081500	3.73221200	-4.72640300
H	-6.13252500	2.99032600	-5.83738600
C	-11.99942200	-0.99092100	-0.98927500
H	-11.66308000	-2.93076400	-1.92445000
H	-11.89281400	-1.59717100	-3.06493500
H	-11.96054200	1.09204200	-0.34709300
H	-12.09182400	0.86812200	-2.09900800
C	-5.16831600	-3.54035000	-4.48709700
H	-4.70620200	-2.17596200	-6.11765900
H	-6.39777000	-2.69798300	-6.06104400
H	-5.94976100	-4.56534100	-2.73113900
H	-7.17087000	-4.18387000	-3.95437100

H	-6.92803700	5.26832800	-5.20502100
H	-6.81192200	4.85200900	-3.48655400
H	-13.09652200	-1.09723100	-0.94491700
H	-11.60837200	-1.33975900	-0.01552900
H	-4.96139500	-4.45877900	-5.06231300
H	-4.23630200	-3.27606100	-3.96058300
C	-7.58232500	1.82881200	5.07005100
C	-6.90201200	3.05022300	5.19822300
C	-7.59626700	0.94293800	6.15499100
C	-6.24996900	3.37743300	6.38846200
H	-6.86537000	3.73928400	4.35263900
C	-6.94880800	1.27078900	7.35169500
H	-8.11427200	-0.01556800	6.06089900
C	-6.27324400	2.48857500	7.47054800
H	-5.71871900	4.32917300	6.47141100
H	-6.96858400	0.56984900	8.19002600
H	-5.76302000	2.74488700	8.40252100
N	-4.75129700	0.82457600	0.02873900
C	-3.83552000	-0.28663000	-0.13902700
H	-2.98541800	-0.20541700	0.57208900
H	-4.31276800	-1.26069100	0.08982100
C	-3.25380000	-0.36449900	-1.54160900
C	-3.18683400	0.77129700	-2.36118500
C	-2.73523200	-1.57327100	-2.03065000
C	-2.61848900	0.70185900	-3.63680100
H	-3.61007300	1.70344400	-1.98521700

C	-2.15625600	-1.64506200	-3.30152600
H	-2.79235000	-2.47259400	-1.40937600
C	-2.09951700	-0.50677800	-4.11354500
H	-2.58666100	1.59673200	-4.26456400
H	-1.75825300	-2.59625400	-3.66477700
H	-1.65848200	-0.56389700	-5.11205600
C	-5.00968700	2.91818600	1.30291300
C	-4.80742600	3.59655400	2.51670100
C	-5.41098000	3.66830600	0.18420200
C	-5.01603300	4.97458300	2.61781800
H	-4.49043300	3.03153900	3.39750400
C	-5.62245700	5.04412400	0.28029600
H	-5.56394700	3.14835600	-0.76083400
C	-5.43100200	5.70659900	1.49932700
H	-4.85306300	5.47902900	3.57449000
H	-5.93920000	5.60447000	-0.60400400
H	-5.59660500	6.78449400	1.57505200
C	-4.72517000	1.45623900	1.21333200
H	-3.91470300	1.16234400	1.90154000

TS-III'-IV *cis (S,S,S)* conf6

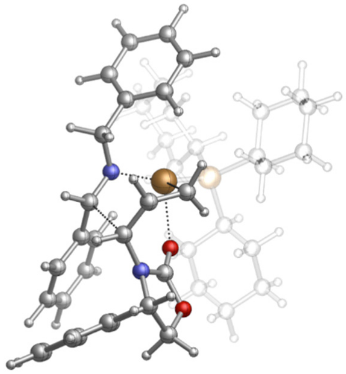

B3LYP-D3/def2-SVP-CPCM(toluene)

Zero-point correction= 0.937699 (Hartree/Particle)

Thermal correction to Energy= 0.985204

Thermal correction to Enthalpy= 0.986148

Thermal correction to Gibbs Free Energy= 0.855011

Sum of electronic and zero-point Energies= -3951.026885

Sum of electronic and thermal Energies= -3950.979380

Sum of electronic and thermal Enthalpies= -3950.978436

Sum of electronic and thermal Free Energies= -3951.109573

O	-8.21255500	3.88937300	0.90566600
C	-8.09133400	4.54999800	2.17053500
C	-7.83602400	3.41197400	3.17706900
H	-7.24471700	5.25340900	2.12570900
H	-9.01377600	5.10899700	2.37643900
H	-8.79257200	3.04349200	3.59034700
N	-7.30060400	2.37738700	2.27229500
C	-7.65800900	2.64723300	0.99354800
O	-7.55942700	1.93541800	0.00940100
C	-6.94925300	1.07860400	2.76495600
H	-6.33623300	1.15297000	3.66518900

C	-7.89179600	0.01481700	2.69943800
H	-7.66034700	-0.81802600	3.37703400
C	-8.92092300	-0.17494100	1.81765300
H	-9.54366700	-1.06996800	1.88562100
H	-9.24680600	0.58995400	1.11438300
Cu	-7.24299100	-0.28050600	-0.64219900
P	-8.18348900	-0.15383300	-2.66599100
C	-8.37728000	1.60155800	-3.32404100
C	-9.93819100	-0.85148800	-2.72110000
C	-7.26945600	-1.09931700	-4.01503500
C	-9.57097800	2.34561500	-2.68742200
C	-7.08333200	2.41402200	-3.09124000
H	-8.56674100	1.50862100	-4.41067600
C	-10.05046600	-2.33880200	-3.10627800
C	-10.60414700	-0.62958600	-1.34713400
H	-10.47927300	-0.26684200	-3.48720000
C	-6.16976700	-0.32413200	-4.76329200
C	-6.66751500	-2.37816500	-3.40041500
H	-8.03164600	-1.37941100	-4.76342900
C	-9.68202800	3.78917900	-3.19877000
H	-9.42838300	2.36300200	-1.59480700
H	-10.51610400	1.82054600	-2.89229700
C	-7.20139200	3.85505100	-3.60241400
H	-6.89004800	2.43371500	-2.01118500
H	-6.21705500	1.92992800	-3.55857300
C	-11.51205500	-2.80999700	-3.09816200

H	-9.47835100	-2.94375300	-2.38545200
H	-9.61269500	-2.52722300	-4.09768800
C	-12.06217200	-1.10045100	-1.32624600
H	-10.02059500	-1.18866700	-0.59588600
H	-10.54220600	0.42465500	-1.04310100
C	-5.49952400	-1.20432300	-5.82945100
H	-5.40207600	0.01047500	-4.04305600
H	-6.57893200	0.57789500	-5.24206200
C	-5.99417800	-3.26181000	-4.45495500
H	-5.92827300	-2.07634900	-2.63792400
H	-7.42383600	-2.95568700	-2.85443100
C	-8.38975200	4.57528600	-2.95865800
H	-10.53568600	4.28679100	-2.70773700
H	-9.90626800	3.77652800	-4.28208500
H	-6.26185900	4.39409800	-3.39164100
H	-7.32442200	3.85348600	-4.70215300
C	-12.17739500	-2.57135500	-1.73847300
H	-11.55668900	-3.87890300	-3.36768500
H	-12.07432700	-2.26534600	-3.87924000
H	-12.48949500	-0.94432200	-0.32141800
H	-12.65624700	-0.47811300	-2.02112500
C	-4.92066400	-2.48897400	-5.22720400
H	-4.71157800	-0.62786200	-6.34307800
H	-6.24760900	-1.46848100	-6.59983600
H	-5.56105900	-4.15042300	-3.96770700
H	-6.75989500	-3.63237800	-5.16198200

H	-8.48318800	5.60229900	-3.35122400
H	-8.21675600	4.65810000	-1.87084600
H	-13.23362700	-2.88815500	-1.76342300
H	-11.67740400	-3.19848000	-0.97706500
H	-4.47681800	-3.11909000	-6.01644000
H	-4.09999700	-2.22484600	-4.53481400
C	-6.91910900	3.76683000	4.32695000
C	-5.63275700	4.27744600	4.09146300
C	-7.33778000	3.57141200	5.64956300
C	-4.78520900	4.58746000	5.15671700
H	-5.28654100	4.41614800	3.06619600
C	-6.49282400	3.88558900	6.71997200
H	-8.33368500	3.16363800	5.84357200
C	-5.21384900	4.39354300	6.47575400
H	-3.78466800	4.97997700	4.95723300
H	-6.83418100	3.72792400	7.74619000
H	-4.55060100	4.63633300	7.30987500
C	-5.26402500	0.13131500	1.39078800
N	-5.82467100	-0.79799500	0.62529800
C	-5.85110500	-2.12939200	1.23511300
H	-4.82471700	-2.42294200	1.52529700
H	-6.43905200	-2.10886900	2.17216800
C	-6.41732300	-3.20681000	0.33844000
C	-7.80287100	-3.42671900	0.27604400
C	-5.57549100	-4.02316500	-0.43009500
C	-8.33095700	-4.44965900	-0.51673000

H	-8.46413300	-2.78439100	0.86003400
C	-6.09885800	-5.04628300	-1.22797400
H	-4.49445200	-3.85990900	-0.39548100
C	-7.47943400	-5.26463300	-1.27146100
H	-9.41103800	-4.61550000	-0.54046100
H	-5.42619800	-5.67730300	-1.81459500
H	-7.89063800	-6.06546100	-1.89138000
C	-4.66422100	1.37122400	0.84431800
C	-4.05888600	2.29317900	1.71757600
C	-4.60877100	1.63832800	-0.53610000
C	-3.44203600	3.44902500	1.23434500
H	-4.08773000	2.10414800	2.79358600
C	-4.00190100	2.79619900	-1.02233600
H	-5.05594400	0.92744600	-1.23235000
C	-3.41772300	3.71332800	-0.14030000
H	-2.97925000	4.14958000	1.93512900
H	-3.98545700	2.98328000	-2.09948300
H	-2.94189000	4.62097000	-0.52044300
H	-4.78419200	-0.19855500	2.32349300

TS-III’-IV *trans (S,S,S)* conf2

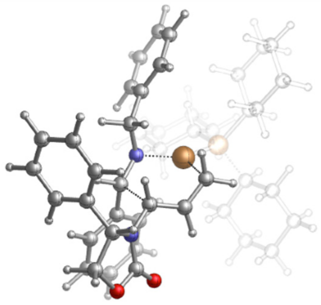

B3LYP-D3/def2-SVP-CPCM(toluene)

Zero-point correction= 0.936459 (Hartree/Particle)

Thermal correction to Energy= 0.984373

Thermal correction to Enthalpy= 0.985317

Thermal correction to Gibbs Free Energy= 0.850530

Sum of electronic and zero-point Energies= -3951.034061

Sum of electronic and thermal Energies= -3950.986148

Sum of electronic and thermal Enthalpies= -3950.985203

Sum of electronic and thermal Free Energies= -3951.119991

O	-0.02406400	6.77527700	-0.19506300
C	-0.03364400	6.04358500	1.02932700
C	-1.35345100	5.25250900	0.99941200
H	0.84426200	5.37784500	1.06012900
H	0.02215000	6.74362800	1.87390200
H	-2.15816600	5.84792200	1.47155500
N	-1.59018800	5.20716200	-0.44833700
C	-0.89833600	6.20507700	-1.08486000
O	-1.00252900	6.59948300	-2.22170000
C	-2.70799700	4.48233300	-0.98271900
H	-3.19872600	3.92777200	-0.17878600
C	-3.59392000	5.07159600	-1.93439000
H	-3.18787600	5.86646800	-2.56532600
C	-4.84211000	4.55918100	-2.22946500
H	-5.35337300	3.90302500	-1.51322500
H	-5.49048300	5.10203800	-2.92127500
Cu	-4.32816700	2.79233600	-3.58846400

P	-5.42876900	2.52043300	-5.50174800
C	-5.36525800	3.97958500	-6.67290900
C	-7.27291700	2.21493600	-5.26573700
C	-4.76860500	1.04957400	-6.45110800
C	-6.12165700	5.19355600	-6.09783600
C	-3.90554300	4.36447500	-6.98323300
H	-5.86382500	3.67080400	-7.60915700
C	-7.67977100	0.73012400	-5.28134300
C	-7.71142900	2.86520900	-3.93462300
H	-7.79087300	2.72164300	-6.10000100
C	-5.17072200	0.95702900	-7.93154100
C	-3.24683600	0.87027900	-6.27521600
H	-5.23021600	0.20412700	-5.91316900
C	-6.02393200	6.41686800	-7.01958800
H	-5.68911700	5.44331400	-5.11319600
H	-7.18119100	4.94874900	-5.92740800
C	-3.81307800	5.58850000	-7.90290900
H	-3.39309000	4.58919500	-6.03162200
H	-3.36794900	3.51896700	-7.43690700
C	-9.18048100	0.55060700	-5.00948700
H	-7.10887300	0.18811200	-4.50994200
H	-7.43082400	0.26658200	-6.24773600
C	-9.21094800	2.68948500	-3.67253200
H	-7.13477200	2.39435400	-3.11772100
H	-7.44351400	3.93193400	-3.91526700
C	-4.70326700	-0.37362000	-8.54080000

H	-4.70724800	1.78920000	-8.48993500
H	-6.26095500	1.06977300	-8.05190200
C	-2.78833400	-0.46199500	-6.88058800
H	-2.70456100	1.69474000	-6.76773900
H	-2.98746100	0.92842900	-5.20701600
C	-4.56713100	6.78593300	-7.31678800
H	-6.55442200	7.26833400	-6.56144800
H	-6.54481500	6.19562100	-7.96950600
H	-2.75368000	5.84269700	-8.07386300
H	-4.24041500	5.33410600	-8.89043100
C	-9.60658300	1.20973200	-3.69369500
H	-9.42455300	-0.52521700	-4.99858600
H	-9.75595700	0.99514300	-5.84269100
H	-9.47618600	3.14889300	-2.70547200
H	-9.78268700	3.23406800	-4.44687700
C	-3.19400300	-0.57794800	-8.35571000
H	-4.96970300	-0.41362400	-9.61043500
H	-5.24598700	-1.20234700	-8.04972900
H	-1.69639900	-0.56964000	-6.77039000
H	-3.24574700	-1.29154100	-6.30976300
H	-4.51897300	7.64789000	-8.00294900
H	-4.07353500	7.09981000	-6.37870300
H	-10.69259000	1.09508300	-3.53889400
H	-9.10770800	0.69221000	-2.85489600
H	-2.88727200	-1.55518800	-8.76482800
H	-2.65263600	0.19029700	-8.93859700

C	-1.33139200	3.88980000	1.66468400
C	-0.31869200	2.95420500	1.39896100
C	-2.37972500	3.51795300	2.51838400
C	-0.35506600	1.68008000	1.96946000
H	0.50467400	3.21610600	0.73211800
C	-2.42184000	2.24239500	3.09128900
H	-3.17907000	4.23398100	2.72870900
C	-1.40991200	1.31859900	2.81559000
H	0.44033400	0.96425400	1.74789200
H	-3.24956100	1.96999200	3.75080500
H	-1.44124900	0.31988500	3.25820000
C	-2.00183500	2.76139500	-1.88321800
N	-3.06349800	2.03376300	-2.30056700
C	-4.67952700	0.23731300	-1.81968000
C	-4.26454300	-0.59763900	-2.87161300
C	-5.97168400	0.06786900	-1.30358300
C	-5.11221000	-1.58284800	-3.38194400
H	-3.26638300	-0.45530200	-3.29011400
C	-6.82380000	-0.92276000	-1.80783100
H	-6.31689900	0.72157500	-0.49710600
C	-6.39612000	-1.75318800	-2.84749200
H	-4.77115100	-2.22112700	-4.20169700
H	-7.82771800	-1.03953400	-1.39139600
H	-7.06266000	-2.52117800	-3.24813900
C	-3.73210200	1.27797100	-1.25565500
H	-4.30210400	1.93537100	-0.56329800

H	-2.98731500	0.76267700	-0.61193500
H	-1.51429500	2.42301400	-0.95639600
C	-1.02540800	3.29291700	-2.86950300
C	0.29031900	3.58224700	-2.46270100
C	-1.36440000	3.54780800	-4.20993900
C	1.21533100	4.14142500	-3.34667700
H	0.58810400	3.37105700	-1.43462400
C	-0.44415300	4.10262700	-5.09869700
H	-2.36606000	3.29887600	-4.56602800
C	0.85164900	4.41244200	-4.66934700
H	2.22709800	4.36787400	-2.99972000
H	-0.74240200	4.29536500	-6.13314600
H	1.57275800	4.85475700	-5.36145700

TS-III'-IV *trans (S,S,S)* conf3

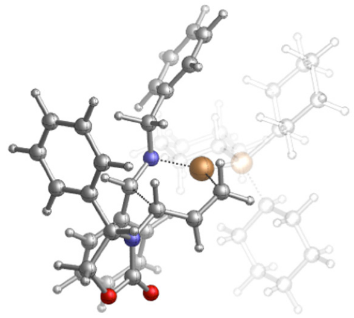

B3LYP-D3/def2-SVP-CPCM(toluene)

Zero-point correction= 0.936459 (Hartree/Particle)

Thermal correction to Energy= 0.984373

Thermal correction to Enthalpy= 0.985317

Thermal correction to Gibbs Free Energy= 0.850530

Sum of electronic and zero-point Energies= -3951.034061

Sum of electronic and thermal Energies= -3950.986148

Sum of electronic and thermal Enthalpies= -3950.985203

Sum of electronic and thermal Free Energies= -3951.119991

O	-0.00147600	6.73280500	-0.15896400
C	-0.00018700	5.97968700	1.05236400
C	-1.31715400	5.18387000	1.01762800
H	0.88070300	5.31736900	1.06584300
H	0.05840300	6.66504500	1.90872500
H	-2.12025700	5.76626900	1.50834500
N	-1.56635200	5.16618100	-0.42863900
C	-0.88126600	6.17675800	-1.05222900
O	-0.99458600	6.59109400	-2.18109100
C	-2.68714300	4.44895900	-0.96702300
H	-3.17273000	3.88223400	-0.16848600
C	-3.57941100	5.05175200	-1.90417200
H	-3.17776900	5.85564100	-2.52640000
C	-4.82955500	4.54340300	-2.19785300
H	-5.33603900	3.87734400	-1.48737900
H	-5.48249000	5.09560900	-2.87786500
Cu	-4.32672100	2.79355300	-3.58406500
P	-5.44442800	2.54641600	-5.49058000
C	-5.38831300	4.01892100	-6.64529100
C	-7.28703200	2.24121200	-5.24207000
C	-4.79586000	1.08575300	-6.46327100
C	-6.13752100	5.22767700	-6.05018000

C	-3.93061700	4.40478900	-6.96382100
H	-5.89545700	3.72169500	-7.58067500
C	-7.69607500	0.75717600	-5.27049100
C	-7.71310300	2.87723500	-3.90010400
H	-7.81130200	2.75785500	-6.06623900
C	-5.21317700	1.01101000	-7.94050500
C	-3.27268300	0.90205000	-6.30513700
H	-5.25332300	0.23476600	-5.93055900
C	-6.04559800	6.46096400	-6.95916900
H	-5.69604500	5.46583400	-5.06662500
H	-7.19598300	4.98277800	-5.87334900
C	-3.84389300	5.63876000	-7.87067200
H	-3.40966200	4.61804900	-6.01420200
H	-3.39831900	3.56354700	-7.43146100
C	-9.19453800	0.57646500	-4.98735400
H	-7.11905000	0.20596800	-4.51023400
H	-7.45634600	0.30400900	-6.24408700
C	-9.21045300	2.70034000	-3.62665100
H	-7.12989600	2.39668300	-3.09359700
H	-7.44376200	3.94337800	-3.87127600
C	-4.75468900	-0.31348700	-8.56959200
H	-4.75367800	1.84865000	-8.49400900
H	-6.30433800	1.12726400	-8.04842600
C	-2.82285300	-0.42403900	-6.93036000
H	-2.73419000	1.73116800	-6.79395300
H	-3.00207500	0.94763700	-5.23908200

C	-4.59078500	6.83089800	-7.26493300
H	-6.57058700	7.30818900	-6.48704500
H	-6.57509900	6.25113500	-7.90691200
H	-2.78555300	5.89306600	-8.04791900
H	-4.28012300	5.39599400	-8.85723300
C	-9.60806600	1.22136100	-3.66056600
H	-9.43992700	-0.49911400	-4.98634800
H	-9.77678700	1.03104000	-5.81037100
H	-9.46649900	3.14939100	-2.65227900
H	-9.78839400	3.25404400	-4.38984100
C	-3.24402900	-0.52266400	-8.40245900
H	-5.03221100	-0.34088300	-9.63680400
H	-5.29394300	-1.14672300	-8.08235000
H	-1.73003000	-0.53478500	-6.83276400
H	-3.27582200	-1.25922200	-6.36424500
H	-4.54710100	7.70025700	-7.94199700
H	-4.08855300	7.13367800	-6.32778500
H	-10.69277300	1.10618900	-3.49728400
H	-9.10228200	0.69402700	-2.83205600
H	-2.94342700	-1.49581600	-8.82562700
H	-2.70722900	0.25108600	-8.98227600
C	-1.28425800	3.80802400	1.65509700
C	-0.26700200	2.88412100	1.36695400
C	-2.32782800	3.41219800	2.50386300
C	-0.29429000	1.59809800	1.91064400
H	0.55290000	3.16463700	0.70340900

C	-2.36097000	2.12462200	3.04982700
H	-3.13064300	4.11898100	2.73155100
C	-1.34456700	1.21271400	2.75186200
H	0.50459900	0.89177500	1.67181700
H	-3.18516300	1.83360600	3.70580400
H	-1.36885500	0.20474400	3.17342500
C	-1.98657500	2.74205000	-1.89818800
N	-3.05107900	2.01919100	-2.31664500
C	-4.66211800	0.21548900	-1.84638700
C	-4.25479300	-0.60642400	-2.91147600
C	-5.95073100	0.04008100	-1.32346800
C	-5.10644200	-1.58484800	-3.42820700
H	-3.25946900	-0.45929800	-3.33506900
C	-6.80677600	-0.94387000	-1.83408200
H	-6.29003600	0.68381500	-0.50653100
C	-6.38669400	-1.76137200	-2.88701200
H	-4.77133200	-2.21301600	-4.25816200
H	-7.80785500	-1.06544500	-1.41221700
H	-7.05631400	-2.52408600	-3.29257400
C	-3.71046800	1.24894200	-1.27638800
H	-4.27499400	1.89665200	-0.57055800
H	-2.96001700	0.72555900	-0.64591900
H	-1.49064900	2.39114600	-0.98054900
C	-1.01955000	3.28908100	-2.88522800
C	0.29815100	3.57842800	-2.48481600
C	-1.36949800	3.55874400	-4.21997600

C 1.21433900 4.15198300 -3.36871600

H 0.60430400 3.35593800 -1.46164900

C -0.45805000 4.12810200 -5.10867200

H -2.37265300 3.30964200 -4.57173800

C 0.83972000 4.43776100 -4.68523400

H 2.22780000 4.37813500 -3.02654600

H -0.76468300 4.33207200 -6.13850500

H 1.55392000 4.89132000 -5.37723000

TS-III'-IV *trans (S,S,S)* conf4

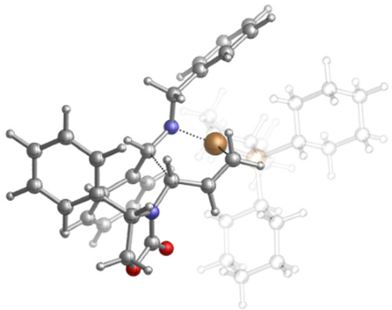

B3LYP-D3/def2-SVP-CPCM(toluene)

Zero-point correction= 0.936910 (Hartree/Particle)

Thermal correction to Energy= 0.984581

Thermal correction to Enthalpy= 0.985525

Thermal correction to Gibbs Free Energy= 0.852936

Sum of electronic and zero-point Energies= -3951.033454

Sum of electronic and thermal Energies= -3950.985783

Sum of electronic and thermal Enthalpies= -3950.984839

Sum of electronic and thermal Free Energies= -3951.117428

O -0.77329900 6.73445400 -1.02162300

C -1.07386200 6.78865300 0.37372400

C	-1.69657300	5.41641600	0.70983700
H	-0.15538300	7.00360200	0.93469200
H	-1.79666300	7.60187600	0.55258500
H	-2.60849700	5.55329500	1.31306900
N	-2.07752000	4.96976300	-0.63490600
C	-1.53510800	5.75753400	-1.61180300
O	-1.67619600	5.68565600	-2.81107800
C	-3.05249300	3.93186800	-0.85160700
H	-3.34033800	3.50023200	0.11096900
C	-4.16124000	4.26323400	-1.70841000
H	-3.99418700	5.07172600	-2.42584200
C	-5.31719000	3.53557600	-1.81679000
H	-5.58885400	2.78269600	-1.07086700
H	-6.12623000	3.88638900	-2.46217000
Cu	-4.38604400	2.02149300	-3.36673000
P	-5.33879200	2.33814100	-5.35031500
C	-4.88312200	3.98643600	-6.10490400
C	-7.19194900	2.33214500	-5.16538900
C	-4.96143400	1.02174500	-6.63329700
C	-5.51533000	5.15802500	-5.32800100
C	-3.34664400	4.11790700	-6.10423100
H	-5.25349400	4.01571100	-7.14375700
C	-8.01886300	2.48230600	-6.45137700
C	-7.63288900	1.09820500	-4.35575900
H	-7.37917600	3.21535300	-4.52887600
C	-4.98694500	1.46571100	-8.10700700

C	-3.64309700	0.29017200	-6.31001400
H	-5.77843800	0.29203600	-6.48425800
C	-5.02861400	6.51753900	-5.85027100
H	-5.24332400	5.06352500	-4.26383400
H	-6.61466000	5.11379900	-5.38312700
C	-2.87462300	5.47308200	-6.64022400
H	-2.99090200	4.00986000	-5.06725200
H	-2.88458200	3.29900200	-6.67867300
C	-9.52182600	2.52629000	-6.13592100
H	-7.82049500	1.62197000	-7.11441000
H	-7.71823600	3.38680800	-7.00555600
C	-9.13475400	1.13723400	-4.05304400
H	-7.40370700	0.17750900	-4.92056500
H	-7.05171200	1.03201500	-3.42245000
C	-4.76981000	0.26843600	-9.04473400
H	-4.18407300	2.20299800	-8.27991700
H	-5.93367200	1.97023800	-8.35225300
C	-3.42271600	-0.90178700	-7.24852400
H	-2.79785200	0.99412800	-6.41049900
H	-3.64739100	-0.03347700	-5.25796400
C	-3.49902500	6.62046600	-5.83962200
H	-5.47617500	7.32415000	-5.24536000
H	-5.39697700	6.65860400	-6.88359500
H	-1.77433000	5.52164200	-6.58886900
H	-3.15115100	5.56726000	-7.70714200
C	-9.96344400	1.29508800	-5.33385400

H	-10.10070700	2.60607000	-7.07140300
H	-9.74034000	3.43921200	-5.55179000
H	-9.42650100	0.22393600	-3.50874400
H	-9.34298900	1.98681600	-3.37670200
C	-3.46745600	-0.47373500	-8.72055200
H	-4.76853900	0.60985900	-10.09356000
H	-5.62209000	-0.42847900	-8.94186000
H	-2.46190000	-1.39082900	-7.01646300
H	-4.21192900	-1.65415100	-7.06313900
H	-3.17948200	7.59483900	-6.24604400
H	-3.12604400	6.56774900	-4.80136200
H	-11.03784500	1.35960600	-5.09309900
H	-9.83543400	0.39250800	-5.96015400
H	-3.34947300	-1.34901500	-9.38109900
H	-2.61097000	0.19436100	-8.92760400
C	-0.79232000	4.42721900	1.42912500
C	0.59420100	4.40301500	1.20884200
C	-1.35523500	3.47025300	2.28518500
C	1.39652800	3.44074000	1.82687400
H	1.05140400	5.12478600	0.52782700
C	-0.55624300	2.50010800	2.89962500
H	-2.43244300	3.48311800	2.47440500
C	0.82286700	2.48344200	2.67138400
H	2.47354300	3.43323700	1.64171200
H	-1.01220800	1.75892200	3.56085700
H	1.45035200	1.72848600	3.15156200

C	-2.22185700	2.17218000	-1.50501300
N	-3.21283300	1.35646300	-1.95444800
C	-4.98000200	-0.28783800	-1.51553400
C	-4.71671800	-1.07006800	-2.65419400
C	-6.26848900	-0.32549000	-0.96515200
C	-5.71222000	-1.86639800	-3.22388800
H	-3.71795900	-1.03212400	-3.09356400
C	-7.26907900	-1.12631200	-1.52991000
H	-6.49515700	0.28746300	-0.08785400
C	-6.99458200	-1.89796200	-2.66221300
H	-5.48813900	-2.46154800	-4.11345100
H	-8.26995200	-1.13563200	-1.09032800
H	-7.77778300	-2.51451700	-3.11080300
C	-3.88727700	0.58241800	-0.92715800
H	-4.33619500	1.22369800	-0.13638700
H	-3.16219900	-0.06809900	-0.39193500
H	-1.81907100	1.93132800	-0.50724700
C	-1.12315400	2.56329000	-2.43772100
C	0.08761200	3.04609900	-1.91177600
C	-1.22764800	2.44305500	-3.83277400
C	1.12808900	3.45778100	-2.74680100
H	0.21224200	3.09875000	-0.83109600
C	-0.19024600	2.84879500	-4.67292300
H	-2.13348900	2.01564100	-4.26444100
C	0.99028800	3.37509200	-4.13610800
H	2.05324800	3.84238800	-2.30816900

H	-0.30636100	2.75525000	-5.75651300
H	1.79952800	3.70287400	-4.79389100

(E) s-*cis*

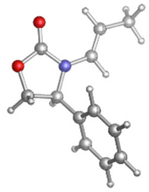

B3LYP-D3/def2-SVP-CPCM(toluene)

Zero-point correction= 0.229153 (Hartree/Particle)

Thermal correction to Energy= 0.242458

Thermal correction to Enthalpy= 0.243402

Thermal correction to Gibbs Free Energy= 0.187828

Sum of electronic and zero-point Energies= -669.623243

Sum of electronic and thermal Energies= -669.609938

Sum of electronic and thermal Enthalpies= -669.608994

Sum of electronic and thermal Free Energies= -669.664568

O	-0.05458200	7.14047700	0.80312300
C	-0.16285400	6.70856300	-0.55020900
C	-1.18450700	5.54985300	-0.52257600
H	0.82468700	6.39666700	-0.91672200
H	-0.53158400	7.54285700	-1.17044200
N	-1.85731200	5.82569600	0.74262100
C	-1.13171200	6.69243000	1.52150700
O	-1.34780200	7.06415800	2.65058700
C	-2.94811300	5.00008000	1.17663900

H	-3.45519600	4.57838700	0.30398300
C	-3.82507600	5.44245100	2.21452200
H	-3.42330300	6.15864000	2.93465100
H	-0.65777000	4.58049400	-0.46479500
C	-2.10593500	5.51447000	-1.72428900
C	-3.10277800	6.48918700	-1.89051300
C	-1.96226800	4.51579900	-2.69558100
C	-3.93501800	6.46649900	-3.01114300
H	-3.23267800	7.25813600	-1.12460200
C	-2.79109300	4.49449400	-3.82310800
H	-1.19750500	3.74445100	-2.56670300
C	-3.77924600	5.46987200	-3.98296500
H	-4.71159100	7.22693400	-3.12658100
H	-2.66751600	3.70983700	-4.57382900
H	-4.43175200	5.45184700	-4.85958000
C	-5.04017179	4.89007114	2.44901243
H	-5.30231670	5.02814793	3.47717333
H	-5.77241247	5.36204474	1.82775201
H	-5.00516630	3.84388863	2.22725408

References

1. Sheldrick, G. M. A short history of SHELX *Acta Cryst.* **2008**, *A64*, 112 – 122.
2. Bourhis, L. J.; Dolomanov, O.V.; Gildea, R.J.; Howard, J.A.K.; Puschmann, H. The anatomy of a comprehensive constrained restrained refinement program for the modern computing environment-Olex2 dissected. *Acta Cryst.* 2015, *A71*, 59 – 75.
3. Dolomanov, O. V.; Bourhis, L. J.; Gildea, R. J.; Howard, J. A. K.; Puschmann, H. OLEX2: a complete structure solution, refinement and analysis program. *J. Appl. Cryst.* 2009, *42*, 339 – 341.
4. Sheldrick, G.M. SHELXT-Inyegrated space-group and crystal-structure determination. *Acta Cryst.* 2015, *A71*, 3 – 8.
5. Rigaku OD. *CrysAlis PRO*. 2020, Rigaku Oxford Diffraction Ltd. Yarnton, Oxfordshire, England .
6. CYLview, 1.0b; Legault, C. Y., Université de Sherbrooke, 2009 (http://www.cylview.org)
7. Wang, Y.; Jin, X.; Yu, H.S.; Truhlar, D. G.; He, X. Revised M06-L Functional for Improved Accuracy on Chemical Reaction Barrier Heights, Noncovalent Interactions, and Solid-State Physics. *P. Natl. Acad. Sci. USA.* **2017***, 114*, 8487-8492.

^{1}H and ^{13}C NMR spectra:

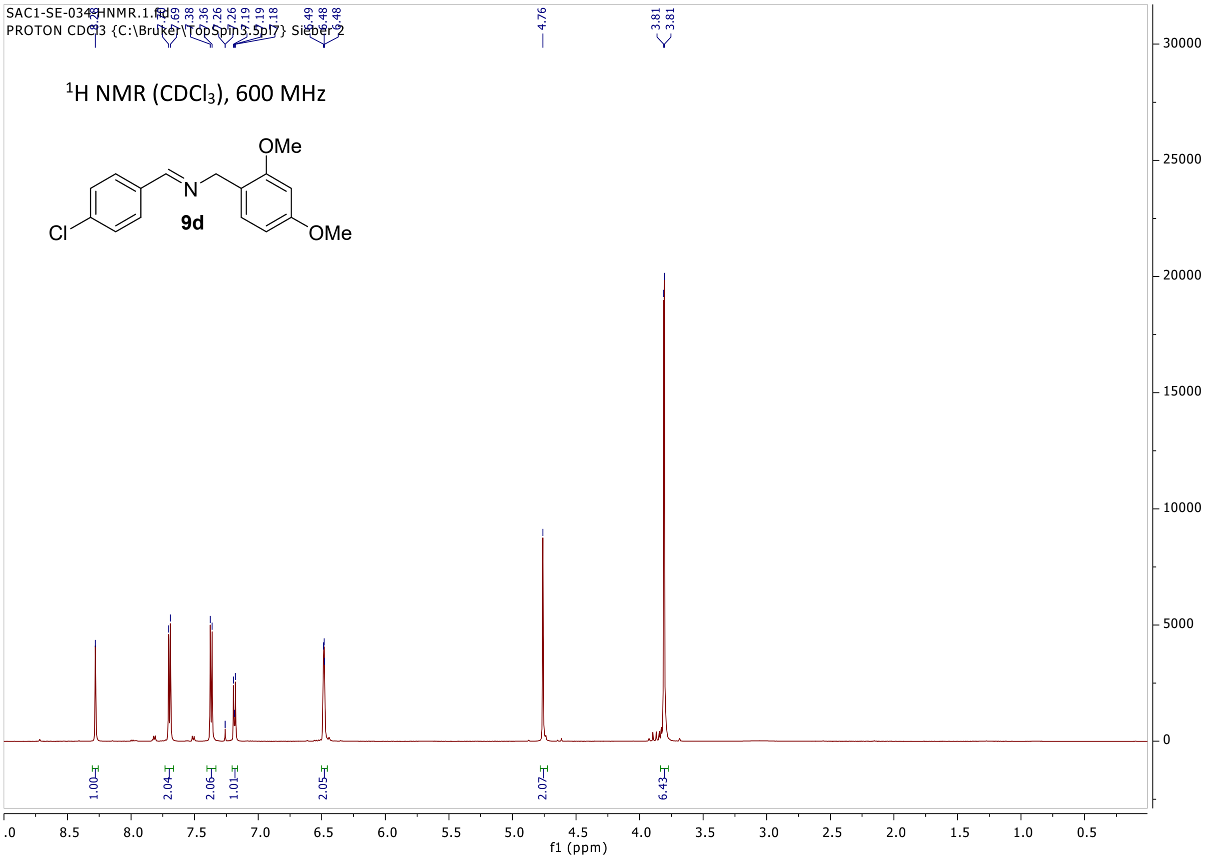

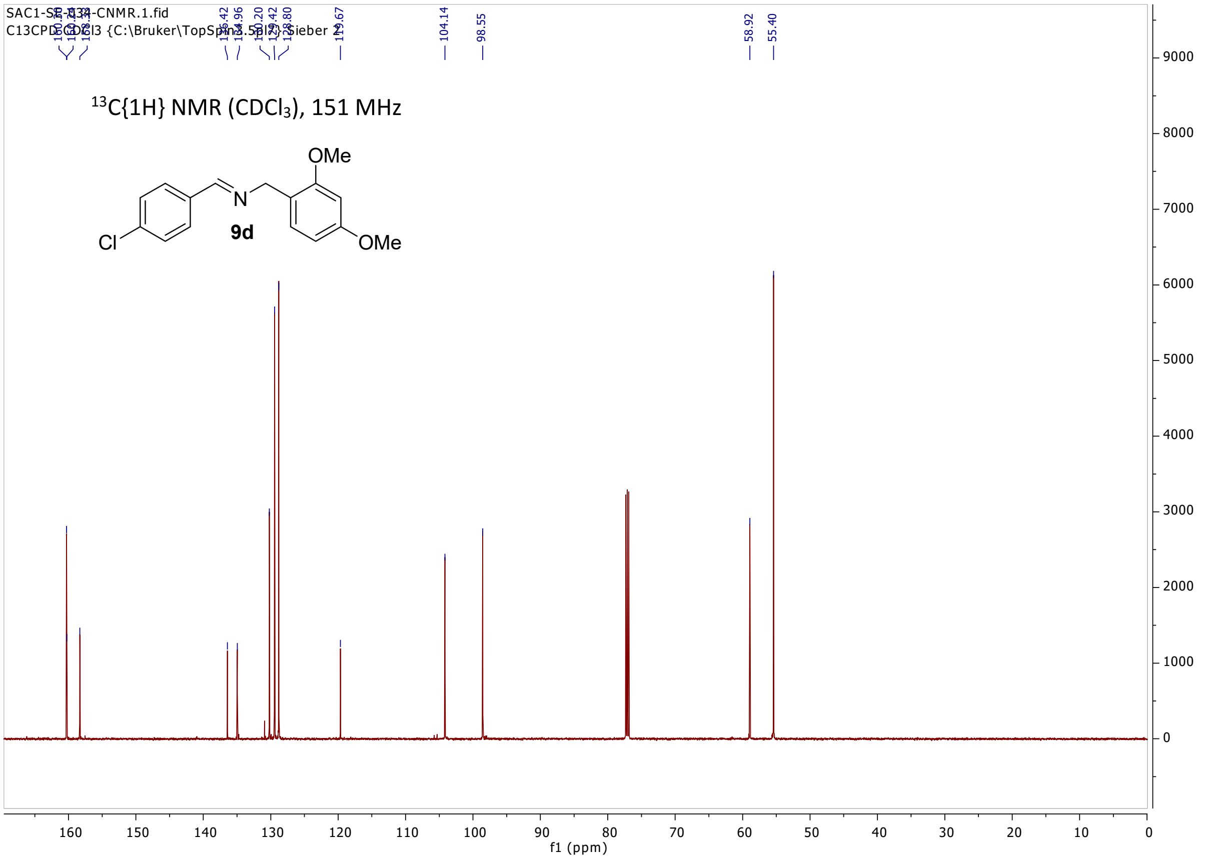

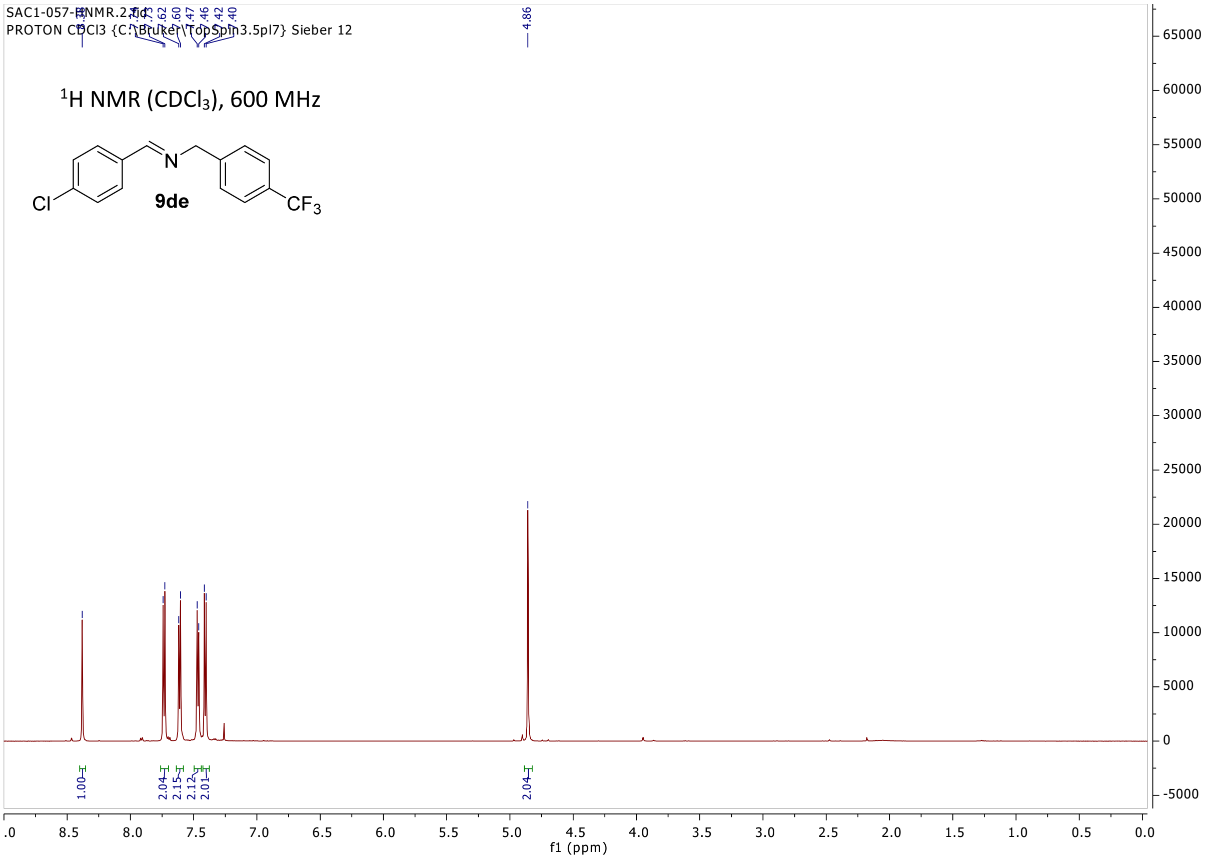
SAC1-057-HNMR.2.fid
PROTON CDCl3 {C:\Bruker\TopSpin3.5pl7} Sieber 12
4.86
^{1}H NMR ($CDCl_3$), 600 MHz
N
Cl
9de
CF_3
1.00
2.04
2.15
2.12
2.01
2.04
f1 (ppm)

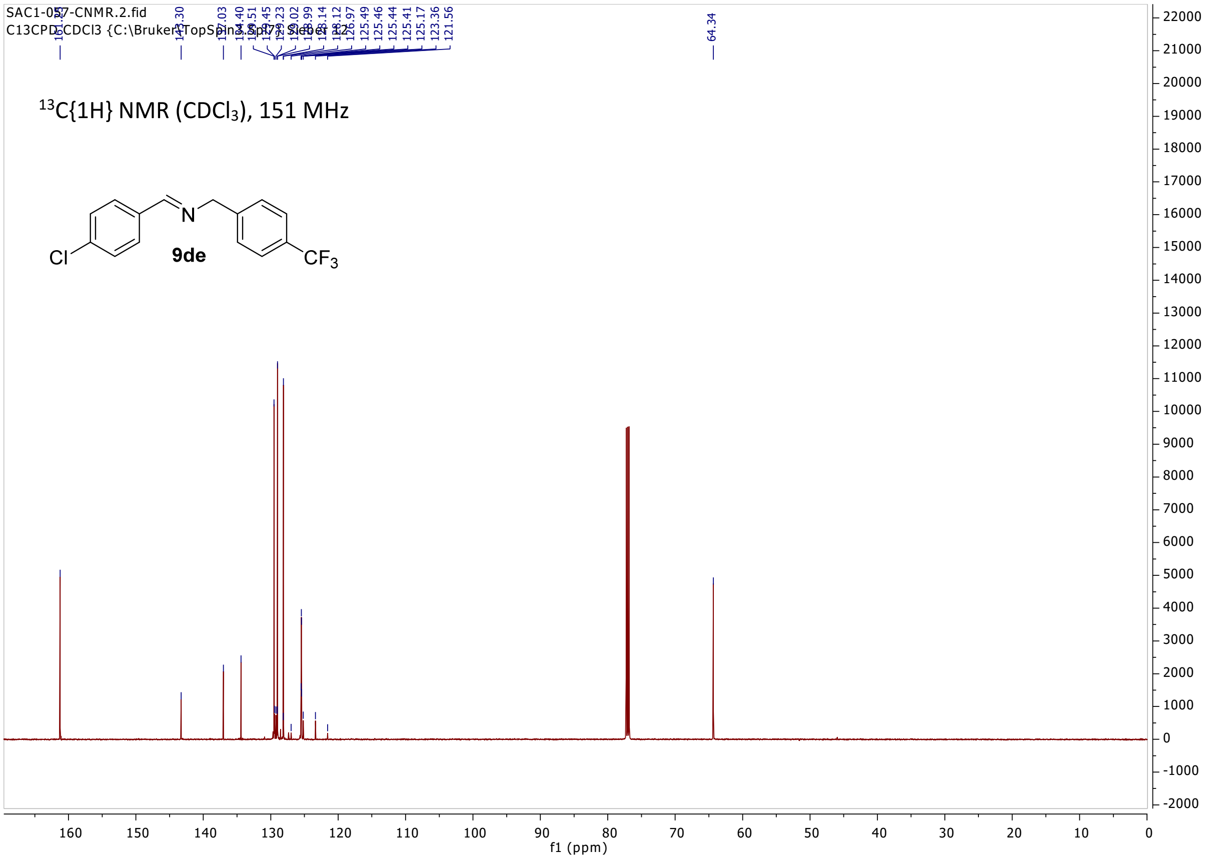
SAC1-057-CNMR.2.fid
C13CPD CDCl3 {C:\Bruker\TopSpin3.5pl7} Sieber 12
143.30
137.03
134.40
129.51
129.45
129.23
129.02
128.99
128.14
128.12
126.97
125.49
125.46
125.44
125.41
125.17
123.36
121.56
64.34
^{13}C{1H} NMR ($CDCl_3$), 151 MHz
N
Cl
9de
CF_3
f1 (ppm)

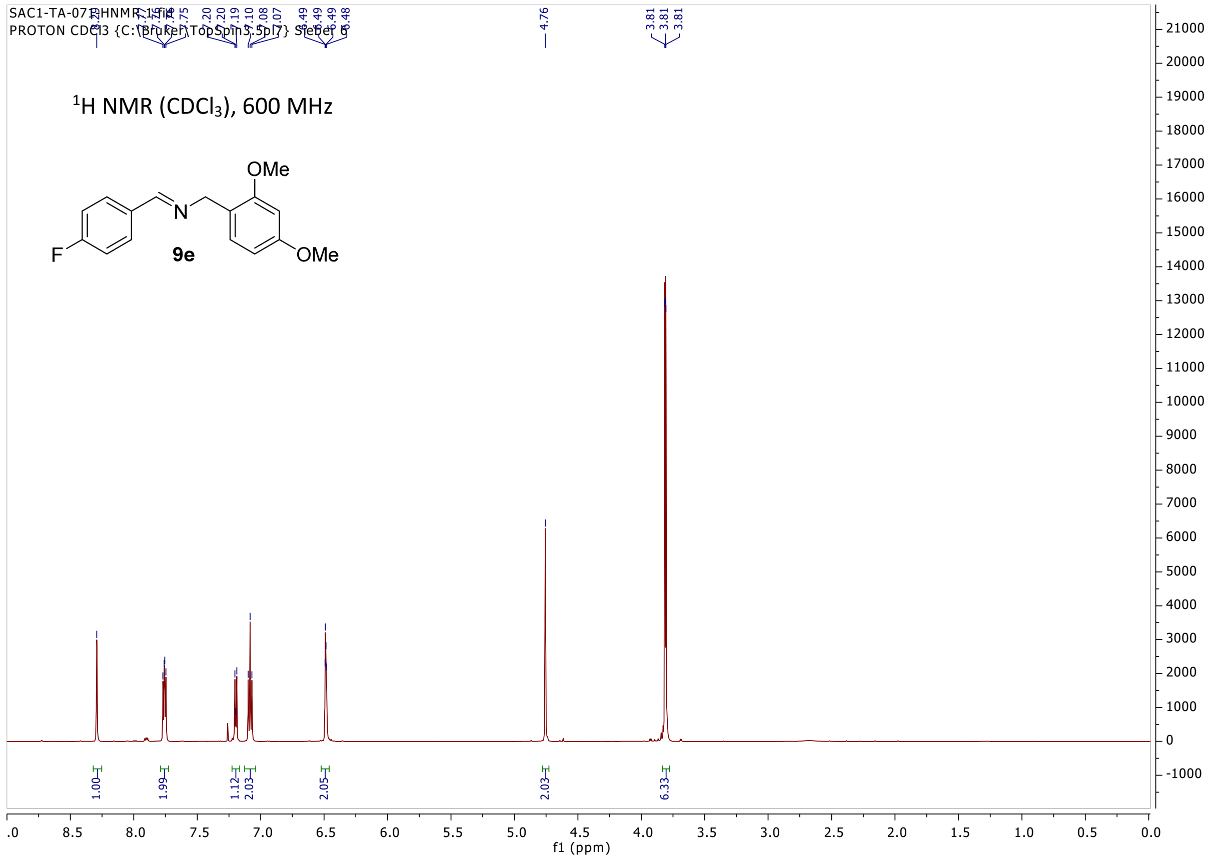
SAC1-TA-071-HNMR.1.fid
PROTON CDCl3 {C:\Bruker\TopSpin3.5pl7} Sieber 6
1H NMR (CDCl3), 600 MHz
OMe
N
F
9e
OMe
4.76
3.81
3.81
3.81
1.00
1.99
1.12
2.03
2.05
2.03
6.33
f1 (ppm)

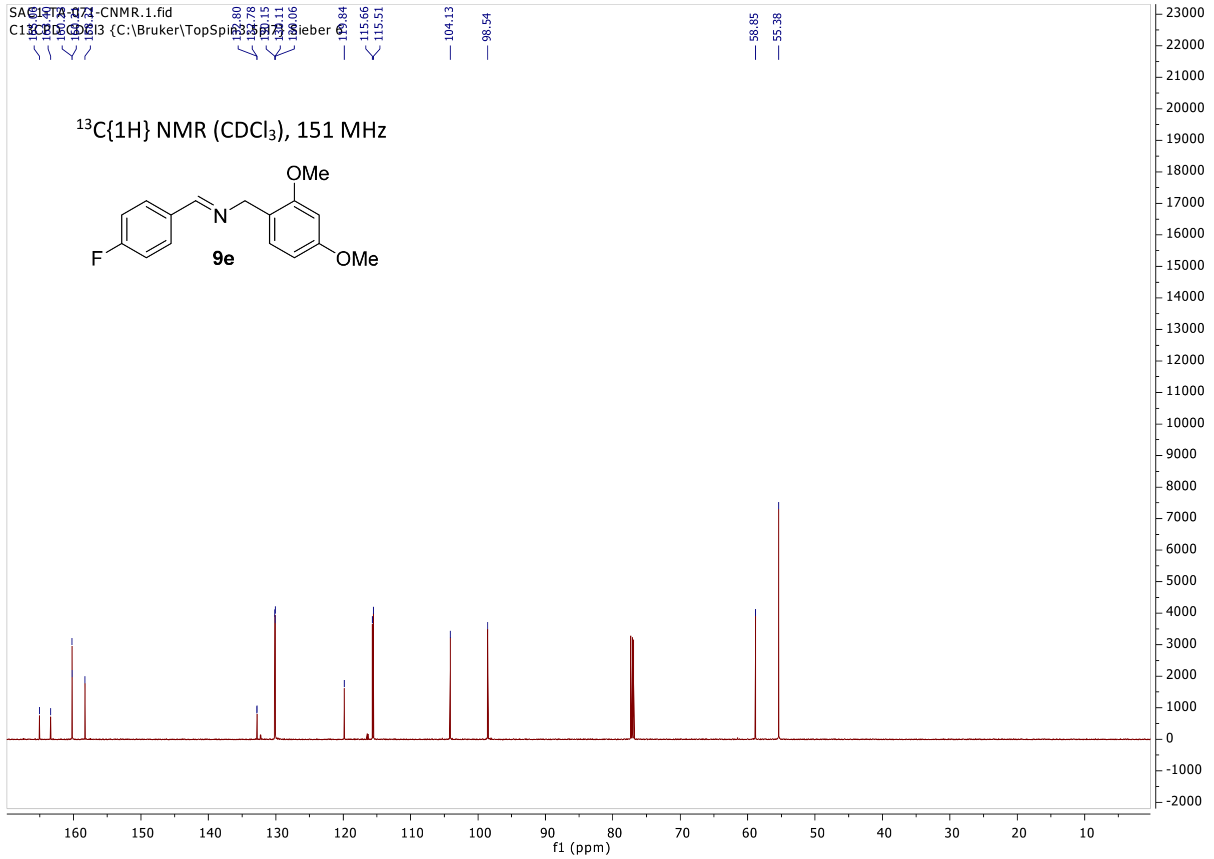
SAC1-TA-071-CNMR.1.fid
C13CPD CDCl3 {C:\Bruker\TopSpin3.5pl7} Sieber 6
13C{1H} NMR (CDCl3), 151 MHz
OMe
N
F
9e
OMe
132.80
132.78
130.15
130.11
130.06
119.84
115.66
115.51
104.13
98.54
58.85
55.38
f1 (ppm)

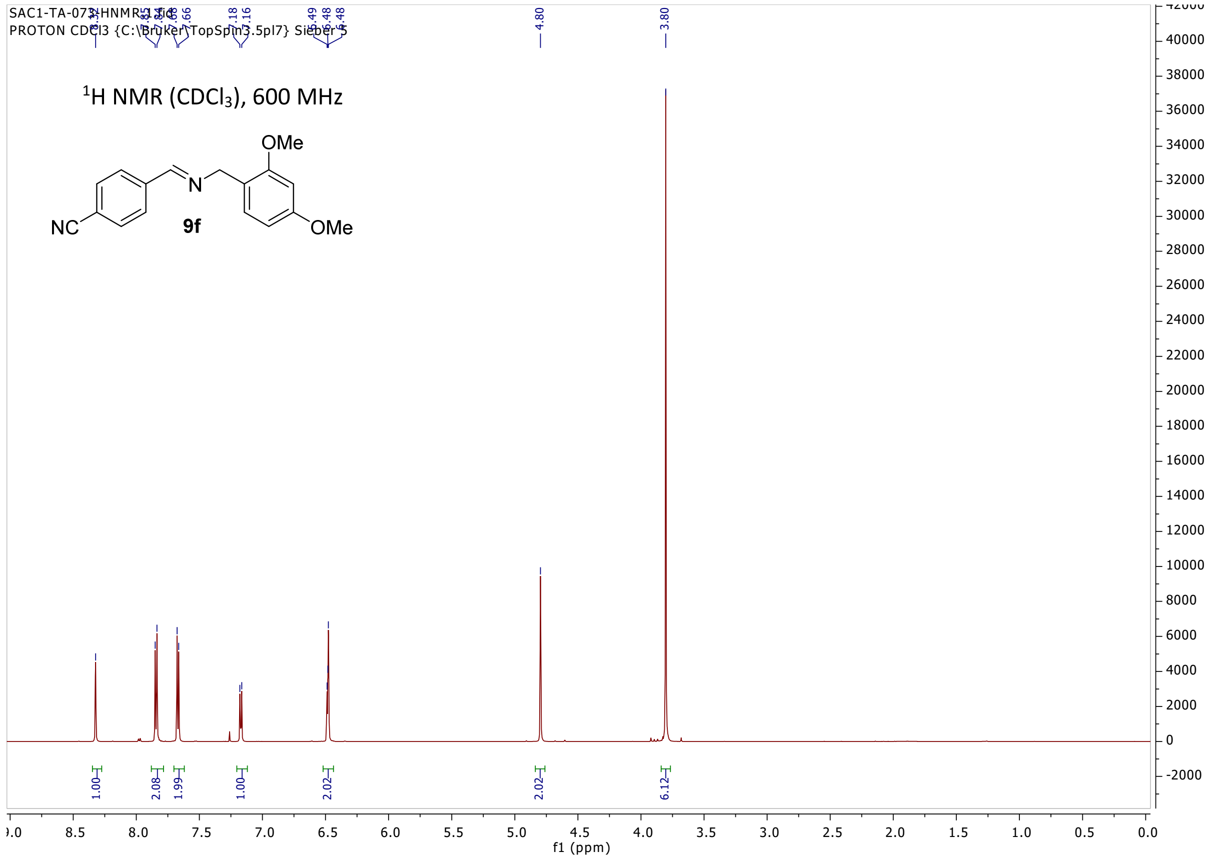
SAC1-TA-073-HNMR.1.fid
PROTON CDCl3 {C:\Bruker\TopSpin3.5pl7} Sieber 5
1H NMR (CDCl3), 600 MHz
OMe
NC
9f
OMe
4.80
3.80
1.00
2.08
1.99
1.00
2.02
2.02
6.12
f1 (ppm)

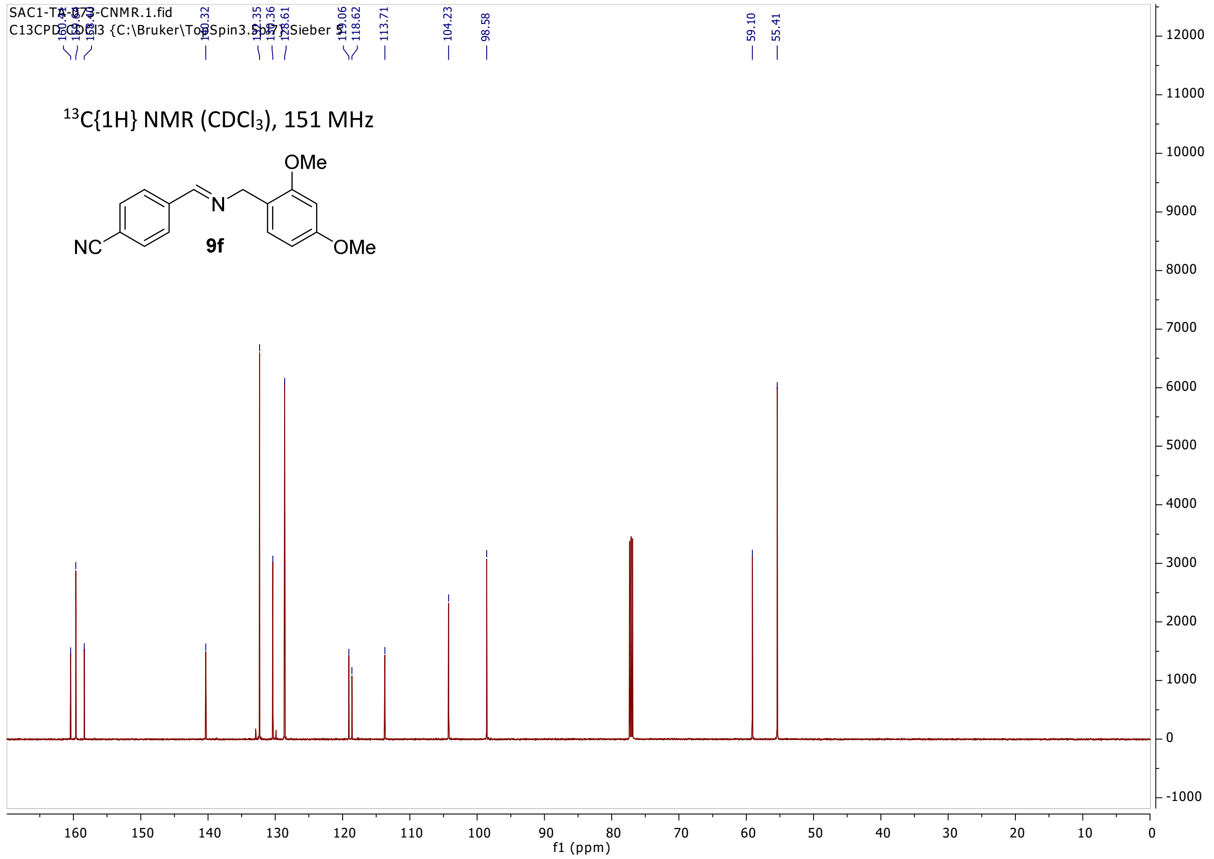
SAC1-TA-073-CNMR.1.fid
C13CPD CDCl3 {C:\Bruker\TopSpin3.5pl7} Sieber 5
13C{1H} NMR (CDCl3), 151 MHz
OMe
NC
9f
OMe
140.32
132.35
130.36
128.61
119.06
118.62
113.71
104.23
98.58
59.10
55.41
f1 (ppm)

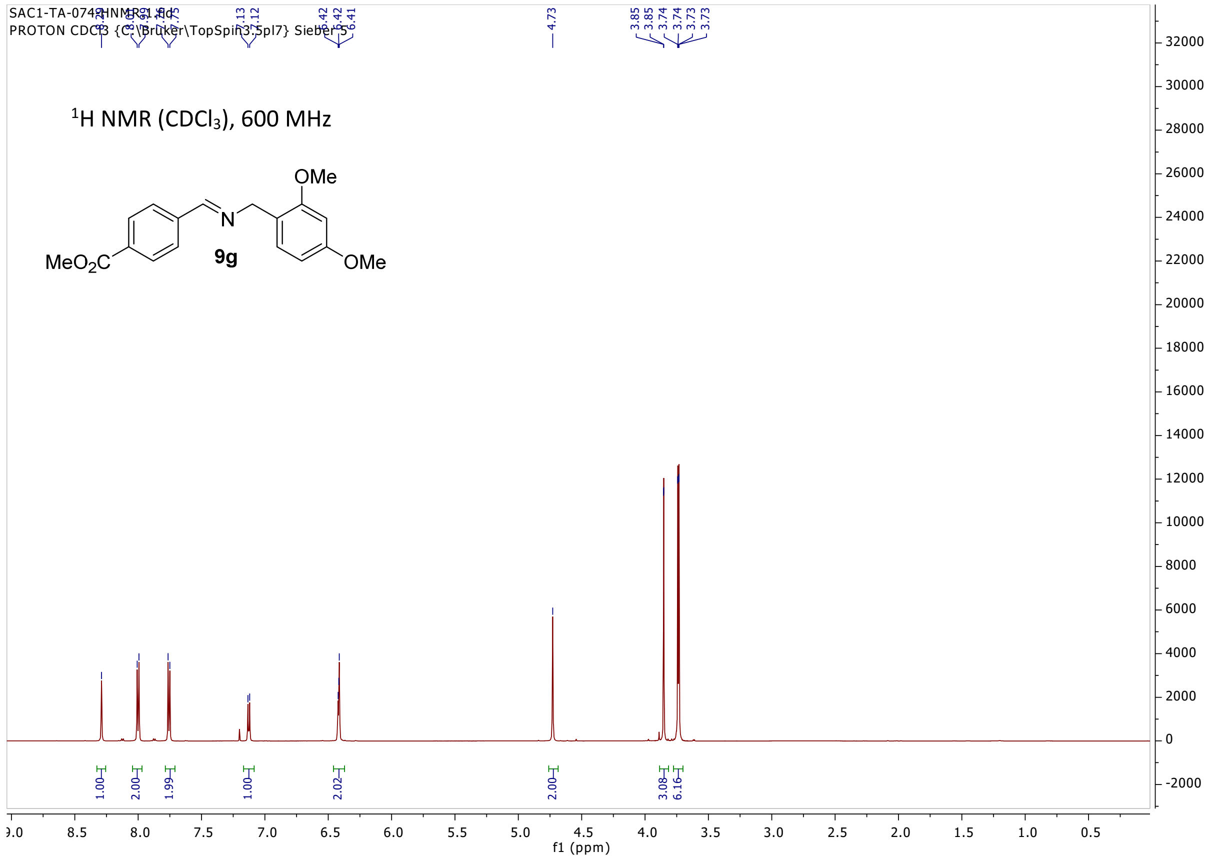

SAC1-TA-074-HNMR.1.fid
PROTON CDCl3 {C:\Bruker\TopSpin3.5pl7} Sieber 5
^{1}H NMR (CDCl$_3$), 600 MHz
OMe
N
MeO$_2$C
9g
OMe
4.73
3.85
3.85
3.74
3.74
3.73
3.73
1.00
2.00
1.99
1.00
2.02
2.00
3.08
6.16
f1 (ppm)

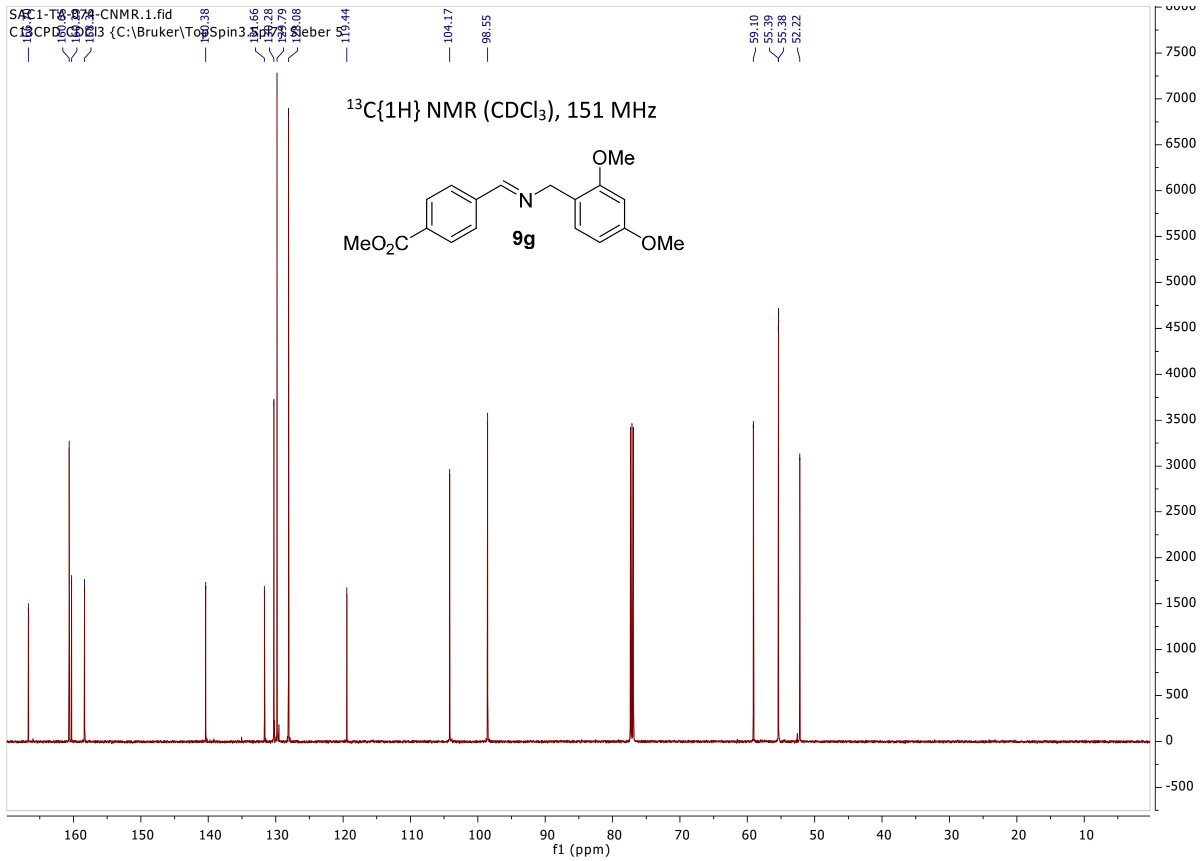

SAC1-TA-074-CNMR.1.fid
C13CPD CDCl3 {C:\Bruker\TopSpin3.5pl7} Sieber 5
140.38
131.66
130.28
129.79
128.08
119.44
104.17
98.55
59.10
55.39
55.38
52.22
^{13}C{1H} NMR (CDCl$_3$), 151 MHz
OMe
N
MeO$_2$C
9g
OMe
f1 (ppm)

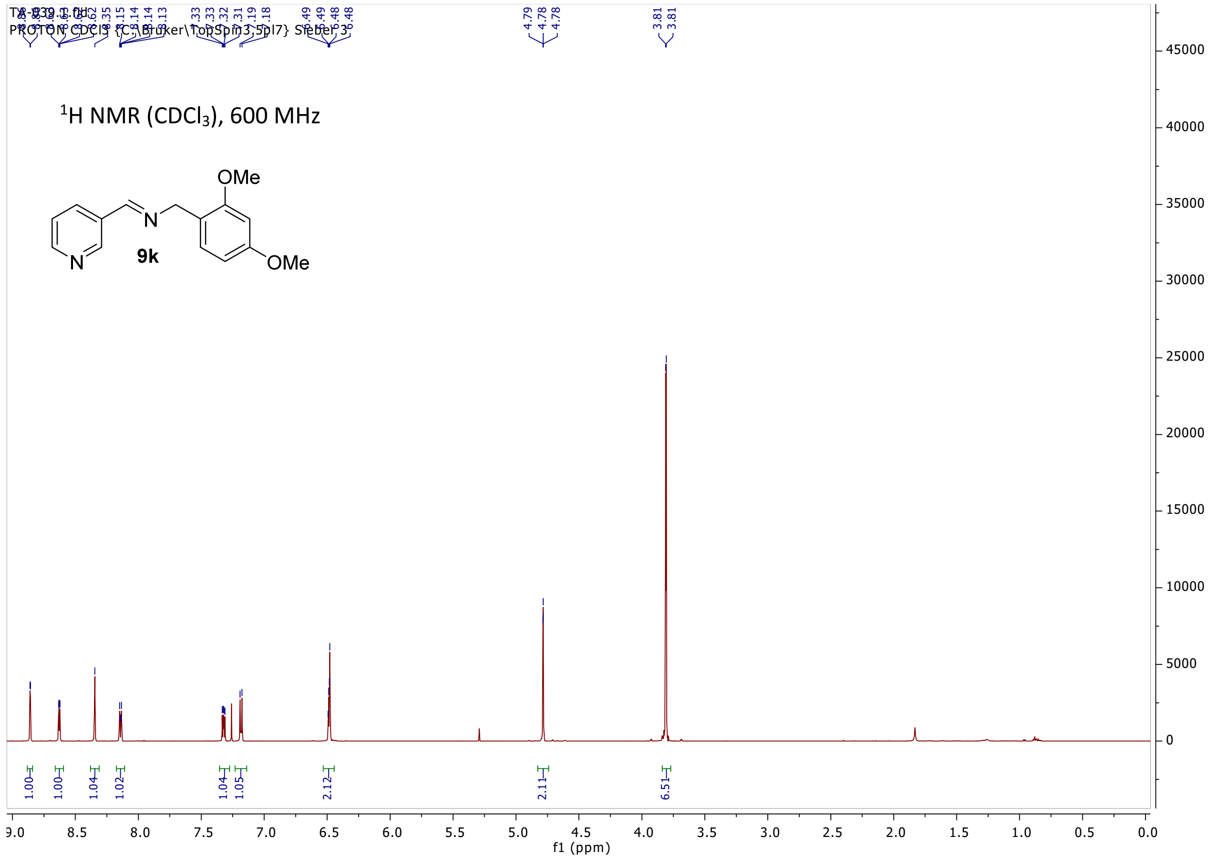
1H NMR (CDCl3), 600 MHz
OMe
N
N
9k
OMe
f1 (ppm)

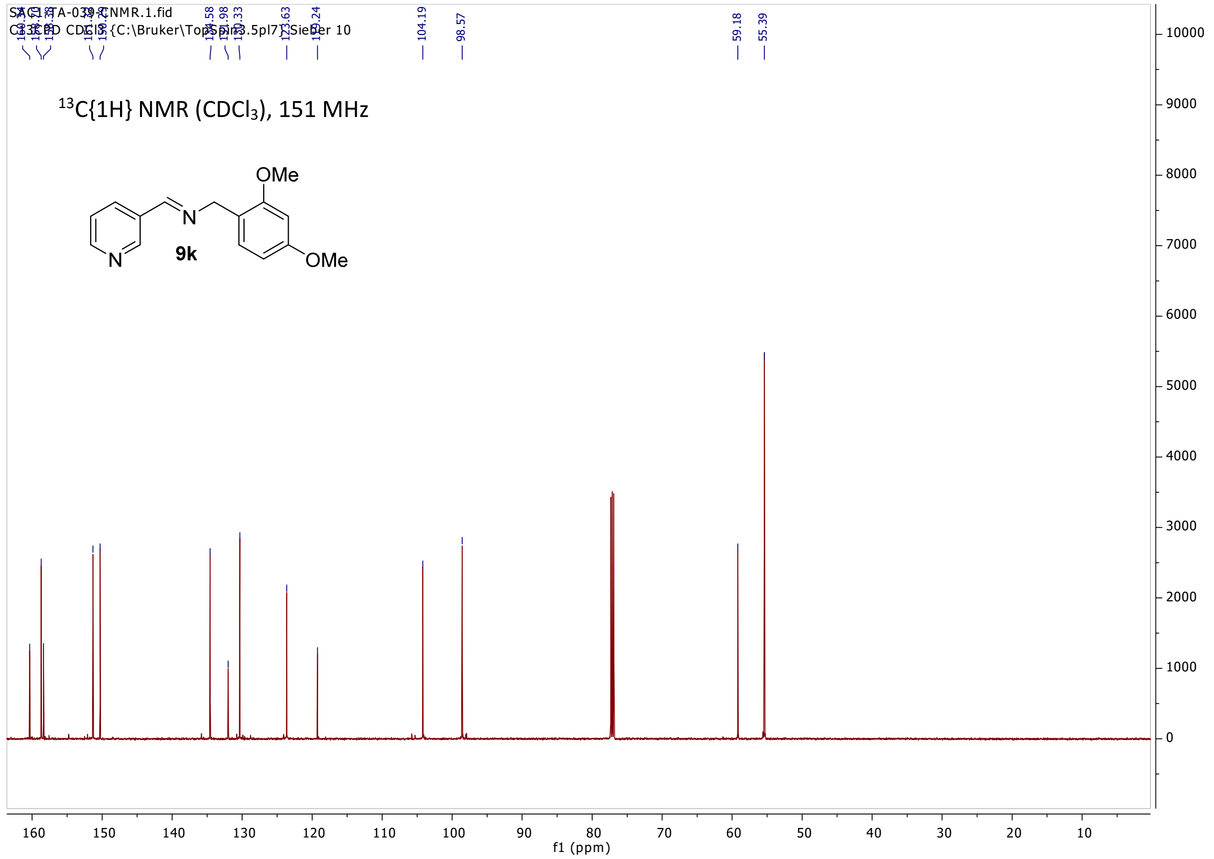
13C{1H} NMR (CDCl3), 151 MHz
OMe
N
N
9k
OMe
f1 (ppm)

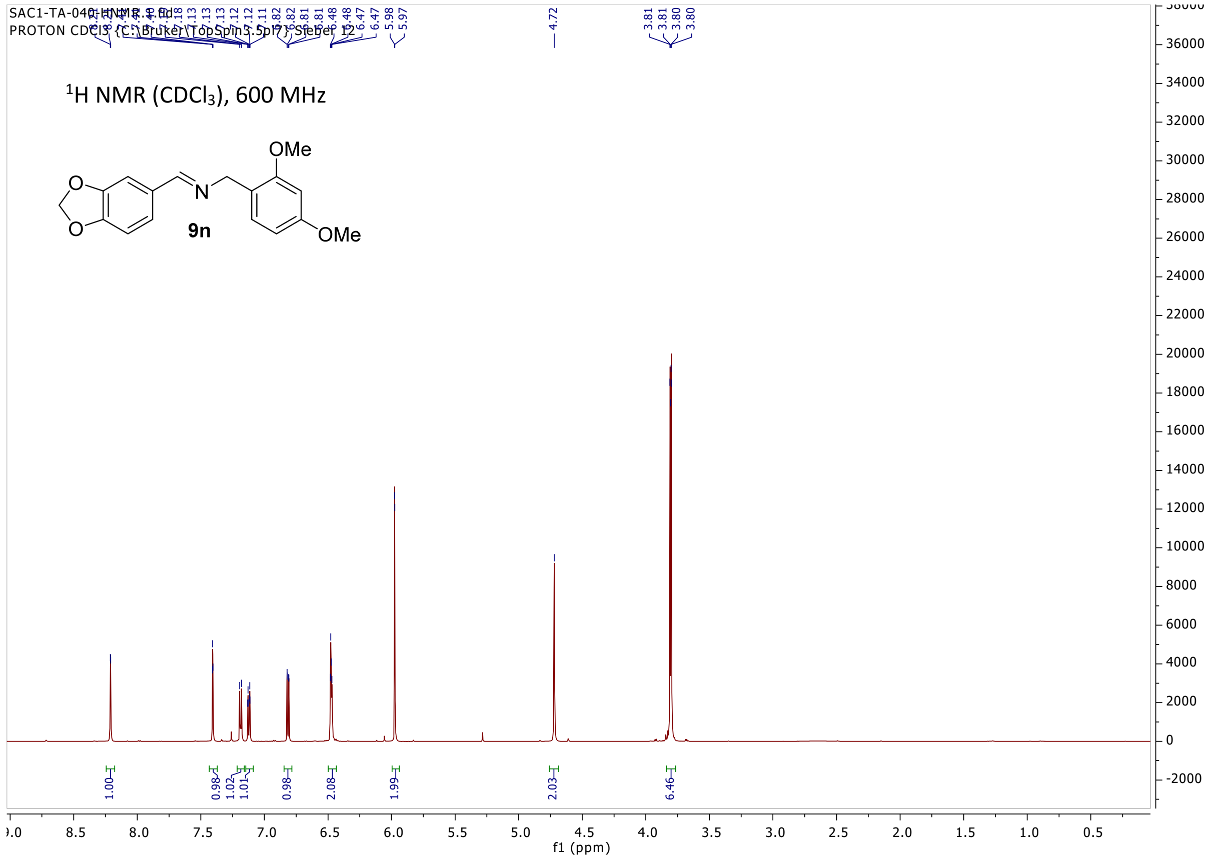
^{1}H NMR ($CDCl_3$), 600 MHz
OMe
N
O
O
9n
OMe
f1 (ppm)

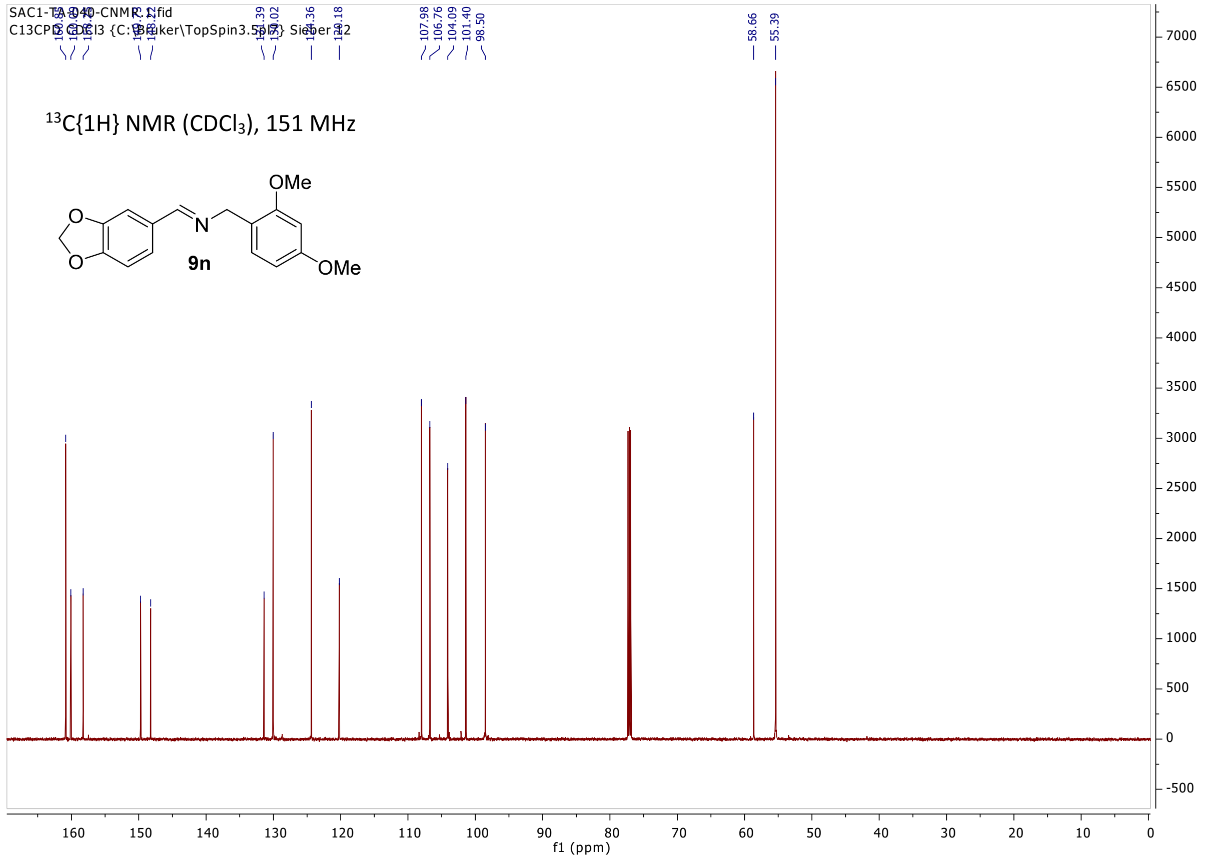
^{13}C{1H} NMR ($CDCl_3$), 151 MHz
OMe
N
O
O
9n
OMe
f1 (ppm)

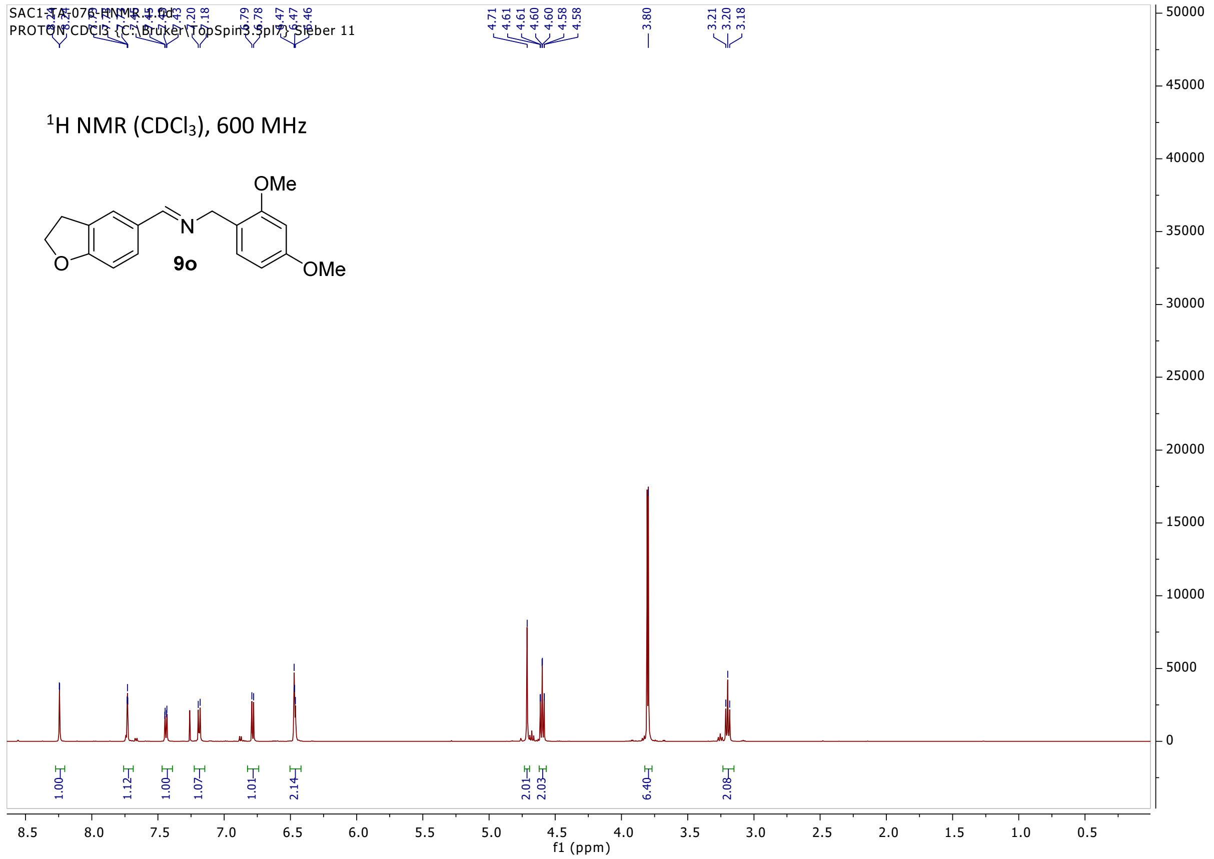

SAC1-TA-076-HNMR.1.fid
PROTON CDCl3 {C:\Bruker\TopSpin3.5pl7} Sieber 11
4.71
4.61
4.61
4.60
4.60
4.58
4.58
3.80
3.21
3.20
3.18
1H NMR (CDCl3), 600 MHz
OMe
N
O
9o
OMe
1.00
1.12
1.00
1.07
1.01
2.14
2.01
2.03
6.40
2.08
f1 (ppm)

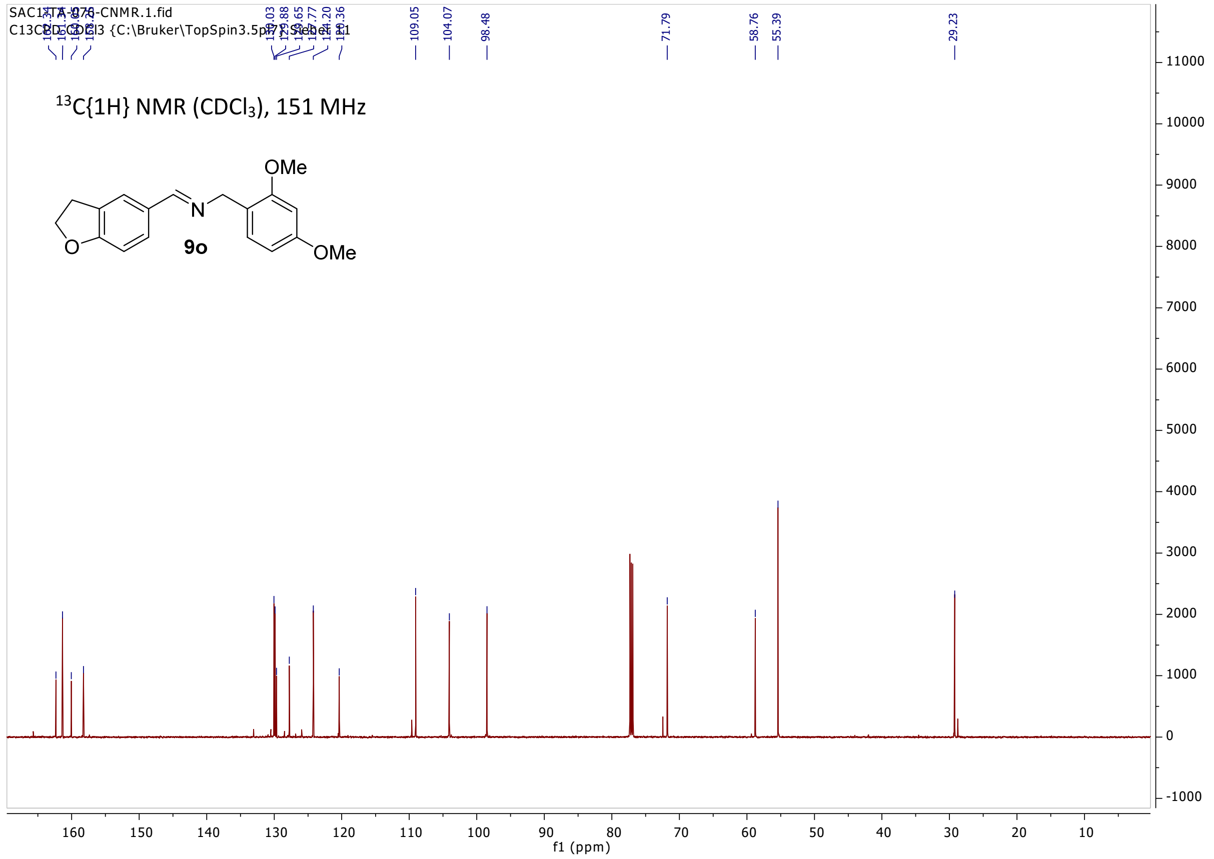

SAC1-TA-076-CNMR.1.fid
C13CPD CDCl3 {C:\Bruker\TopSpin3.5pl7} Sieber 11
109.05
104.07
98.48
71.79
58.76
55.39
29.23
13C{1H} NMR (CDCl3), 151 MHz
OMe
N
O
9o
OMe
f1 (ppm)

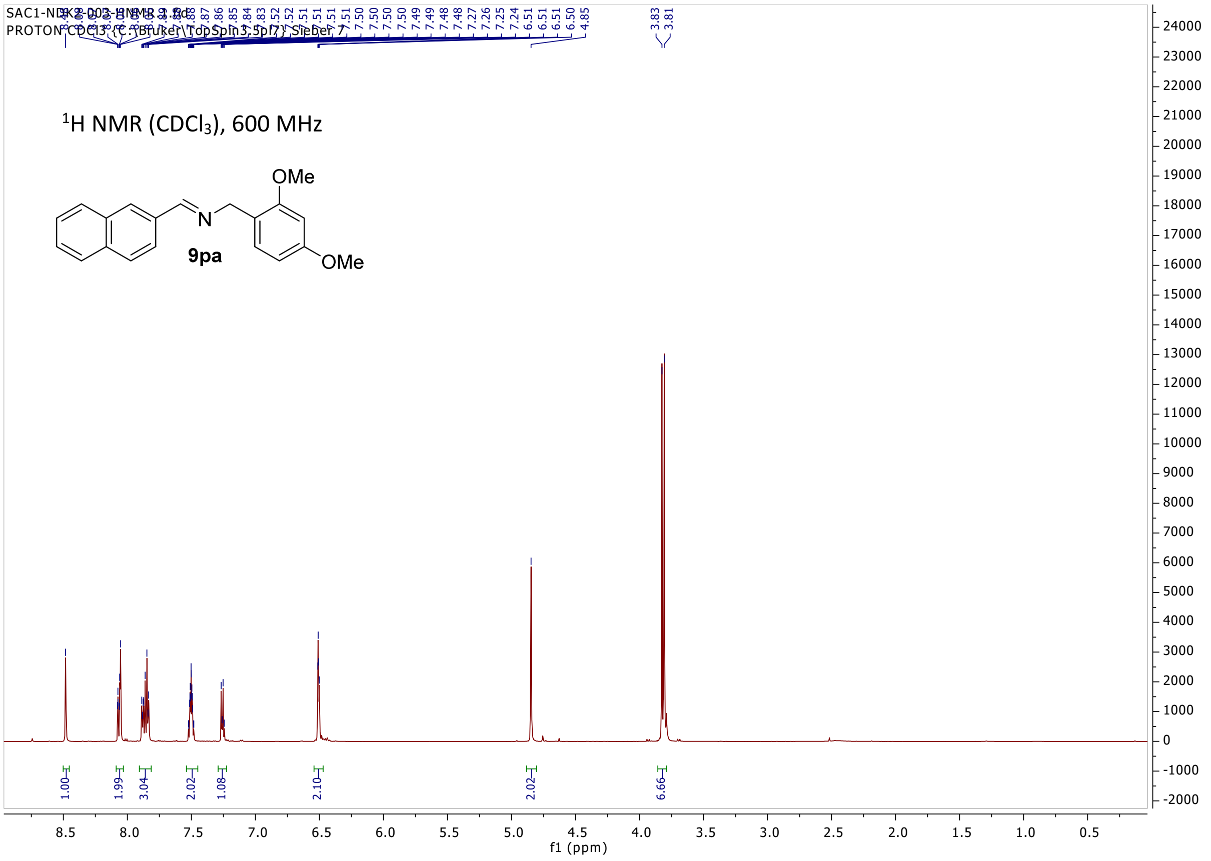
SAC1-NDK2-003-HNMR.1.fid
PROTON CDCl3 {C:\Bruker\TopSpin3.5pl7} Sieber 7
^{1}H NMR (CDCl$_3$), 600 MHz
OMe
N
9pa
OMe
3.83
3.81
1.00
1.99
3.04
2.02
1.08
2.10
2.02
6.66
f1 (ppm)

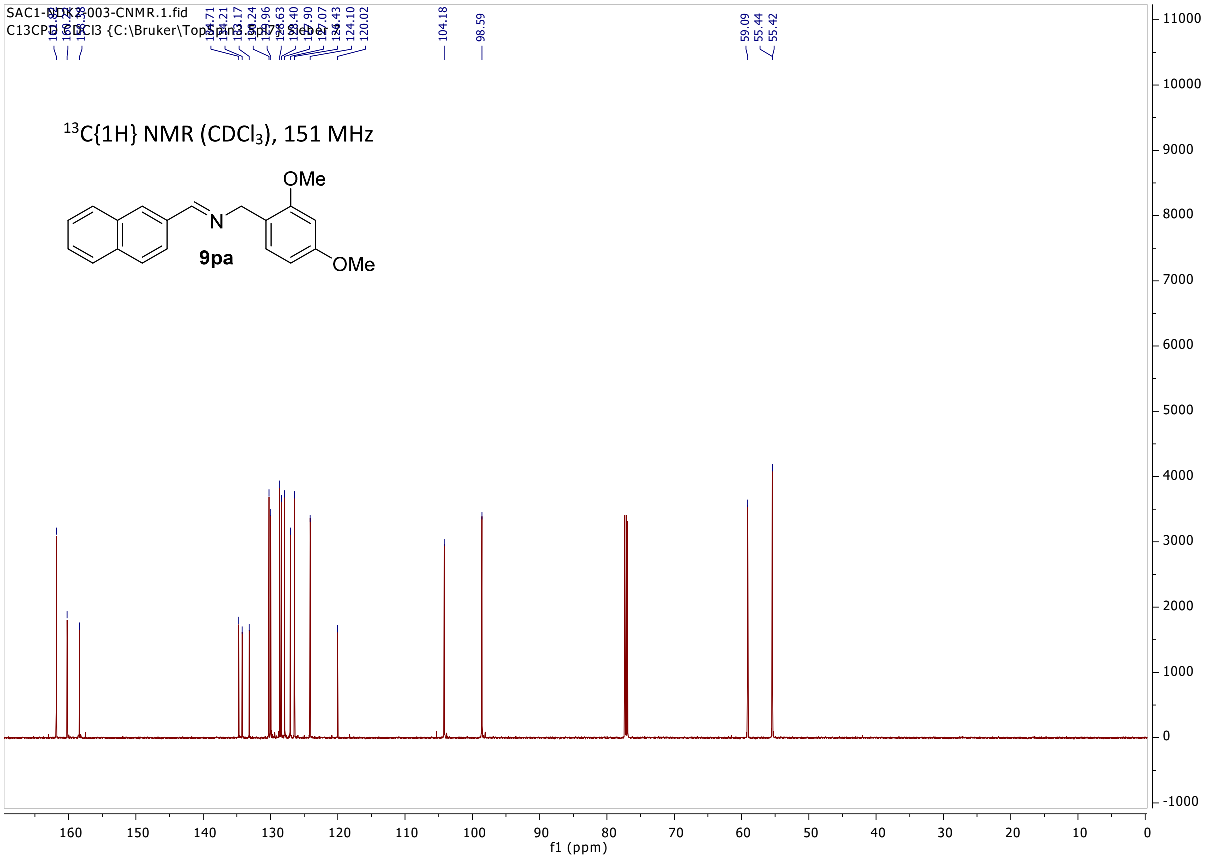
SAC1-NDK2-003-CNMR.1.fid
C13CPD CDCl3 {C:\Bruker\TopSpin3.5pl7} Sieber
^{13}C{1H} NMR (CDCl$_3$), 151 MHz
OMe
N
9pa
OMe
134.71
134.21
133.17
130.24
129.96
128.63
128.40
127.90
127.07
126.43
124.10
120.02
104.18
98.59
59.09
55.44
55.42
f1 (ppm)

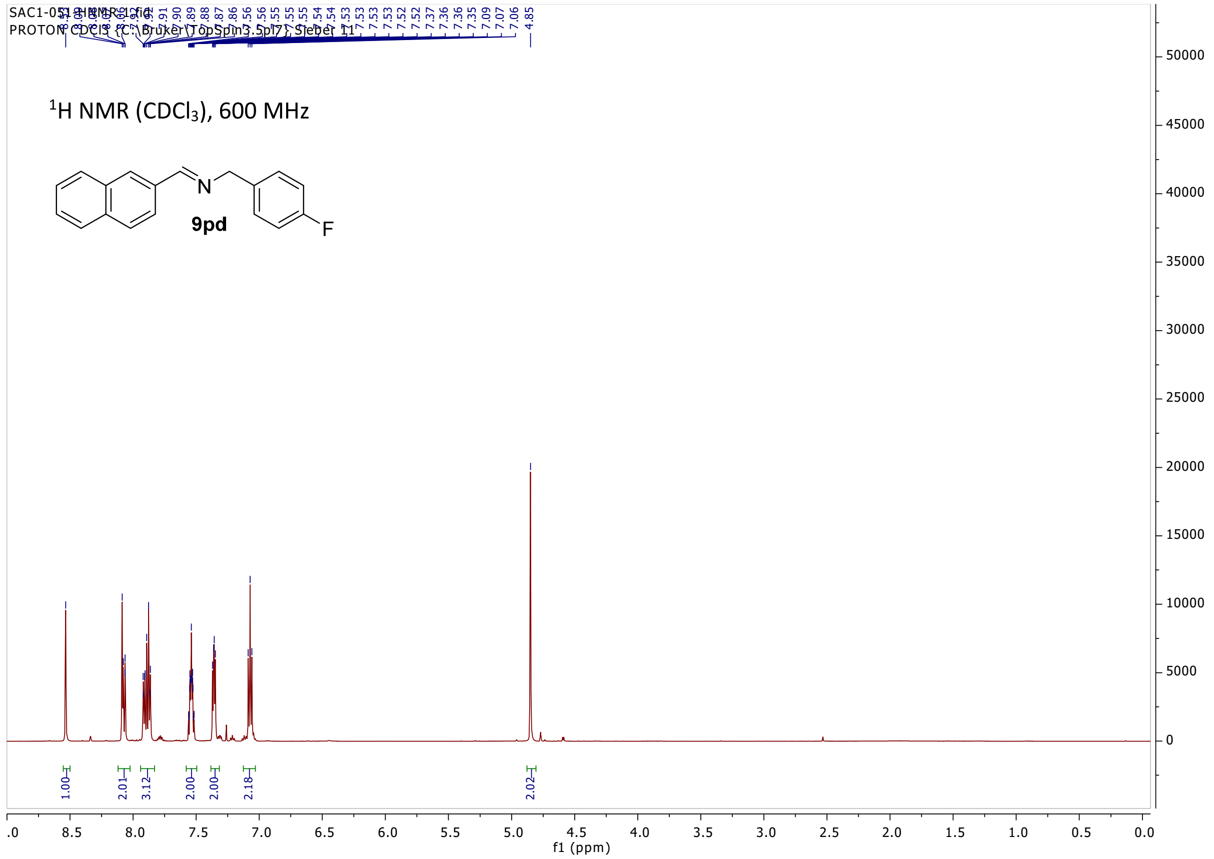

SAC1-051.1HNMR.1.fid
PROTON CDCl3 {C:\Bruker\TopSpin3.5pl7} Sieber 11
^{1}H NMR (CDCl$_3$), 600 MHz
9pd
f1 (ppm)

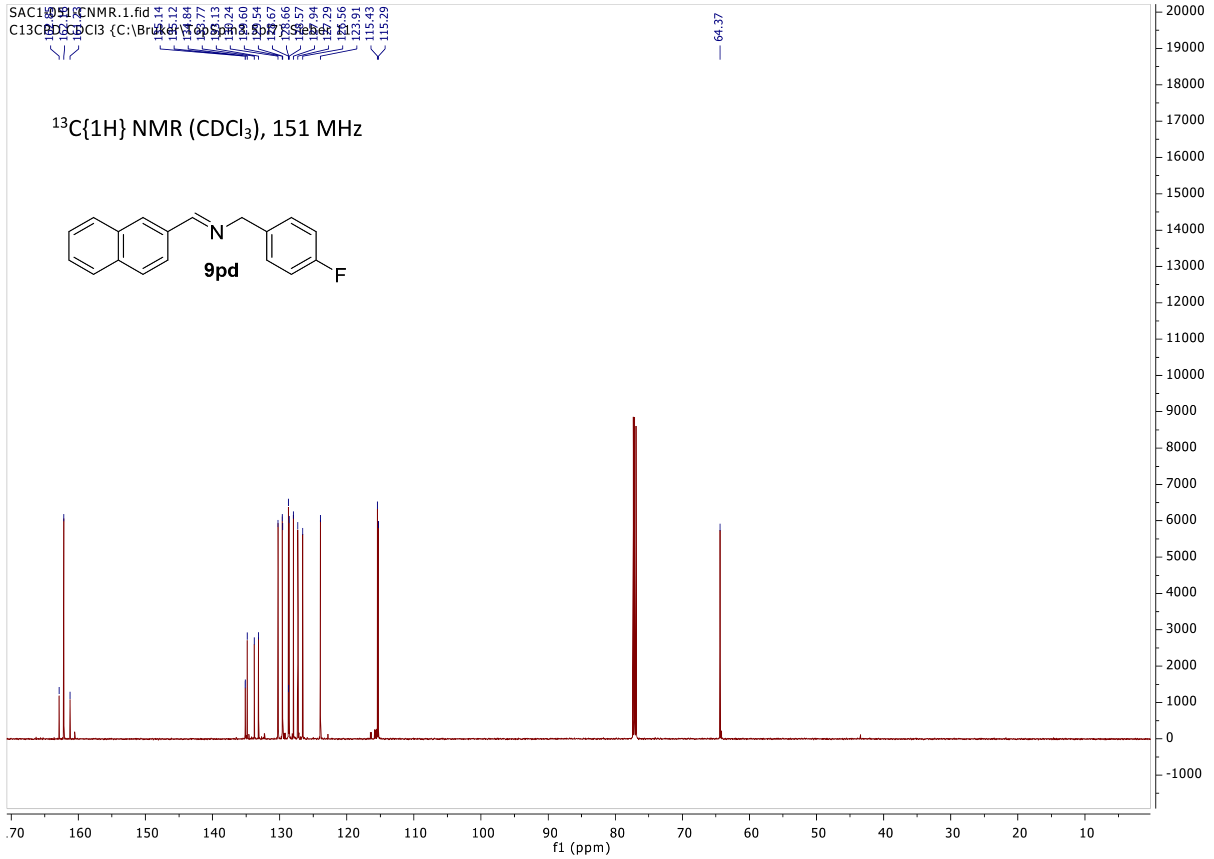

SAC1-051.CNMR.1.fid
C13CPD CDCl3 {C:\Bruker\TopSpin3.5pl7} Sieber 11
^{13}C{1H} NMR (CDCl$_3$), 151 MHz
9pd
f1 (ppm)

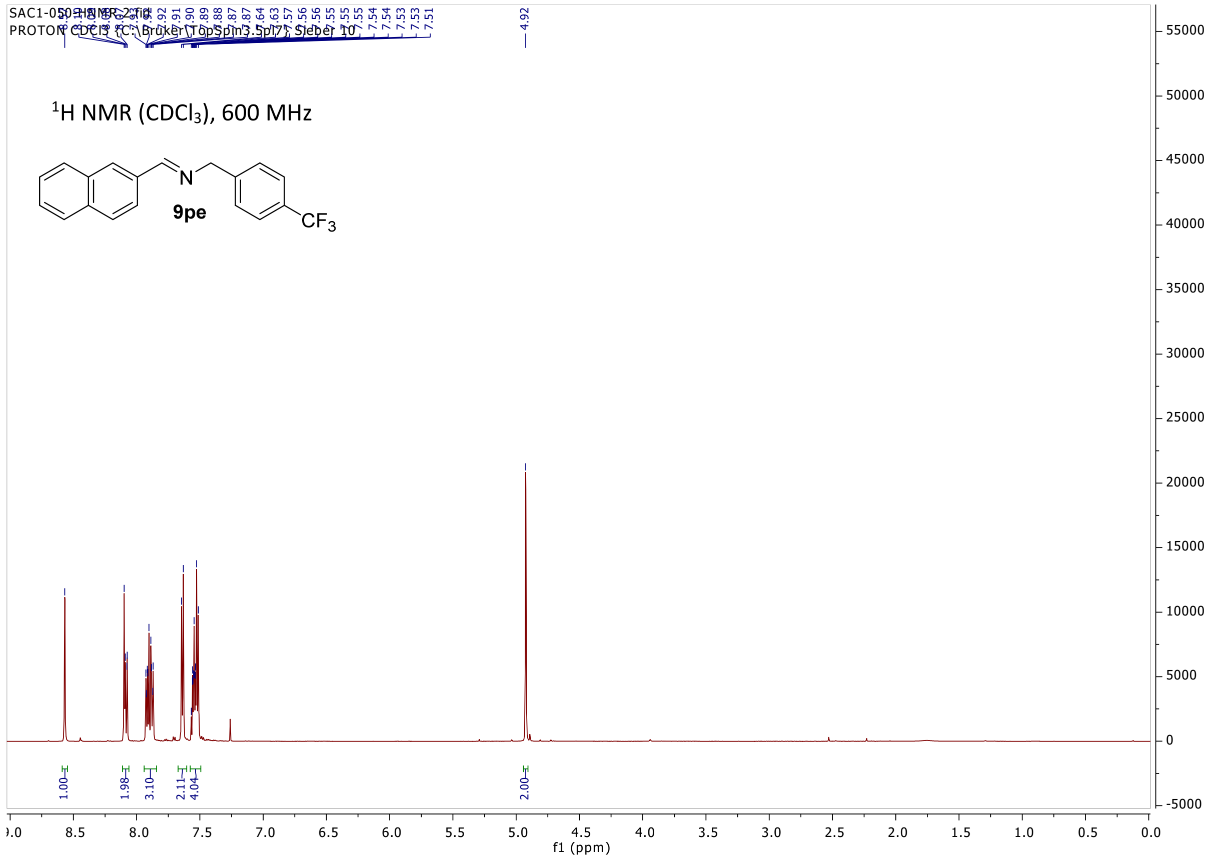

PROTON CDCl3 {C:\Bruker\TopSpin3.5pl7} Sieber 10
^{1}H NMR ($CDCl_3$), 600 MHz
9pe
CF_3
4.92
1.00
1.98
3.10
2.11
4.04
2.00
f1 (ppm)

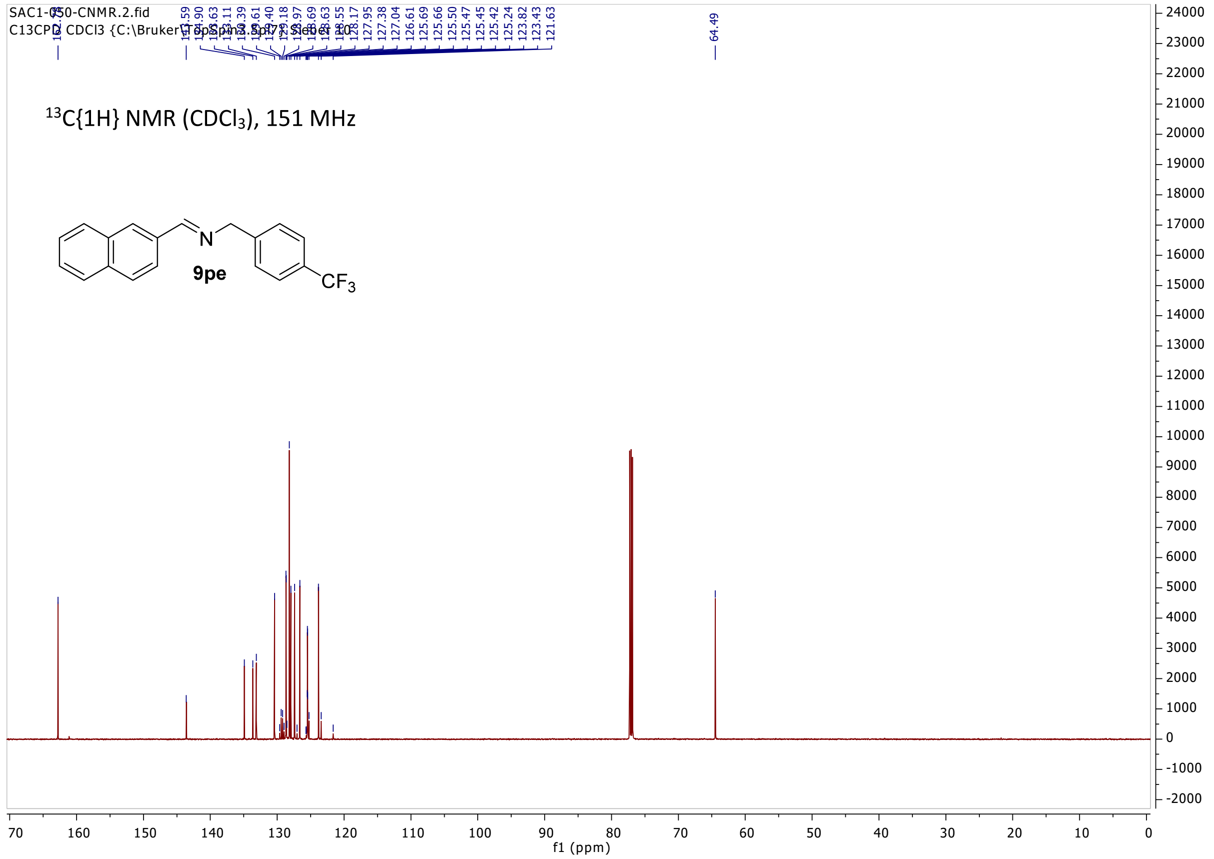

SAC1-050-CNMR.2.fid
C13CPD CDCl3 {C:\Bruker\TopSpin3.5pl7} Sieber 10
^{13}C{1H} NMR ($CDCl_3$), 151 MHz
9pe
CF_3
64.49
f1 (ppm)

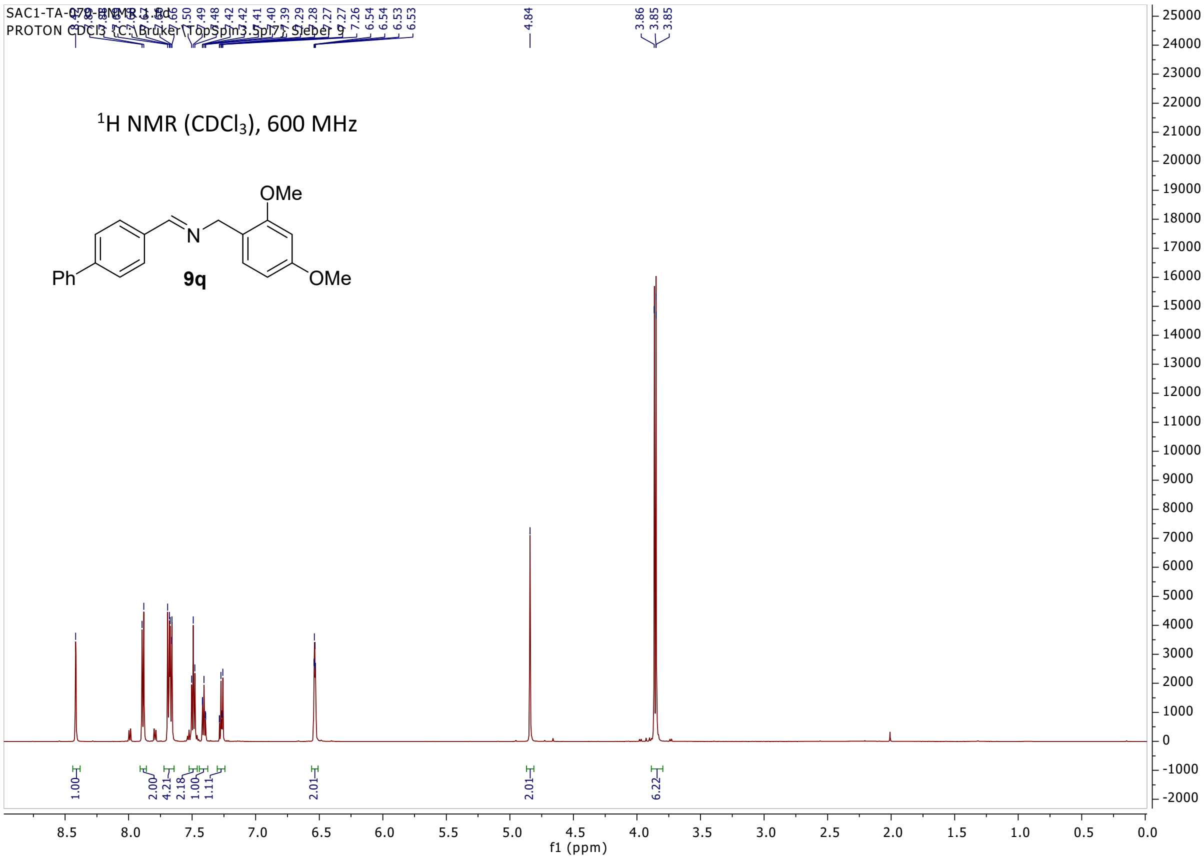

PROTON CDCl3 {C:\Bruker\TopSpin3.5pl7} Sieber 9
1H NMR (CDCl3), 600 MHz
OMe
N
Ph
9q
OMe
4.84
3.86
3.85
3.85
1.00
2.00
4.21
2.18
1.00
1.11
2.01
2.01
6.22
f1 (ppm)

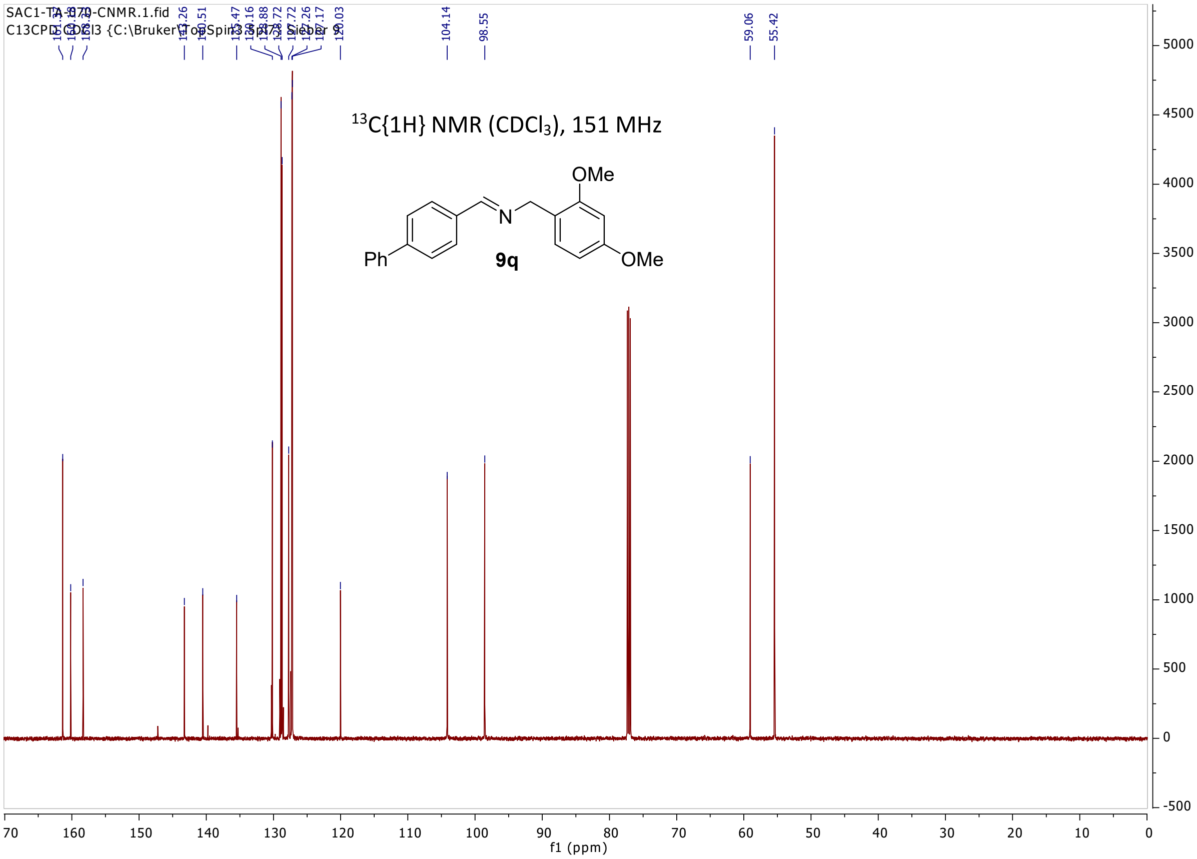

SAC1-TA-070-CNMR.1.fid
104.14
98.55
59.06
55.42
13C{1H} NMR (CDCl3), 151 MHz
OMe
N
Ph
9q
OMe
f1 (ppm)

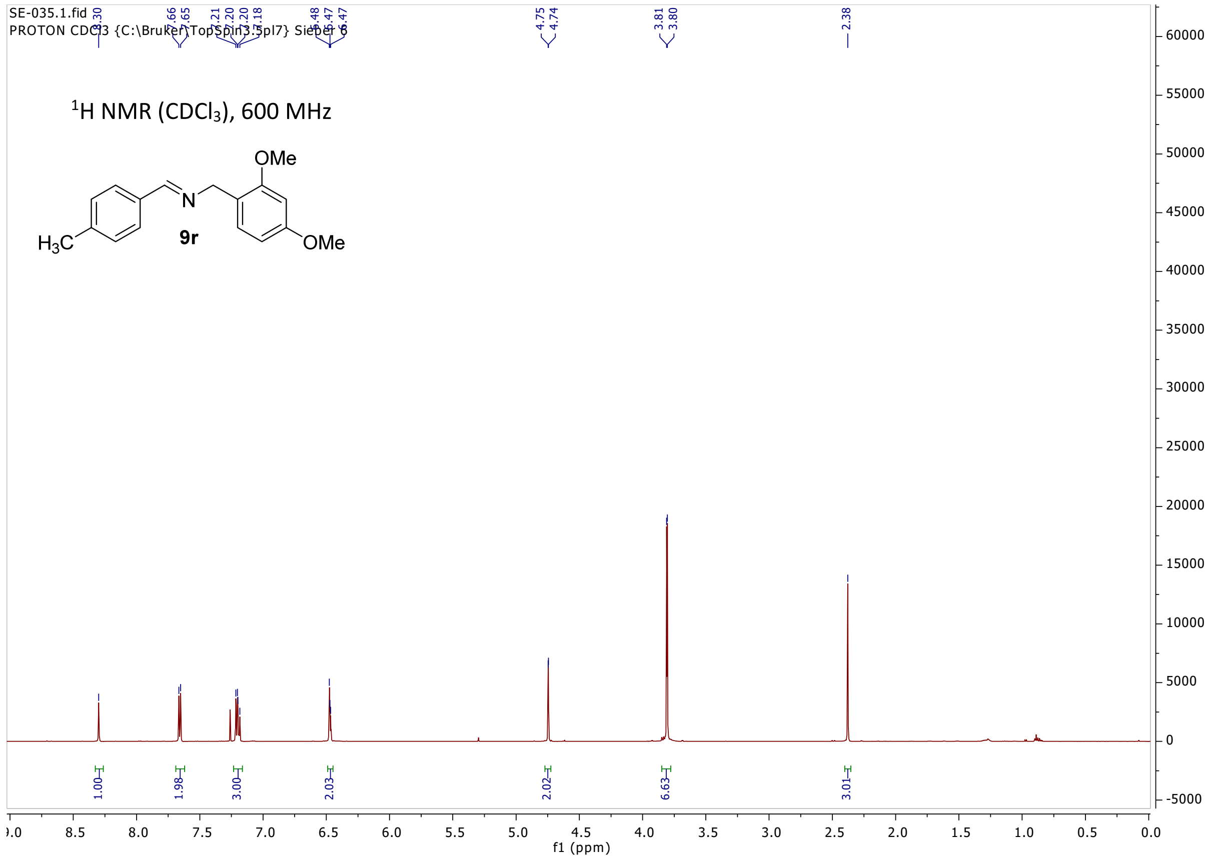
SE-035.1.fid
PROTON CDCl3 {C:\Bruker\TopSpin3.5pl7} Sieber 6
1H NMR (CDCl3), 600 MHz
OMe
N
9r
H3C
OMe
f1 (ppm)

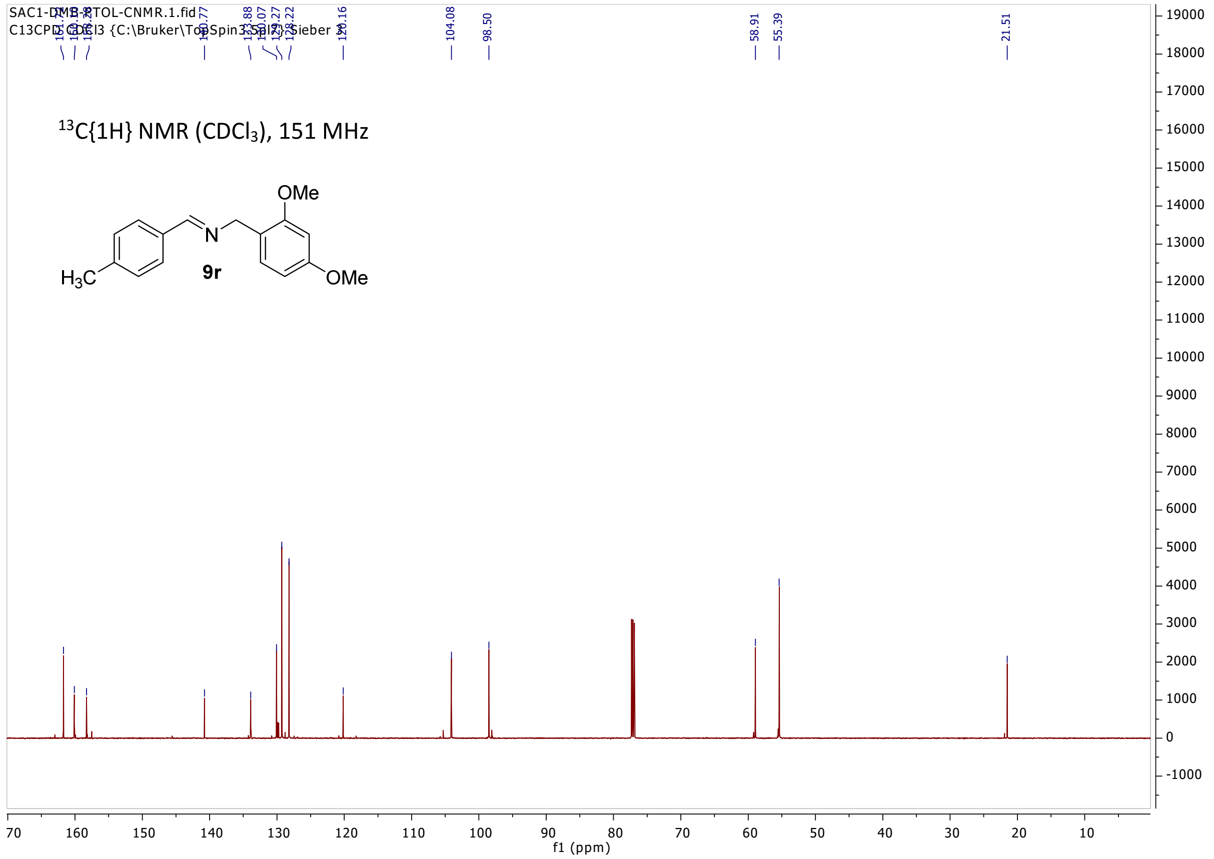
C13CPD CDCl3 {C:\Bruker\TopSpin3.5pl7} Sieber 3
13C{1H} NMR (CDCl3), 151 MHz
OMe
N
9r
H3C
OMe
104.08
98.50
58.91
55.39
21.51
f1 (ppm)

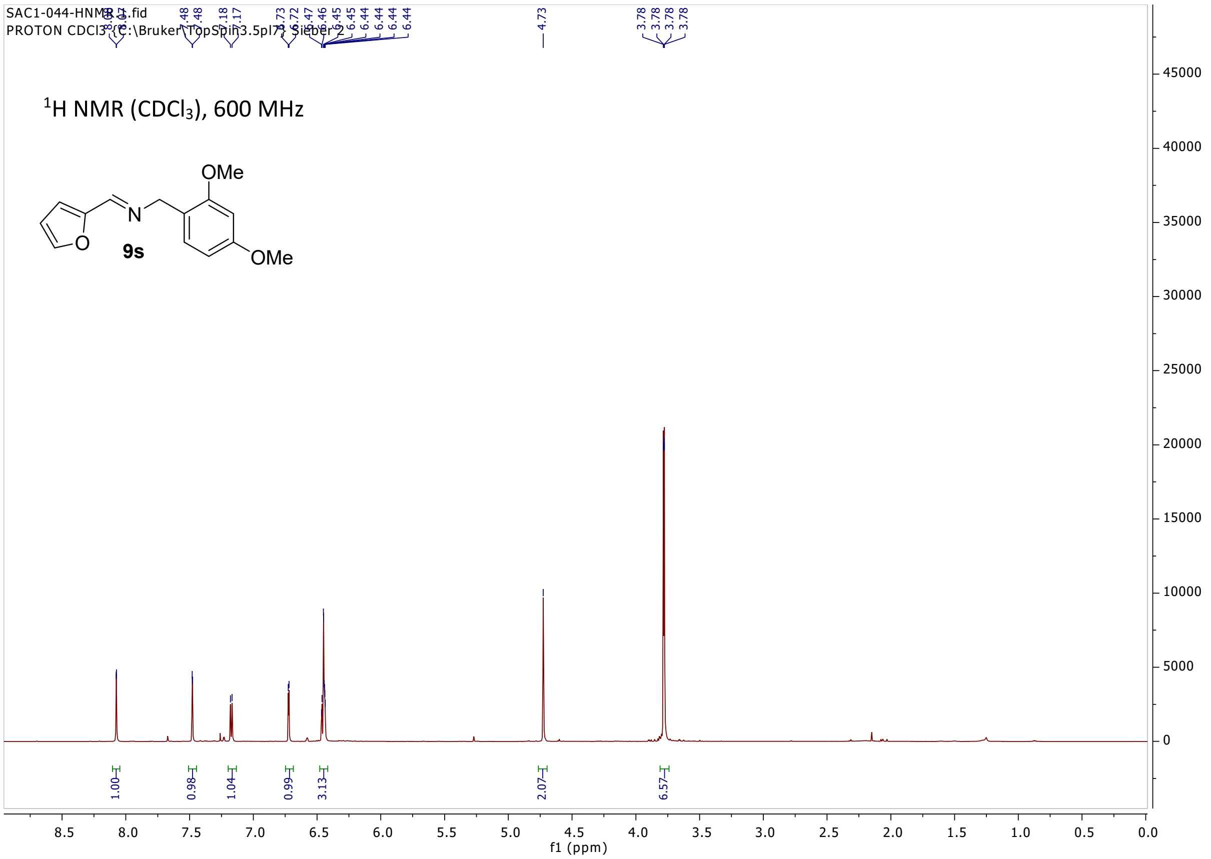

SAC1-044-HNMR.1.fid
PROTON CDCl3 {C:\Bruker\TopSpin3.5pl7} Sieber 2
1H NMR (CDCl3), 600 MHz
9s
OMe
OMe
N
O
f1 (ppm)

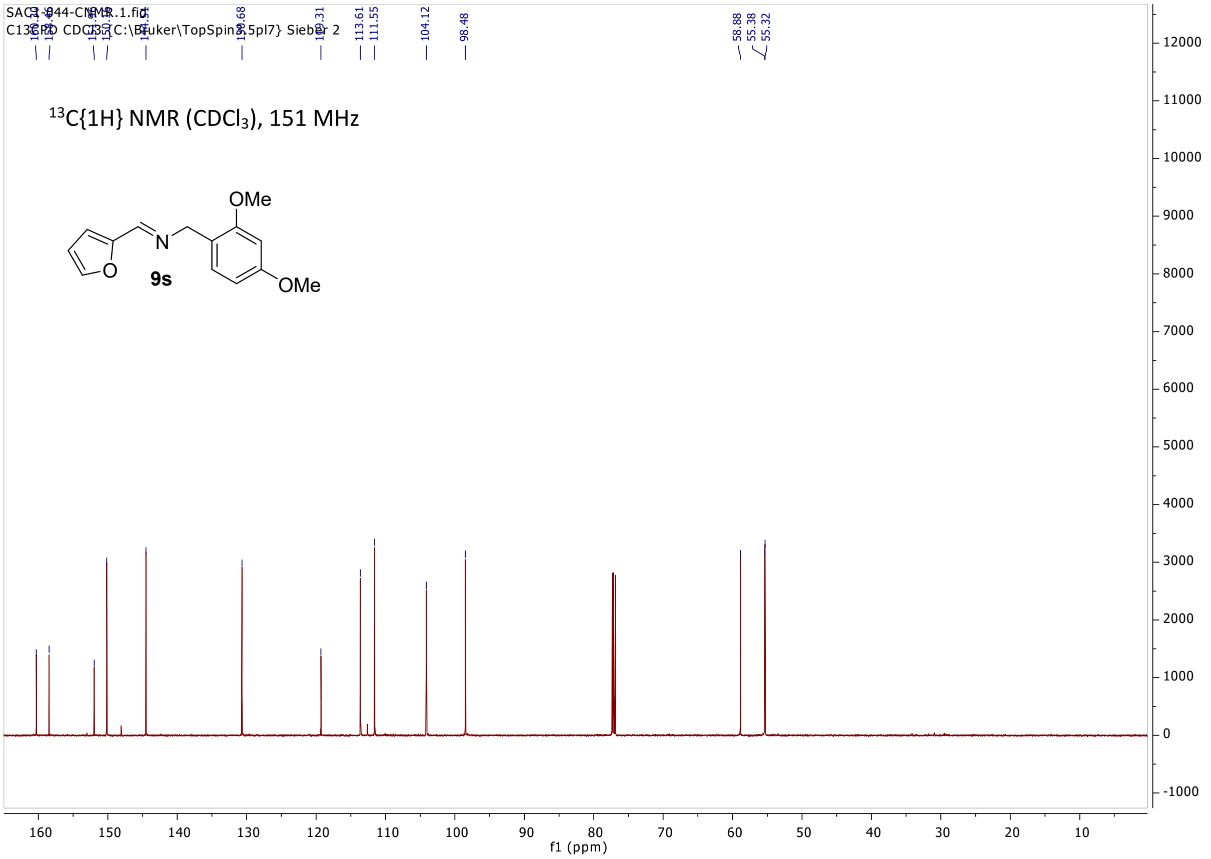

SAC1-044-CNMR.1.fid
C13CPD CDCl3 {C:\Bruker\TopSpin3.5pl7} Sieber 2
13C{1H} NMR (CDCl3), 151 MHz
9s
OMe
OMe
N
O
f1 (ppm)

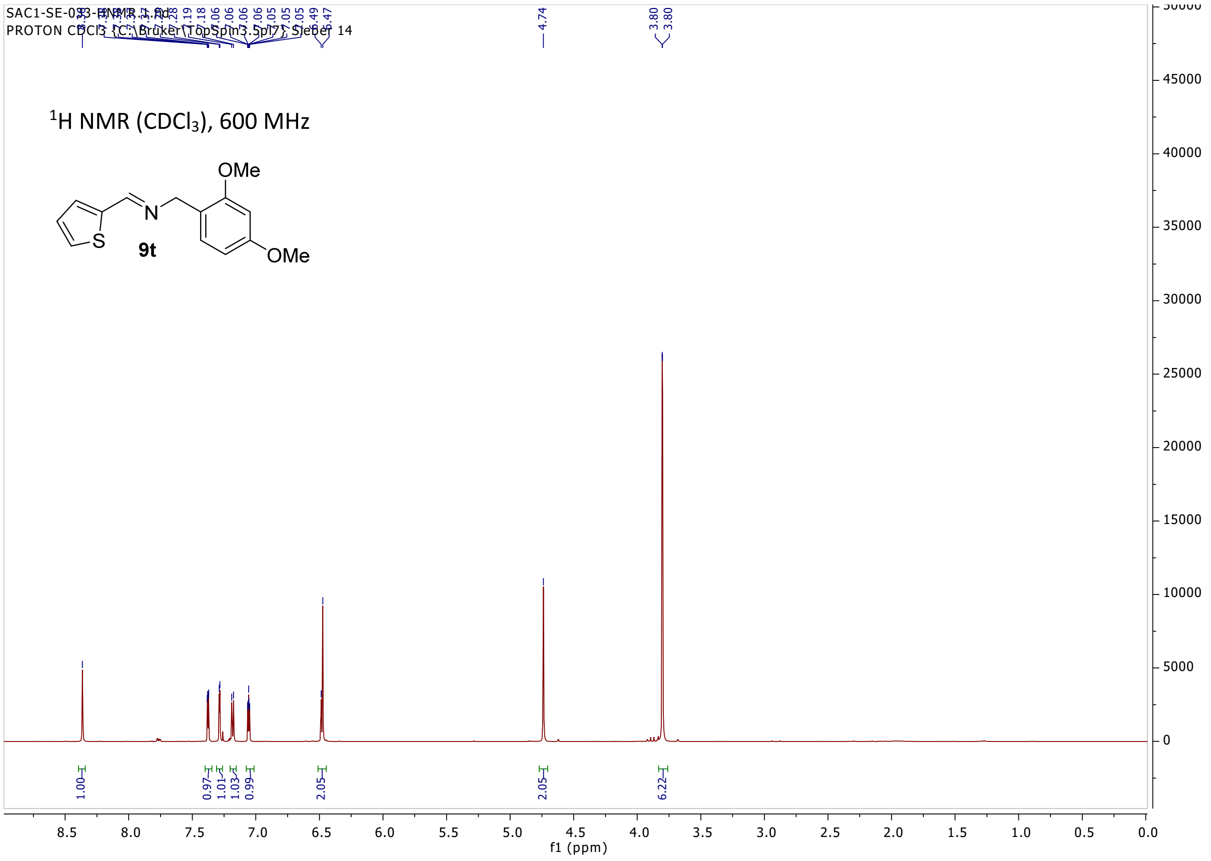

SAC1-SE-033-HNMR.1.fid
PROTON CDCl3 {C:\Bruker\TopSpin3.5pl7} Siebel 14
1H NMR (CDCl3), 600 MHz
OMe
N
S
9t
OMe
4.74
3.80
3.80
1.00
0.97
1.01
1.03
0.99
2.05
2.05
6.22
f1 (ppm)

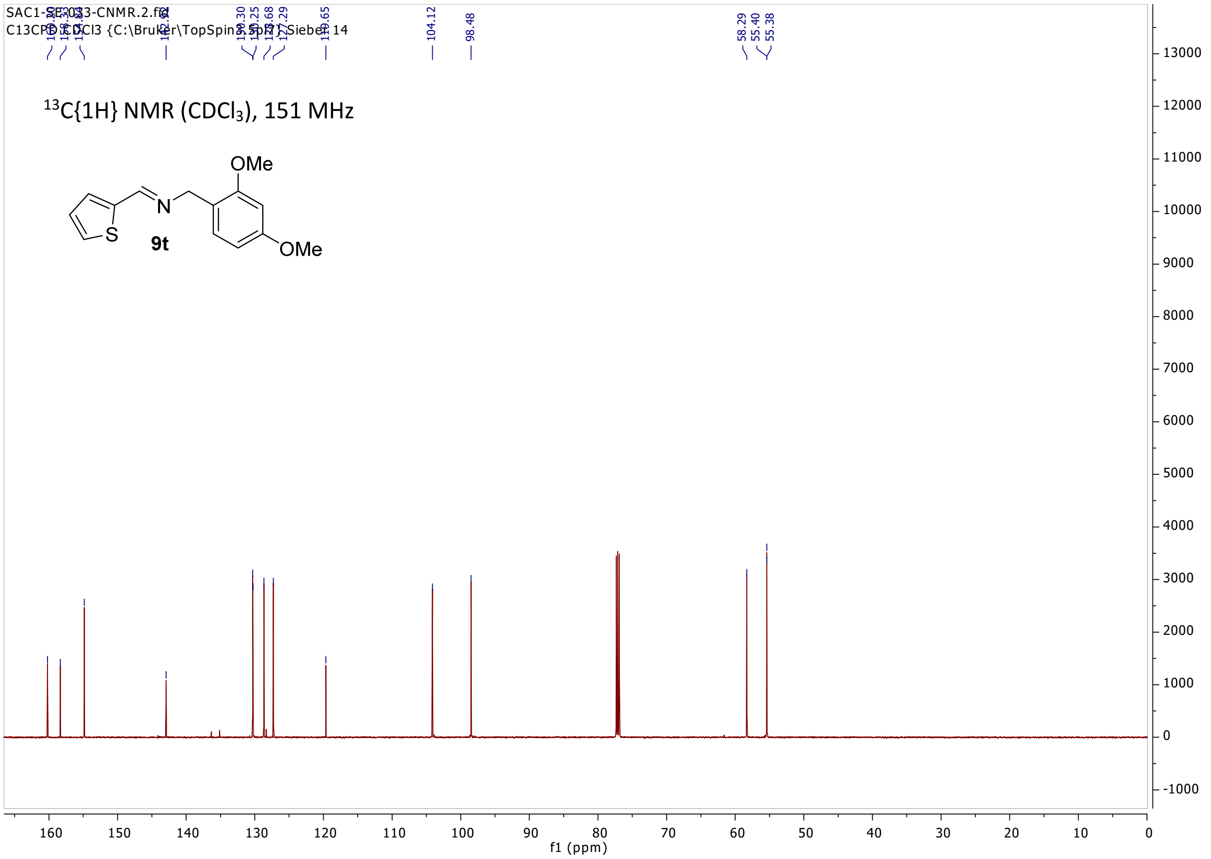

SAC1-SE-033-CNMR.2.fid
C13CPD CDCl3 {C:\Bruker\TopSpin3.5pl7} Siebel 14
13C{1H} NMR (CDCl3), 151 MHz
OMe
N
S
9t
OMe
130.30
130.25
128.68
127.29
119.65
104.12
98.48
58.29
55.40
55.38
f1 (ppm)

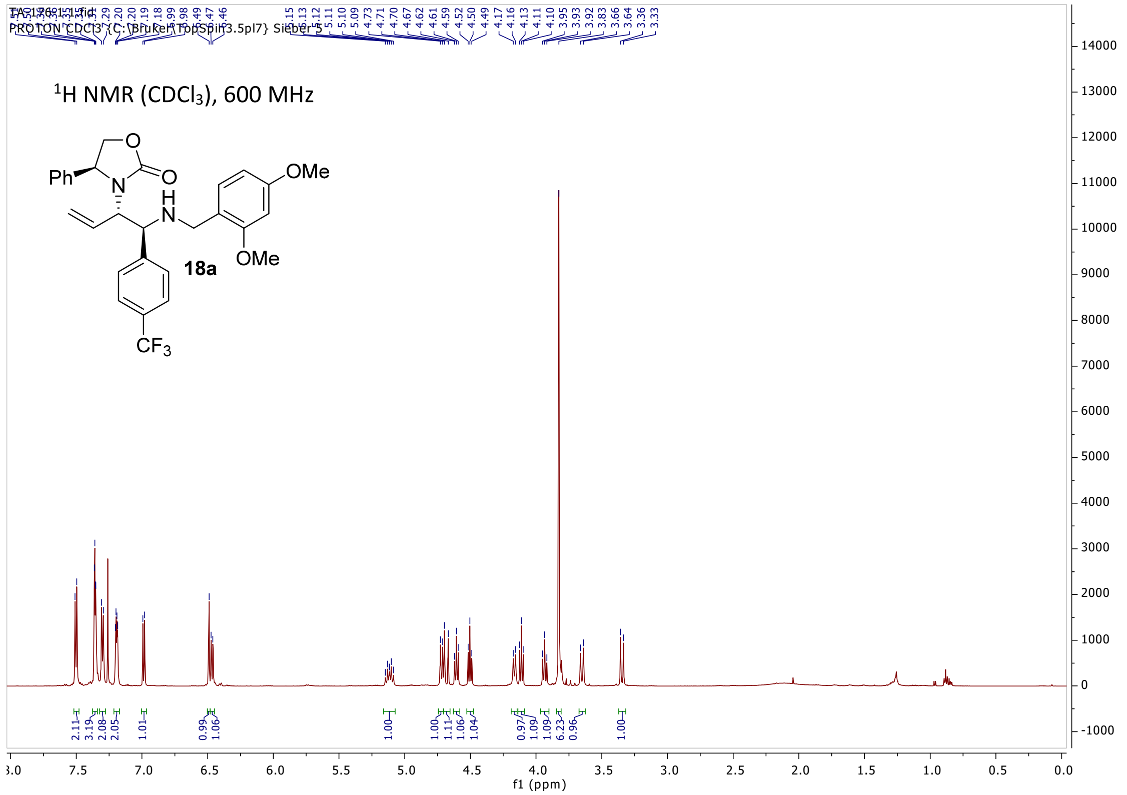

TA-176-AC-C13.1.fid
1H NMR (CDCl3), 600 MHz
Ph
N
O
O
H
N
OMe
OMe
18a
CF3
f1 (ppm)

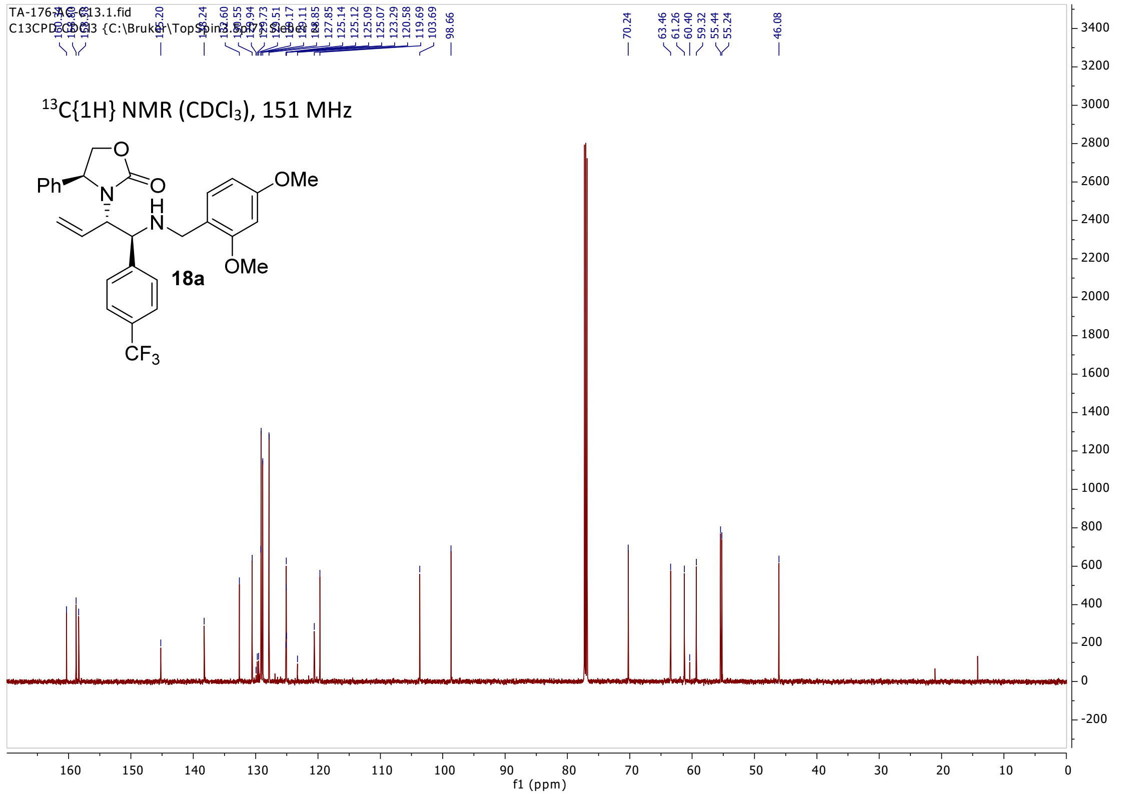

13C{1H} NMR (CDCl3), 151 MHz
Ph
N
O
O
H
N
OMe
OMe
18a
CF3
f1 (ppm)

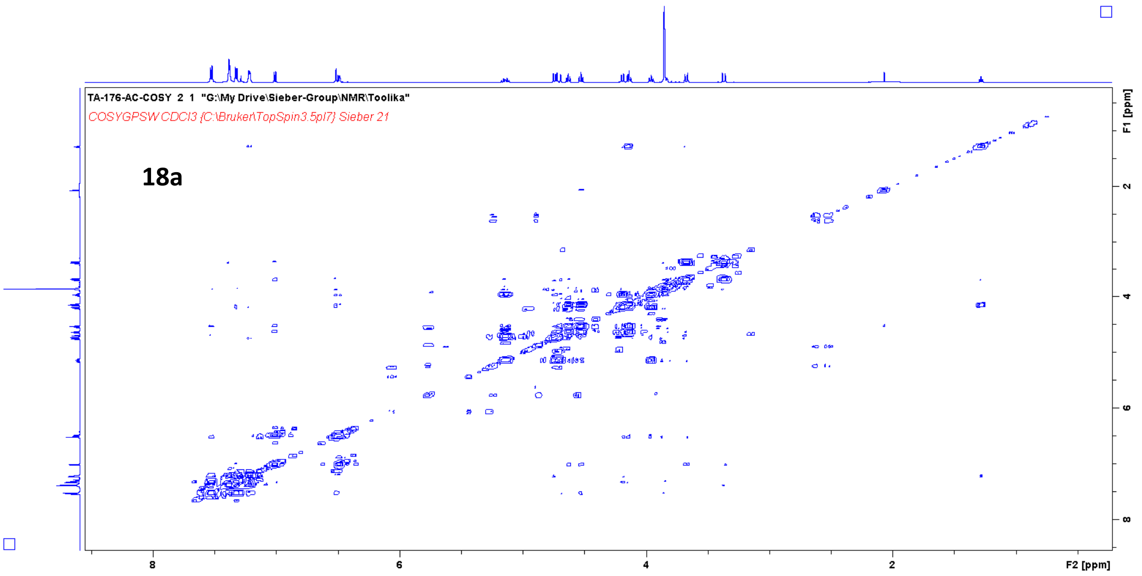
TA-176-AC-COSY 2 1 "G:\My Drive\Sieber-Group\NMR\Toolika"
COSYGPSW CDCl3 {C:\Bruker\TopSpin3.5pl7} Sieber 21
18a
F1 [ppm]
F2 [ppm]

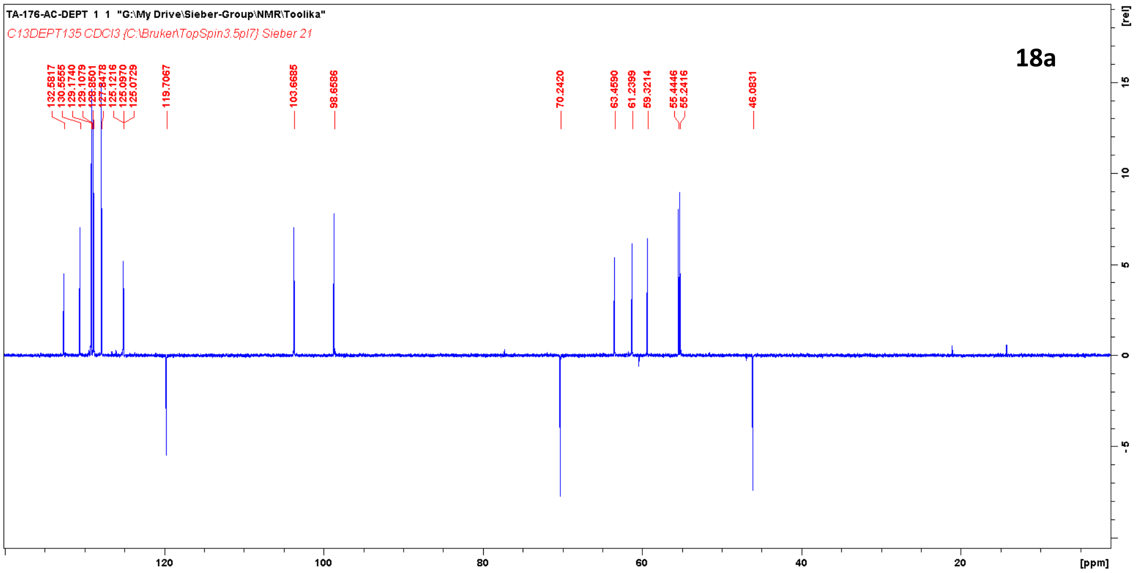
TA-176-AC-DEPT 1 1 "G:\My Drive\Sieber-Group\NMR\Toolika"
C13DEPT135 CDCl3 {C:\Bruker\TopSpin3.5pl7} Sieber 21
18a
132.5817
130.5555
129.1740
129.1079
128.8501
127.8478
125.1216
125.0970
125.0729
119.7067
103.6685
98.6586
70.2420
63.4590
61.2399
59.3214
55.4446
55.2416
46.0831
[rel]
[ppm]

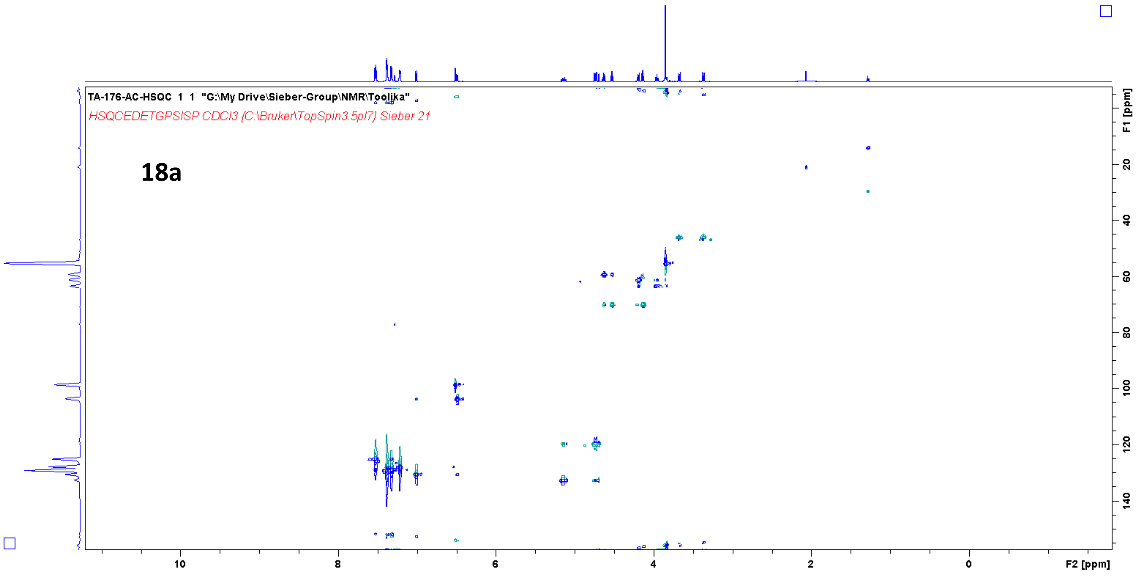
TA-176-AC-HSQC 1 1 "G:\My Drive\Sieber-Group\NMR\Toolika"
HSQCEDETGPSISP CDCl3 {C:\Bruker\TopSpin3.5pl7} Sieber 21
18a
F1 [ppm]
F2 [ppm]

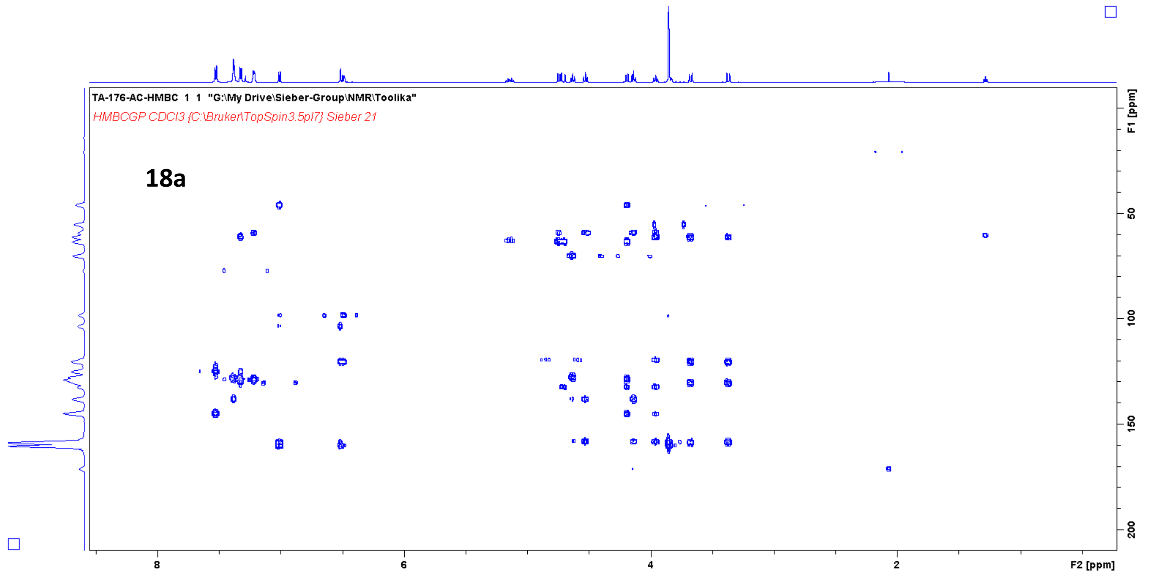
TA-176-AC-HMBC 1 1 "G:\My Drive\Sieber-Group\NMR\Toolika"
HMBCGP CDCl3 {C:\Bruker\TopSpin3.5pl7} Sieber 21
18a
F1 [ppm]
F2 [ppm]

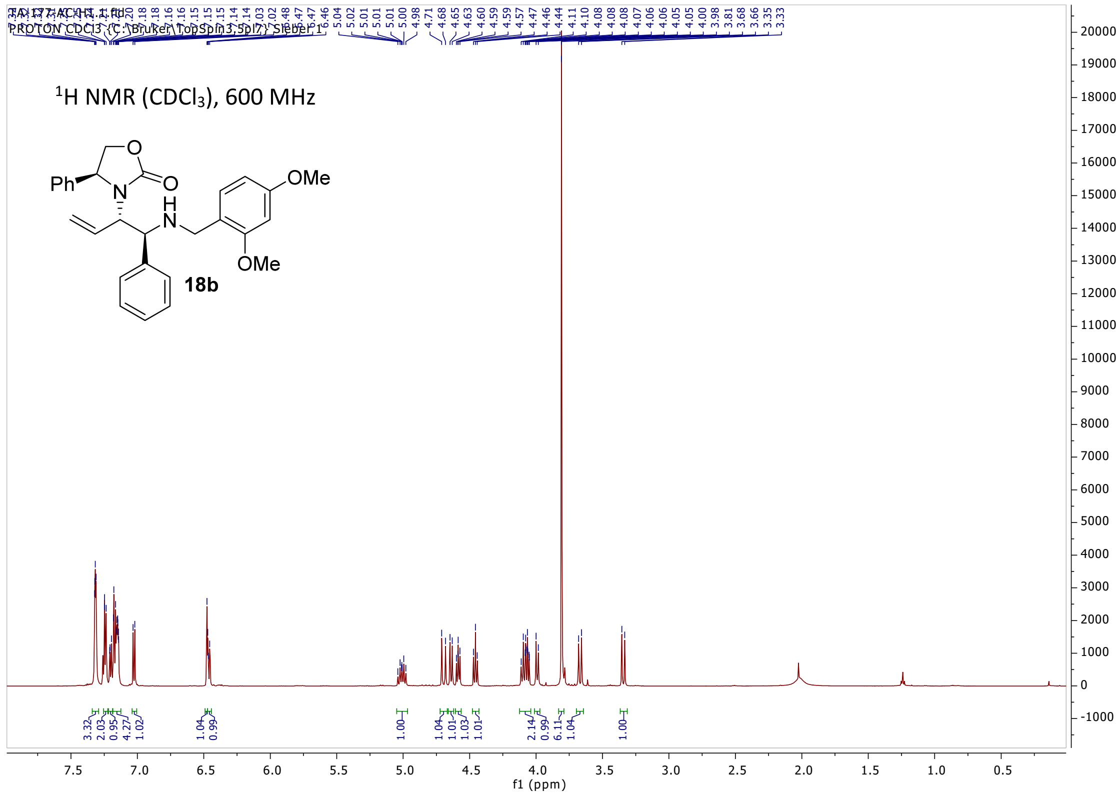
PROTON CDCl3 {C:\Bruker\TopSpin3.5pl7} Sieber 1
^{1}H NMR (CDCl$_3$), 600 MHz
Ph
O
N
H
N
OMe
OMe
18b
f1 (ppm)

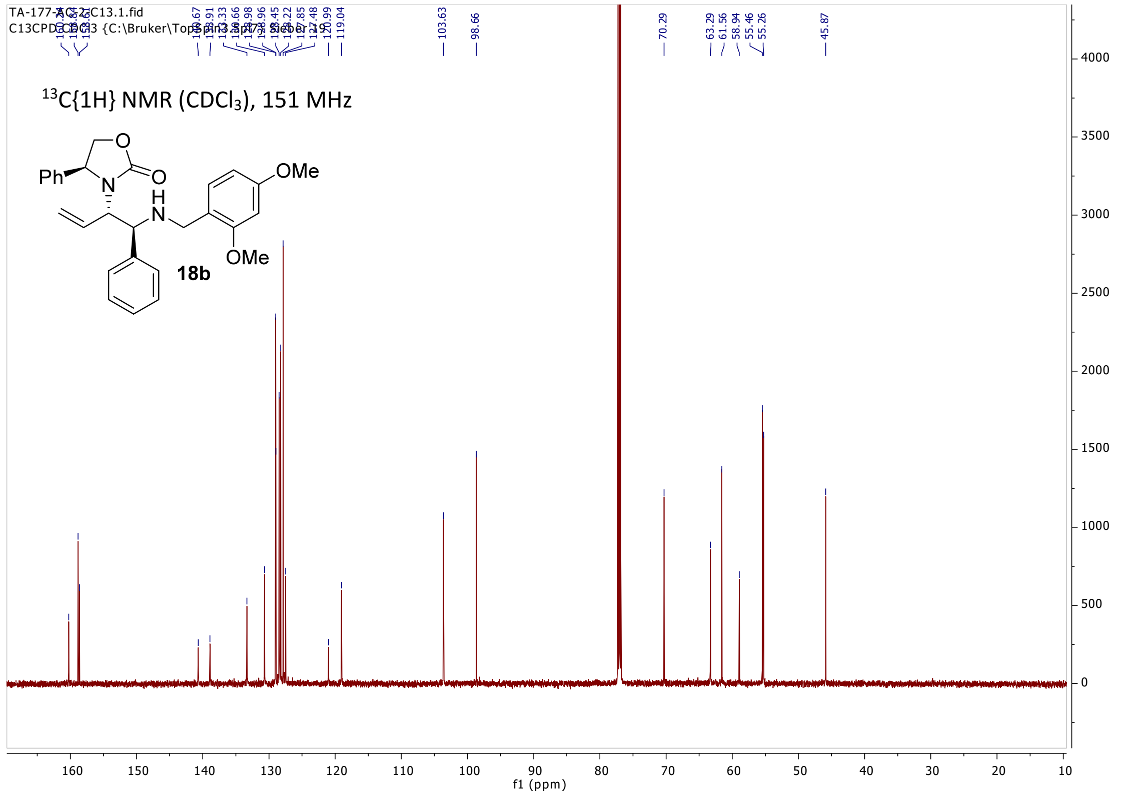
TA-177-AC-2-C13.1.fid
13C{1H} NMR (CDCl3), 151 MHz
Ph
OMe
OMe
18b
f1 (ppm)

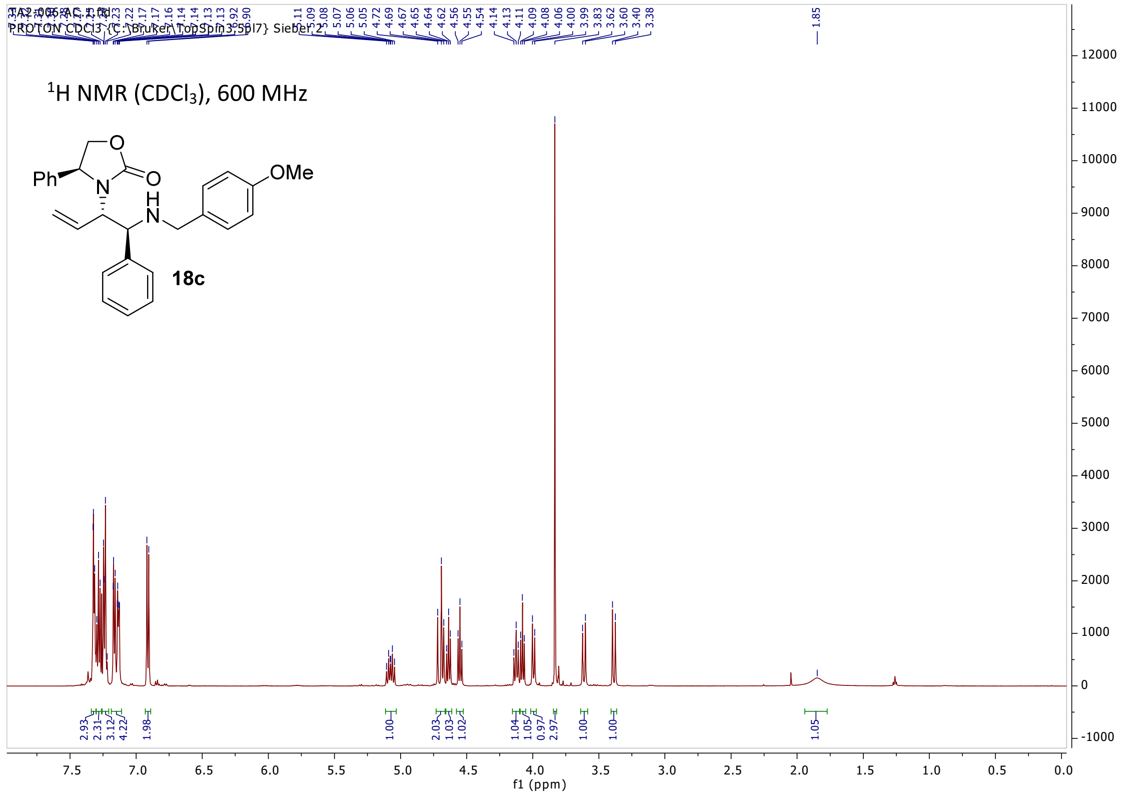
1H NMR (CDCl3), 600 MHz
Ph
OMe
18c
f1 (ppm)

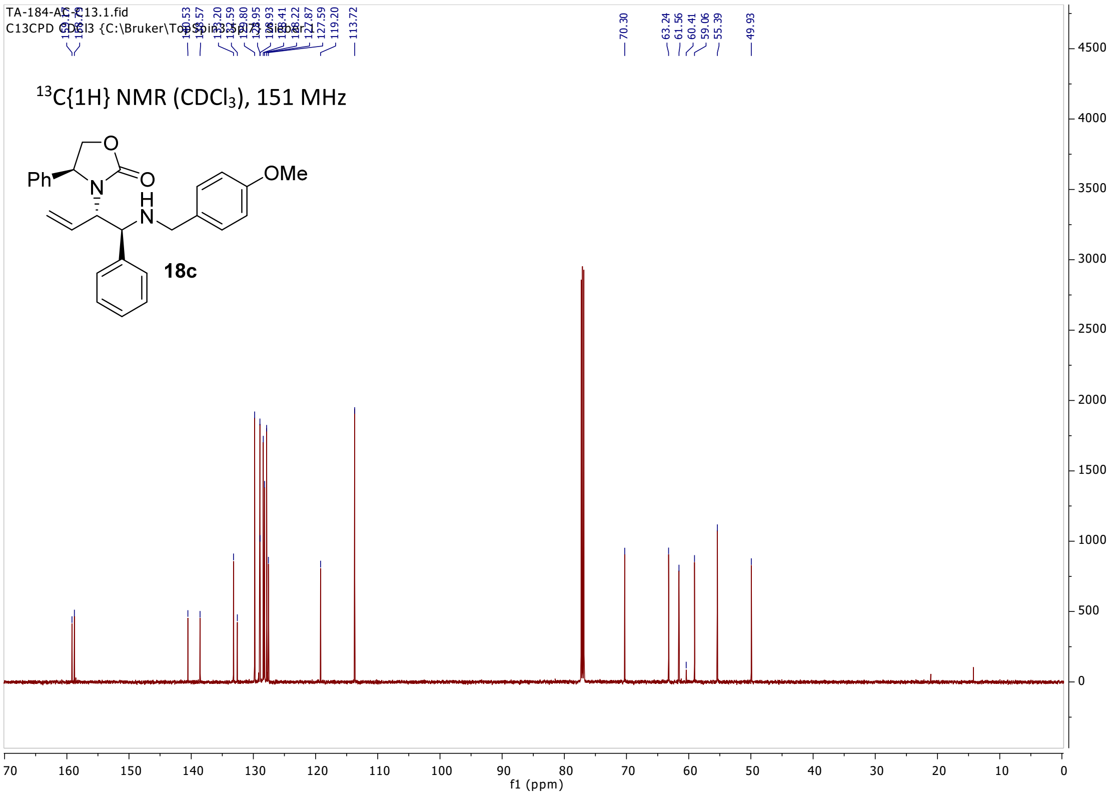
TA-184-AC-C13.1.fid
13C{1H} NMR (CDCl3), 151 MHz
Ph
N
O
O
H
N
OMe
18c
70.30
63.24
61.56
60.41
59.06
55.39
49.93
119.20
113.72
f1 (ppm)

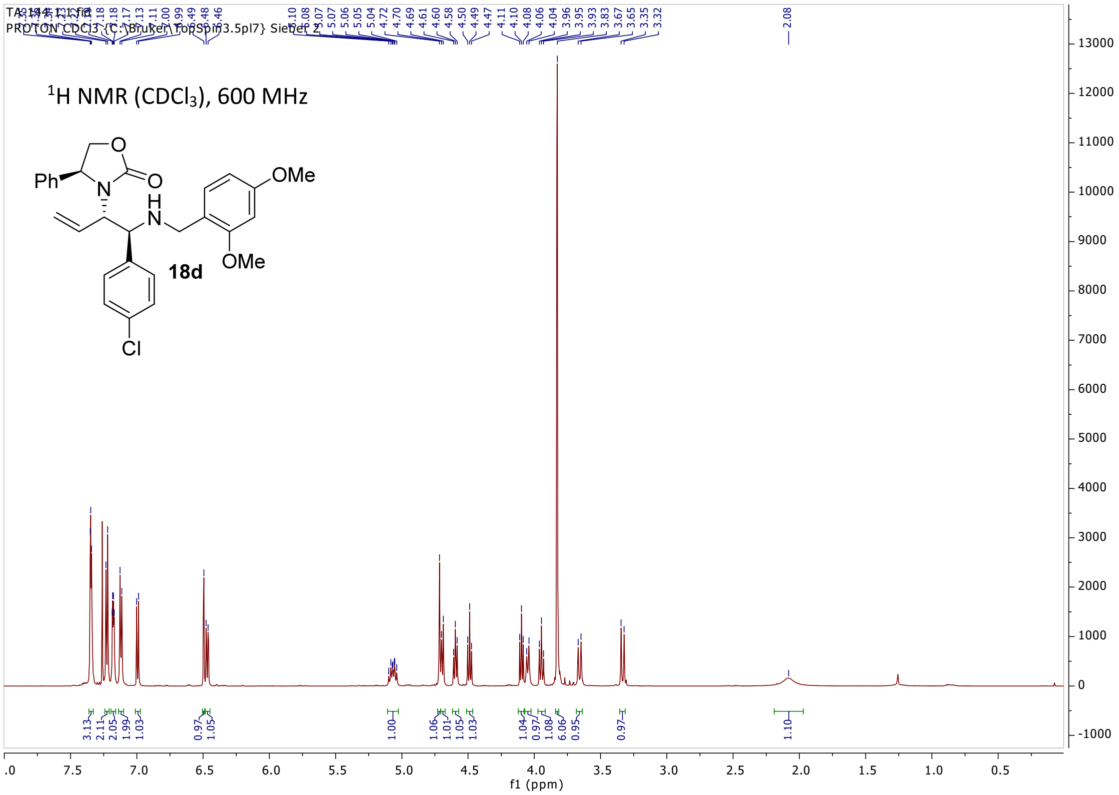
PROTON CDCl3 {C:\Bruker\TopSpin3.5pl7} Siebel 2
1H NMR (CDCl3), 600 MHz
Ph
N
O
O
H
N
OMe
OMe
18d
Cl
2.08
f1 (ppm)

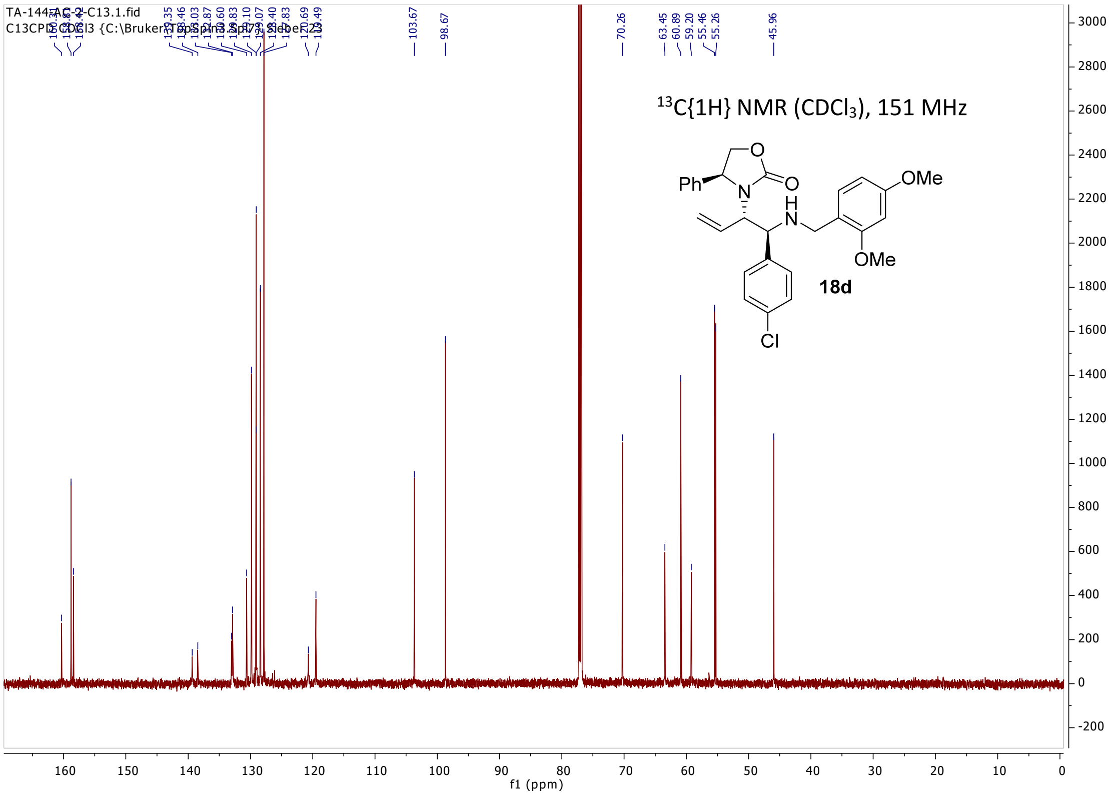

TA-144-AC-2-C13.1.fid
13C{1H} NMR (CDCl3), 151 MHz
Ph
N
O
O
H
N
OMe
OMe
Cl
18d
f1 (ppm)

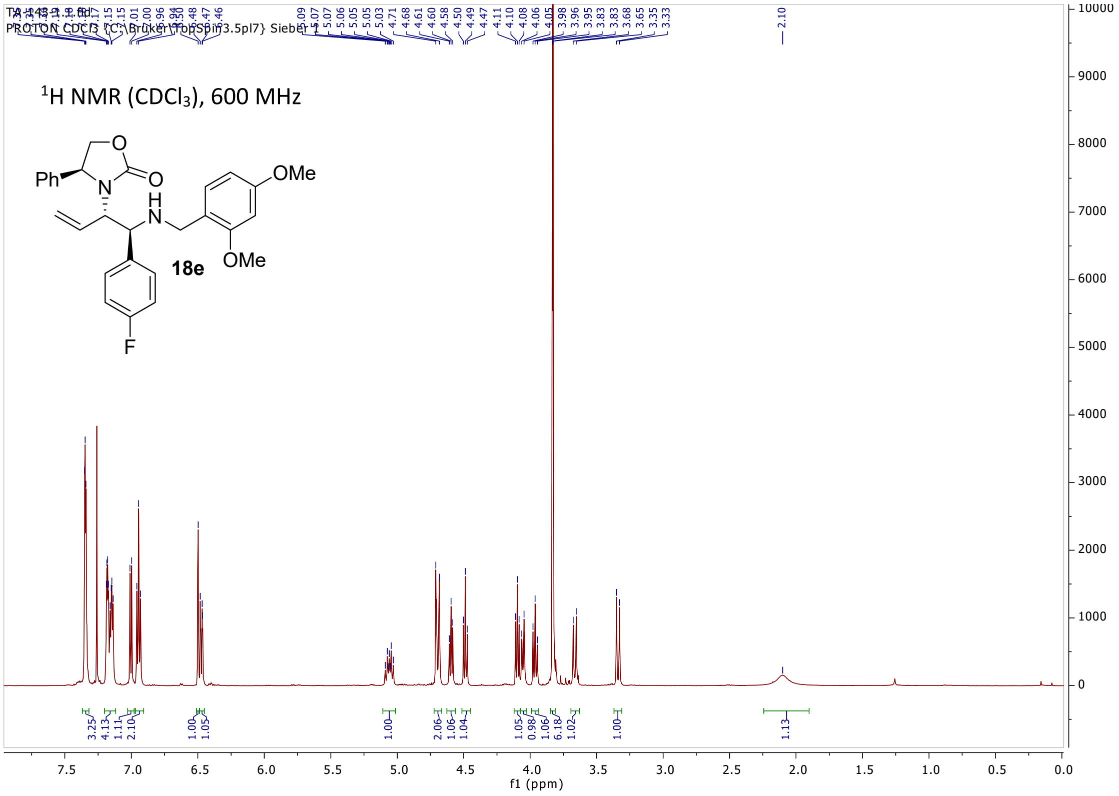

PROTON CDCl3
1H NMR (CDCl3), 600 MHz
Ph
N
O
O
H
N
OMe
OMe
F
18e
f1 (ppm)

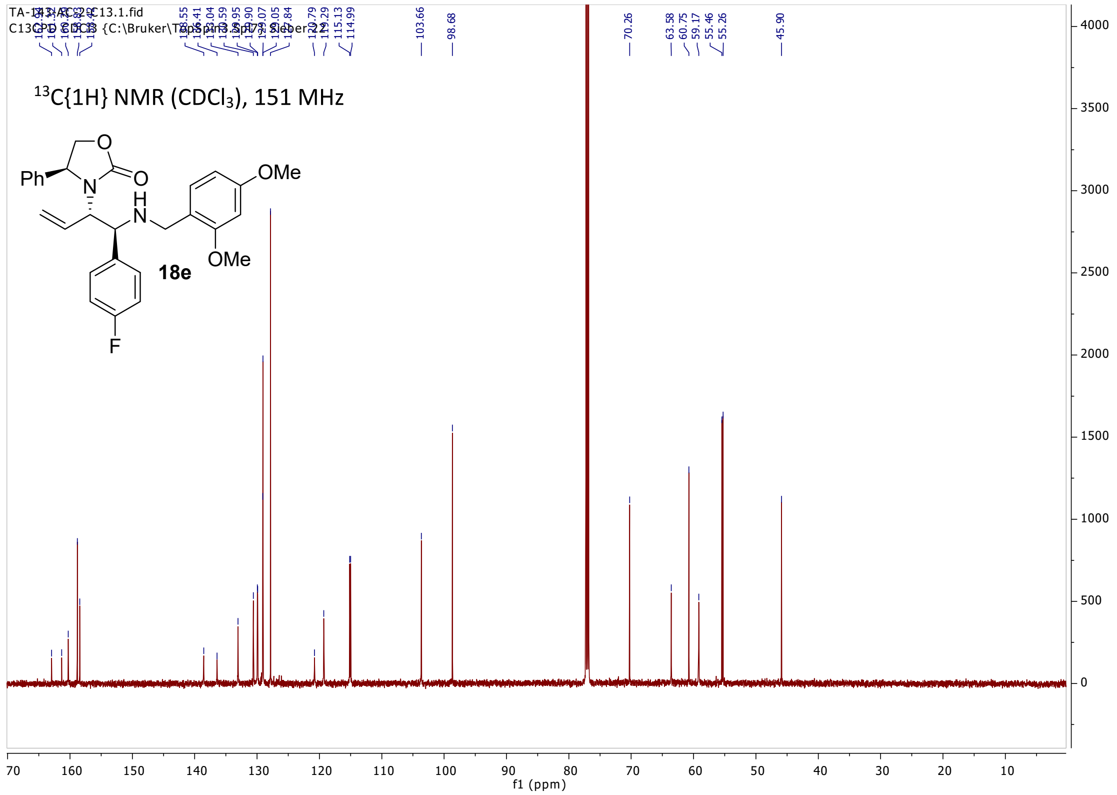
TA-143.AC.29.C13.1.fid
13C{1H} NMR (CDCl3), 151 MHz
Ph
N
O
O
H
N
OMe
OMe
18e
F
103.66
98.68
70.26
63.58
60.75
59.17
55.46
55.26
45.90
f1 (ppm)

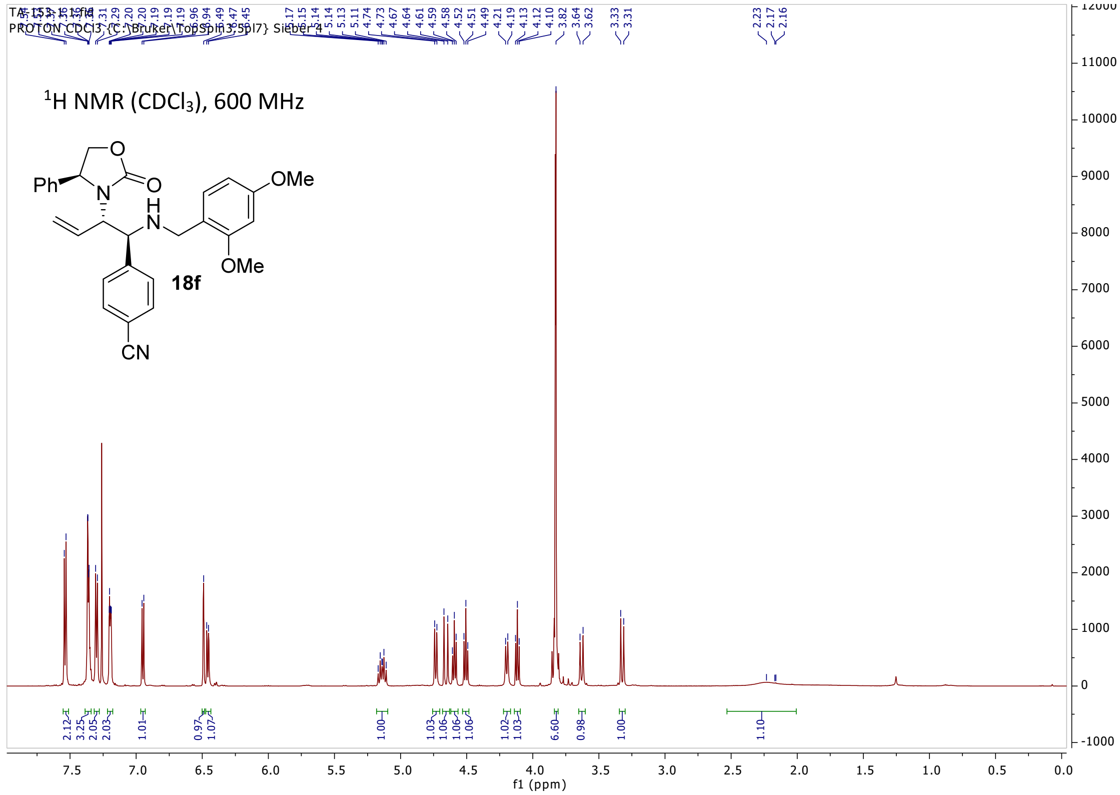
PROTON CDCl3 {C:\Bruker\TopSpin3.5pl7} Sieber 4
1H NMR (CDCl3), 600 MHz
Ph
N
O
O
H
N
OMe
OMe
18f
CN
f1 (ppm)

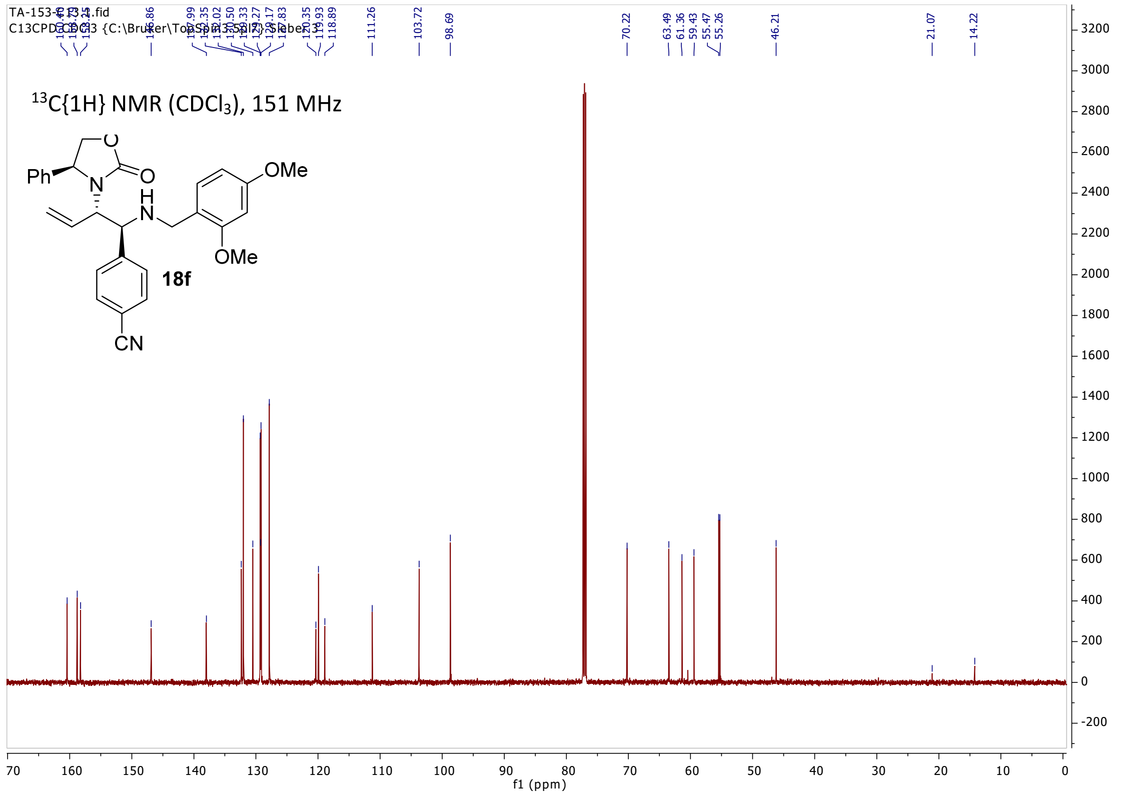
TA-153-C13.1.fid
13C{1H} NMR (CDCl3), 151 MHz
18f
f1 (ppm)

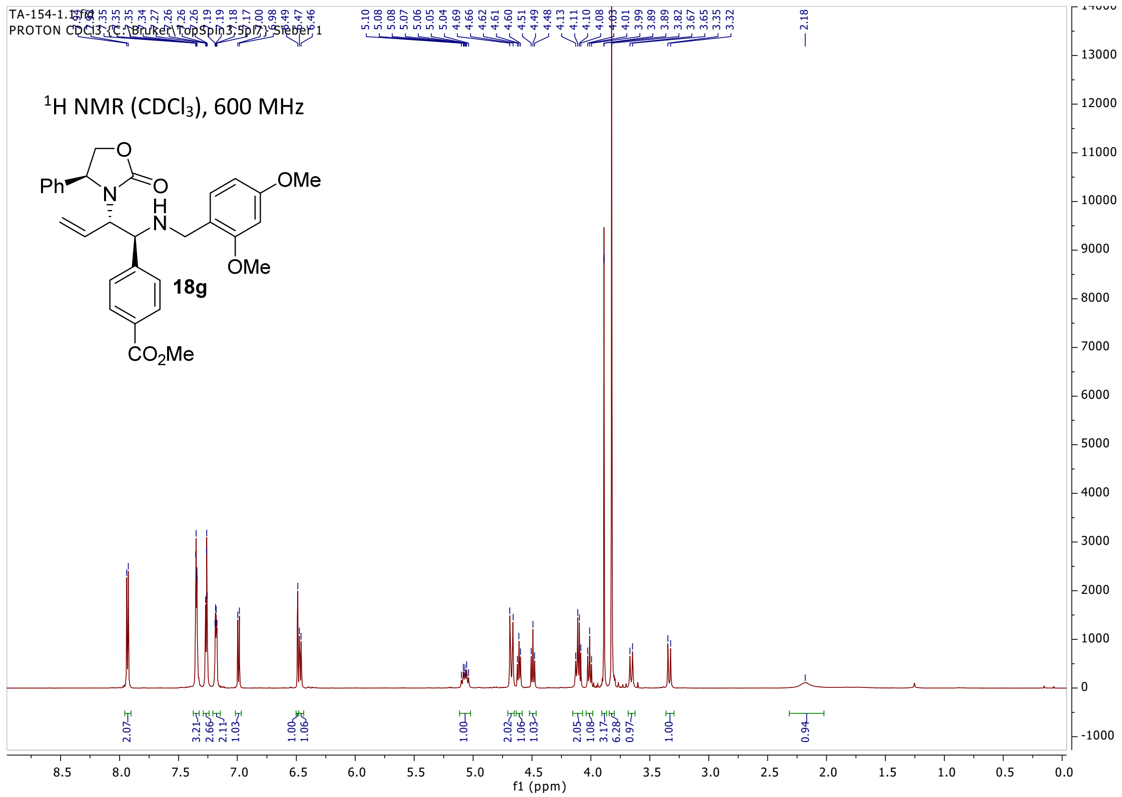
TA-154-1.1.fid
1H NMR (CDCl3), 600 MHz
18g
f1 (ppm)

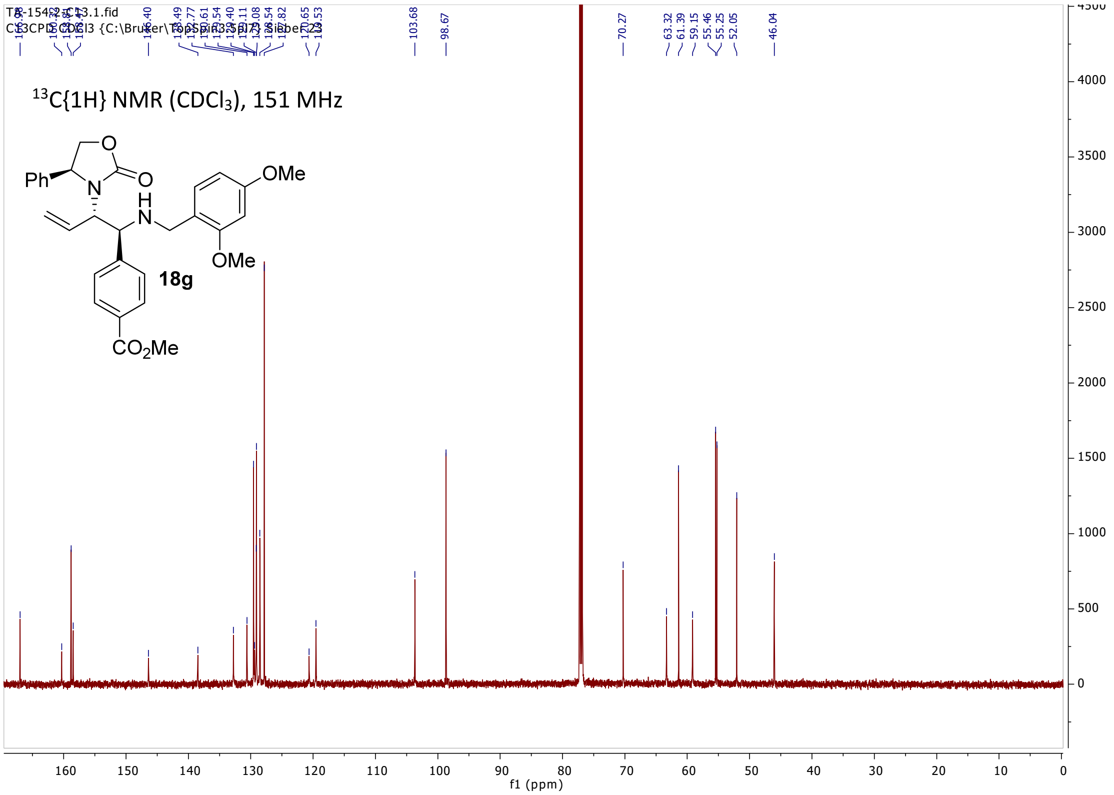

13C{1H} NMR (CDCl3), 151 MHz
Ph
N
O
O
H
N
OMe
OMe
18g
CO2Me
103.68
98.67
70.27
63.32
61.39
59.15
55.46
55.25
52.05
46.04
f1 (ppm)

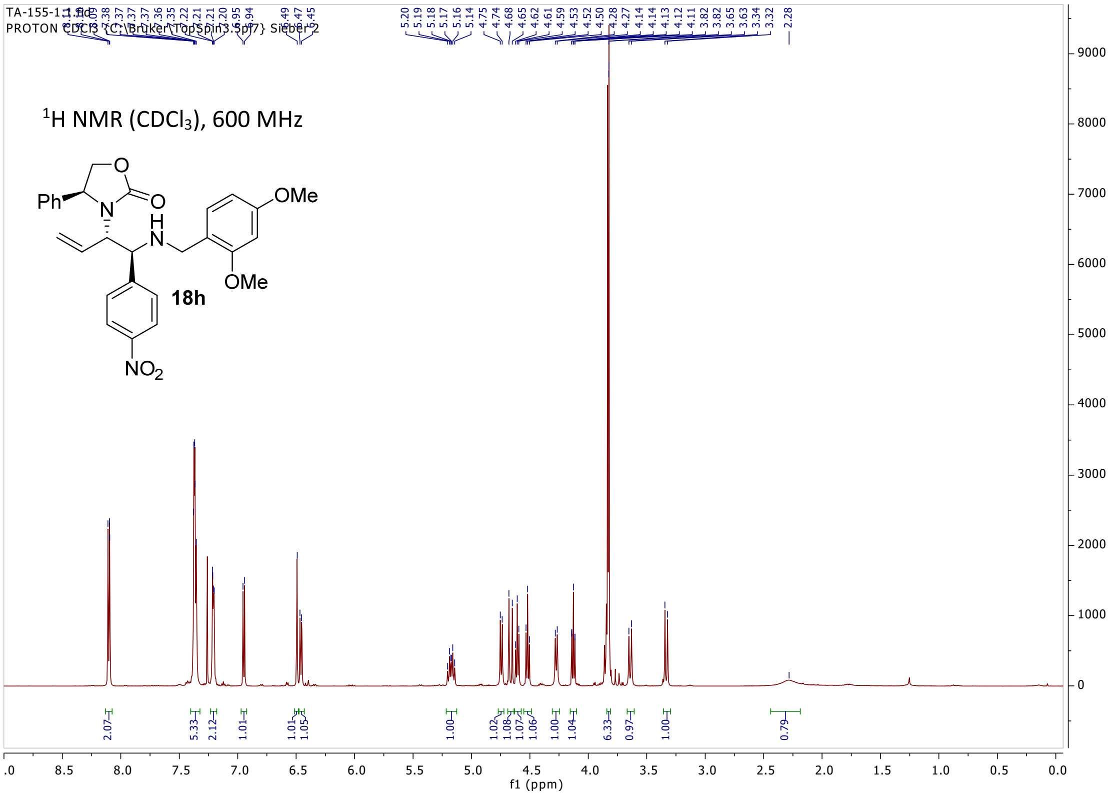

TA-155-1.1.fid
1H NMR (CDCl3), 600 MHz
Ph
N
O
O
H
N
OMe
OMe
18h
NO2
5.20
5.19
5.18
5.17
5.16
5.14
4.75
4.74
4.68
4.65
4.62
4.61
4.59
4.53
4.52
4.50
4.28
4.27
4.14
4.14
4.13
4.12
4.11
3.82
3.82
3.65
3.63
3.34
3.32
2.28
2.07
5.33
2.12
1.01
1.01
1.05
1.00
1.02
1.08
1.07
1.06
1.00
1.04
6.33
0.97
1.00
0.79
f1 (ppm)

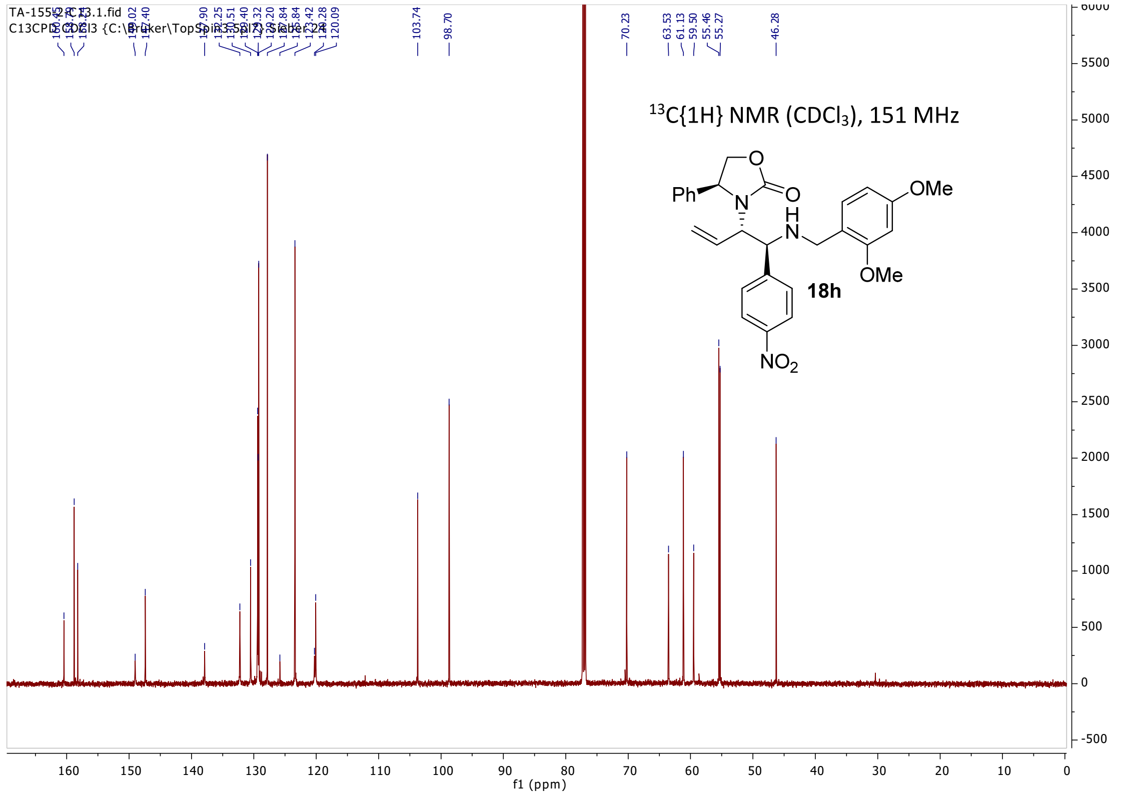

TA-155-2-C13.1.fid
^{13}C{1H} NMR (CDCl$_3$), 151 MHz
Ph
O
N
H
N
OMe
OMe
18h
NO$_2$
103.74
98.70
70.23
63.53
61.13
59.50
55.46
55.27
46.28
f1 (ppm)

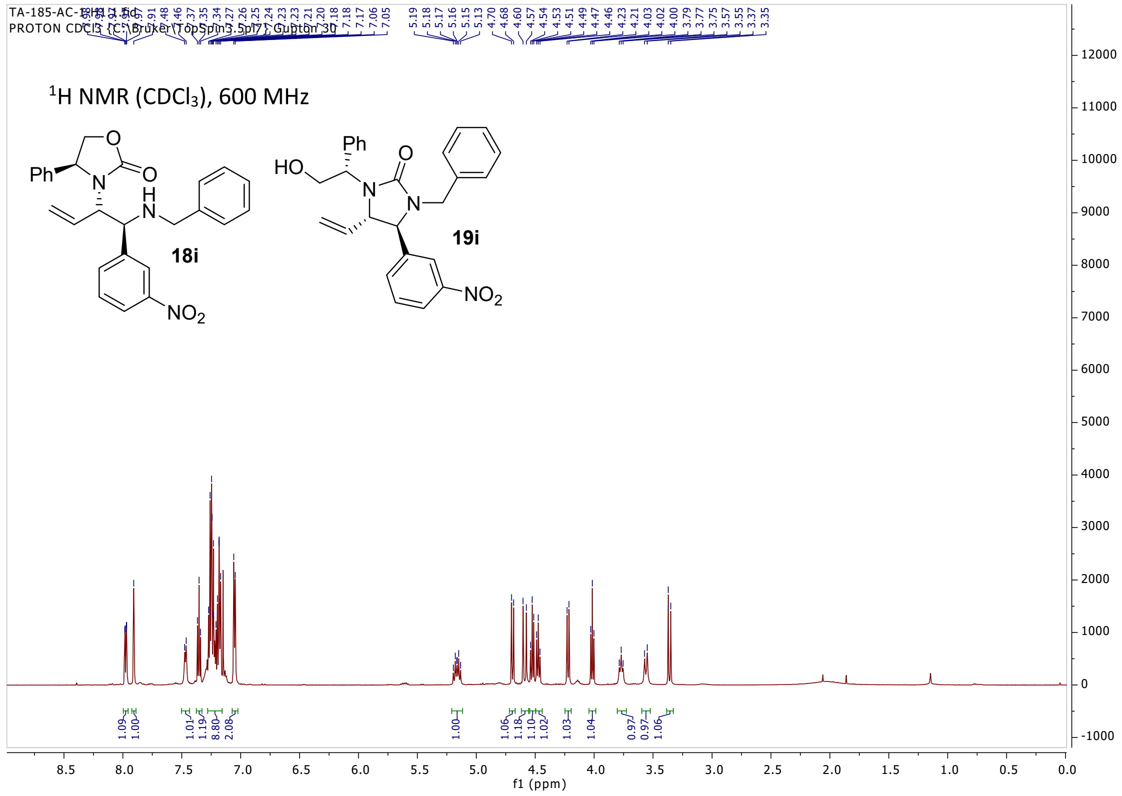

^{1}H NMR (CDCl$_3$), 600 MHz
Ph
O
N
H
N
18i
NO$_2$
HO
Ph
O
N
N
19i
NO$_2$
f1 (ppm)

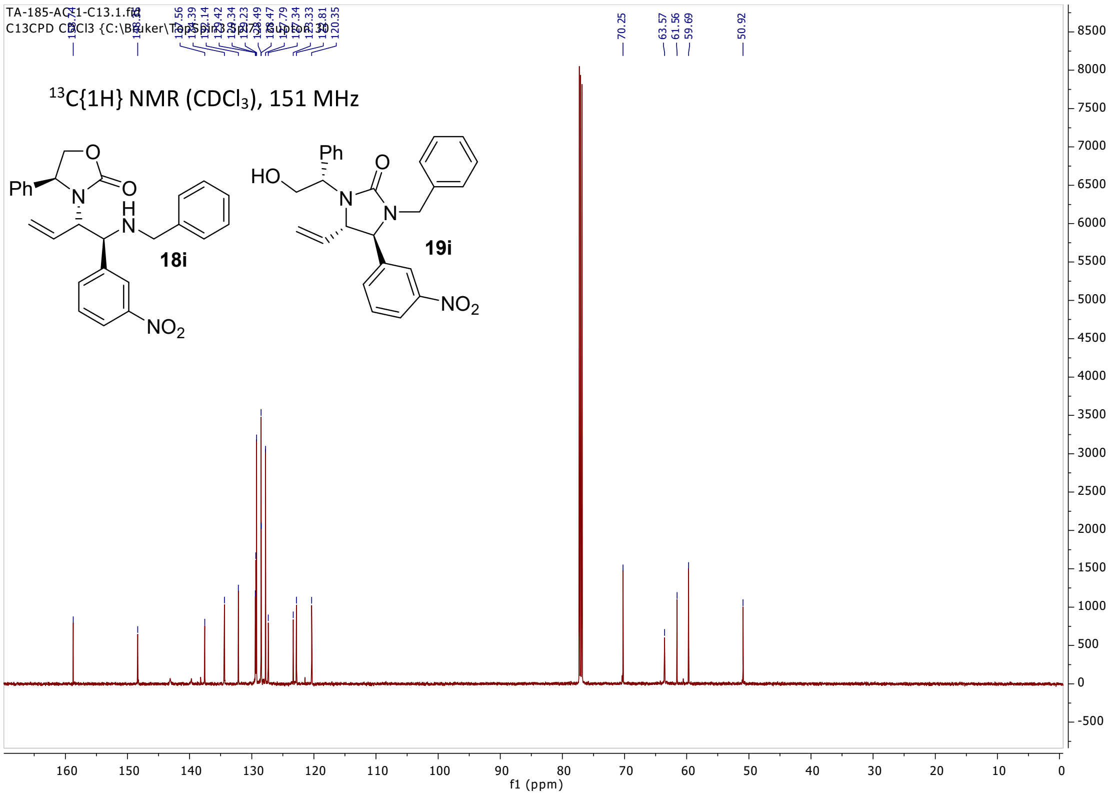

C13CPD CDCl3
^{13}C{1H} NMR (CDCl$_3$), 151 MHz
18i
19i
Ph
HO
NO$_2$
70.25
63.57
61.56
59.69
50.92
f1 (ppm)

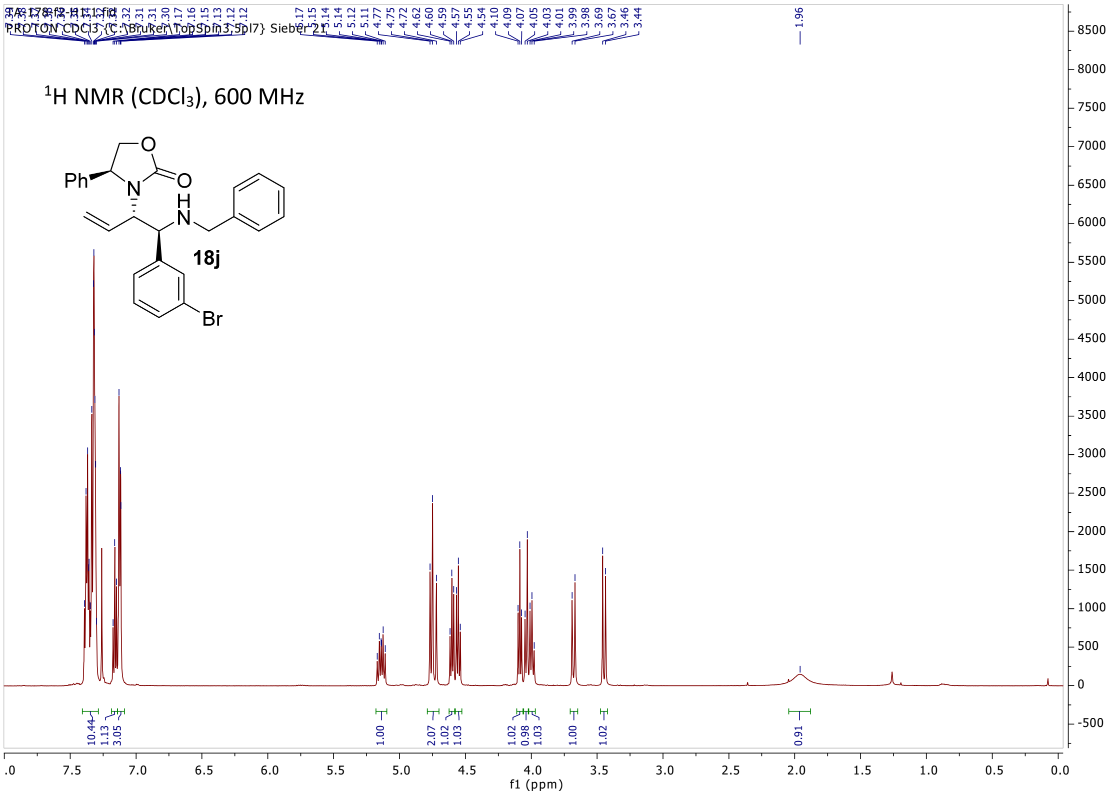

PROTON CDCl3
^{1}H NMR (CDCl$_3$), 600 MHz
18j
Ph
Br
1.96
10.44
1.13
3.05
1.00
2.07
1.02
1.03
1.02
0.98
1.03
1.00
1.02
0.91
f1 (ppm)

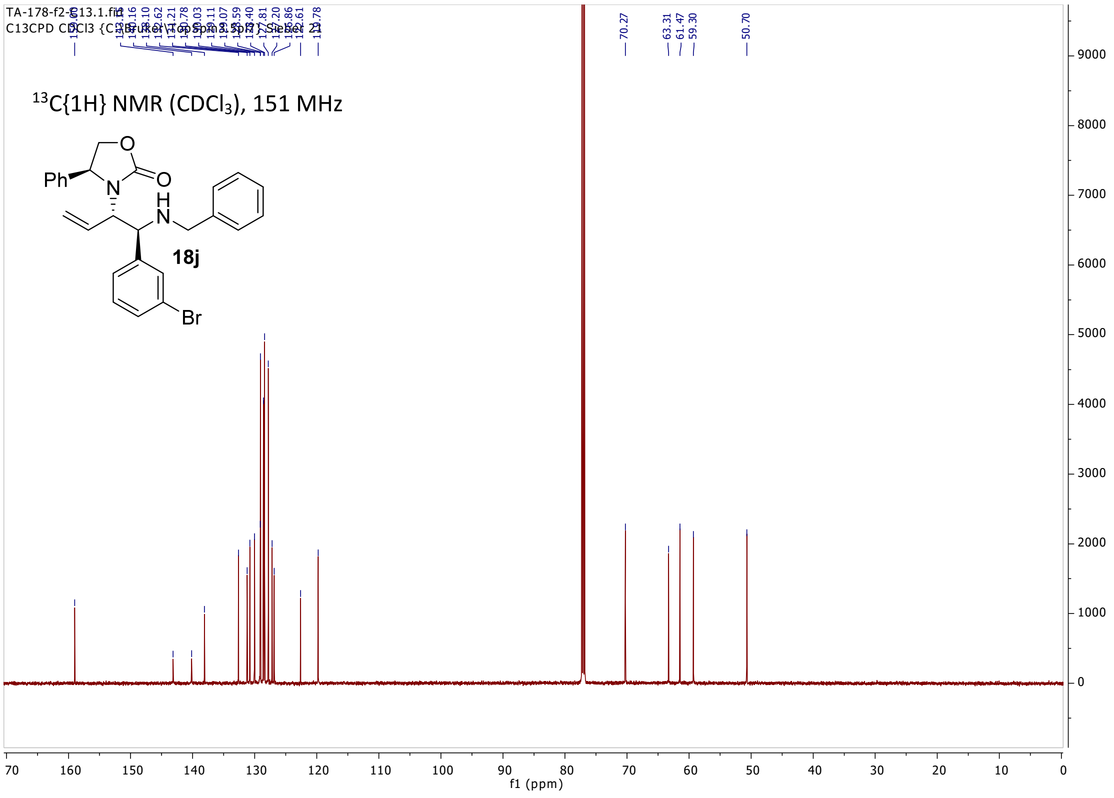
TA-178-f2-C13.1.fid
C13CPD CDCl3 {C:\Bruker\TopSpin3.5pl7} Siebel 21
^{13}C{1H} NMR (CDCl$_3$), 151 MHz
70.27
63.31
61.47
59.30
50.70
18j
Ph
Br
f1 (ppm)

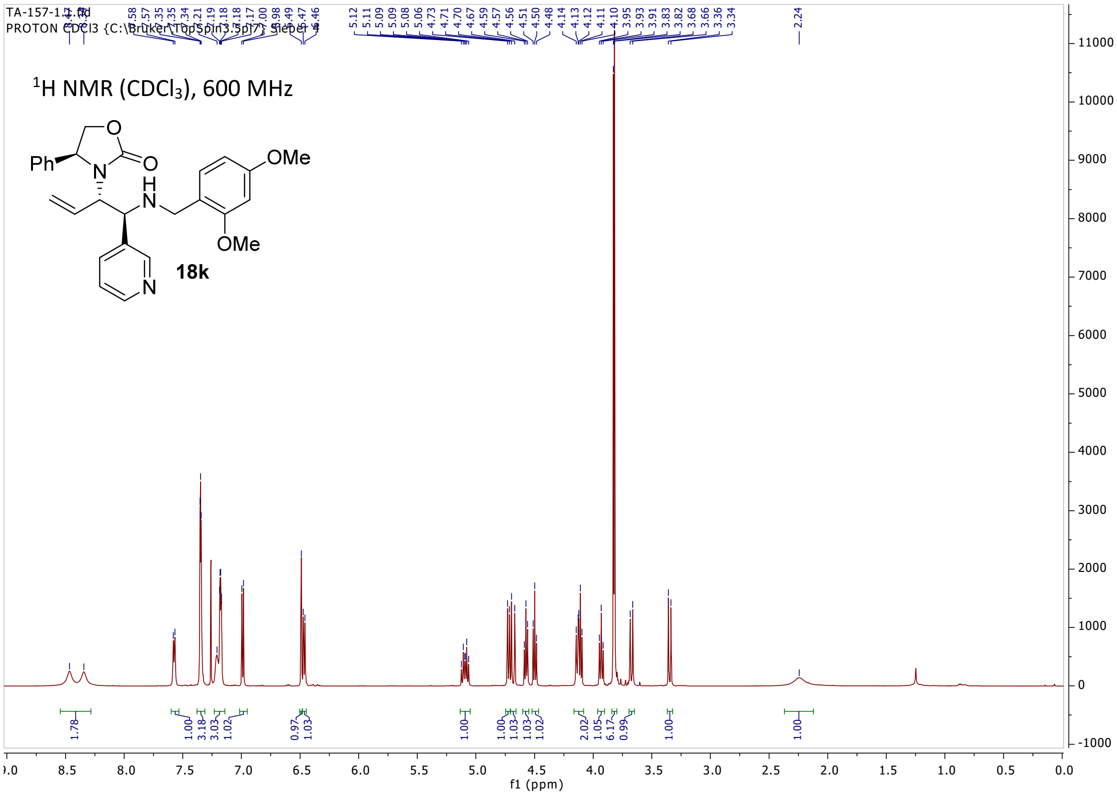
TA-157-1.1.fid
PROTON CDCl3 {C:\Bruker\TopSpin3.5pl7} Siebel 4
^{1}H NMR (CDCl$_3$), 600 MHz
OMe
OMe
Ph
18k
2.24
f1 (ppm)

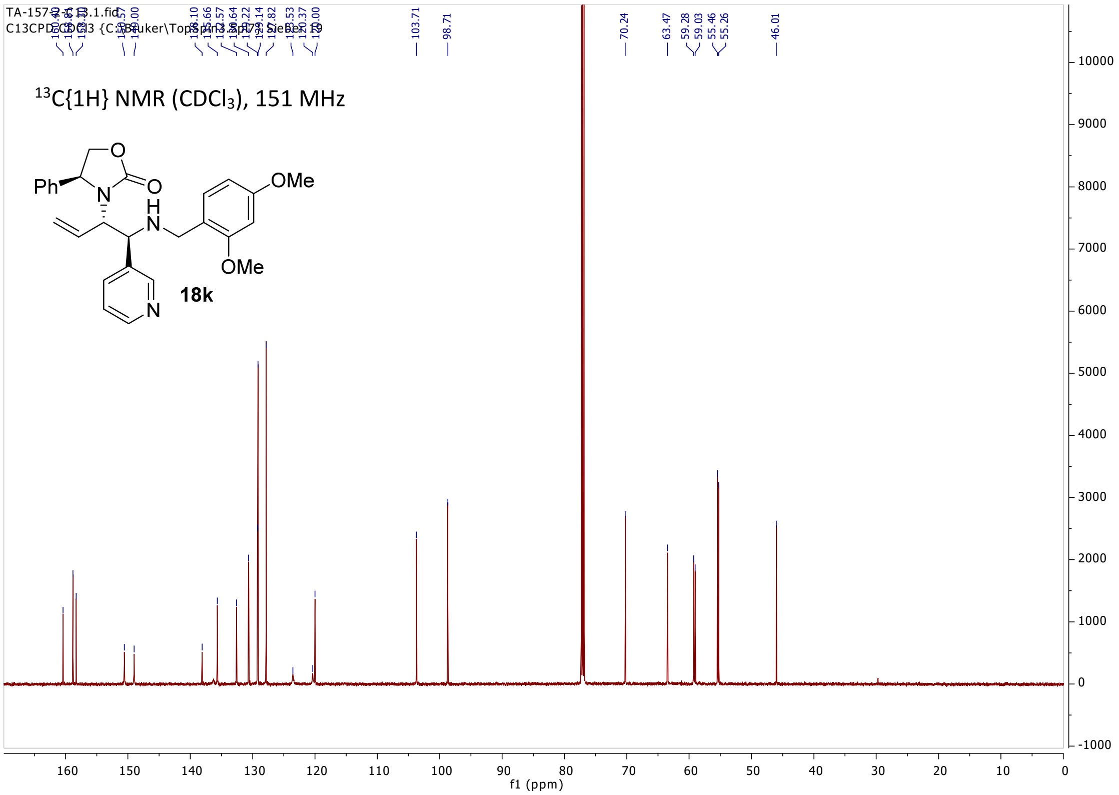

$^{13}C\{1H\}$ NMR ($CDCl_3$), 151 MHz
Ph
O
N
O
H
N
OMe
OMe
N
18k
103.71
98.71
70.24
63.47
59.28
59.03
55.46
55.26
46.01
f1 (ppm)

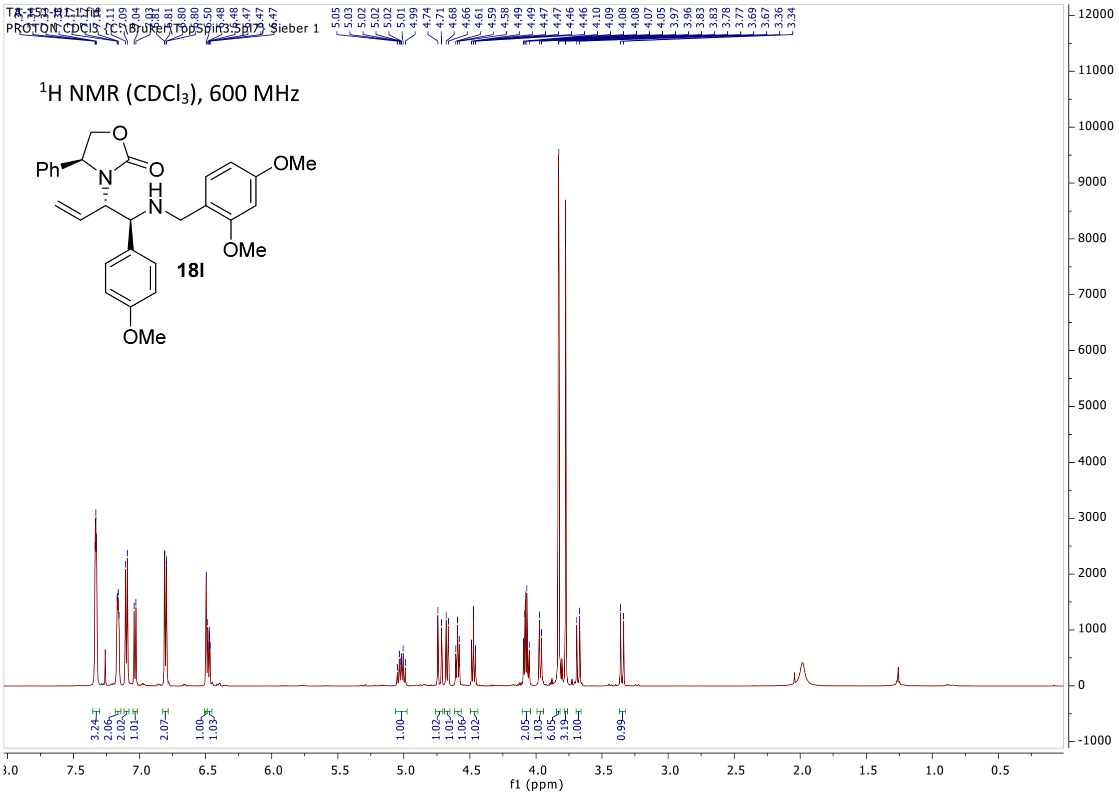

^{1}H NMR ($CDCl_3$), 600 MHz
Ph
O
N
O
H
N
OMe
OMe
OMe
18l
f1 (ppm)

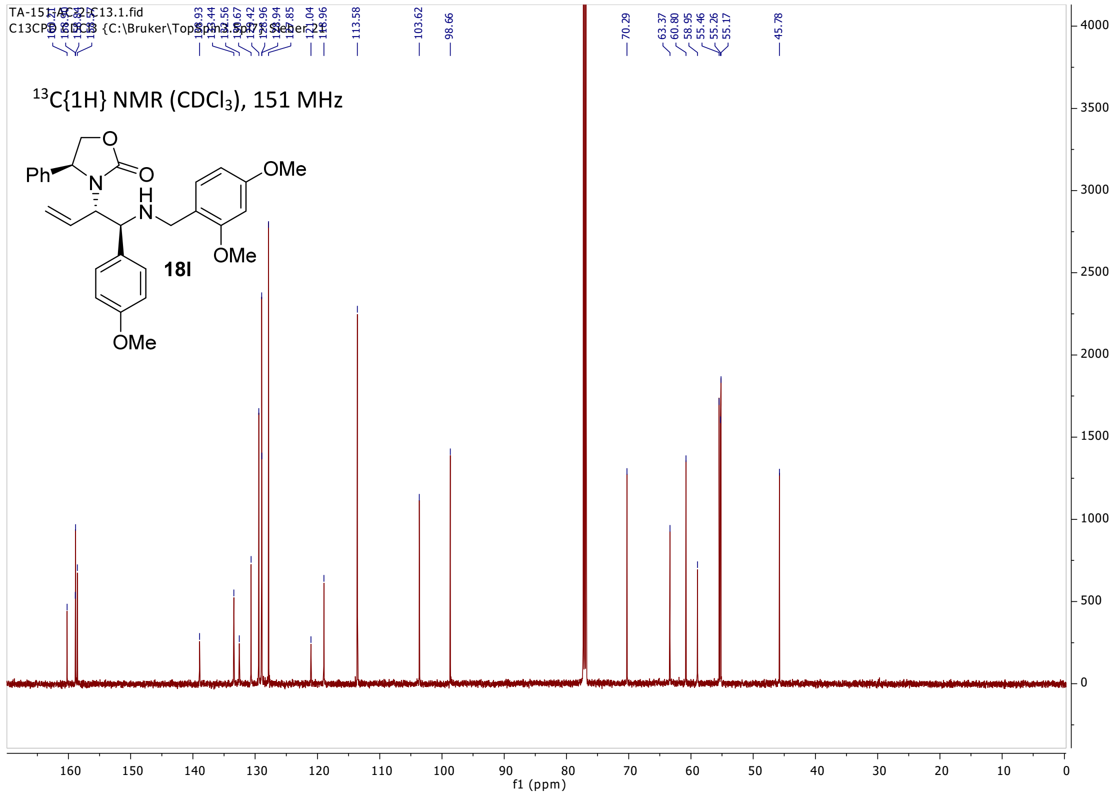
TA-151-AC2-C13.1.fid
^{13}C{1H} NMR (CDCl$_3$), 151 MHz
18l
f1 (ppm)

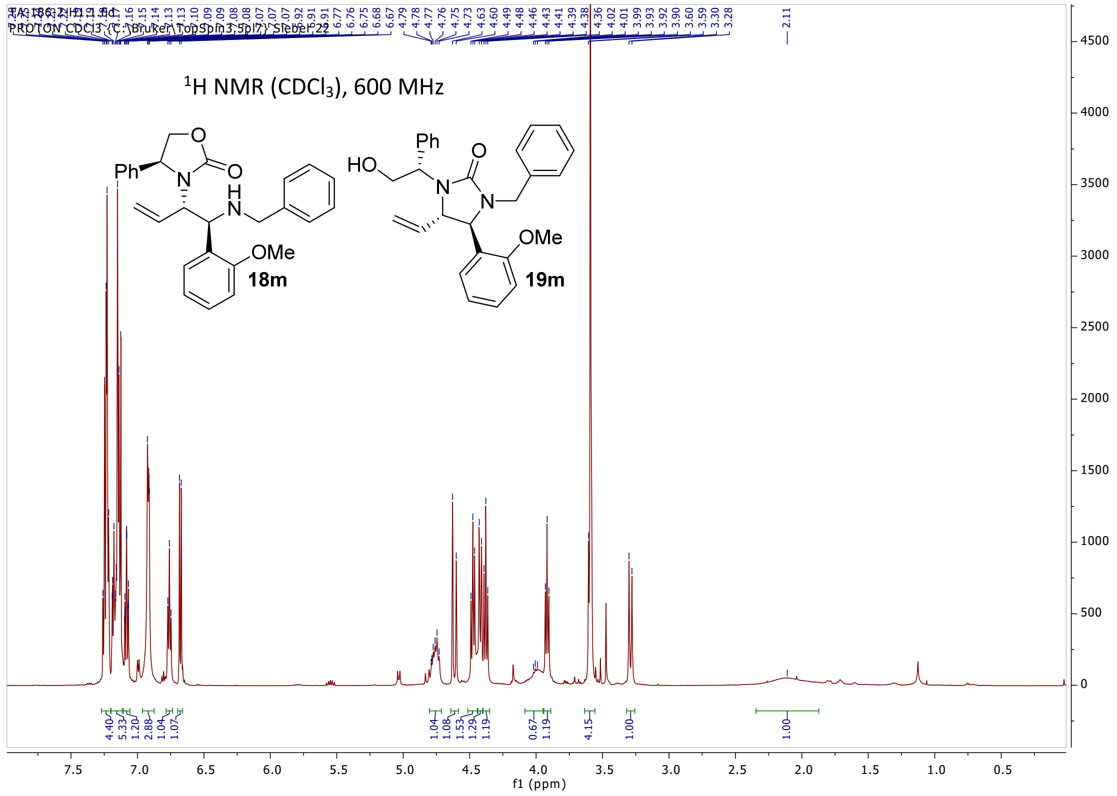
^{1}H NMR (CDCl$_3$), 600 MHz
18m
19m
f1 (ppm)

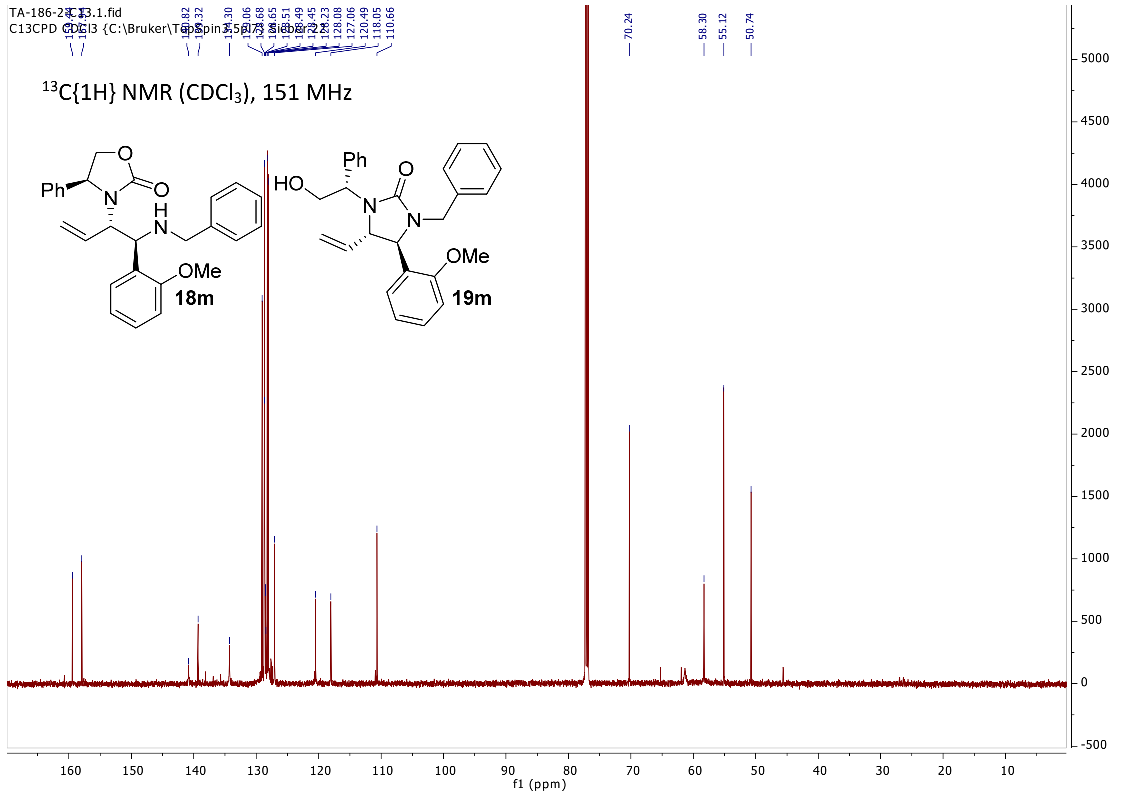

TA-186-24C13.1.fid
13C{1H} NMR (CDCl3), 151 MHz
Ph
O
N
O
H
N
OMe
18m
HO
Ph
O
N
N
OMe
19m
70.24
58.30
55.12
50.74
110.66
118.05
120.49
127.06
128.08
128.23
f1 (ppm)

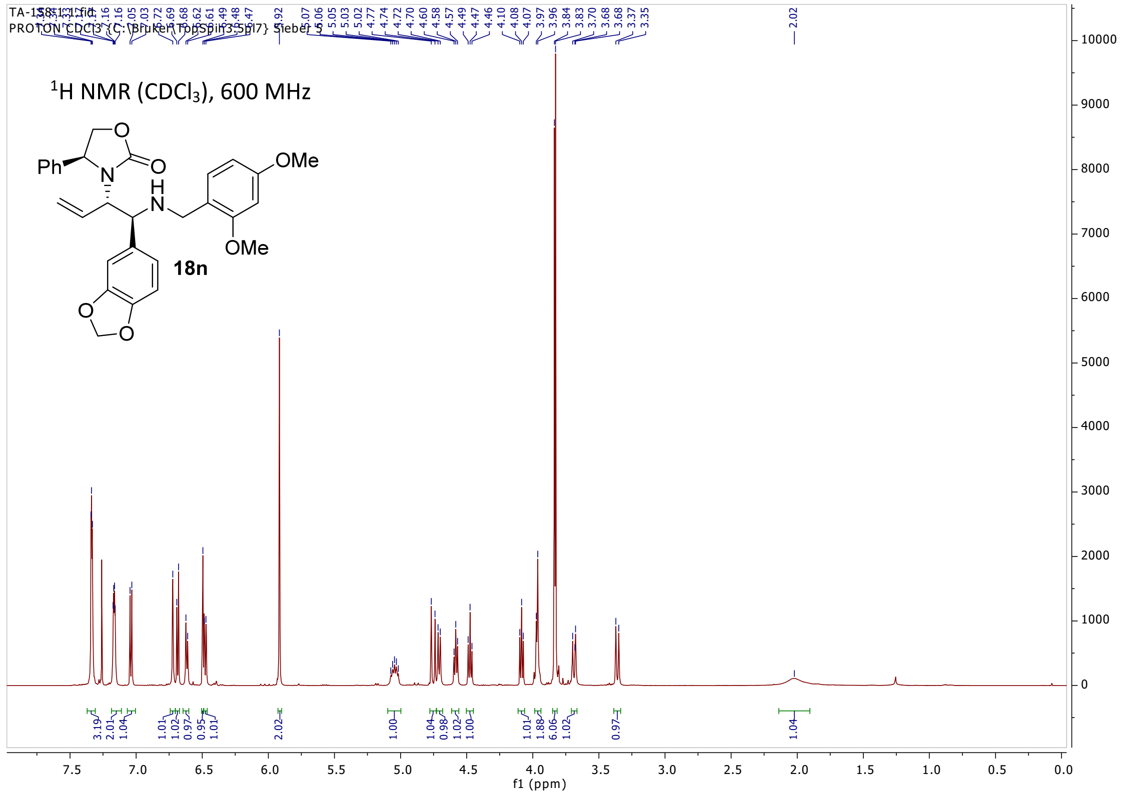

1H NMR (CDCl3), 600 MHz
Ph
O
N
O
H
N
OMe
OMe
18n
O
O
2.02
3.35
3.37
3.68
3.70
3.83
3.84
3.96
3.97
4.07
4.08
4.10
f1 (ppm)

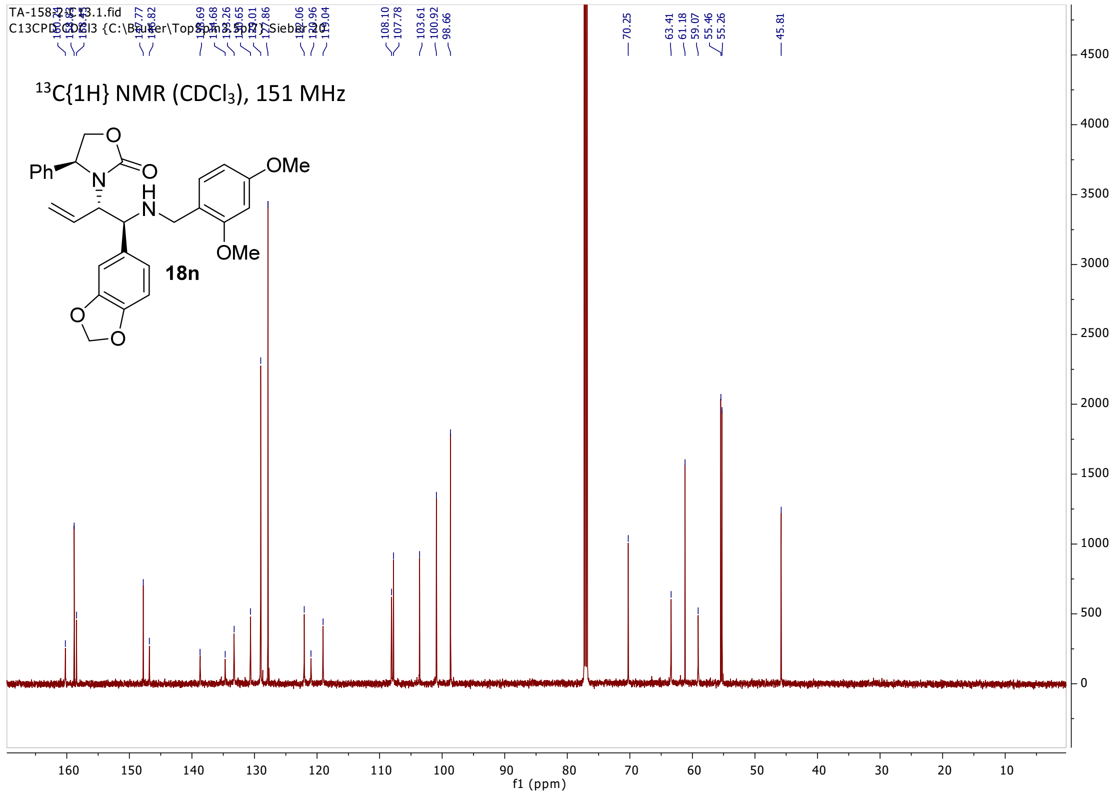
13C{1H} NMR (CDCl3), 151 MHz
OMe
OMe
18n
122.06
120.96
119.04
108.10
107.78
103.61
100.92
98.66
70.25
63.41
61.18
59.07
55.46
55.26
45.81
f1 (ppm)

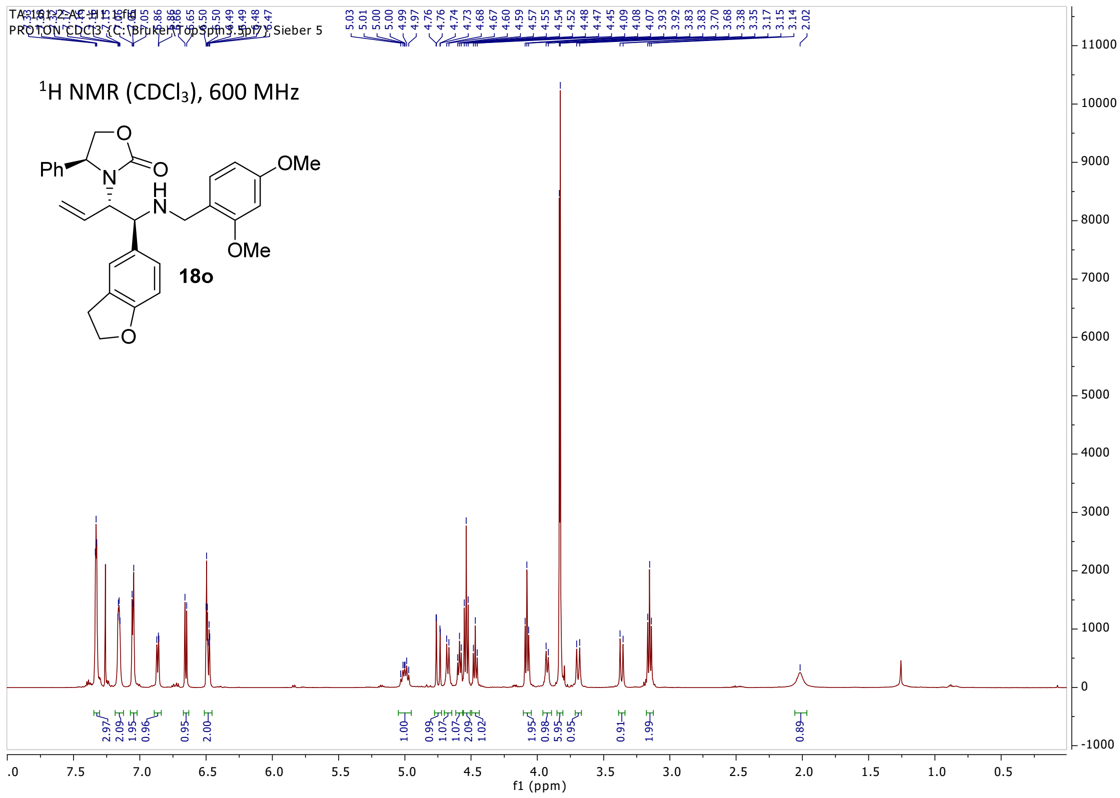
1H NMR (CDCl3), 600 MHz
OMe
OMe
18o
5.03
5.01
5.00
4.99
4.97
4.76
4.74
4.73
4.68
4.67
4.60
4.59
4.57
4.55
4.54
4.52
4.48
4.47
4.45
4.09
4.08
4.07
3.93
3.92
3.83
3.83
3.70
3.68
3.38
3.35
3.17
3.15
3.14
2.02
2.97
2.09
1.95
0.96
0.95
2.00
1.00
0.99
1.07
1.07
2.09
1.02
1.95
0.98
5.95
0.95
0.91
1.99
0.89
f1 (ppm)

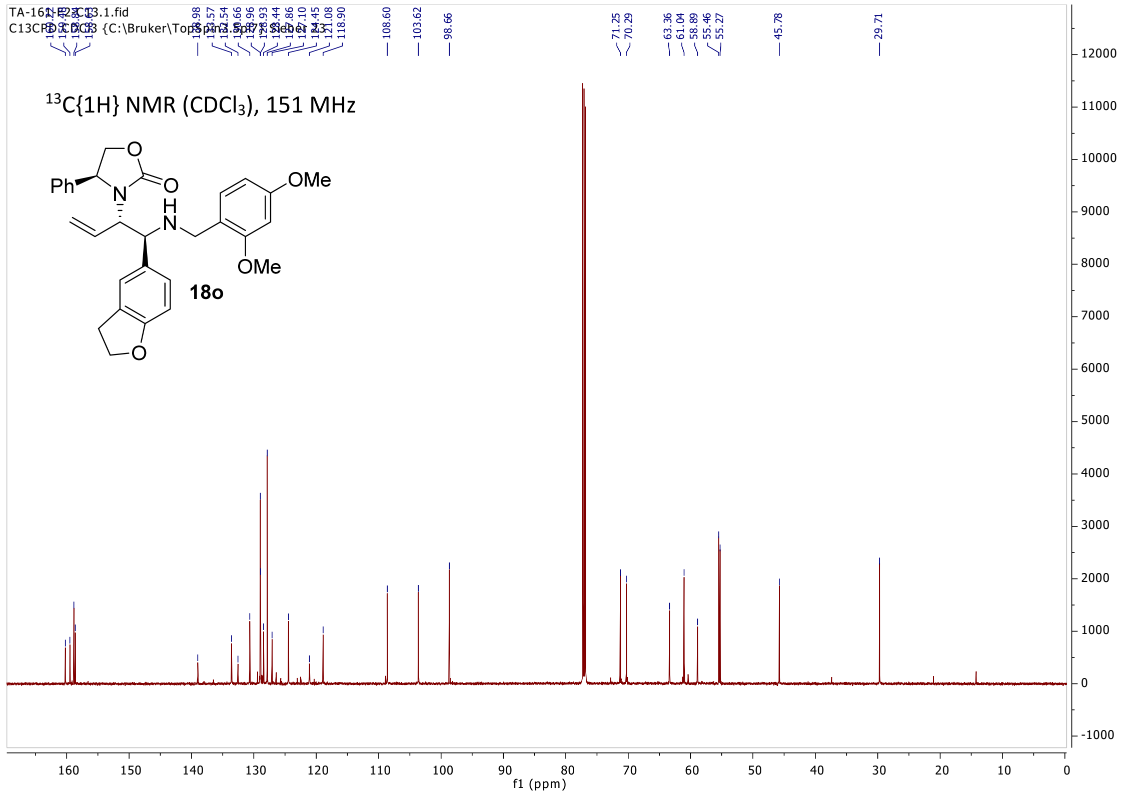
TA-161-F2-C13.1.fid
^{13}C{1H} NMR ($CDCl_3$), 151 MHz
Ph
N
O
O
H
N
OMe
OMe
O
18o
f1 (ppm)

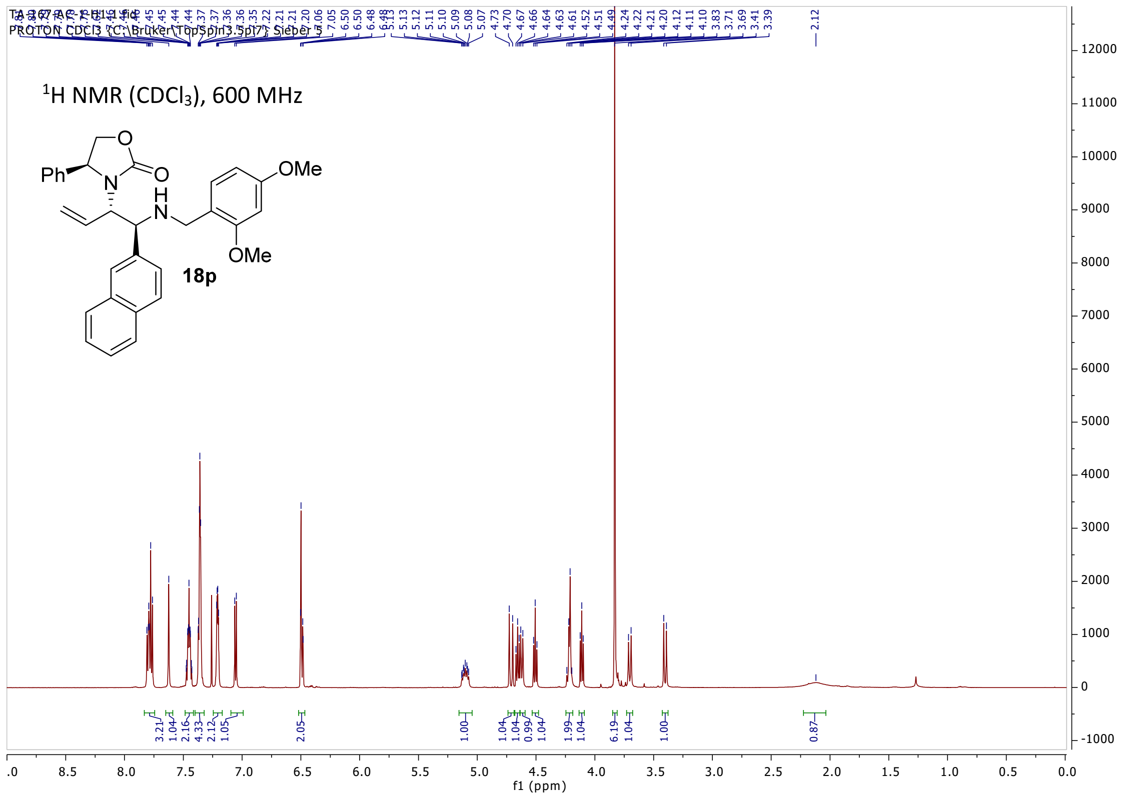
^{1}H NMR ($CDCl_3$), 600 MHz
Ph
N
O
O
H
N
OMe
OMe
18p
f1 (ppm)

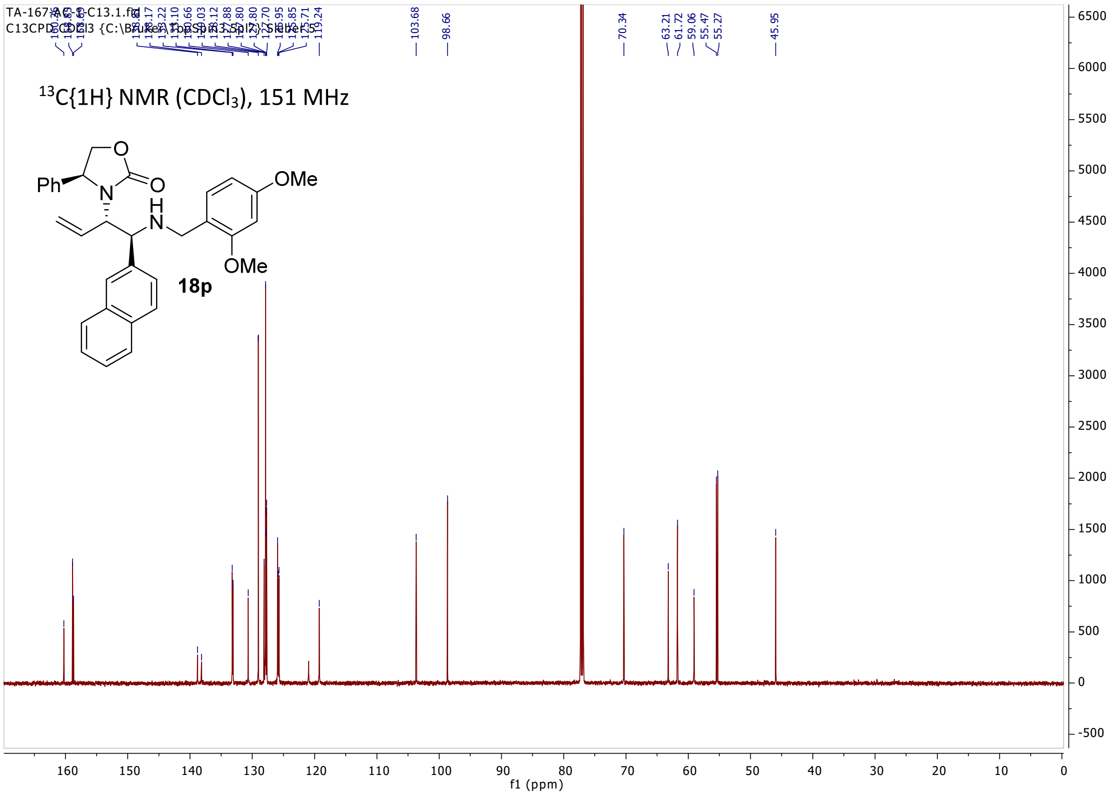
TA-167-AC-1-C13.1.fid
^{13}C{1H} NMR (CDCl$_3$), 151 MHz
Ph
N
O
O
H
N
OMe
OMe
18p
103.68
98.66
70.34
63.21
61.72
59.06
55.47
55.27
45.95
f1 (ppm)

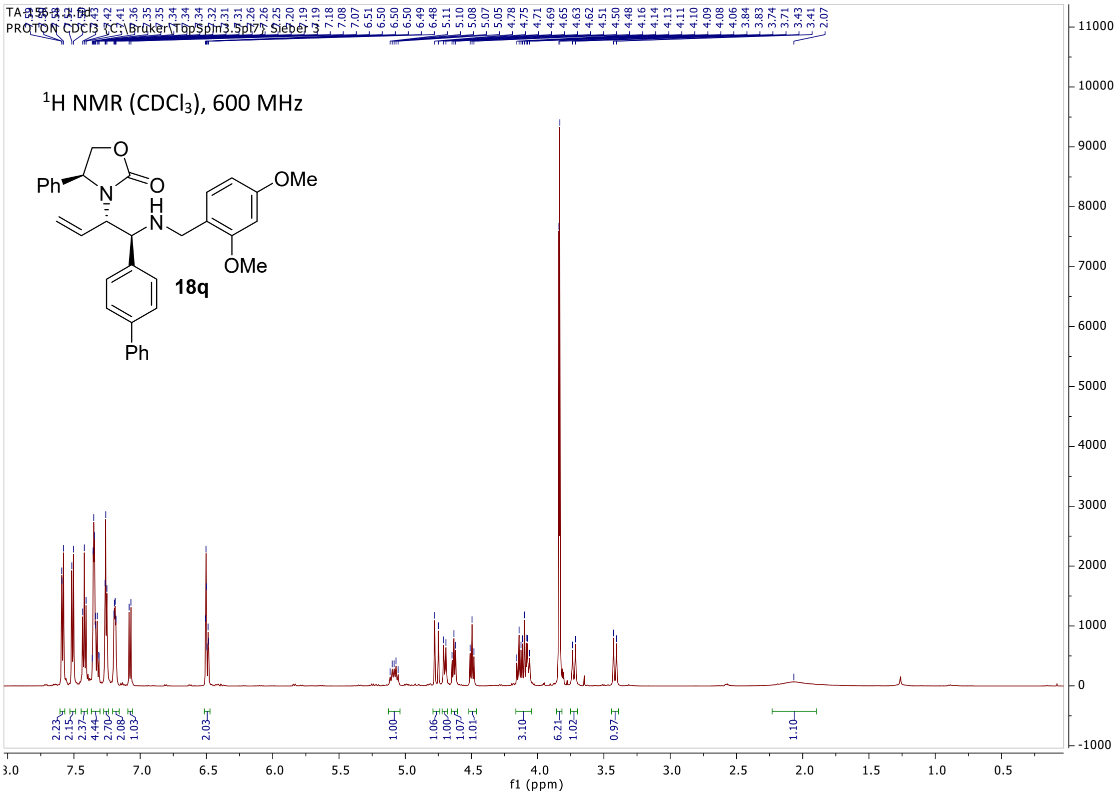
^{1}H NMR (CDCl$_3$), 600 MHz
Ph
N
O
O
H
N
OMe
OMe
18q
Ph
f1 (ppm)

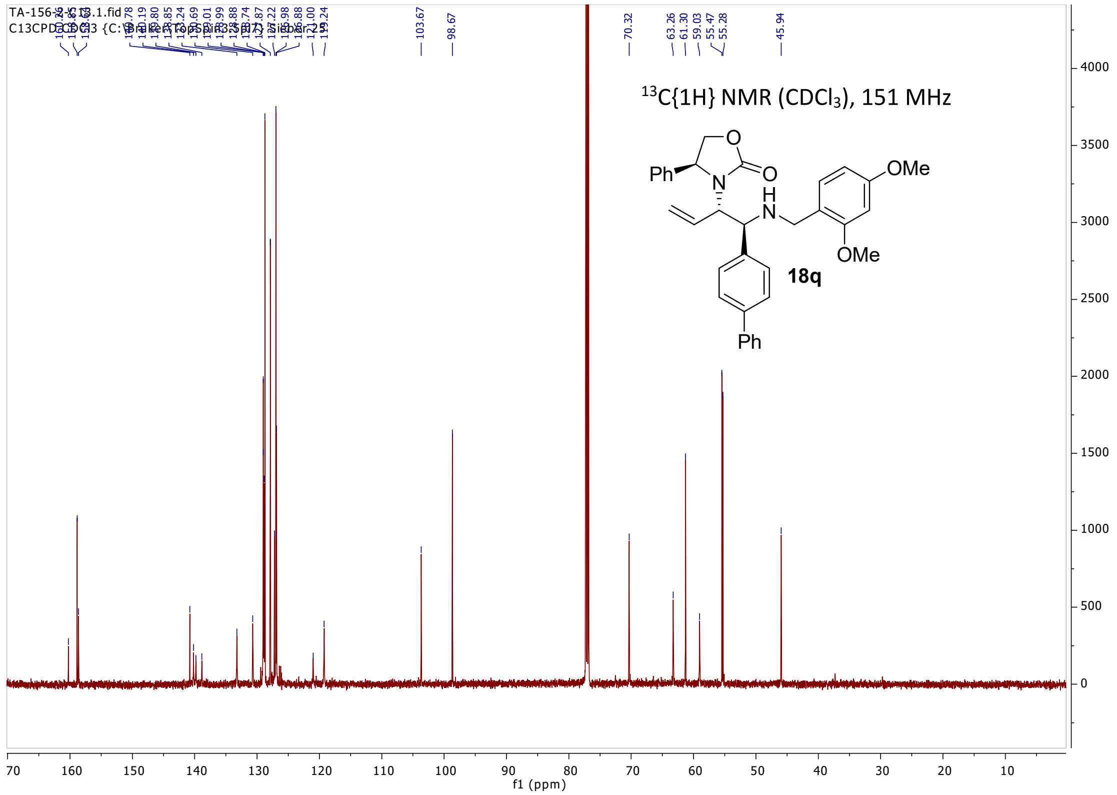
$^{13}C\{1H\}$ NMR ($CDCl_3$), 151 MHz
18q
Ph
OMe
103.67
98.67
70.32
63.26
61.30
59.03
55.47
55.28
45.94
f1 (ppm)

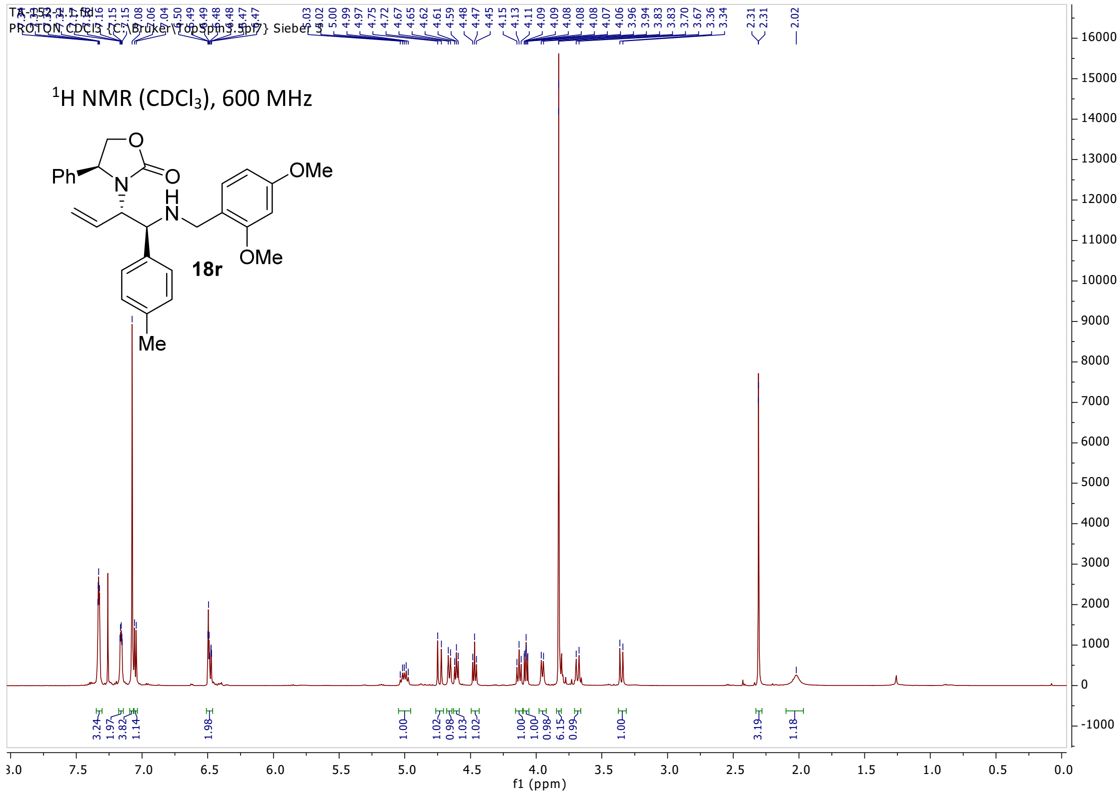
^1H NMR ($CDCl_3$), 600 MHz
18r
Me
OMe
f1 (ppm)

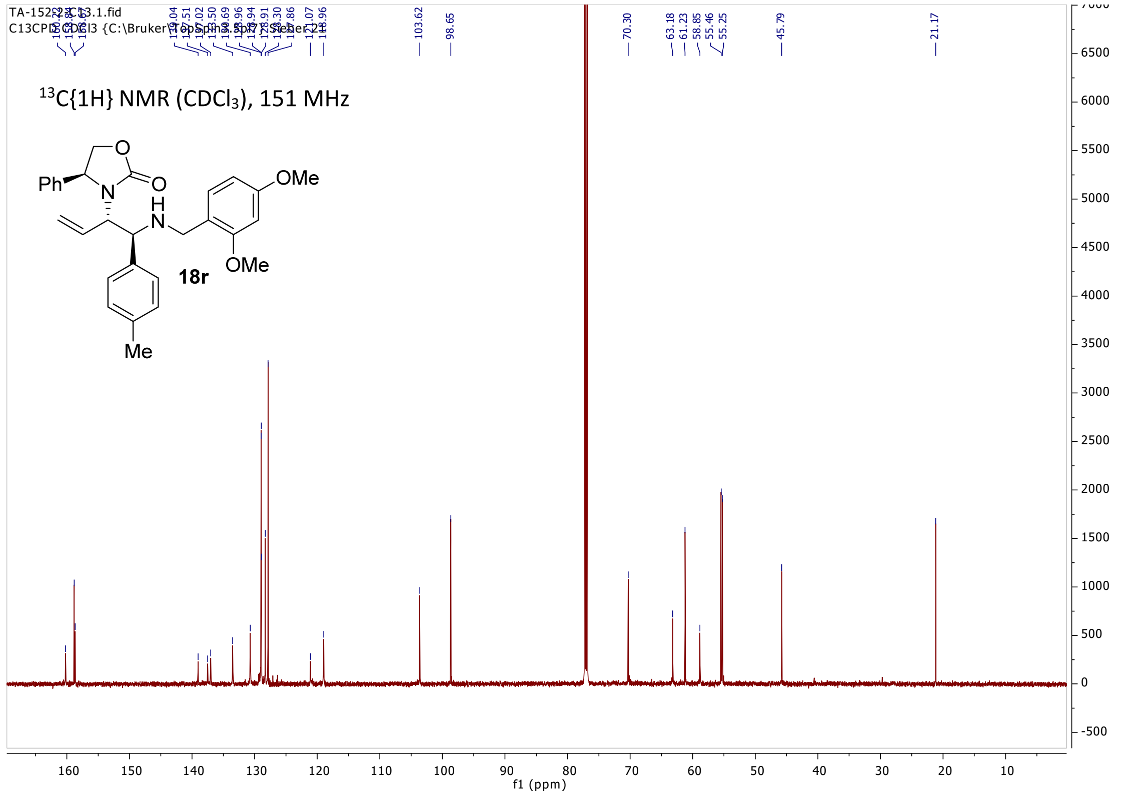

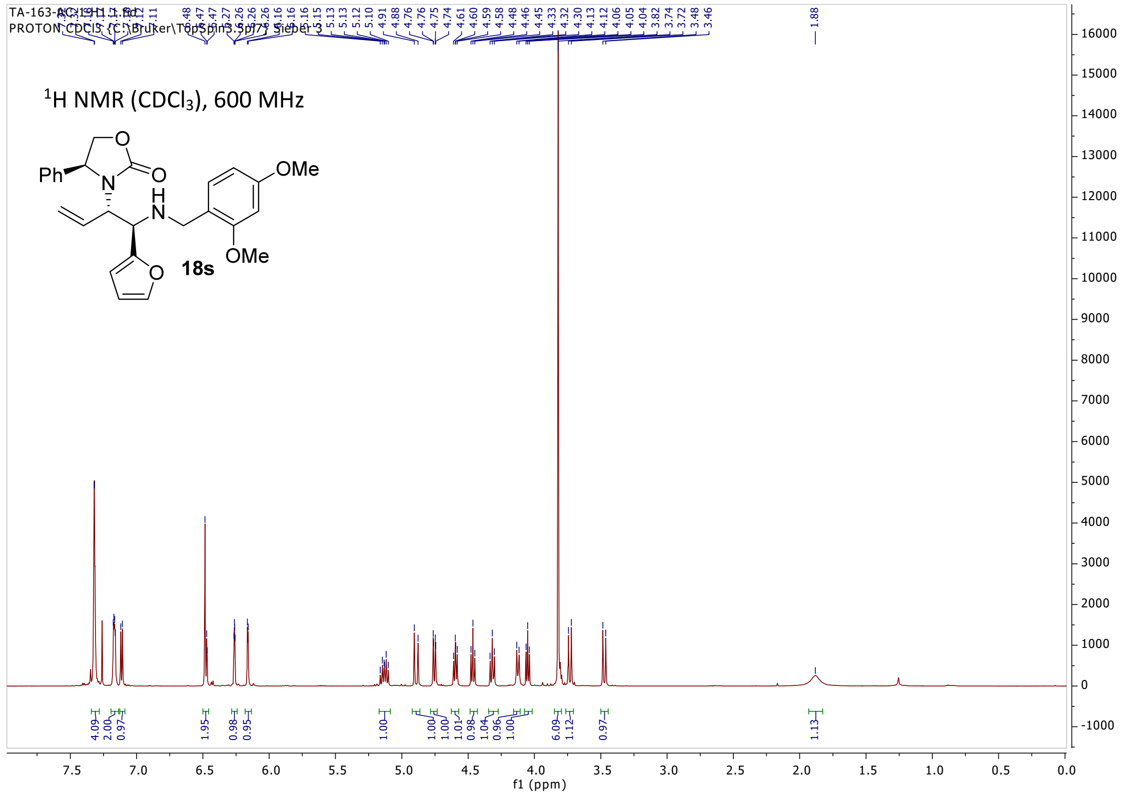

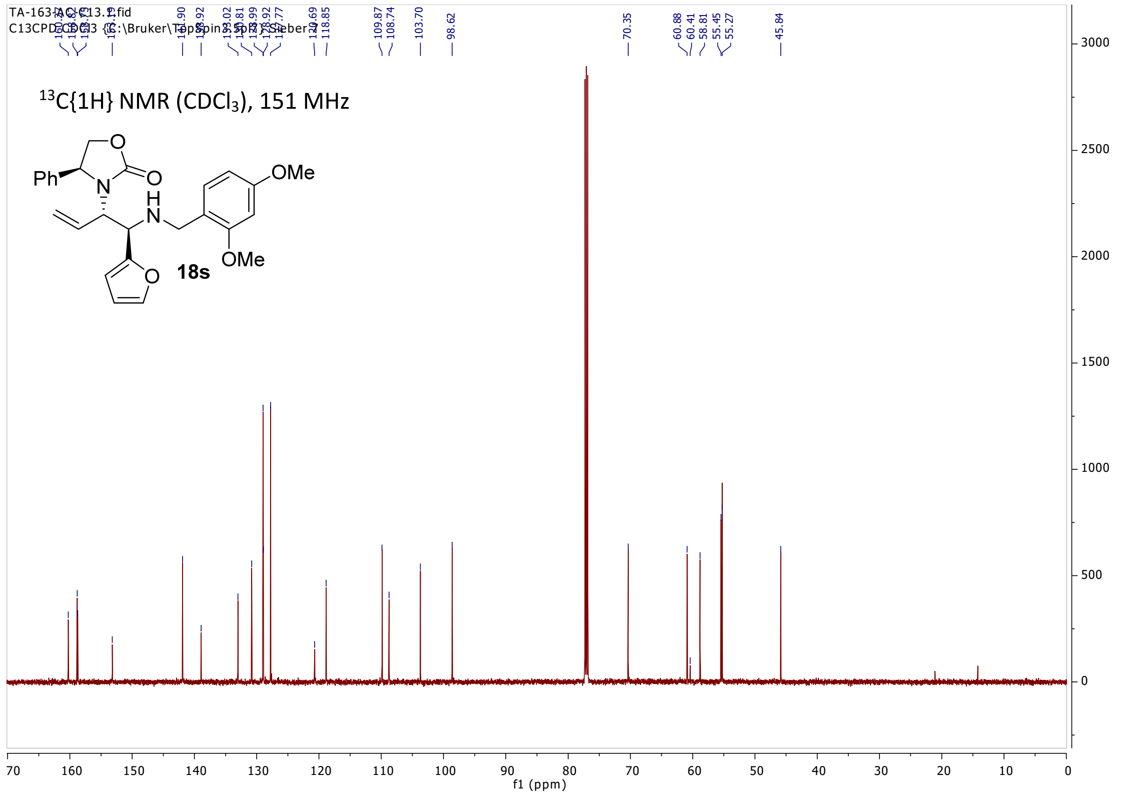
TA-163-AC-C13.1.fid
13C{1H} NMR (CDCl3), 151 MHz
18s
f1 (ppm)

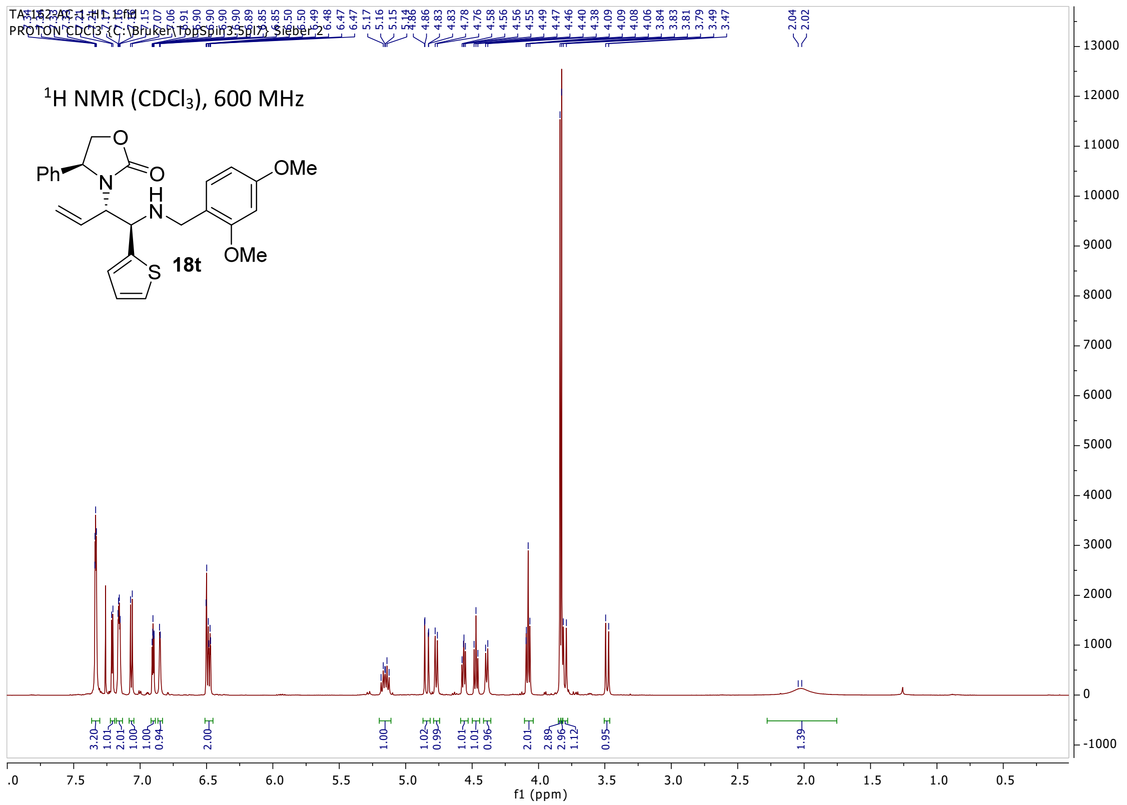
PROTON CDCl3
1H NMR (CDCl3), 600 MHz
18t
f1 (ppm)

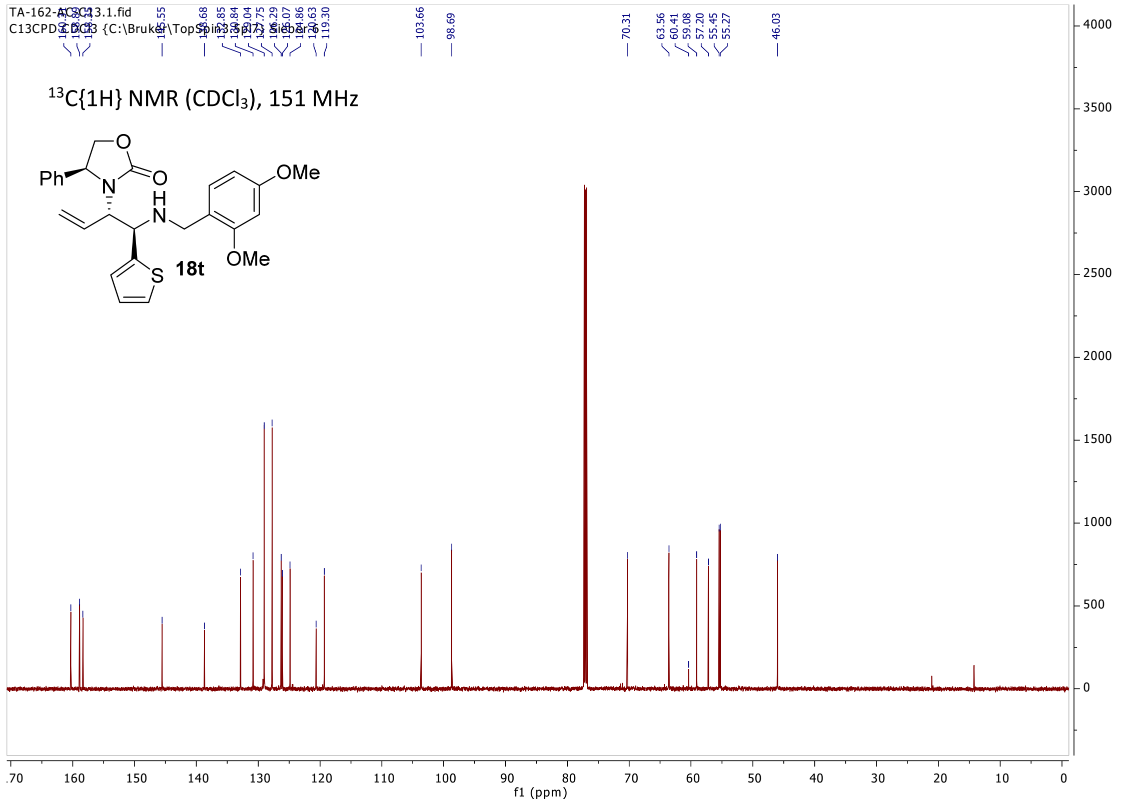
^{13}C{1H} NMR (CDCl$_3$), 151 MHz
18t
f1 (ppm)

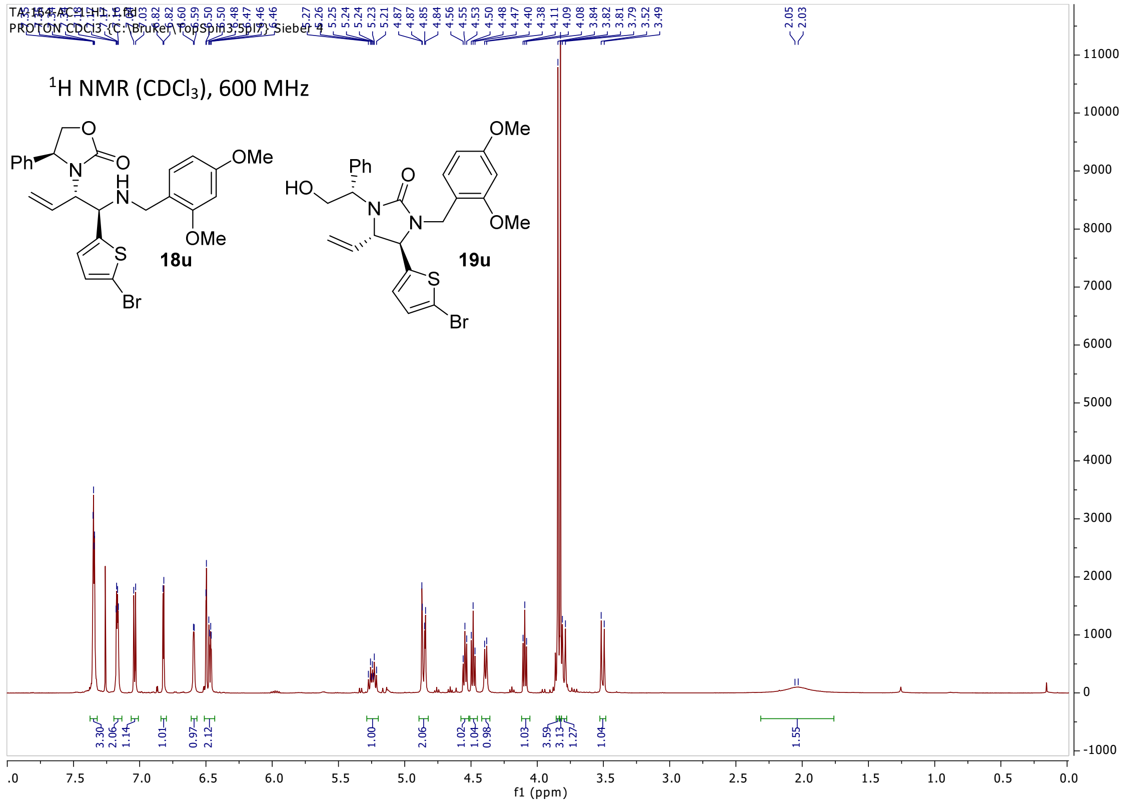
^{1}H NMR (CDCl$_3$), 600 MHz
18u
19u
f1 (ppm)

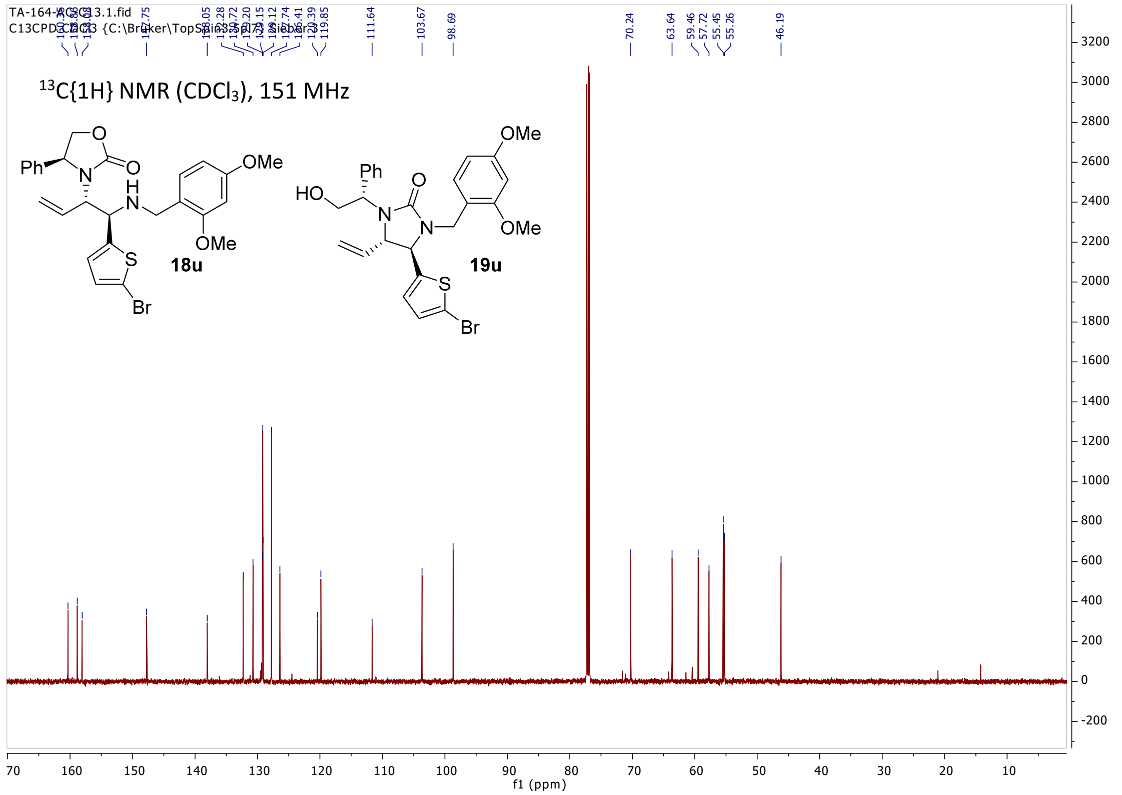
13C{1H} NMR (CDCl3), 151 MHz
18u
19u
OMe
Ph
HO
Br
f1 (ppm)

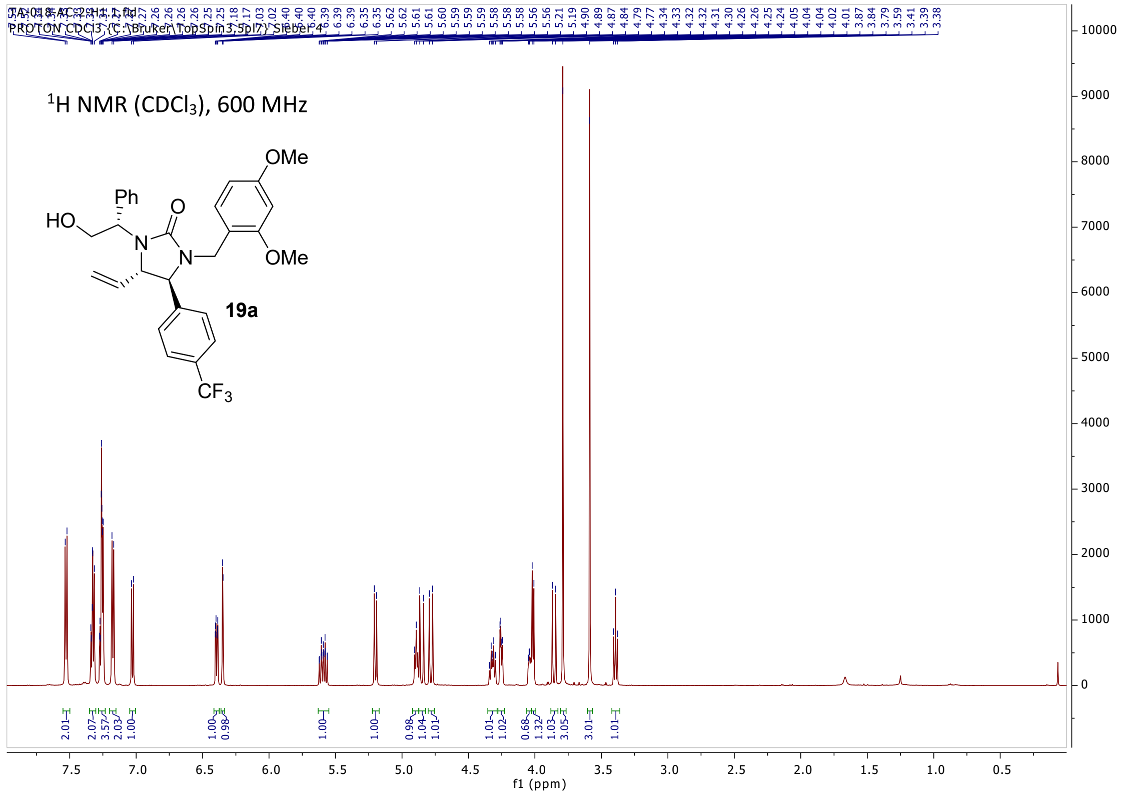
1H NMR (CDCl3), 600 MHz
19a
OMe
Ph
HO
CF3
f1 (ppm)

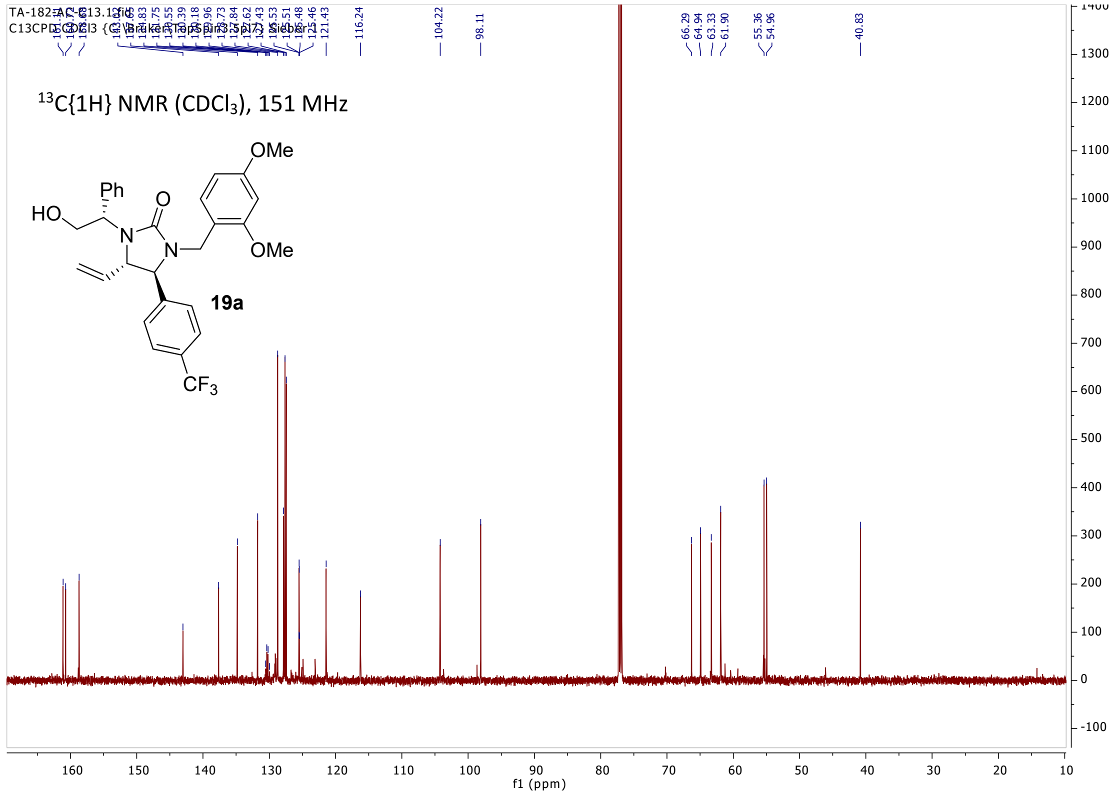
TA-182-AC-C13.1.fid
13C{1H} NMR (CDCl3), 151 MHz
OMe
Ph
HO
O
N
N
OMe
CF3
19a
121.43
116.24
104.22
98.11
66.29
64.94
63.33
61.90
55.36
54.96
40.83
f1 (ppm)

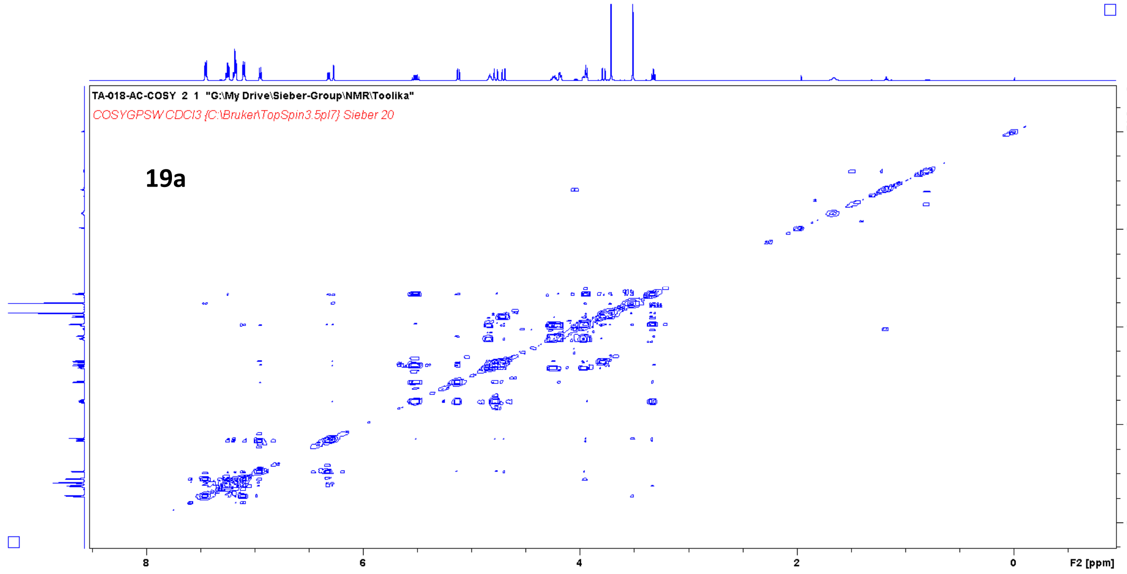
TA-018-AC-COSY 2 1 "G:\My Drive\Sieber-Group\NMR\Toolika"
COSYGPSW CDCl3 {C:\Bruker\TopSpin3.5pl7} Sieber 20
19a
F1 [ppm]
F2 [ppm]

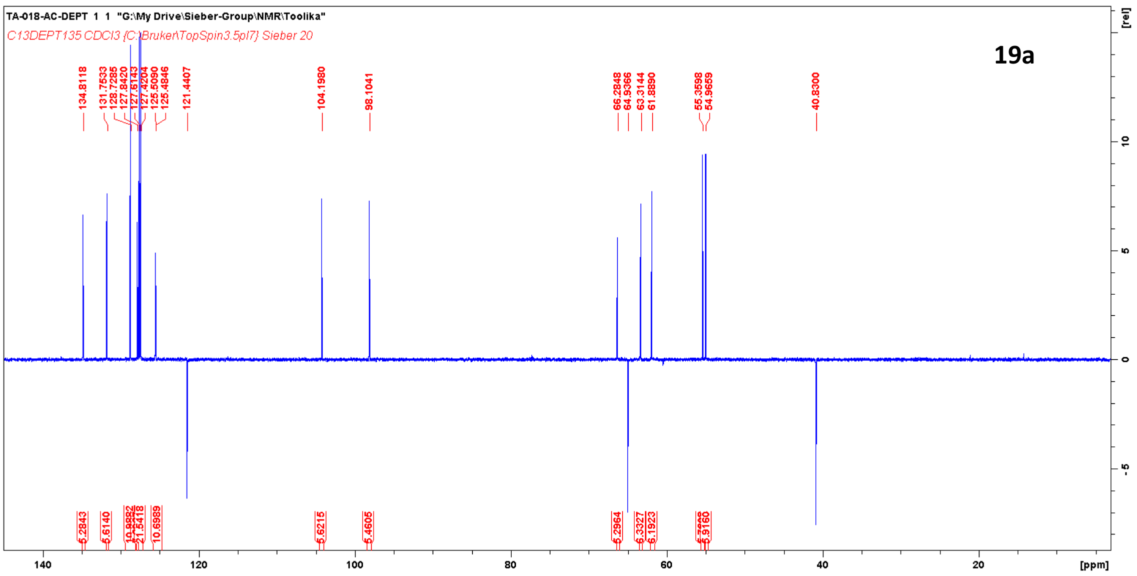
TA-018-AC-DEPT 1 1 "G:\My Drive\Sieber-Group\NMR\Toolika"
C13DEPT135 CDCl3 {C:\Bruker\TopSpin3.5pl7} Sieber 20
19a
134.8118
131.7533
128.7285
127.8420
127.6143
127.4204
125.5090
125.4846
121.4407
104.1980
98.1041
66.2848
64.9366
63.3144
61.8890
55.3598
54.9659
40.8300
[rel]
[ppm]

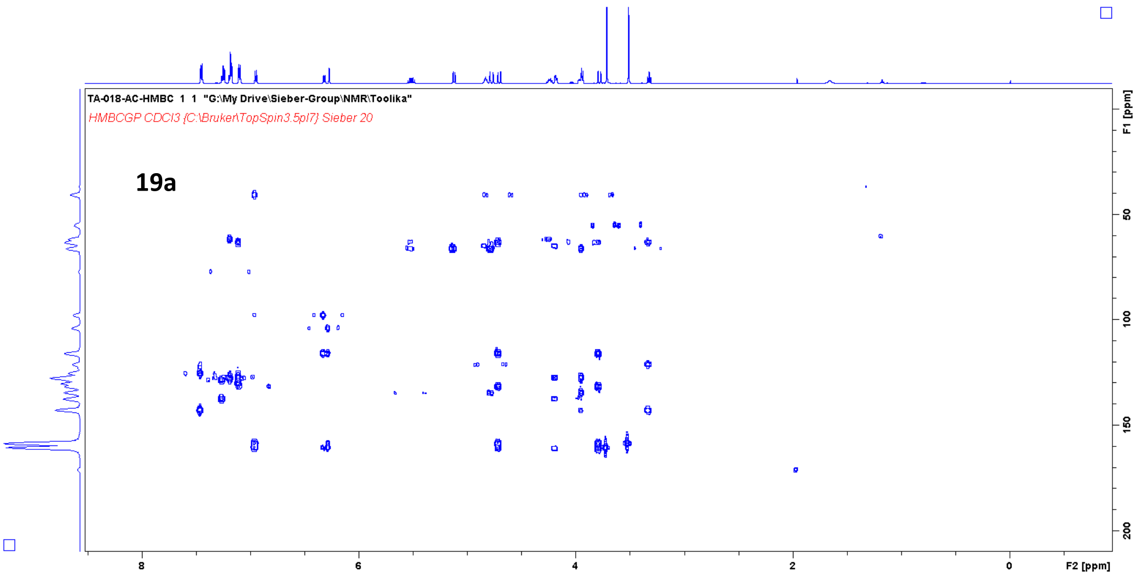
TA-018-AC-HMBC 1 1 "G:\My Drive\Sieber-Group\NMR\Toolika"
HMBCGP CDCl3 {C:\Bruker\TopSpin3.5pl7} Sieber 20
19a
F1 [ppm]
F2 [ppm]

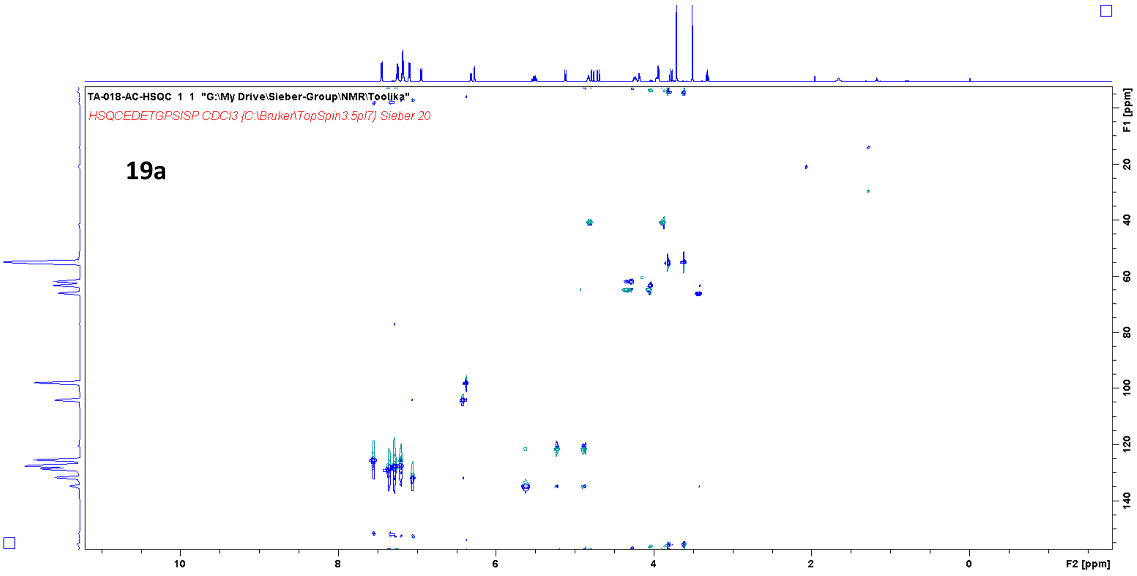
TA-018-AC-HSQC 1 1 "G:\My Drive\Sieber-Group\NMR\Toolika"
HSQCEDETGPSISP CDCl3 {C:\Bruker\TopSpin3.5pl7} Sieber 20
19a
F1 [ppm]
F2 [ppm]

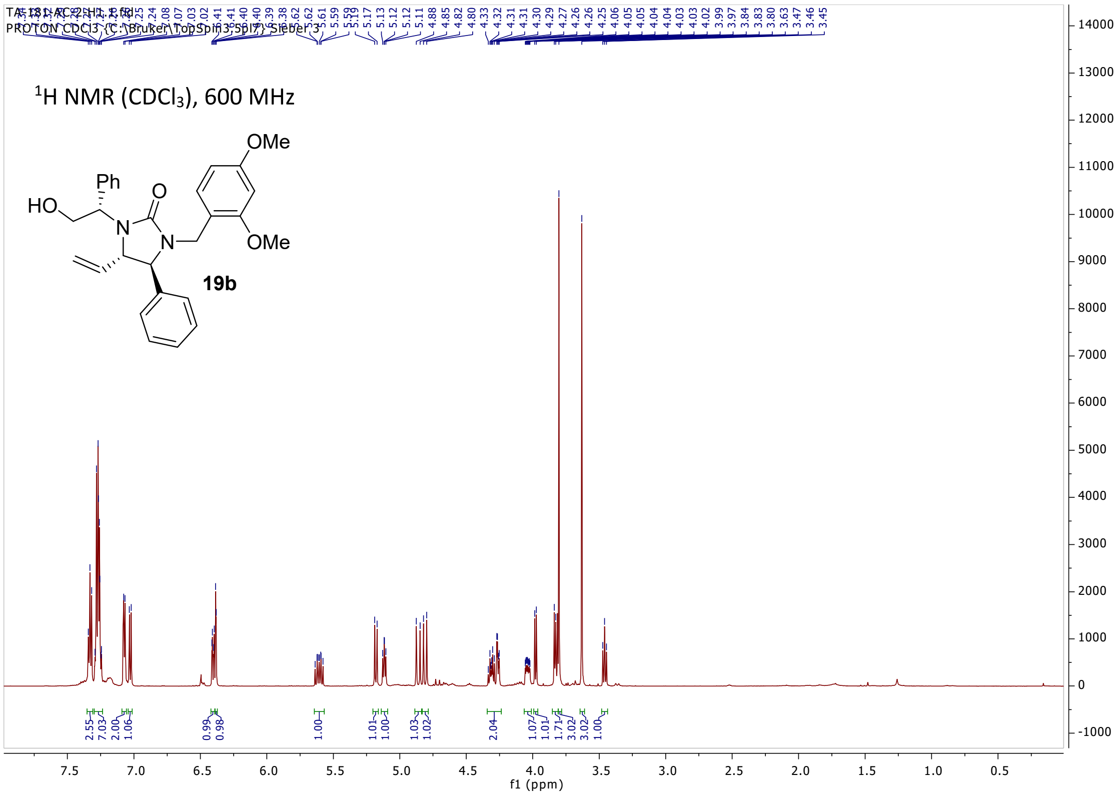
TA-181-AC-2-H1.1.fid
PROTON CDCl3 {C:\Bruker\TopSpin3.5pl7} Sieber 3
¹H NMR (CDCl₃), 600 MHz
OMe
Ph
O
HO
N
N
OMe
19b
f1 (ppm)

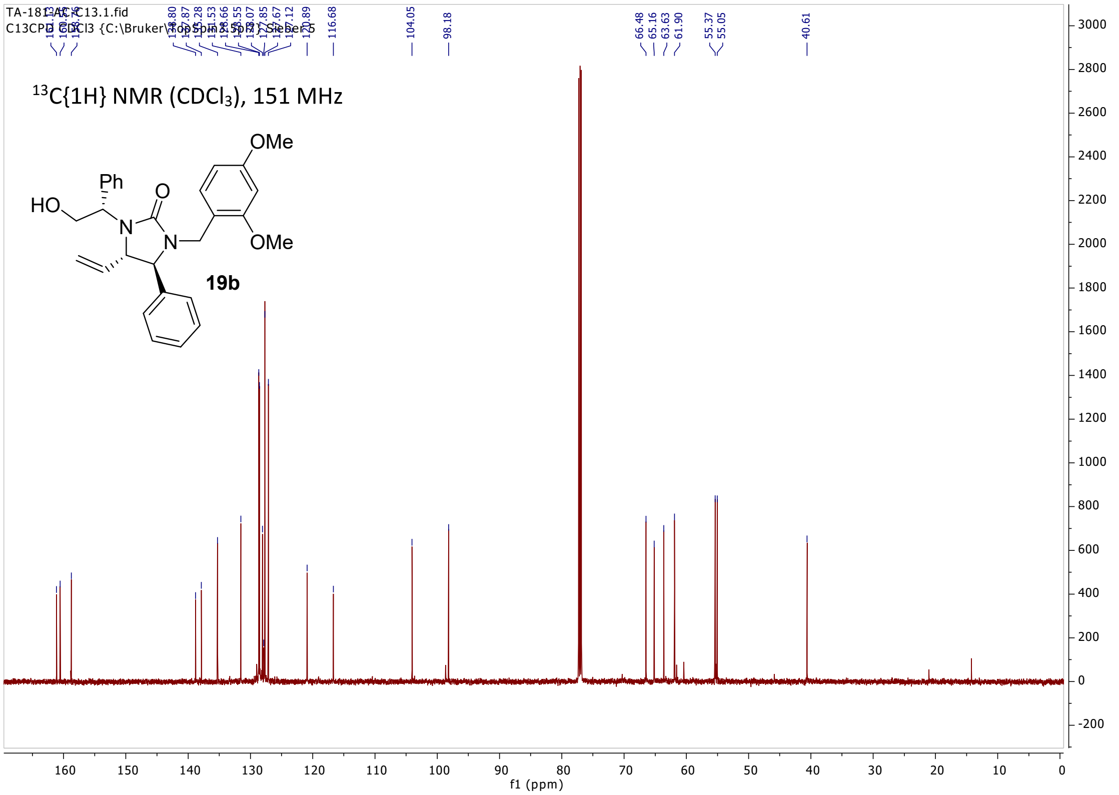
TA-181-AC-C13.1.fid
C13CPD CDCl3 {C:\Bruker\TopSpin3.5pl7} Sieber 5
¹³C{1H} NMR (CDCl₃), 151 MHz
OMe
Ph
O
HO
N
N
OMe
19b
120.89
116.68
104.05
98.18
66.48
65.16
63.63
61.90
55.37
55.05
40.61
f1 (ppm)

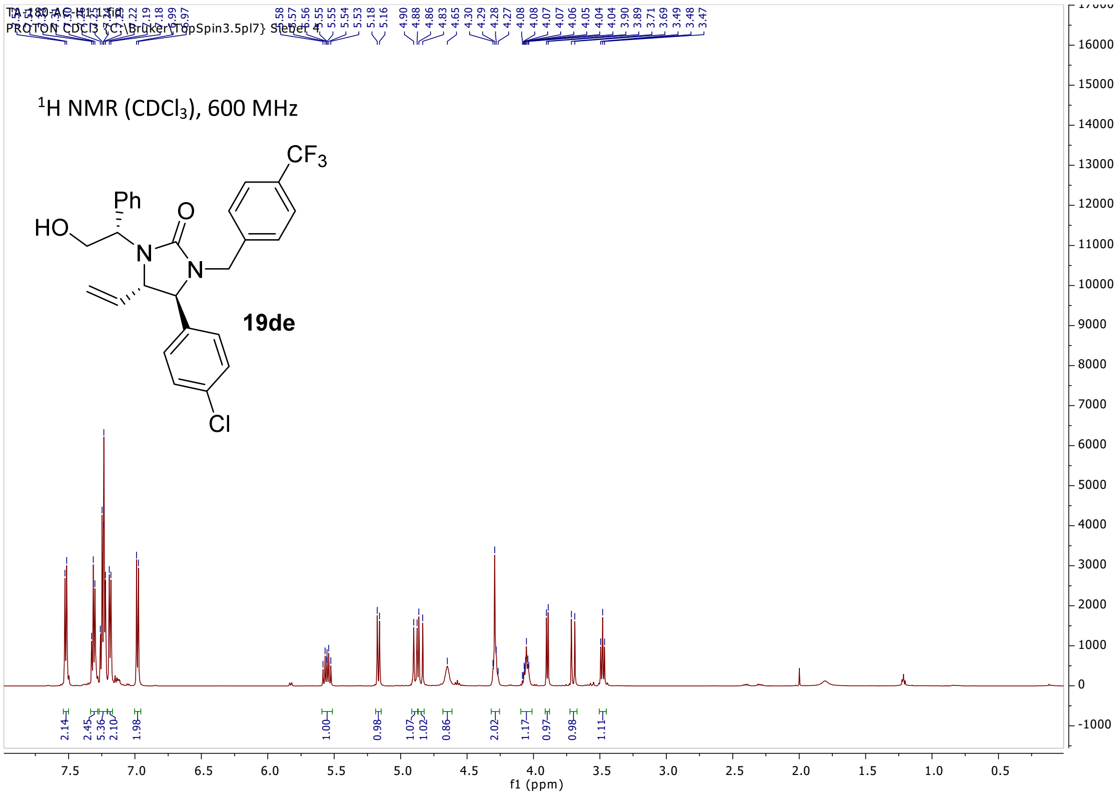
TA-180-AC-H1.1.fid
PROTON CDCl3 {C:\Bruker\TopSpin3.5pl7} Sieber 4
1H NMR (CDCl3), 600 MHz
CF3
Ph
O
HO
N
N
19de
Cl
f1 (ppm)

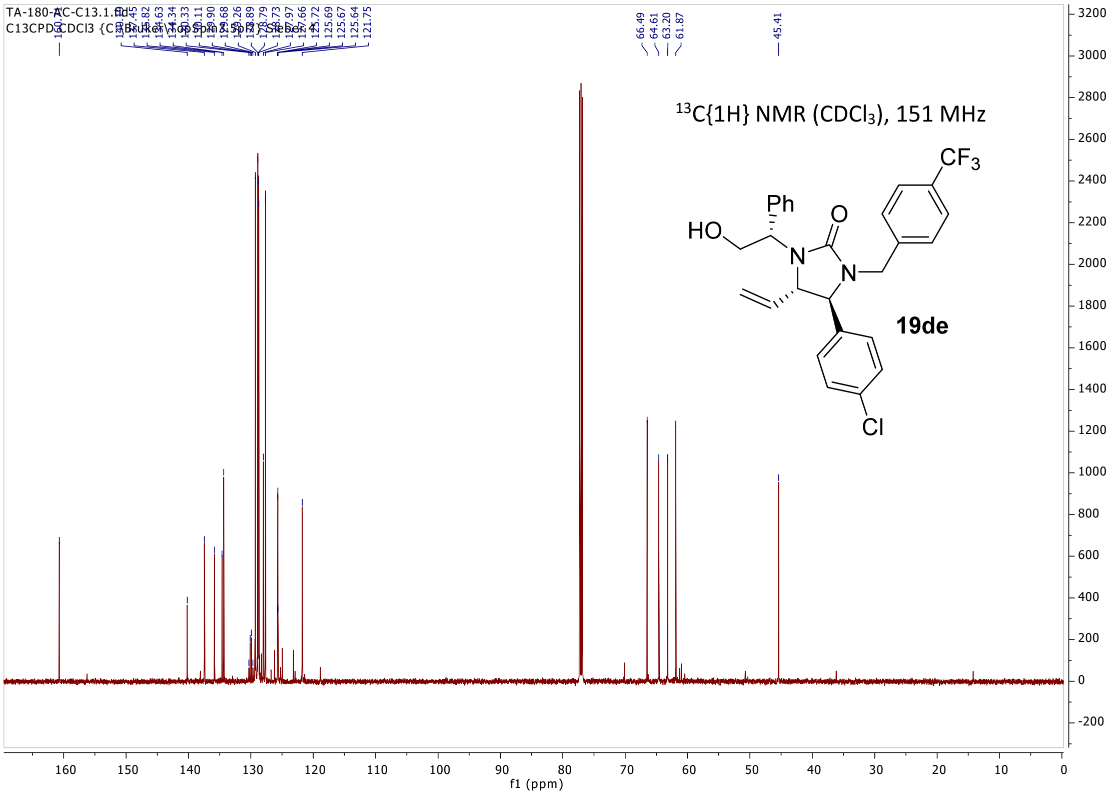
TA-180-AC-C13.1.fid
C13CPD CDCl3 {C:\Bruker\TopSpin3.5pl7} Sieber 4
66.49
64.61
63.20
61.87
45.41
13C{1H} NMR (CDCl3), 151 MHz
CF3
Ph
O
HO
N
N
19de
Cl
f1 (ppm)

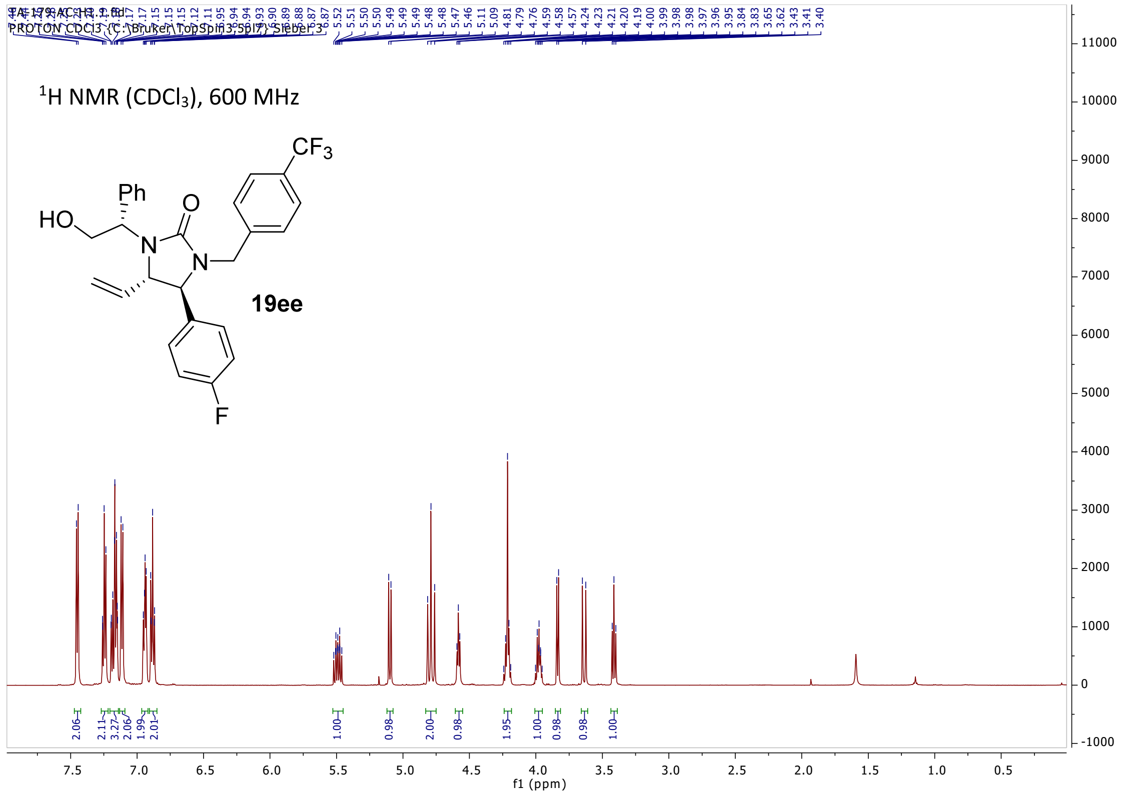
PROTON CDCl3 {C:\Bruker\TopSpin3.5pl7} Sieber 3
1H NMR (CDCl3), 600 MHz
CF3
Ph
HO
O
N
N
19ee
F
5.52
5.51
5.50
5.49
5.49
5.49
5.48
5.48
5.47
5.46
5.11
5.09
4.81
4.79
4.76
4.59
4.58
4.57
4.24
4.23
4.21
4.20
4.19
4.00
3.99
3.98
3.98
3.97
3.96
3.95
3.84
3.83
3.65
3.62
3.43
3.41
3.40
2.06
2.11
3.27
2.06
1.99
2.01
1.00
0.98
2.00
0.98
1.95
1.00
0.98
0.98
1.00
f1 (ppm)

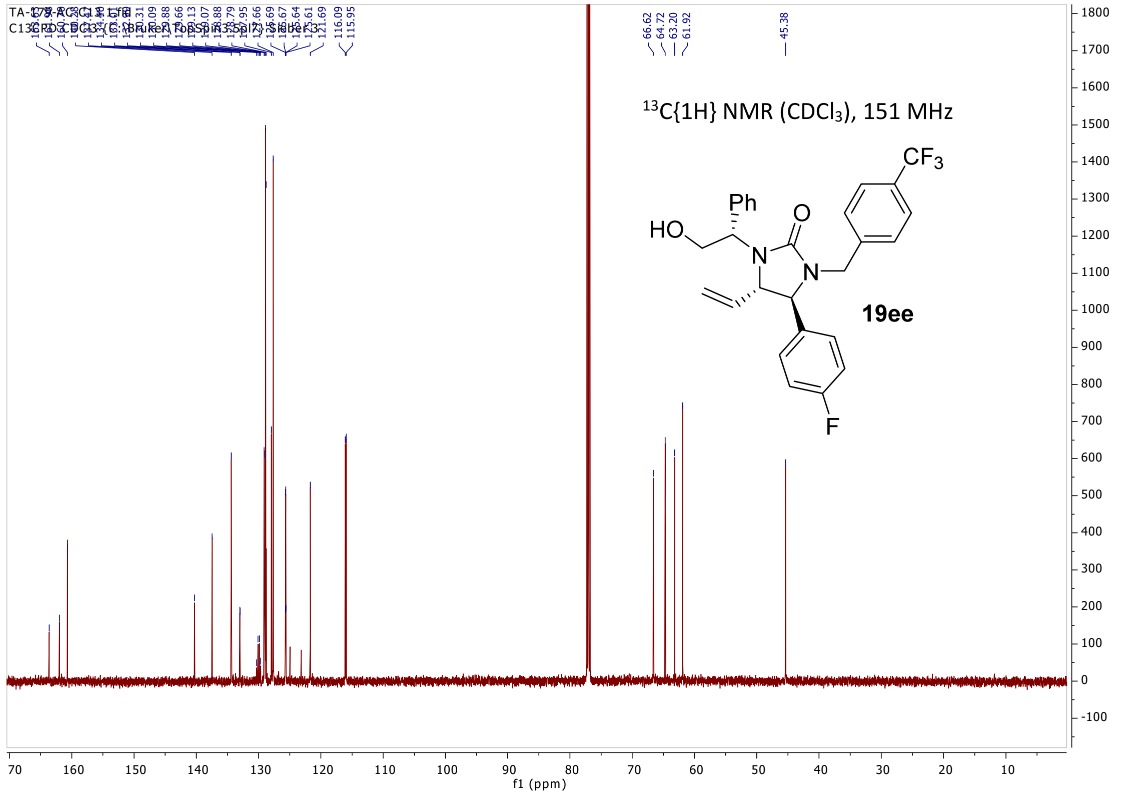
C13CPD CDCl3 {C:\Bruker\TopSpin3.5pl7} Sieber 3
116.09
115.95
66.62
64.72
63.20
61.92
45.38
13C{1H} NMR (CDCl3), 151 MHz
CF3
Ph
HO
O
N
N
19ee
F
f1 (ppm)

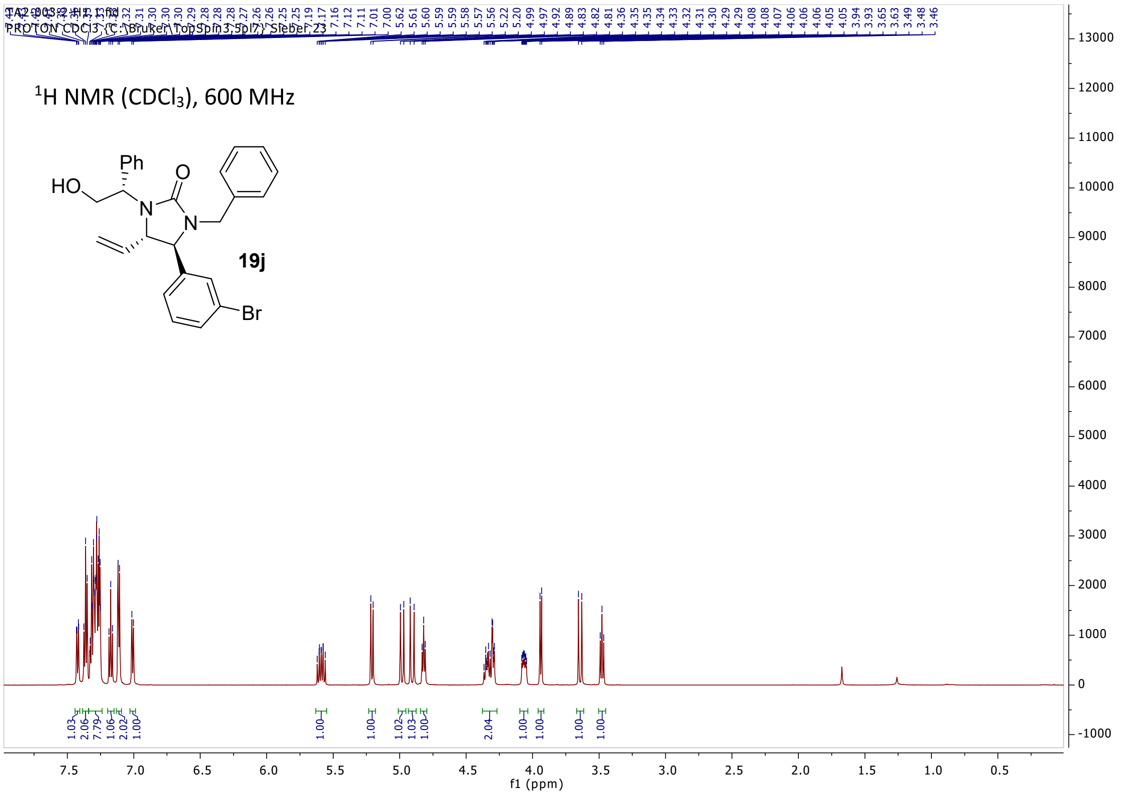

^{1}H NMR ($CDCl_3$), 600 MHz
HO
Ph
O
N
N
19j
Br
f1 (ppm)

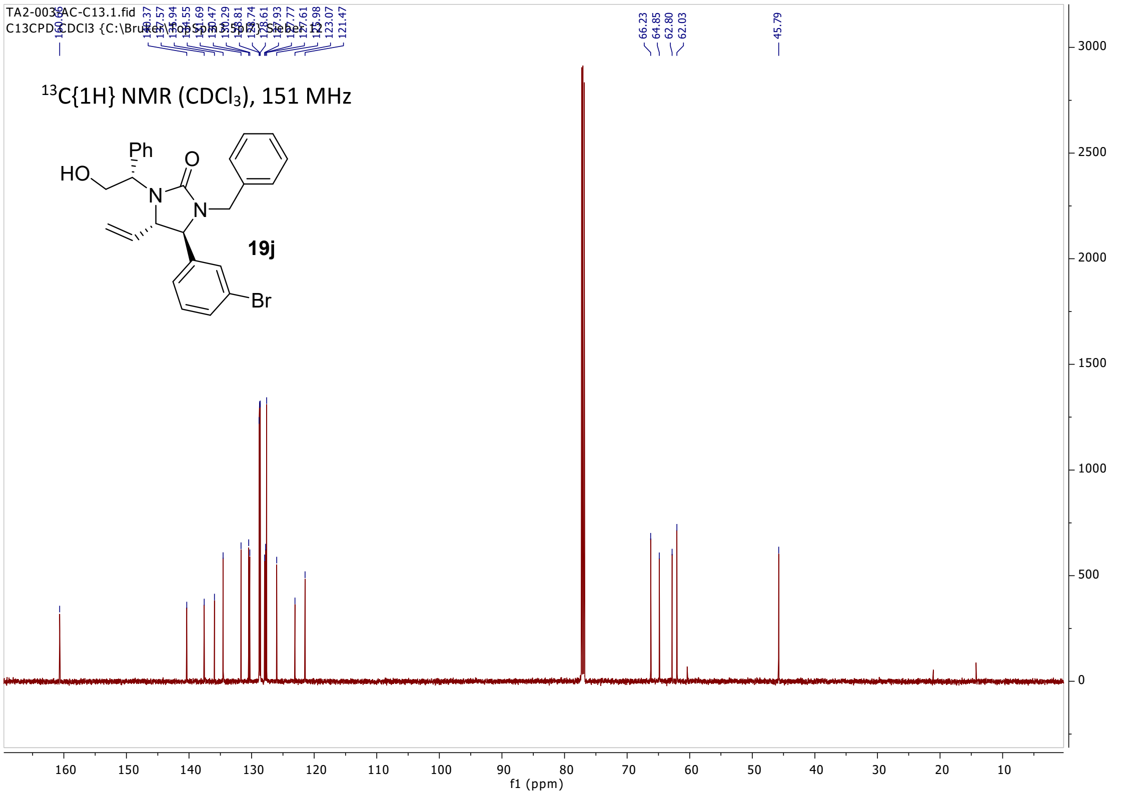

^{13}C{1H} NMR ($CDCl_3$), 151 MHz
HO
Ph
O
N
N
19j
Br
66.23
64.85
62.80
62.03
45.79
f1 (ppm)

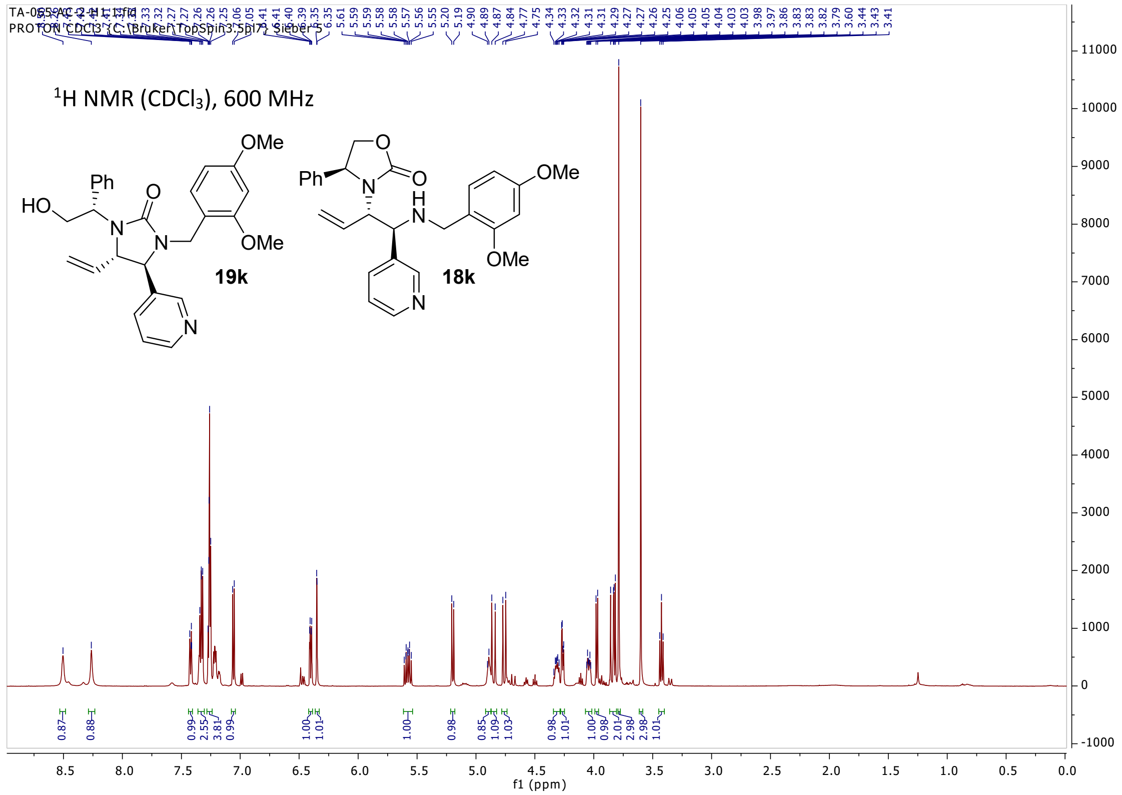
^{1}H NMR (CDCl$_3$), 600 MHz
19k
18k
f1 (ppm)

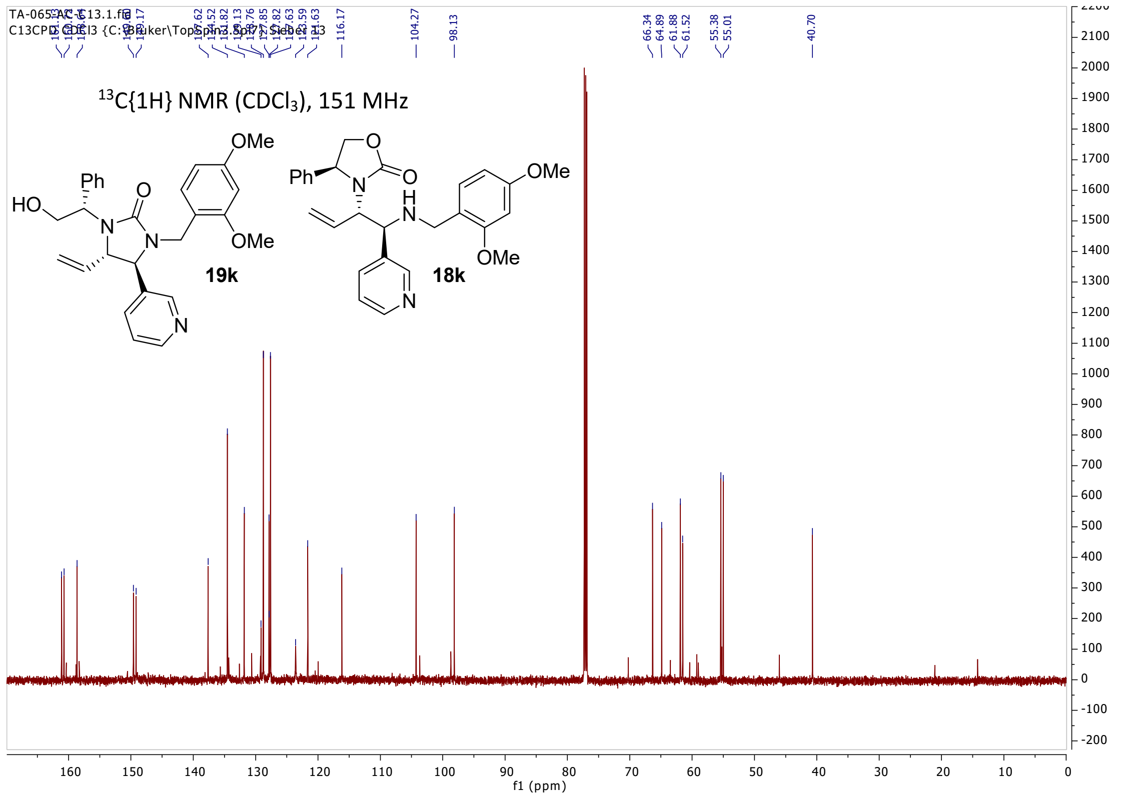
^{13}C{1H} NMR (CDCl$_3$), 151 MHz
19k
18k
f1 (ppm)

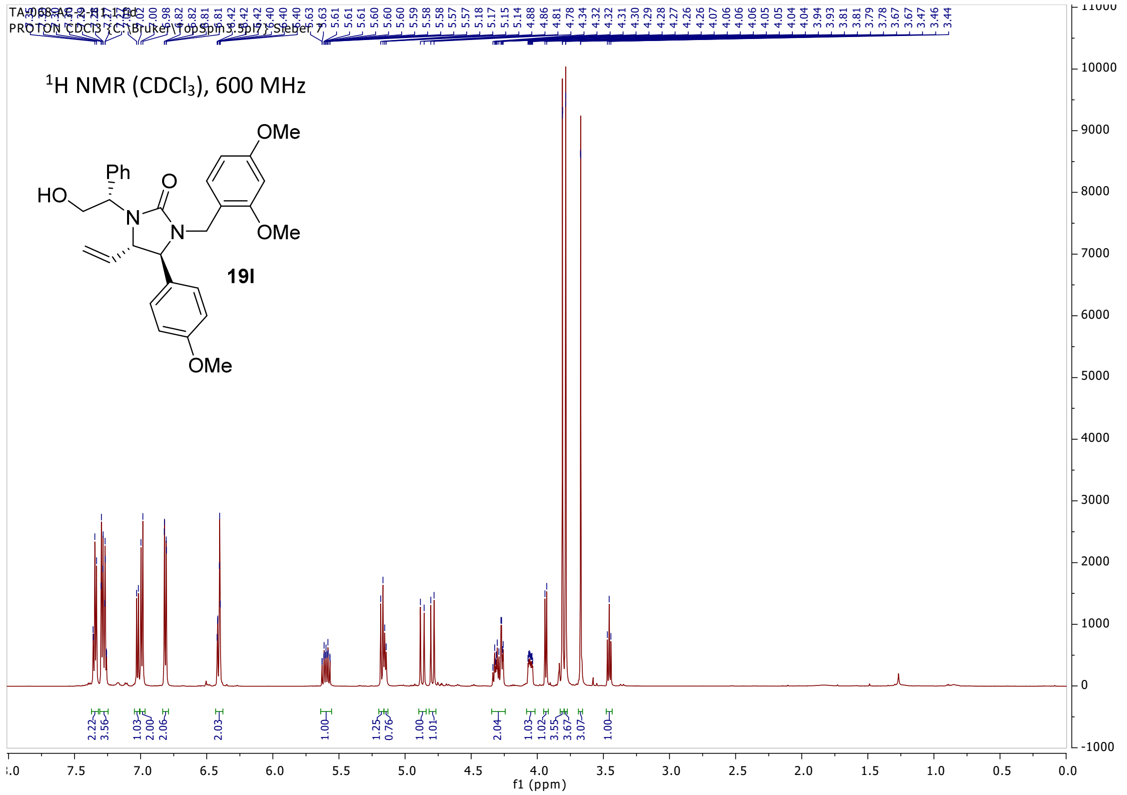
1H NMR (CDCl3), 600 MHz
OMe
Ph
HO
O
N
N
OMe
19l
OMe
f1 (ppm)

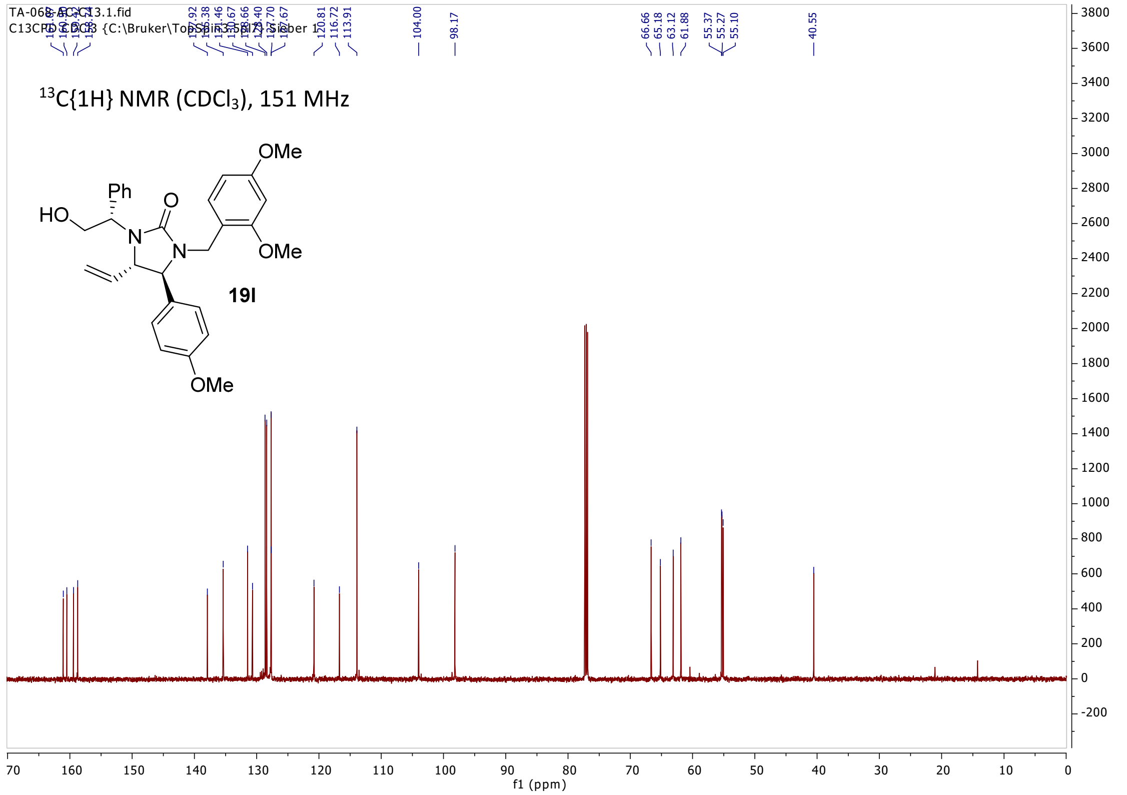
13C{1H} NMR (CDCl3), 151 MHz
OMe
Ph
HO
O
N
N
OMe
19l
OMe
f1 (ppm)

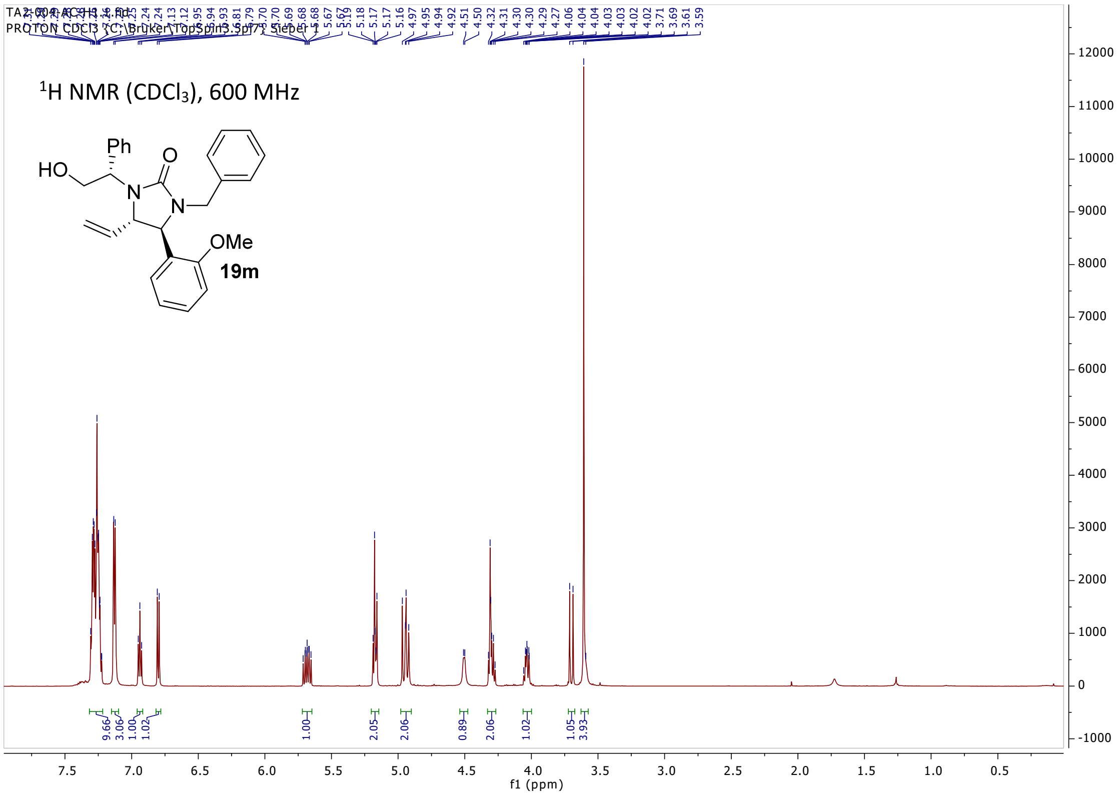
TA2-004-AC-1H-1.fid
PROTON CDCl3 {C:\Bruker\TopSpin3.5pl7} Siebe
^{1}H NMR (CDCl$_3$), 600 MHz
HO
Ph
O
N
N
OMe
19m
f1 (ppm)

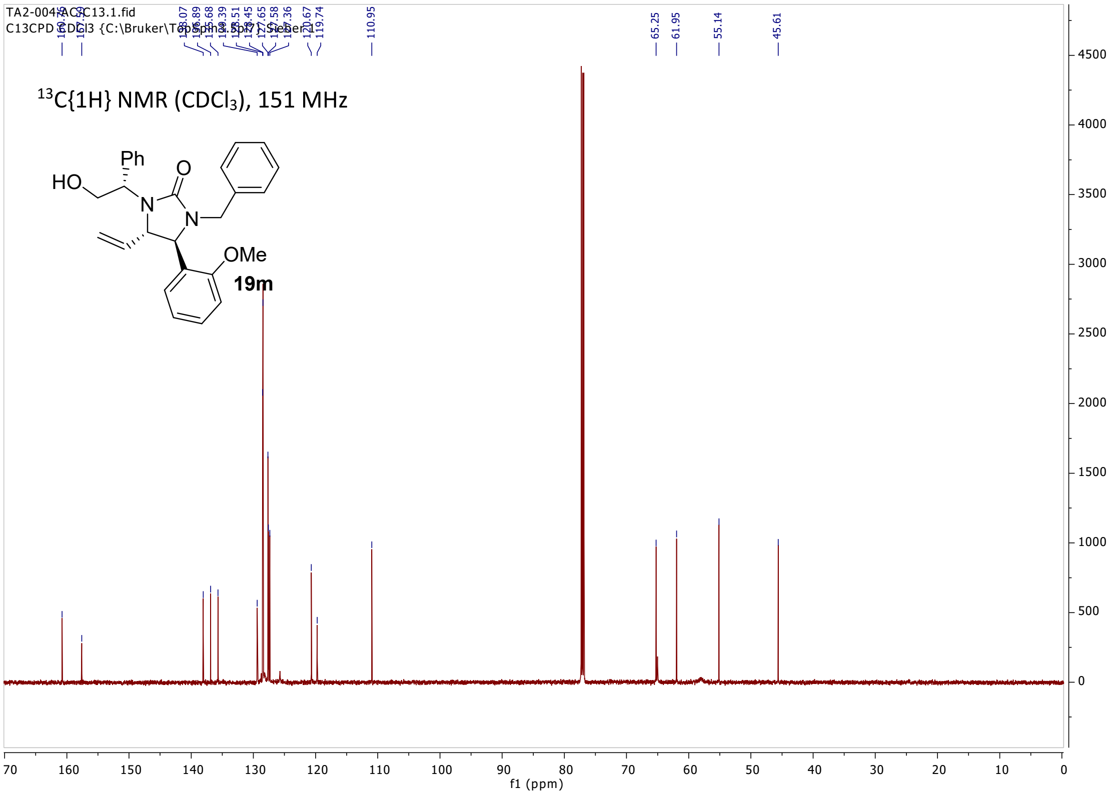
TA2-004-AC-C13.1.fid
C13CPD CDCl3 {C:\Bruker\TopSpin3.5pl7} Siebe
^{13}C{1H} NMR (CDCl$_3$), 151 MHz
HO
Ph
O
N
N
OMe
19m
110.95
65.25
61.95
55.14
45.61
f1 (ppm)

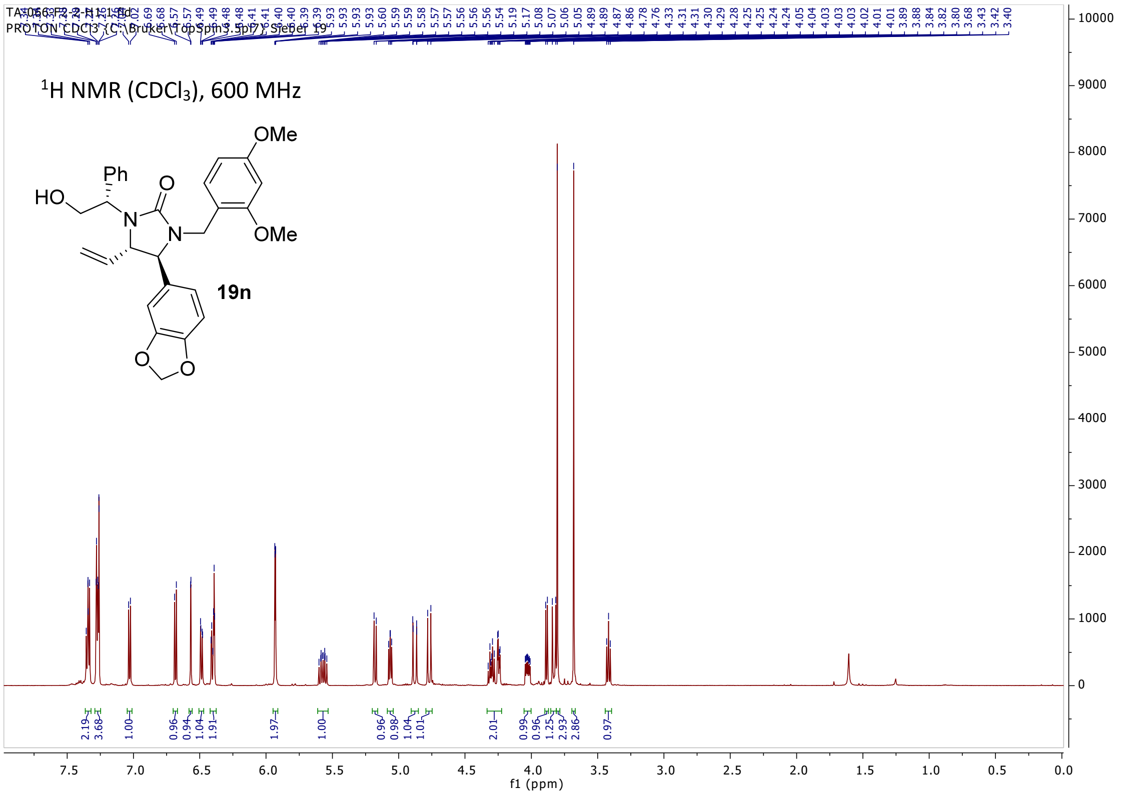
TA-066-F2-2-H1.1.fid
PROTON CDCl3 {C:\Bruker\TopSpin3.5pl7} Sieber 19
1H NMR (CDCl3), 600 MHz
OMe
Ph
HO
O
N
N
OMe
19n
O
O
f1 (ppm)

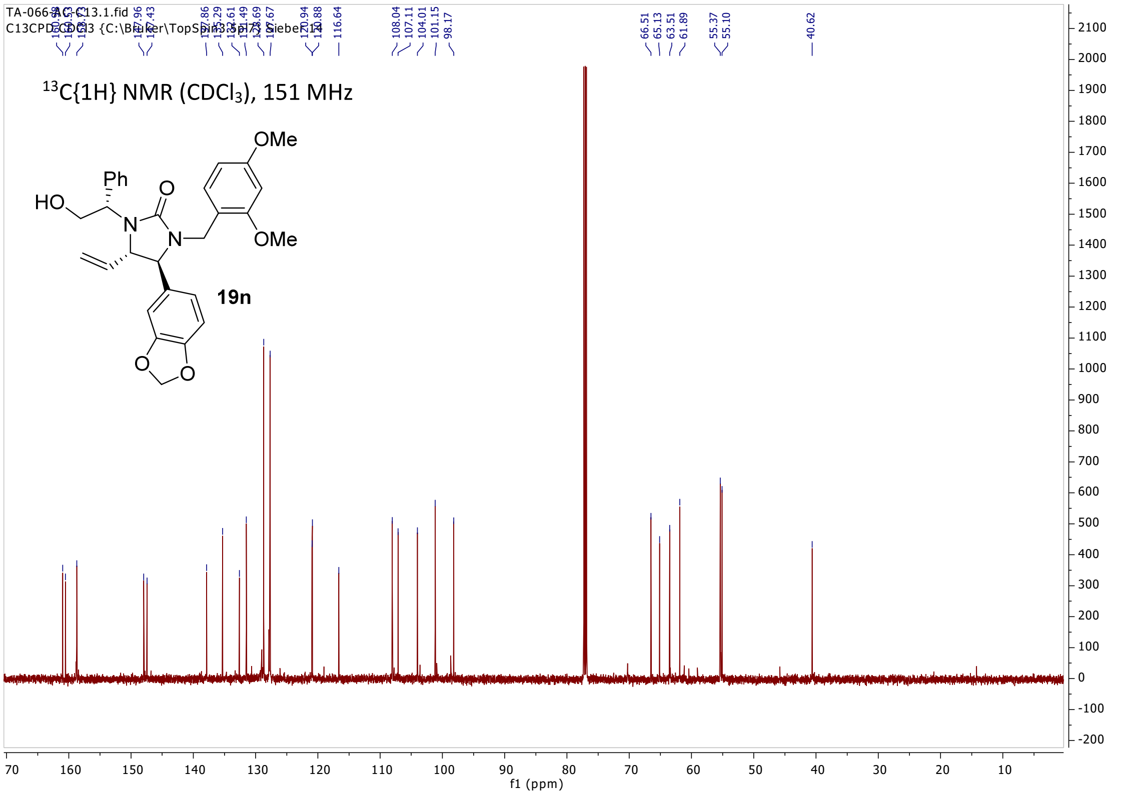
TA-066-AC-C13.1.fid
C13CPD CDCl3 {C:\Bruker\TopSpin3.5pl7} Sieber 14
13C{1H} NMR (CDCl3), 151 MHz
OMe
Ph
HO
O
N
N
OMe
19n
O
O
f1 (ppm)

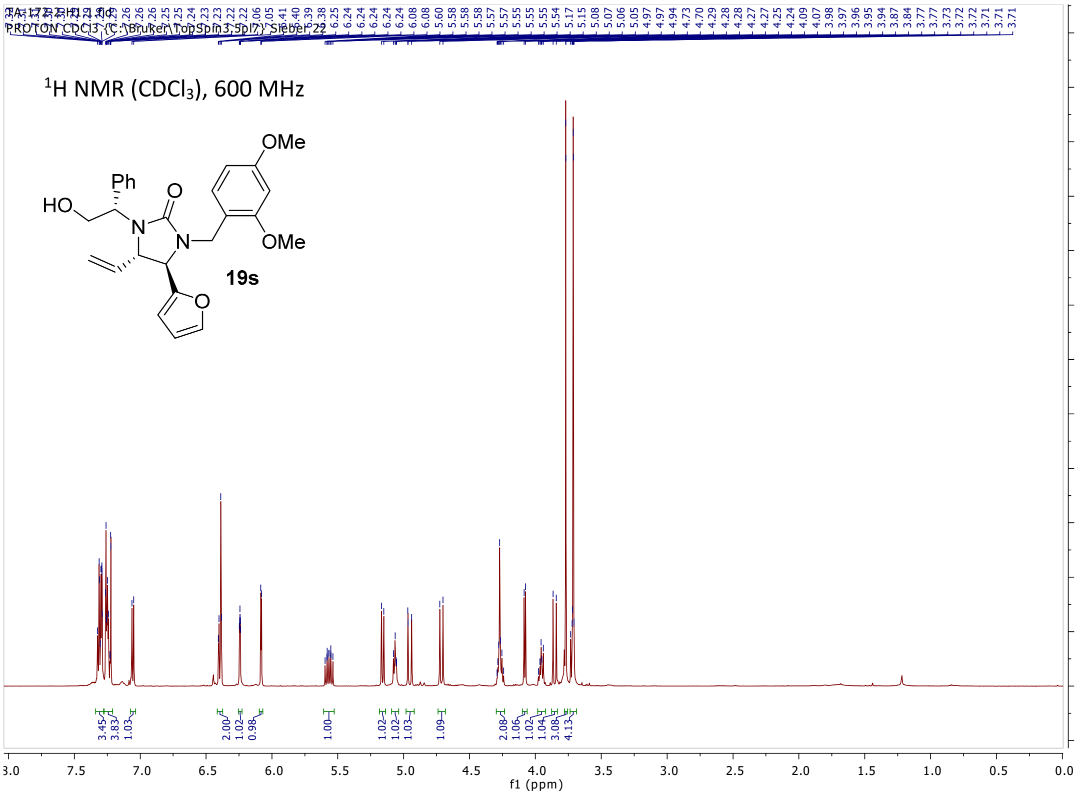

^{1}H NMR (CDCl$_3$), 600 MHz
OMe
Ph
O
HO
N
N
OMe
O
19s
f1 (ppm)

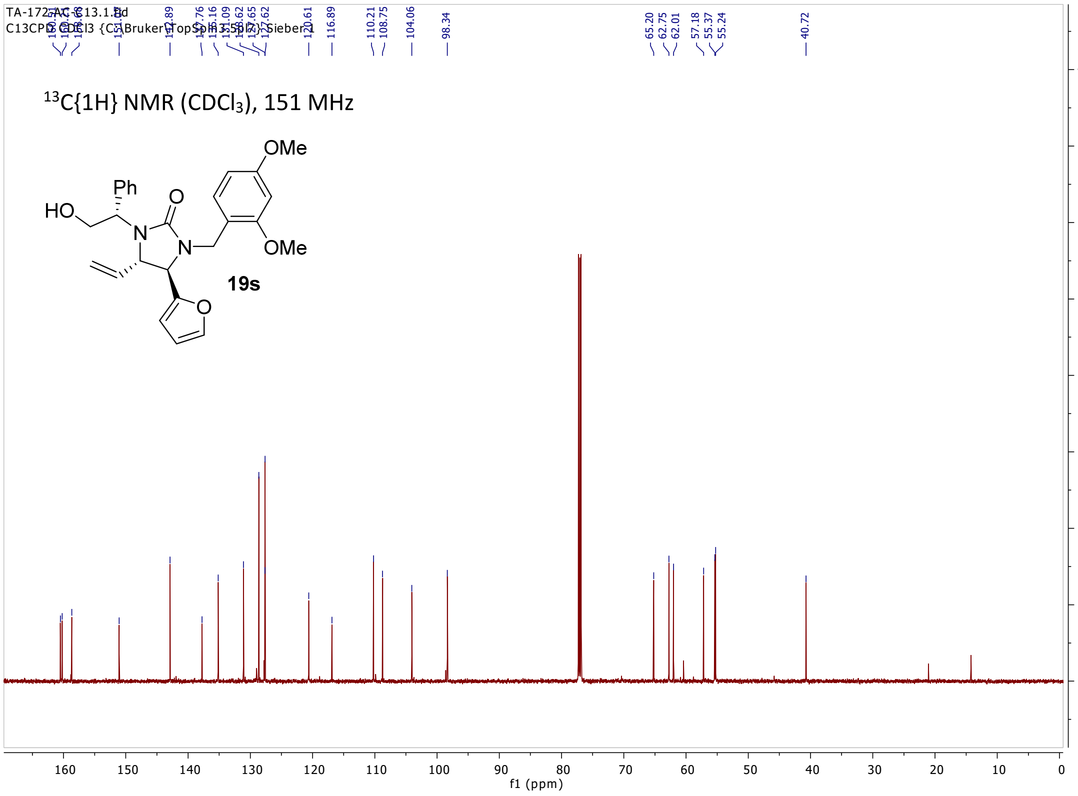

^{13}C{1H} NMR (CDCl$_3$), 151 MHz
OMe
Ph
O
HO
N
N
OMe
O
19s
120.61
116.89
110.21
108.75
104.06
98.34
65.20
62.75
62.01
57.18
55.37
55.24
40.72
f1 (ppm)

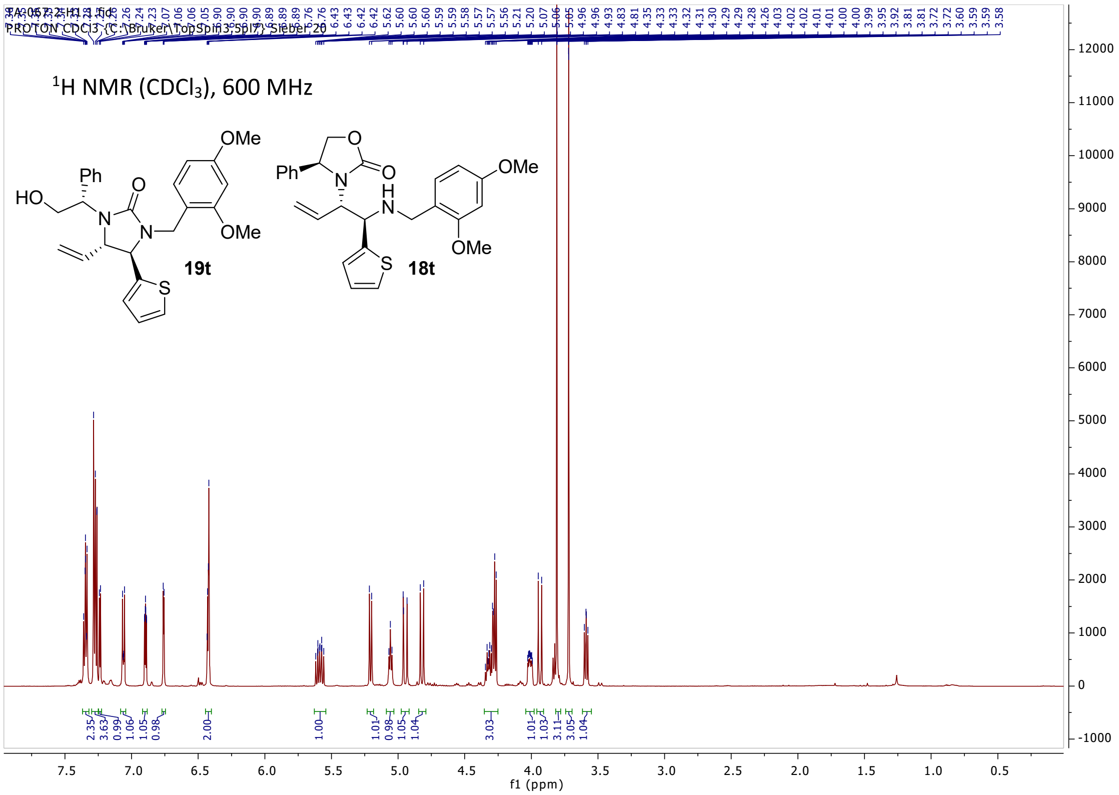

TA-067-2-H1.1.fid
1H NMR (CDCl3), 600 MHz
OMe
Ph
O
HO
N
N
OMe
S
19t
O
O
Ph
N
O
H
N
OMe
OMe
S
18t
2.35
3.63
0.99
1.06
1.05
0.98
2.00
1.00
1.01
0.98
1.05
1.04
3.03
1.01
1.03
3.11
3.05
1.04
f1 (ppm)

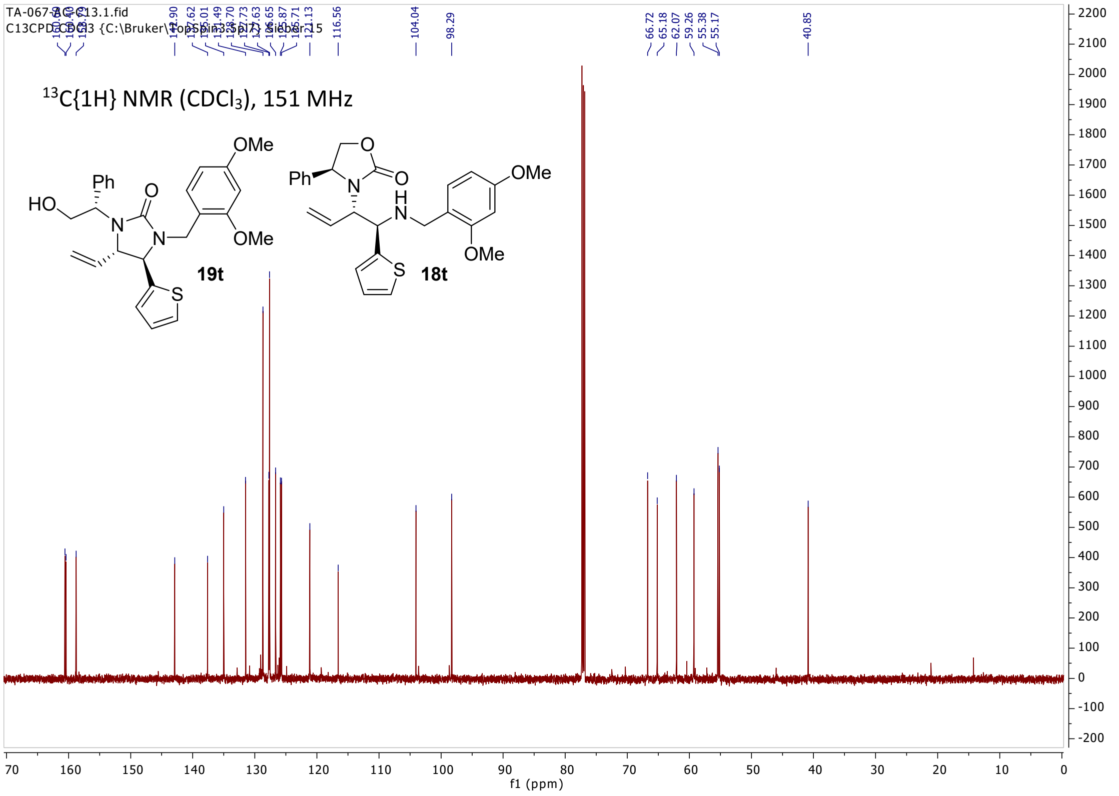

TA-067-2-C13.1.fid
13C{1H} NMR (CDCl3), 151 MHz
OMe
Ph
O
HO
N
N
OMe
S
19t
O
O
Ph
N
O
H
N
OMe
OMe
S
18t
116.56
104.04
98.29
66.72
65.18
62.07
59.26
55.38
55.17
40.85
f1 (ppm)

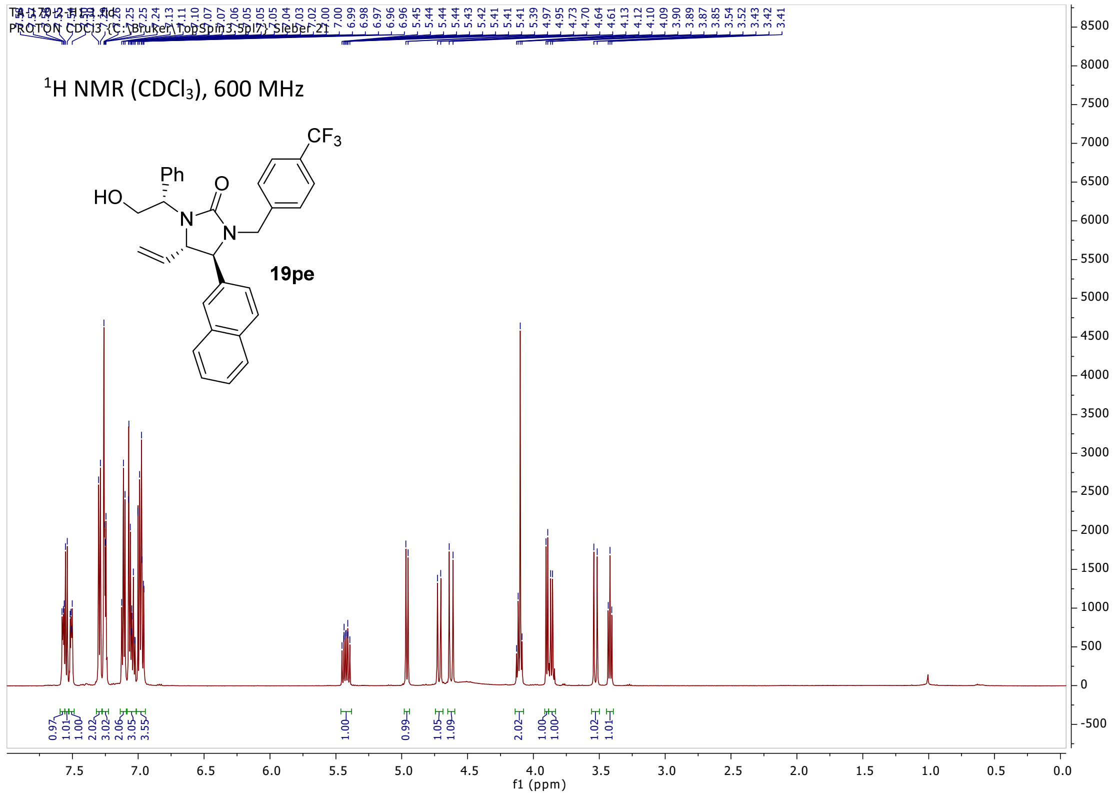
TA-170-2-H1.1.fid
PROTON CDCl3 {C:\Bruker\TopSpin3.5pl7} Sieber 21
^{1}H NMR (CDCl$_3$), 600 MHz
19pe
f1 (ppm)

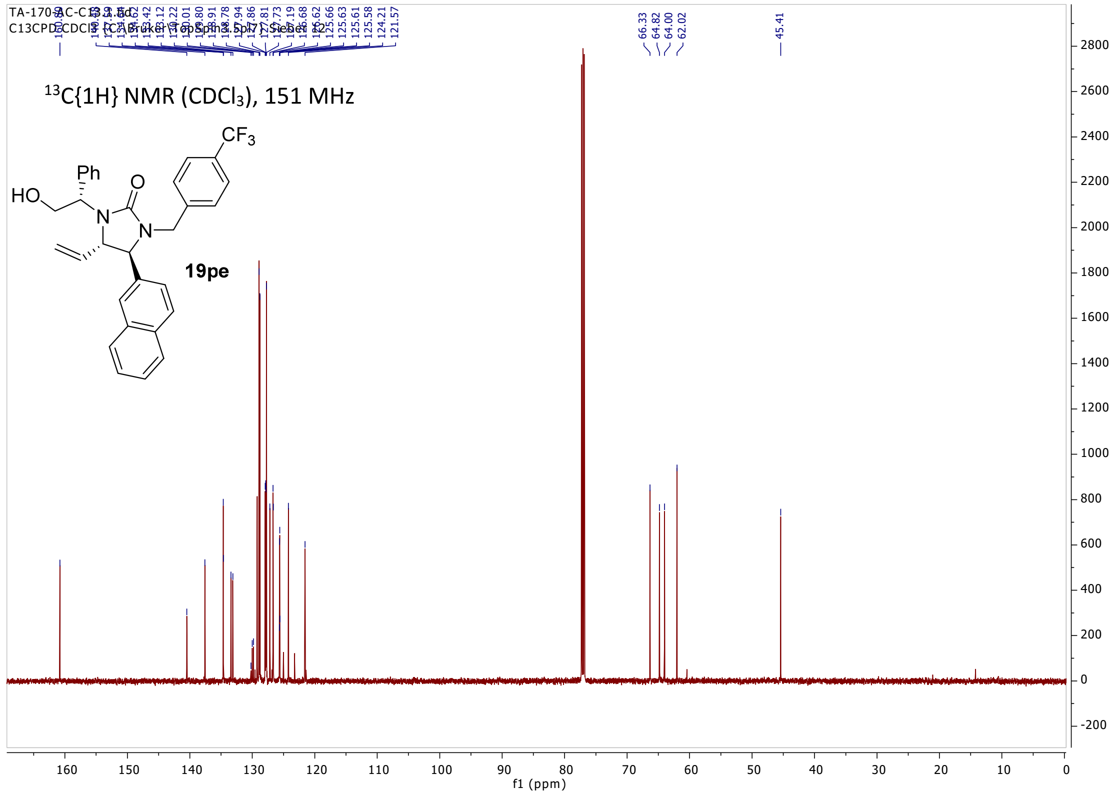
TA-170-AC-C13.1.fid
C13CPD CDCl3 {C:\Bruker\TopSpin3.5pl7} Sieber 12
^{13}C{1H} NMR (CDCl$_3$), 151 MHz
19pe
66.33
64.82
64.00
62.02
45.41
f1 (ppm)

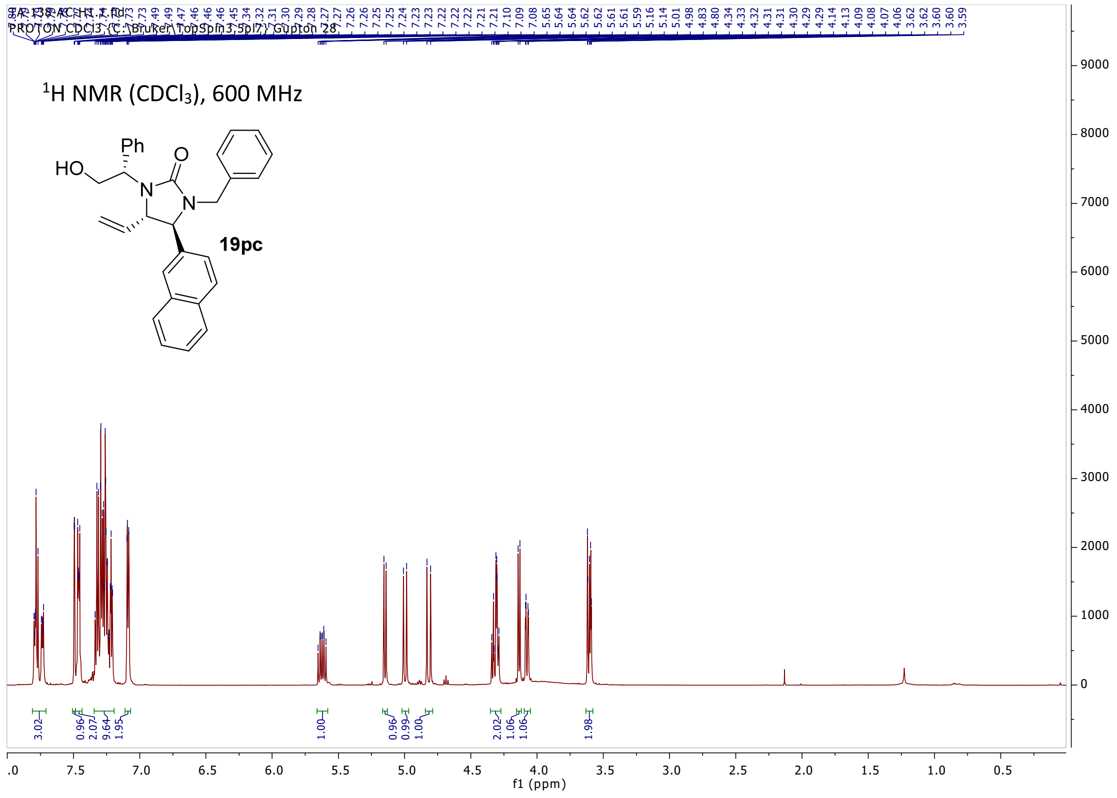

^{1}H NMR (CDCl$_3$), 600 MHz
19pc
f1 (ppm)

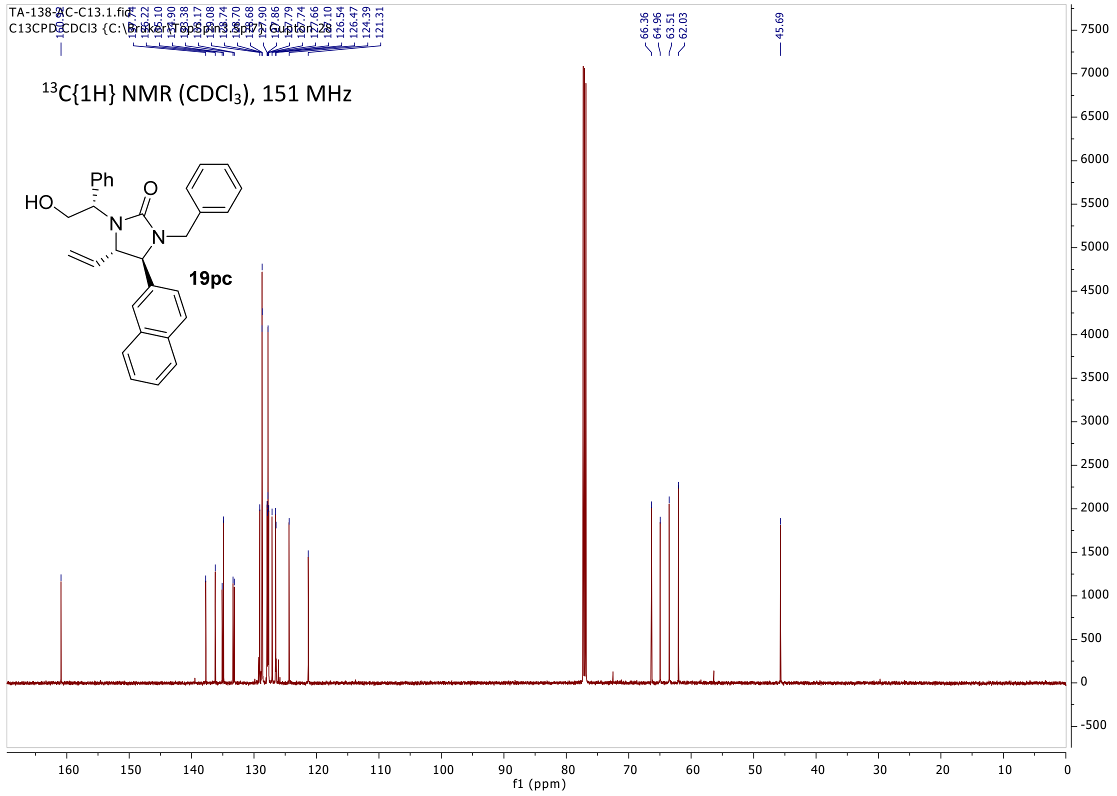

TA-138-AC-C13.1.fid
^{13}C{1H} NMR (CDCl$_3$), 151 MHz
19pc
66.36
64.96
63.51
62.03
45.69
f1 (ppm)

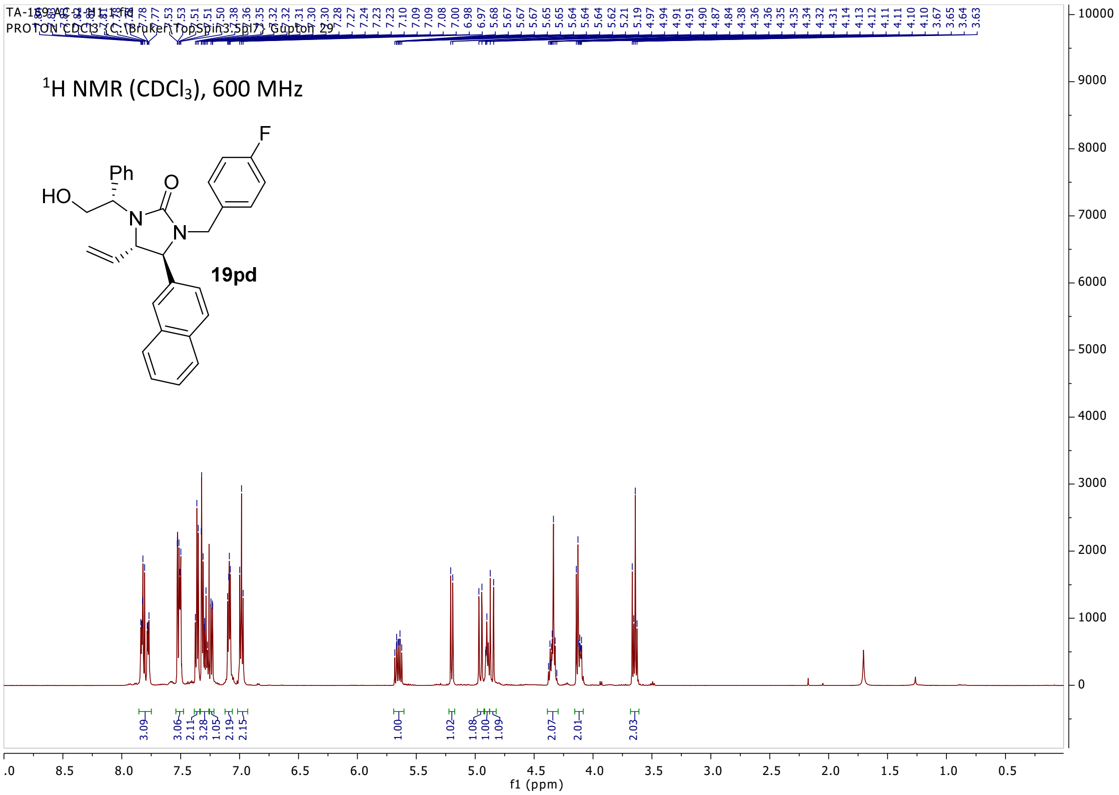
PROTON CDCl3 {C:\Bruker\TopSpin3.5pl7} Gupton 29
¹H NMR (CDCl₃), 600 MHz
19pd
f1 (ppm)

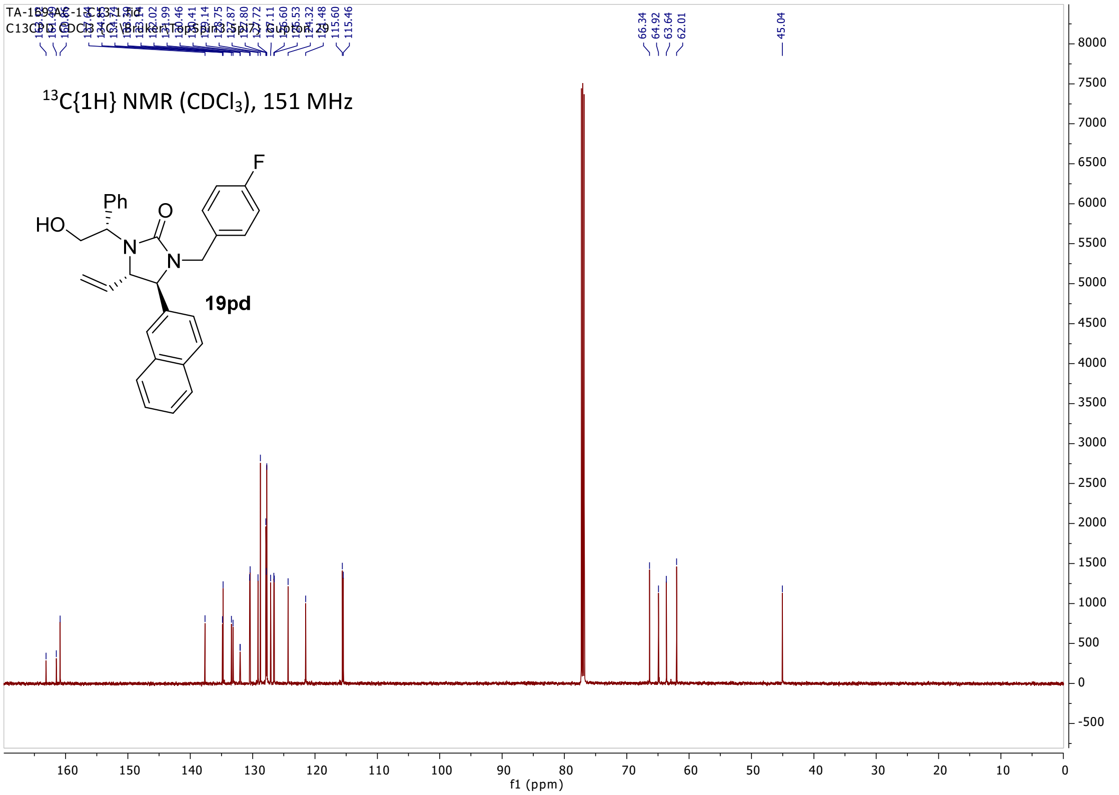
C13CPD CDCl3 {C:\Bruker\TopSpin3.5pl7} Gupton 29
¹³C{1H} NMR (CDCl₃), 151 MHz
19pd
66.34
64.92
63.64
62.01
45.04
f1 (ppm)

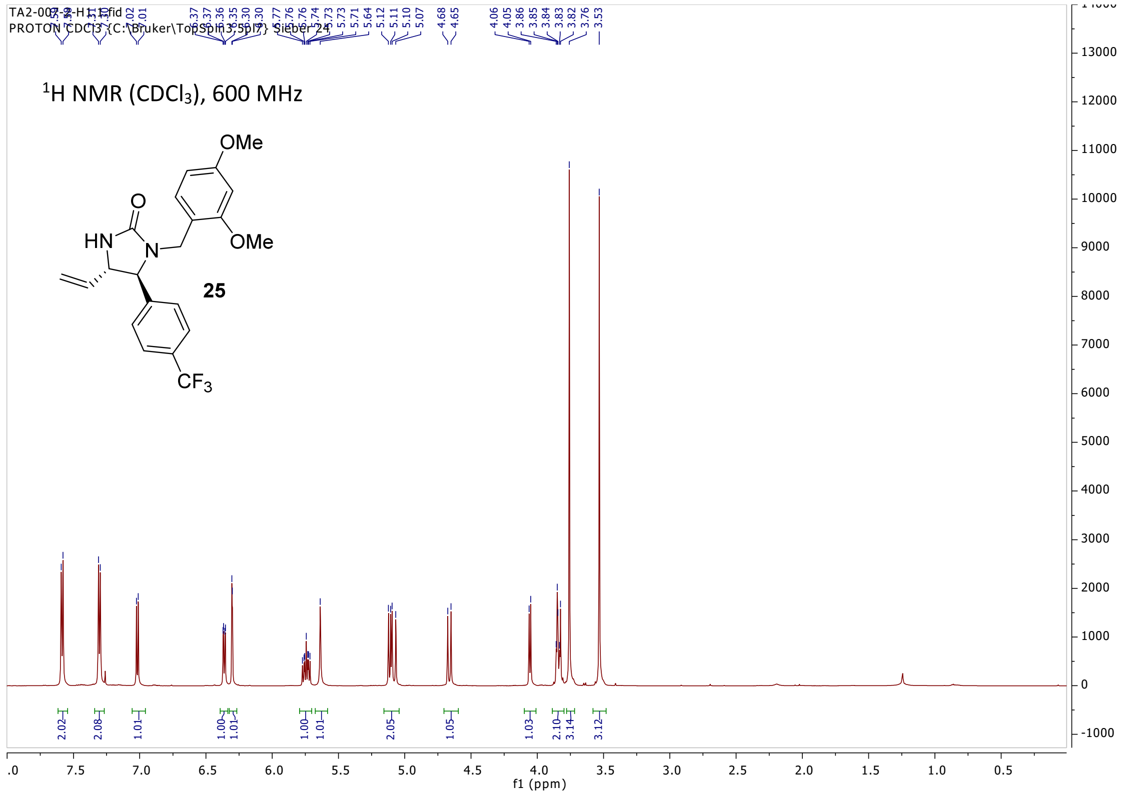

^{1}H NMR ($CDCl_3$), 600 MHz
25
f1 (ppm)

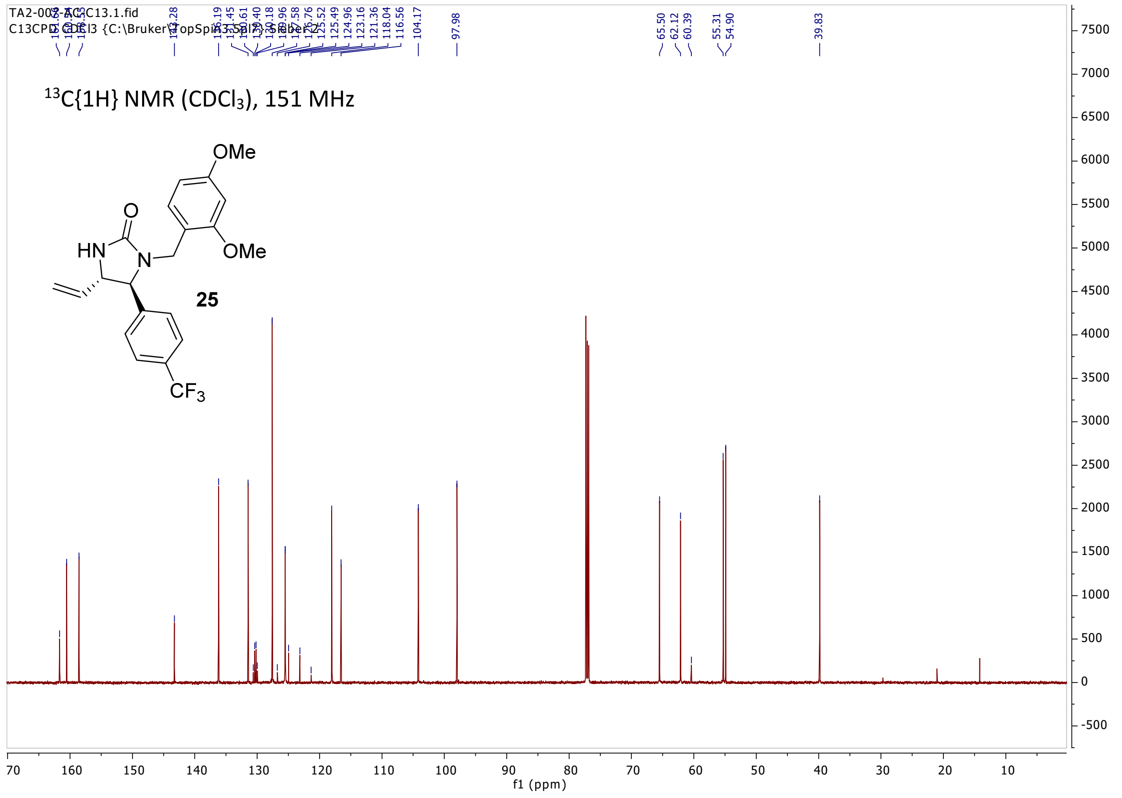

^{13}C{1H} NMR ($CDCl_3$), 151 MHz
25
f1 (ppm)

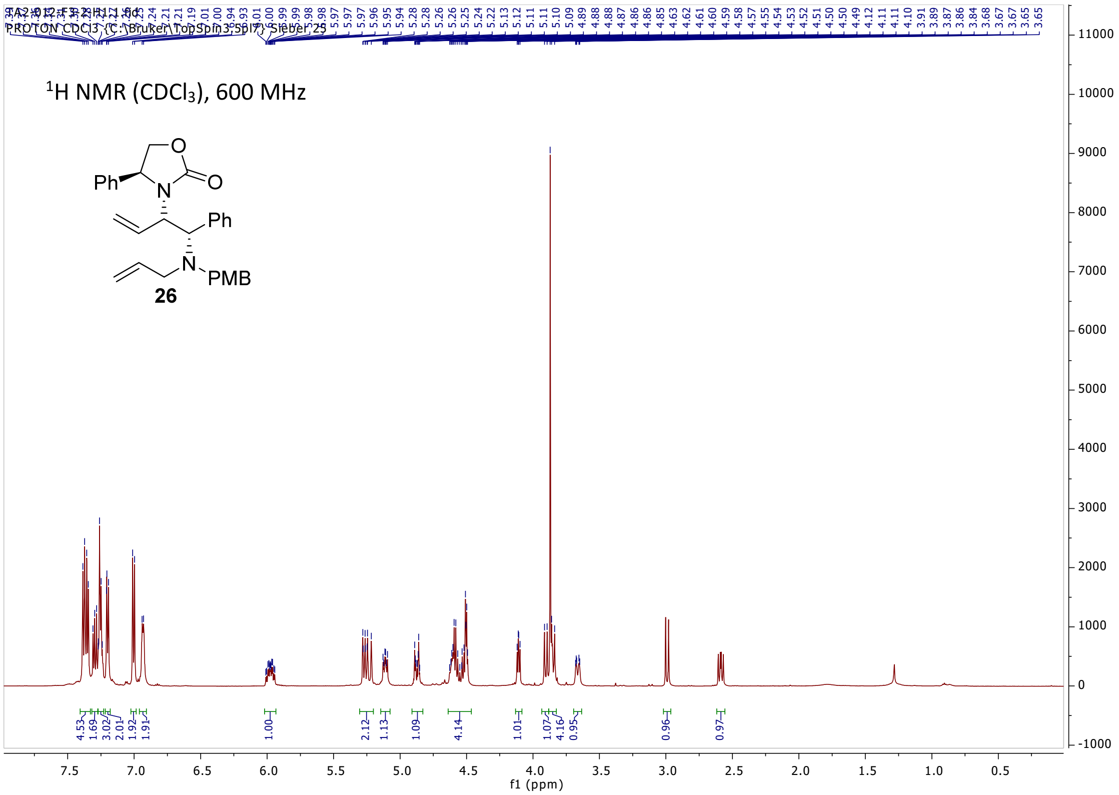

1H NMR (CDCl3), 600 MHz
Ph
N
O
O
Ph
N
PMB
26
f1 (ppm)

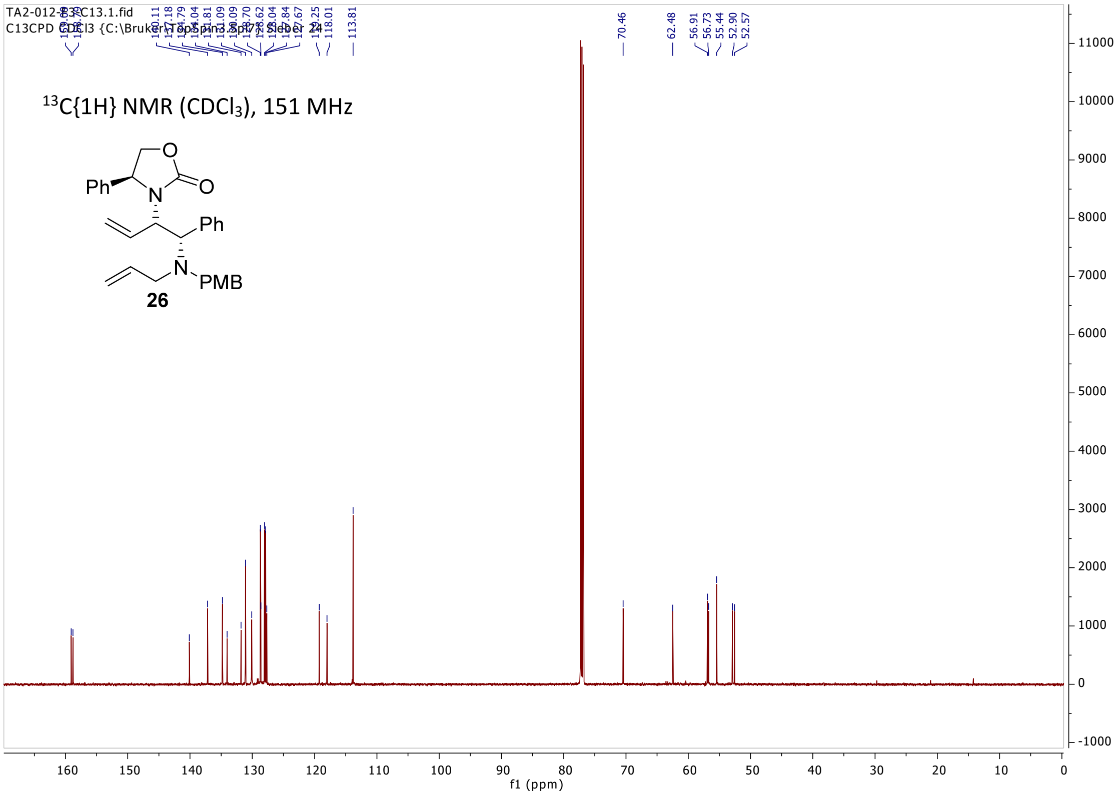

TA2-012-83-C13.1.fid
13C{1H} NMR (CDCl3), 151 MHz
Ph
N
O
O
Ph
N
PMB
26
70.46
62.48
113.81
118.01
f1 (ppm)

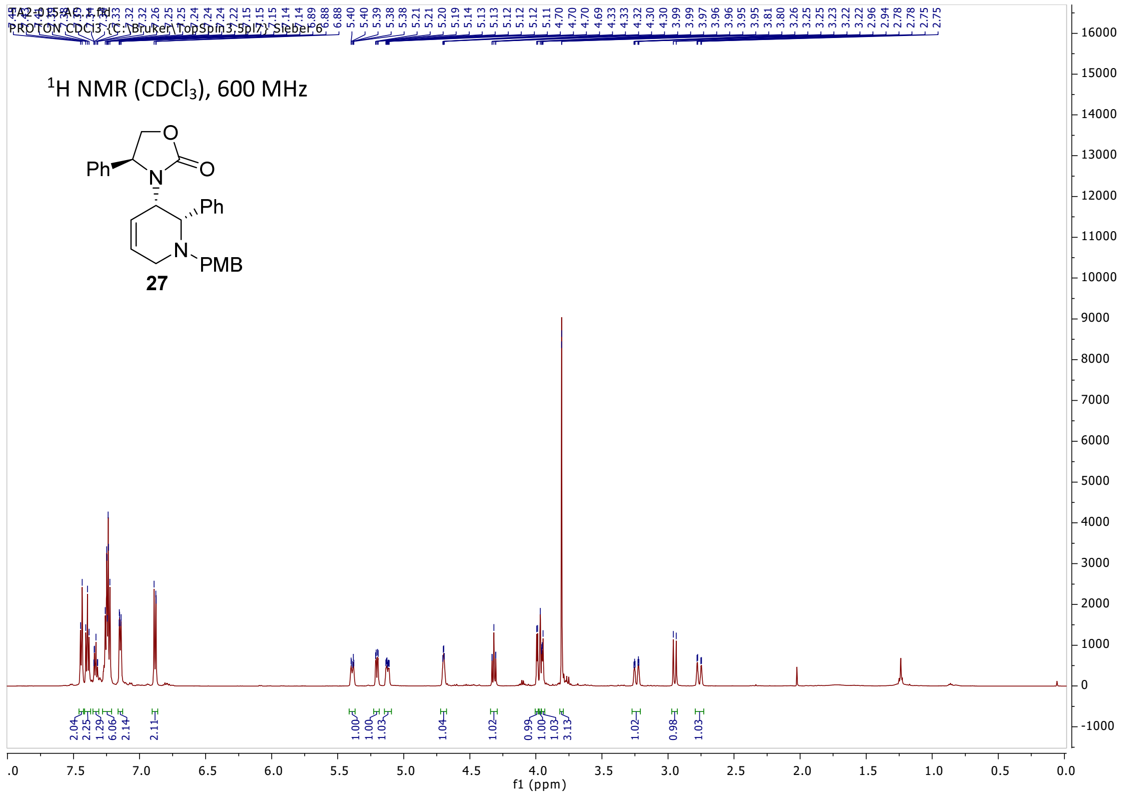

TA2-015-AP.1.fid
PROTON CDCl3 {C:\Bruker\TopSpin3.5pl7} Siebel 6
1H NMR (CDCl3), 600 MHz
Ph
O
O
N
Ph
N
PMB
27
f1 (ppm)

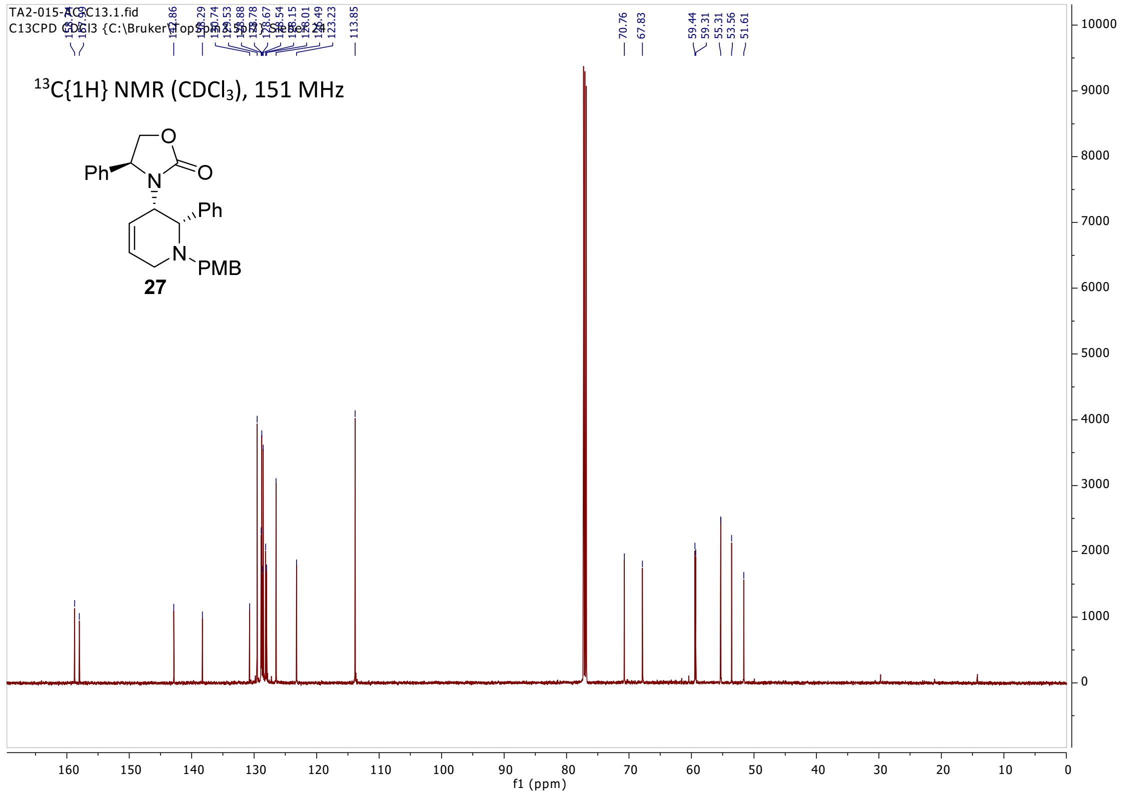

TA2-015-AC.C13.1.fid
C13CPD CDCl3 {C:\Bruker\TopSpin3.5pl7} Siebel 24
13C{1H} NMR (CDCl3), 151 MHz
113.85
70.76
67.83
59.44
59.31
55.31
53.56
51.61
Ph
O
O
N
Ph
N
PMB
27
f1 (ppm)

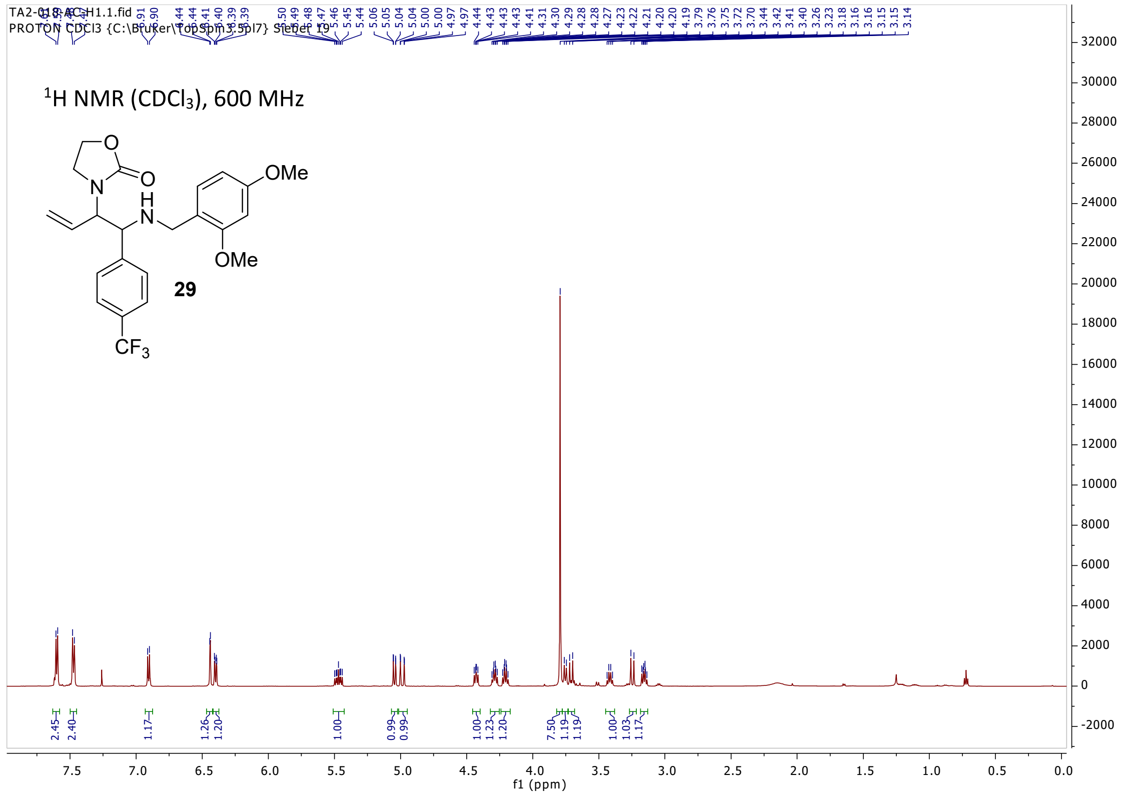
TA2-018-AC.1H1.1.fid
PROTON CDCl3 {C:\Bruker\TopSpin3.5pl7} Sieber 19
1H NMR (CDCl3), 600 MHz
OMe
OMe
29
CF3
f1 (ppm)

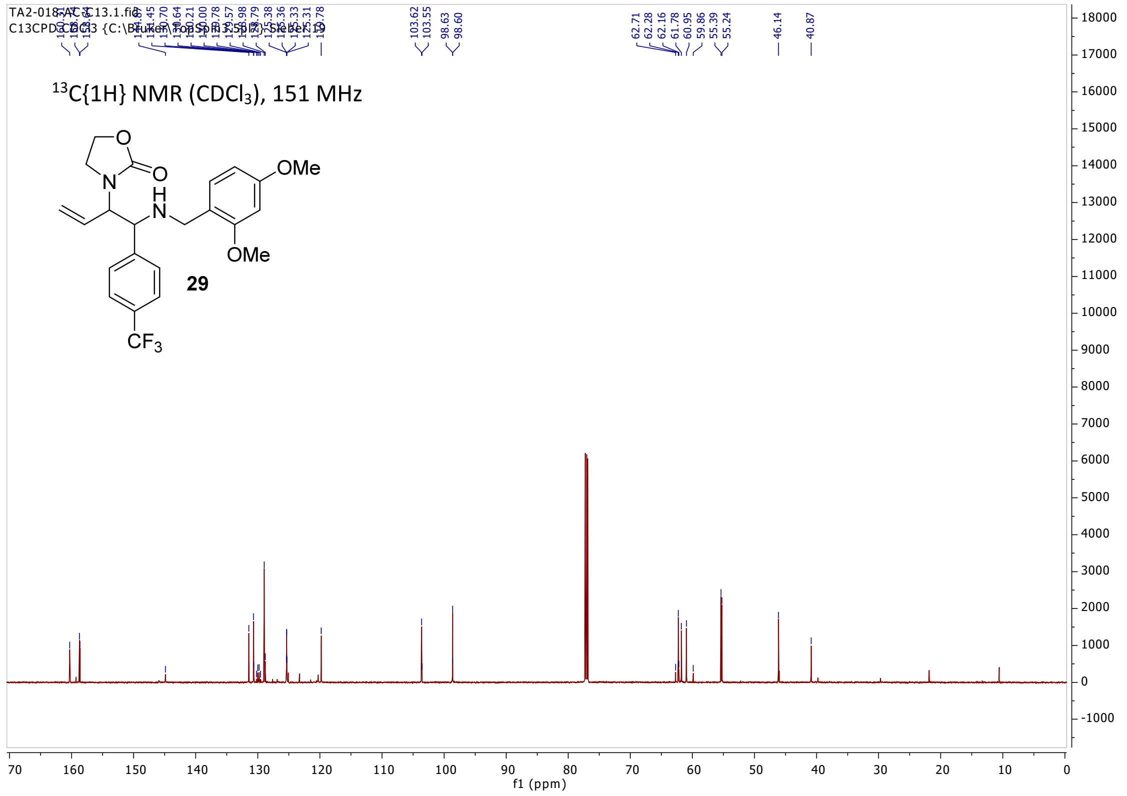
TA2-018-AC.C13.1.fid
C13CPD CDCl3 {C:\Bruker\TopSpin3.5pl7} Sieber 19
13C{1H} NMR (CDCl3), 151 MHz
OMe
OMe
29
CF3
f1 (ppm)

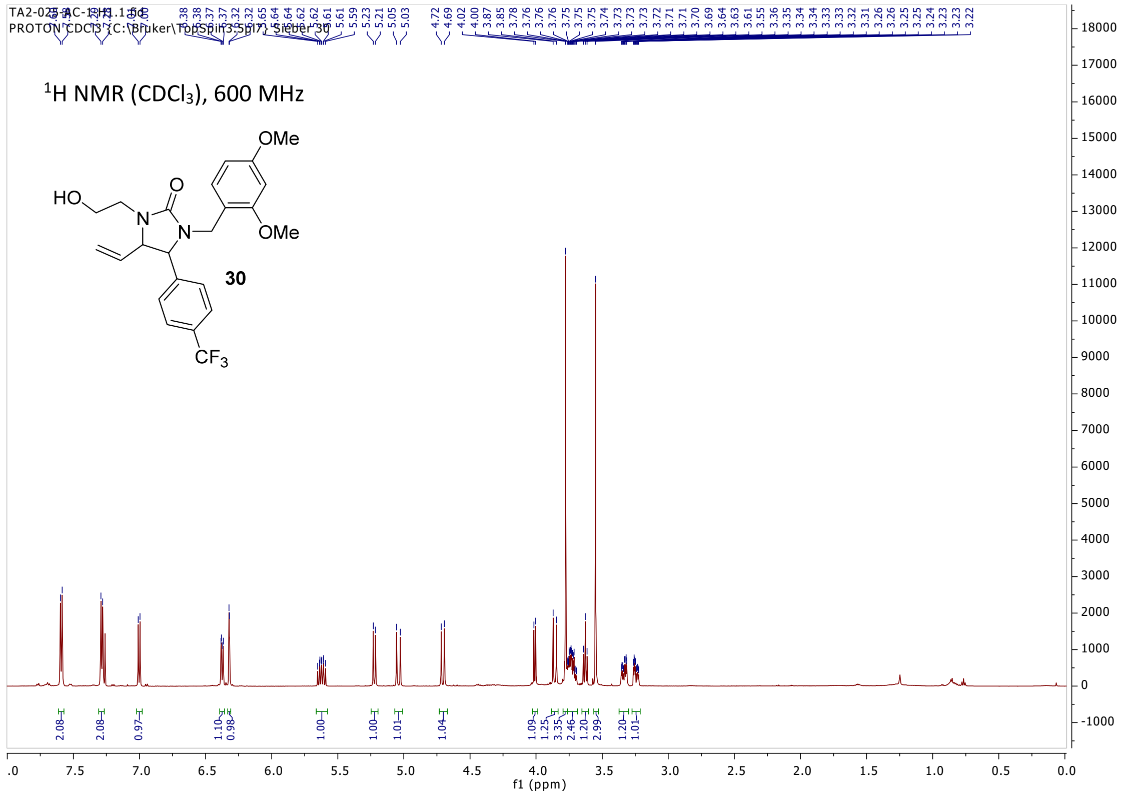

TA2-025-AC-1H1.1.fid
PROTON CDCl3 {C:\Bruker\TopSpin3.5pl7} Sieber 30
^{1}H NMR (CDCl$_3$), 600 MHz
30
f1 (ppm)

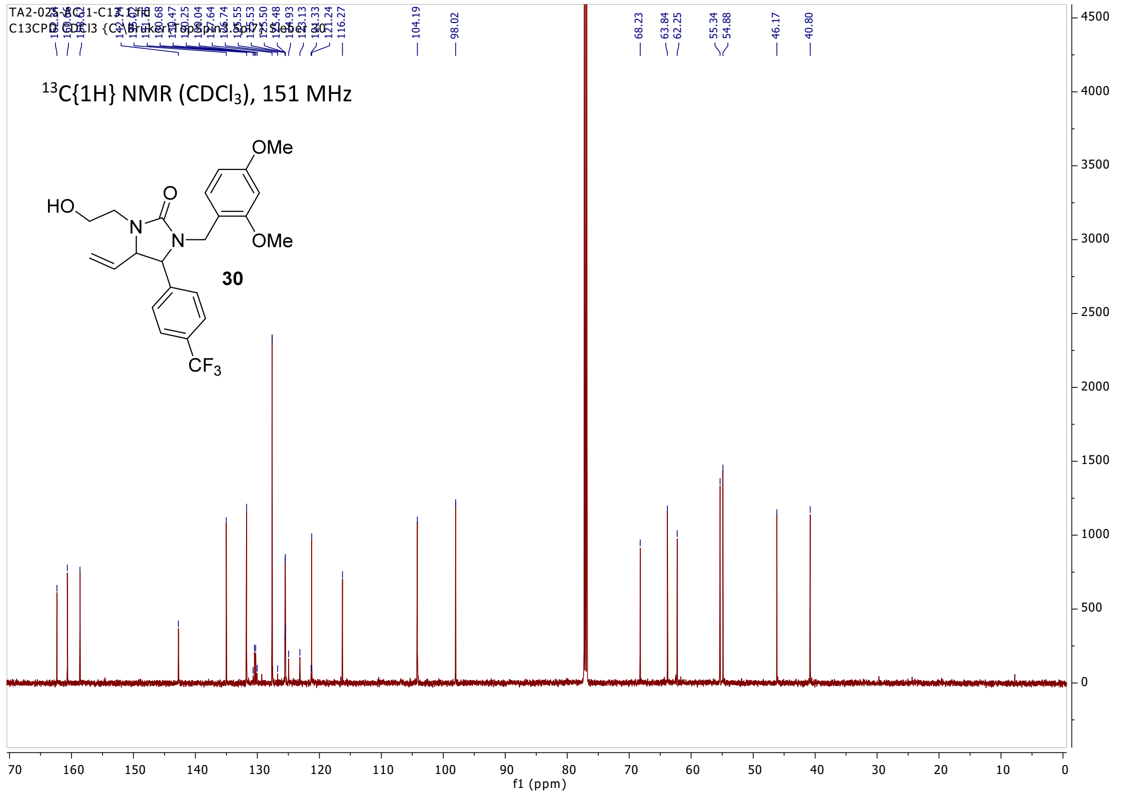

C13CPD CDCl3 {C:\Bruker\TopSpin3.5pl7} Sieber 30
^{13}C{1H} NMR (CDCl$_3$), 151 MHz
30
f1 (ppm)
